# Pd-Catalyzed Strain-Releasing Dyotropic Rearrangement: Ring-Expanding Amidofluorination of Methylenecyclobutanes

**DOI:** 10.1021/jacs.5c01108

**Published:** 2025-03-02

**Authors:** Baochao Yang, Guoqiang Yang, Qian Wang, Jieping Zhu

**Affiliations:** † Laboratory of Synthesis and Natural Products (LSPN), Institute of Chemical Sciences and Engineering, Ecole Polytechnique Fédérale de Lausanne, EPFL-SB-ISIC-LSPN, BCH5304, Lausanne CH-1015, Switzerland; ‡ Shanghai Key Laboratory for Molecular Engineering of Chiral Drugs, Frontiers Science Center for Transformative Molecules, Shanghai Jiao Tong University, 800 Dongchuan Road, Shanghai 200240, China

## Abstract

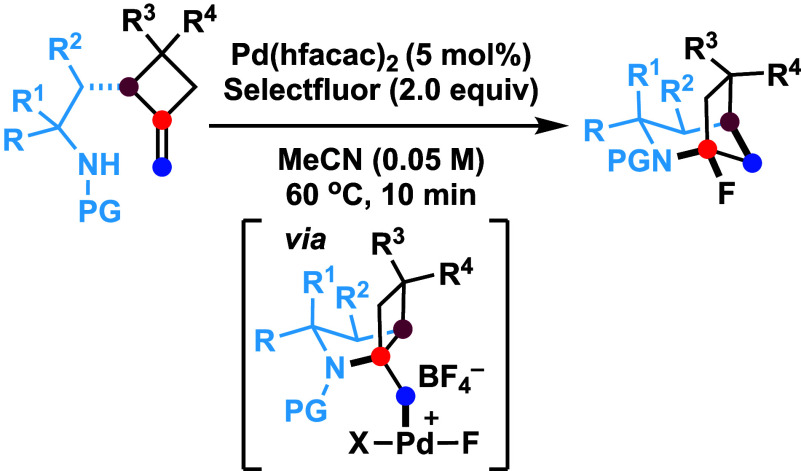

Under the Pd(II)/Pd(IV) catalytic cycle, the cyclization of pent-4-en-1-amine derivatives typically yields either pyrrolidines or piperidines depending on the N-protecting group. We report herein an unprecedented Pd(II)-catalyzed oxidative domino process that converts readily accessible N-protected 2-(2-amidoethyl)-1-methylenecyclobutane derivatives to 1-fluoro-2-azabicyclo[3.2.1]octanes. This transformation constructs three chemical bonds under mild conditions [Pd(hfacac)_2_ (5.0 mol %), Selectfluor (2.0 equiv), MeCN, 60 °C, 10 min] through a domino sequence involving 5-*exo*-trig amidopalladation/Pd(II)–oxidation/chemoselective dyotropic rearrangement/C–F bond-forming reductive elimination. Notably, the cyclization mode remains independent of the N-protecting group under these conditions. Furthermore, diverse functional groups can be introduced at the bridgehead position of a bicyclic compound via an apparent *anti*-Bredt bridgehead iminium intermediate.

## Introduction

Intramolecular amidopalladation-initiated difunctionalization of alkenes, involving Pd(0)/Pd(II) catalytic cycles, has emerged as a powerful strategy for the synthesis of functionalized azaheterocycles.^1−3^ This domino process is typically terminated by C–C bond-forming reductive elimination from the R-Pd(II)-Ar intermediate. However, the reluctance of the alkyl-Pd(II)-X complex to undergo Csp^3^–X bond-forming reductive elimination, coupled with the competitive β*-*hydride elimination of the same Pd(II) species, has made this approach challenging for the introduction of a second heteroatom across the double bond. Although diamination of 1,3-dienes^4,5^ and alkynes^6^ under Pd(0)/Pd(II) and Pd(II)/Pd(0) catalytic conditions, involving Pd(II)-allyl and vinyl-Pd(II) species, respectively, have been successfully achieved, the challenge of Csp^3^–X bond formation remains. To address this limitation, a Pd(II)/Pd(IV) catalytic cycle has been developed, capitalizing on the high-energy Pd(IV) species.^7^ This strategy exploits the facile C–X reductive elimination from the Pd(IV) intermediate and the strong nucleofugal property of Pd(IV). As a result, various transformations, including aminohalogenation,^8^ diamination^9^ and aminoacetoxylation,^10,11^ of alkenes have been successfully realized.

**Scheme 1:**
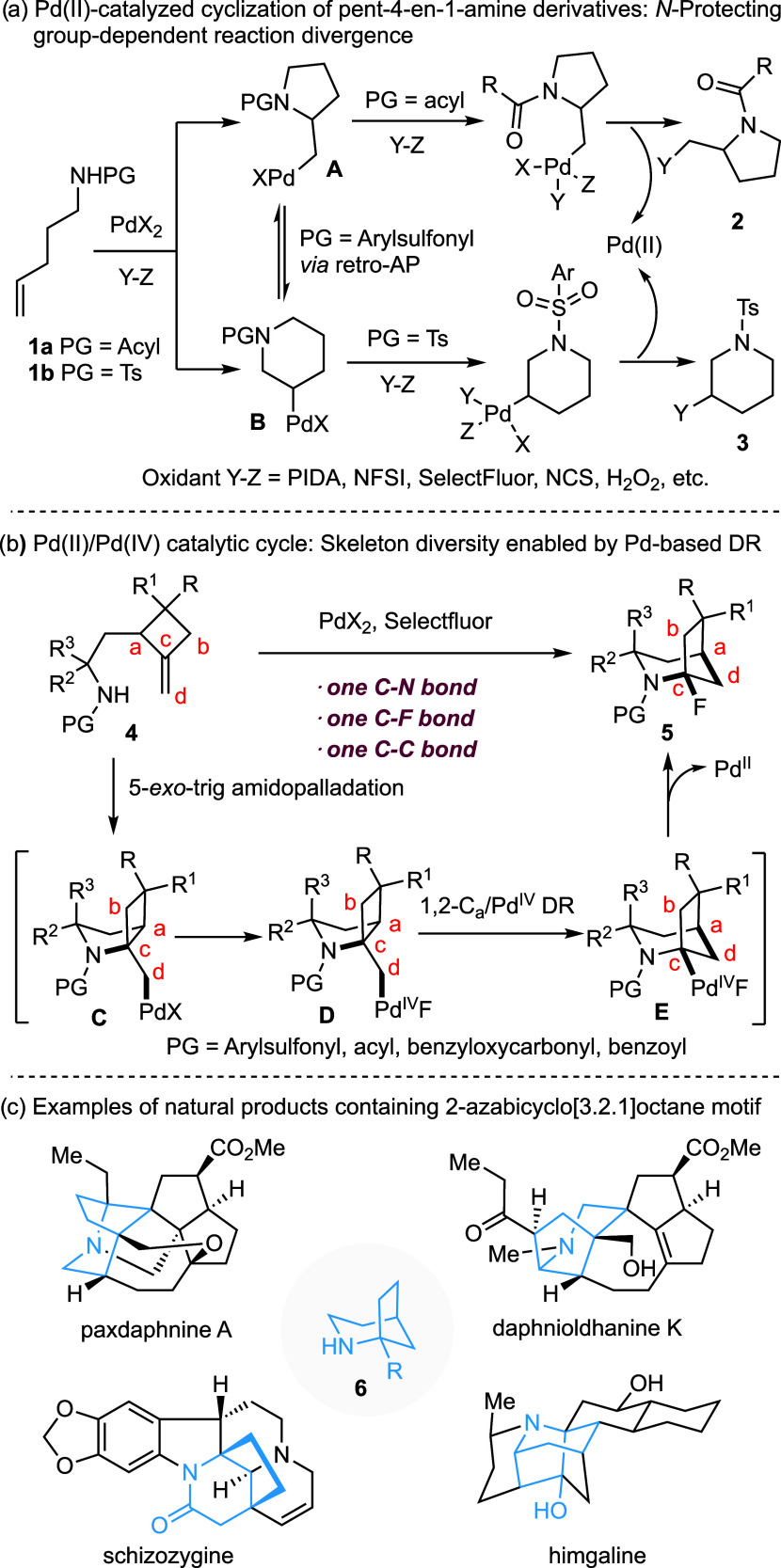
Pd(II)-Catalyzed Cyclization of Pent-4-en-1-amine Derivatives: Reaction Divergence Abbreviations: protecting group (PG), amidopalladation (AP), dyotropic rearrangement (DR), phenyliodine(III) diacetate (PIDA), *N*-fluorobenzenesulfonimide (NFSI), 1-chloromethyl-4-fluoro-1,4-diazoniabicyclo[2.2.2]octane bis(tetrafluoroborate) (Selectfluor), *N*-chlorosuccinimide (NCS).

Under the Pd(0)/Pd(II) catalytic cycle, intramolecular amidopalladation (AP) of pent-4-en-1-amine derivatives **1** typically affords functionalized pyrrolidines through a 5-*exo*-trig cyclization.^1−3^ Interestingly, under Pd(II)/Pd(IV) catalytic conditions, both 5-*exo*-trig and 6-*endo*-trig amidopalladations of **1** have been observed depending on the nature of the N-protecting group. As illustrated in Scheme 1a, the PdX_2_-catalyzed reaction of *N*-acylated derivatives **1a** (PG = acyl, alkoxycarbonyl, aminocarbonyl) in the presence of an oxidant *Y*-*Z* (hypervalent iodine reagent, NFSI, Selectfluor, NCS, H_2_O_2_, etc.) afforded pyrrolidines **2**.^12^ In contrast, the corresponding reaction of the N-Ts derivative (PG = Ts) yielded piperidine derivative **3**.^13^ The formation of **2** from **1a** is proposed to arise from the kinetically favored 5-*exo*-trig cyclization of **1a** and the stabilizing effect of the aminocarbonyl group on the resulting Pd-complex **A** (PG = acyl), attributed to its strong chelating ability. Conversely, the reversible amidopalladation of tosylamide **1b**(14) and faster oxidation of the more electron-rich secondary alkyl-Pd(II) species **B** compared to the primary alkyl-Pd(II) complex **A** are hypothesized to account for the formation of **3** from **1b**, assuming that the Pd(II) oxidation was a rate-determining step.^15^ However, Michael and co-workers have demonstrated that amidopalladation of *N*-acylated pent-4-en-1-amine derivatives in an intramolecular hydroamination of unactivated alkenes can also be reversible.^16^

We have recently discovered a Pd-based dyotropic rearrangement (DR), in which an in situ formed C–Pd(IV) bond undergoes the σ bond metathesis reaction with the vicinal C–C or C–X bond.^17^ Subsequently, our group^18−20^ and others^21,22^ have developed a series of Pd(II)-catalyzed domino processes incorporating this elementary step. Notably, we demonstrated that the conformational property of the Pd(IV) intermediate played a crucial role in determining the chemoselectivity of the migrating group. For instance, in 5-*exo*-trig oxypalladation-initiated domino processes, we successfully directed the reaction toward either 1,2-O/Pd(IV)^19^ or 1,2-C(sp^3^)/Pd(IV)^20^ DR, even though heteroatoms are generally known to exhibit higher migratory aptitude in DR reactions.^23−26^ Intrigued by the aforementioned N-protecting group-depending reaction divergence, we wondered whether dyotropic rearrangement of the Pd(IV) intermediate could be effectively integrated into an amidopalladation-initiated domino process. We report herein that the Pd(II)-catalyzed reaction of N-protected 2-(2-amidoethyl)-1-methylenecyclobutanes **4** with Selectfluor affords 1-fluoro-2-azabicyclo[3.2.1]octanes **5** through the concurrent formation of one C–N, one C–F and one C–C bonds. A plausible reaction pathway is outlined in Scheme 1b. A sequence of 5-*exo*-trig amidopalladation followed by oxidation of the resulting Pd(II) species **C** by Selectfluor would provide Pd(IV) intermediate **D** which, upon regioselective 1,2-C_a_/Pd(IV) DR, would be converted to **E**. A C(sp^3^)-F bond-forming reductive elimination from complex **E** would generate product **5** with the concurrent regeneration of the Pd(II) catalyst. Notably, this reaction proceeds regardless of the nature of the N-protecting group (sulfonyl or acyl). Furthermore, the reaction of **5** with various nucleophiles affords products **6** via an apparent *anti*-Bredt bridgehead iminium intermediate.^27,28^ It is worth noting that the 2-azabicyclo[3.2.1]octane motif **6** is present in various natural products such as paxdaphnine A,^29^ daphnioldhanine K,^30^ schizozygine^31^ and himgaline^32^ etc (Scheme 1c).

## Results and Discussion

### Survey of Reaction Conditions

**Scheme 2:**
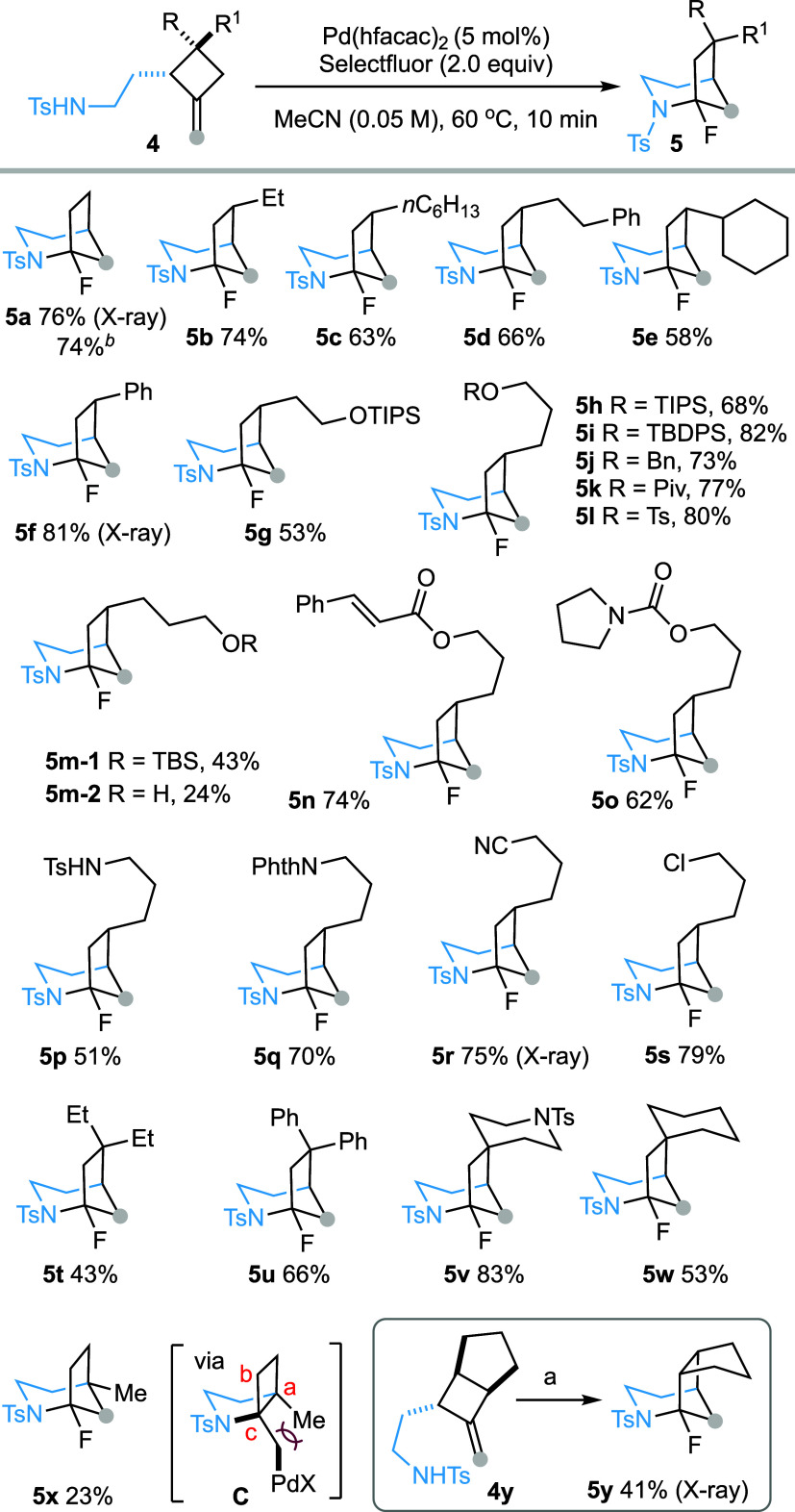
From Methylenecyclobutanes to 2-Azabicyclo[3.2.1]octanes: Scope of Cyclobutane Substitutions Reaction performed at 1.0 mmol scale with 1.0 mol % of Pd(hfacac)_2_, Pd(hfacac)_2_ = palladium hexafluoroacetylacetonate. Reaction conditions: **4** (0.1 mmol), Pd(hfacac)_2_ (5 mol %), and Selectfluor (0.2 mmol) in MeCN (2 mL, *c* 0.05), 60 °C, 10 min.

We selected 2-(2-tosylamidoethyl)-1-methylenecyclobutane (**4a**) as a test substrate to evaluate the reaction conditions (Scheme 2). Gratifyingly, initial experiments on the PdX_2_-catalyzed reaction of *N*-tosyl alkene **4a** with Selectfluor revealed the formation of fluorinated 2-azabicyclo[3.2.1]octane **5a**, albeit in a low yield. Encouraged by these preliminary results, the reaction parameters were systematically surveyed by varying the palladium source, solvent, temperature, and reaction time (See Supporting Information). Ultimately, heating an acetonitrile solution of **4a** (c 0.05 M) and Selectfluor (2.0 equiv) in the presence of a catalytic amount of Pd(hfacac)_2_ (5 mol %) at 60 °C for 10 min afforded compound **5a** in 76% yield (Scheme 2). The structure of **5a** was confirmed by an X-ray crystallographic analysis (CCDC 2417047). Notably, scaling up the reaction to 1 mmol with only 1 mol % of Pd(hfacac)_2_ still produced **5a** in 74% yield, highlighting the practicality of this transformation. It is worth emphasizing that conditions enabling a fast reaction rate are crucial, presumably due to the relative instability of **5a** under slightly acidic conditions (vide infra). Control experiments confirmed that both the palladium catalyst and Selectfluor are essential for the conversion of **4a** to **5a**.

### Reaction Scope

With the optimal reaction conditions in hand [Pd(hfacac)_2_ (5 mol %), Selectfluor (2.0 equiv), CH_3_CN (c 0.05 M), 60 °C], the generality of this reaction was next examined (Scheme 2). The 2,3-*trans*-3-ethyl-2(2-tosylamidoethyl)-1-methylenecyclobutane (**4b**, R = H, R^1^ = Et) was converted regio- and stereoselectively to 6-ethyl-1-fluor-2-tosyl-2-azabicyclo[3.2.1]octane (**5b**) in 74% yield. Other 2,3-*trans*-disubstituted methylenecyclobutanes **4c**–**4s** were similarly transformed to the corresponding bridged bicyclic compounds **5c–5s** in good to high yields. The reaction was tolerant of various functional groups, including silyl ethers (TIPS **5h**; TBDPS **5i**), benzyl ether (**5j**), pivalate (**5k**), tosylate (**5l**), α,β-unsaturated ester (**5n**), carbamoyl (**5o**), sulfonamide (**5p**), phthalimide (**5q**), nitrile (**5r**) and alkyl chloride (**5s**). However, partial deprotection of TBS ether was observed in the cyclization of **4m** (R = H, R^1^ = CH_2_CH_2_CH_2_OTBS) leading to the formation of **5m-1** and **5m-2** in yields of 43 and 24%, respectively. The compatibility of these functional groups provides opportunities for further structural elaborations of the products. In the reaction of 2,3-*trans*-2-tosylamidoethyl-3-tosylamidopropyl-1-methylenecyclobutane (**4p**) leading to **5p**, no product resulting from participation of the 3-tosylamidopropyl group was observed. The 7-*exo*-trig-amidopalladation of this sulfonamide group, which would lead to 2-tosyl-2-azabicyclo[4.1.1]octane, was apparently not competitive compared to the 5-*exo*-trig-amidopalladation of the 2-tosylamidoethyl group, which afforded the 2-tosyl-2-azabicyclo[3.2.0]heptane scaffold. The reaction is applicable to 3,3-disubstituted 2-(2-tosylamidoethyl)-1-methylenecyclobutanes, affording the bridged bicyclic products (**5t**–**5w**). In contrast, 2,2-disubstituted methylenecyclobutane **4x** was converted to bridged bicyclic compound **5x** in only 23% yield. We speculate that severe steric clash between C_a_-Me and C_c_-CH_2_PdX in the intermediate **C** may hamper the initial amidopalladation step or favor the equilibrium toward the starting material. Similarly, a diastereomeric mixture of 2-tosylamidoethyl-4-methyl–methylenecyclobutanes was converted into a mixture of two diastereomeric bicyclic compounds in only 28% yield, whereas 2-tosylamidoethyl-1-methylenespiro[3.5]nonane decomposed under the same conditions (cf, Supporting Information). Finally, 2,3,4-trisubstituted methylenecyclobutane **4y** was converted to **5y**, albeit in moderate yield.

**Scheme 3:**
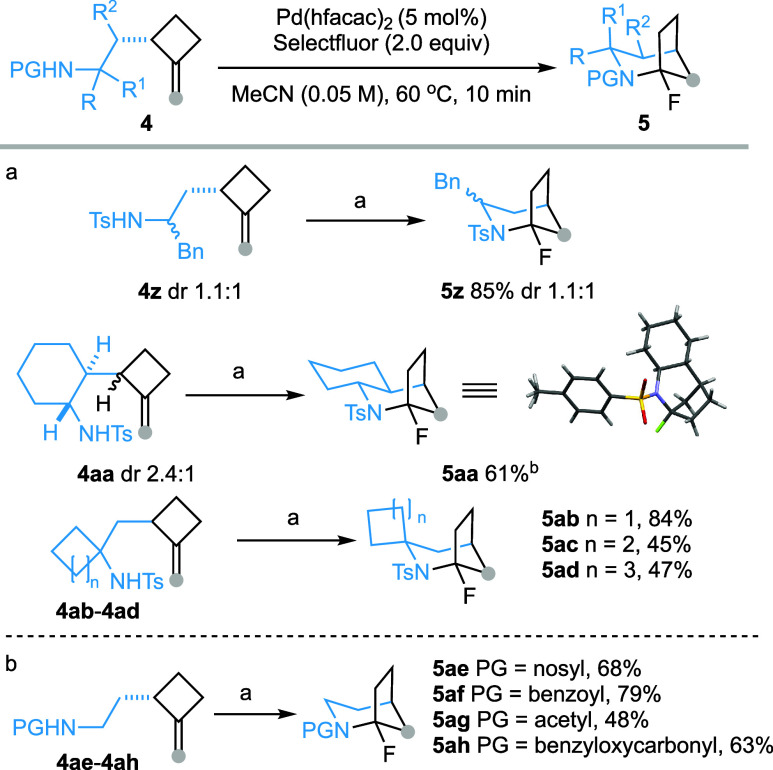
Scope of Substitutions on Amidoethyl Side Chain Only the product resulting from the cyclization of the major diastereoisomer was isolated. Reagents and conditions: **4** (0.1 mmol), Pd(hfacac)_2_ (5 mol %), and Selectfluor (0.2 mmol) in MeCN (2 mL, *c* 0.05), 60 °C, 10 min.

The impact of substitution patterns of the 2-tosylamidoethyl substituent on the reaction outcome was next evaluated. A mixture of two diastereomeric (dr = 1.1:1) α-secondary sulfonamide **4z** (R = H, R^1^ = Bn) was converted into the corresponding aza-bicyclic compound **5z** in 85% yield (Scheme 3a). The α,β-disubstituted tosylamide **4aa** also underwent the reaction smoothly affording tricyclic compound **5aa**. Notably, the α-tertiary sulfonamides proved to be reactive, participating in the reaction to afford **5ab**–**5ad**, which features both bridged and spirocyclic structural motifs. The *N*-(4-nitrophenyl)sulfonamide (*N*-nosyl) effectively initiated the reaction, delivering product **5ae** in 68% yield (Scheme 3b). Interestingly, benzamide (**5af**), acetamide (**5ag**) and carbamate (**5ah**) displayed reactivity comparable to sulfonamide under the current Pd(II)/Pd(IV) catalytic conditions to furnish the 2-azabicyclo[3.2.1]octanes in good to high yields.

**Scheme 4:**
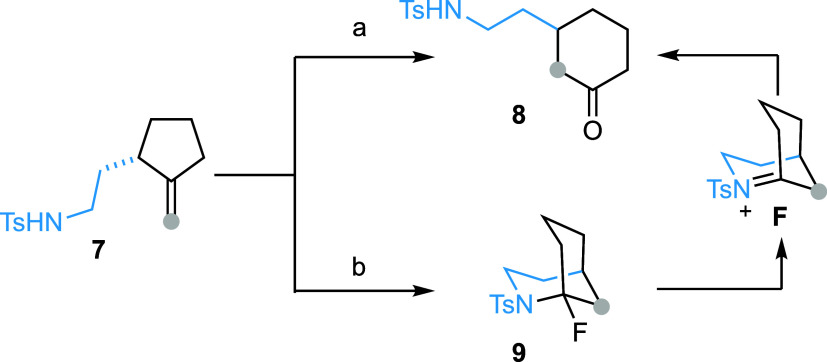
From Methylenecyclopentane to 2-Azabicyclo[3.3.1]nonane Reagents and conditions: **7** (0.1 mmol), Pd(hfacac)_2_ (5 mol %) and Selectfluor (0.2 mmol) in MeCN (2 mL, *c* 0.05), 60 °C, 10 min, flash column chromatography on silica gel, 70%. The same as conditions a, but purification was performed on a basic alumina column, 78%.

Finally, 2-(2-tosylamidoethyl)-1-methylenecyclopentane (**7**) was converted to 2-(2-tosylamidoethyl)-cyclohexan-1-one (**8**) in 70% yield after flash column chromatography on silica gel (Scheme 4). We hypothesized that the same domino process occurred, leading to the formation of 1-fluoro-2-azabicyclo[3.3.1]nonane (**9**). However, this bicyclic compound, bearing an *N*-sulfonylated α-fluoroamine function, was unstable under acidic conditions. It readily underwent hydrolysis during purification via *N*-sulfonyliminium ion intermediate **F**, to afford **8**. The formation of bridgehead iminium ion species from 2-aza-bicyclo[3.3.1]nonanes is known to be much easier than that from 2-aza-bicyclo[3.2.1]octanes **5** (vide supra). On the basis of this assumption, we slightly modified the purification method. Gratifyingly, flash column chromatography of the crude reaction mixture on a basic alumina column allowed us to isolate bridged bicyclic product **9** in 78% yield.

### Mechanistic Studies

**Scheme 5:**
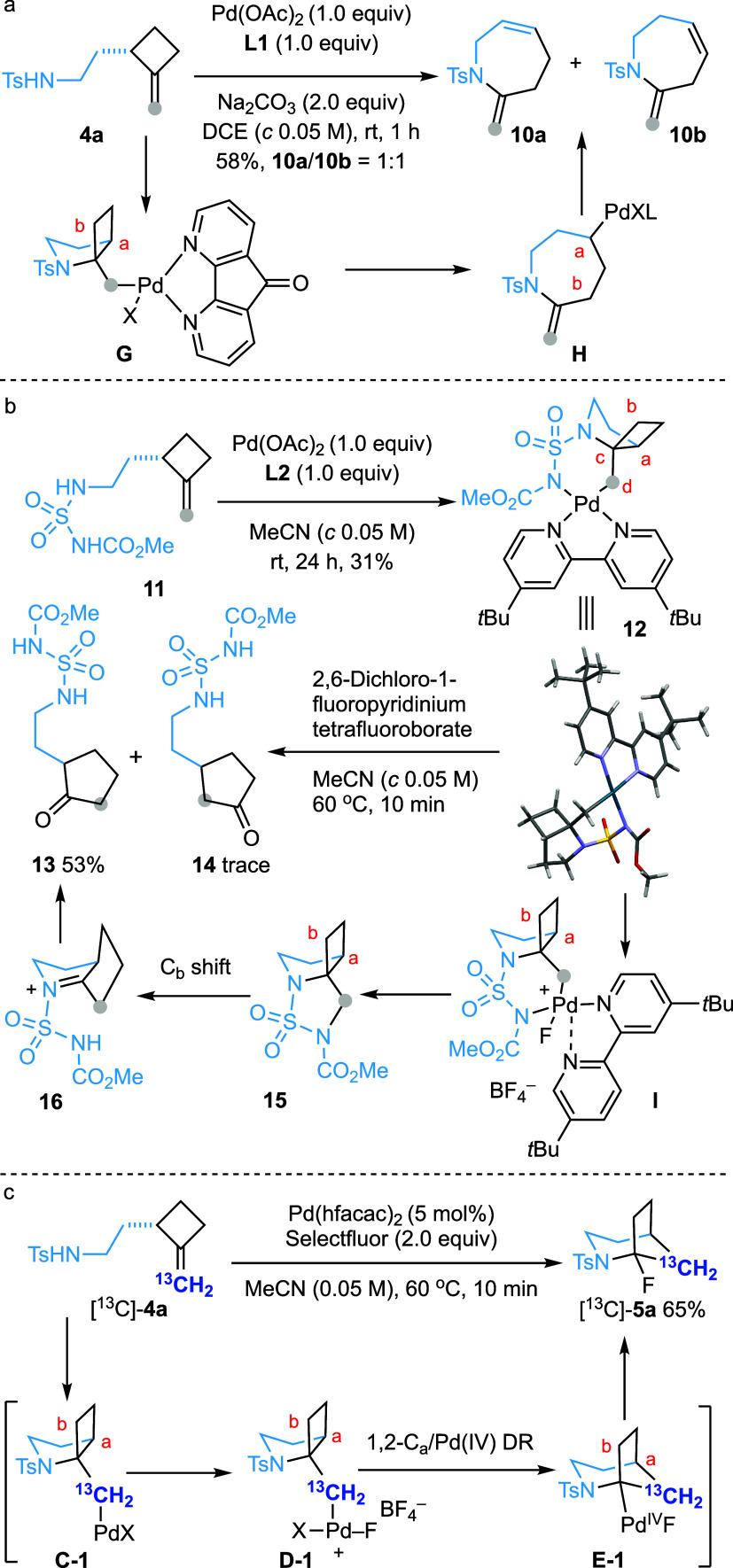
Mechanistic Studies

To gain insight into the reaction mechanism of this catalytic transformation, several experiments were carried out (Scheme 5). Preliminary attempts to prepare the Pd(II) complex by reacting **4a** with diverse ligands in the presence of a stoichiometric amount of Pd(OAc)_2_ were unsuccessful. Interestingly, stirring a DCE solution of **4a** with one equivalent each of Pd(OAc)_2_ and 4,5-diazafluoren-9-one (**L1**) furnished 2-methylene-1-tosyl-tetrahydro-1*H*-azepines **10a** and **10b** as a 1:1 mixture in 58% yield (Scheme 5a). This outcome could be rationalized by a 5-*exo*-trig amidopalladation followed by retro-carbopalladation of the resulting Pd(II) complex **G**(33) and nonregioselective *β-*hydride elimination of **H**, leading to the formation of **10a** and **10b**.

Pleasingly, the reaction of methyl *N*-(2-(2-methylenecyclobutyl)ethyl)sulfamoyl)carbamate (**11**) with Pd(OAc)_2_ and 4,4′-di-*tert*-butyl-2,2′-bipyridine (**L2**) afforded Pd(II) complex **12**, whose structure was confirmed by X-ray crystallographic analysis (Scheme 5b). Heating a MeCN solution of **12** and Selectfluor provided cyclopentanone derivative **13** in 29% yield, along with a trace amount of **14**. Using 2,6-dichloro-1-fluoropyridinium tetrafluoroborate as the oxidant increased the yield of **13** to 53%. In the solid structure of **12**, the dihedral angle of C_a_-C_c_-C_d_-Pd is approximately 170°, while that of C_b_-C_c_-C_d_-Pd is 66°. Consequently, if the corresponding Pd(IV) species underwent a dyotropic rearrangement, C_a_ would be expected to migrate preferentially leading, after hydrolysis, to the formation of **14** as a major product instead of **13**. Therefore, an alternative mechanism might be operating. We surmised that the formation of **13** would involve the C–N bond-forming reductive elimination from Pd(IV) intermediate **I** to generate **15**,^9^ which could then undergo an aza-pinacol rearrangement. In this scenario, C_b_ migration would be favored over C_a_ since C_a_ migration would result in a bridgehead iminium species. Subsequent hydrolysis of **16** would then produce observed product **13**.

Finally, submitting compound [^13^C]-**4a**, labeled at the terminal sp^2^ carbon, to the standard conditions afforded [^13^C]-**5a** in 65% yield (Scheme 5c). The formation of [^13^C]-**5a** is consistent with a reaction sequence involving 5-*exo*-trig amidopalladation followed by Pd oxidation, chemoselective 1,2-C_a_/Pd(IV) dyotropic rearrangement, and C–F bond forming reductive elimination.

### Post-Transformations

**Scheme 6:**
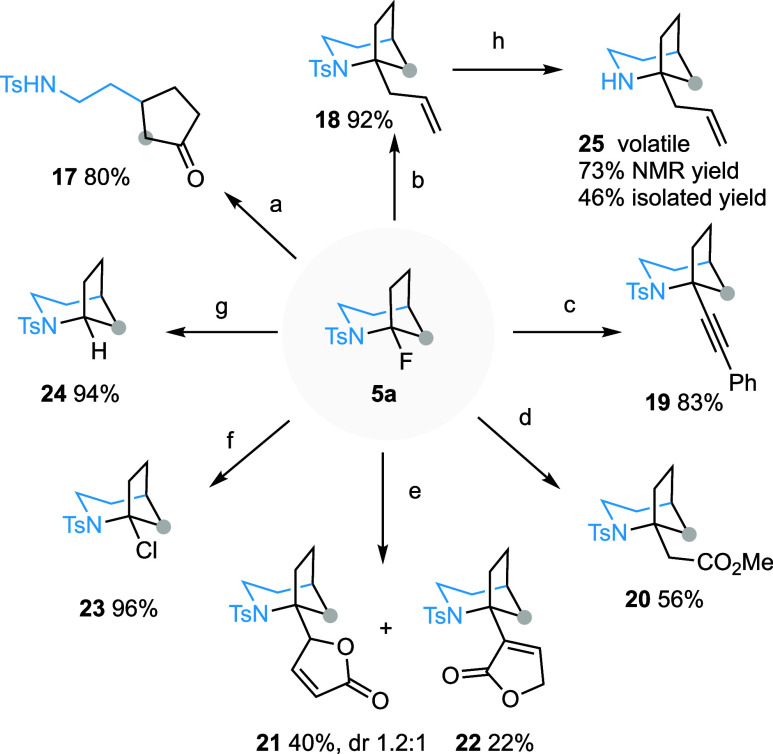
Chemical Transformation of 1-Fluoro-2-azabicyclo[3.2.1]octanes Reagents and conditions: (a) TFA (5 equiv), DCM, rt, 10 h, 80%. (b) BF_3_**·**Et_2_O (3.0 equiv), allyltrimethylsilane (5.0 equiv), DCM, 0 °C, 2.5 h, 92%. (c) BF_3_**·**Et_2_O (3.0 equiv), potassium trifluoro(phenylethynyl)borate (2.0 equiv), TBAB (0.1 equiv), DCM, 0 °C, 1 h, 83%. (d) BF_3_**·**Et_2_O (3.0 equiv), (1-methoxyvinyl)oxy](trimethyl)silane (25 equiv), DCM, rt, 1.5 h, 56%. (e) BF_3_**·**Et_2_O (3.0 equiv), 2-(trimethylsilyloxy)furan (2.0 equiv), DCM, −10 °C, 1.5 h, 40% (dr 1.2:1) for **21**, 22% for **22**. (f) TiCl_4_ (2.0 equiv), DCM, 0 °C, 1.5 h, 96%. (g) BF_3_**·**Et_2_O (5.0 equiv), Et_3_SiH (10.0 equiv), DCM, rt, 2 h, 94%. (h) SmI_2_ (10.0 equiv), pyrrolidine (20.0 equiv), H_2_O (30 equiv), THF, rt, 1 h, 46% isolated yield, 73% NMR yield. Abbreviations: TFA = trifluoroacetic acid, DCM = dichloromethane, TBAB = tetrabutylammonium bromide, THF = tetrahydrofuran.

The presence of an *N*-sulfonylated α-fluoroamine function in 1-fluoro-2-azabicyclo[3.2.1]octanes offers a unique opportunity to introduce diverse functional groups at the C1 position. This is particularly compelling, as all of the natural products depicted in Scheme 1c feature an α-tertiary amine motif. Gratifyingly, stirring a DCM solution of **5a** with TFA (5.0 equiv) at room temperature afforded cyclopentanone derivative **17** in 80% isolated yield. The apparent facile generation of the bridgehead iminium species from this bicyclic compound prompted us to exploit the reactivity of this latent electrophilic species.^27^ As shown in Scheme 6, the BF_3_**·**Et_2_O-promoted reaction of **5a** with allylsilane generated the 1-allylated derivative **18** in 92% yield. Similarly, alkynyl group was introduced at the bridgehead position of **19** by employing alkynyltrifluoroborate^34^ as the nucleophile. When an enol ether was used as the nucleophile, an alkyl group can in turn be introduced at the C1 position of the bicyclic compounds (**20**–**22**). In the reaction with 2-(trimethylsilyloxy)furan, two products **21** and **22**, resulting from the alkylation of the C-3 and C-5 positions of furan, were generated in the yield of 40% and 22%, respectively. Halogen exchange reactions were also possible. Treating **5a** with TiCl_4_ (2.0 equiv, DCM, 0 °C) afforded 1-chloro derivative **23** in 96% yield. Additionally, reduction of **5a** with triethylsilane in the presence of BF_3_**·**Et_2_O delivered 2-tosyl-2-azabicyclo[3.2.1]octane (**24**) in an excellent yield. Finally, removal of *N*-tosyl protecting group from **18** was realized under single electron transfer reductive conditions yielding volatile 1-allyl-2-azabicyclo[3.2.1]octane **25**.^35^

## Conclusions

In summary, we have developed a Pd(II)-catalyzed amidopalladation-initiated domino process that efficiently converts readily accessible *N*-protected 2-(2-amidoethyl)-1-methylenecyclobutane derivatives **4** to 1-fluoro-2-azabicyclo[3.2.1]octanes **5**. This transformation relies on a ring-expanding, chemoselective 1,2-Csp^3^/Pd(IV) dyotropic rearrangement. Additionally, the facile generation of the bridgehead iminium intermediate from **5** in the presence of a Lewis acid enabled the successful introduction of diverse functional groups at the bridgehead position of the bicyclic scaffold.

## Supporting Information Available

Experimental procedures and characterization data; additional experimental details; crystallographic data of **5a**, **5f**, **5r**, **5y**, **5aa** and **12**; copies of the ^1^H, ^13^C and ^19^F NMR spectra (PDF)
