# Supplementary material for: Pd-Catalyzed Strain-Releasing Dyotropic Rearrangement: Ring-Expanding Amidofluorination of Methylenecyclobutanes
Source: J Am Chem Soc. 2025 Mar 2;147(10):8969–77. doi: 10.1021/jacs.5c01108 (PMC11925333; doi:10.1021/jacs.5c01108)
Supplement: Supplementary file 1 — ja5c01108_si_001.pdf [file ja5c01108_si_001.pdf]

Supporting Information

Pd-Catalyzed Strain-Releasing Dyotropic Rearrangement: Ring-Expanding Amidofluorination of Methylenecyclobutanes

Baochao Yang,[1] Guoqiang Yang,[1,2] Qian Wang,[1] and Jieping Zhu[1,]*

[1] Laboratory of Synthesis and Natural Products (LSPN), Institute of Chemical Sciences and Engineering, Ecole Polytechnique Fédérale de Lausanne, EPFL-SB-ISIC-LSPN, BCH5304, CH-1015 Lausanne, Switzerland.

[2] Shanghai Key Laboratory for Molecular Engineering of Chiral Drugs, Frontiers Science Center for Transformative Molecules, Shanghai Jiao Tong University, 800 Dongchuan Road, Shanghai 200240, China.

*Corresponding author: Jieping Zhu, jieping.zhu@epfl.ch

Table of Contents

I. General information……………………………………..………………………..S2

II. Optimization studies ………………………………….……………………S3

III. Synthesis of substrates ………….……….……………………….….……..S5

IV. Synthesis of 1-fluorinated 2-azabicyclo[3.2.1]octane: substrate scope…........S38

V. Mechanistic Studies………………...……………………..…………..……...S59

VI. Synthetic transformation…………….…………...……………………....S68

VII. X-ray crystallographic data………………………………..……………..….....S77

VIII. Copies of the NMR spectra……………………………….……………........S120

IX. References………………………………………….…………..……........S231

I. General information

All reactions were carried out with anhydrous solvents unless noted. Chemicals were purchased from commercial sources (Aldrich, Acros, Alfa Aesar, Merck, TCI, Fluorochem, Abcr, etc.) and used without further purification unless otherwise indicated. Solvents were purchased in HPLC quality, degassed by purging thoroughly with nitrogen and dried over activated molecular sieves of appropriate size. Alternatively, they were obtained using a solvent purification system with an aluminum oxide column (Innovative Technologies). Flash column chromatography was performed using SiliCycle UltraPure Silica Gels: SilicaFlash® P60 40–63 µm (230–400 mesh). Preparative thin layer chromatography (PTLC) was performed with Merck TLC Silica gel 60 F_{254} glass plates. Reactions were monitored by thin layer chromatography (TLC) using Merck Kieselgel 60 F_{254} aluminium plates. TLC was visualized by UV fluorescence (254 nm) and then stained by phosphomolybdic acid or potassium permanganate. NMR spectra were recorded on AV2 400 MHz, AV2 500 MHz, AV2 600 MHz or AV2 800 MHz Bruker spectrometers. Chemical shifts (δ) were reported in parts per million (ppm) relative to residual solvent peaks rounded to the nearest 0.01 for proton and 0.1 for carbon (ref: $CDCl_3$ [^{1}H: 7.26, ^{13}C: 77.16 ppm]). Coupling constants (J) were reported in Hz to the nearest 0.1 Hz. Peak multiplicity was indicated as follows: singlet (s), doublet (d), triplet (t), quartet (q), doublet-doublet (dd), doublet-triplet (dt), triplet-doublet (td), multiplet (m), and broad singlet (brs). ^{19}F NMR chemical shifts were referenced to external $CFCl_3$ (0.0 ppm). High-resolution mass spectra (HRMS) were measured by the mass spectrometry service of the EPFL by ESI-TOF using a QTOF Ultima from Waters or APPI-FT-ICR using a linear ion trap Fourier transform ion cyclotron resonance mass spectrometer from Thermo Scientific. Melting points are uncorrected and were recorded on a Stuart SMP30 melting point apparatus. Infrared (IR) data were recorded on an Alpha-P Bruker FT-IR Spectrometer or Perkin-Elmer FT-IR spectrometer. Absorbance frequencies are reported in reciprocal centimeters (cm^{-1}). The intensity of the absorbance frequencies is indicated with: weak (w), medium (m), strong (s).

II. Optimization studies

Table S1: Overview of the condition optimization

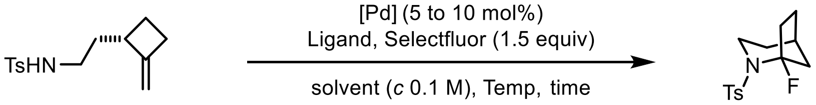

4a 0.05 mmol → **5a**

Entry	[Pd]	Ligand	Solvent	Temp	Time	Yield of **5a** (%)[a]
1	$Pd(OAc)_2$ (10 mol%)	**L3** (15 mol%)	MeCN	40 °C	6 h	decomposed
2	$Pd(OAc)_2$ (10 mol%)	**L3** (15 mol%)	DCE	40 °C	6 h	NR
3	$Pd(OAc)_2$ (10 mol%)	**L1** (15 mol%)	MeCN	40 °C	6 h	28%
4	$Pd(OAc)_2$ (10 mol%)	**L1** (15 mol%)	DCE	40 °C	6 h	trace
5	$Pd(OAc)_2$ (10 mol%)	**L4** (15 mol%)	MeCN	30 °C	6 h	28%
6	$Pd(OAc)_2$ (10 mol%)	**L1** (15 mol%)	MeCN	30 °C	6 h	29%
7	$Pd(OAc)_2$ (10 mol%)	--	MeCN	rt	1 h	33%
8	--	--	MeCN	rt	6 h	decomposed
9[b]	$Pd(OAc)_2$ (10 mol%)	--	MeCN	rt	13 h	no dp
10	$Pd(OPiv)_2$ (10 mol%)	--	MeCN	rt	1 h	31%
11	$Pd(AdCO_2)_2$ (10 mol%)	--	MeCN	rt	1 h	26%
12	$Pd(TFA)_2$ (10 mol%)	--	MeCN	rt	1 h	24%
13	$Pd(dba)_2$ (10 mol%)	--	MeCN	rt	1 h	20%
14	$Pd(MeCN)_4BF_4$ (10 mol%)	--	MeCN	rt	1 h	18%
15	$Pd(PPh_3)_2Cl_2$ (10 mol%)	--	MeCN	rt	1 h	24%
16	$[Pd(\pi\text{-allyl})Cl]_2$ (10 mol%)	--	MeCN	rt	1 h	24%
17	$Pd(acac)_2$ (10 mol%)	--	MeCN	rt	1 h	34%
18	$Pd(hfacac)_2$ (10 mol%)	--	MeCN	rt	1 h	35%
19	$Pd(OAc)_2$ (10 mol%)	--	MeCN	40 °C	10 min	43%
**20**	**$Pd(hfacac)_2$ (10 mol%)**	**--**	**MeCN**	**40 °C**	**10 min**	**63%**

[a] Yields were determined by ^1H NMR of the crude reaction mixture using CH_2Br_2 as an internal standard.
[b] Selectfluor was removed.

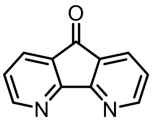

L1

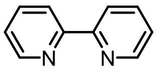

L3

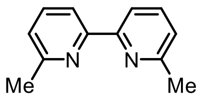

L4

Table S2: Overview of the condition optimization

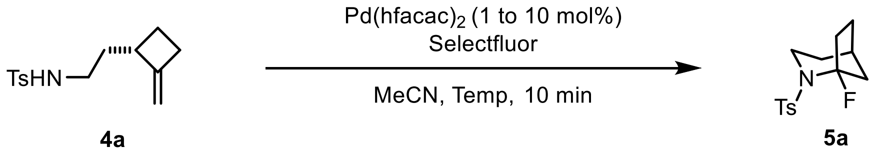

Entry	$Pd(hfacac)_2$ loading	Selectfluor	Temp	Conc	Yield of **5a** (%)[a]
1	10 mol%	1.5 equiv	50 °C	0.1 M	65%
2	10 mol%	1.5 equiv	55 °C	0.1 M	65%
3	10 mol%	1.5 equiv	60°C	0.1 M	66%
4	10 mol%	1.5 equiv	65 °C	0.1 M	63%
5	10 mol%	1.5 equiv	70 °C	0.1 M	61%
6	10 mol%	1.5 equiv	80 °C	0.1 M	48%
7	10 mol%	1.3 equiv	60°C	0.1 M	56%
8	10 mol%	1.7 equiv	60°C	0.1 M	74%
9	10 mol%	2.0 equiv	60°C	0.1 M	76%
10	10 mol%	2.3 equiv	60°C	0.1 M	77%
11	10 mol%	2.0 equiv	60°C	0.067 M	80%
12	10 mol%	2.0 equiv	60°C	0.05 M	82%
13	10 mol%	2.5 equiv	60°C	0.05 M	82%
14	10 mol%	2.5 equiv	60°C	0.033 M	82%
**15**	**5 mol%**	**2.0 equiv**	**60°C**	**0.05 M**	**82%**
16	2.5 mol%	2.0 equiv	60°C	0.05 M	79%
17	1 mol%	2.0 equiv	60°C	0.05 M	78%
**18**	**5 mol%**	**2.0 equiv**	**60°C**	**0.05 M**	**76%**[b]

[a] Yields were determined by ^1H NMR of the crude reaction mixture using CH_2Br_2 as an internal standard.
[b] isolated yield (0.1 mmol scale)

III. Synthesis of Substrates

Scheme S1. General Procedure A

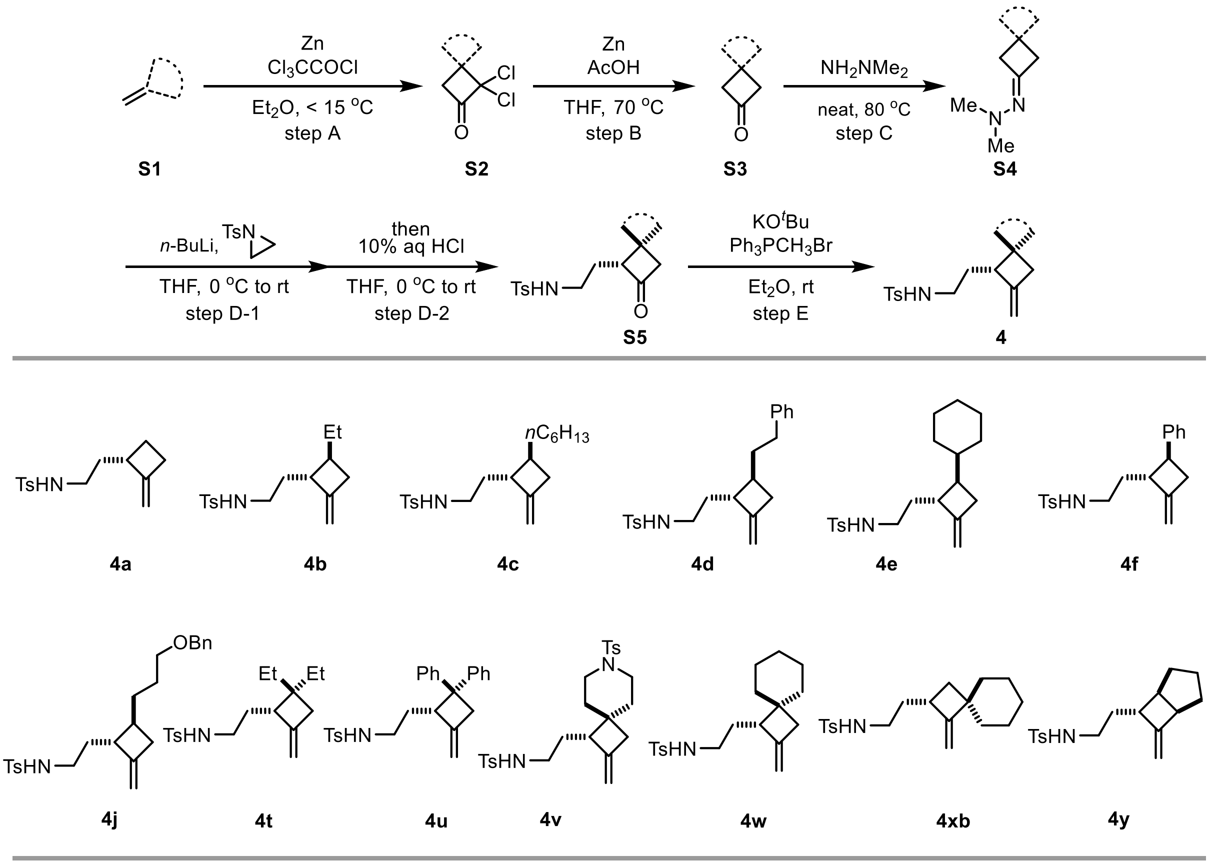

Step A: To a sonicated suspension of **S1** (1.0 equiv) and zinc dust (2.0 equiv) in diethyl ether (c = 0.30 mol/L) was added trichloroacetyl chloride (1.5 equiv) in diethyl ether dropwise over a 1 to 2 h period under N_2 atmosphere. The reaction mixture was sonicated below 15 °C for a certain time (~6 h). Then the reaction mixture was filtered through a pad of Celite and rinsed with diethyl ether. The filtrate was washed sequentially with water, saturated sodium bicarbonate and brine. The organic layer was dried over anhydrous sodium sulfate, filtered and concentrated under vacuum to afford the crude **S2**.

Step B: To a stirred suspension of the above crude **S2** (1.0 equiv) and zinc dust (10.0 equiv) in anhydrous tetrahydrofuran (c = 0.10 mol/L) was added glacial acetic acid (8.0 equiv). The reaction mixture was heated to 70 °C for a certain time (~5 h) until the starting material was completely consumed. After cooling to room temperature, the mixture was filtered through a pad of Celite and rinsed with diethyl ether. The filtrate was washed twice with saturated sodium bicarbonate and twice with brine. The organic layer was dried over anhydrous sodium sulfate,

filtered and concentrated under vacuum. The residue was purified by flash column chromatography on silica gel to give compound **S3**.

Step C: A round-bottom flask charged with the cyclobutanone **S3** (1.0 equiv) and *N*,*N*-dimethylhydrazine (5.0 equiv) was heated to 80 °C overnight with a refluxing condenser. After cooling to room temperature, the reaction mixture was concentrated under vacuum at 30 °C to remove excess *N*,*N*-dimethylhydrazine. The resulting mixture was filtered through a short pad of anhydrous sodium sulfate and the filtrate was purified by distillation under reduced pressure or flash column chromatography on silica gel to afford the title compound **S4**.

Step D-1 and step D-2: The corresponding hydrazone **S4** (1.0 equiv) was dissolved in anhydrous tetrahydrofuran (c = 0.25 mol/L) and the resulting mixture was cooled to 0 °C, then *n*-butyllithium (1.1 equiv) was added dropwise. The resulting mixture was stirred for 1 h at 0 °C and a solution of *N*-tosylaziridine (1.1 equiv) in anhydrous THF (10 mL) was added slowly. After stirring at 0 °C for 1 h, the reaction mixture was allowed to stir overnight at room temperature. A 10% aqueous solution of HCl (the same volume as the reaction solution) was added at 0 °C and the mixture was stirred overnight at room temperature. The resulting mixture was extracted four times with ethyl acetate. The combined organic layers were washed with saturated sodium bicarbonate and brine, dried over anhydrous sodium sulfate, filtered and concentrated under vacuum. The residue was purified by flash column chromatography on silica gel to give title compound **S5**.

Step E: Potassium *tert*-butoxide (3.0 equiv) was added to Ph_3PCH_3Br (2.5 equiv) in anhydrous diethyl ether (c = 0.1 mol/L) at room temperature. The reaction mixture was stirred at room temperature for 2 h. Then the resulting solution was transferred to a round-bottom flask charged with compound **S5** (1.0 equiv) at room temperature. The reaction mixture was stirred overnight at room temperature. TLC showed that the starting material was consumed completely, then the reaction mixture was quenched by the addition of saturated ammonium chloride. The resulting mixture was extracted three times with ethyl acetate. The combined organic layers were washed twice with brine, dried over anhydrous sodium sulfate, filtered and concentrated under vacuum. The residue was purified by flash column chromatography on silica gel to afford title compound **4**.

Scheme S2. General Procedure B

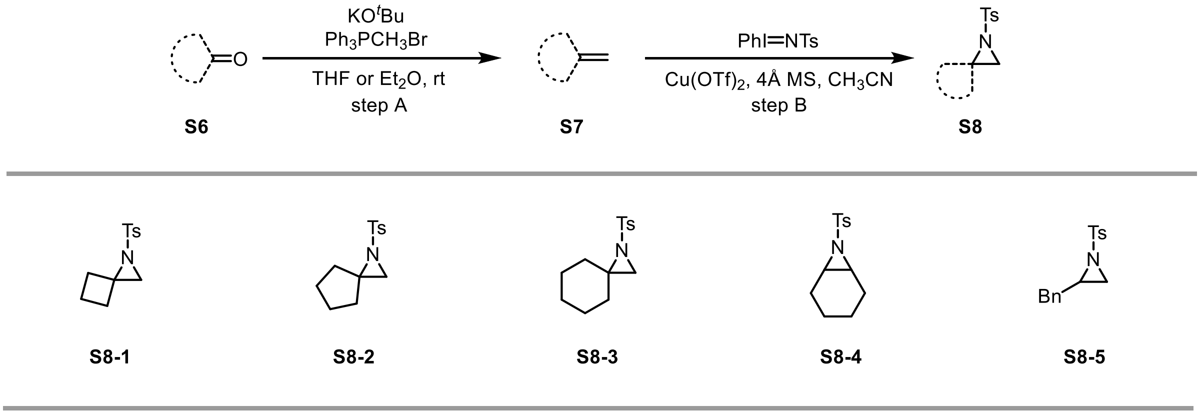

Step A: Potassium *tert*-butoxide (2.0 equiv) was added to Ph_3PCH_3Br (2.0 equiv) in anhydrous tetrahydrofuran (c = 0.3 mol/L) at room temperature. The reaction mixture was stirred at room temperature for 2 h. Then the resulting solution was transferred to a round-bottom flask charged with compound **S6** (1.0 equiv) at room temperature. The reaction mixture was stirred overnight at room temperature. TLC showed that the starting material was consumed completely, then the reaction mixture was quenched by the addition of saturated ammonium chloride. The resulting mixture was extracted three times with diethyl ether. The combined organic layers were washed twice with brine, dried over anhydrous sodium sulfate, filtered and concentrated under vacuum. The residue was purified by flash column chromatography on silica gel to afford title compound **S7**.

Step B: Following the known literature procedure,[1] to a round-bottom flask charged with 4Å molecular sieves (same weight as **S7**), copper(II) triflate (0.05 equiv) and **S7** (3.5 equiv) in anhydrous acetonitrile was added PhI=NTs[1] (1.0 equiv) in portions over a 20 mins period at 0 ^{o}C. The reaction mixture was stirred at 0 ^{o}C for a further 1 h and then allowed to stir at room temperature for 12 h. The mixture was diluted with diethyl ether, filtered through a pad of Celite and rinsed with diethyl ether. The filtrate was concentrated under vacuum. The residue was purified by flash column chromatography on silica gel (eluent: diethyl ether-hexane-triethylamine) to afford the desired compound **S8**. [known compounds **S8-1**[1], **S8-2**[1], **S8-3**[1] and **S8-4**[1] were synthesized from Step B; **S8-5** was commercially available]

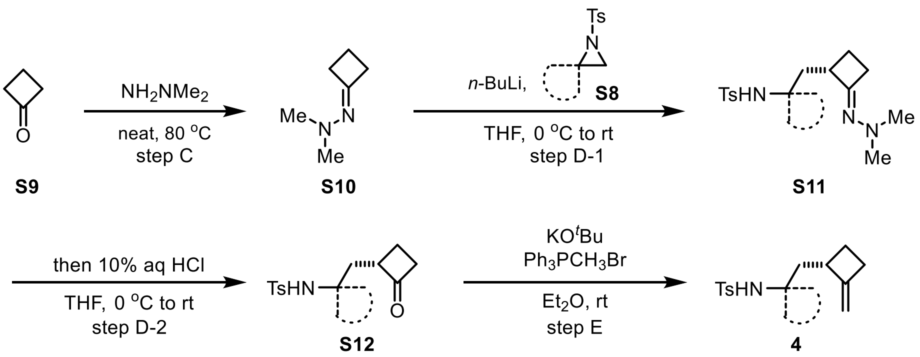

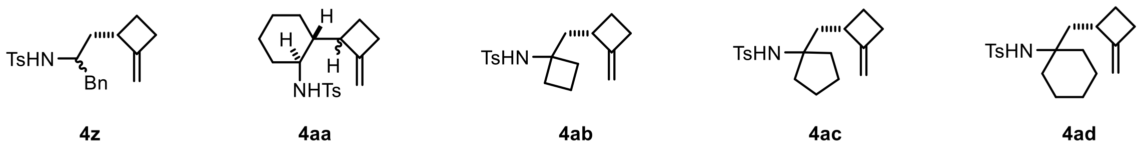

Step C: A round-bottom flask charged with the cyclobutanone **S9** (7.01 g, 100 mmol, 1.0 equiv) and *N*,*N*-dimethylhydrazine (24.0 g, 400 mmol, 4.0 equiv) was heated to 80 °C with a refluxing condenser for 24 h. After cooling to room temperature, the reaction mixture was concentrated under vacuum at 30 °C to remove excess *N*,*N*-dimethylhydrazine. The resulting mixture was filtered through a short pad of anhydrous sodium sulfate and the filtrate was purified by distillation under reduced pressure (20 mbar, 80 °C) to afford the title compound **S10** as a colorless oil (7.29 g, 65%).

Step D-1 and Step D-2: The corresponding hydrazone **S10** (1.0 equiv) was dissolved in anhydrous tetrahydrofuran (c = 0.25 mol/L) and the resulting mixture was cooled to 0 °C, then *n*-butyllithium (1.1 equiv) was added dropwise. The resulting mixture was stirred for 1 h at 0 °C and a solution of substituted *N*-tosylaziridine (1.1 equiv) in anhydrous THF (10 mL) was added slowly. After stirring at 0 °C for 1 h, the reaction mixture was allowed to stir overnight at room temperature to yield compound **S11**. A 10% aqueous solution of HCl (the same volume as the reaction solution) was added at 0 °C and the mixture was stirred overnight at room temperature. The resulting mixture was extracted four times with ethyl acetate. The combined organic layers were washed with saturated sodium bicarbonate and brine, dried over anhydrous sodium sulfate, filtered and concentrated under vacuum. The residue was purified by flash column chromatography on silica gel to give title compound **S12**.

Step E: Potassium *tert*-butoxide (3.0 equiv) was added to Ph_3PCH_3Br (2.5 equiv) in anhydrous diethyl ether (c = 0.1 mol/L) at room temperature. The reaction mixture was stirred at room temperature for 2 h. Then the resulting solution was transferred to a round-bottom flask charged

with compound **S12** (1.0 equiv) at room temperature. The reaction mixture was stirred overnight at room temperature. TLC showed that the starting material was consumed completely, then the reaction mixture was quenched by the addition of saturated ammonium chloride. The resulting mixture was extracted three times with ethyl acetate. The combined organic layers were washed twice with brine, dried over anhydrous sodium sulfate, filtered and concentrated under vacuum. The residue was purified by flash column chromatography on silica gel to afford title compound **4**.

Scheme S3. General Procedure C

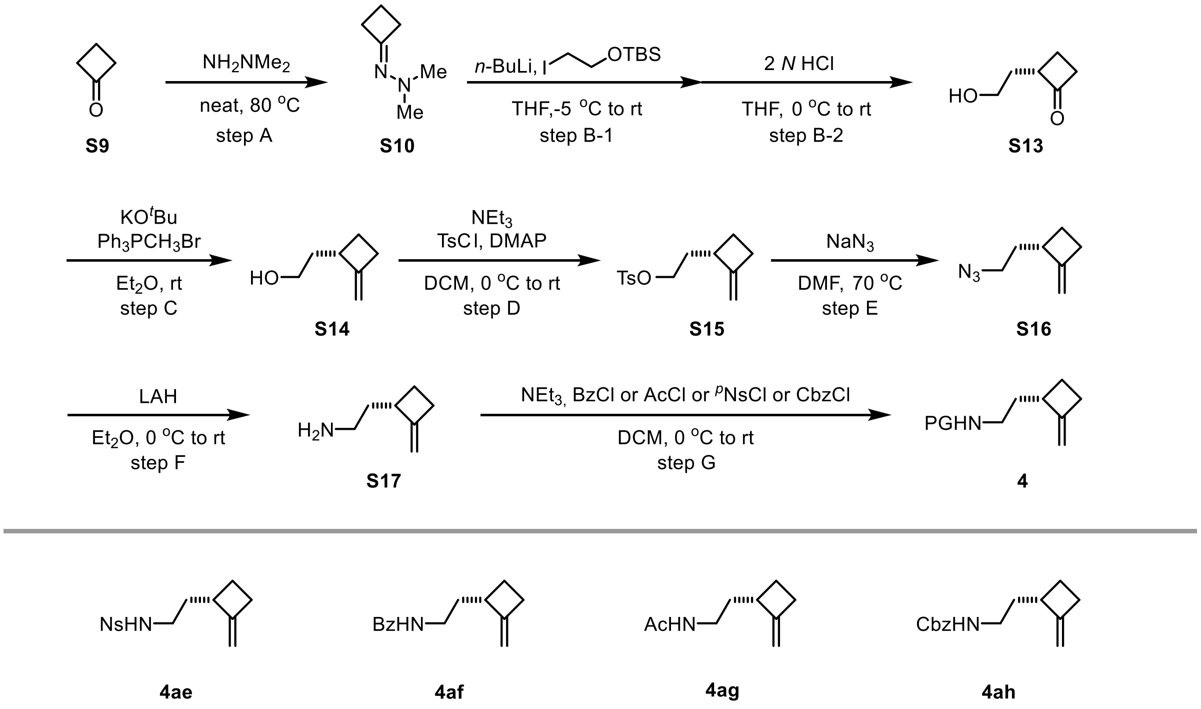

Step A: A round-bottom flask charged with the cyclobutanone **S9** (7.01 g, 100 mmol, 1.0 equiv) and *N*,*N*-dimethylhydrazine (24.0 g, 400 mmol, 4.0 equiv) was heated to 80 ^oC with a refluxing condenser for 24 h. After cooling to room temperature, the reaction mixture was concentrated under vacuum at 30 ^oC to remove excess *N*,*N*-dimethylhydrazine. The resulting mixture was filtered through a short pad of anhydrous sodium sulfate and the filtrate was purified by distillation under reduced pressure (20 mbar, 80 ^oC) to afford the title compound **S10** as a colorless oil (7.29 g, 65%).

Step B-1 and Step B-2: The corresponding hydrazone **S10** (6.73 g, 60.0 mmol, 1.0 equiv) was dissolved in anhydrous tetrahydrofuran (150 mL, c = 0.40 mol/L) and the resulting mixture

was cooled to -5 °C, then *n*-butyllithium (2.5 M in hexane, 25.2 mL, 63.0 mmol, 1.05 equiv) was added dropwise. The resulting mixture was stirred for 1 h at -5 °C and a solution of *tert*-butyl-(2-iodoethoxy)-dimethylsilane[2] in anhydrous tetrahydrofuran (18.9 g, 66.0 mmol, 1.1 equiv) was added slowly. After stirring at -5 °C for 1 h, the reaction mixture was allowed to stir at room temperature for 0.5 h, and an aqueous solution of 2*N* HCl (150 mL) was added at 0 °C. The mixture was stirred for 3 h at room temperature. The resulting mixture was extracted with diethyl ether (4×60 mL). The combined organic layers were washed with brine (2×80 mL), dried over anhydrous sodium sulfate, filtered and concentrated under vacuum at 10 ^{o}C. The residue was purified by flash column chromatography on silica gel (0% to 100% diethyl ether-pentane) to afford compound **S13** (3.42 g, 30 mmol).

Step C: Potassium *tert*-butoxide (10.1 g, 90 mmol, 3.0 equiv) was added to Ph_3PCH_3Br (32.2 g, 90 mmol, 3.0 equiv) in anhydrous diethyl ether (100 mL, c = 0.30 mol/L) at room temperature. The reaction mixture was stirred at room temperature for 2 h. Then the resulting solution was transferred to a round-bottom flask charged with the above crude **S13** (3.42 g, 30 mmol, 1.0 equiv) at room temperature. The reaction mixture was stirred overnight at room temperature. TLC showed that the starting material was consumed completely, then the reaction mixture was quenched by the addition of saturated ammonium chloride. The resulting mixture was extracted with diethyl ether (3×50 mL). The combined organic layers were washed with brine (2×60 mL), dried over anhydrous sodium sulfate, filtered and concentrated under vacuum at 10 ^{o}C. The residue was purified by flash column chromatography on silica gel (0% to 10% to 30% diethyl ether-pentane) to give compound **S14** (960 mg).

Step D: To a stirred solution of **S14** (960 mg, 8.56 mmol, 1.0 equiv) in anhydrous dichloromethane (20 mL, c = 0.43 mol/L) was sequentially added 4-dimethylaminopyridine (105 mg, 0.86 mmol, 0.1 equiv) and triethylamine (1.78 mL, 12.84 mmol, 1.5 equiv) at 0 ^{o}C, followed by tosyl chloride (1.79 g, 9.42 mmol, 1.1 equiv). The reaction mixture was allowed to stir at room temperature for 14 h. TLC showed that the starting material was consumed completely, then the reaction mixture was quenched by the addition of brine. The resulting mixture was extracted with ethyl acetate (3×30 mL). The combined organic layers were washed with brine (2×30 mL), dried over anhydrous sodium sulfate, filtered and concentrated under vacuum. The residue was purified by flash column chromatography on silica gel (0% to 1% ethyl acetate-hexane) to give compound **S15** as colorless oil (1.02 g, 6.4% over 3 steps).

Step E: To a stirred solution of **S15** (600 mg, 2.25 mmol, 1.0 equiv) in anhydrous *N*,*N*-dimethylformamide (8 mL, *c* = 0.28 mol/L) was added sodium azide (293 mg, 4.51 mmol, 2.0 equiv) at room temperature. The reaction mixture was allowed to stir at 70 °C for 5 h. After cooling to room temperature, the reaction mixture was quenched by the addition of water. The resulting mixture was extracted with diethyl ether (3×15 mL). The combined organic layers were washed with water (3×15 mL) and brine (3×15 mL), dried over anhydrous sodium sulfate, filtered and concentrated under vacuum to afford the crude **S16** as pale yellow oil.

Step F: To a stirred solution of **S16** (2.25 mmol, 1.0 equiv) in anhydrous diethyl ether (15 mL, *c* = 0.15 mol/L) was added lithium aluminum hydride (1.0 M in tetrahydrofuran, 4.5 mL, 4.5 mmol, 2.0 equiv) dropwise at 0 °C. The reaction mixture was allowed to stir at room temperature for 13 h. TLC showed that the starting material was consumed completely, then the reaction mixture was cooled to 0 °C and carefully quenched by the sequential addition of water (0.17 mL), 15% aqueous sodium hydroxide (0.17 mL) and water (3×0.17 mL). The resulting mixture was allowed to warm to room temperature and stirred for 30 min, then anhydrous sodium sulfate was added and stirred for additional 30 min. The mixture was filtered through a short pad of Celite and rinsed with diethyl ether. The filtrate was concentrated under vacuum at 10 °C to yield crude amine **S17**.

Step G: To a stirred solution of one quarter of the above crude **S17** (0.563 mmol, 1.0 equiv) in anhydrous dichloromethane (6 mL, *c* = 0.094 mol/L) was added triethylamine (118 µL, 0.85 mmol, 1.5 equiv) at 0 °C, followed by the addition of acyl chloride or sulfonyl chloride (0.62 mmol, 1.1 equiv). The reaction mixture was allowed to stir at room temperature for 16 h. The reaction mixture was quenched by the addition of saturated sodium bicarbonate. The resulting mixture was extracted with ethyl acetate (3×10 mL). The combined organic layers were washed with brine (2×10 mL), dried over anhydrous sodium sulfate, filtered and concentrated under vacuum. The residue was purified by flash column chromatography on silica gel to afford compound **4ae** as white solid (44.6 mg, 27% over 3 steps), compound **4af** as colorless oil (46 mg, 38% over 3 steps), compound **4ag** as pale yellow oil (41.5 mg, 48% over 3 steps), and compound **4ah** as colorless oil (40.4 mg, 29% over 3 steps).

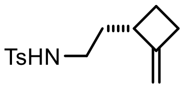

4a

4-Methyl-*N*-(2-(2-methylenecyclobutyl)ethyl)benzenesulfonamide (4a): Following the general procedure A, compound **4a** was isolated after purification by flash column chromatography on silica gel as a colorless oil. **^{1}H NMR** (400 MHz, $CDCl_3$) δ 7.75 (d, J = 8.3 Hz, 2H), 7.31 (d, J = 8.0 Hz, 2H), 4.67 (q, J = 2.3 Hz, 1H), 4.63 (q, J = 2.5 Hz, 1H), 4.43 (t, J = 6.2 Hz, 1H), 3.03 – 2.92 (m, 2H), 2.92 – 2.83 (m, 1H), 2.65 – 2.47 (m, 2H), 2.43 (s, 3H), 2.08 – 1.97 (m, 1H), 1.82 – 1.70 (m, 1H), 1.63 – 1.46 (m, 2H) ppm; **^{13}C NMR** (100 MHz, $CDCl_3$) δ 153.6, 143.5, 137.1, 129.8 (2C), 127.2 (2C), 104.2, 41.8, 41.3, 34.1, 29.4, 23.5, 21.7 ppm; **IR** (ν_{max}, cm^{-1}) 3276 (w), 2922 (w), 1672 (w), 1599 (w), 1419 (m), 1321 (m), 1155 (s), 1092 (m), 872 (m), 816 (m), 661 (s); **HRMS** (ESI/QTOF) m/z: $[M + Na]^+$ Calcd for $C_{14}H_{19}NNaO_2S^+$ 288.1029; Found 288.1039.

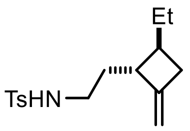

4b

***N*-(2-(2-Ethyl-4-methylenecyclobutyl)ethyl)-4-methylbenzenesulfonamide (4b)**: Following the general procedure A, compound **4b** was isolated after purification by flash column chromatography on silica gel as a colorless oil. **^{1}H NMR** (400 MHz, $CDCl_3$) δ 7.75 (d, J = 8.4 Hz, 2H), 7.30 (d, J = 8.1 Hz, 2H), 4.72 (t, J = 6.0 Hz, 1H), 4.68 (q, J = 2.3 Hz, 1H), 4.64 (q, J = 2.5 Hz, 1H), 3.03 – 2.89 (m, 2H), 2.69 – 2.57 (m, 1H), 2.42 (s, 3H), 2.43 – 2.37 (m, 1H), 2.12 (ddt, J = 15.2, 7.3, 2.6 Hz, 1H), 1.77 – 1.65 (m, 2H), 1.64 – 1.54 (m, 1H), 1.50 – 1.39 (m, 1H), 1.38 – 1.26 (m, 1H), 0.78 (t, J = 7.4 Hz, 3H) ppm; **^{13}C NMR** (100 MHz, $CDCl_3$) δ 150.9, 143.5, 137.0, 129.8 (2C), 127.2 (2C), 104.5, 47.1, 41.5, 39.2, 35.0, 33.6, 29.1, 21.6, 11.8 ppm; **IR** (ν_{max}, cm^{-1}) 3286 (w), 2927 (w), 2861 (w), 1676 (w), 1596 (w), 1431 (w), 1325 (m), 1156 (s), 1094 (m), 874 (m), 814 (m), 662 (s); **HRMS** (ESI/QTOF) m/z: $[M + Na]^+$ Calcd for $C_{16}H_{23}NNaO_2S^+$ 316.1342; Found 316.1348.

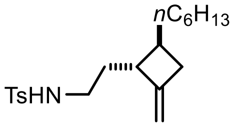

4c

***N*-(2-(2-Hexyl-4-methylenecyclobutyl)ethyl)-4-methylbenzenesulfonamide (4c)**: Following the general procedure A, compound **4c** was isolated after purification by flash column chromatography on silica gel as a colorless oil. **^{1}H NMR** (400 MHz, $CDCl_3$) δ 7.75 (d, *J* = 8.3 Hz, 2H), 7.30 (d, *J* = 8.0 Hz, 2H), 4.78 (t, *J* = 6.1 Hz, 1H), 4.67 (q, *J* = 2.3 Hz, 1H), 4.63 (q, *J* = 2.5 Hz, 1H), 3.03 – 2.88 (m, 2H), 2.68 – 2.57 (m, 1H), 2.42 (s, 3H), 2.43 – 2.35 (m, 1H), 2.11 (ddt, *J* = 15.2, 7.4, 2.6 Hz, 1H), 1.81 – 1.66 (m, 2H), 1.64 – 1.53 (m, 1H), 1.44 – 1.34 (m, 1H), 1.33 – 1.08 (m, 9H), 0.87 (t, *J* = 7.0 Hz, 3H) ppm; **^{13}C NMR** (100 MHz, $CDCl_3$) *δ* 151.0, 143.4, 137.0, 129.8 (2C), 127.2 (2C), 104.4, 47.5, 41.5, 37.6, 36.4, 35.4, 33.6, 31.9, 29.4, 27.5, 22.7, 21.6, 14.2 ppm; **IR** (ν_{max}, cm^{-1}) 3276 (w), 2922 (m), 2850 (m), 1669 (w), 1599 (w), 1422 (m), 1329 (m), 1160 (s), 1094 (m), 873 (m), 813 (m), 660 (s); **HRMS** (ESI/QTOF) m/z: $[M + H]^+$ Calcd for $C_{20}H_{32}NO_2S^+$ 350.2148; Found 350.2147.

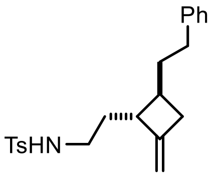

4d

4-Methyl-*N*-(2-(2-methylene-4-phenethylcyclobutyl)ethyl)benzenesulfonamide (4d): Following the general procedure A, compound **4d** was isolated after purification by flash column chromatography on silica gel as a colorless oil. **^{1}H NMR** (400 MHz, $CDCl_3$) *δ* 7.78 – 7.72 (m, 2H), 7.31 – 7.24 (m, 4H), 7.22 – 7.15 (m, 1H), 7.15 – 7.11 (m, 2H), 4.70 (q, *J* = 2.3 Hz, 1H), 4.69 – 4.65 (m, 1H), 4.66 (q, *J* = 2.6 Hz, 1H), 3.03 – 2.89 (m, 2H), 2.72 – 2.62 (m, 1H), 2.58 – 2.43 (m, 3H), 2.41 (s, 3H), 2.18 (ddt, *J* = 15.3, 7.0, 2.6 Hz, 1H), 1.87 – 1.53 (m, 5H) ppm; **^{13}C NMR** (100 MHz, $CDCl_3$) *δ* 150.5, 143.5, 142.2, 137.0, 129.8 (2C), 128.43 (2C), 128.42 (2C), 127.2 (2C), 125.9, 104.7, 47.4, 41.4, 38.0, 37.1, 35.4, 33.9, 33.5, 21.6 ppm; **IR** (ν_{max}, cm^{-1}) 3267 (w), 2924 (w), 1672 (w), 1599 (w), 1419 (m), 1321 (m), 1153 (s), 1092 (m), 874 (m), 816 (m), 698 (s), 665 (s); **HRMS** (ESI/QTOF) m/z: $[M + Na]^+$ Calcd for $C_{22}H_{27}NNaO_2S^+$ 392.1655; Found 392.1666.

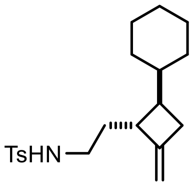

4e

***N*-(2-(2-Cyclohexyl-4-methylenecyclobutyl)ethyl)-4-methylbenzenesulfonamide (4e)**: Following the general procedure A, compound **4e** was isolated after purification by flash column chromatography on silica gel as a colorless oil. **^{1}H NMR** (400 MHz, $CDCl_3$) δ 7.75 (d, J = 8.3 Hz, 2H), 7.31 (d, J = 8.0 Hz, 2H), 4.68 (q, J = 2.4 Hz, 1H), 4.64 (q, J = 2.5 Hz, 1H), 4.59 (t, J = 6.2 Hz, 1H), 3.03 – 2.94 (m, 2H), 2.61 – 2.48 (m, 2H), 2.42 (s, 3H), 2.19 (ddt, J = 15.3, 7.4, 2.6 Hz, 1H), 1.73 – 1.54 (m, 7H), 1.54 – 1.46 (m, 1H), 1.23 – 1.03 (m, 4H), 0.80-0.66 (m, 2H) ppm; **^{13}C NMR** (100 MHz, $CDCl_3$) δ 151.1, 143.5, 137.1, 129.8 (2C), 127.3 (2C), 104.6, 45.5, 43.5, 42.9, 41.6, 34.0, 33.5, 30.8, 30.4, 26.6, 26.2, 26.2, 21.7 ppm; **IR** (ν_{max}, cm^{-1}) 3290 (w), 2924 (m), 2848 (w), 1672 (w), 1599 (w), 1452 (w), 1325 (m), 1153 (s), 1093 (m), 876 (m), 814 (m), 660 (s); **HRMS** (ESI/QTOF) m/z: $[M + Na]^+$ Calcd for $C_{20}H_{29}NNaO_2S^+$ 370.1811; Found 370.1817.

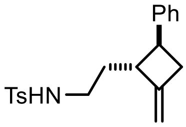

4f

4-Methyl-*N*-(2-(2-methylene-4-phenylcyclobutyl)ethyl)benzenesulfonamide (4f): Following the general procedure A, compound **4f** was isolated after purification by flash column chromatography on silica gel as a white solid. m.p. = 49 ~ 51 ^{o}C; **^{1}H NMR** (400 MHz, $CDCl_3$) δ 7.70 – 7.66 (m, 2H), 7.40 – 7.25 (m, 7H), 4.89 (q, J = 2.4 Hz, 1H), 4.85 (q, J = 2.4 Hz, 1H), 4.57 (t, J = 6.3 Hz, 1H), 3.11 – 2.93 (m, 5H), 2.83 – 2.74 (m, 1H), 2.48 (s, 3H), 2.02 – 1.92 (m, 1H), 1.83 – 1.72 (m, 1H) ppm; **^{13}C NMR** (100 MHz, $CDCl_3$) δ 149.5, 144.2, 143.4, 136.8, 129.7 (2C), 128.7 (2C), 127.1 (2C), 126.8 (2C), 126.6, 104.5, 50.3, 42.8, 41.2, 38.1, 33.5, 21.6 ppm; **IR** (ν_{max}, cm^{-1}) 3275 (w), 2924 (w), 1676 (w), 1603 (w), 1495 (w), 1416 (w), 1325 (m), 1156 (s), 1094 (m), 875 (m), 813 (m), 750 (m), 703 (s), 666 (s); **HRMS** (ESI/QTOF) m/z: $[M + Na]^+$ Calcd for $C_{20}H_{23}NNaO_2S^+$ 364.1342; Found 364.1345.

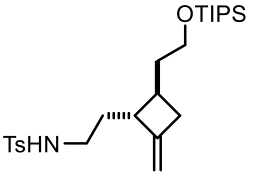

4g

4-Methyl-*N*-(2-(2-methylene-4-(2-((triisopropylsilyl)oxy)ethyl)cyclobutyl)ethyl)benzenesulfonamide (4g): Compound **4g** was obtained as a colorless oil using the same experimental procedure as compound **4h**. **^{1}H NMR** (600 MHz, $CDCl_3$) δ 7.74 (d, J = 8.3 Hz, 2H), 7.30 (d, J = 8.0 Hz, 2H), 4.69 (q, J = 2.4 Hz, 1H), 4.64 (q, J = 2.5 Hz, 1H), 4.59 (t, J = 6.3 Hz, 1H), 3.65-3.56 (m, 2H), 3.03 – 2.92 (m, 2H), 2.71 – 2.63 (m, 1H), 2.52 – 2.46 (m, 1H), 2.42 (s, 3H), 2.21 (ddt, J = 15.3, 7.4, 2.6 Hz, 1H), 1.99-1.92 (m, 1H), 1.77 – 1.65 (m, 2H), 1.64 – 1.55 (m, 2H), 1.10 – 0.98 (m, 21H) ppm; **^{13}C NMR** (100 MHz, $CDCl_3$) δ 151.0, 143.5, 137.1, 129.8 (2C), 127.2 (2C), 104.5, 61.8, 47.4, 41.4, 39.4, 35.6, 34.5, 33.6, 21.6, 18.2 (6C), 12.1 (3C) ppm; **IR** (ν_{max}, cm^{-1}) 3271 (w), 2941 (m), 2864 (m), 1672 (w), 1599 (w), 1462 (m), 1325 (m), 1159 (s), 1095 (s), 879 (s), 814 (m), 731 (m), 660 (s); **HRMS** (ESI/QTOF) m/z: $[M + H]^+$ Calcd for $C_{25}H_{44}NO_3SSi^+$ 466.2806; Found 466.2812.

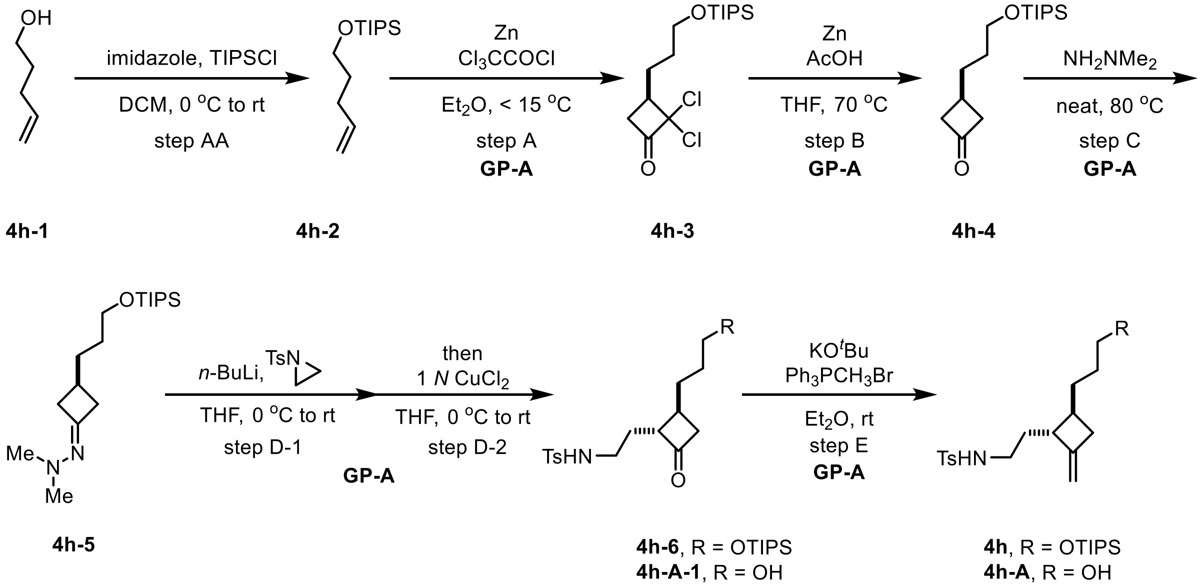

Step AA: Following the known literature procedure,[3] to a stirred solution of compound **4h-1** (2.0 g, 23.2 mmol, 1.0 equiv) and imidazole (2.37 g, 34.8 mmol, 1.5 equiv) in anhydrous dichloromethane (57 mL, c = 0.3 mol/L) was added triisopropylsilyl chloride (4.92 g, 25.5 mmol, 1.1 equiv) at 0 °C. The reaction mixture was allowed to stir at room temperature

overnight. The resulting mixture was quenched with saturated sodium bicarbonate and extracted with diethyl ether (3×50 mL). The combined organic layers were washed with brine (2×60 mL), dried over anhydrous sodium sulfate, filtered and concentrated under vacuum. The residue was purified by flash column chromatography on silica gel (100% hexane) to afford the desired compound **4h-2** as a colorless oil (5.35 g, 95%).

Following the steps A (24 mmol scale) and B of general procedure A, compound **4h-4** was isolated after purification by flash column chromatography on silica gel as a colorless oil (5.6 g, 82% over 2 steps); Following the step C of general procedure A, compound **4h-5** was isolated after purification by flash column chromatography on silica gel as a colorless oil; Following the step D-1 (6.12 mmol scale) and step D-2 [10% aq HCl was replaced by 1M aqueous solution of $CuCl_2$ (1.2 equiv)[4]] of general procedure A, compounds **4h-6** and **4h-A-1** were isolated after purification by flash column chromatography on silica gel (0% to 10% to 17% to 30% to 50% ethyl acetate-hexane) as colorless oil (**4h-6**: 0.6 g, 20%; **4h-A-1**: 1.4 g, 70%). Following the step E (**4h-6**: 1.25 mmol scale; **4h-A-1**: 4.3 mmol scale) of general procedure A, compound **4h** was isolated after purification by flash column chromatography on silica gel (0% to 10% ethyl acetate-hexane) as colorless oil (420 mg, 70%) and compound **4h-A** was isolated after purification by flash column chromatography on silica gel (0% to 35% ethyl acetate-hexane) as colorless oil (880 mg, 63%).

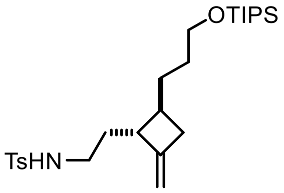

4h

4-Methyl-*N*-(2-(2-methylene-4-(3-((triisopropylsilyl)oxy)propyl)cyclobutyl)ethyl)benzenesulfonamide (4h): **^{1}H NMR** (400 MHz, $CDCl_3$) δ 7.75 (d, J = 8.3 Hz, 2H), 7.31 (d, J = 8.0 Hz, 2H), 4.69 (q, J = 2.3 Hz, 1H), 4.65 (q, J = 2.5 Hz, 1H), 4.47 (t, J = 6.2 Hz, 1H), 3.68 – 3.57 (m, 2H), 3.05 – 2.90 (m, 2H), 2.65 (ddq, J = 15.5, 8.5, 2.4 Hz, 1H), 2.43 (s, 3H), 2.44 – 2.37 (m, 1H), 2.15 (ddt, J = 15.3, 7.4, 2.6 Hz, 1H), 1.85 – 1.68 (m, 2H), 1.66 – 1.55 (m, 1H), 1.53 – 1.34 (m, 4H), 1.13 – 0.98 (m, 21H) ppm; **^{13}C NMR** (100 MHz, $CDCl_3$) δ 150.8, 143.5, 137.1, 129.8 (2C), 127.2 (2C), 104.6, 63.4, 47.6, 41.5, 37.4, 35.5, 33.6, 32.5, 31.1, 21.7, 18.2 (6C), 12.1 (3C) ppm; **IR** (ν_{max}, cm^{-1}) 2933 (m), 2866 (m), 1672 (w), 1597 (w), 1464 (m), 1329 (m), 1159 (s), 1095 (s), 991 (m), 877

(s), 816 (m), 667 (s); **HRMS** (ESI/QTOF) m/z: $[M + Na]^+$ Calcd for $C_{26}H_{45}NNaO_3SSi^+$ 502.2782; Found 502.2777.

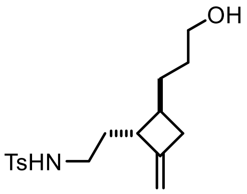

4h-A

***N*-(2-(2-(3-Hydroxypropyl)-4-methylenecyclobutyl)ethyl)-4-methylbenzenesulfonamide (4h-A)**: **^{1}H NMR** (400 MHz, $CDCl_3$) δ ^{1}H NMR (400 MHz, $CDCl_3$) δ 7.78 – 7.71 (m, 2H), 7.30 (d, *J* = 8.0 Hz, 2H), 4.96 (t, *J* = 6.1 Hz, 1H), 4.68 (q, *J* = 2.3 Hz, 1H), 4.64 (q, *J* = 2.5 Hz, 1H), 3.60 (t, *J* = 6.0 Hz, 2H), 3.03 – 2.89 (m, 2H), 2.71 – 2.60 (m, 1H), 2.52 – 2.42 (m, 1H), 2.42 (s, 3H), 2.15 (ddt, *J* = 15.2, 7.2, 2.6 Hz, 1H), 1.92 – 1.79 (m, 2H), 1.74 (dq, *J* = 13.9, 7.0 Hz, 1H), 1.65 – 1.35 (m, 5H) ppm; **^{13}C NMR** (100 MHz, $CDCl_3$) δ 150.7, 143.5, 136.9, 129.8 (2C), 127.2 (2C), 104.6, 62.8, 47.4, 41.4, 37.3, 35.4, 33.5, 32.3, 30.5, 21.6 ppm; **IR** (ν_{max}, cm^{-1}) 3487 (w), 3292 (w), 2918 (w), 2864 (w), 1668 (w), 1599 (w), 1427 (w), 1321 (m), 1153 (s), 1092 (m), 874 (m), 814 (m), 725 (m), 663 (s); **HRMS** (ESI/QTOF) m/z: $[M + Na]^+$ Calcd for $C_{17}H_{25}NNaO_3S^+$ 346.1447; Found 346.1451.

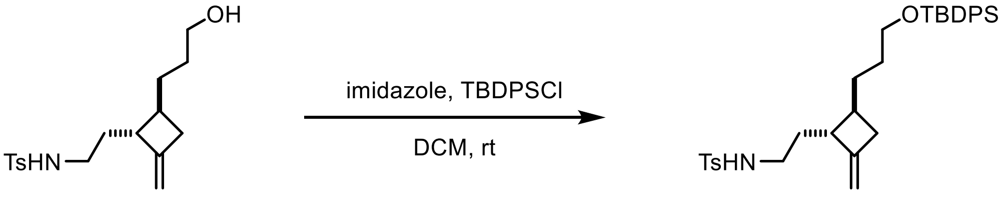

***N*-(2-(2-(3-((*tert*-Butyldiphenylsilyl)oxy)propyl)-4-methylenecyclobutyl)ethyl)-4-methylbenzenesulfonamide (4i)**: To a stirred solution of compound **4h-A** (97 mg, 0.30 mmol, 1.0 equiv) and imidazole (40.8 mg, 0.60 mmol, 2.0 equiv) in anhydrous dichloromethane (6 mL, *c* = 0.05 mol/L) was added *tert*-butylchlorodiphenylsilane (91.8 mg, 0.334 mmol, 1.1 equiv) at room temperature. The reaction mixture was stirred at room temperature until the starting material was completely consumed. The resulting mixture was quenched with saturated sodium bicarbonate and extracted with ethyl acetate (3×10 mL). The combined organic layers were washed with brine (2×10 mL), dried over anhydrous sodium sulfate, filtered and

concentrated under vacuum. The residue was purified by flash column chromatography on silica gel to afford the desired compound **4i** as a colorless oil. **^{1}H NMR** (400 MHz, $CDCl_3$) δ 7.74 (d, J = 8.3 Hz, 2H), 7.69 – 7.63 (m, 4H), 7.46 – 7.35 (m, 6H), 7.28 (d, J = 7.9 Hz, 2H), 4.69 (q, J = 2.3 Hz, 1H), 4.65 (q, J = 2.5 Hz, 1H), 4.47 (t, J = 6.1 Hz, 1H), 3.61 (t, J = 5.8 Hz, 2H), 3.03 – 2.89 (m, 2H), 2.62 (ddq, J = 15.4, 8.4, 2.4 Hz, 1H), 2.40 (s, 3H), 2.45 – 2.35 (m, 1H), 2.12 (ddt, J = 15.3, 7.3, 2.6 Hz, 1H), 1.81 – 1.66 (m, 2H), 1.63 – 1.53 (m, 1H), 1.52 – 1.33 (m, 4H), 1.05 (s, 9H) ppm; **^{13}C NMR** (100 MHz, $CDCl_3$) δ 150.7, 143.5, 137.0, 135.7 (4C), 134.1 (2C), 129.8 (2C), 129.7 (2C), 127.7 (4C), 127.2 (2C), 104.7, 63.9, 47.5, 41.5, 37.3, 35.4, 33.6, 32.5, 30.6, 27.0 (3C), 21.6, 19.3 ppm; **IR** (ν_{max}, cm^{-1}) 3280 (w), 2929 (w), 2856 (w), 1672 (w), 1597 (w), 1427 (m), 1325 (m), 1153 (s), 1095 (s), 874 (m), 818 (m), 742 (m), 702 (s); **HRMS** (nanochip-ESI/LTQ-Orbitrap) m/z: [M + Na]$^+$ Calcd for $C_{33}H_{43}NNaO_3SSi^+$ 584.2625; Found 584.2638.

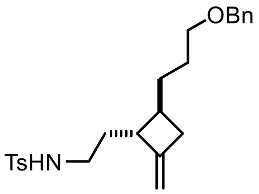

4j

***N*-(2-(2-(3-(Benzyloxy)propyl)-4-methylenecyclobutyl)ethyl)-4-methylbenzenesulfonamide (4j)**: Following the general procedure A, compound **4j** was isolated after purification by flash column chromatography on silica gel as a colorless oil. **^{1}H NMR** (400 MHz, $CDCl_3$) δ 7.75 (d, J = 8.3 Hz, 2H), 7.40 – 7.25 (m, 7H), 4.70 (q, J = 2.4 Hz, 1H), 4.66 (q, J = 2.4 Hz, 1H), 4.57 – 4.51 (m, 1H), 4.49 (s, 2H), 3.47 – 3.40 (m, 2H), 3.06 – 2.90 (m, 2H), 2.72 – 2.60 (m, 1H), 2.48 – 2.42 (m, 1H), 2.41 (s, 3H), 2.21-2.12 (m, 1H), 1.87 – 1.67 (m, 2H), 1.65 – 1.37 (m, 5H) ppm; **^{13}C NMR** (100 MHz, $CDCl_3$) δ 150.7, 143.4, 138.8, 137.4, 129.8 (2C), 128.5 (2C), 127.8 (2C), 127.6, 127.3 (2C), 104.7, 73.1, 70.4, 47.6, 41.5, 37.4, 35.4, 33.7, 32.8, 27.9, 21.6 ppm; **IR** (ν_{max}, cm^{-1}) 3278 (w), 2931 (w), 2856 (w), 1672 (w), 1599 (w), 1452 (m), 1325 (m), 1157 (s), 1088 (s), 874 (m), 814 (m), 742 (m), 700 (m), 661 (s); **HRMS** (ESI/QTOF) m/z: [M + Na]$^+$ Calcd for $C_{24}H_{31}NNaO_3S^+$ 436.1917; Found 436.1922.

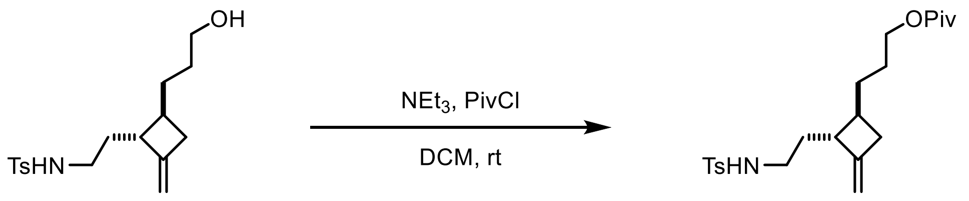

3-(3-Methylene-2-(2-((4-methylphenyl)sulfonamido)ethyl)cyclobutyl)propyl pivalate (4k): To a stirred solution of compound **4h-A** (50 mg, 0.155 mmol, 1.0 equiv) and triethylamine (33 µL, 0.233 mmol, 1.5 equiv) in anhydrous dichloromethane (3.1 mL, *c* = 0.05 mol/L) was added pivaloyl chloride (21 µL, 0.171 mmol, 1.1 equiv) at room temperature. The reaction mixture was stirred at room temperature until the starting material was completely consumed. The resulting mixture was quenched with saturated sodium bicarbonate and extracted with ethyl acetate (3×10 mL). The combined organic layers were washed with brine (2×10 mL), dried over anhydrous sodium sulfate, filtered and concentrated under vacuum. The residue was purified by flash column chromatography on silica gel to afford the desired compound **4k** as a colorless oil. **^{1}H NMR** (400 MHz, $CDCl_3$) δ 7.74 (d, *J* = 8.3 Hz, 2H), 7.29 (d, *J* = 7.7 Hz, 2H), 4.84 (t, *J* = 6.0 Hz, 1H), 4.68 (q, *J* = 2.3 Hz, 1H), 4.64 (q, *J* = 2.5 Hz, 1H), 3.98 (t, *J* = 6.3 Hz, 2H), 3.01 – 2.87 (m, 2H), 2.65 (ddq, *J* = 15.4, 8.5, 2.4 Hz, 1H), 2.48 – 2.41 (m, 1H), 2.41 (s, 3H), 2.12 (ddt, *J* = 15.3, 7.3, 2.6 Hz, 1H), 1.85 – 1.67 (m, 2H), 1.65 – 1.56 (m, 1H), 1.56 – 1.43 (m, 3H), 1.42 – 1.32 (m, 1H), 1.17 (s, 9H) ppm; **^{13}C NMR** (100 MHz, $CDCl_3$) δ 178.7, 150.3, 143.5, 137.0, 129.8 (2C), 127.2 (2C), 104.9, 64.2, 47.4, 41.4, 38.8, 37.1, 35.3, 33.5, 32.4, 27.3 (3C), 26.7, 21.6 ppm; **IR** (ν_{max}, cm^{-1}) 3273 (w), 2922 (m), 1724 (m), 1475 (w), 1437 (m), 1327 (m), 1286 (m), 1165 (s), 1093 (m), 874 (m), 814 (m), 714 (m), 661 (m); **HRMS** (ESI/QTOF) m/z: $[M + Na]^+$ Calcd for $C_{22}H_{33}NNaO_4S^+$ 430.2023; Found 430.2038.

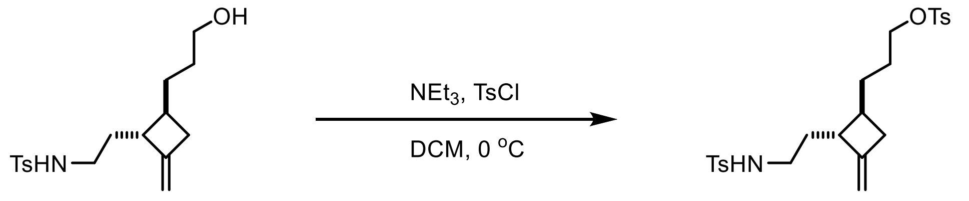

3-(3-Methylene-2-(2-((4-methylphenyl)sulfonamido)ethyl)cyclobutyl)propyl 4-methylbenzenesulfonate (4l): To a stirred solution of compound **4h-A** (255 mg, 0.788 mmol, 1.0 equiv) and triethylamine (164 µL, 1.18 mmol, 1.5 equiv) in anhydrous dichloromethane (7.9 mL, *c* = 0.10 mol/L) was added tosyl chloride (165 mg, 0.867 mmol, 1.1 equiv) at 0 ^{o}C.

The reaction mixture was stirred at 0 °C until the starting material was completely consumed. The resulting mixture was quenched with saturated sodium bicarbonate and extracted with ethyl acetate (3×15 mL). The combined organic layers were washed with brine (2×15 mL), dried over anhydrous sodium sulfate, filtered and concentrated under vacuum. The residue was purified by flash column chromatography on silica gel to afford the desired compound **4l** as a colorless oil (252 mg, 67%). **^{1}H NMR** (400 MHz, $CDCl_3$) δ 7.80 – 7.76 (m, 2H), 7.76 – 7.71 (m, 2H), 7.35 (d, J = 7.7 Hz, 2H), 7.31 (d, J = 7.7 Hz, 2H), 4.68 (q, J = 2.3 Hz, 1H), 4.65 (q, J = 2.5 Hz, 1H), 4.58 (t, J = 6.1 Hz, 1H), 3.97 (t, J = 6.2 Hz, 2H), 3.00 – 2.86 (m, 2H), 2.61 (ddq, J = 15.5, 8.5, 2.4 Hz, 1H), 2.44 (s, 3H), 2.42 (s, 3H), 2.44 – 2.38 (m, 1H), 2.08 (ddt, J = 15.3, 7.3, 2.6 Hz, 1H), 1.80 – 1.65 (m, 2H), 1.63 – 1.43 (m, 4H), 1.43 – 1.30 (m, 1H) ppm; **^{13}C NMR** (100 MHz, $CDCl_3$) δ 150.1, 145.0, 143.7, 137.0, 133.3, 130.1 (2C), 130.0 (2C), 128.1 (2C), 127.3 (2C), 105.1, 70.6, 47.4, 41.5, 36.9, 35.3, 33.6, 31.9, 27.1, 21.9, 21.7 ppm; **IR** (ν_{max}, cm^{-1}) 3292 (w), 2933 (w), 1666 (w), 1601 (w), 1421 (w), 1327 (m), 1151 (s), 1086 (m), 939 (m), 877 (m), 814 (s), 658 (s); **HRMS** (ESI/QTOF) m/z: [M + H]$^+$ Calcd for $C_{24}H_{32}NO_5S_2^+$ 478.1716; Found 478.1730.

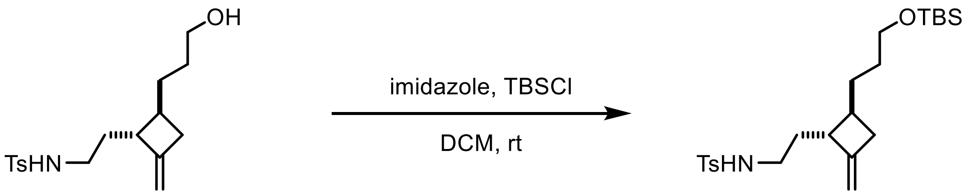

4h-A 4m

***N*-(2-(2-(3-((*tert*-Butyldimethylsilyl)oxy)propyl)-4-methylenecyclobutyl)ethyl)-4-methylbenzenesulfonamide (4m)**: To a stirred solution of compound **4h-A** (80 mg, 0.247 mmol, 1.0 equiv) and imidazole (33.5 mg, 0.49 mmol, 2.0 equiv) in anhydrous dichloromethane (5 mL, c = 0.05 mol/L) was added *tert*-butyldimethylsilyl chloride (41 mg, 0.272 mmol, 1.1 equiv) at room temperature. The reaction mixture was stirred at room temperature until the starting material was completely consumed. The resulting mixture was quenched with saturated sodium bicarbonate and extracted with ethyl acetate (3×10 mL). The combined organic layers were washed with brine (2×10 mL), dried over anhydrous sodium sulfate, filtered and concentrated under vacuum. The residue was purified by flash column chromatography on silica gel to afford the desired compound **4m** as a colorless oil. **^{1}H NMR** (400 MHz, $CDCl_3$) δ 7.75 (d, J = 8.3 Hz, 2H), 7.30 (d, J = 7.9 Hz, 2H), 4.69 (q, J = 2.3 Hz, 1H), 4.64 (q, J = 2.5 Hz, 1H), 4.61 (t, J = 6.6 Hz, 1H), 3.58 – 3.51 (m, 2H), 3.03 – 2.89 (m,

2H), 2.64 (ddq, J = 15.5, 8.5, 2.4 Hz, 1H), 2.42 (s, 3H), 2.42 – 2.37 (m, 1H), 2.14 (ddt, J = 15.3, 7.4, 2.6 Hz, 1H), 1.84 – 1.67 (m, 2H), 1.65 – 1.54 (m, 1H), 1.50 – 1.31 (m, 4H), 0.88 (s, 9H), 0.03 (s, 6H) ppm; **^{13}C NMR** (100 MHz, $CDCl_3$) δ 150.7, 143.5, 137.0, 129.8 (2C), 127.2 (2C), 104.6, 63.2, 47.5, 41.5, 37.4, 35.4, 33.6, 32.5, 30.9, 26.1 (3C), 21.7, 18.5, -5.1 (2C) ppm; **IR** (ν_{max}, cm^{-1}) 3284 (w), 2935 (w), 2858 (w), 1672 (w), 1597 (w), 1464 (w), 1442 (w), 1325 (m), 1255 (m), 1165 (s), 1093 (s), 835 (s), 775 (s), 717 (m), 665 (s); **HRMS** (nanochip-ESI/LTQ-Orbitrap) m/z: $[M + Na]^+$ Calcd for $C_{23}H_{39}NNaO_3SSi^+$ 460.2312; Found 460.2322.

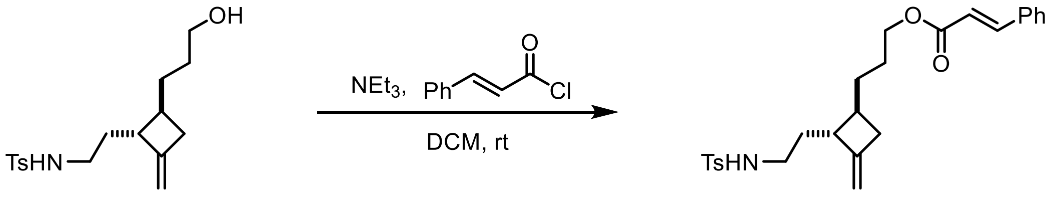

4h-A **4n**

3-(3-Methylene-2-(2-((4-methylphenyl)sulfonamido)ethyl)cyclobutyl)propyl cinnamate (4n): To a stirred solution of compound **4h-A** (85.1 mg, 0.263 mmol, 1.0 equiv) and triethylamine (54.8 µL, 0.394 mmol, 1.5 equiv) in anhydrous dichloromethane (5.3 mL, c = 0.05 mol/L) was added cinnamoyl chloride (48.2 mg, 0.289 mmol, 1.1 equiv) at room temperature. The reaction mixture was stirred at room temperature until the starting material was completely consumed. The resulting mixture was quenched with saturated sodium bicarbonate and extracted with ethyl acetate (3×10 mL). The combined organic layers were washed with brine (2×10 mL), dried over anhydrous sodium sulfate, filtered and concentrated under vacuum. The residue was purified by flash column chromatography on silica gel to afford the desired compound **4n** as a colorless oil. **^{1}H NMR** (400 MHz, $CDCl_3$) δ 7.75 (d, J = 8.3 Hz, 2H), 7.69 (d, J = 16.0 Hz, 1H), 7.57 – 7.51 (m, 2H), 7.42 – 7.36 (m, 3H), 7.30 (d, J = 8.0 Hz, 2H), 6.45 (d, J = 16.0 Hz, 1H), 4.71 (q, J = 2.3 Hz, 1H), 4.67 (q, J = 2.5 Hz, 1H), 4.56 (t, J = 6.1 Hz, 1H), 4.21– 4.11 (m, 2H), 3.06– 2.92 (m, 2H), 2.74 – 2.64 (m, 1H), 2.52 – 2.44 (m, 1H), 2.41 (s, 3H), 2.18 (ddt, J = 15.3, 7.3, 2.6 Hz, 1H), 1.90 – 1.80 (m, 1H), 1.80 – 1.70 (m, 1H), 1.69 – 1.52 (m, 4H), 1.50 – 1.40 (m, 1H) ppm; **^{13}C NMR** (100 MHz, $CDCl_3$) δ 167.2, 150.4, 144.9, 143.6, 137.0, 134.5, 130.4, 129.8 (2C), 129.0 (2C), 128.2 (2C), 127.2 (2C), 118.2, 104.9, 64.5, 47.5, 41.4, 37.1, 35.3, 33.6, 32.5, 26.9, 21.6 ppm; **IR** (ν_{max}, cm^{-1}) 3276 (m), 2922 (m), 1711 (m), 1633 (m), 1452 (m), 1323 (m), 1286 (m), 1163 (s), 1093 (m), 980 (m), 874 (m), 818 (m), 764 (m), 715 (s), 658 (s); **HRMS** (nanochip-ESI/LTQ-Orbitrap) m/z: $[M + Na]^+$ Calcd for $C_{26}H_{31}NNaO_4S^+$ 476.1866; Found 476.1882.

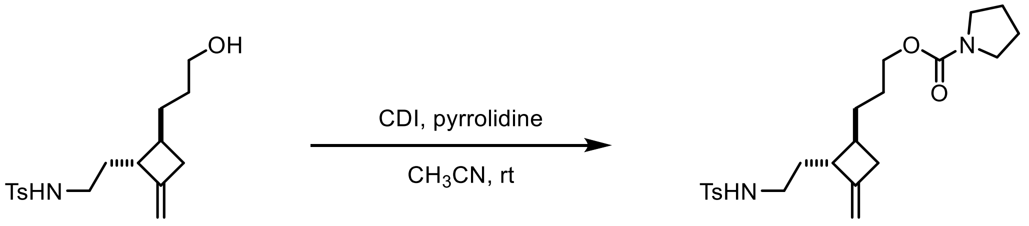

4h-A **4o**

3-(3-Methylene-2-(2-((4-methylphenyl)sulfonamido)ethyl)cyclobutyl)propyl pyrrolidine-1-carboxylate (4o): To a stirred solution of compound **4h-A** (90 mg, 0.278 mmol, 1.0 equiv) in anhydrous acetonitrile (5.6 mL, *c* = 0.05 mol/L) was added 1,1'-carbonyldiimidazole (58.5 mg, 0.361 mmol, 1.3 equiv) at room temperature. After stirring for 1 h, pyrrolidine (46.4 µL, 0.556 mmol, 2.0 equiv) was added to the above mixture. The reaction mixture was stirred at room temperature for 1 h. The solvent was removed under vacuum. The residue was dissolved in dichloromethane (15 mL), and then washed sequentially with 1*N* HCl (2×10 mL), saturated sodium bicarbonate (1×10 mL) and brine (1×10 mL). The organic layer was dried over anhydrous sodium sulfate, filtered and concentrated under vacuum. The residue was purified by flash column chromatography on silica gel (0% to 15% to 25% ethyl acetate-hexane) to afford the desired compound **4o** as a colorless oil (73.6 mg, 63%). **^{1}H NMR** (400 MHz, $CDCl_3$) δ 7.77 – 7.72 (m, 2H), 7.30 (d, *J* = 8.0 Hz, 2H), 4.72 (t, *J* = 6.1 Hz, 1H), 4.69 (q, *J* = 2.4 Hz, 1H), 4.65 (q, *J* = 2.5 Hz, 1H), 4.07 – 3.95 (m, 2H), 3.38 (t, *J* = 6.4 Hz, 2H), 3.31 (t, *J* = 6.4 Hz, 2H), 3.04 – 2.89 (m, 2H), 2.66 (ddq, *J* = 15.5, 8.5, 2.4 Hz, 1H), 2.50 – 2.41 (m, 1H), 2.42 (s, 3H), 2.14 (ddt, *J* = 15.3, 7.3, 2.6 Hz, 1H), 1.92 – 1.79 (m, 5H), 1.78 – 1.68 (m, 1H), 1.67 – 1.35 (m, 5H) ppm; **^{13}C NMR** (100 MHz, $CDCl_3$) δ 155.3, 150.5, 143.3, 137.1, 129.7 (2C), 127.1 (2C), 104.7, 64.8, 47.4, 46.2, 45.8, 41.3, 37.1, 35.3, 33.5, 32.4, 27.2, 25.8, 25.0, 21.6 ppm; **IR** (ν_{max}, cm^{-1}) 3219 (w), 2929 (w), 2877 (w), 1676 (s), 1593 (w), 1429 (s), 1331 (s), 1157 (s), 1095 (s), 870 (m), 816 (m), 769 (m), 731 (m), 661 (s); **HRMS** (nanochip-ESI/LTQ-Orbitrap) m/z: $[M + Na]^+$ Calcd for $C_{22}H_{32}N_2NaO_4S^+$ 443.1975; Found 443.1983.

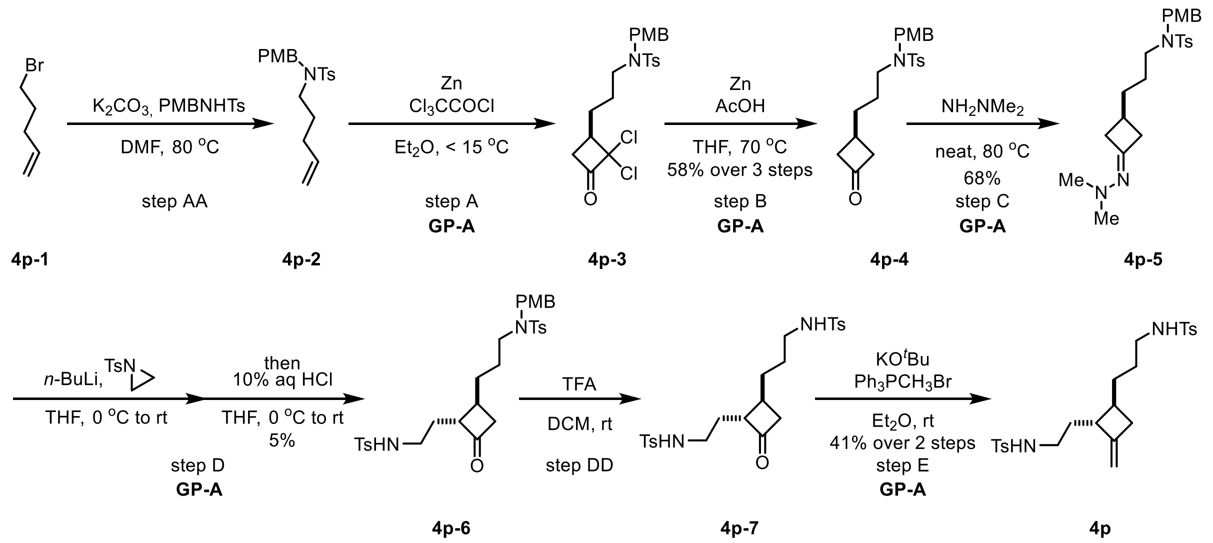

Step AA: Following the known literature procedure,[5] to a stirred solution of *N*-(4-methoxybenzyl)-4-methylbenzenesulfonamide (5.0 g, 17.2 mmol, 1.0 equiv) and 5-bromopent-1-ene **4p-1** (3.32 g, 22.3 mmol, 1.3 equiv) in anhydrous *N,N*-dimethylformamide (57 mL, *c* = 0.3 mol/L) was added anhydrous potassium carbonate (9.51 g, 68.8 mmol, 4.0 equiv) in one portion. The reaction mixture was stirred at 80 °C for 7 h. The resulting mixture was quenched with water and extracted with ethyl acetate (3×40 mL). The combined organic layers were washed with water (3×50 mL) and brine (3×50 mL), dried over anhydrous sodium sulfate, filtered and concentrated under vacuum. The residue **4p-2** was directly used in the next step without further purification.

Following the steps AA, A and B of general procedure A, compound **4p-4** was isolated after purification by flash column chromatography on silica gel as colorless oil (4.0 g, 58% over 3 steps); Following the step C of general procedure A, compound **4p-5** was isolated after purification by flash column chromatography on silica gel as colorless oil (3.0 g, 68%); Following the step D (2.0 g scale) of general procedure A, compound **4p-6** was isolated after purification by flash column chromatography on silica gel as colorless oil (140 mg, 5%).

Step DD: Following the known literature procedure,[6] To a stirred solution of compound **4p-6** (140 mg, 0.234 mmol, 1.0 equiv) in anhydrous dichloromethane (3 mL) was added trifluoroacetic acid (1.0 mL) at room temperature, the reaction mixture was stirred at room temperature for 12 h. The solvent was removed under vacuum. The resulting residue was diluted with ethyl acetate and saturated sodium bicarbonate, extracted with ethyl acetate (3×10 mL). The combined organic layers were washed with water (10 mL) and brine (10 mL), dried

over anhydrous sodium sulfate, filtered and concentrated under vacuum. The residue **4p-7** was directly used in the next step without further purification.

Following the step E of general procedure A, compound **4p** was isolated after purification by flash column chromatography on silica gel as colorless oil (46 mg, 41% over 2 steps).

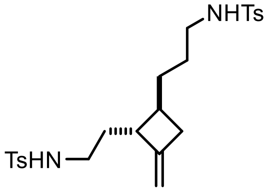

4p

4-Methyl-*N*-(3-((1S,2R)-3-methylene-2-(2-((4-methylphenyl)sulfonamido)ethyl)cyclobutyl)propyl)benzenesulfonamide (4p): **^{1}H NMR** (400 MHz, $CDCl_3$) δ 7.75 (d, *J* = 7.8 Hz, 4H), 7.32 (d, *J* = 8.0 Hz, 2H), 7.31 (d, *J* = 8.1 Hz, 2H), 4.69 (q, *J* = 2.3 Hz, 1H), 4.68 – 4.63 (m, 2H), 4.55 (t, *J* = 6.1 Hz, 1H), 2.98 – 2.84 (m, 4H), 2.62 (ddq, *J* = 15.3, 8.3, 2.3 Hz, 1H), 2.50 – 2.40 (m, 1H), 2.43 (s, 6H), 2.09 (ddt, *J* = 15.2, 7.4, 2.6 Hz, 1H), 1.85 – 1.67 (m, 2H), 1.64 – 1.55 (m, 1H), 1.55 – 1.46 (m, 1H), 1.46 – 1.30 (m, 3H) ppm; **^{13}C NMR** (100 MHz, $CDCl_3$) δ 150.3, 143.6, 143.5, 137.1, 136.8, 129.91 (2C), 129.86 (2C), 127.2 (4C), 104.8, 47.3, 43.2, 41.5, 37.0, 35.3, 33.5, 32.9, 27.6, 21.7 (2C) ppm; **IR** (ν_{max}, cm^{-1}) 3284 (w), 2925 (w), 2854 (w), 1599 (w), 1425 (w), 1321 (m), 1157 (s), 1093 (m), 812 (m), 661 (m); **HRMS** (ESI/QTOF) m/z: $[M + Na]^+$ Calcd for $C_{24}H_{32}N_2NaO_4S_2^+$ 499.1696; Found 499.1697.

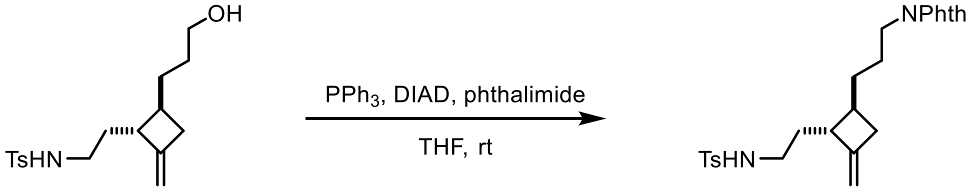

4h-A **4q**

***N*-(2-(2-(3-(1,3-Dioxoisoindolin-2-yl)propyl)-4-methylenecyclobutyl)ethyl)-4-methylbenzenesulfonamide (4q)**: To a stirred solution of compound **4h-A** (50 mg, 0.155 mmol, 1.0 equiv), triphenylphosphine (81.3 mg, 0.31 mmol, 2.0 equiv) and phthalimide (45.6 mg, 0.31 mmol, 2.0 equiv) in anhydrous tetrahydrofuran (3.1 mL, *c* = 0.05 mol/L) was added diisopropyl azodicarboxylate (61 µL, 0.31 mmol, 2.0 equiv) at room temperature. The reaction

mixture was stirred overnight at room temperature. Then silica gel was added to the above mixture and the resulting mixture was concentrated under vacuum. The residue was loaded onto a silica gel column and purified by flash column chromatography on silica gel to afford the desired compound **4q** as a colorless oil. **^{1}H NMR** (400 MHz, $CDCl_3$) δ 7.85 (dd, *J* = 5.4, 3.1 Hz, 2H), 7.76 – 7.73 (m, 2H), 7.72 (dd, *J* = 5.4, 3.1 Hz, 2H), 7.30 (d, *J* = 7.9 Hz, 2H), 4.70 (q, *J* = 2.3 Hz, 1H), 4.65 (q, *J* = 2.5 Hz, 1H), 4.48 (t, *J* = 6.0 Hz, 1H), 3.64 (t, *J* = 7.2 Hz, 2H), 3.05 – 2.91 (m, 2H), 2.72 – 2.61 (m, 1H), 2.49 – 2.40 (m, 1H), 2.42 (s, 3H), 2.14 (ddt, *J* = 15.2, 7.3, 2.6 Hz, 1H), 1.93– 1.83 (m, 1H), 1.78– 1.68 (m, 1H), 1.68 – 1.34 (m, 5H) ppm; **^{13}C NMR** (100 MHz, $CDCl_3$) δ 168.6 (2C), 150.3, 143.4, 137.0, 134.0 (2C), 132.2 (2C), 129.8 (2C), 127.2 (2C), 123.3 (2C), 104.8, 47.4, 41.4, 37.8, 36.7, 35.3, 33.4, 33.2, 26.5, 21.6 ppm; **IR** (ν_{max}, cm^{-1}) 3280 (w), 2925 (w), 1774 (w), 1701 (s), 1433 (w), 1404 (m), 1319 (m), 1155 (s), 1090 (m), 874 (m), 818 (m), 723 (s), 665 (m); **HRMS** (ESI/QTOF) m/z: $[M + Na]^+$ Calcd for $C_{25}H_{28}N_2NaO_4S^+$ 475.1662; Found 475.1664.

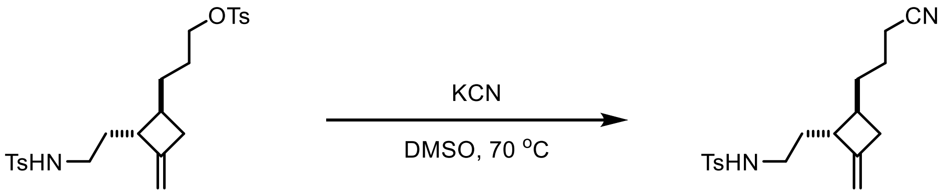

4l 4r

***N*-(2-(3-Cyanopropyl)-4-methylenecyclobutyl)ethyl)-4-methylbenzenesulfonamide (4r)**: To a stirred solution of compound **4l** (1.0 equiv) in anhydrous dimethyl sulfoxide was added potassium cyanide (20 equiv) at room temperature. The reaction mixture was allowed to stir overnight at 70 °C. The resulting mixture was diluted with water and extracted three times with diethyl ether. The combined organic layers were washed with water and brine, dried over anhydrous sodium sulfate, filtered and concentrated under vacuum. The residue was purified by flash column chromatography on silica gel to afford the desired compound **4r** as a colorless oil. **^{1}H NMR** (400 MHz, $CDCl_3$) δ 7.75 (d, *J* = 8.3 Hz, 2H), 7.32 (d, *J* = 8.1 Hz, 2H), 4.78 – 4.71 (m, 1H), 4.71 (q, *J* = 2.4 Hz, 1H), 4.67 (q, *J* = 2.5 Hz, 1H), 3.02 – 2.88 (m, 2H), 2.74 – 2.63 (m, 1H), 2.54 – 2.45 (m, 1H), 2.43 (s, 3H), 2.34 – 2.27 (m, 2H), 2.17 (ddt, *J* = 15.2, 7.3, 2.6 Hz, 1H), 1.88 – 1.79 (m, 1H), 1.79 – 1.69 (m, 1H), 1.69 – 1.44 (m, 5H) ppm; **^{13}C NMR** (100 MHz, $CDCl_3$) δ 149.8, 143.6, 136.9, 129.9 (2C), 127.2 (2C), 119.8, 105.2, 47.3, 41.4, 36.7, 35.1, 35.0, 33.5, 23.5, 21.6, 17.3 ppm; **IR** (ν_{max}, cm^{-1}) 3267 (w), 2929 (w), 1674 (w),

1597 (w), 1425 (w), 1325 (m), 1157 (s), 1093 (m), 879 (m), 816 (m), 667 (s); **HRMS** (ESI/QTOF) m/z: $[M + H]^+$ Calcd for $C_{18}H_{25}N_2O_2S^+$ 333.1631; Found 333.1632.

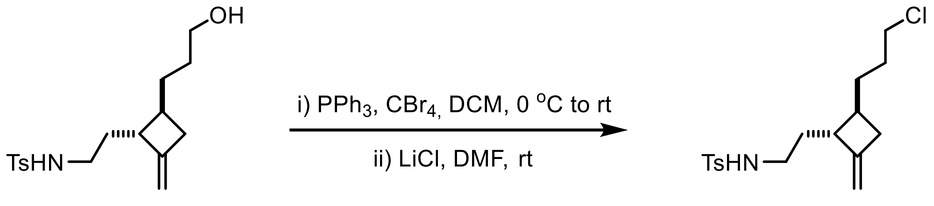

4h-A **4s**

***N*-(2-(2-(3-Chloropropyl)-4-methylenecyclobutyl)ethyl)-4-methylbenzenesulfonamide (4s)**: To a stirred solution of compound **4h-A** (200 mg, 0.618 mmol, 1.0 equiv) and triphenylphosphine (162 mg, 0.618 mmol, 1.0 equiv) in anhydrous dichloromethane (7.9 mL, c = 0.10 mol/L) was added carbon tetrabromide (205 mg, 0.618 mmol, 1.0 equiv) at 0 °C. After stirring at 0 °C for 1 h, the reaction mixture was allowed to stir at room temperature until the starting material was completely consumed. The resulting mixture was concentrated under vacuum. The residue was used in the next step without further purification. To a stirred solution of the half of above residue (0.309 mmol, 1.0 equiv) in anhydrous *N*,*N*-dimethylformamide (3 mL, c = 0.10 mol/L) was added lithium chloride (262 mg, 6.18 mmol, 20 equiv) at room temperature. The reaction mixture was stirred at room temperature until the starting material was completely consumed. The resulting mixture was diluted with water and extracted with diethyl ether (3×10 mL). The combined organic layers were washed with water (3×10 mL) and brine (3×10 mL), dried over anhydrous sodium sulfate, filtered and concentrated under vacuum. The residue was purified by flash column chromatography on silica gel to afford the desired compound **4s** as a colorless oil. **^{1}H NMR** (400 MHz, $CDCl_3$) δ 7.75 (d, J = 8.3 Hz, 2H), 7.31 (d, J = 8.1 Hz, 2H), 4.70 (q, J = 2.3 Hz, 1H), 4.70 – 4.65 (m, 1H), 4.66 (q, J = 2.6 Hz, 1H), 3.48 (td, J = 6.5, 1.3 Hz, 2H), 3.04 – 2.86 (m, 2H), 2.66 (ddq, J = 15.5, 8.4, 2.4 Hz, 1H), 2.51 – 2.41 (m, 1H), 2.43 (s, 3H), 2.16 (ddt, J = 15.2, 7.3, 2.6 Hz, 1H), 1.86 – 1.53 (m, 6H), 1.52 – 1.41 (m, 1H) ppm; **^{13}C NMR** (100 MHz, $CDCl_3$) δ 150.2, 143.6, 136.9, 129.9 (2C), 127.2 (2C), 104.9, 47.4, 45.0, 41.4, 36.9, 35.3, 33.5, 33.4, 30.7, 21.7 ppm; **IR** (ν_{max}, cm^{-1}) 3273 (w), 2918 (w), 1668 (w), 1599 (w), 1439 (w), 1323 (m), 1157 (s), 1092 (m), 876 (m), 814 (m), 717 (m), 661 (s); **HRMS** (ESI/QTOF) m/z: $[M + Na]^+$ Calcd for $C_{17}H_{24}ClNNaO_2S^+$ 364.1108; Found 364.1105.

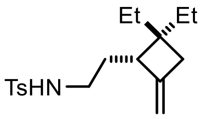

4t

***N*-(2-(2,2-Diethyl-4-methylenecyclobutyl)ethyl)-4-methylbenzenesulfonamide (4t)**: Following the general procedure A, compound **4t** was isolated after purification by flash column chromatography on silica gel as a pale yellow oil. **^{1}H NMR** (400 MHz, $CDCl_3$) δ 7.75 (d, *J* = 8.3 Hz, 2H), 7.29 (d, *J* = 8.1 Hz, 2H), 4.96 – 4.77 (m, 1H), 4.67 (q, *J* = 2.3 Hz, 1H), 4.64 (q, *J* = 2.5 Hz, 1H), 3.01-2.88 (m, 2H), 2.48 – 2.41 (m, 1H), 2.41 (s, 3H), 2.27 – 2.10 (m, 2H), 1.62 – 1.51 (m, 2H), 1.46 (dq, *J* = 14.8, 7.5 Hz, 1H), 1.41 – 1.19 (m, 3H), 0.71 (t, *J* = 7.4 Hz, 6H) ppm; **^{13}C NMR** (100 MHz, $CDCl_3$) δ 150.7, 143.4, 137.1, 129.8 (2C), 127.2 (2C), 105.1, 48.7, 42.5, 41.0, 40.3, 30.5, 29.2, 23.4, 21.6, 8.7, 8.2 ppm; **IR** (ν_{max}, cm^{-1}) 3278 (w), 2961 (w), 1324 (m), 1157 (s), 1094 (m), 814 (m), 661 (m), 550 (m); **HRMS** (nanochip-ESI/LTQ-Orbitrap) m/z: $[M + Na]^+$ Calcd for $C_{18}H_{27}NNaO_2S^+$ 344.1655; Found 344.1666.

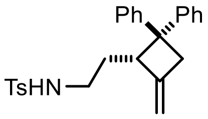

4u

4-Methyl-*N*-(2-(4-methylene-2,2-diphenylcyclobutyl)ethyl)benzenesulfonamide (4u): Following the general procedure A, compound **4u** was isolated after purification by flash column chromatography on silica gel as a colorless oi. **^{1}H NMR** (400 MHz, $CDCl_3$) δ 7.70 (d, *J* = 8.4 Hz, 2H), 7.35 – 7.29 (m, 2H), 7.28 (d, *J* = 8.3 Hz, 2H), 7.25 – 7.11 (m, 6H), 7.06 – 7.00 (m, 2H), 4.91 (q, *J* = 2.3 Hz, 1H), 4.81 (q, *J* = 2.5 Hz, 1H), 4.52 – 4.29 (m, 1H), 3.66 (td, *J* = 7.2, 2.6 Hz, 1H), 3.50 (dq, *J* = 15.3, 2.1 Hz, 1H), 3.11 (dt, *J* = 15.3, 2.7 Hz, 1H), 3.01 (ddt, *J* = 12.8, 7.9, 6.4 Hz, 1H), 2.91 (dtd, *J* = 12.5, 7.5, 6.0 Hz, 1H), 2.42 (s, 3H), 1.38 – 1.23 (m, 2H) ppm; **^{13}C NMR** (100 MHz, $CDCl_3$) δ 149.3, 149.1, 144.2, 143.4, 136.9, 129.8 (2C), 128.4 (2C), 127.9 (2C), 127.8 (2C), 127.2 (2C), 127.1 (2C), 126.2, 126.1, 105.1, 50.7, 50.2, 44.6, 41.7, 31.0, 21.6 ppm; **IR** (ν_{max}, cm^{-1}) 3276 (w), 2923 (w), 1598 (w), 1445 (w), 1324 (m), 1155 (s), 1093 (m), 814 (m), 754 (m), 699 (s), 661 (m), 550 (s); **HRMS** (nanochip-ESI/LTQ-Orbitrap) m/z: $[M + Na]^+$ Calcd for $C_{26}H_{27}NNaO_2S^+$ 440.1655; Found 440.1669.

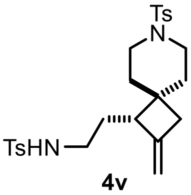

4v

4-Methyl-*N*-(2-(2-methylene-7-tosyl-7-azaspiro[3.5]nonan-1-yl)ethyl)benzenesulfonamide (4v): Following the general procedure A, compound **4v** was isolated after purification by flash column chromatography on silica gel as a white solid. m.p. = 150 ~ 152 °C; **^{1}H NMR** (400 MHz, $CDCl_3$) *δ* 7.74 (d, *J* = 8.3 Hz, 2H), 7.62 (d, *J* = 8.3 Hz, 2H), 7.32 (d, *J* = 8.2 Hz, 2H), 7.31 (d, *J* = 8.2 Hz, 2H), 4.71-4.67 (m, 2H), 4.56 (t, *J* = 6.2 Hz, 1H), 3.61 – 3.50 (m, 2H), 2.99 – 2.84 (m, 2H), 2.51 – 2.45 (m, 1H), 2.44 (s, 3H), 2.43 (s, 3H), 2.32 – 2.18 (m, 2H), 2.15-2.12 (m, 2H), 1.65 (ddd, *J* = 13.9, 12.0, 4.6 Hz, 1H), 1.59 – 1.51 (m, 3H), 1.50 – 1.43 (m, 2H) ppm; **^{13}C NMR** (100 MHz, $CDCl_3$) δ 148.1, 143.7, 143.6, 136.9, 133.3, 129.9 (2C), 129.78 (2C), 127.76 (2C), 127.2 (2C), 106.1, 49.4, 43.7, 43.1, 42.0, 39.7, 37.3, 36.6, 30.1, 28.3, 21.7 (2C) ppm; **IR** (ν_{max}, cm^{-1}) 3292 (w), 2922 (w), 2844 (w), 2360 (w), 1672 (w), 1597 (w), 1425 (w), 1325 (m), 1159 (s), 1093 (m), 947 (m), 901 (w), 816 (m), 729 (m), 656 (m); **HRMS** (ESI/QTOF) m/z: $[M + H]^+$ Calcd for $C_{25}H_{33}N_2O_4S_2^+$ 489.1876; Found 489.1887.

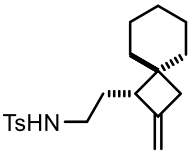

4w

4-Methyl-*N*-(2-(2-methylenespiro[3.5]nonan-1-yl)ethyl)benzenesulfonamide (4w): Following the general procedure A, compound **4w** was isolated after purification by flash column chromatography on silica gel as a colorless oil. **^{1}H NMR** (400 MHz, $CDCl_3$) δ 7.80 – 7.73 (m, 2H), 7.29 (d, *J* = 8.1 Hz, 2H), 4.98 (t, *J* = 6.1 Hz, 1H), 4.67 (q, *J* = 2.2 Hz, 1H), 4.62 (q, *J* = 2.5 Hz, 1H), 2.92 (td, *J* = 7.4, 5.9 Hz, 2H), 2.41 (s, 3H), 2.33 (tq, *J* = 7.5, 2.6 Hz, 1H), 2.29 – 2.16 (m, 2H), 1.61 – 1.42 (m, 6H), 1.42 – 1.34 (m, 1H), 1.32 – 1.13 (m, 3H), 1.13 – 1.01 (m, 2H) ppm; **^{13}C NMR** (100 MHz, $CDCl_3$) δ 150.3, 143.4, 137.0, 129.7 (2C), 127.2 (2C), 104.7, 50.2, 42.3, 40.8, 39.3, 38.7, 30.8, 28.1, 26.1, 23.4, 22.8, 21.6 ppm; **IR** (ν_{max}, cm^{-1}) 3270 (m), 2920 (m), 2854 (m), 1673 (m), 1599 (m), 1436 (m), 1323 (m), 1156 (s), 1094 (s), 875 (m),

813 (s), 660 (s); **HRMS** (ESI/QTOF) m/z: $[M + Na]^+$ Calcd for $C_{19}H_{27}NNaO_2S^+$ 356.1655; Found 356.1653

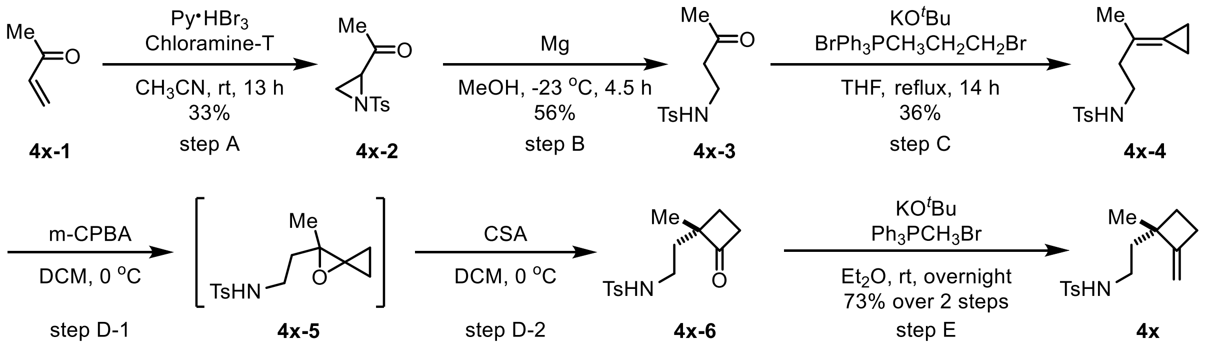

Step A: Following the known literature procedure,[7] to a stirred solution of methyl vinyl ketone **4x-1** (2.8 g, 40 mmol, 1.0 equiv) and anhydrous Chloramine-T (9.56 g, 42 mmol, 1.05 equiv) in anhydrous acetonitrile (200 mL, *c* = 0.2 mol/L) was added pyridinium hydrobromide perbromide (1.28 g, 4.0 mmol, 0.1 equiv) at room temperature. The reaction mixture was stirred vigorously at room temperature for 13 h. The reaction mixture was concentrated under vacuum. The residue was purified by flash column chromatography on silica gel (0% to 15% ethyl acetate-hexane) to afford the desired compound **4x-2** as a pale brown oil (3.18 g, 33%).

Step B: Following the known literature procedure,[8] to a stirred suspension of magnesium turnings (965 mg, 39.7 mmol, 3.0 equiv) in anhydrous methanol (43 mL, *c* = 0.25 mol/L) was added compound **4x-2** (3.18 g, 13.23 mmol, 1.0 equiv) in anhydrous methanol (10 mL) dropwise at -23 °C. The reaction mixture was stirred at -23 °C for 4.5 h. The reaction mixture was diluted with ethyl acetate, filtered through a short pad of silica gel and rinsed with ethyl acetate. The filtrate was concentrated to dryness under vacuum and the residue was purified by flash column chromatography on silica gel (0% to 2% methanol-dichloromethane) to afford desired compound **4x-3** as a pale brown oil (1.8 g, 56%).

Step C: Following the known literature procedure,[9] to a stirred suspension of (3-bromopropyl)triphenylphosphonium bromide (5.19 g, 11.19 mmol, 2.0 equiv) in tetrahydrofuran (38 mL, *c* = 0.15 mol/L) under N_2 atmosphere was added potassium *tert*-butoxide (2.51 g, 22.38 mmol, 4.0 equiv), and the reaction mixture turned to bright orange. The reaction mixture was then heated to reflux for 2 h and compound **4x-3** (1.35 g, 5.59 mmol, 1.0 equiv) was then added. The resulting mixture was continued to reflux overnight. The reaction mixture was quenched by the addition of saturated ammonium chloride. The resulting mixture was extracted with ethyl acetate (3×20 mL). The combined organic layers were washed with

brine (2×20 mL), dried over anhydrous sodium sulfate, filtered and concentrated under vacuum. The residue was purified by flash column chromatography on silica gel (0% to 2% to 5% to 10% ethyl acetate-hexane) to afford desired compound **4x-4** as a pale yellow oil (540 mg, 36%).

Step D: Following the known literature procedure,[9] to a stirred solution of compound **4x-4** (535 mg, 2.02 mmol, 1.0 equiv) in anhydrous dichloromethane (10 mL, *c* = 0.2 mol/L) was added 3-chloroperoxybenzoic acid (75% in water, 511 mg, 2.22 mmol, 1.1 equiv) in 5 portions over a 15 min period at 0 °C. After stirring at 0 °C for 30 min, and disappearance of starting material, camphor sulfonic acid (23.2 mg, 0.1 mmol, 0.05 equiv) was added to ensure a total conversion of epoxides **4x-5** to cyclobutanone **4x-6**, and the reaction mixture was stirred for another 15 min. The reaction mixture was quenched with a 10 % Na_2SO_3 solution (10 mL) and a small amount of 2% KI solution (2 mL). The mixture was stirred vigorously for 20 min at room temperature and extracted with diethyl ether (3×20 mL). The combined organic layers were washed with saturated sodium bicarbonate (3×30 mL) and brine (1×30 mL), dried over anhydrous sodium sulfate, filtered and concentrated under vacuum. The residue **4x-6** was used in the next step without further purification.

Step E: Potassium *tert*-butoxide (792 mg, 7.1 mmol, 3.5 equiv) was added to Ph_3PCH_3Br (2.52 g, 7.1 mmol, 3.5 equiv) in anhydrous diethyl ether (15 mL, *c* = 0.13 mol/L) at room temperature. The reaction mixture was stirred at room temperature for 2 h. Then the resulting solution was transferred to a round-bottom flask charged with the above crude **4x-6** (2.02 mmol, 1.0 equiv) at room temperature. The reaction mixture was stirred overnight at room temperature. TLC showed that the starting material was consumed completely, then the reaction mixture was quenched by the addition of saturated ammonium chloride. The resulting mixture was extracted with diethyl ether (3×20 mL). The combined organic layers were washed with brine (2×30 mL), dried over anhydrous sodium sulfate, filtered and concentrated under vacuum. The residue was purified by flash column chromatography on silica gel (0% to 5% to 15% ethyl acetate-hexane) to give compound **4x** (411 mg, 73% over 2 steps).

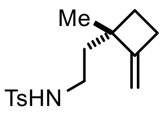

4x

4-Methyl-*N*-(2-(1-methyl-2-methylenecyclobutyl)ethyl)benzenesulfonamide (4x): **^{1}H NMR** (400 MHz, $CDCl_3$) δ 7.75 (d, J = 8.4 Hz, 2H), 7.30 (d, J = 8.1 Hz, 2H), 4.82 (brs, 1H), 4.66 (t, J = 2.3 Hz, 1H), 4.63 (t, J = 2.6 Hz, 1H), 2.98 (dt, J = 8.7, 6.2 Hz, 2H), 2.60 – 2.42 (m, 2H), 2.42 (s, 3H), 1.74 (ddd, J = 11.1, 9.4, 6.2 Hz, 1H), 1.68 – 1.57 (m, 3H), 1.06 (s, 3H) ppm; **^{13}C NMR** (100 MHz, $CDCl_3$) δ 157.7, 143.4, 137.0, 129.8 (2C), 127.2 (2C), 103.9, 46.2, 40.0, 39.2, 30.1, 26.9, 25.3, 21.6 ppm; **IR** (ν_{max}, cm^{-1}) 3280 (w), 2952 (w), 2923 (w), 1425 (w), 1323 (m), 1155 (s), 1093 (m), 875 (m), 814 (m), 663 (m), 551 (s); **HRMS** (nanochip-ESI/LTQ-Orbitrap) m/z: $[M + Na]^+$ Calcd for $C_{15}H_{21}NNaO_2S^+$ 302.1185; Found 302.1196.

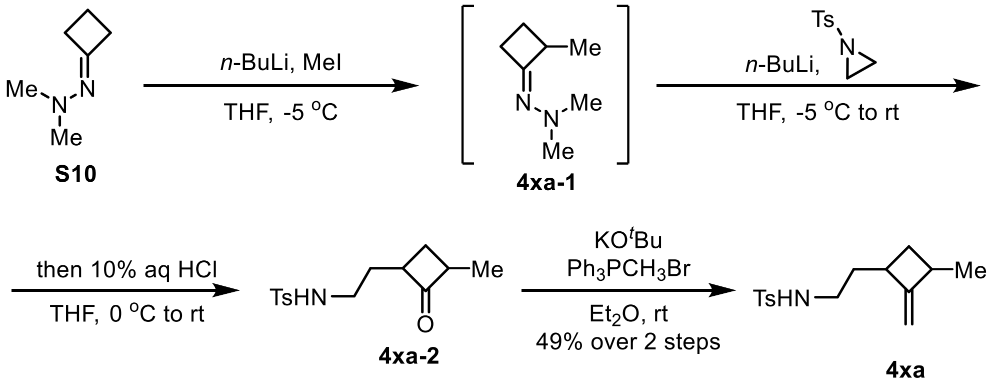

The hydrazone **S10** (320 mg, 2.85 mmol, 1.0 equiv) was dissolved in anhydrous tetrahydrofuran (11 mL, c = 0.25 mol/L) and the resulting mixture was cooled to -5 °C, then *n*-butyllithium (2.5 M in hexane, 1.2 mL, 2.99 mmol, 1.05 equiv) was added dropwise. The resulting mixture was stirred for 1 h at -5 °C and iodomethane (195 µL, 3.14 mmol, 1.1 equiv) was added slowly. After stirring at -5 °C for 1 h to generate compound **4xa-1**, additional *n*-butyllithium (2.5 M in hexane, 1.2 mL, 2.99 mmol, 1.05 equiv) was added dropwise to the above mixture, the resulting mixture was stirred at -5 °C for another 1 h. Then a solution of *N*-tosylaziridine (618 mg, 3.14 mmol, 1.1 equiv) in anhydrous THF (2 mL) was added slowly. After stirring at -5 °C for 1 h, the reaction mixture was allowed to stir overnight at room temperature. A 10% aqueous solution of HCl (12 mL) was added at 0 °C and the mixture was stirred at room temperature for 3 h. The resulting mixture was extracted with ethyl acetate (4×10 mL). The combined organic layers were washed with brine (2×15 mL), dried over anhydrous sodium sulfate, filtered and concentrated under vacuum. The residue was quickly purified by flash column chromatography on silica gel (0% to 5% to 100% ethyl acetate-hexane) to give compound **4xa-2** as a brown oil.

Potassium *tert*-butoxide (959 mg, 8.55 mmol, 3.0 equiv) was added to Ph_3PCH_3Br (3.16 g, 8.84 mmol, 3.1 equiv) in anhydrous diethyl ether (15 mL, *c* = 0.20 mol/L) at room temperature. The reaction mixture was stirred at room temperature for 2 h. Then the resulting solution was transferred to a round-bottom flask charged with the above crude **4xa-2** (2.85 mmol, 1.0 equiv) at room temperature. The reaction mixture was stirred overnight at room temperature. TLC showed that the starting material was consumed completely, then the reaction mixture was quenched by the addition of saturated ammonium chloride. The resulting mixture was extracted with ethyl acetate (4×10 mL). The combined organic layers were washed with brine (2×15 mL), dried over anhydrous sodium sulfate, filtered and concentrated under vacuum. The residue was purified by flash column chromatography on silica gel (0% to 5% to 10% ethyl acetate-hexane) to give compound **4xa** as an inseparable colorless oil (dr = 1:1, 387 mg, 49% over 2 steps).

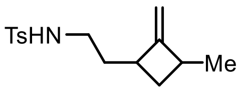

dr = 1:1

4xa

4-Methyl-N-(2-(3-methyl-2-methylenecyclobutyl)ethyl)benzenesulfonamide (4xa): ^{1}H NMR (600 MHz, $CDCl_3$) δ 7.75 (d, *J* = 8.3 Hz, 2H), 7.31 (d, *J* = 8.3 Hz, 2H), 4.70 (t, *J* = 2.3 Hz, 0.5H), 4.65 (t, *J* = 2.6 Hz, 0.5H), 4.62 (t, *J* = 2.3 Hz, 0.5H), 4.60 (t, *J* = 2.6 Hz, 0.5H), 4.55 (t, *J* = 6.1 Hz, 0.5H), 4.50 (t, *J* = 6.2 Hz, 0.5H), 3.02 – 2.87 (m, 2.5H), 2.87 – 2.72 (m, 1H), 2.43 (s, 3H), 2.21 (dtd, *J* = 10.0, 8.7, 1.1 Hz, 0.5H), 1.83 – 1.74 (m, 0.5H), 1.74 – 1.67 (m, 1H), 1.67 – 1.59 (m, 1.5H), 1.52 – 1.44 (m, 0.5H), 1.09 (d, *J* = 7.0 Hz, 1.5H), 1.09 – 1.05 (m, 0.5H), 1.04 (d, *J* = 6.8 Hz, 1.5H) ppm; **^{13}C NMR** (100 MHz, $CDCl_3$) δ 159.8, 158.8, 143.5 (two isomers), 137.1 (two isomers), 129.8 (two isomers, 2C), 127.2 (two isomers, 2C), 103.9, 100.8, 41.6, 41.4, 39.4, 39.0, 36.9, 36.6, 34.4, 33.9, 33.0, 31.3, 21.6 (two isomers), 19.6, 18.6 ppm; **IR** (ν_{max}, cm^{-1}) 3283 (w), 2955 (w), 2865 (w), 1422 (w), 1322 (m), 1155 (s), 1092 (m), 873 (m), 814 (m), 662 (s); **HRMS** (ESI/QTOF) m/z: $[M + H]^+$ Calcd for $C_{15}H_{22}NO_2S^+$ 280.1366; Found 280.1369.

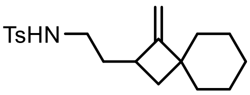

4xb

4-Methyl-N-(2-(1-methylenespiro[3.5]nonan-2-yl)ethyl)benzenesulfonamide (4xb): Following the general procedure A, compound **4xb** was isolated after purification by flash column chromatography on silica gel as a white solid. **^{1}H NMR** (400 MHz, $CDCl_3$) δ 7.75 (d, *J* = 8.3 Hz, 2H), 7.30 (d, *J* = 8.2 Hz, 2H), 4.75 (d, *J* = 2.8 Hz, 1H), 4.72 (brs, 1H), 4.60 (d, *J* = 2.3 Hz, 1H), 3.01 – 2.86 (m, 2H), 2.83 – 2.72 (m, 1H), 2.42 (s, 3H), 1.88 (dd, *J* = 10.9, 9.2 Hz, 1H), 1.81 – 1.70 (m, 1H), 1.58 – 1.16 (m, 12H) ppm; **^{13}C NMR** (100 MHz, $CDCl_3$) δ 163.3, 143.4, 137.1, 129.8 (2C), 127.2 (2C), 102.1, 46.4, 41.4, 37.4, 36.9, 36.1, 36.1, 34.6, 25.9, 22.9, 22.7, 21.6 ppm; **IR** (ν_{max}, cm^{-1}) 3280 (w), 2926 (m), 2848 (w), 1444 (w), 1323 (m), 1155 (s), 1092 (m), 874 (m), 814 (m), 661 (s); **HRMS** (ESI/QTOF) m/z: $[M + Na]^+$ Calcd for $C_{19}H_{27}NNaO_2S^+$ 356.1655; Found 356.1656.

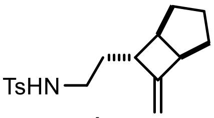

4y

4-Methyl-*N*-(2-(7-methylenebicyclo[3.2.0]heptan-6-yl)ethyl)benzenesulfonamide (4y): Following the general procedure A, compound **4y** was isolated after purification by flash column chromatography on silica gel as a colorless oil. **^{1}H NMR** (400 MHz, $CDCl_3$) δ 7.75 (d, *J* = 8.4 Hz, 2H), 7.31 (d, *J* = 8.0 Hz, 2H), 4.70 (t, *J* = 2.2 Hz, 1H), 4.64 (t, *J* = 2.2 Hz, 1H), 4.49 (t, *J* = 6.1 Hz, 1H), 3.20 – 3.12 (m, 1H), 3.05 – 2.91 (m, 2H), 2.43 (s, 3H), 2.28 – 2.20 (m, 2H), 1.75 – 1.58 (m, 5H), 1.56 – 1.36 (m, 3H) ppm; **^{13}C NMR** (100 MHz, $CDCl_3$) δ 156.9, 143.5, 137.1, 129.8 (2C), 127.2 (2C), 106.1, 45.6, 44.8, 41.4, 41.3, 34.7, 33.1, 32.7, 25.1, 21.7 ppm; **IR** (ν_{max}, cm^{-1}) 3286 (m), 2942 (m), 2857 (m), 1658 (m), 1598 (m), 1429 (m), 1329 (s), 1153 (s), 1093 (s), 1065 (m), 876 (m), 813 (s), 660 (s); **HRMS** (ESI/QTOF) m/z: $[M + Na]^+$ Calcd for $C_{17}H_{23}NNaO_2S^+$ 328.1342; Found 328.1342.

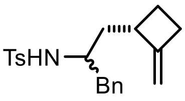

4z

dr = 1.1/1

4-Methyl-*N*-(1-(2-methylenecyclobutyl)-3-phenylpropan-2-yl)benzenesulfonamide (4z): Following the general procedure B (from substituted *N*-tosylaziridine **S8-5**), compound **4z** was isolated after purification by flash column chromatography on silica gel as an inseparable colorless oil (dr = 1.1:1). **^{1}H NMR** (400 MHz, $CDCl_3$) δ 7.69 (d, J = 8.0 Hz, 1H), 7.65 (d, J = 8.2 Hz, 1H), 7.28 – 7.16 (m, 5H), 7.04 – 6.97 (m, 2H), 4.65 – 4.61 (m, 1H), 4.61 – 4.57 (m, 1H), 4.55 – 4.32 (m, 1H), 3.57 – 3.37 (m, 1H), 2.97 – 2.85 (m, 1H), 2.74 – 2.59 (m, 2H), 2.59 – 2.40 (m, 2H), 2.42 (s, 1.5H), 2.41 (s, 1.5H), 2.05 (dtd, J = 10.6, 9.1, 4.7 Hz, 0.5H), 1.82-1.72 (m, 1H), 1.67 – 1.58 (m, 1H), 1.57 – 1.45 (m, 1H), 1.37 – 1.28 (m, 0.5H) ppm; **^{13}C NMR** (100 MHz, $CDCl_3$) δ 154.0 (major + minor), 143.3 (major), 143.2 (minor), 137.99 (minor), 137.97(major), 137.2 (minor), 137.1 (major), 129.7 (major + minor, 2C), 129.64 (major, 2C), 129.62 (minor, 2C), 128.6 (major + minor, 2C), 127.2 (major, 2C), 127.1 (minor, 2C), 126.7 (major + minor), 103.8 (minor), 103.5 (major), 53.9 (minor), 53.1 (major), 42.0 (major), 41.4 (minor), 41.3 (minor), 41.0 (major), 39.3 (minor), 39.1 (major), 29.6 (minor), 29.2 (major), 24.6 (minor), 24.2 (major), 21.6 (major + minor) ppm; **IR** (ν_{max}, cm^{-1}) 3269 (w), 2918 (w), 1670 (w), 1606 (w), 1496 (w), 1421 (m), 1323 (m), 1153 (s), 1084 (m), 874 (m), 814 (m), 739 (m), 700 (s), 660 (s); **HRMS** (ESI/QTOF) m/z: [M + Na]$^+$ Calcd for $C_{21}H_{25}NNaO_2S^+$ 378.1498; Found 378.1496.

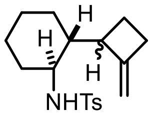

4aa
dr = 2.4/1

4-Methyl-*N*-(2-(2-methylenecyclobutyl)cyclohexyl)benzenesulfonamide (4aa): Following the general procedure B (from substituted *N*-tosylaziridine **S8-4**), compound **4aa** was isolated after purification by flash column chromatography on silica gel as an inseparable white solid (dr = 2.4:1). m.p. = 76 ~ 78 ^{o}C; **^{1}H NMR** (400 MHz, $CDCl_3$) δ 7.84 (d, J = 8.3 Hz, 2H, minor), 7.83 (d, J = 8.3 Hz, 2H, major), 7.33 (d, J = 8.2 Hz, 2H, major + 2H, minor), 5.32 (d, J = 8.3 Hz, 1H, minor), 5.21 (d, J = 9.0 Hz, 1H, major), 4.79 – 4.72 (m, 1H, major + 2H minor), 4.62 (q, J = 2.6 Hz, 1H, major), 3.49 – 3.39 (m, 1H, major), 3.32 – 3.22 (m, 1H, minor), 3.13 (qd, J = 8.8, 3.9 Hz, 1H, minor), 2.92 (qd, J = 9.7, 4.2 Hz, 1H, major), 2.58 – 2.44 (m, 1H, major + 1H, minor), 2.46 (s, 3H, major + 3H, minor), 2.43 – 2.32 (m, 1H, major), 2.10 – 1.99 (m, 1H, minor), 1.96 – 1.82 (m, 1H, major + 1H, minor), 1.80 – 1.51 (m, 5H, major + 5H, minor), 1.50 – 1.37 (m, 1H, major + 1H, minor), 1.37– 1.02 (m, 4H, major + 4H, minor) ppm; **^{13}C NMR**

(100 MHz, $CDCl_3$) δ 152.5 (major), 152.3 (minor), 143.0 (major + minor), 138.7 (minor), 138.6 (major), 129.59 (minor), 129.57 (major), 127.0 (major), 126.9 (minor), 106.1 (minor), 103.8 (major), 55.3 (minor), 54.3 (major), 45.2 (minor), 45.0 (minor), 44.6 (major), 43.6 (major), 34.1 (major), 33.2 (minor), 29.3 (minor), 29.0 (major), 26.5 (minor), 25.2 (major), 25.1 (major), 24.7 (major), 24.3 (minor), 24.2 (minor), 21.5 (major + minor), 21.3 (minor), 18.0 (major) ppm; **IR** (ν_{max}, cm^{-1}) 3273 (w), 2931 (m), 2862 (w), 1672 (w), 1599 (w), 1431 (w), 1323 (m), 1153 (s), 1063 (m), 910 (m), 812 (m), 731 (m), 663 (s); **HRMS** (ESI/QTOF) m/z: [M + Na]$^+$ Calcd for $C_{18}H_{25}NNaO_2S^+$ 342.1498; Found 342.1502.

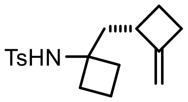

4ab

4-Methyl-*N*-(1-((2-methylenecyclobutyl)methyl)cyclobutyl)benzenesulfonamide (4ab): Following the general procedure B (from substituted *N*-tosylaziridine **S8-1**), compound **4ab** was isolated after purification by flash column chromatography on silica gel as a white solid. m.p. = 96 ~ 98 °C; **^{1}H NMR** (400 MHz, $CDCl_3$) δ 7.80 – 7.76 (m, 2H), 7.30 – 7.25 (m, 2H), 5.08 (s, 1H), 4.64 (q, *J* = 2.5 Hz, 1H), 4.58 (q, *J* = 2.5 Hz, 1H), 3.03 – 2.92 (m, 1H), 2.66 – 2.55 (m, 1H), 2.54 – 2.45 (m, 1H), 2.41 (s, 3H), 2.25 – 2.15 (m, 1H), 2.14 – 2.03 (m, 2H), 2.03 (dd, *J* = 14.3, 3.5 Hz, 1H), 1.93 – 1.78 (m, 3H), 1.77 – 1.59 (m, 3H) ppm; **^{13}C NMR** (100 MHz, $CDCl_3$) δ 154.6, 143.1, 140.4, 129.7 (2C), 126.9 (2C), 102.8, 59.9, 42.7, 40.7, 33.5, 33.1, 30.5, 26.5, 21.6, 15.0 ppm; **IR** (ν_{max}, cm^{-1}) 3263 (w), 2941 (w), 1672 (w), 1599 (w), 1425 (w), 1317 (m), 1146 (s), 1093 (s), 978 (m), 876 (m), 816 (m), 661 (s); **HRMS** (ESI/QTOF) m/z: [M + Na]$^+$ Calcd for $C_{17}H_{23}NNaO_2S^+$ 328.1342; Found 328.1337.

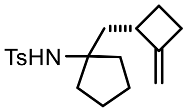

4ac

4-Methyl-*N*-(1-((2-methylenecyclobutyl)methyl)cyclopentyl)benzenesulfonamide (4ac): Following the general procedure B (from substituted *N*-tosylaziridine **S8-2**), compound **4ac** was isolated after purification by flash column chromatography on silica gel as a white solid. **^{1}H NMR** (400 MHz, $CDCl_3$) δ 7.79 (d, *J* = 8.3 Hz, 2H), 7.26 (d, *J* = 8.1 Hz, 2H), 5.01 (s, 1H),

4.63 (q, *J* = 2.4 Hz, 1H), 4.56 (q, *J* = 2.5 Hz, 1H), 3.12 – 3.00 (m, 1H), 2.65 – 2.53 (m, 1H), 2.52 – 2.42 (m, 1H), 2.40 (s, 3H), 2.15 – 2.02 (m, 2H), 1.98 – 1.87 (m, 1H), 1.75 – 1.59 (m, 3H), 1.55 – 1.30 (m, 6H) ppm; **^{13}C NMR** (100 MHz, $CDCl_3$) δ 154.6, 142.8, 140.6, 129.6 (2C), 126.8 (2C), 102.6, 68.3, 43.3, 41.3, 38.2, 37.9, 30.2, 26.7, 22.8, 22.5, 21.6 ppm; **IR** (ν_{max}, cm^{-1}) 3277 (w), 2953 (w), 2920 (w), 1422 (w), 1323 (m), 1151 (s), 1094 (m), 814 (m), 665 (s), 550 (m); **HRMS** (nanochip-ESI/LTQ-Orbitrap) m/z: $[M + H]^+$ Calcd for $C_{18}H_{26}NO_2S^+$ 320.1679; Found 320.1689.

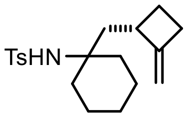

4ad

4-Methyl-*N*-(1-((2-methylenecyclobutyl)methyl)cyclohexyl)benzenesulfonamide (4ad): Following the general procedure B (from substituted *N*-tosylaziridine **S8-3**), compound **4ad** was isolated after purification by flash column chromatography on silica gel as a white solid. m.p. = 125 ~ 127 ^oC; **^{1}H NMR** (400 MHz, $CDCl_3$) δ 7.81 – 7.76 (m, 2H), 7.28 – 7.23 (m, 2H), 4.75 (s, 1H), 4.60 (q, *J* = 2.3 Hz, 1H), 4.50 (q, *J* = 2.5 Hz, 1H), 3.05 – 2.94 (m, 1H), 2.58 (dtt, *J* = 14.8, 9.1, 2.8 Hz, 1H), 2.50 – 2.38 (m, 1H), 2.39 (s, 3H), 2.05 (dtd, *J* = 10.5, 9.1, 3.6 Hz, 1H), 1.97 (dd, *J* = 14.6, 2.9 Hz, 1H), 1.86 – 1.74 (m, 1H), 1.71 – 1.55 (m, 3H), 1.42 – 1.26 (m, 7H), 1.25 – 1.14 (m, 1H) ppm; **^{13}C NMR** (100 MHz, $CDCl_3$) δ 154.6, 142.8, 140.9, 129.6 (2C), 127.0 (2C), 102.6, 60.3, 43.6, 40.2, 36.28, 36.25, 30.4, 27.3, 25.4, 21.7 (2C), 21.6 ppm; **IR** (ν_{max}, cm^{-1}) 3284 (m), 2941 (m), 2860 (m), 1674 (m), 1597 (w), 1452 (m), 1327 (m), 1147 (s), 1093 (s), 989 (s), 877 (m), 810 (m), 661 (s); **HRMS** (ESI/QTOF) m/z: $[M + Na]^+$ Calcd for $C_{19}H_{27}NNaO_2S^+$ 356.1655; Found 356.1654.

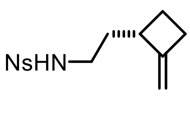

4ae

***N*-(2-(2-Methylenecyclobutyl)ethyl)-4-nitrobenzenesulfonamide (4ae)**: Following the general procedure C, compound **4ae** was isolated after purification by flash column chromatography on silica gel as a white solid. **^{1}H NMR** (400 MHz, $CDCl_3$) δ 8.36 (d, *J* = 8.8 Hz, 2H), 8.06 (d, *J* = 8.8 Hz, 2H), 4.94 (t, *J* = 6.1 Hz, 1H), 4.68 (q, *J* = 2.3 Hz, 1H), 4.64 (q, *J* = 2.5 Hz, 1H), 3.11 – 2.95 (m, 2H), 2.95-2.84 (m, 1H), 2.65 – 2.46 (m, 2H), 2.04 (dtd, *J* = 10.7,

9.1, 5.1 Hz, 1H), 1.79 (dq, *J* = 14.0, 6.8 Hz, 1H), 1.61 (dtd, *J* = 13.9, 8.2, 6.5 Hz, 1H), 1.51 (ddt, *J* = 10.8, 9.4, 7.5 Hz, 1H) ppm; **^{13}C NMR** (100 MHz, $CDCl_3$) δ 153.3, 150.2, 146.0, 128.4 (2C), 124.6 (2C), 104.5, 41.5, 41.5, 34.1, 29.3, 23.4 ppm; **IR** (ν_{max}, cm^{-1}) 3293 (w), 2925 (w), 1530 (s), 1349 (s), 1310 (m), 1163 (s), 1093 (m), 855 (m), 736 (m), 611 (m), 463 (w); **HRMS** (ESI/QTOF) m/z: $[M + H]^+$ Calcd for $C_{13}H_{17}N_2O_4S^+$ 297.0904; Found 297.0905.

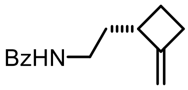

4af

***N*-(2-(2-Methylenecyclobutyl)ethyl)benzamide (4af)**: Following the general procedure C, compound **4af** was isolated after purification by flash column chromatography on silica gel as a colorless oil. **^{1}H NMR** (400 MHz, $CDCl_3$) δ 7.78 – 7.73 (m, 2H), 7.49 – 7.43 (m, 1H), 7.43 – 7.35 (m, 2H), 6.46 (t, *J* = 5.9 Hz, 1H), 4.75 (q, *J* = 2.5 Hz, 1H), 4.72 (q, *J* = 2.3 Hz, 1H), 3.53 – 3.39 (m, 2H), 3.04 – 2.93 (m, 1H), 2.69 – 2.50 (m, 2H), 2.12 (dtd, *J* = 10.7, 9.0, 5.0 Hz, 1H), 1.98 – 1.86 (m, 1H), 1.77 – 1.58 (m, 2H) ppm; **^{13}C NMR** (100 MHz, $CDCl_3$) δ 167.7, 154.1, 134.8, 131.4, 128.6 (2C), 127.0 (2C), 104.0, 42.3, 38.2, 34.0, 29.4, 23.7 ppm; **IR** (ν_{max}, cm^{-1}) 3311 (w), 3068 (w), 2924 (w), 1635 (s), 1540 (s), 1309 (m), 874 (m), 695 (s); **HRMS** (ESI/QTOF) m/z: $[M + Na]^+$ Calcd for $C_{14}H_{17}NNaO^+$ 238.1202; Found 238.1205.

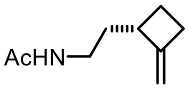

4ag

***N*-(2-(2-Methylenecyclobutyl)ethyl)acetamide (4ag)**: Following the general procedure C, compound **4ag** was isolated after purification by flash column chromatography on silica gel as a pale yellow oil. **^{1}H NMR** (400 MHz, $CDCl_3$) δ 5.93 (s, 1H), 4.69 (q, *J* = 2.5 Hz, 1H), 4.67 (q, *J* = 2.3 Hz, 1H), 3.28-3.15 (m, 2H), 2.96-2.85 (m, 1H), 2.65 – 2.47 (m, 2H), 2.07 (dtd, *J* = 10.8, 9.0, 5.1 Hz, 1H), 1.93 (s, 3H), 1.84 – 1.69 (m, 1H), 1.63 – 1.52 (m, 2H) ppm; **^{13}C NMR** (100 MHz, $CDCl_3$) δ 170.3, 154.0, 103.9, 42.2, 37.6, 33.9, 29.3, 23.7, 23.3 ppm; **IR** (ν_{max}, cm^{-1}) 3293 (m), 2931 (m), 1718 (w), 1651 (s), 1555 (m), 1437 (w), 1369 (w), 1295 (w), 875 (w), 601 (w); **HRMS** (ESI/QTOF) m/z: $[M + H]^+$ Calcd for $C_9H_{16}NO^+$ 154.1226; Found 154.1225.

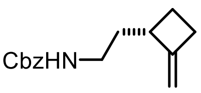

4ah

Benzyl (2-(2-methylenecyclobutyl)ethyl)carbamate (4ah): Following the general procedure C, compound **4ah** was isolated after purification by flash column chromatography on silica gel as a colorless oil. **^{1}H NMR** (400 MHz, $CDCl_3$) δ 7.40 – 7.28 (m, 5H), 5.10 (s, 2H), 4.79 (brs, 1H), 4.74 (q, J = 2.6 Hz, 1H), 4.71 (q, J = 2.4 Hz, 1H), 3.28-3.14 (m, 2H), 3.00 – 2.88 (m, 1H), 2.69 – 2.51 (m, 2H), 2.10 (qd, J = 9.3, 4.8 Hz, 1H), 1.82 (dq, J = 13.8, 6.9 Hz, 1H), 1.66-1.56 (m, 2H) ppm; **^{13}C NMR** (100 MHz, $CDCl_3$) δ 156.5, 154.0, 136.7, 128.6, 128.2, 128.2, 104.0, 66.7, 42.1, 39.2, 34.5, 29.4, 23.7 ppm; **IR** (ν_{max}, cm^{-1}) 3333 (w), 2930 (w), 1694 (s), 1523 (m), 1242 (s), 1135 (m), 872 (m), 734 (m), 695 (s); **HRMS** (ESI/QTOF) m/z: [M + Na]$^+$ Calcd for $C_{15}H_{19}NNaO_2^+$ 268.1308; Found 268.1310.

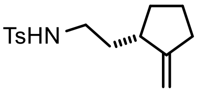

7

4-Methyl-*N*-(2-(2-methylenecyclopentyl)ethyl)benzenesulfonamide (7): Following the general procedure A, compound **7** was isolated after purification by flash column chromatography on silica gel as a white solid. **^{1}H NMR** (400 MHz, $CDCl_3$) δ 7.69 (d, J = 8.3 Hz, 2H), 7.23 (d, J = 7.7 Hz, 2H), 4.88 – 4.63 (m, 1H), 4.76 (s, 1H), 4.59 (s, 1H), 3.00 – 2.82 (m, 2H), 2.35 (s, 3H), 2.28 – 2.09 (m, 3H), 1.79 – 1.52 (m, 3H), 1.48 – 1.36 (m, 1H), 1.36 – 1.25 (m, 1H), 1.14 – 1.02 (m, 1H) ppm; **^{13}C NMR** (100 MHz, $CDCl_3$) δ 155.8, 143.4, 137.1, 129.8 (2C), 127.2 (2C), 104.9, 42.0, 41.3, 34.2, 32.9, 32.5, 24.2, 21.6 ppm; **IR** (ν_{max}, cm^{-1}) 3278 (w), 2948 (w), 1322 (m), 1155 (s), 1093 (m), 814 (m), 661 (m), 507 (m); **HRMS** (nanochip-ESI/LTQ-Orbitrap) m/z: [M + Na]$^+$ Calcd for $C_{15}H_{21}NNaO_2S^+$ 302.1185; Found 302.1195.

IV. Synthesis of 1-fluorinated 2-azabicyclo[3.2.1]octane: substrate scope

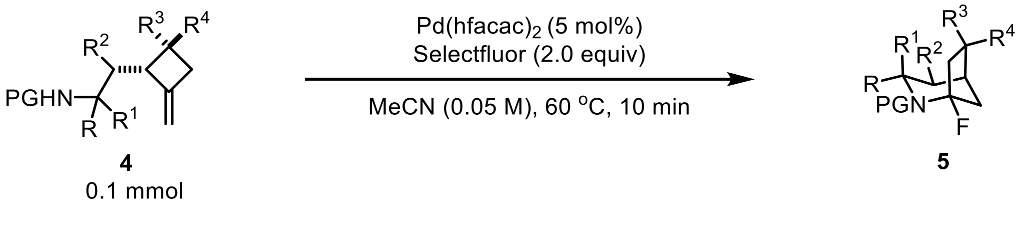

General procedure for the synthesis of 1-fluorinated 2-azabicyclo[3.2.1]octane

To a 10-mL vial charged with substrate **4** (0.10 mmol, 1.0 equiv), Pd(hfacac)$_2$ (2.6 mg, 0.005 mmol, 5 mol%) and Selectfluor (70.8 mg, 0.20 mmol, 2.0 equiv) was added anhydrous acetonitrile (preheated to 60 °C, 2.0 mL, *c* = 0.05 mol/L) under N_2 atmosphere. The sealed vial was allowed to stir at 60 °C for 10 min. The reaction mixture was allowed to cool in the ice bath, diluted with diethyl ether, filtered through a short pad of silica gel and rinsed with diethyl ether. The filtrate was concentrated to dryness at 25 °C and the residue was purified by silica gel flash column chromatography or preparative thin layer chromatography to afford the corresponding fluorine-containing azabicyclo[3.2.1]octane **5**.

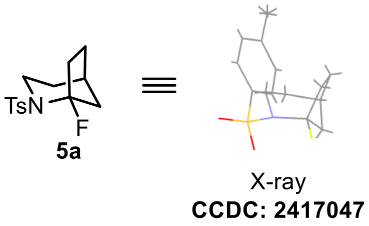

1-Fluoro-2-tosyl-2-azabicyclo[3.2.1]octane (5a): Following the general procedure, compound **5a** was isolated as a white solid (21.5 mg, 76%). m.p. = 102 ~ 104 °C; **^{1}H NMR** (400 MHz, $CDCl_3$) δ 7.75 (d, *J* = 8.0 Hz, 2H), 7.27 (d, *J* = 8.1 Hz, 2H), 4.03 – 3.95 (m, 1H), 3.19 (ddd, *J* = 13.3, 10.8, 4.9 Hz, 1H), 2.48 – 2.40 (m, 1H), 2.41 (s, 3H), 2.08 – 1.95 (m, 2H), 1.93 – 1.80 (m, 3H), 1.80 – 1.69 (m, 1H), 1.53 – 1.45 (m, 1H), 1.45 – 1.37 (m, 1H) ppm; **^{13}C NMR** (100 MHz, $CDCl_3$) δ 143.2, 139.2, 129.5 (2C), 127.5 (d, *J* = 2.1 Hz, 2C), 111.4 (d, *J* = 221.4 Hz), 42.8, 41.9 (d, *J* = 18.3 Hz), 33.4 (d, *J* = 7.3 Hz), 33.3 (d, *J* = 22.7 Hz), 31.3 (d, *J* = 1.1 Hz), 27.1 (d, *J* = 7.3 Hz), 21.7 ppm; **^{19}F NMR** (376 MHz, $CDCl_3$) δ -137.25 ppm; **IR** (ν_{max}, cm^{-1}) 2945 (w), 1458 (w), 1338 (s), 1155 (s), 1086 (m), 993 (m), 957 (m), 814 (m), 715 (m), 687 (s); **HRMS** (ESI/QTOF) m/z: [M + H]$^+$ Calcd for $C_{14}H_{19}FNO_2S^+$ 284.1115; Found 284.1116.

<u>1 mmol scale:</u> same set-up with the loading of 1 mmol% Pd(hfacac)$_2$, work-up and purification – 209.7 mg, 74% yield.

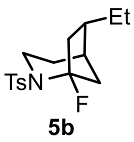

6-Ethyl-1-fluoro-2-tosyl-2-azabicyclo[3.2.1]octane (5b): Following the general procedure, compound **5b** was isolated as a colorless oil (23.0 mg, 74%). **^{1}H NMR** (400 MHz, $CDCl_3$) δ 7.75 (d, J = 8.0 Hz, 2H), 7.27 (d, J = 7.9 Hz, 2H), 3.97 – 3.87 (m, 1H), 3.23 (ddd, J = 13.3, 10.0, 5.2 Hz, 1H), 2.41 (s, 3H), 2.31 (ddt, J = 13.3, 8.3, 2.2 Hz, 1H), 2.12 (dq, J = 6.9, 3.5 Hz, 1H), 2.00 – 1.90 (m, 1H), 1.84 – 1.78 (m, 1H), 1.75 (ddt, J = 10.1, 6.4, 3.5 Hz, 1H), 1.63 – 1.30 (m, 5H), 0.86 (t, J = 7.3 Hz, 3H) ppm; **^{13}C NMR** (100 MHz, $CDCl_3$) δ 143.2, 139.2, 129.5 (2C), 127.5 (d, J = 2.2 Hz, 2C), 111.1 (d, J = 222.2 Hz), 42.83, 42.76 (d, J = 6.4 Hz), 40.9 (d, J = 21.8 Hz), 39.3 (d, J = 18.1 Hz), 38.1 (d, J = 7.0 Hz), 31.6 (d, J = 1.1 Hz), 29.9, 21.7, 12.5 ppm; **^{19}F NMR** (376 MHz, $CDCl_3$) δ -134.54 ppm; **IR** (ν_{max}, cm^{-1}) 2931 (w), 1599 (w), 1458 (w), 1333 (m), 1157 (s), 995 (m), 812 (m), 687 (s); **HRMS** (ESI/QTOF) m/z: $[M + Na]^+$ Calcd for $C_{16}H_{22}FNNaO_2S^+$ 334.1247; Found 334.1254.

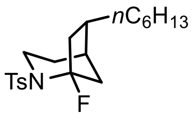

5c

1-Fluoro-6-hexyl-2-tosyl-2-azabicyclo[3.2.1]octane (5c): Following the general procedure, compound **5c** was isolated as a colorless oil (23.2 mg, 63%). **^{1}H NMR** (400 MHz, $CDCl_3$) δ 7.75 (d, J = 8.0 Hz, 2H), 7.27 (d, J = 8.0 Hz, 2H), 3.91 (ddt, J = 13.9, 6.4, 3.9 Hz, 1H), 3.23 (ddd, J = 13.3, 10.0, 5.2 Hz, 1H), 2.41 (s, 3H), 2.30 (ddt, J = 13.4, 8.5, 2.2 Hz, 1H), 2.10 (dq, J = 7.0, 3.6 Hz, 1H), 2.02 – 1.92 (m, 1H), 1.81 (d, J = 11.1 Hz, 1H), 1.74 (dtd, J = 10.2, 6.8, 3.8 Hz, 1H), 1.70 – 1.60 (m, 1H), 1.56 – 1.43 (m, 2H), 1.43 – 1.16 (m, 10H), 0.87 (t, J = 6.8 Hz, 3H) ppm; **^{13}C NMR** (100 MHz, $CDCl_3$) δ 143.2, 139.2, 129.5 (2C), 127.5 (d, J = 2.1 Hz, 2C), 111.1 (d, J = 222.3 Hz), 42.8, 41.2 (d, J = 21.8 Hz), 40.9 (d, J = 6.2 Hz), 39.3 (d, J = 18.0 Hz), 38.4 (d, J = 6.9 Hz), 37.2, 31.9, 31.6 (d, J = 0.7 Hz), 29.3, 27.9, 22.7, 21.7, 14.2 ppm; **^{19}F NMR** (376 MHz, $CDCl_3$) δ -134.46 ppm; **IR** (ν_{max}, cm^{-1}) 2925 (m), 2856 (w), 1601 (w), 1454 (w), 1331 (m), 1155 (s), 1093 (m), 987 (m), 814 (m), 687 (s); **HRMS** (ESI/QTOF) m/z: $[M + Na]^+$ Calcd for $C_{20}H_{30}FNNaO_2S^+$ 390.1873; Found 390.1879.

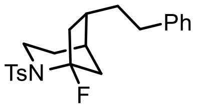

5d

1-Fluoro-6-phenethyl-2-tosyl-2-azabicyclo[3.2.1]octane (5d): Following the general procedure, compound **5d** was isolated as a colorless oil (25.6 mg, 66%). **^{1}H NMR** (400 MHz, $CDCl_3$) δ 7.75 (d, *J* = 8.1 Hz, 2H), 7.33 – 7.24 (m, 4H), 7.22 – 7.10 (m, 3H), 3.91 (ddt, *J* = 13.9, 6.4, 3.9 Hz, 1H), 3.21 (ddd, *J* = 13.1, 9.9, 5.2 Hz, 1H), 2.62 – 2.53 (m, 2H), 2.42 (s, 3H), 2.39 – 2.30 (m, 1H), 2.16 (dq, *J* = 6.8, 3.6 Hz, 1H), 2.05 – 1.94 (m, 1H), 1.85 (dd, *J* = 11.4, 2.4 Hz, 1H), 1.81 – 1.62 (m, 4H), 1.61 – 1.47 (m, 2H) ppm; **^{13}C NMR** (100 MHz, $CDCl_3$) δ 143.2, 141.7, 139.1, 129.5 (2C), 128.6 (2C), 128.5 (2C), 127.5 (d, *J* = 2.1 Hz, 2C), 126.1, 111.0 (d, *J* = 222.2 Hz), 42.8, 41.1 (d, *J* = 22.0 Hz), 40.2 (d, *J* = 6.3 Hz), 39.4 (d, *J* = 18.1 Hz), 38.9, 38.3 (d, *J* = 6.9 Hz), 34.3, 31.4 (d, *J* = 1.4 Hz), 21.7 ppm; **^{19}F NMR** (376 MHz, $CDCl_3$) *δ* -134.43 ppm; **IR** (ν_{max}, cm^{-1}) 2920 (w), 1603 (w), 1496 (w), 1454 (w), 1329 (m), 1157 (s), 982 (m), 812 (m), 685 (s); **HRMS** (ESI/QTOF) m/z: $[M + H]^+$ Calcd for $C_{22}H_{27}FNO_2S^+$ 388.1741; Found 388.1744.

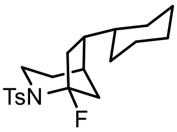

5e

6-Cyclohexyl-1-fluoro-2-tosyl-2-azabicyclo[3.2.1]octane (5e): Following the general procedure, compound **5e** was isolated as a colorless oil (21.2 mg, 58%). **^{1}H NMR** (400 MHz, $CDCl_3$) *δ* 7.75 (d, *J* = 8.1 Hz, 2H), 7.27 (d, *J* = 8.1 Hz, 2H), 3.83 (ddt, *J* = 13.2, 6.4, 4.3 Hz, 1H), 3.30 (dddd, *J* = 13.2, 9.2, 5.6, 1.4 Hz, 1H), 2.41 (s, 3H), 2.33-2.25 (m, 2H), 1.92 – 1.59 (m, 9H), 1.53 – 1.45 (m, 1H), 1.42 (q, *J* = 8.3 Hz, 1H), 1.26 – 1.05 (m, 4H), 0.84 (tdd, *J* = 12.0, 8.5, 3.6 Hz, 2H) ppm; **^{13}C NMR** (100 MHz, $CDCl_3$) *δ* 143.2, 139.2, 129.5 (2C), 127.4 (d, *J* = 2.1 Hz, 2C), 110.8 (d, *J* = 222.6 Hz), 47.4 (d, *J* = 5.8 Hz), 42.5, 42.4, 39.7 (d, *J* = 18.0 Hz), 38.9 (d, *J* = 22.7 Hz), 34.8 (d, *J* = 7.0 Hz), 32.2 (d, *J* = 0.7 Hz), 31.5, 30.7, 26.6, 26.4, 26.3, 21.7 ppm; **^{19}F NMR** (376 MHz, $CDCl_3$) *δ* -133.55 ppm; **IR** (ν_{max}, cm^{-1}) 2924 (m), 2850 (m), 1450 (m), 1333 (s), 1161 (s), 1159 (s), 1088 (m), 995 (m), 937 (m), 814 (m), 688 (s); **HRMS** (ESI/QTOF) m/z: $[M + Na]^+$ Calcd for $C_{20}H_{28}FNNaO_2S^+$ 388.1717; Found 388.1724.

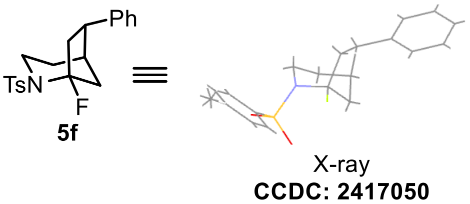

5f

X-ray
CCDC: 2417050

1-Fluoro-6-phenyl-2-tosyl-2-azabicyclo[3.2.1]octane (5f): Following the general procedure, compound **5f** was isolated as a white solid (29.1 mg, 81%). m.p. = 116 ~ 118 °C; **^{1}H NMR** (400 MHz, $CDCl_3$) δ 7.80 (d, J = 8.0 Hz, 2H), 7.35 – 7.27 (m, 4H), 7.24 – 7.17 (m, 3H), 3.94 (ddt, J = 13.3, 6.4, 4.4 Hz, 1H), 3.43 (dddd, J = 13.2, 9.0, 5.7, 1.3 Hz, 1H), 2.99 (dd, J = 9.2, 6.6 Hz, 1H), 2.68 (ddt, J = 13.7, 9.2, 2.1 Hz, 1H), 2.47 – 2.41 (m, 1H), 2.43 (s, 3H), 2.26 (dddd, J = 11.4, 7.7, 5.6, 1.7 Hz, 1H), 2.10 (ddd, J = 14.2, 11.7, 6.6 Hz, 1H), 1.96 (d, J = 11.4 Hz, 1H), 1.91 – 1.81 (m, 1H), 1.81 – 1.71 (m, 1H) ppm; **^{13}C NMR** (100 MHz, $CDCl_3$) δ 145.5, 143.4, 138.8, 129.6 (2C), 128.9 (2C), 127.5 (d, J = 2.1 Hz, 2C), 126.6 (3C), 110.9 (d, J = 223.6 Hz), 46.7 (d, J = 6.4 Hz), 42.6, 42.0 (d, J = 23.6 Hz), 40.7 (d, J = 6.7 Hz), 40.0 (d, J = 18.2 Hz), 32.1, 21.7 ppm; **^{19}F NMR** (376 MHz, $CDCl_3$) δ -134.10 ppm; **IR** (ν_{max}, cm^{-1}) 2939 (w), 1597 (w), 1496 (m), 1452 (m), 1336 (s), 1157 (s), 1084 (m), 1011 (m), 918 (m), 814 (m), 688 (s); **HRMS** (ESI/QTOF) m/z: $[M + Na]^+$ Calcd for $C_{20}H_{22}FNNaO_2S^+$ 382.1247; Found 382.1247.

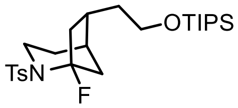

5g

1-Fluoro-2-tosyl-6-(2-((triisopropylsilyl)oxy)ethyl)-2-azabicyclo[3.2.1]octane (5g): Following the general procedure, compound **5g** was isolated as a colorless oil (25.6 mg, 53%). **^{1}H NMR** (400 MHz, $CDCl_3$) δ 7.75 (d, J = 8.1 Hz, 2H), 7.27 (d, J = 8.1 Hz, 2H), 3.95 (ddt, J = 13.7, 6.9, 3.7 Hz, 1H), 3.71-3.60 (m, 2H), 3.20 (ddd, J = 13.3, 10.4, 5.1 Hz, 1H), 2.41 (s, 3H), 2.26 (ddt, J = 13.5, 8.8, 2.1 Hz, 1H), 2.19-2.15 (m, 1H), 2.02-1.94 (m, 1H), 1.93 – 1.80 (m, 2H), 1.80 – 1.62 (m, 2H), 1.61 – 1.48 (m, 3H), 1.15 – 0.96 (m, 21H) ppm; **^{13}C NMR** (100 MHz, $CDCl_3$) δ 143.2, 139.0, 129.5 (2C), 127.5 (d, J = 2.1 Hz, 2C), 111.1 (d, J = 221.8 Hz), 61.7, 43.0, 40.9 (d, J = 21.8 Hz), 40.0, 39.5 (d, J = 18.0 Hz), 38.5 (d, J = 6.9 Hz), 37.2 (d, J = 6.5 Hz), 31.4, 21.7, 18.2 (6C), 12.1 (3C) ppm; **^{19}F NMR** (376 MHz, $CDCl_3$) δ -134.96 ppm; **IR** (ν_{max}, cm^{-1}) 2933 (m), 2870 (m), 1460 (m), 1333 (m), 1157 (s), 1099 (s), 993 (m), 881 (m), 812 (m), 687 (s); **HRMS** (ESI/QTOF) m/z: $[M + Na]^+$ Calcd for $C_{25}H_{42}FNNaO_3SSi^+$ 506.2531; Found 506.2533.

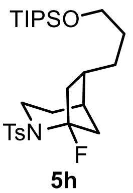

5h

1-Fluoro-2-tosyl-6-(3-((triisopropylsilyl)oxy)propyl)-2-azabicyclo[3.2.1]octane (5h): Following the general procedure, compound **5h** was isolated a white solid (33.8 mg, 68%). m.p. = 66 ~ 68 °C; **^{1}H NMR** (400 MHz, $CDCl_3$) δ 7.75 (d, J = 8.1 Hz, 2H), 7.27 (d, J = 8.1 Hz, 2H), 3.92 (ddt, J = 13.8, 6.4, 3.8 Hz, 1H), 3.65 (t, J = 6.0 Hz, 2H), 3.22 (ddd, J = 13.2, 10.1, 5.2 Hz, 1H), 2.41 (s, 3H), 2.30 (ddt, J = 13.5, 8.5, 2.2 Hz, 1H), 2.14-2.09 (m, 1H), 2.01-1.94 (m, 1H), 1.82 (d, J = 12.6 Hz, 1H), 1.80 – 1.64 (m, 2H), 1.58 – 1.44 (m, 5H), 1.44 – 1.33 (m, 1H), 1.14 – 0.96 (m, 21H) ppm; **^{13}C NMR** (100 MHz, $CDCl_3$) δ 143.2, 139.1, 129.5 (2C), 127.5 (d, J = 2.1 Hz, 2C), 111.1 (d, J = 222.2 Hz), 63.2, 42.9, 41.2 (d, J = 21.9 Hz), 40.7 (d, J = 6.4 Hz), 39.3 (d, J = 18.2 Hz), 38.5 (d, J = 7.0 Hz), 33.4, 31.5, 31.4, 21.7, 18.2 (6C), 12.1 (3C) ppm; **^{19}F NMR** (376 MHz, $CDCl_3$) δ -134.57 ppm; **IR** (ν_{max}, cm^{-1}) 2933 (m), 2862 (m), 1460 (m), 1333 (m), 1159 (s), 1093 (s), 993 (m), 881 (m), 812 (m), 685 (s); **HRMS** (ESI/QTOF) m/z: $[M + H]^+$ Calcd for $C_{26}H_{45}FNO_3SSi^+$ 498.2868; Found 498.2869.

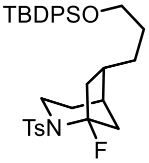

5i

6-(3-((*tert*-Butyldiphenylsilyl)oxy)propyl)-1-fluoro-2-tosyl-2-azabicyclo[3.2.1]octane (5i): Following the general procedure, compound **5i** was isolated as a colorless oil (47.6 mg, 82%). **^{1}H NMR** (400 MHz, $CDCl_3$) δ 7.75 (d, J = 8.1 Hz, 2H), 7.68 – 7.61 (m, 4H), 7.47 – 7.34 (m, 6H), 7.27 (d, J = 8.0 Hz, 2H), 3.91 (ddt, J = 13.8, 6.4, 3.6 Hz, 1H), 3.63 (t, J = 5.9 Hz, 2H), 3.19 (ddd, J = 13.1, 10.1, 5.1 Hz, 1H), 2.42 (s, 3H), 2.27 (ddt, J = 13.2, 8.5, 2.1 Hz, 1H), 2.10-2.04 (m, 1H), 1.99-1.91 (m, 1H), 1.81 (d, J = 11.3 Hz, 1H), 1.74 (dddd, J = 13.6, 10.1, 6.4, 3.7 Hz, 1H), 1.64 (p, J = 7.6 Hz, 1H), 1.55 – 1.32 (m, 6H), 1.04 (s, 9H) ppm; **^{13}C NMR** (100 MHz, $CDCl_3$) δ 143.2, 139.1, 135.7 (4C), 134.1, 134.0, 129.7 (2C), 129.5 (2C), 127.8 (4C), 127.5 (d, J = 2.1 Hz, 2C), 111.1 (d, J = 222.1 Hz), 63.7, 42.8, 41.2 (d, J = 21.9 Hz), 40.5 (d, J = 6.2 Hz), 39.3 (d, J = 18.0 Hz), 38.4 (d, J = 7.0 Hz), 33.3, 31.5, 30.9, 27.0 (3C), 21.7, 19.3 ppm; **^{19}F NMR** (376 MHz, $CDCl_3$) δ -134.59 ppm; **IR** (ν_{max}, cm^{-1}) 2937 (m), 2858 (m), 1423 (w), 1329

(m), 1090 (s), 958 (m), 822 (m), 717 (s), 696 (s); **HRMS** (ESI/QTOF) m/z: $[M + Na]^+$ Calcd for $C_{33}H_{42}FNNaO_3SSi^+$ 602.2531; Found 602.2534.

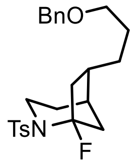

5j

6-(3-(Benzyloxy)propyl)-1-fluoro-2-tosyl-2-azabicyclo[3.2.1]octane (5j): Following the general procedure, compound **5j** was isolated as a colorless oil (31.5 mg, 73%). **^{1}H NMR** (400 MHz, $CDCl_3$) δ 7.75 (d, J = 8.0 Hz, 2H), 7.38 – 7.24 (m, 7H), 4.48 (s, 2H), 3.96 – 3.86 (m, 1H), 3.44 (t, J = 6.2 Hz, 2H), 3.21 (ddd, J = 13.1, 10.0, 5.1 Hz, 1H), 2.41 (s, 3H), 2.29 (ddt, J = 13.4, 8.5, 2.2 Hz, 1H), 2.13-2.08 (m, 1H), 1.97 (dddd, J = 11.1, 7.5, 5.5, 1.6 Hz, 1H), 1.81 (d, J = 11.3 Hz, 1H), 1.78 – 1.62 (m, 2H), 1.61 – 1.36 (m, 6H) ppm; **^{13}C NMR** (100 MHz, $CDCl_3$) δ 143.2, 139.1, 138.6, 129.5 (2C), 128.5 (2C), 127.8 (2C), 127.7, 127.5 (d, J = 2.1 Hz, 2C), 111.0 (d, J = 222.3 Hz), 73.1, 70.1, 42.8, 41.0 (d, J = 22.1 Hz), 40.7 (d, J = 6.4 Hz), 39.3 (d, J = 18.1 Hz), 38.3 (d, J = 7.0 Hz), 33.7, 31.5, 28.3, 21.7 ppm; **^{19}F NMR** (376 MHz, $CDCl_3$) δ -134.52 ppm; **IR** (ν_{max}, cm^{-1}) 2935 (w), 2862 (w), 1599 (w), 1456 (m), 1329 (m), 1163 (s), 1090 (s), 1003 (m), 814 (m), 694 (s); **HRMS** (ESI/QTOF) m/z: $[M + Na]^+$ Calcd for $C_{24}H_{30}FNNaO_3S^+$ 454.1823; Found 454.1828.

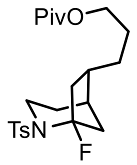

5k

3-(1-Fluoro-2-tosyl-2-azabicyclo[3.2.1]octan-6-yl)propyl pivalate (5k): Following the general procedure, compound **5k** was isolated as a colorless oil (32.8 mg, 77%). **^{1}H NMR** (400 MHz, $CDCl_3$) δ 7.74 (d, J = 8.2 Hz, 2H), 7.27 (d, J = 8.1 Hz, 2H), 4.02 (t, J = 6.4 Hz, 2H), 3.91 (ddt, J = 13.9, 6.5, 4.0 Hz, 1H), 3.23 (ddd, J = 14.3, 9.9, 5.3 Hz, 1H), 2.41 (s, 3H), 2.35 (ddt, J = 13.3, 8.6, 2.1 Hz, 1H), 2.11 (p, J = 3.7 Hz, 1H), 1.96 (dtd, J = 9.8, 6.4, 5.4, 1.6 Hz, 1H), 1.83 (d, J = 12.1 Hz, 1H), 1.77 (dtd, J = 9.7, 6.3, 2.6 Hz, 1H), 1.69 (p, J = 7.6 Hz, 1H), 1.63 – 1.32 (m, 6H), 1.18 (s, 9H) ppm; **^{13}C NMR** (100 MHz, $CDCl_3$) δ 178.7, 143.3, 139.1, 129.5 (2C), 127.4 (d, J = 2.0 Hz, 2C), 110.9 (d, J = 222.4 Hz), 64.1, 42.7, 41.1 (d, J = 22.2 Hz), 40.6 (d, J

= 6.3 Hz), 39.2 (d, J = 18.1 Hz), 38.9, 38.3 (d, J = 7.0 Hz), 33.4, 31.5, 27.3 (3C), 27.2, 21.7 ppm; **^{19}F NMR** (376 MHz, $CDCl_3$) δ -134.42 ppm; **IR** (ν_{max}, cm^{-1}) 2933 (m), 1724 (m), 1464 (w), 1334 (m), 1281 (m), 1155 (s), 814 (m), 688 (s); **HRMS** (ESI/QTOF) m/z: $[M + Na]^+$ Calcd for $C_{22}H_{32}FNNaO_4S^+$ 448.1928; Found 448.1933.

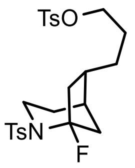

5l

3-(1-Fluoro-2-tosyl-2-azabicyclo[3.2.1]octan-6-yl)propyl 4-methylbenzenesulfonate (5l): Following the general procedure, compound **5l** was isolated as a colorless oil (39.7 mg, 80%). **^{1}H NMR** (400 MHz, $CDCl_3$) δ 7.77 (d, J = 8.3 Hz, 2H), 7.73 (d, J = 8.1 Hz, 2H), 7.34 (d, J = 8.1 Hz, 2H), 7.27 (d, J = 7.9 Hz, 2H), 4.00 (t, J = 6.2 Hz, 2H), 3.86 (ddt, J = 13.3, 6.4, 4.0 Hz, 1H), 3.19 (ddd, J = 14.5, 9.8, 5.4 Hz, 1H), 2.45 (s, 3H), 2.41 (s, 3H), 2.29 (ddt, J = 13.1, 8.4, 2.1 Hz, 1H), 2.06-2.01 (m, 1H), 1.96 – 1.86 (m, 1H), 1.80 (d, J = 12.3 Hz, 1H), 1.73 (dtt, J = 10.1, 6.4, 3.2 Hz, 1H), 1.66 – 1.54 (m, 3H), 1.53 – 1.24 (m, 4H) ppm; **^{13}C NMR** (100 MHz, $CDCl_3$) δ 145.0, 143.3, 139.0, 133.2, 130.0 (2C), 129.5 (2C), 128.0 (2C), 127.4 (d, J = 2.1 Hz, 2C), 110.7 (d, J = 222.5 Hz), 70.3, 42.6, 40.9 (d, J = 22.5 Hz), 40.4 (d, J = 6.4 Hz), 39.1 (d, J = 18.2 Hz), 38.1 (d, J = 6.9 Hz), 32.8, 31.5, 27.4, 21.8, 21.7 ppm; **^{19}F NMR** (376 MHz, $CDCl_3$) δ -134.34 ppm; **IR** (ν_{max}, cm^{-1}) 2933 (w), 1599 (w), 1446 (w), 1348 (s), 1173 (s), 1093 (m), 933 (s), 814 (s), 737 (s), 687 (s); **HRMS** (ESI/QTOF) m/z: $[M + Na]^+$ Calcd for $C_{24}H_{30}FNNaO_5S_2^+$ 518.1442; Found 518.1454.

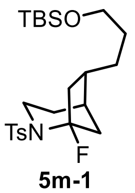

5m-1

6-(3-((*tert*-Butyldimethylsilyl)oxy)propyl)-1-fluoro-2-tosyl-2-azabicyclo[3.2.1]octane (5m-1): Following the general procedure, compound **5m-1** was isolated as a white solid (19.6 mg, 43%). m.p. = 82 ~ 84 ^oC; **^{1}H NMR** (400 MHz, $CDCl_3$) δ 7.75 (d, J = 8.0 Hz, 2H), 7.27 (d, J = 8.1 Hz, 2H), 3.92 (ddt, J = 13.9, 6.5, 3.9 Hz, 1H), 3.57 (t, J = 6.0 Hz, 2H), 3.22 (ddd, J = 13.5, 10.0, 5.2 Hz, 1H), 2.42 (s, 3H), 2.30 (ddt, J = 13.4, 8.5, 2.2 Hz, 1H), 2.14 – 2.08 (m, 1H),

2.02 – 1.92 (m, 1H), 1.82 (d, *J* = 13.0 Hz, 1H), 1.76 (dtd, *J* = 9.8, 6.4, 2.7 Hz, 1H), 1.72 – 1.62 (m, 1H), 1.57 – 1.31 (m, 6H), 0.88 (s, 9H), 0.03 (s, 6H) ppm; ^{13}**C NMR** (100 MHz, $CDCl_3$) δ 143.2, 139.1, 129.5 (2C), 127.5 (d, *J* = 2.1 Hz, 2C), 111.1 (d, *J* = 222.3 Hz), 63.0, 42.8, 41.2 (d, *J* = 21.9 Hz), 40.7 (d, *J* = 6.3 Hz), 39.3 (d, *J* = 18.1 Hz), 38.4 (d, *J* = 7.0 Hz), 33.4, 31.6, 31.3, 26.1 (3C), 21.7, 18.5, -5.1 (2C) ppm; ^{19}**F NMR** (376 MHz, $CDCl_3$) δ -134.53 ppm; **IR** (ν_{max}, cm^{-1}) 2925 (m), 2850 (w), 1460 (w), 1339 (m), 1255 (m), 1158 (s), 1092 (s), 836 (s), 811 (s), 778 (s), 687 (s); **HRMS** (ESI/QTOF) m/z: $[M + Na]^+$ Calcd for $C_{23}H_{38}FNNaO_3SSi^+$ 478.2218; Found 478.2225.

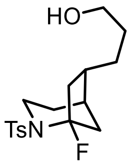

5m-2

3-(1-Fluoro-2-tosyl-2-azabicyclo[3.2.1]octan-6-yl)propan-1-ol (5m-2): Following the general procedure, compound **5m-2** was isolated as a colorless oil (8.2 mg, 24%). ^{1}**H NMR** (400 MHz, $CDCl_3$) δ 7.75 (d, *J* = 8.0 Hz, 2H), 7.27 (d, *J* = 8.0 Hz, 2H), 3.91 (ddt, *J* = 13.9, 7.0, 3.9 Hz, 1H), 3.66-3.60 (m, 2H), 3.28 – 3.19 (m, 1H), 2.42 (s, 3H), 2.38 – 2.29 (m, 1H), 2.15 – 2.09 (m, 1H), 2.03 – 1.94 (m, 1H), 1.83 (d, *J* = 11.7 Hz, 1H), 1.81 – 1.65 (m, 2H), 1.58 – 1.35 (m, 6H) ppm; ^{13}**C NMR** (200 MHz, $CDCl_3$) δ 143.3, 139.1, 129.5 (2C), 127.5 (d, *J* = 1.8 Hz, 2C), 111.0 (d, *J* = 222.4 Hz), 62.8, 42.8, 41.1 (d, *J* = 22.2 Hz), 40.8 (d, *J* = 6.5 Hz), 39.3 (d, *J* = 17.9 Hz), 38.4 (d, *J* = 7.2 Hz), 33.3, 31.6, 31.1, 21.7 ppm; ^{19}**F NMR** (376 MHz, $CDCl_3$) δ -134.46 ppm; **IR** (ν_{max}, cm^{-1}) 2929 (m), 2870 (m), 1333 (m), 1132 (m), 818 (m), 721 (s), 688 (m); **HRMS** (ESI/QTOF) m/z: $[M + Na]^+$ Calcd for $C_{17}H_{24}FNNaO_3S^+$ 364.1353; Found 364.1421.

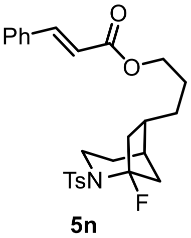

5n

3-(1-Fluoro-2-tosyl-2-azabicyclo[3.2.1]octan-6-yl)propyl cinnamate (5n): Following the general procedure, compound **5n** was isolated as a colorless oil (34.9 mg, 74%). ^{1}**H NMR** (400

MHz, $CDCl_3$) δ 7.75 (d, J = 8.1 Hz, 2H), 7.68 (d, J = 16.0 Hz, 1H), 7.57 – 7.47 (m, 2H), 7.44 – 7.34 (m, 3H), 7.27 (d, J = 8.1 Hz, 2H), 6.43 (d, J = 16.0 Hz, 1H), 4.18 (t, J = 6.5 Hz, 2H), 3.91 (ddt, J = 14.1, 6.5, 3.9 Hz, 1H), 3.24 (ddd, J = 13.4, 9.9, 5.2 Hz, 1H), 2.41 (s, 3H), 2.36 (ddt, J = 13.4, 8.6, 2.1 Hz, 1H), 2.16-2.12 (m, 1H), 2.02-1.96 (m, 1H), 1.85 (d, J = 11.6 Hz, 1H), 1.82 – 1.62 (m, 4H), 1.61 – 1.39 (m, 4H) ppm; **^{13}C NMR** (100 MHz, $CDCl_3$) δ 167.1, 145.0, 143.3, 139.0, 134.5, 130.5, 129.5 (2C), 129.0 (2C), 128.2 (2C), 127.5 (d, J = 2.0 Hz, 2C), 118.1, 110.9 (d, J = 222.4 Hz), 64.4, 42.7, 41.1 (d, J = 22.3 Hz), 40.7 (d, J = 6.3 Hz), 39.2 (d, J = 18.2 Hz), 38.3 (d, J = 6.9 Hz), 33.4, 31.5, 27.3, 21.7 ppm; **^{19}F NMR** (376 MHz, $CDCl_3$) δ -134.40 ppm; **IR** (ν_{max}, cm^{-1}) 2918 (w), 1709 (s), 1635 (m), 1452 (w), 1311 (m), 1163 (s), 984 (m), 816 (m), 769 (m), 688 (s); **HRMS** (ESI/QTOF) m/z: [M + Na]$^+$ Calcd for $C_{26}H_{30}FNNaO_4S^+$ 494.1772; Found 494.1778.

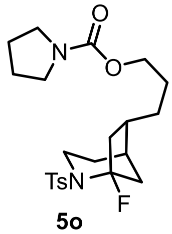

5o

3-(1-Fluoro-2-tosyl-2-azabicyclo[3.2.1]octan-6-yl)propyl pyrrolidine-1-carboxylate (5o): Following the general procedure, compound **5o** was isolated as a colorless oil (27.2 mg, 62%). **^{1}H NMR** (400 MHz, $CDCl_3$) δ 7.74 (d, J = 8.1 Hz, 2H), 7.27 (d, J = 8.1 Hz, 2H), 4.04 (t, J = 6.4 Hz, 2H), 3.91 (ddt, J = 14.0, 6.7, 3.8 Hz, 1H), 3.37 (t, J = 6.4 Hz, 2H), 3.30 (t, J = 6.3 Hz, 2H), 3.22 (ddd, J = 14.1, 9.9, 5.2 Hz, 1H), 2.41 (s, 3H), 2.32 (ddt, J = 13.5, 8.6, 2.1 Hz, 1H), 2.15-2.08 (m, 1H), 2.02 – 1.92 (m, 1H), 1.91 – 1.80 (m, 5H), 1.79 – 1.65 (m, 2H), 1.64 – 1.32 (m, 6H) ppm; **^{13}C NMR** (100 MHz, $CDCl_3$) δ 155.3, 143.3, 139.1, 129.5 (2C), 127.5 (d, J = 2.1 Hz, 2C), 110.9 (d, J = 222.4 Hz), 64.7, 46.3, 45.9, 42.8, 41.1 (d, J = 22.2 Hz), 40.6 (d, J = 6.3 Hz), 39.2 (d, J = 18.1 Hz), 38.3 (d, J = 7.0 Hz), 33.4, 31.5, 27.7, 25.9, 25.1, 21.7 ppm; **^{19}F NMR** (376 MHz, $CDCl_3$) δ -134.52 ppm; **IR** (ν_{max}, cm^{-1}) 2933 (m), 1690 (s), 1416 (s), 1335 (s), 1158 (s), 1105 (s), 817 (s), 685 (s); **HRMS** (ESI/QTOF) m/z: [M + Na]$^+$ Calcd for $C_{22}H_{31}FN_2NaO_4S^+$ 461.1881; Found 461.1895.

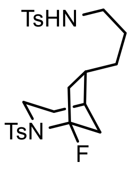

5p

***N*-(3-(1-Fluoro-2-tosyl-2-azabicyclo[3.2.1]octan-6-yl)propyl)-4-methylbenzenesulfonamide (5p)**: Following the general procedure (0.042 mmol scale), compound **5p** was isolated as a colorless oil (10.6 mg, 51%). **^{1}H NMR** (400 MHz, $CDCl_3$) δ 7.73 (d, J = 8.5 Hz, 2H), 7.72 (d, J = 8.2 Hz, 2H), 7.31 (d, J = 8.1 Hz, 2H), 7.27 (d, J = 7.8 Hz, 2H), 4.42 (t, J = 6.3 Hz, 1H), 3.86 (ddt, J = 14.2, 6.4, 4.1 Hz, 1H), 3.20 (ddd, J = 14.0, 9.6, 5.3 Hz, 1H), 2.92 (q, J = 6.5 Hz, 2H), 2.43 (s, 3H), 2.42 (s, 3H), 2.29 (ddt, J = 13.6, 8.7, 2.3 Hz, 1H), 2.05-1.99 (m, 1H), 1.89 (dtd, J = 9.5, 6.3, 5.4, 1.6 Hz, 1H), 1.79 (d, J = 12.6 Hz, 1H), 1.73 (dtd, J = 10.0, 6.7, 3.7 Hz, 1H), 1.65 – 1.56 (m, 1H), 1.53 – 1.46 (m, 1H), 1.46 – 1.23 (m, 5H) ppm; **^{13}C NMR** (100 MHz, $CDCl_3$) δ 143.7, 143.3, 139.0, 137.0, 129.9 (2C), 129.5 (2C), 127.4 (d, J = 2.0 Hz, 2C), 127.2 (2C), 110.8 (d, J = 222.5 Hz), 43.2, 42.6, 41.0 (d, J = 22.5 Hz), 40.5 (d, J = 6.4 Hz), 39.1 (d, J = 18.0 Hz), 38.2 (d, J = 6.9 Hz), 33.9, 31.5, 28.1, 21.7, 21.7 ppm; **^{19}F NMR** (376 MHz, $CDCl_3$) δ -134.31 ppm; **IR** (ν_{max}, cm^{-1}) 3273 (w), 2933 (w), 1597 (w), 1446 (w), 1327 (m), 1157 (s), 1092 (m), 918 (w), 816 (m), 688 (m); **HRMS** (ESI/QTOF) m/z: [M + Na]$^+$ Calcd for $C_{24}H_{31}FN_2NaO_4S_2^+$ 517.1601; Found 517.1612.

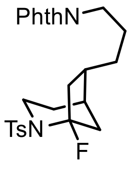

5q

2-(3-(1-Fluoro-2-tosyl-2-azabicyclo[3.2.1]octan-6-yl)propyl)isoindoline-1,3-dione (5q): Following the general procedure, compound **5q** was isolated as a colorless oil (33.0 mg, 70%). **^{1}H NMR** (400 MHz, $CDCl_3$) δ 7.87 – 7.80 (m, 2H), 7.76 – 7.68 (m, 4H), 7.26 (d, J = 8.2 Hz, 2H), 3.91 (ddt, J = 13.8, 6.4, 3.7 Hz, 1H), 3.65 (t, J = 7.1 Hz, 2H), 3.20 (ddd, J = 13.4, 10.1, 5.2 Hz, 1H), 2.41 (s, 3H), 2.28 (ddt, J = 13.5, 8.7, 2.2 Hz, 1H), 2.13-2.06 (m, 1H), 1.99 – 1.90 (m, 1H), 1.81 (d, J = 11.4 Hz, 1H), 1.78 – 1.57 (m, 4H), 1.55 – 1.32 (m, 4H) ppm; **^{13}C NMR** (100 MHz, $CDCl_3$) δ 168.5 (2C), 143.3, 139.0, 134.1 (2C), 132.2 (2C), 129.5 (2C), 127.5 (d, J = 2.0 Hz, 2C), 123.4 (2C), 110.9 (d, J = 222.3 Hz), 42.8, 40.9 (d, J = 22.2 Hz), 40.5 (d, J = 6.3 Hz), 39.3 (d, J = 18.2 Hz), 38.3 (d, J = 7.0 Hz), 37.9, 34.1, 31.5, 27.2, 21.7 ppm; **^{19}F NMR** (376 MHz, $CDCl_3$) δ -134.64 ppm; **IR** (ν_{max}, cm^{-1}) 2931 (w), 2873 (w), 1768 (w), 1709 (s),

1442 (w), 1333 (m), 1153 (m), 1078 (m), 816 (w), 719 (s), 688 (m); **HRMS** (ESI/QTOF) m/z: $[M + H]^+$ Calcd for $C_{25}H_{28}FN_2O_4S^+$ 471.1748; Found 471.1752.

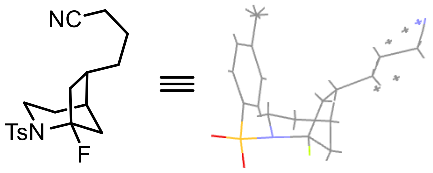

5r

CCDC: 2417051

4-(1-Fluoro-2-tosyl-2-azabicyclo[3.2.1]octan-6-yl)butanenitrile (5r): Following the general procedure, compound **5r** was isolated as a white solid (26.3 mg, 75%). m.p. = 106 ~ 108 ^oC; ^{1}**H NMR** (400 MHz, $CDCl_3$) δ 7.74 (d, J = 8.0 Hz, 2H), 7.27 (d, J = 8.3 Hz, 2H), 3.87 (ddt, J = 13.3, 6.4, 4.1 Hz, 1H), 3.24 (dddd, J = 13.3, 9.5, 5.4, 1.1 Hz, 1H), 2.41 (s, 3H), 2.41-2.37 (m, 1H), 2.34 (t, J = 6.8 Hz, 2H), 2.14-2.08 (m, 1H), 1.97 (dddd, J = 13.1, 7.4, 5.4, 1.6 Hz, 1H), 1.85 (d, J = 11.4 Hz, 1H), 1.78 (dtd, J = 13.6, 6.2, 3.2 Hz, 1H), 1.73 – 1.66 (m, 1H), 1.66 – 1.42 (m, 6H) ppm; ^{13}**C NMR** (100 MHz, $CDCl_3$) δ 143.4, 138.9, 129.5 (2C), 127.4 (d, J = 2.1 Hz, 2C), 119.5, 110.6 (d, J = 222.8 Hz), 42.5, 40.9 (d, J = 22.9 Hz), 40.4 (d, J = 6.4 Hz), 39.1 (d, J = 18.1 Hz), 38.1 (d, J = 6.9 Hz), 35.9, 31.5, 23.9, 21.7, 17.3 ppm; ^{19}**F NMR** (376 MHz, $CDCl_3$) δ -134.20 ppm; **IR** (ν_{max}, cm^{-1}) 2922 (m), 1599 (w), 1446 (w), 1329 (s), 1157 (s), 1086 (m), 991 (m), 812 (m), 690 (s); **HRMS** (ESI/QTOF) m/z: $[M + Na]^+$ Calcd for $C_{18}H_{23}FN_2NaO_2S^+$ 373.1356; Found 373.1362.

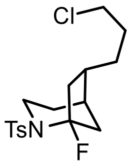

5s

6-(3-Chloropropyl)-1-fluoro-2-tosyl-2-azabicyclo[3.2.1]octane (5s): Following the general procedure, compound **5s** was isolated as a colorless oil (28.5 mg, 79%). ^{1}**H NMR** (400 MHz, $CDCl_3$) δ 7.75 (d, J = 8.0 Hz, 2H), 7.28 (d, J = 8.0 Hz, 2H), 3.90 (ddt, J = 13.3, 6.4, 4.1 Hz, 1H), 3.51 (t, J = 6.4 Hz, 2H), 3.28 – 3.19 (m, 1H), 2.42 (s, 3H), 2.36 (ddt, J = 13.8, 8.5, 2.2 Hz, 1H), 2.12 (p, J = 3.5 Hz, 1H), 1.98 (dddd, J = 11.1, 7.4, 5.4, 1.6 Hz, 1H), 1.85 (d, J = 11.3 Hz, 1H), 1.82 – 1.65 (m, 4H), 1.64 – 1.42 (m, 4H) ppm; ^{13}**C NMR** (100 MHz, $CDCl_3$) δ 143.3, 139.0, 129.5 (2C), 127.5 (d, J = 2.1 Hz, 2C), 110.8 (d, J = 222.6 Hz), 44.9, 42.7, 41.1 (d, J =

22.4 Hz), 40.4 (d, *J* = 6.4 Hz), 39.2 (d, *J* = 18.2 Hz), 38.3 (d, *J* = 7.0 Hz), 34.3, 31.6, 31.0, 21.7 ppm; **^{19}F NMR** (376 MHz, $CDCl_3$) δ -134.35 ppm; **IR** (ν_{max}, cm^{-1}) 2930 (w), 1438 (w), 1334 (m), 1157 (m), 1087 (m), 984 (m), 814 (m), 686 (m); **HRMS** (ESI/QTOF) m/z: [M + Na]$^+$ Calcd for $C_{17}H_{23}ClFNNaO_2S^+$ 382.1014; Found 382.1022.

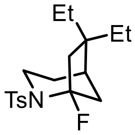

5t

6,6-Diethyl-1-fluoro-2-tosyl-2-azabicyclo[3.2.1]octane (5t): Following the general procedure, compound **5t** was isolated as a colorless oil (14.6 mg, 43%). **^{1}H NMR** (600 MHz, $CDCl_3$) δ 7.75 (d, *J* = 8.0 Hz, 2H), 7.27 (d, *J* = 8.0 Hz, 2H), 3.61 (ddt, *J* = 12.9, 7.2, 5.2 Hz, 1H), 3.42 (dtd, *J* = 13.8, 7.0, 2.4 Hz, 1H), 2.41 (s, 3H), 2.20 – 2.13 (m, 1H), 2.05 (dd, *J* = 13.9, 1.7 Hz, 1H), 2.01 (hept, *J* = 2.5 Hz, 1H), 1.90 (dddt, *J* = 14.1, 7.0, 5.2, 1.9 Hz, 1H), 1.85 (dd, *J* = 11.6, 2.5 Hz, 1H), 1.74 (t, *J* = 13.2 Hz, 1H), 1.66 (dtd, *J* = 14.3, 7.2, 5.7 Hz, 1H), 1.50 – 1.39 (m, 4H), 0.79 (t, *J* = 7.6 Hz, 3H), 0.78 (t, *J* = 7.6 Hz, 3H) ppm; **^{13}C NMR** (150 MHz, $CDCl_3$) δ 143.2, 139.1, 129.5 (2C), 127.4 (d, *J* = 2.2 Hz, 2C), 110.2 (d, *J* = 224.2 Hz), 46.7 (d, *J* = 22.9 Hz), 44.4 (d, *J* = 5.5 Hz), 42.0, 39.6 (d, *J* = 17.6 Hz), 39.5 (d, *J* = 6.6 Hz), 31.2, 26.0, 25.2, 21.7, 8.9, 8.7 ppm; **^{19}F NMR** (376 MHz, $CDCl_3$) δ -128.31 ppm; **IR** (ν_{max}, cm^{-1}) 2964 (w), 1348 (m), 1162 (s), 1129 (m), 989 (m), 708 (m), 563 (m), 549 (m); **HRMS** (ESI/QTOF) m/z: [M + Na]$^+$ Calcd for $C_{18}H_{26}FNNaO_2S^+$ 362.1560; Found 362.1556.

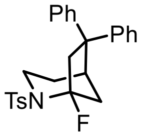

5u

1-Fluoro-6,6-diphenyl-2-tosyl-2-azabicyclo[3.2.1]octane (5u): Following the general procedure, compound **5u** was isolated as a colorless oil (28.8 mg, 66%). **^{1}H NMR** (600 MHz, $CDCl_3$) δ 7.78 (d, *J* = 8.0 Hz, 2H), 7.31 (d, *J* = 8.0 Hz, 2H), 7.28 – 7.20 (m, 6H), 7.20 – 7.16 (m, 2H), 7.13 (tt, *J* = 6.8, 1.3 Hz, 1H), 7.08 (tt, *J* = 6.8, 1.6 Hz, 1H), 3.82 (dddd, *J* = 13.2, 6.6, 4.3, 2.4 Hz, 1H), 3.50 (p, *J* = 3.8 Hz, 1H), 3.26 (dd, *J* = 15.1, 2.2 Hz, 1H), 2.90 (ddd, *J* = 13.3, 11.6, 4.8 Hz, 1H), 2.66 (t, *J* = 16.0 Hz, 1H), 2.46 (s, 3H), 2.33 (ddq, *J* = 12.1, 6.8, 2.0 Hz, 1H), 2.12 (d, *J* = 11.0 Hz, 1H), 1.75 (dddd, *J* = 13.9, 11.6, 6.7, 3.7 Hz, 1H), 1.62 – 1.55 (m, 1H)

ppm; ^{13}**C NMR** (150 MHz, $CDCl_3$) δ 150.4, 145.0, 143.5, 139.0, 129.5 (2C), 128.8 (2C), 128.7 (2C), 127.6 (d, J = 2.0 Hz, 2C), 127.1 (2C), 126.2, 126.1 (2C), 125.8, 111.2 (d, J = 220.0 Hz), 55.4 (d, J = 6.1 Hz), 46.2 (d, J = 23.1 Hz), 43.1, 41.5 (d, J = 18.9 Hz), 41.2 (d, J = 6.7 Hz), 27.0, 21.7 ppm; ^{19}**F NMR** (376 MHz, $CDCl_3$) δ -132.36 ppm; **IR** (ν_{max}, cm^{-1}) 2960 (w), 1447 (m), 1340 (m), 1153 (s), 1021 (m), 814 (m), 693 (s), 572 (m), 543 (s); **HRMS** (ESI/QTOF) m/z: $[M + Na]^+$ Calcd for $C_{26}H_{26}FNNaO_2S^+$ 458.1560; Found 458.1565.

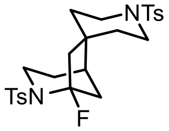

5v

1-Fluoro-1',2-ditosyl-2-azaspiro[bicyclo[3.2.1]octane-6,4'-piperidine] (5v): Following the general procedure, compound **5v** was isolated as white solid (42.1 mg, 83%). m.p. = 187 ~ 189 ^{o}C; ^{1}**H NMR** (400 MHz, $CDCl_3$) δ 7.70 (d, J = 8.1 Hz, 2H), 7.62 (d, J = 8.2 Hz, 2H), 7.33 (d, J = 8.1 Hz, 2H), 7.25 (d, J = 8.0 Hz, 2H), 3.58 – 3.47 (m, 3H), 3.35 (dtd, J = 13.4, 6.8, 2.5 Hz, 1H), 2.46 (s, 3H), 2.40 (s, 3H), 2.43 – 2.33 (m, 2H), 2.13 (d, J = 14.1 Hz, 1H), 2.10 – 2.03 (m, 1H), 1.99 – 1.88 (m, 2H), 1.87 (dd, J = 11.6, 2.5 Hz, 1H), 1.75 – 1.57 (m, 6H) ppm; ^{13}**C NMR** (100 MHz, $CDCl_3$) δ 143.9, 143.4, 138.8, 132.5, 129.9 (2C), 129.5 (2C), 127.8 (2C), 127.3 (d, J = 2.2 Hz, 2C), 109.6 (d, J = 225.0 Hz), 44.8 (d, J = 24.8 Hz), 44.2, 43.4, 41.5 (d, J = 6.3 Hz), 41.3, 39.6 (d, J = 5.8 Hz), 39.4, 38.8 (d, J = 17.6 Hz), 33.0, 25.2, 21.7, 21.6 ppm; ^{19}**F NMR** (376 MHz, $CDCl_3$) δ -128.04 ppm; **IR** (ν_{max}, cm^{-1}) 2937 (w), 2844 (w), 1597 (w), 1466 (w), 1340 (m), 1165 (s), 1092 (m), 1051 (m), 995 (w), 945 (m), 816 (m), 725 (s), 688 (m); **HRMS** (ESI/QTOF) m/z: $[M + Na]^+$ Calcd for $C_{25}H_{31}FN_2NaO_4S_2^+$ 529.1601; Found 529.1623.

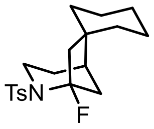

5w

1-Fluoro-2-tosyl-2-azaspiro[bicyclo[3.2.1]octane-6,1'-cyclohexane] (5w): Following the general procedure, compound **5w** was isolated as a white solid (18.6 mg, 53%). m.p. = 61 ~ 63 ^{o}C; ^{1}**H NMR** (400 MHz, $CDCl_3$) δ 7.75 (d, J = 8.0 Hz, 2H), 7.27 (d, J = 8.0 Hz, 2H), 3.72 (ddt, J = 13.0, 7.1, 4.5 Hz, 1H), 3.36 (dddd, J = 13.1, 8.3, 6.2, 1.8 Hz, 1H), 2.41 (s, 3H), 2.17 (dddd, J = 11.1, 8.7, 5.3, 1.9 Hz, 1H), 2.07 (dd, J = 14.2, 2.5 Hz, 1H), 2.00 (dp, J = 5.5, 2.7 Hz, 1H),

1.94 – 1.85 (m, 1H), 1.84 (dd, *J* = 11.5, 2.5 Hz, 1H), 1.76 (t, *J* = 13.9 Hz, 1H), 1.69 – 1.59 (m, 1H), 1.58 – 1.42 (m, 5H), 1.41 – 1.21 (m, 5H) ppm; **^{13}C NMR** (100 MHz, $CDCl_3$) δ 143.2, 139.2, 129.5 (2C), 127.4 (d, *J* = 2.2 Hz, 2C), 110.5 (d, *J* = 223.2 Hz), 46.2 (d, *J* = 22.8 Hz), 42.3, 41.7 (d, *J* = 5.6 Hz), 41.5 (d, *J* = 5.7 Hz), 40.5, 39.7 (d, *J* = 17.6 Hz), 33.9, 26.0, 25.6, 23.8, 23.2, 21.7 ppm; **^{19}F NMR** (376 MHz, $CDCl_3$) δ -129.15 ppm; **IR** (ν_{max}, cm^{-1}) 2922 (m), 2852 (m), 1601 (w), 1450 (m), 1344 (s), 1161 (s), 989 (m), 812 (m), 696 (s); **HRMS** (ESI/QTOF) m/z: $[M + Na]^+$ Calcd for $C_{19}H_{26}FNNaO_2S^+$ 374.1560; Found 374.1555.

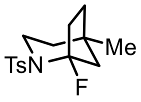

5x

0.05 mmol scale, 0.1 equiv Pd loading: 32% HNMR yield of **5x**; 0.1 mmol scale, 0.05 equiv Pd loading, 23% yield of **5x**.

1-Fluoro-5-methyl-2-tosyl-2-azabicyclo[3.2.1]octane (5x): Following the general procedure, compound **5x** was isolated as a colorless oil (6.9 mg, 23%). **^{1}H NMR** (800 MHz, $CDCl_3$) δ 7.75 (d, *J* = 7.9 Hz, 2H), 7.27 (d, *J* = 8.7 Hz, 2H), 4.16 – 4.11 (m, 1H), 3.13 (tdd, *J* = 12.2, 4.5, 1.4 Hz, 1H), 2.41 (s, 3H), 1.98 – 1.90 (m, 2H), 1.82 – 1.76 (m, 2H), 1.65 – 1.59 (m, 1H), 1.59 – 1.54 (m, 1H), 1.43 (dt, *J* = 14.1, 7.2 Hz, 1H), 1.36 (d, *J* = 15.1 Hz, 1H), 1.09 (s, 3H) ppm; **^{13}C NMR** (200 MHz, $CDCl_3$) δ 143.3, 139.2, 129.5 (2C), 127.5 (d, *J* = 1.6 Hz, 2C), 111.4 (d, *J* = 220.0 Hz), 48.6 (d, *J* = 17.5 Hz), 43.6, 40.5 (d, *J* = 7.2 Hz), 37.9, 34.6 (d, *J* = 21.5 Hz), 33.8 (d, *J* = 7.0 Hz), 26.6, 21.7 ppm; **^{19}F NMR** (376 MHz, $CDCl_3$) δ -135.65 ppm; **IR** (ν_{max}, cm^{-1}) 2951 (w), 2871 (w), 1336 (m), 1153 (s), 1114 (m), 815 (m), 687 (s), 620 (m), 550 (s); **HRMS** (ESI/QTOF) m/z: $[M + Na]^+$ Calcd for $C_{15}H_{20}FNNaO_2S^+$ 320.1091; Found 320.1094.

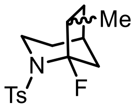

dr = 1.9:1
5xa

1-Fluoro-7-methyl-2-tosyl-2-azabicyclo[3.2.1]octane (5xa): Following the general procedure, compound **5xa** was isolated as an inseparable colorless oil (8.3 mg, 28%, dr = 1.9:1). **^{1}H NMR** (600 MHz, $CDCl_3$) δ 7.77 – 7.72 (m, 2H, major + 2H, minor), 7.27 (d, *J* = 8.0 Hz, 2H, major), 7.25 (d, *J* = 8.1 Hz, 2H, minor), 4.40 – 4.34 (m, 1H, minor), 4.00 – 3.94 (m, 1H,

major), 3.30 (ddd, J = 14.7, 12.4, 4.5 Hz, 1H, minor), 3.16 (ddd, J = 13.1, 10.8, 5.0 Hz, 1H, major), 2.51 – 2.41 (m, 1H, major + 1H, minor), 2.41 (s, 3H, major), 2.40 (s, 3H, minor), 2.35 – 2.16 (m, 4H, major + minor), 1.99 – 1.91 (m, 2H), 1.79 – 1.66 (m, 4H), 1.51 – 1.44 (m, 2H), 1.41 – 1.35 (m, 1H, minor), 1.16 – 1.07 (m, 1H, major), 1.02 (d, J = 6.4 Hz, 3H, minor), 0.91 (dd, J = 7.2, 3.2 Hz, 3H, major) ppm; **^{13}C NMR** (150 MHz, $CDCl_3$) δ 143.2 (major), 142.8 (minor), 140.6 (minor), 139.3 (major), 129.4 (major, 2C), 129.3 (minor, 2C), 127.5 (d, J = 1.6 Hz, major, 2C), 127.2 (d, J = 1.6 Hz, minor, 2C), 111.8 (d, J = 226.5 Hz, major), 111.7 (d, J = 219.2 Hz, minor), 42.6 (major), 42.5 (minor), 41.9 (d, J = 19.1 Hz, minor), 40.7 (d, J = 20.3 Hz, minor), 38.9 (d, J = 18.5 Hz, major), 37.41 (d, J = 20.7 Hz, major), 37.38 (d, J = 4.3 Hz, major), 33.3 (d, J = 8.8 Hz, minor), 33.2 (d, J = 6.8 Hz, minor), 31.4 (d, J = 8.7 Hz, major), 31.2 (minor), 30.8 (major), 21.7 (major), 21.6 (minor), 18.4 (d, J = 7.8 Hz, major), 11.6 (minor) ppm; **^{19}F NMR** (377 MHz, $CDCl_3$) δ -143.04, -147.32 ppm; **IR** (ν_{max}, cm^{-1}) 3285 (w), 2929 (m), 2870 (w), 1734 (m), 1326 (m), 1159 (s), 1088 (m), 973 (w), 813 (m), 670 (s), 609 (m); **HRMS** (ESI/QTOF) m/z: [M + Na]$^+$ Calcd for $C_{15}H_{20}FNNaO_2S^+$ 320.1091; Found 320.1098.

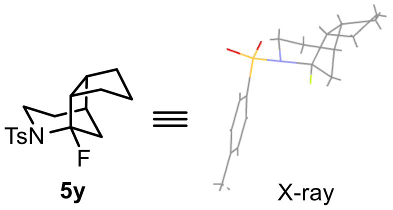

5y

CCDC: 2417052

1-Fluoro-2-tosyldecahydro-1,5-methanocyclopenta[c]azepine (5y): Following the general procedure, compound **5y** was isolated as a white solid (13.3 mg, 41%). m.p. = 86 ~ 88 °C; **^{1}H NMR** (400 MHz, CD_2Cl_2) δ 7.71 (d, J = 8.0 Hz, 2H), 7.30 (d, J = 8.0 Hz, 2H), 3.98 (dddd, J = 13.1, 6.6, 4.3, 2.4 Hz, 1H), 3.10 (ddd, J = 13.0, 11.5, 4.8 Hz, 1H), 2.42 (s, 3H), 2.28 (tdd, J = 10.6, 7.8, 2.1 Hz, 1H), 2.12 (qd, J = 8.8, 1.8 Hz, 1H), 2.05 – 1.91 (m, 3H), 1.76 – 1.58 (m, 4H), 1.57 – 1.48 (m, 1H), 1.41 – 1.20 (m, 2H), 1.14 (tdd, J = 11.8, 8.4, 5.8 Hz, 1H) ppm; **^{13}C NMR** (100 MHz, CD_2Cl_2) δ 143.7, 139.4, 129.7 (2C), 127.6 (d, J = 2.0 Hz, 2C), 112.6 (d, J = 224.0 Hz), 49.0 (d, J = 19.9 Hz), 47.4 (d, J = 4.7 Hz), 43.6, 38.1 (d, J = 7.8 Hz), 37.7 (d, J = 18.7 Hz), 34.1, 31.0, 29.6 (d, J = 3.7 Hz), 28.1, 21.6 ppm; **^{19}F NMR** (376 MHz, CD_2Cl_2) δ -147.06 ppm; **IR** (ν_{max}, cm^{-1}) 2941 (m), 2862 (m), 1599 (w), 1454 (m), 1333 (s), 1157 (s), 1080 (s), 989 (m), 816 (m), 717 (s); **HRMS** (ESI/QTOF) m/z: [M + H]$^+$ Calcd for $C_{17}H_{23}FNO_2S^+$ 324.1428; Found 324.1433.

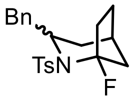

5z

3-Benzyl-1-fluoro-2-tosyl-2-azabicyclo[3.2.1]octane (5z): Following the general procedure, compound **5z** was isolated as an inseparable mixture of two diastereoisomers as a colorless oil (31.8 mg, 85%, dr = 1.1:1). **^{1}H NMR** (600 MHz, $CDCl_3$) δ 7.93 (d, J = 8.0 Hz, 2H), 7.48 (d, J = 8.0 Hz, 2H), 7.36 – 7.22 (m, 9H), 7.20-7.17 (m, 3H), 6.97 (d, J = 7.4 Hz, 2H), 4.92 (tt, J = 10.0, 5.1 Hz, 1H), 4.22 (qt, J = 9.3, 2.6 Hz, 1H), 3.31 (dd, J = 13.1, 5.9 Hz, 1H), 2.97 (dd, J = 13.1, 10.4 Hz, 1H), 2.90-2.87 (m, 1H), 2.85 (dd, J = 13.2, 3.2 Hz, 1H), 2.57 (t, J = 12.5 Hz, 1H), 2.53-2.49 (m, 1H), 2.41 (s, 3H), 2.39 (s, 3H), 2.41 – 2.33 (m, 2H), 2.18 – 1.95 (m, 6H), 1.86 – 1.75 (m, 4H), 1.73-1.68 (m, 1H), 1.64 – 1.58 (m, 2H), 1.36 (dt, J = 15.1, 8.3 Hz, 1H) ppm; **^{13}C NMR** (150 MHz, $CDCl_3$) δ 143.6, 142.8, 140.8, 139.3, 139.1, 138.5, 130.0, 129.7, 129.32, 129.28, 128.73, 128.71, 127.9 (d, J = 2.2 Hz), 127.2 (d, J = 2.5 Hz), 126.70, 126.69, 111.7 (d, J = 217.7 Hz), 109.2 (d, J = 226.4 Hz), 56.3, 54.4, 45.8, 43.9, 41.2 (d, J = 19.4 Hz), 39.5 (d, J = 16.7 Hz), 36.0, 35.4 (d, J = 13.7 Hz), 35.3, 34.9 (d, J = 28.3 Hz), 31.4, 29.4 (d, J = 6.6 Hz), 29.3 (d, J = 7.0 Hz), 26.4 (d, J = 8.2 Hz), 21.7, 21.6 ppm; **^{19}F NMR** (376 MHz, $CDCl_3$) δ -123.04, -136.88 ppm; **IR** (ν_{max}, cm^{-1}) 2954 (w), 1599 (w), 1495 (w), 1450 (w), 1327 (s), 1149 (s), 1092 (m), 1009 (m), 945 (m), 816 (m), 742 (m), 702 (s), 663 (s); **HRMS** (ESI/QTOF) m/z: $[M + Na]^+$ Calcd for $C_{21}H_{24}FNNaO_2S^+$ 396.1404; Found 396.1399.

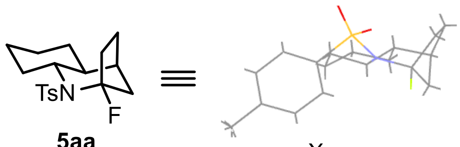

5aa X-ray
CCDC: 2417053

2-Fluoro-1-tosyldecahydro-1H-2,5-methanobenzo[b]azepine (5aa): Following the general procedure, compound **5aa** was isolated as a white solid (20.6 mg, 61%). m.p. = 127 ~ 129 ^{o}C; **^{1}H NMR** (600 MHz, $CDCl_3$) δ 7.80 (d, J = 8.0 Hz, 2H), 7.27 (d, J = 8.2 Hz, 2H), 3.02 (tt, J = 10.8, 3.7 Hz, 1H), 2.89 (ddt, J = 12.8, 9.6, 2.7 Hz, 1H), 2.41 (s, 3H), 2.26 (d, J = 11.0 Hz, 1H), 2.24 – 2.16 (m, 1H), 2.04 (tt, J = 12.5, 5.8 Hz, 1H), 1.87 – 1.79 (m, 3H), 1.76 – 1.64 (m, 3H), 1.63 – 1.55 (m, 2H), 1.41 (td, J = 12.4, 11.4, 3.2 Hz, 1H), 1.20 (qt, J = 12.9, 3.8 Hz, 1H), 1.10 (qd, J = 12.7, 3.5 Hz, 1H), 0.99 (qt, J = 13.3, 3.8 Hz, 1H) ppm; **^{13}C NMR** (150 MHz, $CDCl_3$) δ 142.8, 141.3, 129.7 (2C), 127.0 (d, J = 3.1 Hz, 2C), 111.2 (d, J = 231.9 Hz), 59.0, 53.1, 36.8

(d, *J* = 18.5 Hz), 36.0 (d, *J* = 7.8 Hz), 35.0 (d, *J* = 28.8 Hz), 32.9, 30.9 (d, *J* = 6.2 Hz), 30.0, 26.4, 25.8, 21.6 ppm; **^{19}F NMR** (376 MHz, $CDCl_3$) *δ* -124.40 ppm; **IR** (ν_{max}, cm^{-1}) 2925 (w), 1597 (w), 1456 (w), 1329 (m), 1157 (s), 1097 (m), 1003 (m), 816 (m), 717 (s), 663 (m); **HRMS** (ESI/QTOF) m/z: $[M + Na]^+$ Calcd for $C_{18}H_{24}FNNaO_2S^+$ 360.1404; Found 360.1405.

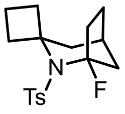

5ab

1-Fluoro-2-tosyl-2-azaspiro[bicyclo[3.2.1]octane-3,1'-cyclobutane] (5ab): Following the general procedure, compound **5ab** was isolated as a colorless oil (27.2 mg, 84%). **^{1}H NMR** (400 MHz, $CDCl_3$) δ 7.87 (d, *J* = 8.4 Hz, 2H), 7.30 (d, *J* = 8.1 Hz, 2H), 2.95 – 2.81 (m, 2H), 2.49 – 2.41 (m, 1H), 2.43 (s, 3H), 2.41 – 2.29 (m, 2H), 2.20 – 2.03 (m, 3H), 2.02 – 1.91 (m, 2H), 1.90 – 1.82 (m, 1H), 1.79 (d, *J* = 13.2 Hz, 1H), 1.67 – 1.49 (m, 2H), 1.39 (qt, *J* = 11.2, 2.8 Hz, 1H) ppm; **^{13}C NMR** (100 MHz, $CDCl_3$) *δ* 142.9, 141.7, 129.6 (2C), 127.2 (d, *J* = 2.4 Hz, 2C), 110.9 (d, *J* = 227.8 Hz), 60.3 (d, *J* = 1.3 Hz), 46.4, 39.7 (d, *J* = 1.9 Hz), 39.2 (d, *J* = 18.0 Hz), 35.4 (d, *J* = 1.9 Hz), 35.1 (d, *J* = 27.8 Hz), 30.6 (d, *J* = 6.8 Hz), 29.8 (d, *J* = 7.7 Hz), 21.7, 14.7 ppm; **^{19}F NMR** (376 MHz, $CDCl_3$) *δ* -122.36 ppm; **IR** (ν_{max}, cm^{-1}) 2943 (w), 1603 (w), 1454 (w), 1344 (s), 1153 (s), 1088 (s), 1018 (m), 966 (m), 816 (m), 723 (m), 673 (s); **HRMS** (ESI/QTOF) m/z: $[M + Na]^+$ Calcd for $C_{17}H_{22}FNNaO_2S^+$ 346.1247; Found 346.1248.

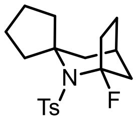

5ac

1-Fluoro-2-tosyl-2-azaspiro[bicyclo[3.2.1]octane-3,1'-cyclopentane] (5ac): Following the general procedure, compound **5ac** was isolated as a colorless oil (15.2 mg, 45%), which turned into a white solid in the freezer. **^{1}H NMR** (800 MHz, $CDCl_3$) *δ* 7.74 (d, *J* = 8.1 Hz, 2H), 7.24 (d, *J* = 8.1 Hz, 2H), 2.93 (ddd, *J* = 13.1, 9.4, 5.3 Hz, 1H), 2.74-2.69 (m, 1H), 2.51 (dt, *J* = 12.1, 8.8 Hz, 1H), 2.39 (s, 3H), 2.37 – 2.33 (m, 1H), 2.13 – 2.06 (m, 2H), 2.06 – 2.00 (m, 1H), 1.94 – 1.88 (m, 3H), 1.87 – 1.78 (m, 3H), 1.74 – 1.67 (m, 2H), 1.57 – 1.48 (m, 2H) ppm; **^{13}C NMR** (200 MHz, $CDCl_3$) δ 143.1, 142.3, 129.2 (2C), 126.4 (d, *J* = 3.0 Hz, 2C), 112.3 (d, *J* = 217.0 Hz), 71.1, 49.0, 44.0, 43.5 (d, *J* = 17.8 Hz), 39.4, 34.0 (d, *J* = 23.9 Hz), 33.2 (d, *J* = 9.5 Hz),

27.4 (d, J = 7.5 Hz), 25.4, 23.9, 21.6 ppm; **^{19}F NMR** (376 MHz, $CDCl_3$) δ -128.15 ppm; **IR** (ν_{max}, cm^{-1}) 2955 (m), 1339 (m), 1156 (s), 1092 (w), 814 (w), 685 (w), 663 (m), 558 (w); **HRMS** (ESI/QTOF) m/z: $[M + Na]^+$ Calcd for $C_{18}H_{24}FNNaO_2S^+$ 360.1404; Found 360.1410.

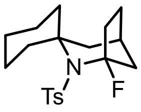

5ad

1-Fluoro-2-tosyl-2-azaspiro[bicyclo[3.2.1]octane-3,1'-cyclohexane] (5ad): Following the general procedure, compound **5ad** was isolated as a white solid (16.5 mg, 47%). m.p. = 95 ~ 97 °C; **^{1}H NMR** (400 MHz, $CDCl_3$) δ 7.73 (d, J = 8.1 Hz, 2H), 7.23 (d, J = 8.1 Hz, 2H), 3.21 (td, J = 12.8, 12.3, 4.5 Hz, 1H), 2.81 (tdd, J = 12.9, 4.8, 1.6 Hz, 1H), 2.69 – 2.60 (m, 1H), 2.39 (s, 3H), 2.44 – 2.32 (m, 2H), 2.14 – 1.98 (m, 3H), 1.89 (dddd, J = 12.3, 10.8, 5.3, 2.7 Hz, 1H), 1.78 – 1.51 (m, 7H), 1.50-1.38 (m, 2H), 1.36 – 1.22 (m, 1H) ppm; **^{13}C NMR** (100 MHz, $CDCl_3$) δ 144.2 (d, J = 2.5 Hz), 141.9, 129.1 (2C), 126.2 (d, J = 3.2 Hz, 2C), 112.0 (d, J = 216.1 Hz), 67.1, 43.3 (d, J = 17.9 Hz), 42.3 (d, J = 1.4 Hz), 41.1, 35.2 (d, J = 1.5 Hz), 34.8 (d, J = 24.2 Hz), 33.0 (d, J = 9.6 Hz), 27.0 (d, J = 8.0 Hz), 24.5, 24.4, 23.9, 21.6 ppm; **^{19}F NMR** (376 MHz, $CDCl_3$) δ -127.48 ppm; **IR** (ν_{max}, cm^{-1}) 2929 (s), 2870 (m), 1795 (m), 1450 (m), 1329 (s), 1248 (s), 1149 (s), 1038 (s), 916 (s), 810 (s), 737 (s), 669 (s); **HRMS** (ESI/QTOF) m/z: $[M + Na]^+$ Calcd for $C_{19}H_{26}FNNaO_2S^+$ 374.1560; Found 374.1558.

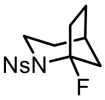

5ae

1-Fluoro-2-((4-nitrophenyl)sulfonyl)-2-azabicyclo[3.2.1]octane (5ae): Following the general procedure, compound **5ae** was isolated as a white solid (21.5 mg, 68%). **^{1}H NMR** (600 MHz, $CDCl_3$) δ 8.33 – 8.30 (m, 2H), 8.04 (d, J = 8.1 Hz, 2H), 4.06 (ddt, J = 13.5, 6.8, 3.5 Hz, 1H), 3.30 (ddd, J = 13.4, 10.9, 4.9 Hz, 1H), 2.54-2.50 (m, 1H), 2.17 – 2.11 (m, 1H), 2.10 – 2.02 (m, 1H), 1.97 (td, J = 15.6, 14.6, 6.0 Hz, 1H), 1.95 – 1.90 (m, 2H), 1.81 – 1.74 (m, 1H), 1.58-1.53 (m, 1H), 1.52 – 1.46 (m, 1H) ppm; **^{13}C NMR** (150 MHz, $CDCl_3$) δ 149.9, 148.2, 128.6 (d, J = 2.7 Hz, 2C), 124.1 (2C), 111.8 (d, J = 221.3 Hz), 42.9, 41.8 (d, J = 18.4 Hz), 33.9 (d, J = 22.1 Hz), 33.5 (d, J = 7.3 Hz), 31.3, 26.9 (d, J = 7.3 Hz) ppm; **^{19}F NMR** (376 MHz, $CDCl_3$) δ -136.80 ppm; **IR** (ν_{max}, cm^{-1}) 3107 (w), 2950 (w), 1527 (s), 1347 (s), 1159 (s), 1129 (s), 1088

(m), 996 (m), 855 (m), 739 (s), 686 (s), 666 (m), 619 (m), 593 (m), 464 (w); **HRMS** (nanochip-ESI/LTQ-Orbitrap) m/z: $[M + Na]^+$ Calcd for $C_{13}H_{15}FN_2NaO_4S^+$ 337.0629; Found 337.0631.

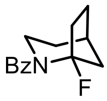

5af

1-Fluoro-2-azabicyclo[3.2.1]octan-2-yl)(phenyl)methanone (5af): Following the general procedure, compound **5af** was isolated as a white solid (18.5 mg, 79%). **^{1}H NMR** (600 MHz, $CDCl_3$) δ 7.47 – 7.44 (m, 2H), 7.42 – 7.35 (m, 3H), 3.70 (dddd, J = 14.0, 7.8, 6.3, 3.6 Hz, 1H), 3.48 (dddd, J = 14.1, 6.4, 5.3, 2.7 Hz, 1H), 2.56 (ddp, J = 13.5, 9.4, 1.9 Hz, 1H), 2.49-2.45 (m, 1H), 2.17 – 2.09 (m, 2H), 2.00 (tdd, J = 13.0, 10.8, 5.5 Hz, 1H), 1.94 (dddd, J = 11.6, 6.6, 5.0, 1.4 Hz, 1H), 1.81 (ddt, J = 13.3, 6.6, 5.3 Hz, 1H), 1.48 – 1.39 (m, 2H) ppm; **^{13}C NMR** (150 MHz, $CDCl_3$) δ 171.7, 137.8, 129.9, 128.3 (2C), 127.3 (br, 2C), 108.7 (d, J = 224.7 Hz), 42.4, 38.8 (d, J = 18.3 Hz), 32.8, 32.8 (d, J = 24.2 Hz), 31.0 (d, J = 7.2 Hz), 29.2 (d, J = 6.6 Hz) ppm; **^{19}F NMR** (376 MHz, $CDCl_3$) δ -133.42 ppm; **IR** (ν_{max}, cm^{-1}) 2948 (w), 1649 (s), 1446 (w), 1389 (s), 1262 (w), 1084 (w), 680 (w), 703 (m); **HRMS** (nanochip-ESI/LTQ-Orbitrap) m/z: $[M + H]^+$ Calcd for $C_{14}H_{17}FNO^+$ 234.1289; Found 234.1292.

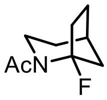

5ag

1-(1-Fluoro-2-azabicyclo[3.2.1]octan-2-yl)ethan-1-one (5ag): Following the general procedure, compound **5ag** was isolated as a colorless oil (8.3 mg, 48%). **^{1}H NMR** (600 MHz, $CDCl_3$) δ 4.00 (brs, 1H), 3.41 (dddd, J = 13.9, 9.7, 6.6, 3.6 Hz, 1H), 2.47 – 2.42 (m, 1H), 2.21 (d, J = 4.6 Hz, 3H), 2.20 – 2.10 (m, 2H), 2.09 – 2.01 (m, 2H), 1.93 – 1.86 (m, 2H), 1.44 (dddd, J = 13.8, 8.8, 7.2, 1.7 Hz, 1H), 1.40 – 1.34 (m, 1H) ppm; **^{13}C NMR** (150 MHz, $CDCl_3$) δ 171.0 (d, J = 3.3 Hz), 108.5 (d, J = 223.1 Hz), 38.8 (brd, J = 14.8 Hz), 37.1 (brs), 34.5 (brs), 32.0, 30.2, 29.7 (d, J = 6.6 Hz), 24.2 (d, J = 9.5 Hz) ppm; **^{19}F NMR** (376 MHz, $CDCl_3$) δ -128.07 (brs) ppm; **IR** (ν_{max}, cm^{-1}) 2946 (w), 1650 (s), 1385 (s), 1358 (m), 1281 (m), 1235 (m), 1181 (m), 1120 (m), 1100 (m), 1044 (m), 1024 (m), 938 (m), 574 (m); **HRMS** (nanochip-ESI/LTQ-Orbitrap) m/z: $[M + H]^+$ Calcd for $C_9H_{15}FNO^+$ 172.1132; Found 172.1135.

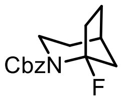

5ah

Benzyl-1-fluoro-2-azabicyclo[3.2.1]octane-2-carboxylate (5ah): Following the general procedure, compound **5ah** was isolated as a colorless oil (16.6 mg, 63%). **^{1}H NMR** (600 MHz, $CDCl_3$) δ 7.40 – 7.33 (m, 4H), 7.32 – 7.28 (m, 1H), 5.22 (d, J = 12.6 Hz, 1H), 5.19 (d, J = 12.6 Hz, 1H), 3.99 (dddd, J = 13.8, 7.7, 4.2, 1.6 Hz, 1H), 3.31 – 3.24 (m, 1H), 2.46 – 2.37 (m, 2H), 2.16 – 2.06 (m, 2H), 1.97 – 1.82 (m, 3H), 1.52 (dddd, J = 13.7, 11.0, 7.7, 1.2 Hz, 1H), 1.31-1.25 (m, 1H) ppm; **^{13}C NMR** (150 MHz, $CDCl_3$) δ 155.2, 136.7, 128.6 (2C), 128.0, 127.8 (2C), 107.4 (d, J = 225.9 Hz), 67.2, 38.3, 37.1 (d, J = 18.1 Hz), 33.2, 32.1 (d, J = 27.7 Hz), 30.2 (d, J = 6.1 Hz), 28.8 (d, J = 7.2 Hz) ppm; **^{19}F NMR** (376 MHz, $CDCl_3$) δ -131.32 ppm; **IR** (ν_{max}, cm^{-1}) 2948 (w), 1698 (s), 1404 (s), 1358 (s), 1315 (m), 1283 (s), 1184 (m), 1112 (m), 1073 (w), 990 (w), 772 (w), 697 (m); **HRMS** (nanochip-ESI/LTQ-Orbitrap) m/z: $[M + H]^+$ Calcd for $C_{15}H_{19}FNO_2^+$ 264.1394; Found 264.1397.

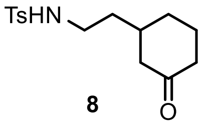

8

4-Methyl-*N*-(2-(3-oxocyclohexyl)ethyl)benzenesulfonamide (8): Following the general procedure to afford the crude residue, which was loaded onto the preparative thin layer chromatography and allowed to stand for two hours before separation. Compound **8** was isolated as a colorless oil (20.6 mg, 70%). **^{1}H NMR** (500 MHz, $CDCl_3$) δ 7.73 (d, J = 8.3 Hz, 2H), 7.31 (d, J = 8.2 Hz, 2H), 4.72 (brs, 1H), 2.98 – 2.91 (m, 2H), 2.43 (s, 3H), 2.37 – 2.29 (m, 2H), 2.26 – 2.17 (m, 1H), 2.03 – 1.91 (m, 2H), 1.86-1.77 (m, 2H), 1.64 – 1.50 (m, 2H), 1.46 (dp, J = 14.0, 7.0 Hz, 1H), 1.29 (dtd, J = 14.8, 11.5, 3.6 Hz, 1H) ppm; **^{13}C NMR** (125 MHz, $CDCl_3$) δ 211.3, 143.7, 136.8, 129.9 (2C), 127.2 (2C), 47.7, 41.4, 40.8, 36.3, 36.2, 30.9, 25.1, 21.7 ppm; **IR** (ν_{max}, cm^{-1}) 3276 (w), 2928 (w), 1701 (m), 1184 (s), 1092 (s), 815 (m), 663 (m), 550 (s); **HRMS** (ESI/QTOF) m/z: $[M + Na]^+$ Calcd for $C_{15}H_{21}NNaO_3S^+$ 318.1134; Found 318.1136.

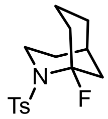

9

1-Fluoro-2-tosyl-2-azabicyclo[3.3.1]nonane (9): To a 10-mL vial charged with substrate **7** (0.05 mmol, 1.0 equiv), Pd(hfacac)$_2$ (1.3 mg, 2.5 μmol, 5 mol%) and Selectfluor (35.4 mg, 0.1 mmol, 2.0 equiv) was added anhydrous acetonitrile (preheated to 60 °C, 1.0 mL, *c* = 0.05 mol/L) under N_2 atmosphere. The sealed vial was allowed to stir at 60 °C for 10 min. The reaction mixture was allowed to cool in the ice bath, diluted with diethyl ether, filtered through a pad of basic alumina column and rinsed with diethyl ether. The filtrate was concentrated to dryness to afford the corresponding pure product **9** (11.6 mg, 78%). **^{1}H NMR** (600 MHz, $CDCl_3$) δ 7.78 (d, *J* = 7.7 Hz, 2H), 7.27 (d, *J* = 8.1 Hz, 2H), 3.71 – 3.66 (m, 1H), 3.01 (dp, *J* = 12.1, 3.8 Hz, 1H), 2.93 (tt, *J* = 12.8, 5.1 Hz, 1H), 2.46 – 2.36 (m, 1H), 2.41 (s, 3H), 2.07 – 2.01 (m, 1H), 1.94 – 1.78 (m, 2H), 1.75 – 1.68 (m, 1H), 1.62 – 1.50 (m, 3H), 1.42 – 1.34 (m, 2H) ppm; **^{13}C NMR** (150 MHz, $CDCl_3$) δ 143.2, 138.3, 129.5 (2C), 127.6 (d, *J* = 3.2 Hz, 2C), 104.3 (d, *J* = 217.2 Hz), 41.5, 37.5 (d, *J* = 28.7 Hz), 37.1 (d, *J* = 17.7 Hz), 31.4 (d, *J* = 1.8 Hz), 27.3, 26.8 (d, *J* = 7.7 Hz), 21.6, 19.9 (d, *J* = 7.4 Hz) ppm; **^{19}F NMR** (377 MHz, $CDCl_3$) δ -105.37 ppm; **IR** (ν_{max}, cm^{-1}) 2933 (w), 1459 (w), 1337 (s), 1159 (s), 1090 (m), 1069 (m), 1032 (m), 941 (m), 879 (w), 814 (s), 709 (w), 678 (s), 633 (m); **HRMS** (ESI/QTOF) m/z: $[M + Na]^+$ Calcd for $C_{15}H_{20}FNNaO_2S^+$ 320.1091; Found 320.1090.

V. Mechanistic Studies

***β*-C elimination experiment**

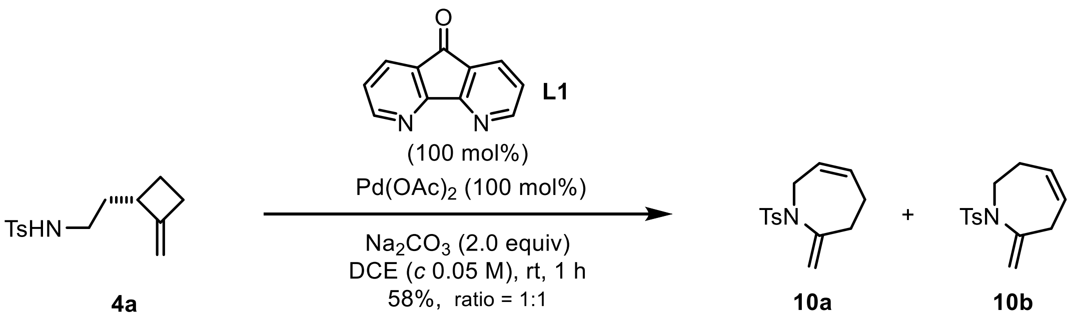

2-Methylene-1-tosyl-2,3,4,7-tetrahydro-1*H*-azepine and 2-methylene-1-tosyl-2,3,6,7-tetrahydro-1*H*-azepine (10a and 10b): Under N_2 atmosphere, to a 10-mL vial charged with substrate **4a** (13.3 mg, 0.05 mmol, 1.0 equiv), $Pd(OAc)_2$ (11.2 mg, 0.05 mmol, 1.0 equiv) and 4,5-diazafluoren-9-one **L1** (9.1 mg, 0.05 mmol, 1.0 equiv) was added anhydrous 1,2-dichloroethane (1.00 mL, *c* = 0.05 mol/L). The sealed vial was allowed to stir at room temperature for 1 h. TLC showed that the starting material was consumed completely, the reaction mixture was diluted with diethyl ether, filtered through a short pad of neutral alumina and rinsed with diethyl ether. The filtrate was concentrated to dryness at 25 °C. The residue was purified by flash column chromatography on neutral alumina (0% to 5% to 10% ethyl acetate-hexane) to give inseparable compound **10a** and **10b** as a colorless oil (7.6 mg, 58%, ratio = ~1:1). **^{1}H NMR** (500 MHz, $CDCl_3$) δ 7.71 (d, J = 8.4 Hz, 2H), 7.69 (d, J = 8.3 Hz, 2H), 7.28 (d, J = 8.1 Hz, 2H), 7.24 (d, J = 8.0 Hz, 2H), 5.72 – 5.60 (m, 2H), 5.59 – 5.53 (m, 1H), 5.52 – 5.45 (m, 1H), 5.14 (s, 1H), 5.09 (s, 1H), 4.81 (s, 1H), 4.65 (s, 1H), 4.27 – 4.23 (m, 2H), 3.75 – 3.71 (m, 2H), 2.65-2.62 (m, 2H), 2.46 – 2.40 (m, 4H), 2.42 (s, 3H), 2.41 (s, 3H), 1.88-1.84 (m, 2H) ppm; **^{13}C NMR** (125 MHz, $CDCl_3$) δ 145.2, 143.6, 143.5, 143.4, 136.7, 136.2, 132.6, 129.6 (2C), 129.2 (2C), 128.4, 128.2 (2C), 127.6 (2C), 126.6, 125.4, 110.6, 104.9, 47.1, 45.5, 34.3, 34.0, 28.5, 28.0, 21.7 (2C) ppm; **IR** (ν_{max}, cm^{-1}) 2926 (w), 1709 (w), 1333 (m), 1157 (s), 1121 (m), 815 (w), 662 (w), 549 (m); **HRMS** (nanochip-ESI/LTQ-Orbitrap) m/z: $[M + H]^+$ Calcd for $C_{14}H_{18}NO_2S^+$ 264.1053; Found 264.1052.

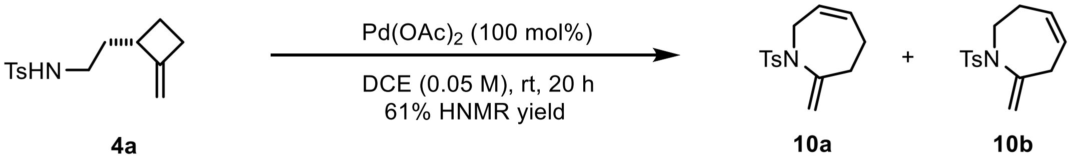

Under N_2 atmosphere, to a 10-mL vial charged with substrate **4a** (6.63 mg, 0.025 mmol, 1.0 equiv) and $Pd(OAc)_2$ (5.60 mg, 0.025 mmol, 1.0 equiv) was added anhydrous 1,2-dichloroethane (0.50 mL, *c* = 0.05 mol/L). The sealed vial was allowed to stir at room temperature for 20 h. TLC showed that the starting material was consumed completely, the reaction mixture was diluted with diethyl ether, filtered through a short pad of neutral alumina and rinsed with diethyl ether. The filtrate was concentrated to dryness at 25 °C. Inseparable compound **10a** and **10b**: 61% ^{1}H NMR yield (ratio = ~1:1).

Synthesis of Pd(II) complex

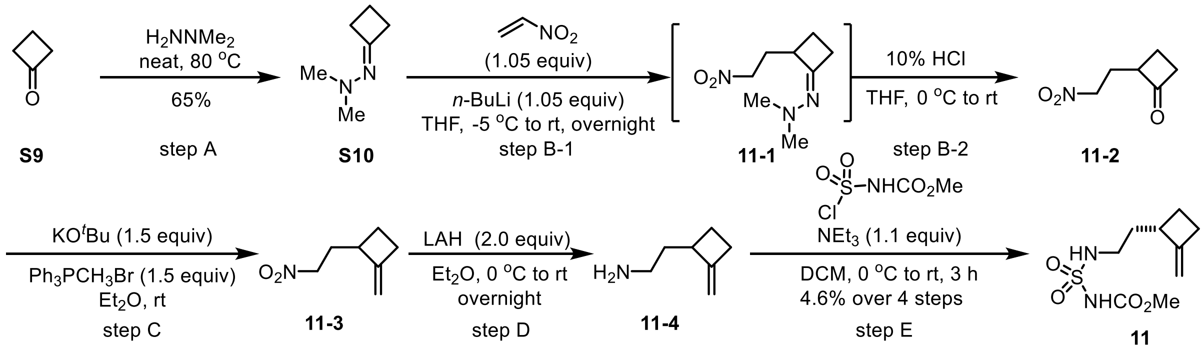

Step A: A round-bottom flask charged with the cyclobutanone **S9** (7.01 g, 100 mmol, 1.0 equiv) and *N*,*N*-dimethylhydrazine (24.0 g, 400 mmol, 4.0 equiv) was heated to 80 °C with refluxing condenser for 24 h. After cooling to room temperature, the reaction mixture was concentrated under vacuum at 30 °C to remove excess *N*,*N*-dimethylhydrazine. The resulting mixture was filtered through a short pad of anhydrous sodium sulfate and the filtrate was purified by distillation under reduced pressure (20 mbar, 80 °C) to afford the title compound **S10** as a colorless oil (7.29 g, 65%).

Step B: The corresponding hydrazone **S10** (5.61 g, 50 mmol, 1.0 equiv) was dissolved in anhydrous tetrahydrofuran (100 mL, c = 0.50 mol/L) and the resulting mixture was cooled to -5 °C, then *n*-butyllithium (2.5 M in hexane, 21.0 mL, 52.5 mmol, 1.05 equiv) was added dropwise. The resulting mixture was stirred for 1 h at -5 °C and a solution of nitroethylene[10] (2.0 M in toluene, 26.3 mL, 52.5 mmol, 1.05 equiv) was added slowly. After stirring at -5 °C for 1 h, the reaction mixture was allowed to stir overnight at room temperature to yield compound **11-1**, which was hydrolyzed with a 10% aqueous solution of HCl (100 mL) at 0 °C and stirred for 3 h at room temperature. The resulting mixture was extracted with diethyl ether (4×50 mL). The combined organic layers were washed with brine (2×60 mL), dried over anhydrous sodium sulfate, filtered and concentrated under vacuum at 10 °C to afford the crude compound **11-2** in toluene.

Step C: Potassium *tert*-butoxide (8.41 g, 75 mmol, 1.5 equiv) was added to Ph_3PCH_3Br (26.8 g, 75 mmol, 1.5 equiv) in anhydrous diethyl ether (100 mL) at room temperature. The reaction mixture was stirred at room temperature for 2 h. Then the resulting solution was transferred to a round-bottom flask charged with the above crude **11-2** (1.0 equiv) at room temperature. The reaction mixture was stirred overnight at room temperature. TLC showed that the starting material was consumed completely, then the reaction mixture was quenched by the addition of saturated ammonium chloride. The resulting mixture was extracted with diethyl ether (3×50 mL). The combined organic layers were washed with brine (2×60 mL), dried over anhydrous

sodium sulfate, filtered and concentrated under vacuum at 10 ^{o}C. The residue was purified by flash column chromatography on silica gel (0% to 1% diethyl ether-pentane) to give compound **11-3** (1.5 g).

Step D: To a stirred solution of **11-3** (1.5 g, 10.6 mmol, 1.0 equiv) in anhydrous diethyl ether (50 mL, *c* = 0.21 mol/L) was added lithium aluminum hydride (807 mg, 21.3 mmol, 2.0 equiv) in 4 portions at 0 ^{o}C. The reaction mixture was allowed to stir overnight at room temperature. TLC showed that the starting material was consumed completely, then the reaction mixture was cooled to 0 ^{o}C and carefully quenched by the sequential addition of water (0.81 mL), 15% aqueous sodium hydroxide (0.81 mL) and water (3×0.81 mL). The resulting mixture was allowed to warm to room temperature and stirred for 30 min, then anhydrous sodium sulfate was added and the mixture was stirred for additional 30 min. The mixture was filtered through a short pad of Celite and rinsed with diethyl ether. The filtrate was concentrated under vacuum at 10 ^{o}C to yield crude amine **11-4** (1.07 g, ~50% purity).

Step E: To a stirred solution of **11-4** [267 mg (~50% purity), ~1.2 mmol, 1.0 equiv] in anhydrous dichloromethane (6 mL, *c* = 0.20 mol/L) was added triethylamine (183 µL, 1.32 mmol, 1.1 equiv) at 0 ^{o}C, followed by dropwise addition of methyl *N*-(chlorosulfonyl)carbamate[11] (208 mg, 1.2 mmol, 1.0 equiv) in anhydrous dichloromethane (3 mL) over a 45 min period. The reaction mixture was allowed to stir at room temperature for 3 h. The reaction mixture was quenched by the addition of water (10 mL). The resulting mixture was extracted with ethyl acetate (3×20 mL). The combined organic layers were washed with brine (2×20 mL), dried over anhydrous sodium sulfate, filtered and concentrated under vacuum. The residue was purified by flash column chromatography on silica gel (0% to 10% ethyl acetate-hexane) to give compound **11** as a white solid (143 mg, 4.6% yield over 4 steps).

Methyl (*N*-(2-(2-methylenecyclobutyl)ethyl)sulfamoyl)carbamate (11): **^{1}H NMR** (400 MHz, $CDCl_3$) δ 8.12 (s, 1H), 5.54 (t, *J* = 6.1 Hz, 1H), 4.73-4.70 (m, 2H), 3.79 (s, 3H), 3.09 (ddt, *J* = 12.7, 9.9, 6.1 Hz, 2H), 3.02 – 2.91 (m, 1H), 2.68 – 2.50 (m, 2H), 2.10 (dtd, *J* = 10.6, 9.0, 5.0 Hz, 1H), 1.87 (dq, *J* = 13.8, 7.0 Hz, 1H), 1.68 (dt, *J* = 14.3, 6.8 Hz, 1H), 1.63 – 1.52 (m, 1H) ppm; **^{13}C NMR** (100 MHz, $CDCl_3$) δ 153.5, 152.3, 104.3, 53.7, 41.8, 41.6, 33.5, 29.3, 23.5 ppm; **IR** (ν_{max}, cm^{-1}) 3276 (m), 2942 (w), 1725 (s), 1468 (s), 1352 (m), 1242 (m), 1159 (s), 1076 (w), 877 (w), 593 (m); **HRMS** (ESI/QTOF) m/z: $[M + Na]^+$ Calcd for $C_9H_{16}N_2NaO_4S^+$ 271.0723; Found 271.0725.

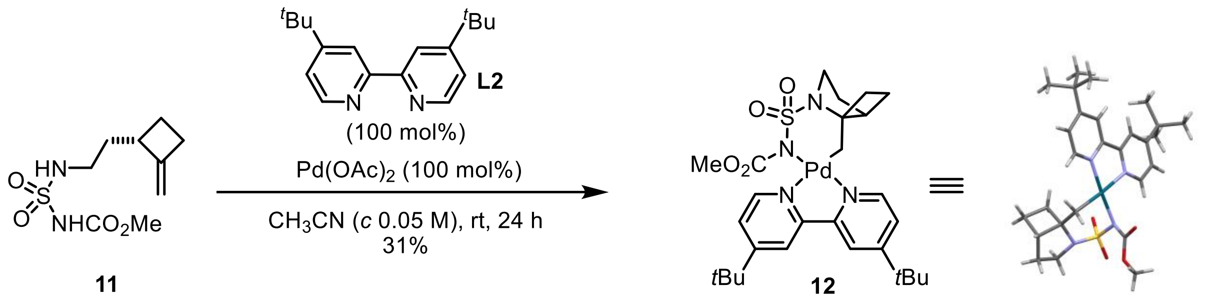

X-ray
CCDC: 2417054

Pd complex (12): To a 10-mL round-bottomed flask charged with substrate **11** (49.7 mg, 0.20 mmol, 1.0 equiv), palladium acetate (44.9 mg, 0.20 mmol, 1.0 equiv) and 4,4'-di-*tert*-butyl-2,2'-bipyridine **L2** (53.7 mg, 0.20 mmol, 1.0 equiv) was added anhydrous acetonitrile (4 mL, c = 0.05 mol/L). The mixture was stirred at room temperature under N_2 atmosphere for 24 h. The mixture was diluted with ethyl acetate (10 mL), filtered through a short pad of silica gel and rinsed with ethyl acetate. The filtrate was concentrated to dryness at 25 °C. The residue was purified by flash column chromatography on silica gel (0% to 50% to 90% ethyl acetate-hexane) to afford the desired Pd(II) complex **12** as a pale yellow foam (39 mg, 31%). Recrystallization of Pd(II) complex **12** from dichloromethane/diethyl acetate/hexane (v/v/v, 1:1:1); **^{1}H NMR** (600 MHz, $CDCl_3$) δ 8.66 (brs, 2H), 7.96 (d, J = 2.1 Hz, 1H), 7.89 (d, J = 1.8 Hz, 1H), 7.50 (dd, J = 5.8, 1.9 Hz, 1H), 7.47 (dd, J = 5.8, 1.9 Hz, 1H), 3.81 (ddd, J = 9.3, 7.7, 4.0 Hz, 1H), 3.66 (s, 3H), 3.69 – 3.62 (m, 1H), 2.59 (brs, 2H), 2.15 – 1.92 (m, 4H), 1.82 (brs, 1H), 1.59 (brs, 1H), 1.52 – 1.44 (m, 1H), 1.42 (s, 9H), 1.38 (s, 9H) ppm; **^{13}C NMR** (150 MHz, $CDCl_3$) δ 163.9, 163.4, 159.1, 157.5, 152.7, 149.2, 149.1, 124.0, 123.3, 119.1, 117.7, 74.5, 52.8, 48.6, 47.5, 35.63, 35.57, 30.6, 30.5 (3C), 30.4 (3C), 20.7 ppm; *Note:* "*Two CH_2 carbon signals were not resolvable due to their anticipated weak intensity of broaden peaks, which correspond to 2.59 (brs, 1H), 2.15 – 1.92 (m, 2H), 1.82 (brs, 1H).*" **IR** (ν_{max}, cm^{-1}) 2958 (m), 1688 (m), 1615 (m), 1434 (m), 1307 (m), 1271 (s), 1252 (s), 1148 (s), 1093 (m), 889 (w), 602 (w), 582 (w); **HRMS** (ESI/QTOF) m/z: $[M + H]^+$ Calcd for $C_{27}H_{39}N_4O_4PdS^+$ 621.1721; Found 621.1747.

Table S3: Oxidative transformation of Pd(II) complex 12

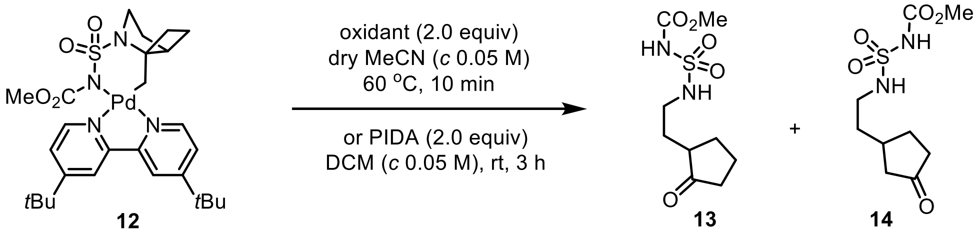

Entry	oxidant	Solvent	Temp	Time	Yield of **13**	Yield of **14**
1	SelectFluor	MeCN	60 °C	10 min	29%	trace
2	NFSI	MeCN	60 °C	10 min	27%	trace
3	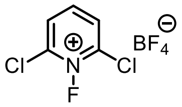	MeCN	60 °C	10 min	53%	trace
4	PIDA	DCM	rt	3 h	28%	trace

Experiment procedure of Entry 1 to 3: To a 10-mL vial charged with Pd(II) complex **12** (12.4 mg, 0.02 mmol, 1.0 equiv) and oxidant (2.0 equiv) was added anhydrous acetonitrile (preheated to 60 °C, 0.4 mL, c = 0.05 mol/L) under N_2 atmosphere. The sealed vial was allowed to stir at 60 °C for 10 min. The reaction mixture was allowed to cool in the ice bath, diluted with ethyl acetate, filtered through a short pad of silica gel and rinsed with ethyl acetate. The filtrate was concentrated to dryness and the residue was purified by preparative thin layer chromatography to afford compounds **13** and **14**.

Experiment procedure of Entry 4**:** To a 10-mL vial charged with Pd(II) complex **12** (7.5 mg, 0.012 mmol, 1.0 equiv) and iodobenzene diacetate (7.8 mg, 0.024 mmol, 2.0 equiv) was added anhydrous dichloromethane (0.24 mL, c = 0.05 mol/L) under N_2 atmosphere. The sealed vial was allowed to stir at room temperature for 3 h. The reaction mixture was diluted with ethyl acetate, filtered through a short pad of silica gel and rinsed with ethyl acetate. The filtrate was concentrated to dryness and the residue was purified by preparative thin layer chromatography to afford compound **13** (0.9 mg, 28%) as colorless oil and trace amount of compound **14**.

Direct oxidation of sulfonyl carbamate substrate 11:

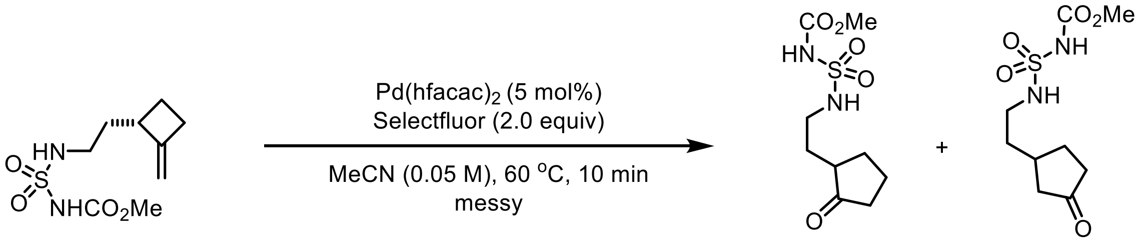

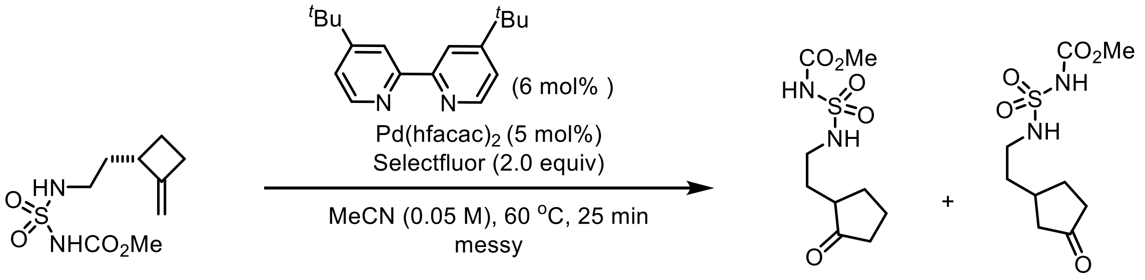

To a 10-mL vial charged with substrate **11** (0.05 mmol, 1.0 equiv), Pd(hfacac)$_2$ (1.3 mg, 2.5 μmol, 5 mol%), Selectfluor (35.4 mg, 0.10 mmol, 2.0 equiv) and 4,4'-di-tert-butyl-2,2'-bipyridine (0.8 mg, 3.0 μmol, 6 mol%) was added anhydrous acetonitrile (preheated to 60 °C, 1.0 mL, c = 0.05 mol/L) under N_2 atmosphere. The sealed vial was allowed to stir at 60 °C for a certain time (without ligand: 10 min; with ligand: 25 min). The reaction mixture was allowed to cool in the ice bath, diluted with ethyl acetate, filtered through a short pad of silica gel and rinsed with ethyl acetate. The filtrate was concentrated to dryness at 25 °C and the residue was purified by preparative thin layer chromatography to afford the desired products **13** and **14**.

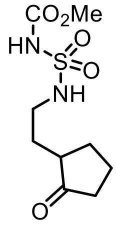

13

Methyl (*N*-(2-(2-oxocyclopentyl)ethyl)sulfamoyl)carbamate (13): **^{1}H NMR** (400 MHz, $CDCl_3$) δ 7.42 (s, 1H), 5.59 (t, J = 6.2 Hz, 1H), 3.80 (s, 3H), 3.31 – 3.13 (m, 2H), 2.40 – 2.23 (m, 2H), 2.23 – 2.09 (m, 2H), 2.04 (dddt, J = 13.3, 8.9, 6.5, 2.2 Hz, 1H), 1.94 (dq, J = 14.0, 6.9 Hz, 1H), 1.82 (dtdd, J = 12.6, 10.8, 8.4, 6.2 Hz, 1H), 1.67 – 1.49 (m, 2H) ppm; **^{13}C NMR** (150 MHz, $CDCl_3$) δ 221.1, 151.9, 53.8, 47.1, 42.6, 38.0, 30.0, 29.2, 20.9 ppm; **IR** (ν_{max}, cm^{-1}) 3256 (w), 2960 (w), 2875 (w), 1722 (s), 1468 (m), 1350 (m), 1158 (s), 1079 (w), 870 (w), 587 (m);

HRMS (nanochip-ESI/LTQ-Orbitrap) m/z: $[M + Na]^+$ Calcd for $C_9H_{16}N_2NaO_5S^+$ 287.0672; Found 287.0672.

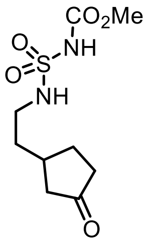

14

Methyl (*N*-(2-(3-oxocyclopentyl)ethyl)sulfamoyl)carbamate (14): **^{1}H NMR** (500 MHz, $CDCl_3$) δ 7.32 (s, 1H), 5.16 (t, J = 6.3 Hz, 1H), 3.81 (s, 3H), 3.15 (qd, J = 7.0, 2.4 Hz, 2H), 2.43 (dd, J = 18.1, 7.3 Hz, 1H), 2.37 – 2.25 (m, 2H), 2.25 – 2.13 (m, 2H), 1.82 (ddd, J = 18.0, 10.4, 1.4 Hz, 1H), 1.75 (qd, J = 7.0, 2.8 Hz, 2H), 1.60 – 1.50 (m, 1H) ppm; **^{13}C NMR** (125 MHz, $CDCl_3$) δ 218.4, 152.0, 53.8, 44.8, 42.6, 38.5, 35.1, 34.4, 29.4 ppm; **IR** (ν_{max}, cm^{-1}) 2954 (w), 2924 (s), 2854 (m), 1732 (m), 1464 (w), 1160 (m), 963 (w); **HRMS** (ESI/QTOF) m/z: $[M + Na]^+$ Calcd for $C_9H_{16}N_2NaO_5S^+$ 287.0672; Found 287.0675.

^{13}C labeling experiment

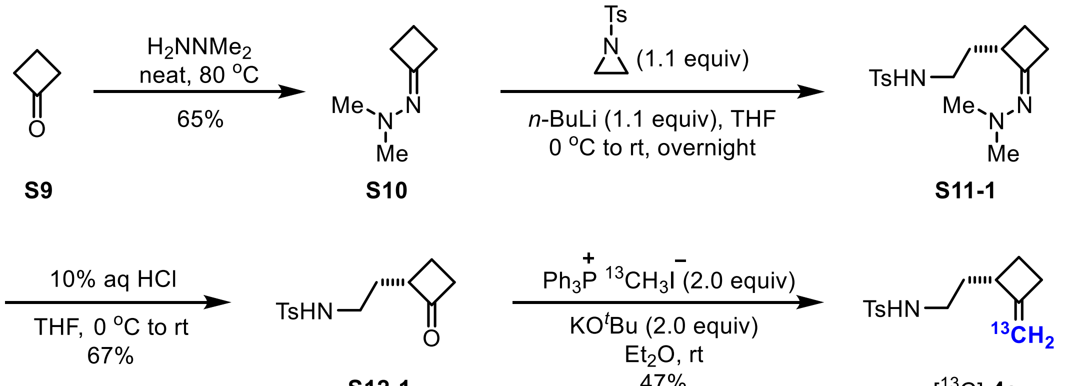

4-Methyl-*N*-(2-(2-(methylene-^{13}C)cyclobutyl)ethyl)benzenesulfonamide {[^{13}C]-4a}: A round-bottom flask charged with the cyclobutanone **S9** (7.01 g, 100 mmol, 1.0 equiv) and *N*,*N*-dimethylhydrazine (24.0 g, 400 mmol, 4.0 equiv) was heated to 80 °C with refluxing condenser for 24 h. After cooling to room temperature, the reaction mixture was concentrated under vacuum at 30 °C to remove excess *N*,*N*-dimethylhydrazine. The resulting mixture was filtered through a short pad of anhydrous sodium sulfate and the filtrate was purified by distillation

under reduced pressure (20 mbar, 80 °C) to afford the title compound **S10** as a colorless oil (7.29 g, 65%).

The corresponding hydrazone **S10** (1.57 g, 14 mmol, 1.0 equiv) was dissolved in anhydrous tetrahydrofuran (56 mL, c = 0.25 mol/L) and the resulting mixture was cooled to 0 °C, then *n*-butyllithium (2.5 M in hexane, 6.16 mL, 15.4 mmol, 1.1 equiv) was added dropwise. The resulting mixture was stirred for 1 h at 0 °C and a solution of *N*-tosylaziridine (3.04 g, 15.4 mmol, 1.1 equiv) in anhydrous THF (10 mL) was added slowly. After stirring at 0 °C for 1 h, the reaction mixture was allowed to stir overnight at room temperature to yield compound **S11-1**. A 10% aqueous solution of HCl (66 mL) was added at 0 °C and the mixture was stirred overnight at room temperature. The resulting mixture was extracted with ethyl acetate (4×30 mL). The combined organic layers were washed with brine (2×30 mL), dried over anhydrous sodium sulfate, filtered and concentrated under vacuum. The residue was purified by flash column chromatography on silica gel (0% to 20% ethyl acetate-hexane) to give compound **S12-1** as a pale yellow oil (2.5 g, 67%).

Potassium *tert*-butoxide (112 mg, 1.0 mmol, 2.0 equiv) was added to $Ph_3P^{13}CH_3I$ (405 mg, 1.0 mmol, 2.0 equiv) in anhydrous diethyl ether (5 mL, c = 0.1 mol/L) at room temperature. The reaction mixture was stirred at room temperature for 2 h. Then the resulting solution was transferred to a round-bottom flask charged with compound **S12-1** (134 mg, 0.5 mmol, 1.0 equiv) at room temperature. The reaction mixture was stirred overnight at room temperature. TLC showed that the starting material was consumed completely, then the reaction mixture was quenched by the addition of saturated ammonium chloride. The resulting mixture was extracted with ethyl acetate (3×10 mL). The combined organic layers were washed with brine (2×10 mL), dried over anhydrous sodium sulfate, filtered and concentrated under vacuum. The residue was purified by flash column chromatography on silica gel (0% to 10% ethyl acetate-hexane) to give compound [^{13}C]-**4a** as a colorless oil (62.6 mg, 47%). **^{1}H NMR** (400 MHz, $CDCl_3$) δ 7.75 (d, J = 8.3 Hz, 2H), 7.30 (d, J = 8.1 Hz, 2H), 4.86-4.80 (m, 1H, NH), 4.65 (dd, J = 154.5, 2.4 Hz, 1H), 4.62 (dd, J = 155.4, 2.4 Hz, 1H), 3.01 – 2.82 (m, 3H), 2.63 – 2.45 (m, 2H), 2.42 (s, 3H), 2.01 (qdd, J = 9.0, 5.0, 1.7 Hz, 1H), 1.80 – 1.70 (m, 1H), 1.62 – 1.44 (m, 2H) ppm; **^{13}C NMR** (100 MHz, $CDCl_3$) δ 153.6 (d, J = 72.5 Hz), 143.5, 137.0, 129.8 (2C), 127.2 (2C), 104.1, 41.7, 41.2, 34.0 (d, J = 1.5 Hz), 29.3, 23.5 (d, J = 6.1 Hz), 21.6 ppm; **IR** (ν_{max}, cm^{-1}) 3279 (w), 2925 (w), 1323 (m), 1156 (s), 1093 (m), 867 (m), 814 (m), 663 (m), 551 (m); **HRMS** (nanochip-ESI/LTQ-Orbitrap) m/z: $[M + H]^+$ Calcd for $C_{13}[_{13}C]H_{20}NO_2S^+$ 267.1243; Found 267.1243.

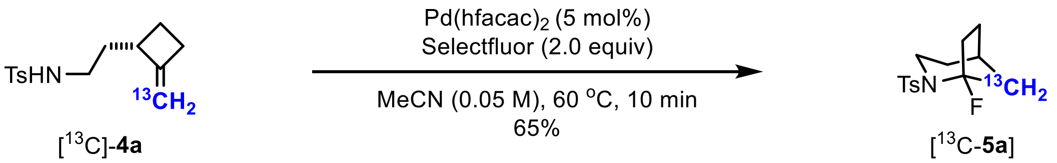

1-Fluoro-2-tosyl-2-azabicyclo[3.2.1]octane-8-^{13}C {[^{13}C]-5a}: To a 10-mL vial charged with substrate [^{13}C]-**4a** (26.6 mg, 0.10 mmol, 1.0 equiv), Pd(hfacac)$_2$ (2.6 mg, 0.005 mmol, 5 mol%) and Selectfluor (70.8 mg, 0.20 mmol, 2.0 equiv) was added anhydrous MeCN (preheated to 60 °C, 2.0 mL, c = 0.05 mol/L) under N_2 atmosphere. The sealed vial was allowed to stir at 60 °C for 10 min. The reaction mixture was allowed to cool in the ice bath, diluted with diethyl ether, filtered through a short pad of silica gel and rinsed with diethyl ether. The filtrate was concentrated to dryness at 25 °C and the residue was purified by preparative thin layer chromatography (20% ethyl acetate-hexane) to afford the desired product [^{13}C]-**5a** as a white solid (18.5 mg, 65%). **^{1}H NMR** (500 MHz, $CDCl_3$) δ 7.75 (d, J = 8.0 Hz, 2H), 7.27 (d, J = 8.1 Hz, 2H), 3.98 (dddd, J = 13.5, 6.8, 4.4, 2.9 Hz, 1H), 3.18 (ddd, J = 13.2, 10.8, 4.9 Hz, 1H), 2.47 – 2.41 (m, 1H), 2.41 (s, 3H), 2.06 – 1.95 (m, 3H), 1.90-1.81 (m, 1H), 1.79 – 1.70 (m, 2H), 1.53 – 1.45 (m, 1H), 1.45 – 1.37 (m, 1H) ppm; **^{13}C NMR** (150 MHz, $CDCl_3$) δ 143.2, 139.1, 129.5 (2C), 127.5 (d, J = 2.1 Hz, 2C), 111.3 (dd, J = 221.3, 40.4 Hz), 42.8, 41.9 (d, J = 18.3 Hz), 33.4 (dd, J = 31.0, 7.2 Hz), 33.2 (dd, J = 22.7, 6.6 Hz), 31.2, 27.0 (d, J = 7.2 Hz), 21.7 ppm; **^{19}F NMR** (377 MHz, $CDCl_3$) δ -137.26 (d, J = 18.5 Hz); **IR** (ν_{max}, cm^{-1}) 2946 (w), 2876 (w), 1333 (m), 1318 (m), 1156 (s), 1129 (m), 1090 (m), 815 (m), 683 (s), 546 (s); **HRMS** (Sicrit plasma/LTQ-Orbitrap) m/z: [M + H]$^+$ Calcd for $C_{13}[_{13}C]H_{19}FNO_2S^+$ 285.1149; Found 285.1148.

VI. Synthetic transformation

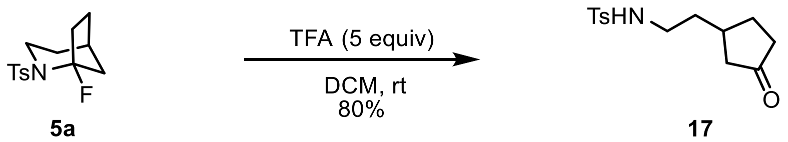

4-Methyl-*N*-(2-(3-oxocyclopentyl)ethyl)benzenesulfonamide (17): To a stirred solution of compound **5a** (14.2 mg, 0.05 mmol, 1.0 equiv) in anhydrous dichloromethane (1.0 mL, c = 0.05 mol/L) was added trifluoroacetic acid (19.3 μL, 0.25 mmol, 5.0 equiv) at room

temperature under N_2. The mixture was allowed to stir at room temperature for 10 h. TLC showed that the starting material was consumed completely, then the reaction mixture was quenched by the addition of saturated sodium bicarbonate. The resulting mixture was extracted with ethyl acetate (3×10 mL). The combined organic layers were washed with brine (1×10 mL), dried over anhydrous sodium sulfate, filtered and concentrated under vacuum. The residue was purified by preparative thin layer chromatography (50% ethyl acetate-hexane) to give compound **17** as a colorless oil (11.2 mg, 80%). **^{1}H NMR** (600 MHz, $CDCl_3$) δ 7.74 (d, J = 8.3 Hz, 2H), 7.31 (d, J = 8.0 Hz, 2H), 4.80 (t, J = 6.2 Hz, 1H), 3.02 – 2.93 (m, 2H), 2.43 (s, 3H), 2.32 (dd, J = 18.2, 7.6 Hz, 1H), 2.26 (dd, J = 16.2, 8.4 Hz, 1H), 2.22-2.17 (m, 1H), 2.14 – 2.06 (m, 2H), 1.72 (ddd, J = 18.0, 10.4, 1.4 Hz, 1H), 1.66 – 1.56 (m, 2H), 1.50 – 1.40 (m, 1H) ppm; **^{13}C NMR** (150 MHz, $CDCl_3$) δ 218.9, 143.7, 136.8, 129.9 (2C), 127.2 (2C), 44.8, 41.7, 38.5, 35.5, 34.4, 29.3, 21.7 ppm; **IR** (ν_{max}, cm^{-1}) 3280 (w), 2928 (w), 1735 (m), 1324 (m), 1156 (s), 1093 (m), 816 (w), 663 (m), 551 (m); **HRMS** (ESI/QTOF) m/z: $[M + Na]^+$ Calcd for $C_{14}H_{19}NNaO_3S^+$ 304.0978; Found 304.0987.

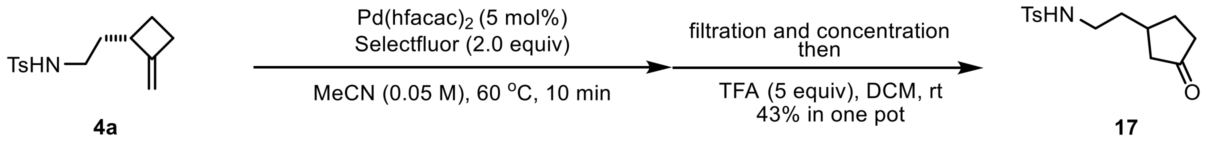

Under N_2 atmosphere, to a 10-mL vial charged with substrate **4a** (26.5 mg, 0.10 mmol, 1.0 equiv), $Pd(hfacac)_2$ (2.6 mg, 0.005 mmol, 5 mol%) and Selectfluor (70.8 mg, 0.20 mmol, 2.0 equiv) was added anhydrous MeCN (preheated to 60 °C, 2.0 mL, c = 0.05 mol/L). The sealed vial was allowed to stir at 60 °C for 10 minutes. The reaction mixture was allowed to cool in the ice bath, diluted with diethyl ether, filtered through a short pad of silica gel and rinsed with diethyl ether. The filtrate was concentrated to dryness at 25 °C and the residue was re-dissolved in anhydrous dichloromethane (2.0 mL, c = 0.05 mol/L). Trifluoroacetic acid (38.5 µL, 0.50 mmol, 5.0 equiv) was added to the above mixture at room temperature under N_2. The mixture was allowed to stir at room temperature for 16 h. TLC showed that the starting material was consumed completely, then the reaction mixture was quenched by the addition of saturated sodium bicarbonate. The resulting mixture was extracted with ethyl acetate (3×10 mL). The combined organic layers were washed with brine (1×10 mL), dried over anhydrous sodium sulfate, filtered and concentrated under vacuum. The residue was purified by preparative thin

layer chromatography (50% ethyl acetate-hexane) to give compound **17** as a colorless oil (12.1 mg, 43%).

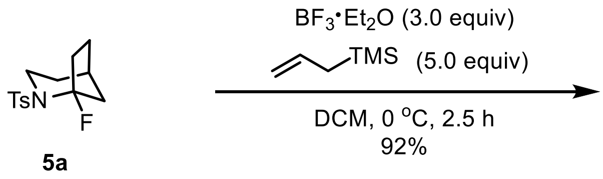

1-Allyl-2-tosyl-2-azabicyclo[3.2.1]octane (18): To a stirred solution of compound **5a** (70.8 mg, 0.25 mmol, 1.0 equiv) in anhydrous dichloromethane (5.0 mL, *c* = 0.05 mol/L) was added allyltrimethylsilane (199 µL, 1.25 mmol, 5.0 equiv) at 0 °C under N_2, followed by the addition of boron trifluoride diethyl etherate (93.0 µL, 0.75 mmol, 3.0 equiv). The mixture was allowed to stir at 0 °C for 2.5 h. TLC showed that the starting material was consumed completely, then the reaction mixture was quenched by the addition of saturated sodium bicarbonate. The resulting mixture was extracted with ethyl acetate (3×15 mL). The combined organic layers were washed with brine (2×10 mL), dried over anhydrous sodium sulfate, filtered and concentrated under vacuum. The residue was purified by flash column chromatography on silica gel (0% to 5% ethyl acetate-hexane) to give compound **18** as a colorless oil (70.0 mg, 92%). **^{1}H NMR** (600 MHz, $CDCl_3$) δ 7.72 (d, *J* = 8.3 Hz, 2H), 7.28 (d, *J* = 8.1 Hz, 2H), 5.83 (ddt, *J* = 17.2, 10.3, 7.0 Hz, 1H), 5.06 – 5.00 (m, 2H), 3.67 (ddd, *J* = 13.8, 6.1, 4.6 Hz, 1H), 3.16 (ddd, *J* = 14.1, 9.2, 5.3 Hz, 1H), 2.93 (ddt, *J* = 14.3, 6.7, 1.4 Hz, 1H), 2.67 (ddt, *J* = 14.2, 7.4, 1.2 Hz, 1H), 2.42 (s, 3H), 2.33 – 2.28 (m, 1H), 1.88 – 1.71 (m, 4H), 1.53 (ddd, *J* = 13.7, 11.8, 5.1 Hz, 1H), 1.43 – 1.36 (m, 2H), 1.32 (ddd, *J* = 12.2, 5.2, 1.8 Hz, 1H) ppm; **^{13}C NMR** (150 MHz, $CDCl_3$) δ 142.9, 139.3, 135.3, 129.6 (2C), 127.5 (2C), 117.7, 70.7, 42.6, 42.3, 42.1, 34.7, 33.3, 32.2, 30.3, 21.6 ppm; **IR** (ν_{max}, cm^{-1}) 2931 (w), 2869 (w), 1340 (m), 1322 (m), 1151 (s), 1090 (m), 815 (m), 675 (s), 546 (s); **HRMS** (ESI/QTOF) m/z: $[M + H]^+$ Calcd for $C_{17}H_{24}NO_2S^+$ 306.1522; Found 306.1521.

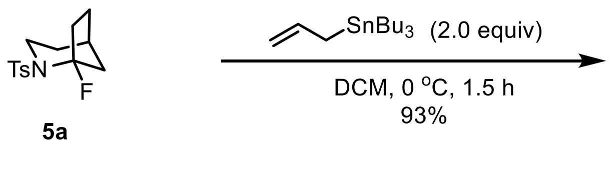

1-Allyl-2-tosyl-2-azabicyclo[3.2.1]octane (18): To a stirred solution of compound **5a** (14.2 mg, 0.05 mmol, 1.0 equiv) in anhydrous dichloromethane (1.0 mL, *c* = 0.05 mol/L) was added

allyltributylstannane (31.0 µL, 0.10 mmol, 2.0 equiv) at 0 °C under N_2, followed by the addition of boron trifluoride diethyl etherate (18.5 µL, 0.15 mmol, 3.0 equiv). The mixture was allowed to stir at 0 °C for 1.5 h. TLC showed that the starting material was consumed completely, then the reaction mixture was quenched by the addition of saturated sodium bicarbonate. The resulting mixture was extracted with ethyl acetate (3×3 mL). The combined organic layers were washed with brine (2×3 mL), dried over anhydrous sodium sulfate, filtered and concentrated under vacuum. The residue was purified twice by preparative thin layer chromatography (15% ethyl acetate-hexane) to give compound **18** as a colorless oil (14.2 mg, 93%).

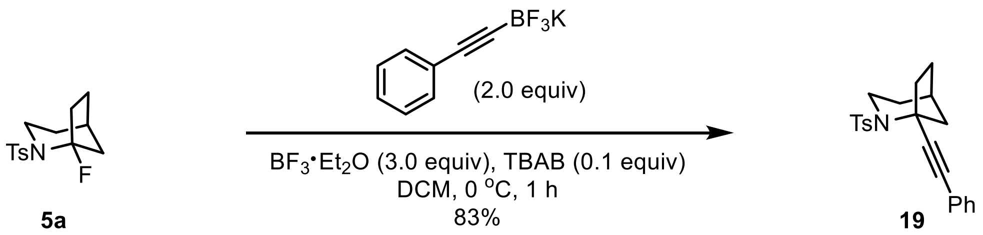

1-(Phenylethynyl)-2-tosyl-2-azabicyclo[3.2.1]octane (19): To a stirred solution of compound **5a** (28.3 mg, 0.1 mmol, 1.0 equiv) in anhydrous dichloromethane (2.0 mL, *c* = 0.05 mol/L) was added potassium trifluoro(phenylethynyl)borate (41.6 mg, 0.20 mmol, 2.0 equiv) and tetrabutylammonium bromide (3.2 mg, 0.01 mmol, 0.1 equiv) at 0 °C under N_2, followed by the addition of boron trifluoride diethyl etherate (37.0 µL, 0.30 mmol, 3.0 equiv). The mixture was allowed to stir at 0 °C for 1.0 h. TLC showed that the starting material was consumed completely, then the reaction mixture was quenched by the addition of saturated sodium bicarbonate. The resulting mixture was extracted with ethyl acetate (3×5 mL). The combined organic layers were washed with brine (2×5 mL), dried over anhydrous sodium sulfate, filtered and concentrated under vacuum. The residue was purified by preparative thin layer chromatography (20% ethyl acetate-hexane) to give compound **19** as a colorless oil (30.4 mg, 83%), which turned into a white solid in the freezer. **^{1}H NMR** (600 MHz, $CDCl_3$) δ 7.77 (d, J = 8.3 Hz, 2H), 7.30 – 7.21 (m, 5H), 7.17 (d, J = 7.8 Hz, 2H), 4.10 (ddd, J = 13.1, 6.1, 2.1 Hz, 1H), 3.11 (ddd, J = 13.2, 11.9, 4.4 Hz, 1H), 2.48-2.45 (m, 1H), 2.36 (s, 3H), 2.14-2.10 (m, 1H), 2.08 (ddd, J = 9.0, 5.2, 2.2 Hz, 1H), 2.03 (ddd, J = 14.2, 12.4, 4.7 Hz, 1H), 1.93 (tdd, J = 12.5, 6.5, 1.4 Hz, 1H), 1.84 (ddd, J = 11.7, 5.1, 2.1 Hz, 1H), 1.80 (dddd, J = 11.9, 6.2, 2.8, 1.3 Hz, 1H), 1.58 – 1.53 (m, 1H), 1.50 (dtt, J = 12.8, 4.2, 2.1 Hz, 1H) ppm; **^{13}C NMR** (150 MHz, $CDCl_3$) δ 142.7, 139.1, 131.7 (2C), 129.3 (2C), 128.2, 128.1 (2C), 127.7 (2C), 123.2, 90.4, 85.8, 61.0, 47.6, 41.6, 39.0, 34.7, 31.4, 29.1, 21.6 ppm; **IR** (ν_{max}, cm^{-1}) 2937 (w), 1491 (w),

1343 (s), 1154 (s), 1091 (m), 983 (m), 758 (s), 707 (s), 692 (s), 556 (m), 544 (s); **HRMS** (ESI/QTOF) m/z: $[M + Na]^+$ Calcd for $C_{22}H_{23}NNaO_2S^+$ 388.1342; Found 388.1344.

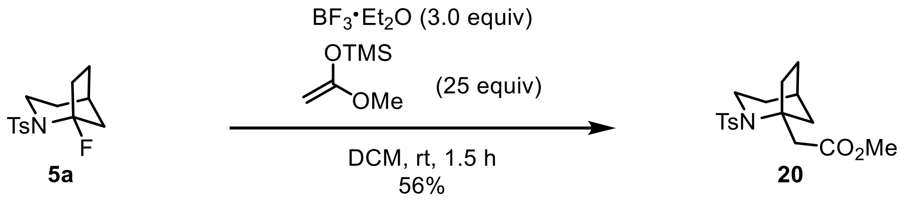

Methyl 2-(2-tosyl-2-azabicyclo[3.2.1]octan-1-yl)acetate (20): To a stirred solution of compound **5a** (14.2 mg, 0.05 mmol, 1.0 equiv) in anhydrous dichloromethane (1.0 mL, c = 0.05 mol/L) was added [(1-methoxyvinyl)oxy](trimethyl)silane (146.3 mg, 1.0 mmol, 20 equiv) at room temperature under N_2, followed by the addition of boron trifluoride diethyl etherate (18.5 µL, 0.15 mmol, 3.0 equiv). The mixture was allowed to stir at room temperature for 1.5 h. The reaction mixture was quenched by the addition of saturated sodium bicarbonate. The resulting mixture was extracted with ethyl acetate (3×5 mL). The combined organic layers were washed with brine (2×5 mL), dried over anhydrous sodium sulfate, filtered and concentrated under vacuum. The residue was purified by preparative thin layer chromatography (eluted twice with 15% ethyl acetate-hexane) to give compound **20** as a colorless oil (9.4 mg, 56%). ^{1}**H NMR** (600 MHz, $CDCl_3$) δ 7.72 (d, J = 8.3 Hz, 2H), 7.27 (d, J = 8.2 Hz, 2H), 3.60 (s, 3H), 3.54 (dt, J = 13.1, 5.7 Hz, 1H), 3.25 (d, J = 16.9 Hz, 1H), 3.17 (ddd, J = 13.9, 8.4, 5.6 Hz, 1H), 2.94 (d, J = 16.8 Hz, 1H), 2.41 (s, 3H), 2.38 – 2.34 (m, 1H), 2.26 (dddd, J = 13.9, 9.1, 4.4, 2.4 Hz, 1H), 2.20 (dt, J = 12.1, 2.2 Hz, 1H), 1.90 – 1.82 (m, 1H), 1.81 – 1.74 (m, 1H), 1.63 – 1.55 (m, 1H), 1.47 – 1.40 (m, 2H), 1.32 (ddd, J = 12.2, 5.2, 1.7 Hz, 1H) ppm; ^{13}**C NMR** (150 MHz, $CDCl_3$) δ 171.5, 143.0, 138.7, 129.6 (2C), 127.5 (2C), 67.6, 51.4, 42.9, 42.8, 42.0, 36.8, 33.2, 32.2, 30.0, 21.6 ppm; **IR** (ν_{max}, cm^{-1}) 2946 (w), 1736 (s), 1338 (w), 1152 (s), 1089 (m), 816 (m), 678 (s), 547 (s); **HRMS** (ESI/QTOF) m/z: $[M + H]^+$ Calcd for $C_{17}H_{24}NO_4S^+$ 338.1421; Found 338.1422.

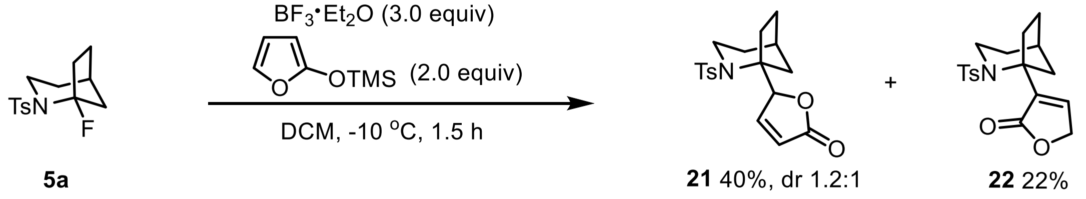

To a stirred solution of compound **5a** (14.2 mg, 0.05 mmol, 1.0 equiv) in anhydrous dichloromethane (1.0 mL, *c* = 0.05 mol/L) was added 2-(trimethylsilyloxy)furan (16.8 µL, 0.10 mmol, 2.0 equiv) at -10 °C under N_2, followed by the addition of boron trifluoride diethyl etherate (18.5 µL, 0.15 mmol, 3.0 equiv). The mixture was allowed to stir at -10 °C for 1.5 h. TLC showed that the starting material was consumed completely, then the reaction mixture was quenched by the addition of saturated sodium bicarbonate. The resulting mixture was extracted with ethyl acetate (3×5 mL). The combined organic layers were washed with brine (2×5 mL), dried over anhydrous sodium sulfate, filtered and concentrated under vacuum. The residue was purified by preparative thin layer chromatography (developed 3 times with 35% ethyl acetate-hexane) to give compound **21** as an inseparable mixture of 2 diastereoisomers as a colorless oil (6.9 mg, 40%, dr = 1.2:1) and compound **22** as a colorless oil (3.9 mg, 22%).

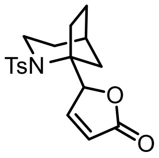

21

5-(2-tosyl-2-azabicyclo[3.2.1]octan-1-yl)furan-2(5*H*)-one (21): **^{1}H NMR** (600 MHz, $CDCl_3$) δ 7.93 (dd, *J* = 5.7, 1.4 Hz, 1H), 7.79 (d, *J* = 8.4 Hz, 2H), 7.75 – 7.71 (m, 3H), 7.37 – 7.33 (m, 4H), 6.27 (t, *J* = 1.7 Hz, 1H), 6.16 (dd, *J* = 3.6, 2.0 Hz, 1H), 6.15 (dd, *J* = 3.6, 2.0 Hz, 1H), 6.12 (t, *J* = 2.0 Hz, 1H), 3.62 (ddd, *J* = 15.2, 6.0, 4.4 Hz, 1H), 3.48 (dt, *J* = 13.2, 5.9 Hz, 1H), 3.26 (ddd, *J* = 15.0, 9.6, 5.2 Hz, 1H), 3.07 – 3.01 (m, 1H), 2.46 (s, 3H), 2.45 (s, 3H), 2.35-2.32 (m, 1H), 2.29-2.26 (m, 1H), 1.90 – 1.73 (m, 5H), 1.68 – 1.52 (m, 5H), 1.48 (ddd, *J* = 12.2, 5.3, 1.6 Hz, 1H), 1.45 – 1.38 (m, 2H), 1.37 – 1.30 (m, 2H), 1.19 – 1.13 (m, 1H) ppm; **^{13}C NMR** (150 MHz, $CDCl_3$) δ 173.21, 173.16, 157.3, 156.2, 144.1, 144.0, 138.3, 136.3, 130.12 (2C), 130.07 (2C), 127.9 (2C), 127.7 (2C), 122.4, 121.9, 83.6, 83.2, 72.0, 71.5, 43.1, 42.3, 36.9, 36.0, 33.4, 31.9, 31.4, 31.3, 31.0, 30.0, 29.8, 29.3, 21.7 (2C) ppm; **IR** (ν_{max}, cm^{-1}) 2941 (w), 1754 (s), 1342 (w), 1318 (m), 1152 (s), 1088 (s), 916 (m), 919 (s), 680 (s), 556 (s); **HRMS** (ESI/QTOF) m/z: $[M + H]^+$ Calcd for $C_{18}H_{22}NO_4S^+$ 348.1264; Found 348.1264.

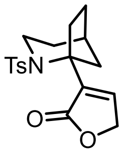

22

3-(2-Tosyl-2-azabicyclo[3.2.1]octan-1-yl)furan-2(5*H*)-one (22): **^{1}H NMR** (600 MHz, $CDCl_3$) *δ* 7.68 – 7.65 (m, 2H), 7.26 – 7.23 (m, 3H), 4.74 (d, *J* = 1.8 Hz, 2H), 3.63 (ddd, *J* = 14.1, 6.0, 3.6 Hz, 1H), 3.27 (ddd, *J* = 14.1, 10.2, 5.0 Hz, 1H), 2.59 (dddd, *J* = 14.1, 9.4, 4.4, 2.4 Hz, 1H), 2.54 – 2.49 (m, 2H), 2.40 (s, 3H), 1.96 – 1.87 (m, 2H), 1.82 (ddd, *J* = 14.3, 12.4, 4.8 Hz, 1H), 1.64 – 1.56 (m, 1H), 1.53 (ddd, *J* = 11.8, 5.3, 1.6 Hz, 1H), 1.46 (ddtd, *J* = 13.1, 5.1, 3.5, 1.5 Hz, 1H) ppm; **^{13}C NMR** (150 MHz, $CDCl_3$) *δ* 172.0, 145.1, 143.4, 138.0, 136.9, 129.5 (2C), 127.7 (2C), 69.8, 65.1, 41.9, 41.5, 36.1, 33.8, 31.7, 29.2, 21.7 ppm; **IR** (ν_{max}, cm^{-1}) 2934 (w), 1755 (s), 1343 (m), 1154 (s), 1068 (m), 819 (w), 679 (s), 556 (m), 544 (m); **HRMS** HRMS (ESI/QTOF) m/z: $[M + H]^+$ Calcd for $C_{18}H_{22}NO_4S^+$ 348.1264; Found 348.1261.

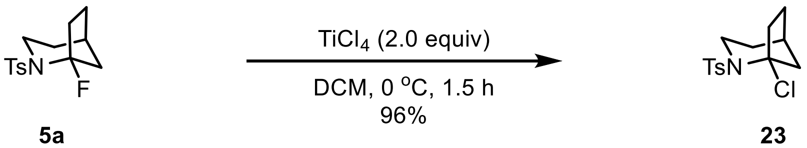

5a **23**

1-Chloro-2-tosyl-2-azabicyclo[3.2.1]octane (23): To a stirred solution of compound **5a** (14.2 mg, 0.05 mmol, 1.0 equiv) in anhydrous dichloromethane (1.0 mL, *c* = 0.05 mol/L) was added titanium tetrachloride (1.0 M in dichloromethane, 100 µL, 0.10 mmol, 2.0 equiv) at 0 °C under N_2, The mixture was allowed to stir at 0 °C for 1.5 h. TLC showed that the starting material was consumed completely, then the reaction mixture was quenched by the addition of saturated sodium bicarbonate. The resulting mixture was extracted with ethyl acetate (3×3 mL). The combined organic layers were washed with brine (2×3 mL), dried over anhydrous sodium sulfate, filtered and concentrated at ≤ 10 °C under vacuum. The residue was purified by flash column chromatography on neutral alumina (0% to 15% ethyl acetate-hexane) to give compound **23** as a colorless oil (14.4 mg, 96%). **^{1}H NMR** (600 MHz, $CDCl_3$) *δ* 7.80 (d, *J* = 8.3 Hz, 2H), 7.27 (d, *J* = 8.0 Hz, 2H), 3.46 (ddd, *J* = 13.9, 7.0, 3.9 Hz, 1H), 3.38 (ddd, *J* = 13.9, 8.9, 6.0 Hz, 1H), 3.01 (ddt, *J* = 13.9, 9.0, 2.9 Hz, 1H), 2.47 – 2.41 (m, 1H), 2.41 (s, 3H), 2.36 (dt, *J* = 12.2, 2.2 Hz, 1H), 2.19 – 2.12 (m, 1H), 2.03 (ddd, *J* = 13.5, 11.9, 6.7 Hz, 1H), 1.96 – 1.88 (m, 2H), 1.68 – 1.61 (m, 1H), 1.57 – 1.50 (m, 1H) ppm; **^{13}C NMR** (150 MHz, $CDCl_3$) *δ* 143.3, 138.8, 129.5 (2C), 127.6 (2C), 85.8, 46.0, 42.1, 41.2, 33.4, 31.3, 31.2, 21.7 ppm; **IR** (ν_{max}, cm^{-1}) 2950 (w), 1334 (m), 1158 (s), 1106 (m), 902 (m), 869 (m), 678 (m), 550 (m); **HRMS** (ESI/QTOF) m/z: $[M + H]^+$ Calcd for $C_{14}H_{19}ClNO_2S^+$ 300.0820; Found 300.0817.

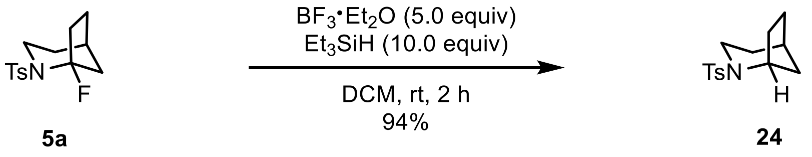

2-Tosyl-2-azabicyclo[3.2.1]octane (24): To a stirred solution of compound **5a** (14.2 mg, 0.05 mmol, 1.0 equiv) in anhydrous dichloromethane (1.0 mL, *c* = 0.05 mol/L) was added triethylsilane (80.0 µL, 0.50 mmol, 10.0 equiv) at room temperature under N_2, followed by the addition of boron trifluoride diethyl etherate (30.9 µL, 0.25 mmol, 5.0 equiv). The mixture was allowed to stir at room temperature for 2 h. TLC showed that the starting material was consumed completely, then the reaction mixture was quenched by the addition of saturated sodium bicarbonate. The resulting mixture was extracted with ethyl acetate (3×3 mL). The combined organic layers were washed with brine (2×3 mL), dried over anhydrous sodium sulfate, filtered and concentrated under vacuum. The residue was purified by preparative thin layer chromatography (20% ethyl acetate-hexane) to give compound **24** as a pale yellow oil (12.5 mg, 94%). **^{1}H NMR** (800 MHz, $CDCl_3$) δ 7.65 (d, *J* = 7.8 Hz, 2H), 7.29 (d, *J* = 7.9 Hz, 2H), 4.34 (t, *J* = 5.1 Hz, 1H), 3.62 (dd, *J* = 12.2, 6.3 Hz, 1H), 2.66 (td, *J* = 12.3, 4.5 Hz, 1H), 2.43 (s, 3H), 2.32 – 2.28 (m, 1H), 1.69 (td, *J* = 12.6, 5.9 Hz, 1H), 1.63 (d, *J* = 11.8 Hz, 1H), 1.59 (dq, *J* = 12.4, 6.4, 5.6 Hz, 1H), 1.48 (ddd, *J* = 18.7, 13.2, 5.2 Hz, 1H), 1.41 (dt, *J* = 10.8, 4.8 Hz, 1H), 1.37 – 1.32 (m, 1H), 1.31 – 1.27 (m, 1H), 1.07 – 1.01 (m, 1H) ppm; **^{13}C NMR** (200 MHz, $CDCl_3$) δ 143.2, 136.2, 129.7 (2C), 127.5 (2C), 56.5, 39.8, 39.1, 33.5, 30.6, 27.5, 27.3, 21.7 ppm; **IR** (ν_{max}, cm^{-1}) 2933 (w), 2858 (w), 1346 (m), 1333 (m), 1166 (s), 1154 (s), 1094 (s), 969 (m), 687 (s), 570 (m), 550 (s); **HRMS** (ESI/QTOF) m/z: $[M + H]^+$ Calcd for $C_{14}H_{20}NO_2S^+$ 266.1209; Found 266.1205.

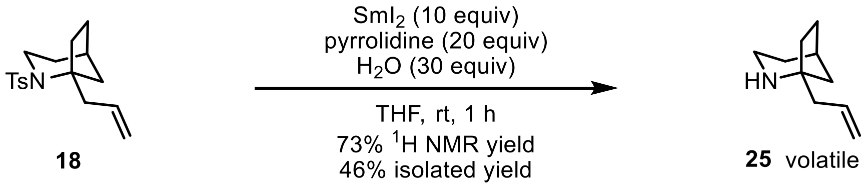

1-Allyl-2-azabicyclo[3.2.1]octane (25): To a flask charged with sulfonamide **18** (15.3 mg, 0.05 mmol, 1.0 equiv) was added samarium(II) iodide (5.0 mL, 0.1 M in tetrahydrofuran, 0.50 mmol, 10 equiv) at room temperature, followed by water (27 µL, 1.5 mmol, 30 equiv) and pyrrolidine (84 µL, 1.0 mmol, 20 equiv) under N_2 atmosphere. After stirring at room temperature for 1 h, TLC showed that the starting material was consumed completely, the

resulting mixture was diluted with diethyl ether (10 mL) and treated with an aqueous solution of 10% potassium sodium tartrate (5 mL) and an aqueous solution of 10% potassium carbonate (5 mL). The resulting mixture was extracted with diethyl ether (3×10 mL). The combined organic layers were dried over anhydrous sodium sulfate, filtered and concentrated under vacuum at 0 ºC to yield the crude amine (73% ^{1}H NMR yield using CH_2Br_2 as internal standard). The residue was purified by preparative thin layer chromatography (methanol/dichloromethane/ammonium hydroxide, 7:93:2) to give volatile compound **25** as a colorless oil (3.5 mg, 46%). **^{1}H NMR** (600 MHz, $CDCl_3$) δ 5.82 (dddd, *J* = 17.1, 10.1, 7.8, 7.1 Hz, 1H), 5.11 – 5.06 (m, 2H), 2.98-2.90 (m, 2H), 2.33-2.30 (m, 1H), 2.29 – 2.21 (m, 2H), 2.19 (br, 1H), .81 – 1.73 (m, 1H), 1.69 – 1.52 (m, 4H), 1.45 (ddd, *J* = 11.1, 5.0, 1.9 Hz, 1H), 1.40 (dq, *J* = 11.0, 1.7 Hz, 1H), 1.35 (dddd, *J* = 12.6, 5.9, 3.9, 1.6 Hz, 1H) ppm; **^{13}C NMR** (150 MHz, $CDCl_3$) δ 134.8, 118.1, 62.1, 44.9, 44.5, 40.4, 34.9, 34.5, 32.2, 28.9 ppm; **IR** (ν_{max}, cm^{-1}) 2923 (s), 2855 (m), 1639 (w), 1446 (m), 1100 (m), 1034 (m), 996 (m), 914 (m), 701 (w), 532 (w); **HRMS** (Nanochip-based ESI/LTQ-Orbitrap) m/z: $[M + H]^+$ Calcd for $C_{10}H_{18}N^+$ 152.1434; Found 152.1435.

VII. X-ray crystallographic data

Crystal Data and Experimental of 1-fluoro-2-tosyl-2-azabicyclo[3.2.1]octane (5a) (CCDC: 2417047)

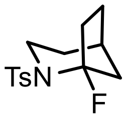

5a

***R₁*=6.78%**

Crystal Data and Experimental

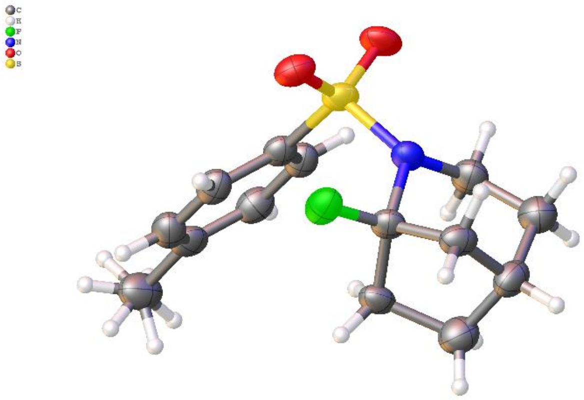

Compound	ygq-bridged-ring-f
Formula	$C_{14}H_{18}FNO_2S$
$D_{calc.}$/ g cm^{-3}	1.422
μ/mm^{-1}	2.273
Formula Weight	283.35
Colour	clear pale colourless
Shape	plate-shaped
Size/mm^3	0.17×0.15×0.07
T/K	140.00(10)
Crystal System	triclinic
Space Group	P-1
a/Å	6.3479(4)
b/Å	7.6082(4)
c/Å	14.5729(9)
α/°	74.925(5)
β/°	88.147(5)
γ/°	76.876(5)
V/Å^3	661.58(7)
Z	2
Z'	1
Wavelength/Å	1.54184
Radiation type	Cu K$_\alpha$
Θ_{min}/°	3.142
Θ_{max}/°	75.208
Measured Refl's.	5908
Indep't Refl's	2518
Refl's I≥2 σ(I)	1922
R_{int}	0.0312
Parameters	174
Restraints	0
Largest Peak	0.981
Deepest Hole	-0.522
GooF	1.074
wR_2 (all data)	0.2004
wR_2	0.1872
R_1 (all data)	0.0874
R_1	0.0678

Experimental. Single clear pale colourless plate-shaped crystals of **ygq-bridged-ring-f** were used as supplied. A suitable crystal with dimensions 0.17 × 0.15 × 0.07 mm^3 was selected and mounted on a XtaLAB Synergy R, DW system, HyPix-Arc 150 diffractometer. The crystal was kept at a steady T = 140.00(10) K during data collection. The structure was solved with the **ShelXT** (Sheldrick, 2015) solution program using dual methods and by using **Olex2** 1.5 (Dolomanov et al., 2009) as the graphical interface. The model was refined with **ShelXL** 2018/3 (Sheldrick, 2015) using full matrix least squares minimisation on $\boldsymbol{F}^2$.

Crystal Data. $C_{14}H_{18}FNO_2S$, M_r = 283.35, triclinic, P-1 (No. 2), a = 6.3479(4) Å, b = 7.6082(4) Å, c = 14.5729(9) Å, α = 74.925(5)°, β = 88.147(5)°, γ = 76.876(5)°, V = 661.58(7) Å^3, T = 140.00(10) K, Z = 2, Z' = 1, μ(Cu K$_\alpha$) = 2.273, 5908 reflections measured, 2518 unique (R_{int} = 0.0312) which were used in all calculations. The final wR_2 was 0.2004 (all data) and R_1 was 0.0678 (I≥2 σ(I)).

Structure Quality Indicators

**Reflections:**	d min (Cu\a) 2Θ=150.4° 0.80	I/σ(I) 22.1	Rint 3.12%	CAP 133.9° 92% to 150.4° 97.9
**Refinement:**	Shift 0.000	Max Peak 1.0	Min Peak -0.5	GooF 1.074

A clear pale colourless plate-shaped-shaped crystal with dimensions 0.17 × 0.15 × 0.07 mm^3 was mounted. Data were collected using a XtaLAB Synergy R, DW system, HyPix-Arc 150 diffractometer operating at T = 140.00(10) K.

Data were measured using ω scans with Cu K_α radiation. The diffraction pattern was indexed and the total number of runs and images was based on the strategy calculation from the program CrysAlisPro 1.171.41.120a (Rigaku OD, 2021). The maximum resolution that was achieved was Θ = 75.208° (0.80 Å).

The unit cell was refined using CrysAlisPro 1.171.41.120a (Rigaku OD, 2021) on 2233 reflections, 38% of the observed reflections.

Data reduction, scaling and absorption corrections were performed using CrysAlisPro 1.171.41.120a (Rigaku OD, 2021). The final completeness is 97.70 % out to 75.208° in Θ. A gaussian absorption correction was performed using CrysAlisPro 1.171.41.120a (Rigaku Oxford Diffraction, 2021). The numerical absorption correction was based on gaussian integration over a multifaceted crystal model. The empirical absorption correction was done using spherical harmonics, implemented in SCALE3 ABSPACK scaling algorithm. The absorption coefficient μ of this crystal is 2.273 mm^{-1} at this wavelength (λ = 1.54184Å) and the minimum and maximum transmissions are 0.665 and 1.000.

The structure was solved and the space group P-1 (# 2) determined by the ShelXT (Sheldrick, 2015) structure solution program using dual methods and refined by full matrix least squares minimisation on $\boldsymbol{F^2}$ using version 2018/3 of **ShelXL** (Sheldrick, 2015). All non-hydrogen atoms were refined anisotropically. Hydrogen atom positions were calculated geometrically and refined using the riding model.

There is a single molecule in the asymmetric unit, which is represented by the reported sum formula. In other words: Z is 2 and Z' is 1.

Data Plots: Diffraction Data

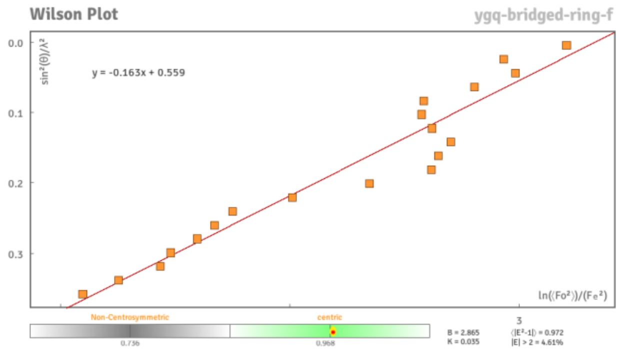

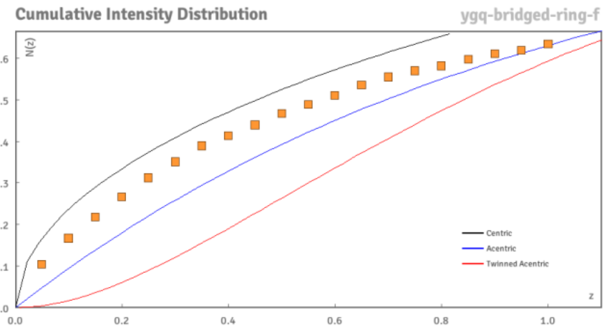

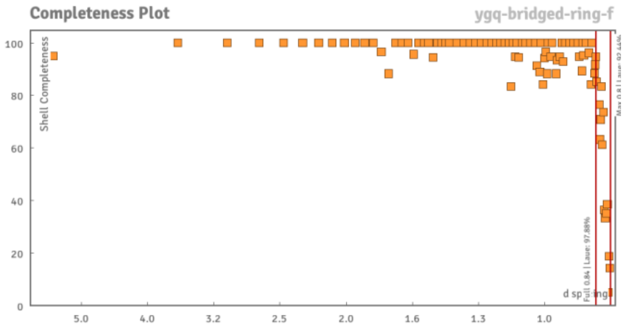

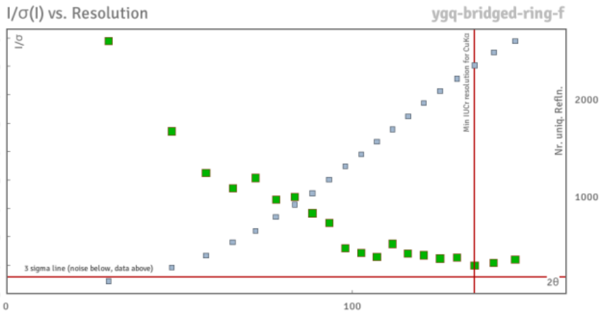

Data Plots: Refinement and Data

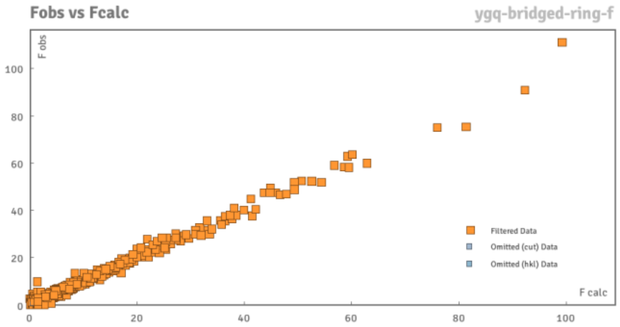

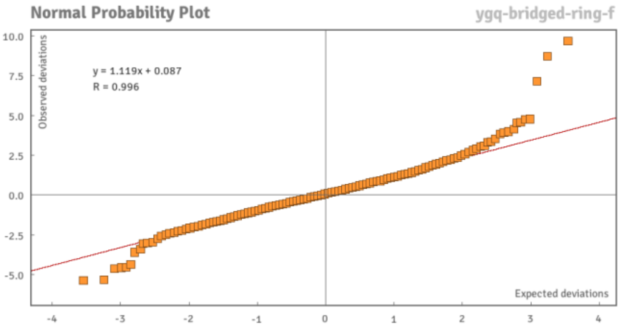

Reflection Statistics

Total reflections (after filtering)	5908	Unique reflections	2518
Completeness	0.924	Mean I/σ	13.85
hkl_{max} collected	(7, 9, 17)	hkl_{min} collected	(-7, -9, -17)
hkl_{max} used	(7, 9, 17)	hkl_{min} used	(-7, -9, 0)
Lim d_{max} collected	100.0	Lim d_{min} collected	0.77
d_{max} used	14.07	d_{min} used	0.8
Friedel pairs	754	Friedel pairs merged	1
Inconsistent equivalents	6	R_{int}	0.0312
R_{sigma}	0.0451	Intensity transformed	0
Omitted reflections	0	Omitted by user (OMIT hkl)	0
Multiplicity	(1802, 951, 271, 89, 51, 39, 30, 24, 8, 5, 2)	Maximum multiplicity	11
Removed systematic absences	0	Filtered off (Shel/OMIT)	0

Table 1: Fractional Atomic Coordinates (×10^4) and Equivalent Isotropic Displacement Parameters (Å^2×10^3) for **ygq-bridged-ring-f.** U_{eq} is defined as 1/3 of the trace of the orthogonalised U_{ij}.

Atom	x	y	z	U_{eq}
S1	4347.0(15)	7479.5(10)	7751.3(6)	37.1(3)
F1	3126(3)	4032(3)	7793.7(15)	46.2(6)
O1	2085(4)	7633(3)	7623.2(19)	44.8(7)
O2	5046(5)	8787(3)	8158.8(18)	47.5(7)
N1	5413(5)	5410(4)	8452.9(19)	34.2(7)
C1	5095(6)	3651(4)	8249(2)	35.2(8)
C2	6923(6)	2774(5)	7686(2)	40.9(9)
C3	8615(7)	1477(6)	8446(3)	52.2(10)
C4	7668(7)	1694(6)	9390(3)	48.1(10)
C5	5235(6)	2200(5)	9195(2)	40.6(9)
C6	8323(7)	3424(6)	9609(3)	52.2(10)

Atom	x	y	z	U_{eq}
C7	7682(7)	5152(5)	8809(3)	46.2(9)
C8	5561(6)	7597(4)	6641(2)	34.4(7)
C9	4607(6)	7090(5)	5933(3)	40.7(8)
C10	5605(7)	7152(5)	5082(3)	44.3(9)
C11	7599(7)	7660(5)	4918(3)	42.3(9)
C12	8513(6)	8149(5)	5634(3)	43.6(9)
C13	7533(6)	8137(5)	6492(3)	39.7(8)
C14	8706(8)	7648(6)	3990(3)	57.7(12)

Table 2: Anisotropic Displacement Parameters (×10[4]) for **ygq-bridged-ring-f**. The anisotropic displacement factor exponent takes the form: $-2\pi^2[h^2a^{*2} \times U_{11} + ... + 2hka^* \times b^* \times U_{12}]$

Atom	U_{11}	U_{22}	U_{33}	U_{23}	U_{13}	U_{12}
S1	45.4(5)	22.2(4)	43.9(5)	-10.6(3)	2.3(4)	-6.0(3)
F1	47.8(13)	32.6(11)	59.2(13)	-10.7(9)	-12.8(10)	-10.4(9)
O1	43.1(15)	31.2(13)	56.1(15)	-9.4(11)	3.2(12)	-2.9(10)
O2	70.0(19)	26.4(12)	51.0(15)	-18.3(11)	4.5(13)	-11.9(12)
N1	44.6(17)	24.6(13)	34.7(14)	-9.1(10)	-0.1(12)	-8.7(11)
C1	42(2)	25.0(16)	41.1(18)	-11.1(13)	-0.3(15)	-10.0(14)
C2	57(2)	28.6(17)	38.6(18)	-13.9(13)	4.5(16)	-8.0(15)
C3	51(2)	46(2)	55(2)	-11.3(18)	2.5(19)	-4.7(18)
C4	50(2)	41(2)	42(2)	-1.6(15)	-0.7(17)	1.1(16)
C5	52(2)	27.7(17)	41.1(18)	-7.4(14)	7.6(16)	-8.8(15)
C6	57(3)	46(2)	53(2)	-15.4(18)	-3.1(19)	-5.6(18)
C7	56(2)	34.5(19)	51(2)	-15.1(16)	-8.0(18)	-11.0(17)
C8	40.8(19)	22.3(15)	39.1(17)	-5.5(12)	-1.3(14)	-7.7(13)
C9	44(2)	29.5(17)	49(2)	-7.3(14)	-7.8(16)	-11.1(15)
C10	57(2)	32.9(18)	43.8(19)	-8.8(14)	-7.9(17)	-11.3(16)
C11	55(2)	23.0(16)	43.6(19)	-4.7(13)	2.3(16)	-3.9(14)
C12	42(2)	33.2(18)	51(2)	-3.9(15)	0.5(16)	-8.8(15)
C13	45(2)	30.1(17)	44.9(19)	-7.8(14)	-6.1(16)	-11.5(15)
C14	78(3)	44(2)	46(2)	-9.2(17)	15(2)	-10(2)

Table 3: Bond Lengths in Å for **ygq-bridged-ring-f**.

Atom	Atom	Length/Å
S1	O1	1.429(3)
S1	O2	1.437(3)
S1	N1	1.645(3)
S1	C8	1.759(3)
F1	C1	1.369(4)
N1	C1	1.501(4)
N1	C7	1.500(5)
C1	C2	1.528(5)
C1	C5	1.516(4)
C2	C3	1.543(6)
C3	C4	1.517(5)
C4	C5	1.522(5)
C4	C6	1.576(6)
C6	C7	1.498(5)
C8	C9	1.390(5)
C8	C13	1.398(5)
C9	C10	1.369(5)
C10	C11	1.401(5)
C11	C12	1.381(6)
C11	C14	1.504(5)
C12	C13	1.377(5)

Table 4: Bond Angles in ° for **ygq-bridged-ring-f**.

Atom	Atom	Atom	Angle/°
O1	S1	O2	119.32(17)
O1	S1	N1	108.86(15)

Atom	Atom	Atom	Angle/°
O1	S1	C8	108.22(17)
O2	S1	N1	105.09(15)
O2	S1	C8	107.50(15)
N1	S1	C8	107.27(15)
C1	N1	S1	121.4(2)
C7	N1	S1	115.6(2)
C7	N1	C1	110.6(3)
F1	C1	N1	108.5(3)
F1	C1	C2	111.6(3)
F1	C1	C5	112.4(3)
N1	C1	C2	113.4(3)
N1	C1	C5	107.1(3)
C5	C1	C2	103.7(3)
C1	C2	C3	104.9(3)
C4	C3	C2	105.1(3)
C3	C4	C5	103.9(3)

Atom	Atom	Atom	Angle/°
C3	C4	C6	108.0(3)
C5	C4	C6	107.9(3)
C1	C5	C4	100.2(3)
C7	C6	C4	111.9(3)
C6	C7	N1	111.6(3)
C9	C8	S1	120.3(3)
C9	C8	C13	120.5(3)
C13	C8	S1	119.2(3)
C10	C9	C8	119.3(3)
C9	C10	C11	121.5(4)
C10	C11	C14	120.6(4)
C12	C11	C10	117.9(3)
C12	C11	C14	121.5(4)
C13	C12	C11	122.0(4)
C12	C13	C8	118.7(3)

Table 5: Torsion Angles in ° for **ygq-bridged-ring-f**.

Atom	Atom	Atom	Atom	Angle/°
S1	N1	C1	F1	-30.6(3)
S1	N1	C1	C2	94.0(3)
S1	N1	C1	C5	-152.2(2)
S1	N1	C7	C6	166.6(3)
S1	C8	C9	C10	-178.5(3)
S1	C8	C13	C12	177.3(3)
F1	C1	C2	C3	-149.0(3)
F1	C1	C5	C4	165.4(3)
O1	S1	N1	C1	52.6(3)
O1	S1	N1	C7	-168.9(2)
O1	S1	C8	C9	-24.9(3)
O1	S1	C8	C13	157.6(3)
O2	S1	N1	C1	-178.5(2)
O2	S1	N1	C7	-40.0(3)
O2	S1	C8	C9	-155.0(3)
O2	S1	C8	C13	27.5(3)
N1	S1	C8	C9	92.4(3)
N1	S1	C8	C13	-85.1(3)
N1	C1	C2	C3	88.1(3)
N1	C1	C5	C4	-75.5(3)
C1	N1	C7	C6	-50.6(4)
C1	C2	C3	C4	-0.3(4)
C2	C1	C5	C4	44.7(3)
C2	C3	C4	C5	28.1(4)
C2	C3	C4	C6	-86.3(4)
C3	C4	C5	C1	-45.0(4)
C3	C4	C6	C7	54.1(4)
C4	C6	C7	N1	45.6(4)
C5	C1	C2	C3	-27.7(4)
C5	C4	C6	C7	-57.6(4)
C6	C4	C5	C1	69.5(3)
C7	N1	C1	F1	-171.0(3)
C7	N1	C1	C2	-46.3(4)
C7	N1	C1	C5	67.5(3)
C8	S1	N1	C1	-64.3(3)
C8	S1	N1	C7	74.2(3)
C8	C9	C10	C11	2.0(5)

Atom	Atom	Atom	Atom	Angle/°
C9	C8	C13	C12	-0.2(5)
C9	C10	C11	C12	-1.7(5)
C9	C10	C11	C14	177.7(3)
C10	C11	C12	C13	0.4(5)
C11	C12	C13	C8	0.5(5)
C13	C8	C9	C10	-1.0(5)
C14	C11	C12	C13	-178.9(3)

Table 6: Hydrogen Fractional Atomic Coordinates (×10^4) and Equivalent Isotropic Displacement Parameters (Å^2×10^3) for **ygq-bridged-ring-f**. U_{eq} is defined as 1/3 of the trace of the orthogonalised U_{ij}.

Atom	x	y	z	*U*eq
H2A	7550.35	3748.6	7253.78	49
H2B	6388.42	2052.37	7305.13	49
H3A	8845.67	164.59	8411.86	63
H3B	10014.5	1857.62	8356.65	63
H4	8131.18	528.66	9914.31	58
H5A	4690.8	1109.29	9141.08	49
H5B	4436.42	2734.38	9691.18	49
H6A	7615.89	3655.36	10195.74	63
H6B	9908.35	3135.48	9723.48	63
H7A	8689.82	5066.1	8281.64	55
H7B	7796.7	6253.32	9029.49	55
H9	3277.93	6705.05	6039.39	49
H10	4929.39	6843.42	4591.63	53
H12	9859.68	8505.67	5532.7	52
H13	8185.63	8488.45	6974.01	48
H14A	9520.59	6377.51	4012.34	69
H14B	9700.09	8491.85	3884.09	69
H14C	7619.25	8066.11	3470.14	69
H14D	8372.69	8912.8	3565.37	69
H14E	8193.2	6798.46	3693.62	69
H14F	10274.03	7224.2	4107.58	69

Table 7: Atomic Occupancies for all atoms that are not fully occupied in **ygq-bridged-ring-f**.

Atom	Occupancy	Atom	Occupancy	Atom	Occupancy
H14A	0.62(5)	H14C	0.62(5)	H14E	0.38(5)
H14B	0.62(5)	H14D	0.38(5)	H14F	0.38(5)

Crystal Data and Experimental of 1-fluoro-6-phenyl-2-tosyl-2-azabicyclo[3.2.1]octane (5f) (CCDC: 2417050)

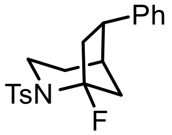

5f

R_1=6.03%

Crystal Data and Experimental

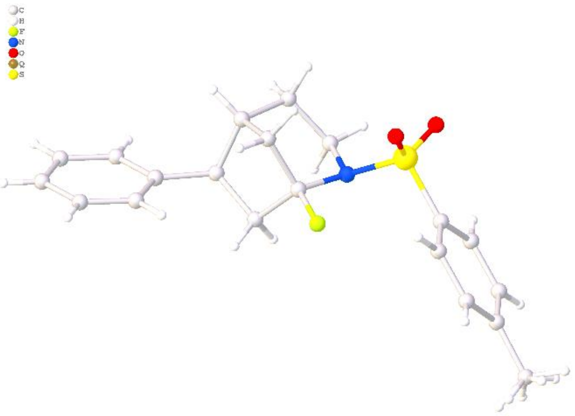

Experimental. Single clear pale colourless plate-shaped crystals of **ygq-701-2** were used as supplied. A suitable crystal with dimensions 0.24 × 0.07 × 0.05 mm^3 was selected and mounted on a XtaLAB Synergy R, DW system, HyPix-Arc 150 diffractometer. The crystal was kept at a steady T = 140.00(10) K during data collection. The structure was solved with the ShelXT 2018/2 (Sheldrick, 2018) solution program using dual methods and by using Olex2 1.5 (Dolomanov et al., 2009) as the graphical interface. The model was refined with ShelXL 2019/3 (Sheldrick, 2015) using full matrix least squares minimisation on $\boldsymbol{F}^2$.

Crystal Data. $C_{20}H_{22}NO_2FS$, M_r = 359.44, monoclinic, $P2_1/n$ (No. 14), a = 6.8200(3) Å, b = 38.2364(9) Å, c = 7.5082(3) Å, β = 117.008(5)°, α = γ = 90°, V = 1744.40(13) Å^3, T = 140.00(10) K, Z = 4, Z' = 1, μ(Cu K$_\alpha$) = 1.849, 18893 reflections measured, 3593 unique (R_{int} = 0.0431) which were used in all calculations. The final wR_2 was 0.1293 (all data) and R_1 was 0.0603 (I≥2 σ(I)).

**Compound**	**ygq-701-2**
Formula	$C_{20}H_{22}NO_2FS$
$D_{calc.}$/ g cm^{-3}	1.369
μ/mm^{-1}	1.849
Formula Weight	359.44
Colour	clear pale colourless
Shape	plate-shaped
Size/mm^3	0.24×0.07×0.05
T/K	140.00(10)
Crystal System	monoclinic
Space Group	$P2_1/n$
a/Å	6.8200(3)
b/Å	38.2364(9)
c/Å	7.5082(3)
α/°	90
β/°	117.008(5)
γ/°	90
V/Å^3	1744.40(13)
Z	4
Z'	1
Wavelength/Å	1.54184
Radiation type	Cu K$_\alpha$
Θ_{min}/°	2.311
Θ_{max}/°	75.426
Measured Refl's.	18893
Indep't Refl's	3593
Refl's I≥2 σ(I)	3127
R_{int}	0.0431
Parameters	229
Restraints	0
Largest Peak	0.670
Deepest Hole	-0.415
GooF	1.125
wR_2 (all data)	0.1293
wR_2	0.1261
R_1 (all data)	0.0686
R_1	0.0603

Structure Quality Indicators

**Reflections:**	d min (CuKα) 2Θ=150.9° 0.80	I/σ(I) 33.8	Rint m=5.34 4.31%	Full 135.4° 99% to 150.9° 99.8
**Refinement:**	Shift -0.001	Max Peak 0.7	Min Peak -0.4	GooF 1.125

A clear pale colourless plate-shaped-shaped crystal with dimensions 0.24 × 0.07 × 0.05 mm^3 was mounted. Data were collected using a XtaLAB Synergy R, DW system, HyPix-Arc 150 diffractometer operating at T = 140.00(10) K.

Data were measured using ω scans with Cu K_{α} radiation. The diffraction pattern was indexed and the total number of runs and images was based on the strategy calculation from the program CrysAlisPro system (CCD 43.143a 64-bit (release 25-10-2024)). The maximum resolution that was achieved was Θ = 75.426° (0.80 Å).

The unit cell was refined using CrysAlisPro 1.171.43.143a (Rigaku OD, 2024) on 8688 reflections, 46% of the observed reflections.

Data reduction, scaling and absorption corrections were performed using CrysAlisPro 1.171.43.143a (Rigaku OD, 2024). The final completeness is 99.80 % out to 75.426° in Θ. A analytical absorption correction was performed using CrysAlisPro 1.171.43.143a (Rigaku Oxford Diffraction, 2024) Analytical numeric absorption correction using a multifaceted crystal model based on expressions derived by R.C. Clark & J.S. Reid. (Clark, R. C. & Reid, J. S. (1995). Acta Cryst. A51, 887-897) Empirical absorption correction using spherical harmonics, implemented in SCALE3 ABSPACK scaling algorithm.. The absorption coefficient μ of this material is 1.849 mm^{-1} at this wavelength (λ = 1.54184Å) and the minimum and maximum transmissions are 0.723 and 0.925.

The structure was solved and the space group $P2_1/n$ (# 14) determined by the ShelXT 2018/2 (Sheldrick, 2018) structure solution program using using dual methods and refined by full matrix least squares minimisation on $\boldsymbol{F}^2$ using version 2019/3 of ShelXL 2019/3 (Sheldrick, 2015). All non-hydrogen atoms were refined anisotropically. Hydrogen atom positions were calculated geometrically and refined using the riding model. Hydrogen atom positions were calculated geometrically and refined using the riding model.

_exptl_absorpt_process_details: CrysAlisPro 1.171.43.143a (Rigaku Oxford Diffraction, 2024)Analytical numeric absorption correction using a multifaceted crystalmodel based on expressions derived by R.C. Clark & J.S. Reid.(Clark, R. C. & Reid, J. S. (1995). Acta Cryst. A51, 887-897) using spherical harmonics,implemented in SCALE3 ABSPACK scaling algorithm.

There is a single formula unit in the asymmetric unit, which is represented by the reported sum formula. In other words: Z is 4 and Z' is 1. The moiety formula is C20 H22 F N O2 S.

Data Plots: Diffraction Data

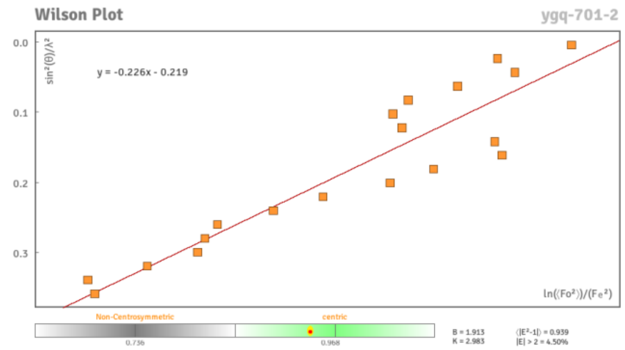

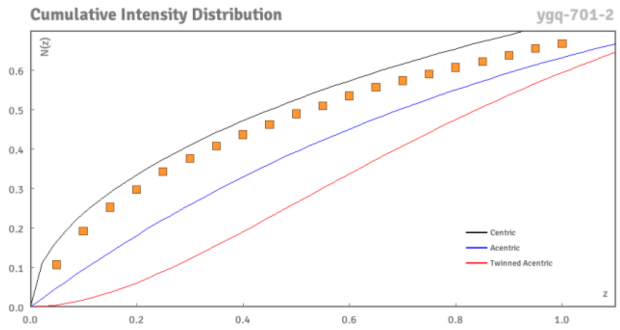

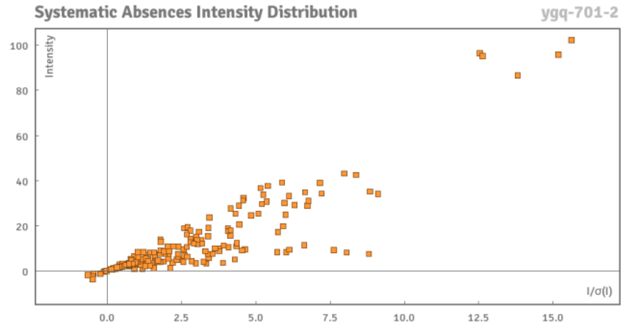

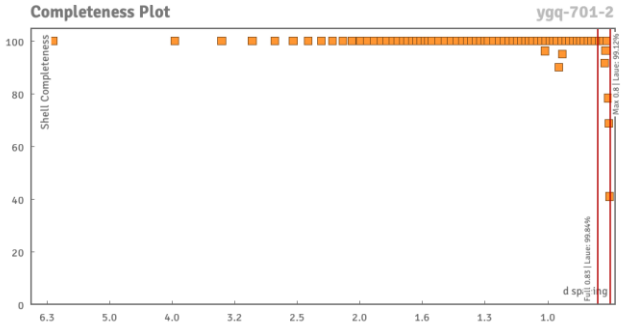

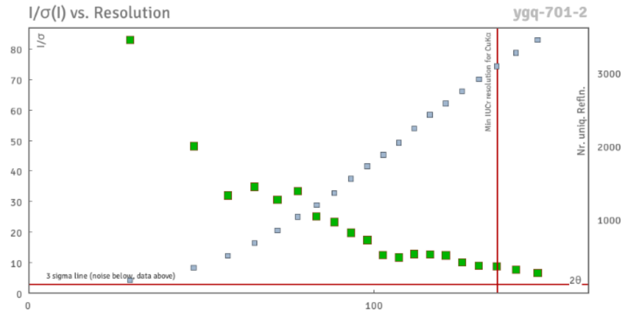

Data Plots: Refinement and Data

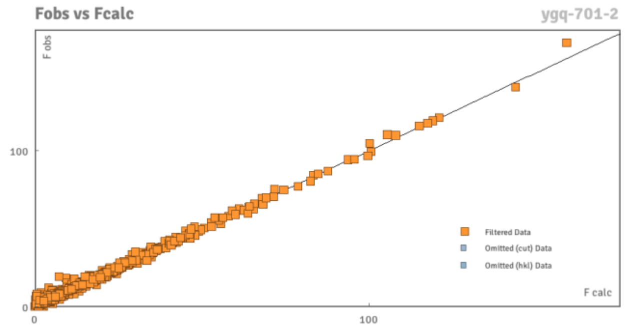

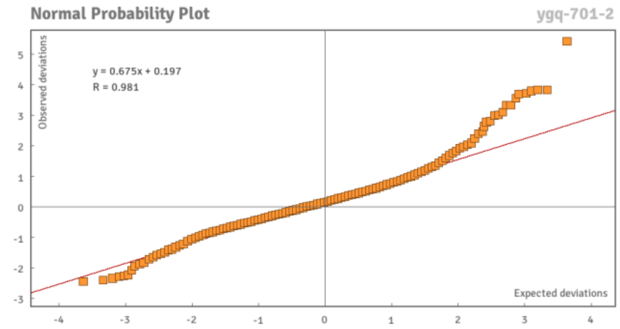

Reflection Statistics

Total reflections (after filtering)	19170	Unique reflections	3593
Completeness	0.991	Mean I/σ	22.73
hkl_{max} collected	(8, 45, 9)	hkl_{min} collected	(-8, -47, -8)
hkl_{max} used	(7, 47, 9)	hkl_{min} used	(-8, 0, 0)
Lim d_{max} collected	100.0	Lim d_{min} collected	0.77

d_{max} used	19.12	d_{min} used	0.8
Friedel pairs	2482	Friedel pairs merged	1
Inconsistent equivalents	10	R_{int}	0.0431
R_{sigma}	0.0296	Intensity transformed	0
Omitted reflections	0	Omitted by user (OMIT hkl)	0
Multiplicity	(3784, 2481, 1138, 620, 284, 149, 88, 82, 43, 23, 13, 11, 4)	Maximum multiplicity	24
Removed systematic absences	277	Filtered off (Shel/OMIT)	0

Table 8: Fractional Atomic Coordinates (×10⁴) and Equivalent Isotropic Displacement Parameters (Å²×10³) for **ygq-701-2.** U_{eq} is defined as 1/3 of the trace of the orthogonalised U_{ij}.

Atom	x	y	z	U_{eq}
S1	5577.6(11)	5941.3(2)	7240.8(10)	21.80(18)
F1	1135(3)	6168.3(5)	4843(3)	30.2(4)
O1	5212(4)	6212.6(5)	8370(3)	28.3(5)
O2	7793(3)	5839.1(5)	7757(3)	28.8(5)
N1	4415(4)	6034.3(6)	4825(3)	22.0(5)
C1	2492(4)	6268.0(8)	3989(4)	22.2(6)
C2	3153(5)	6649.6(8)	4324(5)	26.7(6)
C3	4103(5)	6686.8(8)	2830(5)	29.8(7)
C4	2292(5)	6517.6(8)	915(5)	29.1(7)
C5	1263(5)	6234.0(8)	1722(4)	27.8(6)
C6	6236(5)	6469.7(9)	3602(5)	33.2(7)
C7	5848(5)	6089.0(8)	3819(5)	31.4(7)
C8	725(5)	6793.9(8)	-462(5)	29.4(7)
C9	-1123(5)	6919.8(9)	-319(5)	34.5(7)
C10	-2442(6)	7178.3(9)	-1591(6)	39.9(8)
C11	-1957(6)	7316.4(9)	-3043(5)	43.5(9)
C12	-127(7)	7197.8(9)	-3191(5)	43.9(9)
C13	1192(6)	6941.8(9)	-1919(5)	37.6(8)
C14	4178(4)	5563.4(7)	7402(4)	20.9(6)
C15	2448(5)	5589.5(8)	7892(4)	23.7(6)
C16	1378(5)	5286.8(8)	7983(4)	25.5(6)
C17	1992(5)	4959.3(8)	7605(4)	24.9(6)
C18	3754(5)	4939.5(8)	7143(4)	26.2(6)
C19	4842(5)	5239.6(8)	7034(4)	25.3(6)
C20	779(5)	4635.0(8)	7667(5)	30.5(7)

Table 9: Anisotropic Displacement Parameters (×10⁴) for **ygq-701-2.** The anisotropic displacement factor exponent takes the form: $-2\pi^2[h^2a^{*2} \times U_{11} + ... + 2hka^* \times b^* \times U_{12}]$

Atom	U_{11}	U_{22}	U_{33}	U_{23}	U_{13}	U_{12}
S1	17.9(3)	22.7(3)	22.0(3)	-1.8(3)	6.7(3)	-1.2(3)
F1	21.6(8)	42.6(11)	31.5(9)	7.6(8)	16.5(7)	2.1(7)
O1	33.4(11)	24.9(11)	26.8(11)	-7.5(8)	13.9(9)	-3.3(9)
O2	16.6(10)	31.6(11)	33.1(11)	2.3(9)	6.8(9)	1.1(8)
N1	20.5(12)	24.1(12)	22.5(12)	0.9(9)	10.7(10)	1.9(9)
C1	18.3(13)	29.6(15)	20.5(13)	1.0(11)	10.4(11)	-0.8(11)
C2	28.5(15)	24.4(15)	28.0(15)	-2.6(12)	13.7(13)	3.8(12)
C3	31.7(17)	23.8(15)	36.8(17)	-1.8(13)	18.0(14)	-2.6(12)
C4	29.6(16)	31.0(16)	28.7(16)	-2.4(13)	15.0(13)	-2.7(13)
C5	24.4(15)	32.1(16)	23.4(14)	-2.2(12)	7.9(12)	-6.1(12)
C6	24.9(16)	37.9(18)	38.4(18)	2.1(14)	15.8(14)	-1.3(13)
C7	28.5(16)	33.7(17)	39.3(18)	4.9(14)	21.6(14)	7.7(13)
C8	30.9(16)	27.2(16)	27.7(16)	1.2(12)	11.2(13)	-2.0(13)
C9	29.0(17)	33.9(18)	40.1(18)	3.9(14)	15.2(15)	-0.7(13)

Atom	U_{11}	U_{22}	U_{33}	U_{23}	U_{13}	U_{12}
C10	28.2(17)	31.0(18)	47(2)	-0.3(15)	5.5(15)	2.6(14)
C11	50(2)	25.6(17)	35.3(19)	2.9(14)	1.9(17)	-0.2(15)
C12	60(2)	34.9(19)	32.3(18)	4.5(15)	17.1(18)	-2.8(17)
C13	49(2)	33.9(18)	33.8(17)	4.3(14)	21.8(16)	-1.4(15)
C14	18.6(13)	22.6(14)	18.2(13)	0.9(11)	5.4(11)	-1.3(11)
C15	23.0(14)	25.5(15)	22.4(14)	-0.1(11)	10.2(12)	3.4(11)
C16	23.6(14)	30.7(16)	22.4(14)	1.5(12)	10.5(12)	-0.8(12)
C17	24.4(15)	28.3(15)	16.5(13)	2.9(11)	4.4(12)	-2.5(12)
C18	29.1(16)	24.5(15)	24.1(14)	-1.1(12)	11.1(12)	2.1(12)
C19	25.0(14)	27.9(15)	23.6(14)	-1.3(12)	11.5(12)	-0.5(12)
C20	33.6(17)	32.2(16)	23.8(15)	2.6(12)	11.4(14)	-5.1(13)

Table 10: Bond Lengths in Å for **ygq-701-2**.

Atom	Atom	Length/Å
S1	O1	1.432(2)
S1	O2	1.433(2)
S1	N1	1.655(2)
S1	C14	1.766(3)
F1	C1	1.397(3)
N1	C1	1.471(4)
N1	C7	1.496(4)
C1	C2	1.514(4)
C1	C5	1.524(4)
C2	C3	1.535(4)
C3	C4	1.549(4)
C3	C6	1.541(4)
C4	C5	1.556(4)
C4	C8	1.524(4)
C6	C7	1.502(4)
C8	C9	1.398(4)
C8	C13	1.391(4)
C9	C10	1.385(5)
C10	C11	1.380(5)
C11	C12	1.379(6)
C12	C13	1.378(5)
C14	C15	1.390(4)
C14	C19	1.388(4)
C15	C16	1.386(4)
C16	C17	1.390(4)
C17	C18	1.396(4)
C17	C20	1.503(4)
C18	C19	1.389(4)

Table 11: Bond Angles in ° for **ygq-701-2**.

Atom	Atom	Atom	Angle/°
O1	S1	O2	118.89(13)
O1	S1	N1	111.59(12)
O1	S1	C14	107.91(13)
O2	S1	N1	106.03(12)
O2	S1	C14	107.14(13)
N1	S1	C14	104.26(13)
C1	N1	S1	119.22(18)
C1	N1	C7	111.1(2)
C7	N1	S1	119.1(2)
F1	C1	N1	106.7(2)
F1	C1	C2	113.4(2)
F1	C1	C5	110.3(2)
N1	C1	C2	112.0(2)
N1	C1	C5	110.9(2)
C2	C1	C5	103.8(2)
C1	C2	C3	99.3(2)
C2	C3	C4	102.5(2)
C2	C3	C6	108.3(3)
C6	C3	C4	110.0(3)
C3	C4	C5	103.9(2)
C8	C4	C3	111.0(3)
C8	C4	C5	116.5(3)
C1	C5	C4	105.0(2)
C7	C6	C3	112.4(3)
N1	C7	C6	112.2(2)
C9	C8	C4	124.4(3)
C13	C8	C4	118.3(3)
C13	C8	C9	117.2(3)
C10	C9	C8	121.3(3)
C11	C10	C9	120.3(3)
C12	C11	C10	119.2(3)
C13	C12	C11	120.5(3)
C12	C13	C8	121.5(3)
C15	C14	S1	120.6(2)
C19	C14	S1	118.7(2)
C19	C14	C15	120.6(3)
C16	C15	C14	118.8(3)
C15	C16	C17	121.9(3)
C16	C17	C18	118.2(3)
C16	C17	C20	121.1(3)
C18	C17	C20	120.7(3)
C19	C18	C17	120.9(3)

Atom	Atom	Atom	Angle/˚
C14	C19	C18	119.6(3)

Table 12: Torsion Angles in ˚ for **ygq-701-2**.

Atom	Atom	Atom	Atom	Angle/˚
S1	N1	C1	F1	-42.7(3)
S1	N1	C1	C2	81.8(3)
S1	N1	C1	C5	-162.80(19)
S1	N1	C7	C6	-98.8(3)
S1	C14	C15	C16	179.3(2)
S1	C14	C19	C18	-179.6(2)
F1	C1	C2	C3	-167.3(2)
F1	C1	C5	C4	150.6(2)
O1	S1	N1	C1	-26.1(2)
O1	S1	N1	C7	115.4(2)
O1	S1	C14	C15	21.4(3)
O1	S1	C14	C19	-158.5(2)
O2	S1	N1	C1	-157.0(2)
O2	S1	N1	C7	-15.5(2)
O2	S1	C14	C15	150.5(2)
O2	S1	C14	C19	-29.4(3)
N1	S1	C14	C15	-97.4(2)
N1	S1	C14	C19	82.7(2)
N1	C1	C2	C3	72.0(3)
N1	C1	C5	C4	-91.6(3)
C1	N1	C7	C6	45.6(3)
C1	C2	C3	C4	48.1(3)
C1	C2	C3	C6	-68.2(3)
C2	C1	C5	C4	28.8(3)
C2	C3	C4	C5	-30.7(3)
C2	C3	C4	C8	95.3(3)
C2	C3	C6	C7	59.8(3)
C3	C4	C5	C1	1.5(3)
C3	C4	C8	C9	-86.2(4)
C3	C4	C8	C13	91.4(3)
C3	C6	C7	N1	-45.6(4)
C4	C3	C6	C7	-51.5(4)
C4	C8	C9	C10	178.3(3)
C4	C8	C13	C12	-178.7(3)
C5	C1	C2	C3	-47.6(3)
C5	C4	C8	C9	32.3(4)
C5	C4	C8	C13	-150.1(3)
C6	C3	C4	C5	84.4(3)
C6	C3	C4	C8	-149.7(3)
C7	N1	C1	F1	172.9(2)
C7	N1	C1	C2	-62.5(3)
C7	N1	C1	C5	52.9(3)
C8	C4	C5	C1	-120.9(3)
C8	C9	C10	C11	0.3(5)
C9	C8	C13	C12	-1.0(5)
C9	C10	C11	C12	-1.0(5)
C10	C11	C12	C13	0.7(5)
C11	C12	C13	C8	0.3(6)
C13	C8	C9	C10	0.6(5)
C14	S1	N1	C1	90.1(2)
C14	S1	N1	C7	-128.4(2)

Atom	Atom	Atom	Atom	Angle/°
C14	C15	C16	C17	0.2(4)
C15	C14	C19	C18	0.5(4)
C15	C16	C17	C18	0.6(4)
C15	C16	C17	C20	-178.7(3)
C16	C17	C18	C19	-0.9(4)
C17	C18	C19	C14	0.4(4)
C19	C14	C15	C16	-0.8(4)
C20	C17	C18	C19	178.3(3)

Table 13: Hydrogen Fractional Atomic Coordinates (×10[4]) and Equivalent Isotropic Displacement Parameters ($Å^2×10^3$) for **ygq-701-2**. U_{eq} is defined as 1/3 of the trace of the orthogonalised U_{ij}.

Atom	x	y	z	U_{eq}
H2A	4275.5	6695.1	5713.94	32
H2B	1870.56	6804.87	3984.37	32
H3	4359.28	6937.19	2610.73	36
H4	3017.02	6397.61	185.96	35
H5A	-330.53	6276.67	1233.12	33
H5B	1466.93	5997.73	1292.3	33
H6A	6878.71	6494.79	2662.7	40
H6B	7312.21	6562.69	4915.38	40
H7A	5144.76	5979.35	2478.22	38
H7B	7280.27	5971.95	4606.75	38
H9	-1480.63	6826.21	668.92	41
H10	-3685.87	7260.94	-1463.43	48
H11	-2872.78	7491.13	-3931.04	52
H12	227.66	7293.3	-4176.69	53
H13	2449.72	6864.42	-2040.4	45
H15	2006.04	5810.88	8159.92	28
H16	190.79	5303.66	8311.93	31
H18	4214.29	4717.8	6899.88	31
H19	6032.77	5223.38	6709.66	30
H20A	-565	4610.97	6402.31	37
H20B	392.26	4652.56	8771.52	37
H20C	1720.42	4430.13	7868.62	37
H20D	1596.79	4518.14	8959.33	37
H20E	639.52	4476.55	6590.11	37
H20F	-688.64	4698.97	7493.02	37

Table 14: Atomic Occupancies for all atoms that are not fully occupied in **ygq-701-2**.

Atom	Occupancy	Atom	Occupancy	Atom	Occupancy
H20A	0.52(4)	H20C	0.52(4)	H20E	0.48(4)
H20B	0.52(4)	H20D	0.48(4)	H20F	0.48(4)

Crystal Data and Experimental of 1-fluoro-2-tosyl-2-azabicyclo[3.2.1]octan-6-yl)butanenitrile (5r) (CCDC: 2417051)

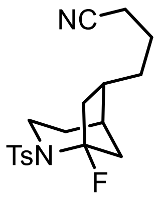

5r

R_1=3.63%

Crystal Data and Experimental

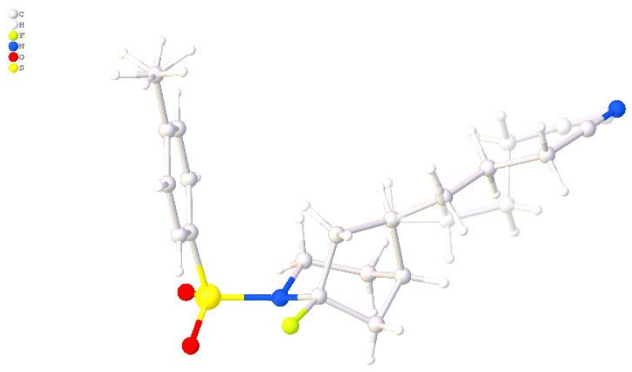

Compound	ygq-682-5
Formula	$C_{18}H_{23}FN_2O_2S$
$D_{calc.}$/ g cm^{-3}	1.366
μ/mm^{-1}	1.891
Formula Weight	350.44
Colour	clear pale colourless
Shape	irregular-shaped
Size/mm^3	0.26×0.24×0.10
T/K	139.99(10)
Crystal System	triclinic
Space Group	P-1
a/Å	6.3156(2)
b/Å	8.2646(2)
c/Å	16.7228(6)
α/°	80.995(3)
β/°	84.167(3)
γ/°	82.691(3)
V/Å^3	852.12(5)
Z	2
Z'	1
Wavelength/Å	1.54184
Radiation type	Cu K_α
Θ_{min}/°	5.375
Θ_{max}/°	76.137
Measured Refl's.	8412
Indep't Refl's	3500
Refl's I≥2 σ(I)	3336
R_{int}	0.0193
Parameters	317
Restraints	155
Largest Peak	0.355
Deepest Hole	-0.261
GooF	1.046
wR_2 (all data)	0.0979
wR_2	0.0966
R_1 (all data)	0.0379
R_1	0.0363

Experimental. Single clear pale colourless irregular-shaped crystals of **ygq-682-5** were used as supplied. A suitable crystal with dimensions 0.26 × 0.24 × 0.10 mm^3 was selected and mounted on a SuperNova, Dual, Cu at home/near, AtlasS2 diffractometer. The crystal was kept at a steady T = 139.99(10) K during data collection. The structure was solved with the ShelXT (Sheldrick, 2015) solution program using dual methods and by using Olex2 1.5 (Dolomanov et al., 2009) as the graphical interface. The model was refined with ShelXL 2019/3 (Sheldrick, 2015) using full matrix least squares minimisation on $\boldsymbol{F}^2$.

Crystal Data. $C_{18}H_{23}FN_2O_2S$, M_r = 350.44, triclinic, P-1 (No. 2), a = 6.3156(2) Å, b = 8.2646(2) Å, c = 16.7228(6) Å, α = 80.995(3)°, β = 84.167(3)°, γ = 82.691(3)°, V = 852.12(5) Å^3, T = 139.99(10) K, Z = 2, Z' = 1, μ(Cu K_α) = 1.891, 8412 reflections measured, 3500 unique (R_{int} = 0.0193) which were used in all calculations. The final wR_2 was 0.0979 (all data) and R_1 was 0.0363 (I≥2 σ(I)).

Structure Quality Indicators

**Reflections:**	d min (CuKα) 2Θ=152.3° 0.79	I/σ(I) 43.0	Rint m=2.40 1.93%	Full 135.4° 98% to 152.3° 99.9
**Refinement:**	Shift 0.001	Max Peak 0.3	Min Peak -0.3	GooF 1.046

A clear pale colourless irregular-shaped-shaped crystal with dimensions 0.26 × 0.24 × 0.10 mm^3 was mounted. Data were collected using a SuperNova, Dual, Cu at home/near, AtlasS2 diffractometer operating at T = 139.99(10) K.

Data were measured using ω scans with Cu K_α radiation. The diffraction pattern was indexed and the total number of runs and images was based on the strategy calculation from the program CrysAlisPro system (CCD 43.143a 64-bit (release 25-10-2024)). The maximum resolution that was achieved was Θ = 76.137° (0.79 Å).

The unit cell was refined using CrysAlisPro 1.171.43.143a (Rigaku OD, 2024) on 5532 reflections, 66% of the observed reflections.

Data reduction, scaling and absorption corrections were performed using CrysAlisPro 1.171.43.143a (Rigaku OD, 2024). The final completeness is 99.90 % out to 76.137° in Θ. A gaussian absorption correction was performed using CrysAlisPro 1.171.43.143a (Rigaku Oxford Diffraction, 2024) Numerical absorption correction based on gaussian integration over a multifaceted crystal model Empirical absorption correction using spherical harmonics, implemented in SCALE3 ABSPACK scaling algorithm.. The absorption coefficient μ of this material is 1.891 mm^{-1} at this wavelength (λ = 1.54184Å) and the minimum and maximum transmissions are 0.467 and 1.000.

The structure was solved and the space group P-1 (# 2) determined by the ShelXT (Sheldrick, 2015) structure solution program using using dual methods and refined by full matrix least squares minimisation on $\boldsymbol{F}^2$ using version 2019/3 of ShelXL 2019/3 (Sheldrick, 2015). All non-hydrogen atoms were refined anisotropically. Hydrogen atom positions were calculated geometrically and refined using the riding model. Most hydrogen atom positions were calculated geometrically and refined using the riding model, but some hydrogen atoms were refined freely.

_exptl_absorpt_process_details: CrysAlisPro 1.171.43.143a (Rigaku Oxford Diffraction, 2024)Numerical absorption correction based on gaussian integration overa multifaceted crystal modelEmpirical absorption correction using spherical harmonics,implemented in SCALE3 ABSPACK scaling algorithm.

There is a single formula unit in the asymmetric unit, which is represented by the reported sum formula. In other words: Z is 2 and Z' is 1. The moiety formula is C18 H23 F N2 O2 S.

Data Plots: Diffraction Data

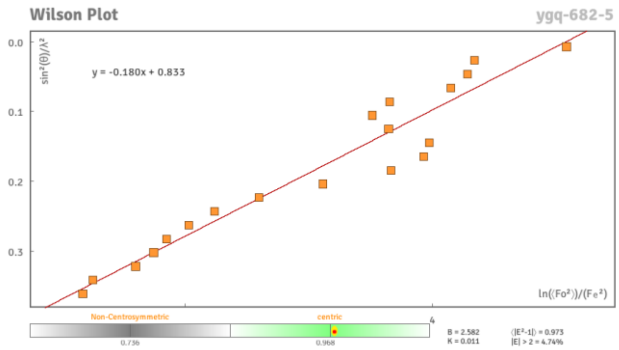

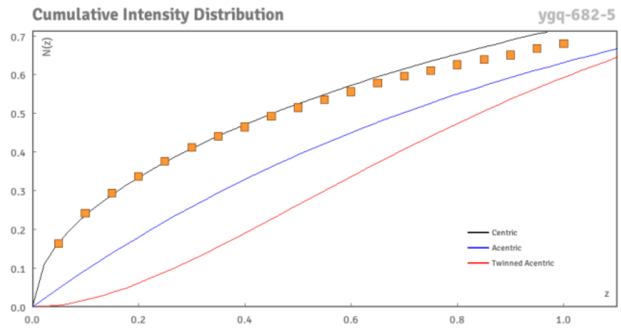

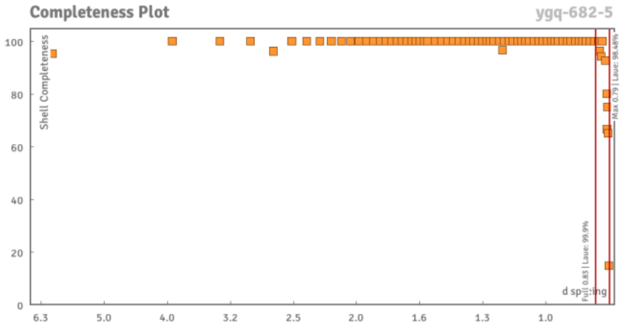

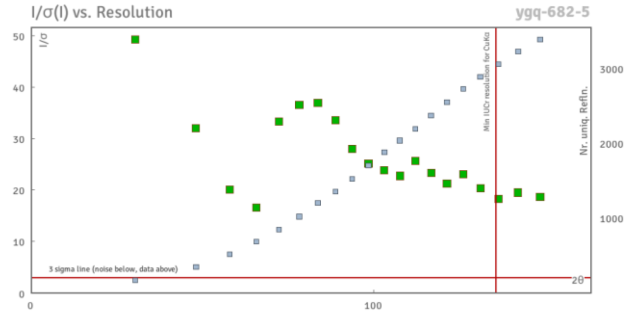

Data Plots: Refinement and Data

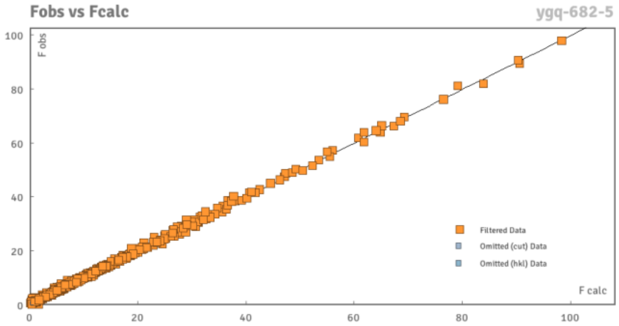

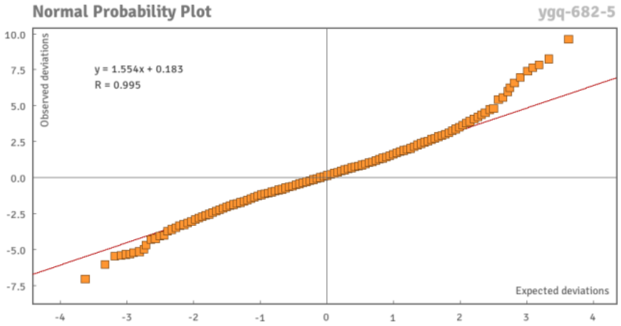

Reflection Statistics

Total reflections (after filtering)	8412	Unique reflections	3500
Completeness	0.985	Mean I/σ	26.55
hkl_{max} collected	(7, 10, 20)	hkl_{min} collected	(-7, -7, -21)
hkl_{max} used	(7, 10, 21)	hkl_{min} used	(-7, -10, 0)
Lim d_{max} collected	100.0	Lim d_{min} collected	0.77
d_{max} used	8.23	d_{min} used	0.79
Friedel pairs	821	Friedel pairs merged	1
Inconsistent equivalents	0	R_{int}	0.0193
R_{sigma}	0.0233	Intensity transformed	0
Omitted reflections	0	Omitted by user (OMIT hkl)	0
Multiplicity	(2115, 1216, 537, 237, 107, 48, 32, 15, 6, 4, 3, 1)	Maximum multiplicity	12
Removed systematic absences	0	Filtered off (Shel/OMIT)	0

Table 15: Fractional Atomic Coordinates (×10⁴) and Equivalent Isotropic Displacement Parameters (Å²×10³) for **ygq-682-5**. U_{eq} is defined as 1/3 of the trace of the orthogonalised U_{ij}.

Atom	x	y	z	U_{eq}
S1	550.3(5)	10473.4(4)	3081.4(2)	31.64(12)
F1	-970.4(14)	7762.3(12)	2507.8(6)	44.3(2)
O1	-1730.5(17)	10636.6(15)	3059.7(7)	41.8(3)
O2	1591(2)	11933.7(13)	3025.1(7)	42.4(3)
N1	1662.2(18)	9540.0(14)	2308.7(7)	29.7(2)
N2	11740(17)	2477(13)	307(6)	55.6(15)
N3	11510(50)	2830(40)	340(20)	68(6)
C1	1142(2)	7878.0(18)	2214.9(9)	33.1(3)
C2	2627(2)	6431.1(18)	2621.7(9)	34.5(3)
C3	4575(3)	6178.7(18)	1988.4(10)	37.6(3)
C4	3989(3)	7469.2(19)	1235.8(9)	39.1(3)
C5	4764(3)	9113.7(19)	1293.9(10)	37.7(3)
C6	3993(2)	9698.8(18)	2103.7(10)	34.8(3)
C7	1539(3)	7705(2)	1321.3(10)	42.5(4)
C8	5180(5)	4424(4)	1818(2)	36.3(7)
C9	7410(4)	4211(3)	1388.1(14)	35.5(6)
C10	7957(3)	2561(2)	1062.6(12)	35.2(5)
C11	10092(5)	2479(4)	633.1(19)	41.7(6)
C12	4436(17)	4470(11)	1667(7)	43(2)
C13	6218(10)	3945(7)	1036(4)	40.3(16)
C14	8357(14)	3593(12)	1394(4)	54(2)
C15	10162(13)	3151(13)	808(5)	44.6(19)
C16	1223(2)	9169.4(16)	3977.7(8)	29.0(3)
C17	-246(2)	8146.7(18)	4384.7(9)	32.9(3)
C18	320(2)	7067.2(18)	5068.4(9)	35.5(3)
C19	2339(3)	6966.3(18)	5347.2(9)	35.0(3)
C20	3779(2)	8015(2)	4928.9(9)	36.5(3)
C21	3244(2)	9116.1(18)	4249.9(9)	33.8(3)
C22	2956(3)	5764(2)	6082.9(11)	48.3(4)

Table 16: Anisotropic Displacement Parameters (×10[4]) for **ygq-682-5**. The anisotropic displacement factor exponent takes the form: $-2\pi^2[h^2a^{*2} \times U_{11}+ ... +2hka^* \times b^* \times U_{12}]$

Atom	U_{11}	U_{22}	U_{33}	U_{23}	U_{13}	U_{12}
S1	31.60(19)	29.02(18)	31.51(18)	-3.80(12)	3.53(13)	1.54(12)
F1	26.8(4)	55.9(6)	52.3(5)	-11.6(4)	2.1(4)	-12.1(4)
O1	31.3(6)	49.2(6)	39.3(6)	-3.7(5)	2.3(4)	9.4(4)
O2	53.8(7)	27.9(5)	42.7(6)	-5.4(4)	8.9(5)	-4.6(4)
N1	26.7(6)	30.6(6)	30.2(6)	-4.4(4)	2.8(4)	-1.6(4)
N2	52(2)	57(4)	44.8(18)	6(2)	12.4(15)	13(2)
N3	61(10)	58(12)	72(10)	20(8)	1(6)	0(8)
C1	26.7(7)	39.3(7)	34.5(7)	-7.9(6)	1.2(5)	-8.0(5)
C2	36.6(8)	30.8(7)	35.4(7)	-5.1(5)	3.9(6)	-6.1(6)
C3	36.8(8)	31.3(7)	42.3(8)	-4.4(6)	7.9(6)	-4.7(6)
C4	44.7(9)	37.8(8)	33.0(7)	-7.7(6)	7.6(6)	-3.8(6)
C5	35.4(8)	35.2(7)	37.9(8)	-0.7(6)	9.1(6)	-1.6(6)
C6	29.0(7)	33.8(7)	40.4(8)	-5.8(6)	6.0(6)	-6.1(5)
C7	45.8(9)	50.4(9)	34.0(8)	-10.9(7)	-4.1(6)	-9.8(7)
C8	34.1(16)	35.5(12)	37.4(13)	-2.3(9)	0.2(11)	-2.7(12)
C9	37.7(13)	27.2(10)	40.4(11)	-5.4(9)	5.5(10)	-4.8(9)
C10	39.0(11)	32.7(10)	33.0(10)	-5.4(8)	-0.8(8)	-1.2(8)
C11	48.7(16)	36.0(14)	35.8(13)	-0.7(11)	0.3(11)	5.0(12)
C12	48(5)	26(3)	51(5)	-12(3)	19(4)	-3(4)
C13	44(3)	35(3)	41(3)	-10(2)	5(3)	-3(2)
C14	62(5)	63(5)	33(3)	-12(3)	9(3)	2(4)

Atom	U_{11}	U_{22}	U_{33}	U_{23}	U_{13}	U_{12}
C15	42(4)	50(5)	36(4)	6(3)	0(3)	0(4)
C16	29.6(7)	28.2(6)	29.2(6)	-7.8(5)	1.8(5)	-1.8(5)
C17	27.3(7)	37.2(7)	34.1(7)	-8.1(6)	3.6(5)	-4.5(5)
C18	36.1(8)	33.9(7)	35.0(7)	-4.3(6)	6.1(6)	-5.5(6)
C19	40.2(8)	32.7(7)	30.0(7)	-8.3(5)	1.3(6)	5.3(6)
C20	30.4(7)	44.8(8)	35.6(7)	-12.3(6)	-3.6(6)	-0.3(6)
C21	32.0(7)	37.1(7)	34.5(7)	-10.4(6)	1.9(6)	-9.8(6)
C22	57.9(11)	43.1(9)	38.5(8)	-2.6(7)	-3.6(7)	11.0(8)

Table 17: Bond Lengths in Å for **ygq-682-5**.

Atom	Atom	Length/Å
S1	O1	1.4332(11)
S1	O2	1.4321(11)
S1	N1	1.6576(12)
S1	C16	1.7596(14)
F1	C1	1.3833(16)
N1	C1	1.4874(18)
N1	C6	1.4948(17)
N2	C11	1.125(8)
N3	C15	1.132(15)
C1	C2	1.537(2)
C1	C7	1.516(2)
C2	C3	1.555(2)
C3	C4	1.560(2)
C3	C8	1.516(4)
C3	C12	1.603(8)
C4	C5	1.522(2)

Atom	Atom	Length/Å
C4	C7	1.530(2)
C5	C6	1.522(2)
C8	C9	1.517(4)
C9	C10	1.533(3)
C10	C11	1.461(4)
C12	C13	1.531(9)
C13	C14	1.509(9)
C14	C15	1.473(11)
C16	C17	1.389(2)
C16	C21	1.391(2)
C17	C18	1.383(2)
C18	C19	1.390(2)
C19	C20	1.396(2)
C19	C22	1.506(2)
C20	C21	1.381(2)

Table 18: Bond Angles in ° for **ygq-682-5**.

Atom	Atom	Atom	Angle/°
O1	S1	N1	108.45(7)
O1	S1	C16	108.26(7)
O2	S1	O1	118.77(7)
O2	S1	N1	105.40(6)
O2	S1	C16	108.32(7)
N1	S1	C16	107.08(6)
C1	N1	S1	121.00(9)
C1	N1	C6	111.50(11)
C6	N1	S1	114.81(9)
F1	C1	N1	107.89(11)
F1	C1	C2	110.93(12)
F1	C1	C7	112.57(12)
N1	C1	C2	114.81(11)
N1	C1	C7	107.59(12)
C7	C1	C2	103.06(13)
C1	C2	C3	105.32(12)
C2	C3	C4	103.78(12)
C2	C3	C12	107.0(4)
C4	C3	C12	102.1(4)
C8	C3	C2	115.39(17)
C8	C3	C4	115.27(17)
C5	C4	C3	110.48(13)
C5	C4	C7	107.74(13)

Atom	Atom	Atom	Angle/°
C7	C4	C3	103.58(12)
C6	C5	C4	111.63(12)
N1	C6	C5	110.96(12)
C1	C7	C4	100.06(12)
C3	C8	C9	111.2(2)
C8	C9	C10	113.6(2)
C11	C10	C9	110.2(2)
N2	C11	C10	177.3(7)
C13	C12	C3	116.7(7)
C14	C13	C12	111.1(7)
C15	C14	C13	113.9(6)
N3	C15	C14	178(2)
C17	C16	S1	119.23(11)
C17	C16	C21	120.77(13)
C21	C16	S1	119.93(11)
C18	C17	C16	119.23(14)
C17	C18	C19	121.32(14)
C18	C19	C20	118.20(14)
C18	C19	C22	120.96(15)
C20	C19	C22	120.84(15)
C21	C20	C19	121.58(14)
C20	C21	C16	118.89(13)

Table 19: Torsion Angles in ° for **ygq-682-5**.

Atom	Atom	Atom	Atom	Angle/°
S1	N1	C1	F1	33.16(15)
S1	N1	C1	C2	-91.09(13)
S1	N1	C1	C7	154.85(11)
S1	N1	C6	C5	-168.58(10)
S1	C16	C17	C18	177.06(11)
S1	C16	C21	C20	-176.33(11)
F1	C1	C2	C3	152.08(12)
F1	C1	C7	C4	-167.17(12)
O1	S1	N1	C1	-58.10(12)
O1	S1	N1	C6	163.42(10)
O1	S1	C16	C17	18.54(13)
O1	S1	C16	C21	-164.42(11)
O2	S1	N1	C1	173.72(11)
O2	S1	N1	C6	35.24(11)
O2	S1	C16	C17	148.57(11)
O2	S1	C16	C21	-34.39(13)
N1	S1	C16	C17	-98.20(12)
N1	S1	C16	C21	78.84(12)
N1	C1	C2	C3	-85.29(14)
N1	C1	C7	C4	74.09(14)
C1	N1	C6	C5	49.06(16)
C1	C2	C3	C4	-2.81(15)
C1	C2	C3	C8	-129.89(18)
C1	C2	C3	C12	-110.3(5)
C2	C1	C7	C4	-47.60(14)
C2	C3	C4	C5	88.67(14)
C2	C3	C4	C7	-26.47(15)
C2	C3	C8	C9	-164.11(19)
C2	C3	C12	C13	-179.5(7)
C3	C4	C5	C6	-52.40(17)
C3	C4	C7	C1	45.86(15)
C3	C8	C9	C10	-169.6(2)
C3	C12	C13	C14	65.2(10)
C4	C3	C8	C9	74.9(3)
C4	C3	C12	C13	71.9(8)
C4	C5	C6	N1	-46.66(17)
C5	C4	C7	C1	-71.21(15)
C6	N1	C1	F1	172.86(11)
C6	N1	C1	C2	48.61(16)
C6	N1	C1	C7	-65.44(15)
C7	C1	C2	C3	31.38(15)
C7	C4	C5	C6	60.09(17)
C8	C3	C4	C5	-144.18(18)
C8	C3	C4	C7	100.68(19)
C8	C9	C10	C11	177.9(2)
C12	C3	C4	C5	-160.2(4)
C12	C3	C4	C7	84.7(4)
C12	C13	C14	C15	-177.4(8)
C16	S1	N1	C1	58.52(12)
C16	S1	N1	C6	-79.96(11)
C16	C17	C18	C19	-1.1(2)
C17	C16	C21	C20	0.7(2)
C17	C18	C19	C20	1.4(2)
C17	C18	C19	C22	-178.83(14)
C18	C19	C20	C21	-0.7(2)

Atom	Atom	Atom	Atom	Angle/°
C19	C20	C21	C16	-0.3(2)
C21	C16	C17	C18	0.0(2)
C22	C19	C20	C21	179.56(14)

Table 20: Hydrogen Fractional Atomic Coordinates (×10[4]) and Equivalent Isotropic Displacement Parameters (Å[2]×10[3]) for **ygq-682-5**. U_{eq} is defined as 1/3 of the trace of the orthogonalised U_{ij}.

Atom	x	y	z	U_{eq}
H2A	3050(30)	6640(20)	3142(11)	35(4)
H2B	1850(30)	5420(30)	2739(12)	47(5)
H3A	5839.38	6524.64	2205.32	45
H3B	5988.34	6269.58	2188.8	45
H4	4500(30)	7060(20)	730(12)	43(5)
H5A	4200(30)	9940(30)	840(13)	46(5)
H5B	6330(30)	9020(20)	1242(13)	47(5)
H6A	4840(30)	9040(20)	2530(13)	43(5)
H6B	4160(30)	10870(30)	2092(12)	42(5)
H7A	890(30)	8610(20)	1015(12)	37(5)
H7B	960(30)	6720(30)	1243(13)	50(5)
H8A	4131.46	4140.79	1475.21	44
H8B	5125.74	3658.97	2336.12	44
H9A	8471.05	4301.74	1771.31	43
H9B	7536.42	5118.91	929.48	43
H10A	7906.46	1643.54	1519.53	42
H10B	6885.84	2444.1	688.42	42
H12A	4427.06	3587.37	2141.22	51
H12B	3047.53	4545.89	1429.42	51
H13A	5881.76	2944.28	840.27	48
H13B	6291.74	4832.23	565.83	48
H14A	8639.35	4579.39	1616.1	64
H14B	8281.36	2677.65	1851.12	64
H17	-1640(30)	8220(20)	4206(12)	41(5)
H18	-740(40)	6390(30)	5355(13)	54(6)
H20	5130(30)	7970(30)	5123(13)	51(6)
H21	4230(30)	9800(30)	3976(13)	48(5)
H22A	2076.95	6072.14	6565.09	58
H22B	4471.65	5788.09	6155.5	58
H22C	2720.94	4650.82	6006.91	58
H22D	4102.74	4935.23	5919.91	58
H22E	1708.04	5219.28	6329.5	58
H22F	3458.75	6356.55	6478.09	58

Table 21: Atomic Occupancies for all atoms that are not fully occupied in **ygq-682-5**.

Atom	Occupancy	Atom	Occupancy	Atom	Occupancy
N2	0.741(4)	H9B	0.741(4)	H13A	0.259(4)
N3	0.259(4)	C10	0.741(4)	H13B	0.259(4)
H3A	0.741(4)	H10A	0.741(4)	C14	0.259(4)
H3B	0.259(4)	H10B	0.741(4)	H14A	0.259(4)
C8	0.741(4)	C11	0.741(4)	H14B	0.259(4)
H8A	0.741(4)	C12	0.259(4)	C15	0.259(4)
H8B	0.741(4)	H12A	0.259(4)	H22A	0.45(2)
C9	0.741(4)	H12B	0.259(4)	H22B	0.45(2)
H9A	0.741(4)	C13	0.259(4)	H22C	0.45(2)

Atom	Occupancy	Atom	Occupancy	Atom	Occupancy
H22D	0.55(2)	H22E	0.55(2)	H22F	0.55(2)

Citations

CrysAlisPro (ROD), Rigaku Oxford Diffraction, Poland (?).

CrysAlisPro Software System, Rigaku Oxford Diffraction, (2024).

O.V. Dolomanov and L.J. Bourhis and R.J. Gildea and J.A.K. Howard and H. Puschmann, Olex2: A complete structure solution, refinement and analysis program, *J. Appl. Cryst.*, (2009), **42**, 339-341.

Sheldrick, G.M., Crystal structure refinement with ShelXL, *Acta Cryst.*, (2015), **C71**, 3-8.

Sheldrick, G.M., ShelXT-Integrated space-group and crystal-structure determination, *Acta Cryst.*, (2015), **A71**, 3-8.

Crystal Data and Experimental of 1-fluoro-2-tosyldecahydro-1,5-methanocyclopenta[c]azepine (5y) (CCDC: 2417052)

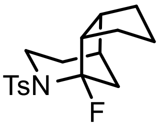

5y

R₁=2.80%

Crystal Data and Experimental

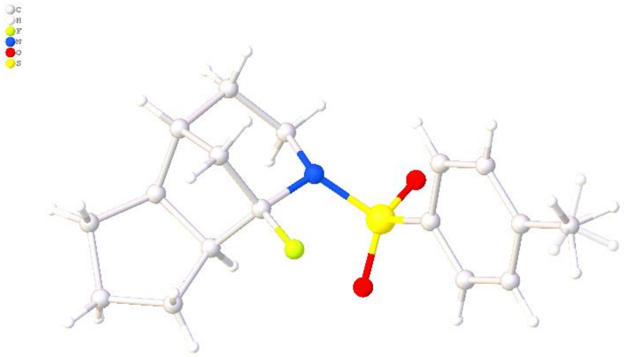

Experimental. Single clear pale colourless irregular-shaped crystals of **ygq-679-3** were used as supplied. A suitable crystal with dimensions 0.25 × 0.19 × 0.13 mm^3 was selected and mounted on a XtaLAB Synergy R, DW system, HyPix-Arc 150 diffractometer. The crystal was kept at a steady T = 140.00(10) K during data collection. The structure was solved with the ShelXS (Sheldrick, 2008) solution program using direct methods and by using Olex2 1.5 (Dolomanov et al., 2009) as the graphical interface. The model was refined with ShelXL 2019/3 (Sheldrick, 2015) using full matrix least squares minimisation on $\boldsymbol{F}^2$.

Crystal Data. $C_{17}H_{22}FNO_2S$, M_r = 323.41, triclinic, P-1 (No. 2), a = 8.55615(15) Å, b = 8.74496(17) Å, c = 11.0725(3) Å, α = 96.6163(18)°, β = 104.0129(19)°, γ = 101.7890(16)°, V = 774.95(3) Å^3, T = 140.00(10) K, Z = 2, Z' = 1, μ(Cu K$_\alpha$) = 2.011, 16699 reflections measured, 3121 unique (R_{int} = 0.0140) which were used in all calculations. The final wR_2 was 0.0783 (all data) and R_1 was 0.0280 (I≥2 σ(I)).

**Compound**	**ygq-679-3**
Formula	$C_{17}H_{22}FNO_2S$
$D_{calc.}$/ g cm^{-3}	1.386
μ/mm^{-1}	2.011
Formula Weight	323.41
Colour	clear pale colourless
Shape	irregular-shaped
Size/mm^3	0.25×0.19×0.13
T/K	140.00(10)
Crystal System	triclinic
Space Group	P-1
a/Å	8.55615(15)
b/Å	8.74496(17)
c/Å	11.0725(3)
α/°	96.6163(18)
β/°	104.0129(19)
γ/°	101.7890(16)
V/Å^3	774.95(3)
Z	2
Z'	1
Wavelength/Å	1.54184
Radiation type	Cu K$_\alpha$
Θ_{min}/°	4.179
Θ_{max}/°	75.436
Measured Refl's.	16699
Indep't Refl's	3121
Refl's I≥2 σ(I)	2973
R_{int}	0.0140
Parameters	301
Restraints	21
Largest Peak	0.296
Deepest Hole	-0.345
GooF	1.094
wR_2 (all data)	0.0783
wR_2	0.0775
R_1 (all data)	0.0291
R_1	0.0280

Structure Quality Indicators

**Reflections:**	d min (CuKα) 2Θ=150.9° 0.80	I/σ(I) 100.0	Rint m=5.35 1.40%	Full 135.4° 97% to 150.9° 99.3
**Refinement:**	Shift 0.001	Max Peak 0.3	Min Peak -0.3	GooF 1.094

A clear pale colourless irregular-shaped-shaped crystal with dimensions 0.25 × 0.19 × 0.13 mm^3 was mounted. Data were collected using a XtaLAB Synergy R, DW system, HyPix-Arc 150 diffractometer operating at T = 140.00(10) K.

Data were measured using ω scans with Cu K_α radiation. The diffraction pattern was indexed and the total number of runs and images was based on the strategy calculation from the program CrysAlisPro system (CCD 43.143a 64-bit (release 25-10-2024)). The maximum resolution that was achieved was Θ = 75.436° (0.80 Å).

The unit cell was refined using CrysAlisPro 1.171.43.143a (Rigaku OD, 2024) on 13378 reflections, 80% of the observed reflections.

Data reduction, scaling and absorption corrections were performed using CrysAlisPro 1.171.43.143a (Rigaku OD, 2024). The final completeness is 99.30 % out to 75.436° in Θ. A gaussian absorption correction was performed using CrysAlisPro 1.171.43.143a (Rigaku Oxford Diffraction, 2024) Numerical absorption correction based on gaussian integration over a multifaceted crystal model Empirical absorption correction using spherical harmonics, implemented in SCALE3 ABSPACK scaling algorithm.. The absorption coefficient μ of this material is 2.011 mm^{-1} at this wavelength (λ = 1.54184Å) and the minimum and maximum transmissions are 0.513 and 1.000.

The structure was solved and the space group P-1 (# 2) determined by the ShelXS (Sheldrick, 2008) structure solution program using using direct methods and refined by full matrix least squares minimisation on F^2 using version 2019/3 of ShelXL 2019/3 (Sheldrick, 2015). All non-hydrogen atoms were refined anisotropically. Hydrogen atom positions were calculated geometrically and refined using the riding model.

_exptl_absorpt_process_details: CrysAlisPro 1.171.43.143a (Rigaku Oxford Diffraction, 2024)Numerical absorption correction based on gaussian integration overa multifaceted crystal modelEmpirical absorption correction using spherical harmonics,implemented in SCALE3 ABSPACK scaling algorithm.

There is a single formula unit in the asymmetric unit, which is represented by the reported sum formula. In other words: Z is 2 and Z' is 1. The moiety formula is C17 H22 F N O2 S.

Data Plots: Diffraction Data

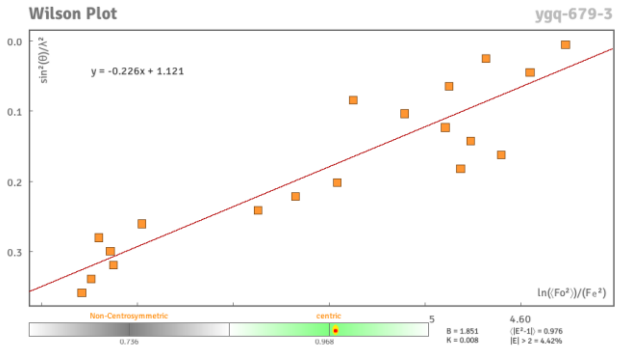

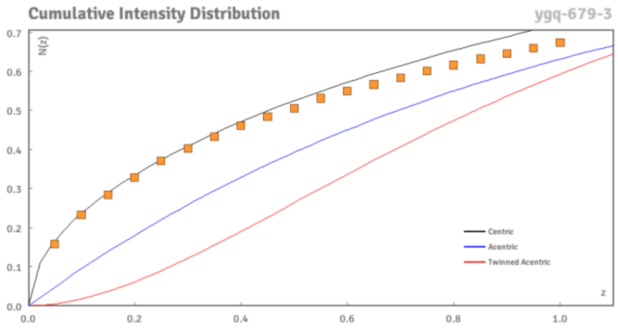

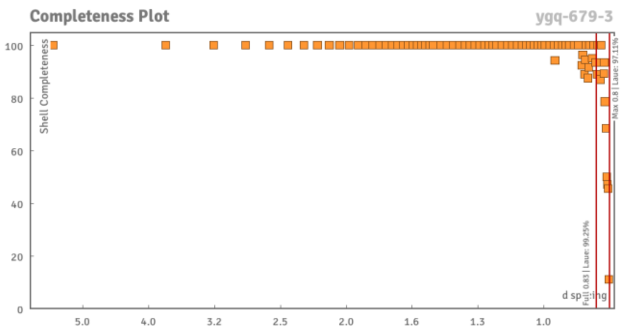

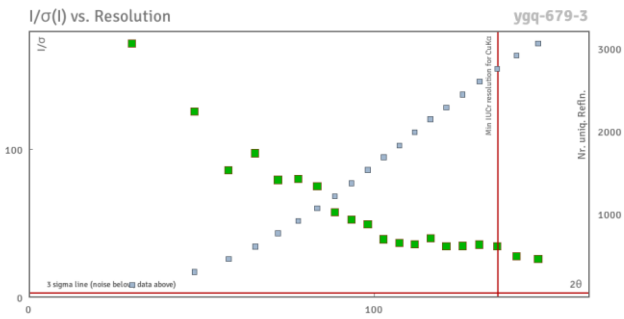

Data Plots: Refinement and Data

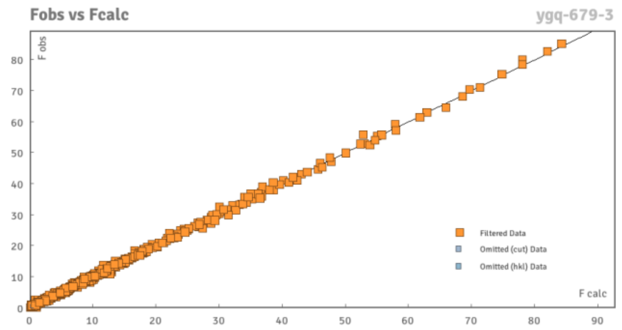

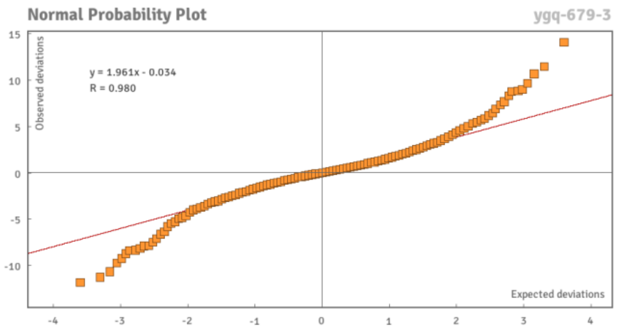

Reflection Statistics

Total reflections (after filtering)	16699	Unique reflections	3121
Completeness	0.971	Mean I/σ	61.65
hkl$_{max}$ collected	(10, 10, 13)	hkl$_{min}$ collected	(-10, -9, -13)
hkl$_{max}$ used	(10, 10, 13)	hkl$_{min}$ used	(-10, -10, 0)
Lim d$_{max}$ collected	100.0	Lim d$_{min}$ collected	0.77
d$_{max}$ used	10.58	d$_{min}$ used	0.8
Friedel pairs	1598	Friedel pairs merged	1
Inconsistent equivalents	1	R$_{int}$	0.014
R$_{sigma}$	0.01	Intensity transformed	0
Omitted reflections	0	Omitted by user (OMIT hkl)	0
Multiplicity	(1215, 1065, 832, 523, 329, 223, 133, 82, 56, 46, 33, 39, 28, 16, 19, 12, 12, 13, 14, 9, 4, 10, 1, 1, 3, 1)	Maximum multiplicity	26
Removed systematic absences	0	Filtered off (Shel/OMIT)	0

Table 22: Fractional Atomic Coordinates (×10^4) and Equivalent Isotropic Displacement Parameters (Å^2×10^3) for **ygq-679-3**. U_{eq} is defined as 1/3 of the trace of the orthogonalised U_{ij}.

Atom	x	y	z	U_{eq}
S1	8012.0(4)	2307.5(3)	7890.9(3)	23.91(11)
F1	9077.4(8)	423.0(9)	6167.1(6)	26.54(18)
O1	8040.9(13)	3636.9(12)	8794.4(9)	35.5(2)
O2	7134.5(11)	763.9(11)	7999.6(9)	30.0(2)
N1	9976.4(12)	2382.6(12)	7930.9(9)	22.5(2)
C1	10334.5(14)	960.4(14)	7289.1(11)	21.4(2)
C2	12020.0(15)	1456.8(16)	7045.3(12)	25.8(3)
C3	13143.7(15)	1672.8(16)	8391.4(12)	26.6(3)
C4	12940.1(16)	3164.5(16)	9142.8(13)	29.4(3)
C5	11159.8(16)	3019.1(16)	9212.8(12)	26.1(3)

Atom	x	y	z	U_{eq}
C6	10518.1(15)	-332.3(14)	8104.3(12)	22.9(3)
C7	10231.7(17)	-2002.4(16)	7345.4(14)	30.2(3)
C8	11571.1(18)	-2689.5(16)	8121.6(14)	32.2(3)
C9	13086.8(17)	-1289.7(17)	8521.5(14)	31.2(3)
C10	12402.6(15)	154.6(15)	8854.6(12)	24.7(3)
C11	7148.2(15)	2691.0(14)	6376.9(11)	22.2(2)
C12	8004.9(16)	3885.6(15)	5886.0(13)	26.9(3)
C13	7215.3(16)	4260.5(16)	4750.0(13)	28.6(3)
C14	5586.8(16)	3462.9(15)	4093.8(12)	25.7(3)
C15	4762.8(16)	2274.0(16)	4609.1(13)	27.7(3)
C16	5534.2(15)	1885.2(15)	5747.9(12)	25.5(3)
C17	4736.1(18)	3873.0(19)	2861.5(14)	34.4(3)

Table 23: Anisotropic Displacement Parameters (×10^4) for **ygq-679-3.** The anisotropic displacement factor exponent takes the form: $-2\pi^2[h^2a^{*2} \times U_{11}+ ... +2hka^* \times b^* \times U_{12}]$

Atom	U_{11}	U_{22}	U_{33}	U_{23}	U_{13}	U_{12}
S1	24.73(16)	27.37(18)	20.86(16)	2.74(11)	6.44(11)	9.53(12)
F1	22.8(3)	31.6(4)	20.3(3)	-2.8(3)	-0.6(3)	7.5(3)
O1	40.5(5)	40.9(6)	25.7(5)	-4.0(4)	6.5(4)	19.5(4)
O2	26.4(4)	35.9(5)	31.6(5)	13.1(4)	10.9(4)	8.4(4)
N1	22.0(5)	23.5(5)	19.4(5)	0.2(4)	2.2(4)	5.6(4)
C1	19.2(5)	24.4(6)	17.8(5)	0.3(4)	2.1(4)	4.2(4)
C2	23.1(6)	32.1(7)	22.6(6)	6.6(5)	7.3(5)	5.2(5)
C3	18.4(6)	32.7(7)	25.8(6)	5.3(5)	3.4(5)	2.9(5)
C4	25.8(6)	27.3(7)	27.0(7)	3.5(5)	-0.6(5)	-1.1(5)
C5	28.8(6)	24.1(6)	20.1(6)	-1.3(5)	0.8(5)	4.2(5)
C6	20.9(6)	23.8(6)	24.5(6)	4.0(5)	6.8(5)	5.7(4)
C7	25.2(6)	24.2(6)	37.7(8)	0.0(5)	5.2(5)	5.6(5)
C8	34.3(7)	27.4(7)	37.1(7)	5.0(6)	10.0(6)	12.6(5)
C9	26.8(6)	34.1(7)	32.7(7)	4.9(6)	3.9(6)	13.2(5)
C10	22.7(6)	28.9(6)	21.9(6)	4.8(5)	3.8(5)	7.3(5)
C11	23.4(6)	21.3(6)	22.6(6)	1.8(4)	5.7(5)	8.6(5)
C12	23.1(6)	24.5(6)	30.6(7)	3.5(5)	5.0(5)	3.4(5)
C13	28.4(6)	26.9(6)	32.4(7)	8.9(5)	10.0(5)	6.6(5)
C14	27.2(6)	28.7(6)	24.9(6)	5.0(5)	8.3(5)	13.2(5)
C15	22.1(6)	29.4(6)	28.8(6)	2.9(5)	3.2(5)	5.9(5)
C16	23.3(6)	24.2(6)	29.0(6)	4.9(5)	7.4(5)	5.1(5)
C17	34.2(7)	45.8(8)	29.2(7)	12.6(6)	8.8(6)	19.4(6)

Table 24: Bond Lengths in Å for **ygq-679-3**.

Atom	Atom	Length/Å
S1	O1	1.4357(10)
S1	O2	1.4378(10)
S1	N1	1.6581(10)
S1	C11	1.7606(12)
F1	C1	1.3903(13)
N1	C1	1.4843(15)
N1	C5	1.4971(15)
C1	C2	1.5169(16)
C1	C6	1.5350(17)
C2	C3	1.5325(17)

Atom	Atom	Length/Å
C3	C4	1.5288(19)
C3	C10	1.5486(17)
C4	C5	1.5240(18)
C6	C7	1.5396(17)
C6	C10	1.5686(16)
C7	C8	1.5285(19)
C8	C9	1.525(2)
C9	C10	1.5406(18)
C11	C12	1.3910(17)
C11	C16	1.3829(17)
C12	C13	1.3856(19)

Atom	Atom	Length/Å
C13	C14	1.3970(19)
C14	C15	1.3912(18)
C14	C17	1.5029(18)
C15	C16	1.3887(18)

Table 25: Bond Angles in ° for **ygq-679-3**.

Atom	Atom	Atom	Angle/°
O1	S1	O2	117.77(6)
O1	S1	N1	106.05(6)
O1	S1	C11	107.42(6)
O2	S1	N1	112.35(5)
O2	S1	C11	108.43(6)
N1	S1	C11	103.84(5)
C1	N1	S1	117.13(8)
C1	N1	C5	112.50(9)
C5	N1	S1	113.62(8)
F1	C1	N1	106.45(9)
F1	C1	C2	111.56(9)
F1	C1	C6	112.68(9)
N1	C1	C2	108.06(10)
N1	C1	C6	113.82(10)
C2	C1	C6	104.27(10)
C1	C2	C3	99.88(9)
C2	C3	C10	102.54(10)
C4	C3	C2	107.30(11)
C4	C3	C10	111.65(10)
C5	C4	C3	112.09(10)
N1	C5	C4	110.08(10)
C1	C6	C7	114.30(10)
C1	C6	C10	103.46(9)
C7	C6	C10	105.72(10)
C8	C7	C6	103.69(11)
C9	C8	C7	102.91(11)
C8	C9	C10	104.61(11)
C3	C10	C6	105.28(10)
C9	C10	C3	114.80(11)
C9	C10	C6	105.11(10)
C12	C11	S1	120.70(9)
C16	C11	S1	118.08(10)
C16	C11	C12	120.90(12)
C13	C12	C11	118.93(12)
C12	C13	C14	121.34(12)
C13	C14	C17	121.14(12)
C15	C14	C13	118.38(12)
C15	C14	C17	120.48(12)
C16	C15	C14	121.04(12)
C11	C16	C15	119.41(12)

Table 26: Torsion Angles in ° for **ygq-679-3**.

Atom	Atom	Atom	Atom	Angle/°
S1	N1	C1	F1	40.92(11)
S1	N1	C1	C2	160.88(8)
S1	N1	C1	C6	-83.82(11)
S1	N1	C5	C4	-175.71(8)
S1	C11	C12	C13	-173.39(10)
S1	C11	C16	C15	173.69(10)
F1	C1	C2	C3	-169.79(10)
F1	C1	C6	C7	36.78(14)
F1	C1	C6	C10	151.21(9)
O1	S1	N1	C1	167.36(8)
O1	S1	N1	C5	33.47(10)
O1	S1	C11	C12	68.79(11)
O1	S1	C11	C16	-104.75(11)
O2	S1	N1	C1	37.39(10)
O2	S1	N1	C5	-96.50(9)
O2	S1	C11	C12	-162.95(10)
O2	S1	C11	C16	23.51(11)
N1	S1	C11	C12	-43.28(11)
N1	S1	C11	C16	143.18(10)
N1	C1	C2	C3	73.52(12)
N1	C1	C6	C7	158.11(10)
N1	C1	C6	C10	-87.45(11)
C1	N1	C5	C4	48.26(13)
C1	C2	C3	C4	-71.29(12)

Atom	Atom	Atom	Atom	Angle/˚
C1	C2	C3	C10	46.43(12)
C1	C6	C7	C8	139.57(11)
C1	C6	C10	C3	-0.76(12)
C1	C6	C10	C9	-122.37(11)
C2	C1	C6	C7	-84.37(12)
C2	C1	C6	C10	30.06(12)
C2	C3	C4	C5	61.11(14)
C2	C3	C10	C6	-28.25(12)
C2	C3	C10	C9	86.84(12)
C3	C4	C5	N1	-46.74(14)
C4	C3	C10	C6	86.33(12)
C4	C3	C10	C9	-158.58(11)
C5	N1	C1	F1	175.30(9)
C5	N1	C1	C2	-64.74(12)
C5	N1	C1	C6	50.56(13)
C6	C1	C2	C3	-47.90(12)
C6	C7	C8	C9	-41.29(14)
C7	C6	C10	C3	119.70(11)
C7	C6	C10	C9	-1.91(13)
C7	C8	C9	C10	40.33(14)
C8	C9	C10	C3	-138.69(11)
C8	C9	C10	C6	-23.51(14)
C10	C3	C4	C5	-50.50(14)
C10	C6	C7	C8	26.48(13)
C11	S1	N1	C1	-79.58(9)
C11	S1	N1	C5	146.53(9)
C11	C12	C13	C14	-0.1(2)
C12	C11	C16	C15	0.16(19)
C12	C13	C14	C15	0.1(2)
C12	C13	C14	C17	-179.96(12)
C13	C14	C15	C16	0.01(19)
C14	C15	C16	C11	-0.15(19)
C16	C11	C12	C13	-0.04(19)
C17	C14	C15	C16	-179.91(12)

Table 27: Hydrogen Fractional Atomic Coordinates (×10[4]) and Equivalent Isotropic Displacement Parameters (Å[2]×10[3]) for **ygq-679-3**. U_{eq} is defined as 1/3 of the trace of the orthogonalised U_{ij}.

Atom	x	y	z	U_{eq}
H2A	12156(19)	594(19)	6489(16)	31(4)
H2B	12139(19)	2416(19)	6648(15)	32(4)
H3	14300(20)	1753(18)	8425(15)	32(4)
H4A	13630(20)	3356(19)	10019(16)	36(4)
H4B	13280(20)	4070(20)	8758(16)	40(4)
H5A	10933(18)	2331(18)	9836(15)	26(4)
H5B	11010(20)	4010(20)	9492(15)	33(4)
H6	9790(19)	-342(17)	8677(15)	27(4)
H7A	9120(20)	-2620(20)	7219(16)	36(4)
H7B	10430(20)	-1898(19)	6498(17)	37(4)
H8A	11750(20)	-3624(19)	7632(15)	33(4)
H8B	11260(20)	-3000(20)	8870(17)	36(4)
H9A	13570(20)	-1170(20)	7804(17)	40(4)
H9B	13970(20)	-1400(20)	9225(17)	40(4)
H10	12544(18)	384(17)	9782(15)	28(4)
H12	9110(20)	4450(19)	6339(15)	33(4)
H13	7820(20)	5080(20)	4408(17)	40(4)

Atom	x	y	z	U_{eq}
H15	3660(20)	1698(19)	4172(15)	33(4)
H16	4970(20)	1045(19)	6099(15)	32(4)
H17A	5350(50)	4900(40)	2720(50)	55(17)
H17B	3620(30)	4010(50)	2840(40)	26(13)
H17C	4610(60)	3100(50)	2110(40)	70(20)
H17D	5510(30)	4060(30)	2330(20)	29(9)
H17E	3820(30)	2950(20)	2390(20)	27(9)
H17F	4290(40)	4800(30)	2990(30)	42(10)

Table 28: Atomic Occupancies for all atoms that are not fully occupied in **ygq-679-3**.

Atom	Occupancy	Atom	Occupancy	Atom	Occupancy
H17A	0.40(5)	H17C	0.40(5)	H17E	0.60(5)
H17B	0.40(5)	H17D	0.60(5)	H17F	0.60(5)

Citations

CrysAlisPro (ROD), Rigaku Oxford Diffraction, Poland (?).

CrysAlisPro Software System, Rigaku Oxford Diffraction, (2024).

O.V. Dolomanov and L.J. Bourhis and R.J. Gildea and J.A.K. Howard and H. Puschmann, Olex2: A complete structure solution, refinement and analysis program, *J. Appl. Cryst.*, (2009), **42**, 339-341.

Sheldrick, G.M., A short history of ShelX, *Acta Cryst.*, (2008), **A64**, 339-341.

Sheldrick, G.M., Crystal structure refinement with ShelXL, *Acta Cryst.*, (2015), **C71**, 3-8.

Crystal Data and Experimental of 2-fluoro-1-tosyldecahydro-1*H*-2,5-methanobenzo[b]azepine (5aa) (CCDC: 2417053)

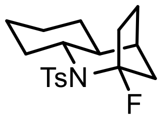

5aa

R_1=3.00%

Crystal Data and Experimental

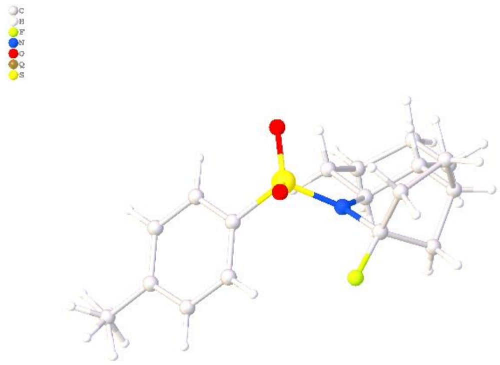

**Compound**	**ygq-706-2**
Formula	$C_{18}H_{24}FNO_2S$
$D_{calc.}$/ g cm^{-3}	1.358
μ/mm^{-1}	1.911
Formula Weight	337.44
Colour	clear pale colourless
Shape	prism-shaped
Size/mm^3	0.42×0.15×0.13
T/K	140.00(10)
Crystal System	monoclinic
Space Group	$P2_1/c$
a/Å	12.32328(13)
b/Å	11.14786(13)
c/Å	12.03217(12)
α/°	90
β/°	93.3816(9)
γ/°	90
V/Å^3	1650.08(3)
Z	4
Z'	1
Wavelength/Å	1.54184
Radiation type	Cu K$_\alpha$
Θ_{min}/°	3.593
Θ_{max}/°	75.443
Measured Refl's.	18370
Indep't Refl's	3392
Refl's I≥2 σ(I)	3160
R_{int}	0.0192
Parameters	295
Restraints	0
Largest Peak	0.325
Deepest Hole	-0.409
GooF	1.048
wR_2 (all data)	0.0841
wR_2	0.0827
R_1 (all data)	0.0320
R_1	0.0300

Experimental. Single clear pale colourless prism-shaped crystals of **ygq-706-2** were used as supplied. A suitable crystal with dimensions 0.42 × 0.15 × 0.13 mm^3 was selected and mounted on a XtaLAB Synergy R, DW system, HyPix-Arc 150 diffractometer. The crystal was kept at a steady T = 140.00(10) K during data collection. The structure was solved with the ShelXT (Sheldrick, 2015) solution program using dual methods and by using Olex2 1.5 (Dolomanov et al., 2009) as the graphical interface. The model was refined with ShelXL 2019/3 (Sheldrick, 2015) using full matrix least squares minimisation on ***F*²**.

Crystal Data. $C_{18}H_{24}FNO_2S$, M_r = 337.44, monoclinic, $P2_1/c$ (No. 14), a = 12.32328(13) Å, b = 11.14786(13) Å, c = 12.03217(12) Å, β = 93.3816(9)°, α = γ = 90°, V = 1650.08(3) Å^3, T = 140.00(10) K, Z = 4, Z' = 1, μ(Cu K$_\alpha$) = 1.911, 18370 reflections measured, 3392 unique (R_{int} = 0.0192) which were used in all calculations. The final wR_2 was 0.0841 (all data) and R_1 was 0.0300 (I≥2 σ(I)).

Structure Quality Indicators

**Reflections:**	d min (CuKα) 2Θ=150.9° 0.80	I/σ(I) 75.1	Rint m=5.61 1.92%	Full 135.4° 99% to 150.9° 100
**Refinement:**	Shift 0.000	Max Peak 0.3	Min Peak -0.4	GooF 1.048

A clear pale colourless prism-shaped-shaped crystal with dimensions 0.42 × 0.15 × 0.13 mm^3 was mounted. Data were collected using a XtaLAB Synergy R, DW system, HyPix-Arc 150 diffractometer operating at T = 140.00(10) K.

Data were measured using ω scans with Cu K_α radiation. The diffraction pattern was indexed and the total number of runs and images was based on the strategy calculation from the program CrysAlisPro system (CCD 43.143a 64-bit (release 25-10-2024)). The maximum resolution that was achieved was Θ = 75.443° (0.80 Å).

The unit cell was refined using CrysAlisPro 1.171.43.143a (Rigaku OD, 2024) on 13993 reflections, 76% of the observed reflections.

Data reduction, scaling and absorption corrections were performed using CrysAlisPro 1.171.43.143a (Rigaku OD, 2024). The final completeness is 100.00 % out to 75.443° in Θ. A gaussian absorption correction was performed using CrysAlisPro 1.171.43.143a (Rigaku Oxford Diffraction, 2024) Numerical absorption correction based on gaussian integration over a multifaceted crystal model Empirical absorption correction using spherical harmonics, implemented in SCALE3 ABSPACK scaling algorithm.. The absorption coefficient μ of this material is 1.911 mm^{-1} at this wavelength (λ = 1.54184Å) and the minimum and maximum transmissions are 0.445 and 1.000.

The structure was solved and the space group $P2_1/c$ (# 14) determined by the ShelXT (Sheldrick, 2015) structure solution program using using dual methods and refined by full matrix least squares minimisation on $\boldsymbol{F}^2$ using version 2019/3 of ShelXL 2019/3 (Sheldrick, 2015). All non-hydrogen atoms were refined anisotropically. Hydrogen atom positions were calculated geometrically and refined using the riding model. Most hydrogen atom positions were calculated geometrically and refined using the riding model, but some hydrogen atoms were refined freely.

_exptl_absorpt_process_details: CrysAlisPro 1.171.43.143a (Rigaku Oxford Diffraction, 2024)Numerical absorption correction based on gaussian integration overa multifaceted crystal modelEmpirical absorption correction using spherical harmonics,implemented in SCALE3 ABSPACK scaling algorithm.

There is a single formula unit in the asymmetric unit, which is represented by the reported sum formula. In other words: Z is 4 and Z' is 1. The moiety formula is C18 H24 F N O2 S.

Data Plots: Diffraction Data

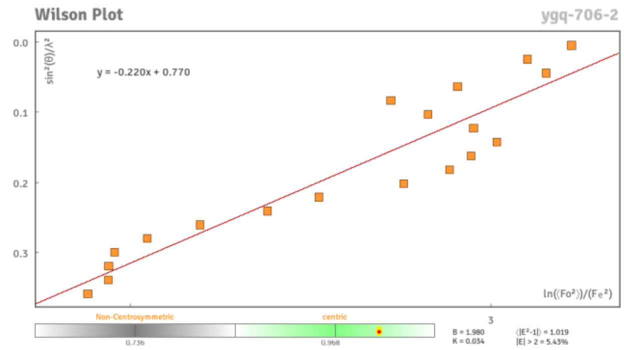

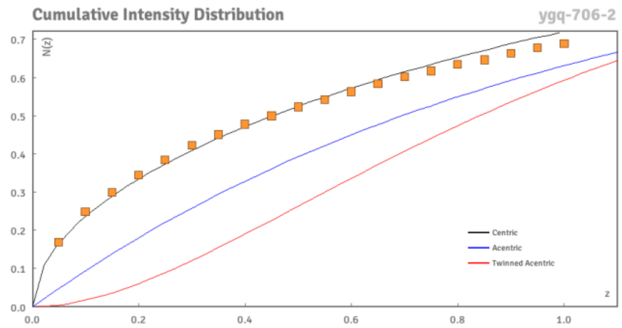

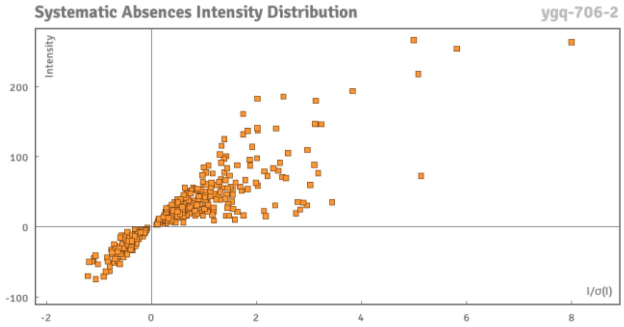

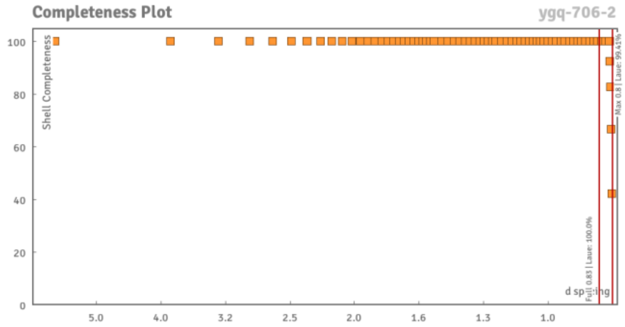

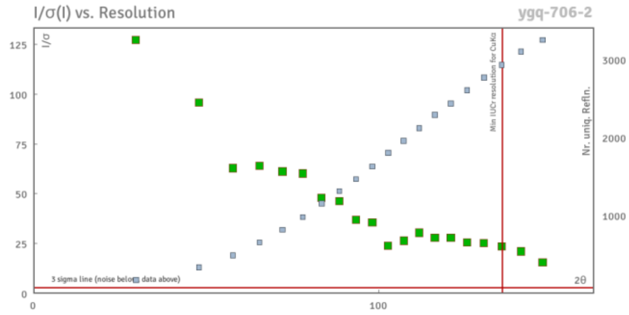

Data Plots: Refinement and Data

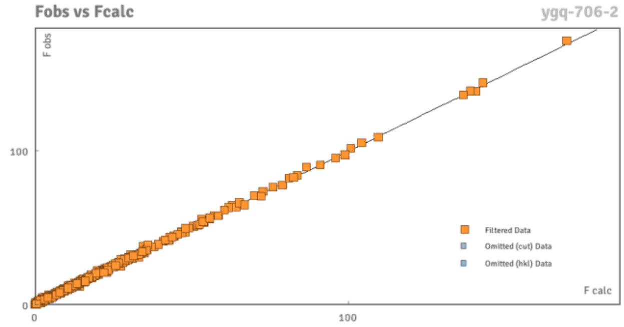

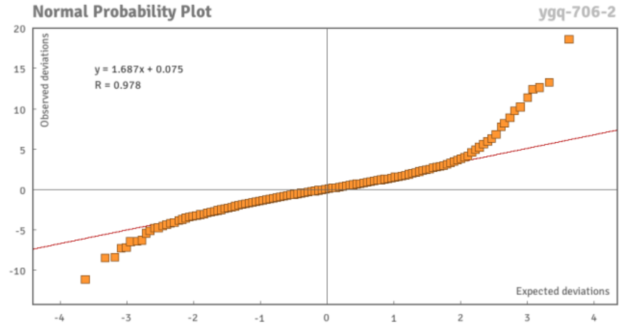

Reflection Statistics

Total reflections (after filtering)	19026	Unique reflections	3392
Completeness	0.994	Mean I/σ	44.46
hkl_{max} collected	(15, 13, 15)	hkl_{min} collected	(-15, -13, -14)
hkl_{max} used	(15, 13, 15)	hkl_{min} used	(-15, 0, 0)
Lim d_{max} collected	100.0	Lim d_{min} collected	0.77

d_{max} used	12.3	d_{min} used	0.8
Friedel pairs	3040	Friedel pairs merged	1
Inconsistent equivalents	1	R_{int}	0.0192
R_{sigma}	0.0133	Intensity transformed	0
Omitted reflections	0	Omitted by user (OMIT hkl)	0
Multiplicity	(3534, 2360, 1521, 863, 371, 94, 38, 9)	Maximum multiplicity	19
Removed systematic absences	656	Filtered off (Shel/OMIT)	0

Table 29: Fractional Atomic Coordinates (×10^4) and Equivalent Isotropic Displacement Parameters (Å^2×10^3) for **ygq-706-2.** U_{eq} is defined as 1/3 of the trace of the orthogonalised U_{ij}.

Atom	x	y	z	U_{eq}
S1	7654.7(2)	4068.5(3)	4482.5(2)	23.35(11)
F1	9631.4(6)	2530.3(8)	5334.5(7)	39.7(2)
O1	8553.6(8)	4085.3(8)	3779.5(8)	30.4(2)
O2	6579.1(8)	4179.2(9)	3974.8(8)	31.7(2)
N1	7758.8(8)	2830.6(9)	5213.5(8)	24.0(2)
C1	8642.3(10)	1976.1(12)	5046.4(11)	27.1(3)
C2	6878.2(10)	2386.8(11)	5915.6(10)	23.0(3)
C3	6479.7(10)	1171.6(11)	5453.5(10)	25.0(3)
C4	7451.2(12)	374.3(12)	5167.2(11)	31.2(3)
C5	7722.3(14)	460.5(14)	3924.3(13)	39.5(3)
C6	8602.5(12)	1439.0(13)	3872.4(12)	32.2(3)
C7	8484.3(12)	868.6(13)	5760.5(12)	32.7(3)
C8	5923.0(12)	3223.7(13)	6078.1(12)	32.6(3)
C9	5192.9(12)	2647.6(14)	6915.4(12)	35.0(3)
C10	4804.7(12)	1404.7(15)	6541.4(13)	37.8(3)
C11	5752.4(12)	595.5(13)	6290.7(11)	31.0(3)
C12	7834.8(9)	5255.2(11)	5445.8(10)	22.3(2)
C13	8824.6(10)	5385.0(12)	6047.6(10)	25.7(3)
C14	8966.8(11)	6344.5(12)	6772.9(11)	28.3(3)
C15	8137.8(11)	7169.6(11)	6912.5(10)	27.6(3)
C16	7162.1(11)	7029.9(12)	6286.7(11)	29.2(3)
C17	7002.1(11)	6077.5(12)	5550.3(11)	27.4(3)
C18	8293.7(14)	8185.8(14)	7729.5(13)	39.7(3)

Table 30: Anisotropic Displacement Parameters (×10^4) for **ygq-706-2.** The anisotropic displacement factor exponent takes the form: $-2\pi^2[h^2a^{*2} \times U_{11} + ... +2hka^* \times b^* \times U_{12}]$

Atom	U_{11}	U_{22}	U_{33}	U_{23}	U_{13}	U_{12}
S1	27.59(17)	23.31(17)	19.34(16)	0.95(10)	2.99(11)	-1.13(11)
F1	24.4(4)	50.3(5)	44.0(5)	4.4(4)	-0.6(3)	-4.9(3)
O1	38.4(5)	27.6(5)	26.5(5)	0.8(3)	12.8(4)	-1.2(4)
O2	33.7(5)	37.1(5)	23.6(4)	3.2(4)	-4.7(4)	-2.0(4)
N1	26.5(5)	20.9(5)	25.3(5)	0.7(4)	7.0(4)	-0.6(4)
C1	23.1(6)	28.7(7)	29.6(6)	1.6(5)	1.1(5)	1.0(5)
C2	25.5(6)	24.0(6)	19.9(5)	0.9(4)	3.3(4)	-2.7(5)
C3	29.9(6)	25.2(6)	19.7(6)	-0.5(5)	0.3(5)	-4.3(5)
C4	42.9(8)	20.6(6)	30.6(7)	0.2(5)	6.8(6)	-0.3(5)
C5	54.1(9)	32.4(8)	33.5(8)	-11.5(6)	14.3(7)	-4.8(7)
C6	38.6(7)	27.8(7)	31.1(7)	-0.7(5)	10.1(5)	6.2(6)
C7	33.5(7)	31.8(7)	33.0(7)	6.8(5)	3.2(6)	8.1(6)
C8	34.6(7)	29.0(7)	35.5(7)	2.7(6)	12.6(6)	2.5(6)
C9	35.0(7)	35.7(8)	35.7(7)	-0.4(6)	14.7(6)	-1.4(6)
C10	35.1(7)	45.9(9)	33.3(7)	-3.0(6)	10.1(6)	-13.2(6)
C11	40.3(7)	29.2(7)	23.8(6)	-1.4(5)	3.7(5)	-12.4(6)

Atom	U_{11}	U_{22}	U_{33}	U_{23}	U_{13}	U_{12}
C12	25.6(6)	20.0(6)	21.8(5)	2.4(4)	5.5(4)	-0.6(4)
C13	24.8(6)	25.6(6)	27.1(6)	0.8(5)	4.7(5)	1.8(5)
C14	28.0(6)	29.3(7)	27.8(6)	-0.8(5)	2.4(5)	-2.5(5)
C15	37.2(7)	21.4(6)	25.2(6)	2.1(5)	9.0(5)	-1.9(5)
C16	32.0(7)	23.1(6)	33.3(7)	3.6(5)	9.2(5)	5.3(5)
C17	26.3(6)	25.1(6)	30.9(7)	3.6(5)	2.1(5)	2.4(5)
C18	52.9(9)	29.9(7)	36.7(8)	-7.3(6)	5.1(6)	1.8(6)

Table 31: Bond Lengths in Å for **ygq-706-2**.

Atom	Atom	Length/Å
S1	O1	1.4330(9)
S1	O2	1.4317(10)
S1	N1	1.6373(10)
S1	C12	1.7643(12)
F1	C1	1.3921(14)
N1	C1	1.4694(16)
N1	C2	1.4981(15)
C1	C6	1.5323(18)
C1	C7	1.5232(18)
C2	C3	1.5340(17)
C2	C8	1.5236(18)
C3	C4	1.5461(19)
C3	C11	1.5287(17)

Atom	Atom	Length/Å
C4	C5	1.5545(19)
C4	C7	1.525(2)
C5	C6	1.542(2)
C8	C9	1.5309(18)
C9	C10	1.525(2)
C10	C11	1.520(2)
C12	C13	1.3889(17)
C12	C17	1.3872(17)
C13	C14	1.3850(19)
C14	C15	1.3922(19)
C15	C16	1.3893(19)
C15	C18	1.5047(19)
C16	C17	1.3895(19)

Table 32: Bond Angles in ° for **ygq-706-2**.

Atom	Atom	Atom	Angle/°
O1	S1	N1	106.82(5)
O1	S1	C12	107.83(6)
O2	S1	O1	118.39(6)
O2	S1	N1	110.07(6)
O2	S1	C12	107.04(6)
N1	S1	C12	106.04(5)
C1	N1	S1	120.54(8)
C1	N1	C2	115.48(10)
C2	N1	S1	123.02(8)
F1	C1	N1	108.86(10)
F1	C1	C6	112.51(10)
F1	C1	C7	111.00(11)
N1	C1	C6	113.31(10)
N1	C1	C7	109.15(10)
C7	C1	C6	101.84(11)
N1	C2	C3	108.32(10)
N1	C2	C8	117.45(10)
C8	C2	C3	110.73(11)
C2	C3	C4	110.66(10)
C11	C3	C2	108.65(10)
C11	C3	C4	113.39(11)

Atom	Atom	Atom	Angle/°
C3	C4	C5	113.08(12)
C7	C4	C3	108.90(11)
C7	C4	C5	101.95(12)
C6	C5	C4	105.92(12)
C1	C6	C5	102.91(11)
C1	C7	C4	99.19(11)
C2	C8	C9	108.43(11)
C10	C9	C8	111.91(12)
C11	C10	C9	111.50(12)
C10	C11	C3	111.63(12)
C13	C12	S1	119.55(9)
C17	C12	S1	119.34(10)
C17	C12	C13	121.03(12)
C14	C13	C12	118.92(12)
C13	C14	C15	121.32(12)
C14	C15	C18	120.66(13)
C16	C15	C14	118.57(12)
C16	C15	C18	120.77(12)
C15	C16	C17	121.13(12)
C12	C17	C16	119.00(12)

Table 33: Torsion Angles in ° for **ygq-706-2**.

Atom	Atom	Atom	Atom	Angle/°
S1	N1	C1	F1	63.41(12)
S1	N1	C1	C6	-62.58(13)
S1	N1	C1	C7	-175.29(9)
S1	N1	C2	C3	117.29(10)
S1	N1	C2	C8	-9.02(16)
S1	C12	C13	C14	177.82(10)
S1	C12	C17	C16	-178.10(10)
F1	C1	C6	C5	156.53(12)
F1	C1	C7	C4	-172.65(10)
O1	S1	N1	C1	-1.74(11)
O1	S1	N1	C2	-170.00(9)
O1	S1	C12	C13	-50.17(11)
O1	S1	C12	C17	126.75(10)
O2	S1	N1	C1	128.00(10)
O2	S1	N1	C2	-40.26(11)
O2	S1	C12	C13	-178.56(9)
O2	S1	C12	C17	-1.64(12)
N1	S1	C12	C13	63.95(11)
N1	S1	C12	C17	-119.12(10)
N1	C1	C6	C5	-79.45(14)
N1	C1	C7	C4	67.37(13)
N1	C2	C3	C4	43.64(13)
N1	C2	C3	C11	168.75(10)
N1	C2	C8	C9	-174.00(11)
C1	N1	C2	C3	-51.51(13)
C1	N1	C2	C8	-177.82(11)
C2	N1	C1	F1	-127.49(10)
C2	N1	C1	C6	106.52(12)
C2	N1	C1	C7	-6.19(15)
C2	C3	C4	C5	-94.89(13)
C2	C3	C4	C7	17.68(14)
C2	C3	C11	C10	56.93(14)
C2	C8	C9	C10	-56.88(17)
C3	C2	C8	C9	60.88(14)
C3	C4	C5	C6	93.28(15)
C3	C4	C7	C1	-73.48(12)
C4	C3	C11	C10	-179.58(11)
C4	C5	C6	C1	-8.53(15)
C5	C4	C7	C1	46.26(13)
C6	C1	C7	C4	-52.68(12)
C7	C1	C6	C5	37.63(14)
C7	C4	C5	C6	-23.47(15)
C8	C2	C3	C4	173.76(10)
C8	C2	C3	C11	-61.13(13)
C8	C9	C10	C11	54.06(17)
C9	C10	C11	C3	-54.01(16)
C11	C3	C4	C5	142.73(12)
C11	C3	C4	C7	-104.70(12)
C12	S1	N1	C1	-116.56(10)
C12	S1	N1	C2	75.18(10)
C12	C13	C14	C15	0.42(19)
C13	C12	C17	C16	-1.22(19)
C13	C14	C15	C16	-1.47(19)
C13	C14	C15	C18	178.13(13)
C14	C15	C16	C17	1.19(19)
C15	C16	C17	C12	0.13(19)
C17	C12	C13	C14	0.95(18)
C18	C15	C16	C17	-178.42(13)

Table 34: Hydrogen Fractional Atomic Coordinates (×10⁴) and Equivalent Isotropic Displacement Parameters (Å²×10³) for **ygq-706-2**. U_{eq} is defined as 1/3 of the trace of the orthogonalised U_{ij}.

Atom	x	y	z	U_{eq}
H2	7206(12)	2254(14)	6656(13)	27(4)
H3	6059(12)	1331(14)	4779(13)	27(4)
H4	7315(13)	-434(17)	5364(14)	37(4)
H5A	7049(17)	665(18)	3433(17)	56(6)
H5B	8035(15)	-294(19)	3685(16)	54(5)
H6A	8430(13)	2028(15)	3285(14)	33(4)
H6B	9332(13)	1085(15)	3793(14)	35(4)
H7A	8434(14)	1029(15)	6548(16)	40(5)
H7B	9112(14)	321(16)	5655(14)	41(4)
H8A	6199(14)	4019(16)	6362(15)	41(5)
H8B	5496(14)	3369(16)	5346(15)	41(5)
H9A	4565(14)	3175(16)	7017(15)	43(5)
H9B	5610(12)	2584(15)	7656(14)	33(4)
H10A	4311(15)	1494(18)	5854(17)	51(5)
H10B	4378(15)	1027(16)	7113(16)	44(5)
H11A	6200(12)	410(14)	6988(13)	30(4)
H11B	5489(13)	-192(16)	5981(14)	39(4)
H13	9405(13)	4837(15)	5959(13)	35(4)
H14	9652(14)	6441(16)	7202(14)	39(4)
H16	6578(13)	7614(15)	6345(13)	32(4)
H17	6330(13)	5983(14)	5113(14)	32(4)
H18A	8350.72	7864.56	8488.9	48
H18B	7671.18	8732.24	7647.82	48
H18C	8960.83	8622.42	7584.01	48
H18D	8304.43	8948.25	7324.92	48
H18E	8983.97	8080.57	8166	48
H18F	7694.32	8190.39	8229.81	48

Table 35: Atomic Occupancies for all atoms that are not fully occupied in **ygq-706-2**.

Atom	Occupancy	Atom	Occupancy	Atom	Occupancy
H18A	0.689(19)	H18C	0.689(19)	H18E	0.311(19)
H18B	0.689(19)	H18D	0.311(19)	H18F	0.311(19)

Citations

CrysAlisPro (ROD), Rigaku Oxford Diffraction, Poland (?).

CrysAlisPro Software System, Rigaku Oxford Diffraction, (2024).

O.V. Dolomanov and L.J. Bourhis and R.J. Gildea and J.A.K. Howard and H. Puschmann, Olex2: A complete structure solution, refinement and analysis program, *J. Appl. Cryst.*, (2009), **42**, 339-341.

Sheldrick, G.M., Crystal structure refinement with ShelXL, *Acta Cryst.*, (2015), **C71**, 3-8.

Sheldrick, G.M., ShelXT-Integrated space-group and crystal-structure determination, *Acta Cryst.*, (2015), **A71**, 3-8.

Crystal Data and Experimental of Pd complex (12) (CCDC: 2417054)

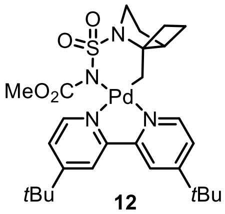

12

***R₁*=3.41%**

Crystal Data and Experimental

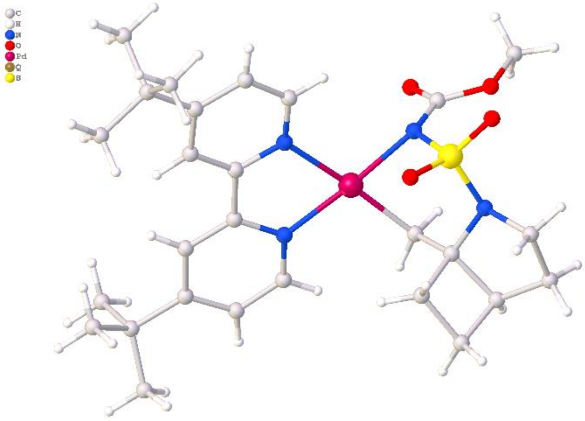

Compound	ybc-XIV-047-1-p2
Formula	$C_{33}H_{52}N_4O_4PdS$
$D_{calc.}$/ g cm^{-3}	1.381
μ/mm^{-1}	5.302
Formula Weight	707.24
Colour	clear pale colourless
Shape	needle
Size/mm^3	0.17×0.06×0.05
T/K	199.99(10)
Crystal System	triclinic
Space Group	P-1
a/Å	10.8334(4)
b/Å	12.8063(4)
c/Å	13.5342(5)
α/°	112.783(3)
β/°	95.164(3)
γ/°	96.725(3)
V/Å^3	1700.38(11)
Z	2
Z'	1
Wavelength/Å	1.54184
Radiation type	Cu K_α
Θ_{min}/°	3.581
Θ_{max}/°	72.591
Measured Refl's.	15820
Indep't Refl's	6531
Refl's I≥2 σ(I)	5343
R_{int}	0.0389
Parameters	341
Restraints	0
Largest Peak	0.527
Deepest Hole	-0.795
GooF	0.993
wR_2 (all data)	0.0849
wR_2	0.0799
R_1 (all data)	0.0467
R_1	0.0341

Experimental. Single clear pale colourless needle-shaped crystals of **ybc-XIV-047-1-p2** were used as supplied. A suitable crystal with dimensions 0.17 × 0.06 × 0.05 mm^3 was selected and mounted on a SuperNova, Dual, Cu at home/near, Atlas diffractometer. The crystal was kept at a steady T = 199.99(10) K during data collection. The structure was solved with the ShelXT 2018/2 (Sheldrick, 2018) solution program using dual methods and by using Olex2 1.5 (Dolomanov et al., 2009) as the graphical interface. The model was refined with ShelXL 2019/3 (Sheldrick, 2015) using full matrix least squares minimisation on F^2.

Crystal Data. $C_{33}H_{52}N_4O_4PdS$, M_r = 707.24, triclinic, P-1 (No. 2), a = 10.8334(4) Å, b = 12.8063(4) Å, c = 13.5342(5) Å, α = 112.783(3)°, β = 95.164(3)°, γ = 96.725(3)°, V = 1700.38(11) Å^3, T = 199.99(10) K, Z = 2, Z' = 1, μ(Cu K_α) = 5.302, 15820 reflections measured, 6531 unique (R_{int} = 0.0389) which were used in all calculations. The final wR_2 was 0.0849 (all data) and R_1 was 0.0341 (I≥2 σ(I)).

Structure Quality Indicators

**Reflections:**	d min (CuKα) 2Θ=145.2° 0.81	I/σ(I) 20.6	Rint m=2.42 3.89%	Full 135.4° 97% to 145.2° 99.7
**Refinement:**	Shift -0.001	Max Peak 0.5	Min Peak -0.8	GooF 0.993

A clear pale colourless needle-shaped crystal with dimensions 0.17 × 0.06 × 0.05 mm^3 was mounted. Data were collected using a SuperNova, Dual, Cu at home/near, Atlas diffractometer operating at T = 199.99(10) K.

Data were measured using ω scans with Cu K_α radiation. The diffraction pattern was indexed and the total number of runs and images was based on the strategy calculation from the program CrysAlisPro system (CCD 43.128a 64-bit (release 20-06-2024)). The maximum resolution that was achieved was Θ = 72.591° (0.81 Å).

The unit cell was refined using CrysAlisPro 1.171.43.129a (Rigaku OD, 2024) on 6839 reflections, 43% of the observed reflections.

Data reduction, scaling and absorption corrections were performed using CrysAlisPro 1.171.43.129a (Rigaku OD, 2024). The final completeness is 99.70 % out to 72.591° in Θ. A gaussian absorption correction was performed using CrysAlisPro 1.171.43.129a (Rigaku Oxford Diffraction, 2024). The numerical absorption correction was based on gaussian integration over a multifaceted crystal model. The empirical absorption correction was done using spherical harmonics, implemented in SCALE3 ABSPACK scaling algorithm. The absorption coefficient μ of this crystal is 5.302 mm^{-1} at this wavelength (λ = 1.54184Å) and the minimum and maximum transmissions are 0.722 and 1.000.

The structure was solved and the space group *P*-1 (# 2) determined by the ShelXT 2018/2 (Sheldrick, 2018) structure solution program using dual methods and refined by full matrix least squares minimisation on $\boldsymbol{F}^2$ using version 2019/3 of ShelXL (Sheldrick, 2015). All non-hydrogen atoms were refined anisotropically. Hydrogen atom positions were calculated geometrically and refined using the riding model.

_smtbx_masks_special_details: A solvent mask was calculated and 101 electrons were found in a volume of 486$Å^3$ in 1 void per unit cell. This is consistent with the presence of 1[C6H14] per formula unit which account for 100 electrons per unit cell.

There is a single formula unit in the asymmetric unit, which is represented by the reported sum formula. In other words: Z is 2 and Z' is 1. The moiety formula is C27 H38 N4 O4 Pd S, 1[C6H14].

Data Plots: Diffraction Data

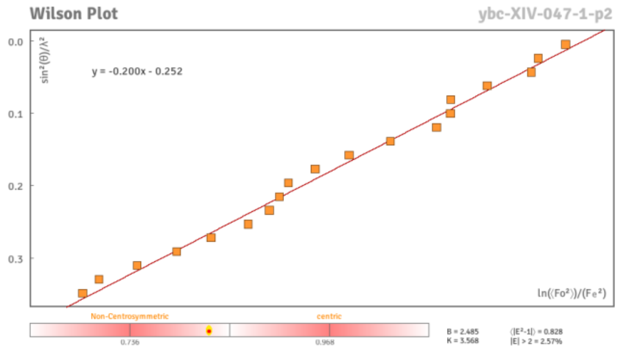

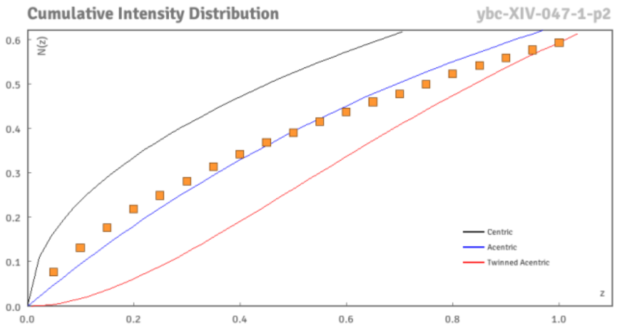

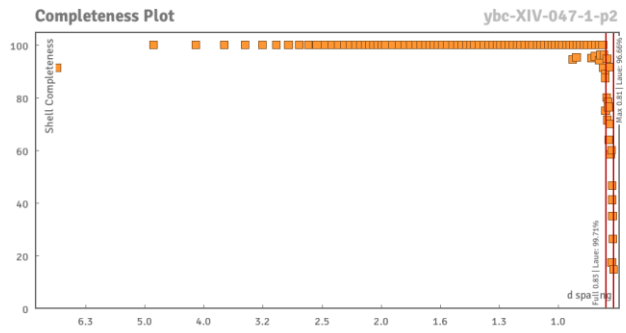

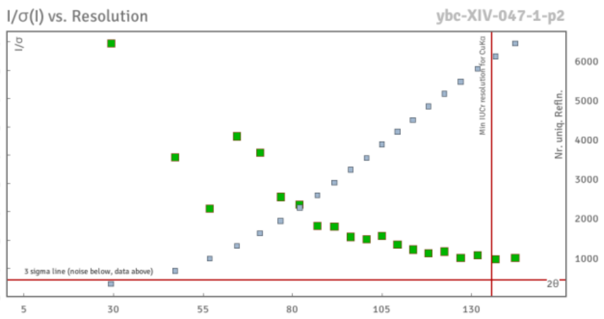

Data Plots: Refinement and Data

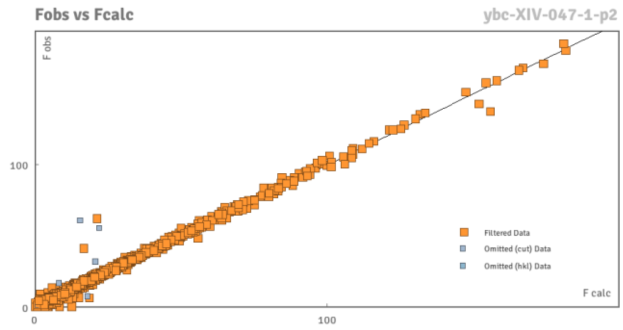

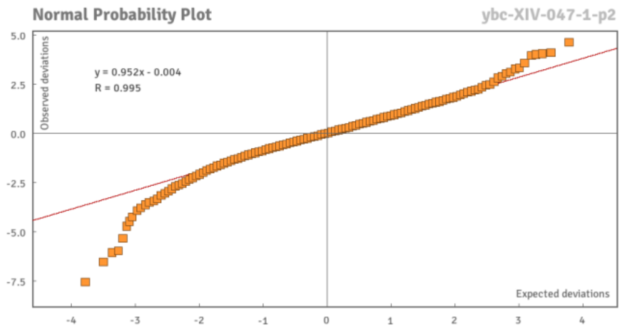

Reflection Statistics

Total reflections (after filtering)	15820	Unique reflections	6531
Completeness	0.967	Mean I/σ	14.73
hkl_{max} collected	(12, 15, 16)	hkl_{min} collected	(-13, -15, -12)
hkl_{max} used	(13, 14, 16)	hkl_{min} used	(-13, -15, 0)
Lim d_{max} collected	100.0	Lim d_{min} collected	0.77
d_{max} used	12.34	d_{min} used	0.81
Friedel pairs	1792	Friedel pairs merged	1
Inconsistent equivalents	8	R_{int}	0.0389
R_{sigma}	0.0485	Intensity transformed	0
Omitted reflections	8	Omitted by user (OMIT hkl)	0
Multiplicity	(3739, 2847, 1050, 389, 171, 99, 32, 2)	Maximum multiplicity	8
Removed systematic absences	0	Filtered off (Shel/OMIT)	0

Table 36: Fractional Atomic Coordinates (×10^4) and Equivalent Isotropic Displacement Parameters (Å^2×10^3) for **ybc-XIV-047-1-p2.** U_{eq} is defined as 1/3 of the trace of the orthogonalised U_{ij}.

Atom	x	y	z	U_{eq}
Pd1	6670.4(2)	7248.5(2)	6352.2(2)	33.83(8)
S1	9475.4(7)	8165.8(6)	6719.3(7)	41.18(18)
O1	7156(2)	7561(2)	4246(2)	50.6(6)
O2	9072(2)	8608.0(19)	4813.0(19)	46.0(5)
O3	9408(2)	7494(2)	7354(2)	54.0(6)
O4	10651(2)	8331(2)	6351(2)	52.9(6)
N1	8286(2)	7591(2)	5758(2)	37.5(6)
N2	9146(2)	9456(2)	7391(2)	40.2(6)
N3	5065(2)	6790.9(19)	6886(2)	35.2(6)
N4	6651(2)	5462.0(18)	5862(2)	31.6(5)
C1	6793(3)	9003(2)	7027(3)	40.5(7)

Atom	x	y	z	U_{eq}
C2	7958(3)	9543(3)	7868(3)	42.9(7)
C3	7925(4)	9248(3)	8888(3)	54.8(9)
C4	7872(5)	10525(3)	9591(3)	65.6(11)
C5	8115(4)	10849(3)	8630(3)	51.5(9)
C6	9471(4)	11349(3)	8661(3)	59.0(10)
C7	10145(3)	10312(3)	8214(3)	51.7(9)
C8	8115(3)	7912(2)	4896(3)	36.7(7)
C9	8903(3)	8913(3)	3904(3)	50.2(8)
C10	4216(3)	7447(3)	7333(3)	46.9(8)
C11	3152(3)	7060(3)	7655(3)	47.9(8)
C12	2900(3)	5925(2)	7529(3)	36.1(6)
C13	3751(3)	5226(2)	7021(2)	34.9(6)
C14	4805(3)	5664(2)	6717(2)	31.6(6)
C15	5742(3)	4950(2)	6221(2)	30.4(6)
C16	5725(3)	3841(2)	6151(3)	36.4(7)
C17	6681(3)	3229(2)	5734(3)	37.9(7)
C18	7576(3)	3762(2)	5334(3)	37.9(7)
C19	7540(3)	4871(2)	5421(3)	35.7(6)
C20	1740(3)	5430(3)	7875(3)	44.7(8)
C21	1315(4)	6379(3)	8825(3)	59.9(10)
C22	2033(5)	4481(4)	8236(4)	71.2(13)
C23	691(4)	4949(4)	6914(3)	61.7(10)
C24	6737(3)	2032(3)	5706(3)	46.7(8)
C25	5797(4)	1713(3)	6358(4)	73.6(14)
C26	6445(4)	1158(3)	4530(4)	69.5(12)
C27	8060(3)	1987(3)	6165(4)	58.6(10)

Table 37: Anisotropic Displacement Parameters (×10[4]) for **ybc-XIV-047-1-p2.** The anisotropic displacement factor exponent takes the form: $-2\pi^2[h^2a^{*2} \times U_{11} + \ldots + 2hka^* \times b^* \times U_{12}]$

Atom	U_{11}	U_{22}	U_{33}	U_{23}	U_{13}	U_{12}
Pd1	31.40(12)	21.58(10)	51.71(15)	16.70(9)	11.20(9)	5.69(7)
S1	34.2(4)	34.9(4)	60.1(5)	23.0(4)	9.8(3)	11.2(3)
O1	39.6(13)	49.0(13)	58.8(15)	21.9(12)	2.8(11)	-6.9(10)
O2	36.8(12)	40.4(11)	63.3(15)	28.4(11)	3.2(11)	-7.0(9)
O3	53.3(15)	47.7(13)	74.4(17)	35.7(13)	10.3(13)	19.7(11)
O4	35.2(12)	52.2(14)	73.3(17)	25.7(13)	11.4(11)	11.3(10)
N1	33.8(13)	26.2(11)	53.7(16)	16.5(11)	10.9(12)	4.8(10)
N2	36.1(14)	30.5(12)	54.3(16)	18.1(12)	4.3(12)	4.8(10)
N3	33.1(13)	25.1(11)	51.4(15)	17.1(11)	12.6(11)	8.8(10)
N4	30.5(12)	19.5(10)	43.7(14)	11.3(10)	7.0(10)	4.4(9)
C1	38.1(17)	23.5(13)	59(2)	14.4(14)	11.7(15)	5.8(12)
C2	45.2(18)	27.2(14)	58(2)	15.3(14)	15.1(15)	11.0(13)
C3	60(2)	42.9(18)	64(2)	23.8(17)	11.5(19)	10.0(16)
C4	91(3)	44(2)	60(2)	16.8(18)	22(2)	14(2)
C5	55(2)	30.8(15)	65(2)	15.1(16)	8.3(18)	6.4(14)
C6	57(2)	40.9(18)	70(3)	15.9(18)	4.5(19)	1.4(16)
C7	45(2)	48.0(19)	58(2)	20.3(17)	-1.1(16)	1.0(15)
C8	32.1(16)	22.7(12)	53.7(19)	11.9(13)	13.9(14)	5.1(11)
C9	46(2)	43.6(18)	66(2)	29.8(17)	9.6(17)	1.4(15)
C10	48.7(19)	26.7(14)	74(2)	22.9(15)	28.1(17)	16.7(13)
C11	45.5(19)	35.6(16)	71(2)	22.8(16)	28.3(17)	19.0(14)
C12	34.8(16)	30.4(14)	43.9(17)	14.9(13)	8.9(13)	6.4(12)
C13	29.9(15)	25.7(13)	48.2(18)	13.4(13)	6.5(13)	5.1(11)
C14	29.9(14)	20.7(12)	39.4(16)	8.4(11)	-0.4(12)	3.0(10)
C15	24.6(13)	24.8(12)	39.9(16)	12.2(12)	1.3(12)	2.1(10)

Atom	U_{11}	U_{22}	U_{33}	U_{23}	U_{13}	U_{12}
C16	25.8(14)	25.1(13)	59(2)	17.9(13)	7.0(13)	2.5(11)
C17	28.1(14)	23.8(13)	60(2)	16.7(13)	2.4(13)	2.8(11)
C18	29.0(15)	28.4(14)	54.5(19)	13.0(13)	10.8(13)	7.4(11)
C19	30.4(15)	28.0(13)	49.1(18)	15.1(13)	9.1(13)	5.1(11)
C20	39.1(17)	35.5(15)	61(2)	17.5(15)	22.3(16)	9.6(13)
C21	57(2)	51(2)	73(3)	21.4(19)	30(2)	10.8(17)
C22	78(3)	60(2)	106(3)	52(2)	52(3)	26(2)
C23	44(2)	55(2)	75(3)	18(2)	14.5(19)	-4.9(17)
C24	32.2(16)	26.4(14)	87(3)	26.3(16)	12.4(16)	9.2(12)
C25	54(2)	48(2)	150(4)	65(3)	41(3)	20.7(18)
C26	60(2)	27.5(16)	109(4)	16(2)	4(2)	7.4(16)
C27	42(2)	46.8(19)	102(3)	44(2)	9(2)	13.6(16)

Table 38: Bond Lengths in Å for **ybc-XIV-047-1-p2**.

Atom	Atom	Length/Å
Pd1	N1	2.062(3)
Pd1	N3	2.050(2)
Pd1	N4	2.118(2)
Pd1	C1	2.054(3)
S1	O3	1.433(3)
S1	O4	1.431(3)
S1	N1	1.622(3)
S1	N2	1.648(3)
O1	C8	1.215(4)
O2	C8	1.328(4)
O2	C9	1.431(4)
N1	C8	1.383(4)
N2	C2	1.487(4)
N2	C7	1.475(4)
N3	C10	1.344(4)
N3	C14	1.361(3)
N4	C15	1.348(4)
N4	C19	1.334(4)
C1	C2	1.515(5)
C2	C3	1.569(5)
C2	C5	1.567(4)
C3	C4	1.551(5)
C4	C5	1.545(6)
C5	C6	1.521(5)
C6	C7	1.532(5)
C10	C11	1.375(5)
C11	C12	1.387(4)
C12	C13	1.398(4)
C12	C20	1.532(4)
C13	C14	1.379(4)
C14	C15	1.477(4)
C15	C16	1.385(4)
C16	C17	1.399(4)
C17	C18	1.387(4)
C17	C24	1.528(4)
C18	C19	1.385(4)
C20	C21	1.540(5)
C20	C22	1.530(5)
C20	C23	1.521(5)
C24	C25	1.525(5)
C24	C26	1.528(6)
C24	C27	1.526(5)

Table 39: Bond Angles in ° for **ybc-XIV-047-1-p2**.

Atom	Atom	Atom	Angle/°
N1	Pd1	N4	97.03(9)
N3	Pd1	N1	176.10(9)
N3	Pd1	N4	79.16(9)
N3	Pd1	C1	99.69(11)
C1	Pd1	N1	84.20(11)
C1	Pd1	N4	172.41(12)
O3	S1	N1	105.61(15)
O3	S1	N2	111.08(15)
O4	S1	O3	116.41(15)
O4	S1	N1	114.16(15)
O4	S1	N2	106.52(14)
N1	S1	N2	102.20(13)
C8	O2	C9	115.7(3)
S1	N1	Pd1	111.22(15)
C8	N1	Pd1	115.3(2)
C8	N1	S1	122.9(2)
C2	N2	S1	118.2(2)
C7	N2	S1	117.6(2)
C7	N2	C2	107.4(3)
C10	N3	Pd1	128.3(2)
C10	N3	C14	116.3(3)
C14	N3	Pd1	115.29(19)
C15	N4	Pd1	113.43(17)
C19	N4	Pd1	126.91(19)
C19	N4	C15	118.6(2)
C2	C1	Pd1	110.0(2)

Atom	Atom	Atom	Angle/°
N2	C2	C1	113.2(3)
N2	C2	C3	116.4(3)
N2	C2	C5	103.1(3)
C1	C2	C3	114.0(3)
C1	C2	C5	118.6(3)
C5	C2	C3	89.1(3)
C4	C3	C2	89.5(3)
C5	C4	C3	90.5(3)
C4	C5	C2	89.8(3)
C6	C5	C2	105.7(3)
C6	C5	C4	115.0(4)
C5	C6	C7	105.4(3)
N2	C7	C6	101.5(3)
O1	C8	O2	122.2(3)
O1	C8	N1	121.8(3)
O2	C8	N1	115.9(3)
N3	C10	C11	124.3(3)
C10	C11	C12	120.0(3)
C11	C12	C13	115.9(3)
C11	C12	C20	123.2(3)
C13	C12	C20	120.9(3)
C14	C13	C12	121.4(3)
N3	C14	C13	122.0(3)

Atom	Atom	Atom	Angle/°
N3	C14	C15	115.8(3)
C13	C14	C15	122.2(2)
N4	C15	C14	115.5(2)
N4	C15	C16	121.4(3)
C16	C15	C14	123.1(3)
C15	C16	C17	120.6(3)
C16	C17	C24	122.1(3)
C18	C17	C16	116.5(3)
C18	C17	C24	121.3(3)
C19	C18	C17	120.1(3)
N4	C19	C18	122.6(3)
C12	C20	C21	110.2(3)
C22	C20	C12	110.7(3)
C22	C20	C21	108.4(3)
C23	C20	C12	108.2(3)
C23	C20	C21	109.3(3)
C23	C20	C22	110.0(3)
C25	C24	C17	111.7(3)
C25	C24	C26	109.2(3)
C25	C24	C27	109.3(3)
C26	C24	C17	108.8(3)
C27	C24	C17	109.5(3)
C27	C24	C26	108.4(3)

Table 40: Torsion Angles in ° for **ybc-XIV-047-1-p2**.

Atom	Atom	Atom	Atom	Angle/°
Pd1	N1	C8	O1	-30.7(4)
Pd1	N1	C8	O2	150.5(2)
Pd1	N3	C10	C11	179.1(3)
Pd1	N3	C14	C13	-179.3(2)
Pd1	N3	C14	C15	2.6(3)
Pd1	N4	C15	C14	10.0(3)
Pd1	N4	C15	C16	-168.1(2)
Pd1	N4	C19	C18	166.1(2)
Pd1	C1	C2	N2	69.7(3)
Pd1	C1	C2	C3	-66.4(3)
Pd1	C1	C2	C5	-169.3(2)
S1	N1	C8	O1	-172.1(2)
S1	N1	C8	O2	9.1(4)
S1	N2	C2	C1	-62.4(3)
S1	N2	C2	C3	72.7(3)
S1	N2	C2	C5	168.2(2)
S1	N2	C7	C6	-178.4(2)
O3	S1	N1	Pd1	45.50(17)
O3	S1	N1	C8	-171.7(2)
O3	S1	N2	C2	-52.5(3)
O3	S1	N2	C7	79.0(3)
O4	S1	N1	Pd1	174.66(13)
O4	S1	N1	C8	-42.6(3)
O4	S1	N2	C2	179.8(2)
O4	S1	N2	C7	-48.7(3)
N1	S1	N2	C2	59.7(3)
N1	S1	N2	C7	-168.8(3)
N2	S1	N1	Pd1	-70.77(15)
N2	S1	N1	C8	72.0(3)

Atom	Atom	Atom	Atom	Angle/°
N2	C2	C3	C4	112.5(3)
N2	C2	C5	C4	-125.2(3)
N2	C2	C5	C6	-9.2(4)
N3	C10	C11	C12	0.1(6)
N3	C14	C15	N4	-8.6(4)
N3	C14	C15	C16	169.5(3)
N4	C15	C16	C17	2.0(5)
C1	C2	C3	C4	-112.9(3)
C1	C2	C5	C4	108.8(4)
C1	C2	C5	C6	-135.3(3)
C2	N2	C7	C6	-42.2(4)
C2	C3	C4	C5	-8.4(3)
C2	C5	C6	C7	-15.7(4)
C3	C2	C5	C4	-8.3(3)
C3	C2	C5	C6	107.7(3)
C3	C4	C5	C2	8.4(3)
C3	C4	C5	C6	-98.9(3)
C4	C5	C6	C7	81.6(4)
C5	C2	C3	C4	8.2(3)
C5	C6	C7	N2	34.7(4)
C7	N2	C2	C1	161.7(3)
C7	N2	C2	C3	-63.2(3)
C7	N2	C2	C5	32.3(3)
C9	O2	C8	O1	-0.3(4)
C9	O2	C8	N1	178.4(3)
C10	N3	C14	C13	-2.2(5)
C10	N3	C14	C15	179.7(3)
C10	C11	C12	C13	-2.8(5)
C10	C11	C12	C20	179.6(3)
C11	C12	C13	C14	3.0(5)
C11	C12	C20	C21	-28.3(5)
C11	C12	C20	C22	-148.3(4)
C11	C12	C20	C23	91.1(4)
C12	C13	C14	N3	-0.6(5)
C12	C13	C14	C15	177.4(3)
C13	C12	C20	C21	154.1(3)
C13	C12	C20	C22	34.2(5)
C13	C12	C20	C23	-86.4(4)
C13	C14	C15	N4	173.3(3)
C13	C14	C15	C16	-8.6(5)
C14	N3	C10	C11	2.4(5)
C14	C15	C16	C17	-176.0(3)
C15	N4	C19	C18	-1.1(5)
C15	C16	C17	C18	-4.2(5)
C15	C16	C17	C24	176.3(3)
C16	C17	C18	C19	3.9(5)
C16	C17	C24	C25	-10.3(5)
C16	C17	C24	C26	110.2(4)
C16	C17	C24	C27	-131.4(3)
C17	C18	C19	N4	-1.4(5)
C18	C17	C24	C25	170.2(3)
C18	C17	C24	C26	-69.2(4)
C18	C17	C24	C27	49.1(5)
C19	N4	C15	C14	178.9(3)
C19	N4	C15	C16	0.8(4)
C20	C12	C13	C14	-179.3(3)
C24	C17	C18	C19	-176.6(3)

Table 41: Hydrogen Fractional Atomic Coordinates (×10[4]) and Equivalent Isotropic Displacement Parameters (Å[2]×10[3]) for **ybc-XIV-047-1-p2**. U_{eq} is defined as 1/3 of the trace of the orthogonalised U_{ij}.

Atom	x	y	z	U_{eq}
H1A	6830.02	9301.15	6453.81	49
H1B	6037.34	9209.28	7370.38	49
H3A	7164.08	8712.31	8843.03	66
H3B	8695.96	8986.41	9090.02	66
H4A	7040.96	10655.02	9819.87	79
H4B	8550.17	10883.27	10219.17	79
H5	7475.69	11264.64	8431.13	62
H6A	9855.86	11857.53	9412.82	71
H6B	9513.85	11796.18	8207.19	71
H7A	10878.96	10486.57	7884.58	62
H7B	10423.17	10052.01	8785.14	62
H9A	8820	8223.29	3232.13	75
H9B	9632.11	9464.75	3940.77	75
H9C	8142	9260.37	3917.7	75
H10	4360.81	8230.21	7432.03	56
H11	2588.42	7570.74	7965.13	57
H13	3599.92	4432.96	6882.58	42
H16	5058.86	3492.12	6389.66	44
H18	8215.31	3364.79	5000.45	45
H19	8173.46	5225.25	5156.26	43
H21A	2019.42	6749.4	9417.31	90
H21B	620.72	6040.49	9081.57	90
H21C	1032.73	6952.07	8582.54	90
H22A	2277.23	3857.08	7631.51	107
H22B	1285.08	4184.37	8465.01	107
H22C	2723.46	4793.31	8843.78	107
H23A	521.09	5557.87	6677.35	93
H23B	-71.05	4655.09	7127.36	93
H23C	945.2	4323.53	6317.13	93
H25A	5948.21	2297.63	7103.35	110
H25B	5898.11	965.32	6364.59	110
H25C	4941.01	1672.8	6026.49	110
H26A	5595.06	1171.79	4223.42	104
H26B	6497.44	388.06	4501.67	104
H26C	7053.93	1352.26	4109.36	104
H27A	8659.38	2133.72	5713.85	88
H27B	8089.19	1226.55	6167.58	88
H27C	8281.22	2573.33	6906.9	88

Table 42: Solvent masking (PLATON/SQUEEZE) information for **ybc-XIV-047-1-p2**.

No	x	y	z	V	e	Content
1	0.534	-0.394	0.000	486.2	101.3	2hexane

VIII. Copies of the NMR spectra

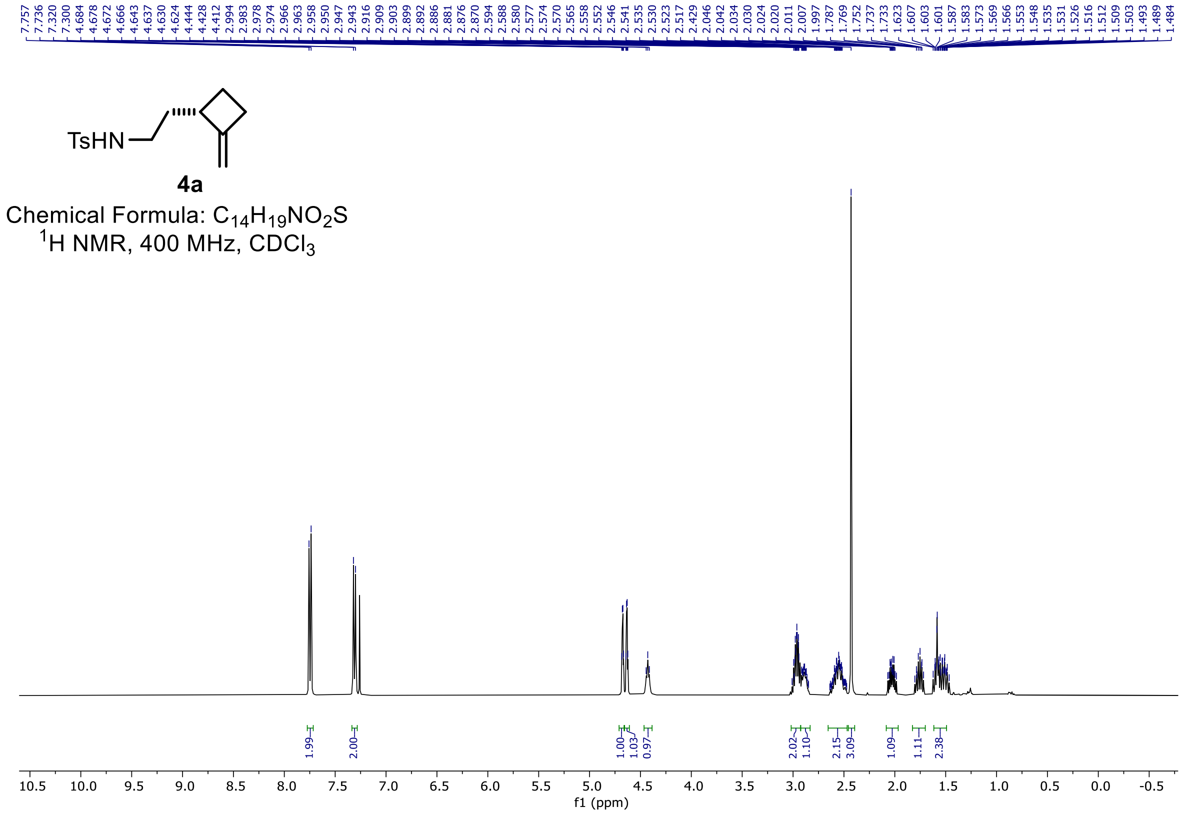

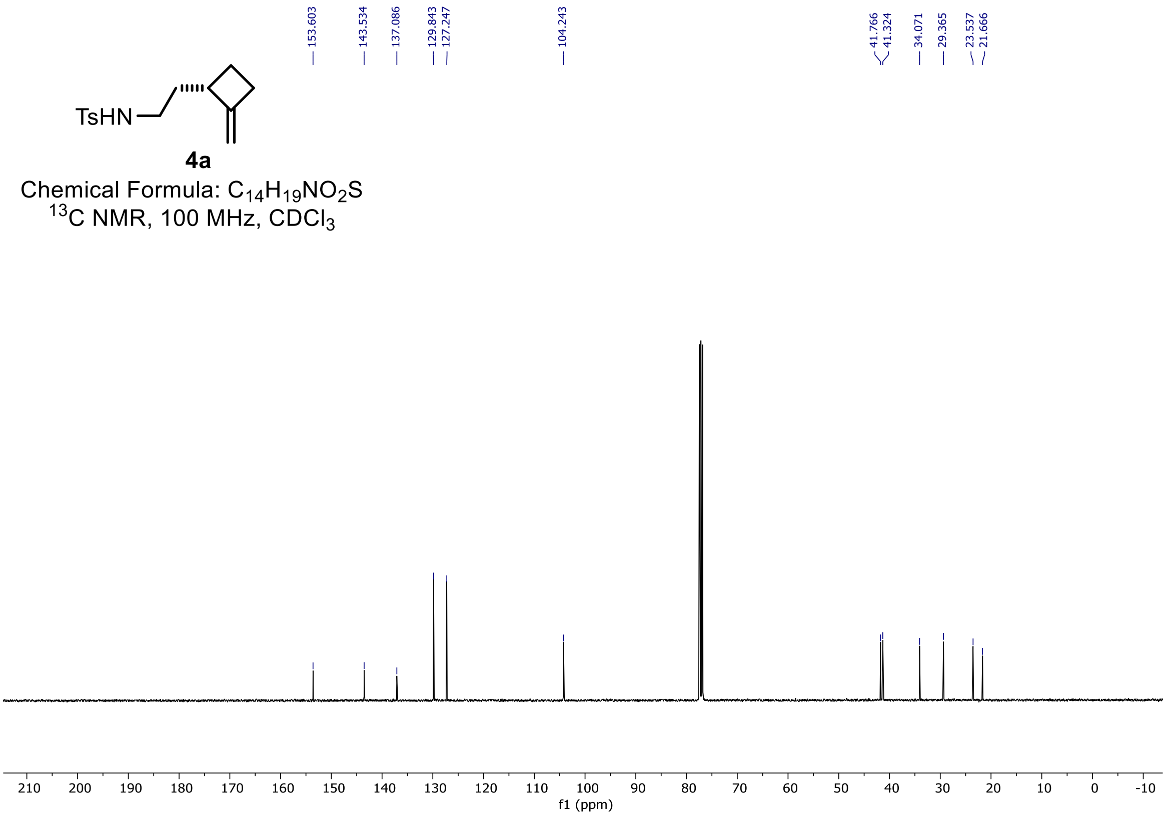

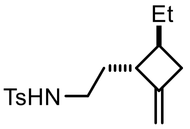

4b

Chemical Formula: $C_{16}H_{23}NO_2S$
^{1}H NMR, 400 MHz, $CDCl_3$

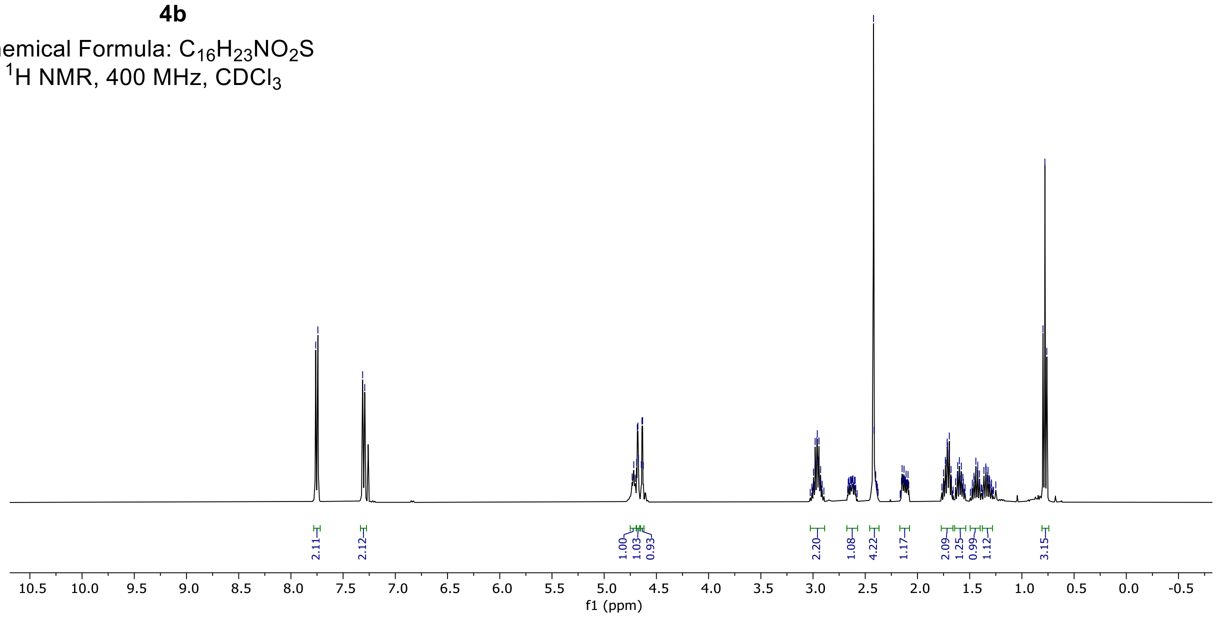

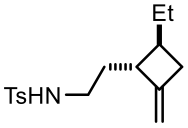

4b

Chemical Formula: $C_{16}H_{23}NO_2S$
^{13}C NMR, 100 MHz, $CDCl_3$

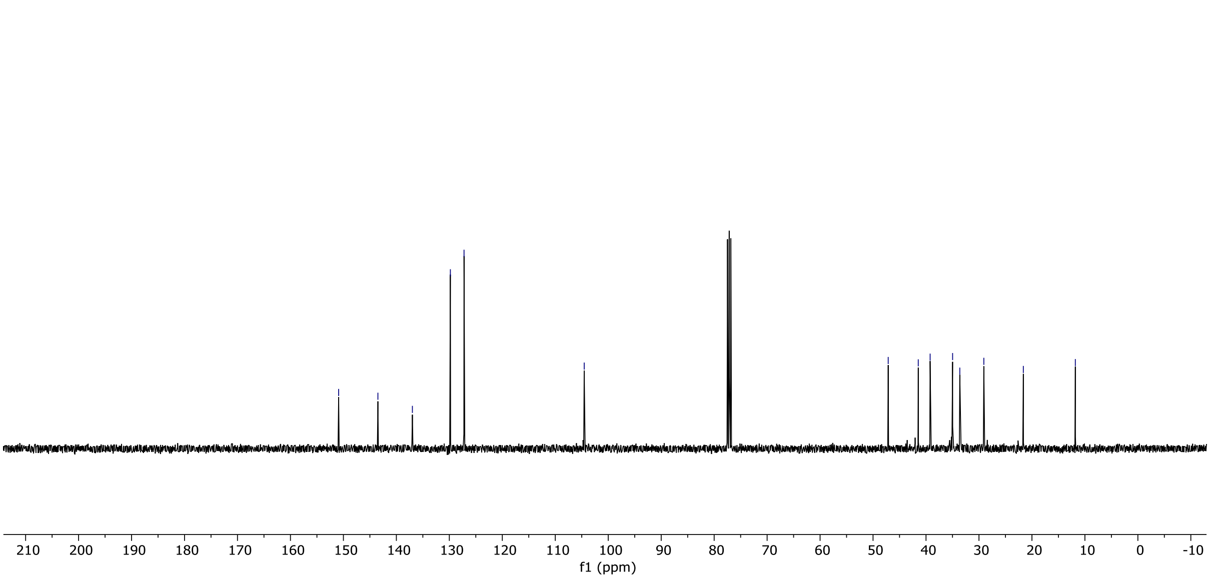

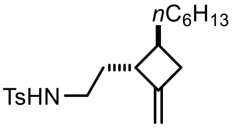

4c

Chemical Formula: $C_{20}H_{31}NO_2S$
^1H NMR, 400 MHz, $CDCl_3$

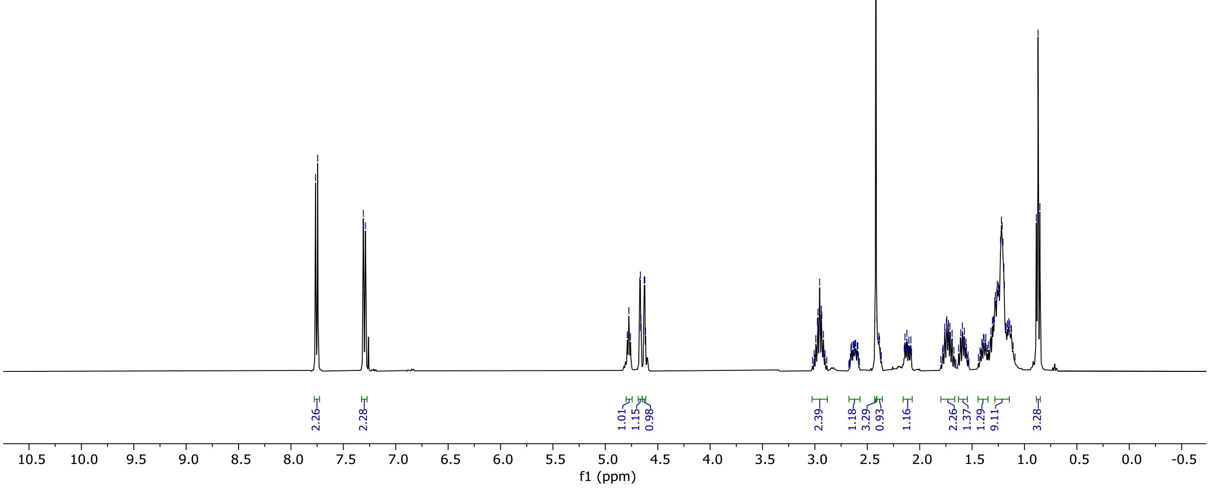

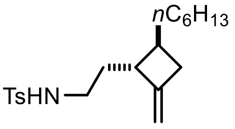

4c

Chemical Formula: $C_{20}H_{31}NO_2S$
^{13}C NMR, 100 MHz, $CDCl_3$

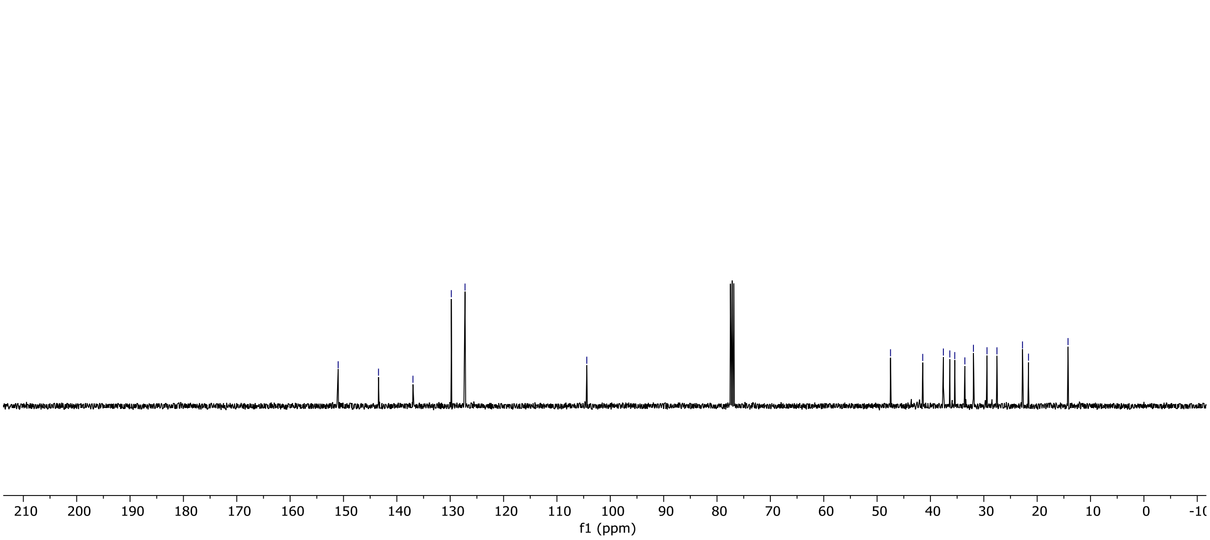

4d

Chemical Formula: $C_{22}H_{27}NO_2S$

^1H NMR, 400 MHz, $CDCl_3$

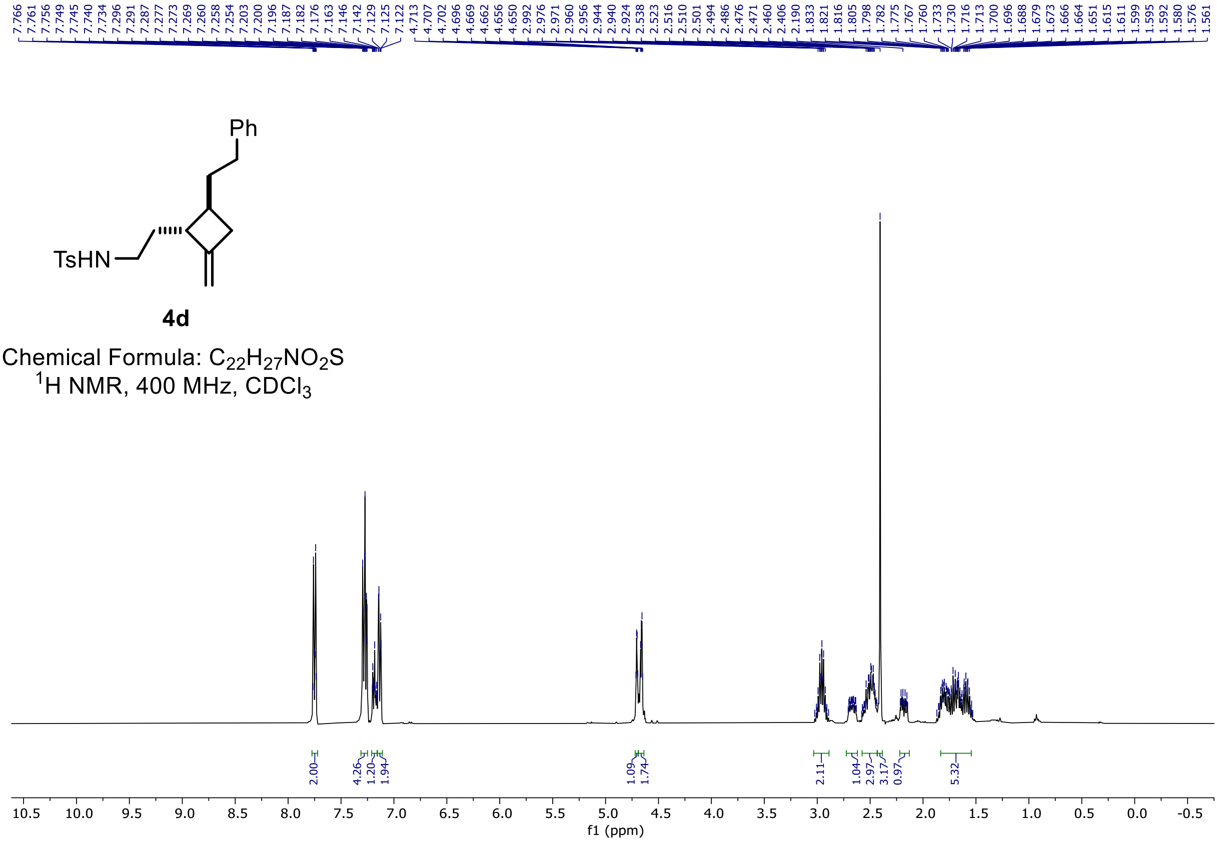

4d

Chemical Formula: $C_{22}H_{27}NO_2S$

^{13}C NMR, 100 MHz, $CDCl_3$

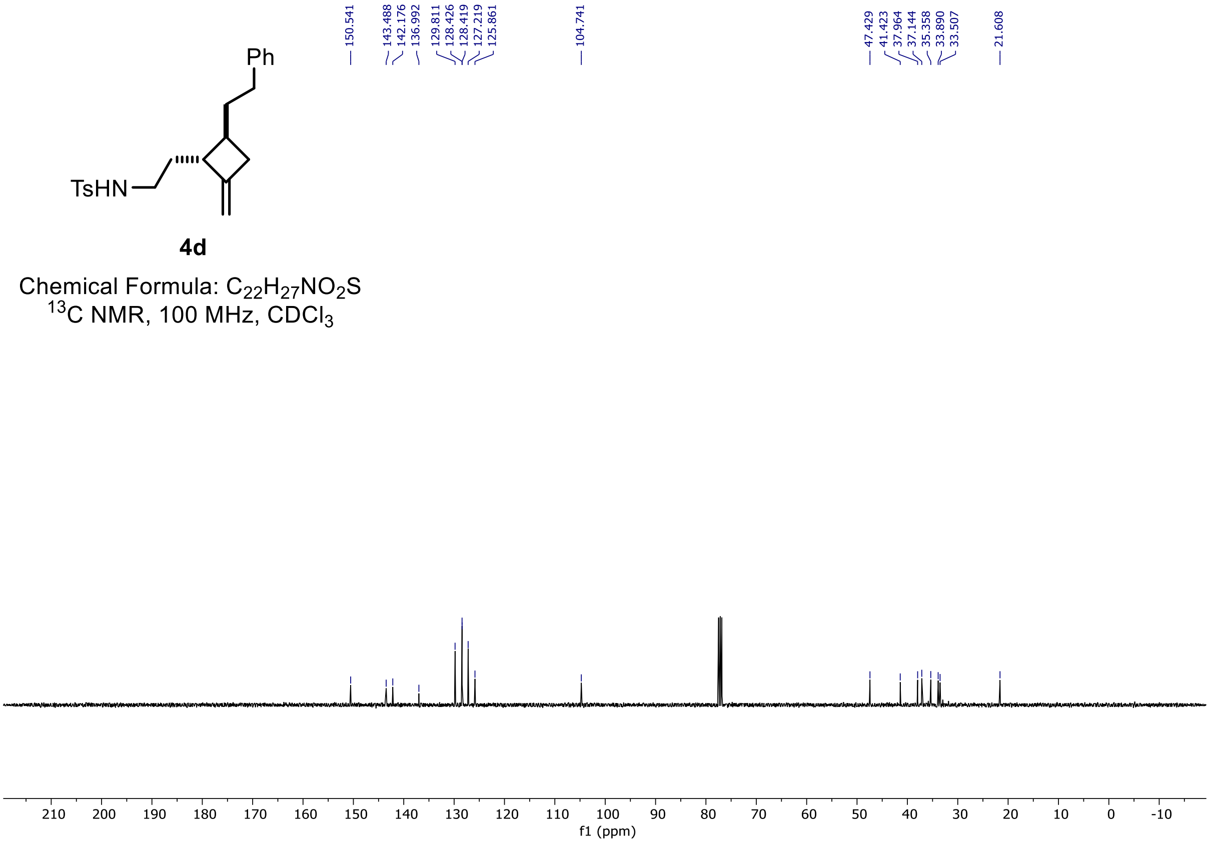

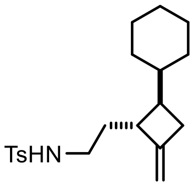

4e

Chemical Formula: $C_{20}H_{29}NO_2S$

^{1}H NMR, 400 MHz, $CDCl_3$

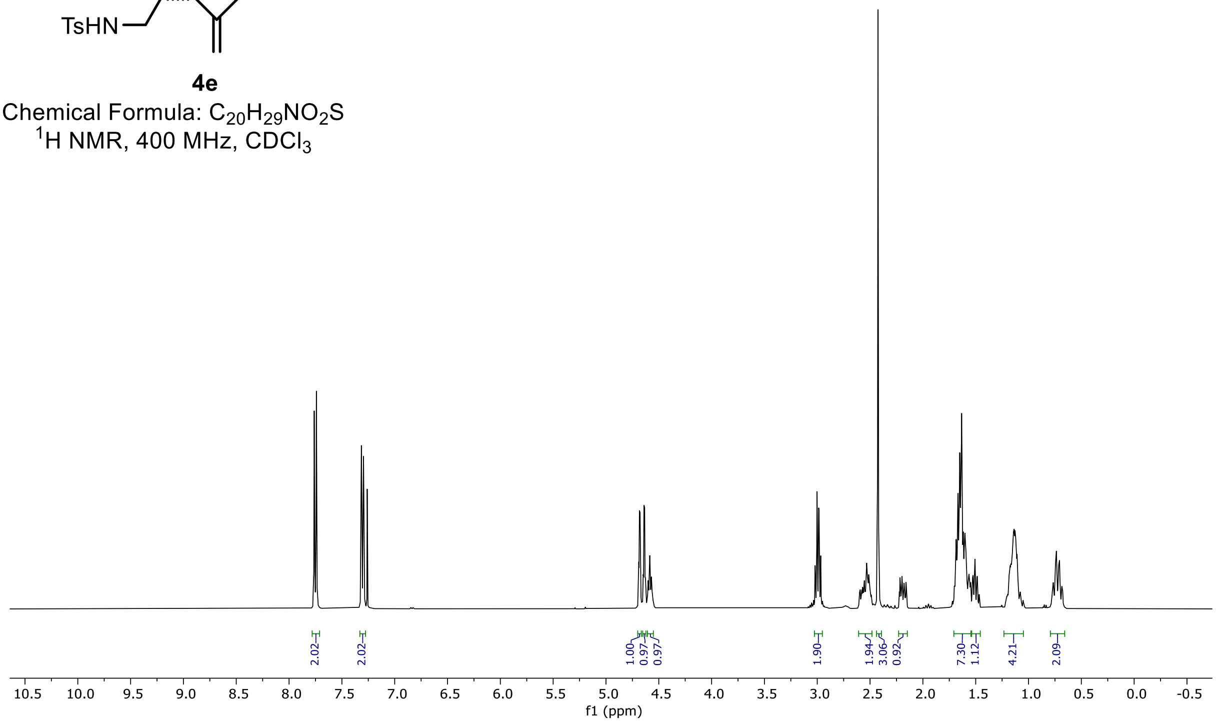

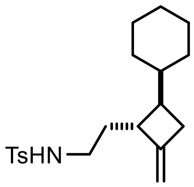

4e

Chemical Formula: $C_{20}H_{29}NO_2S$

^{13}C NMR, 100 MHz, $CDCl_3$

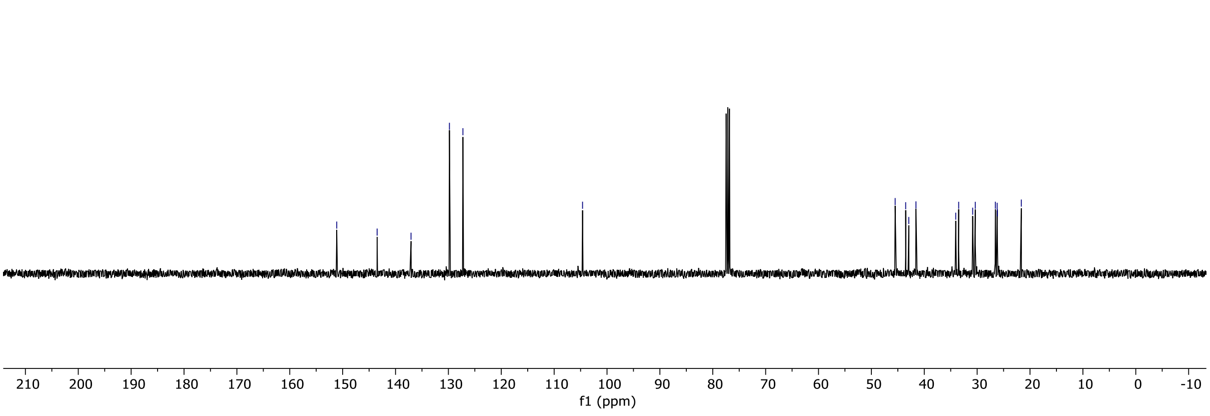

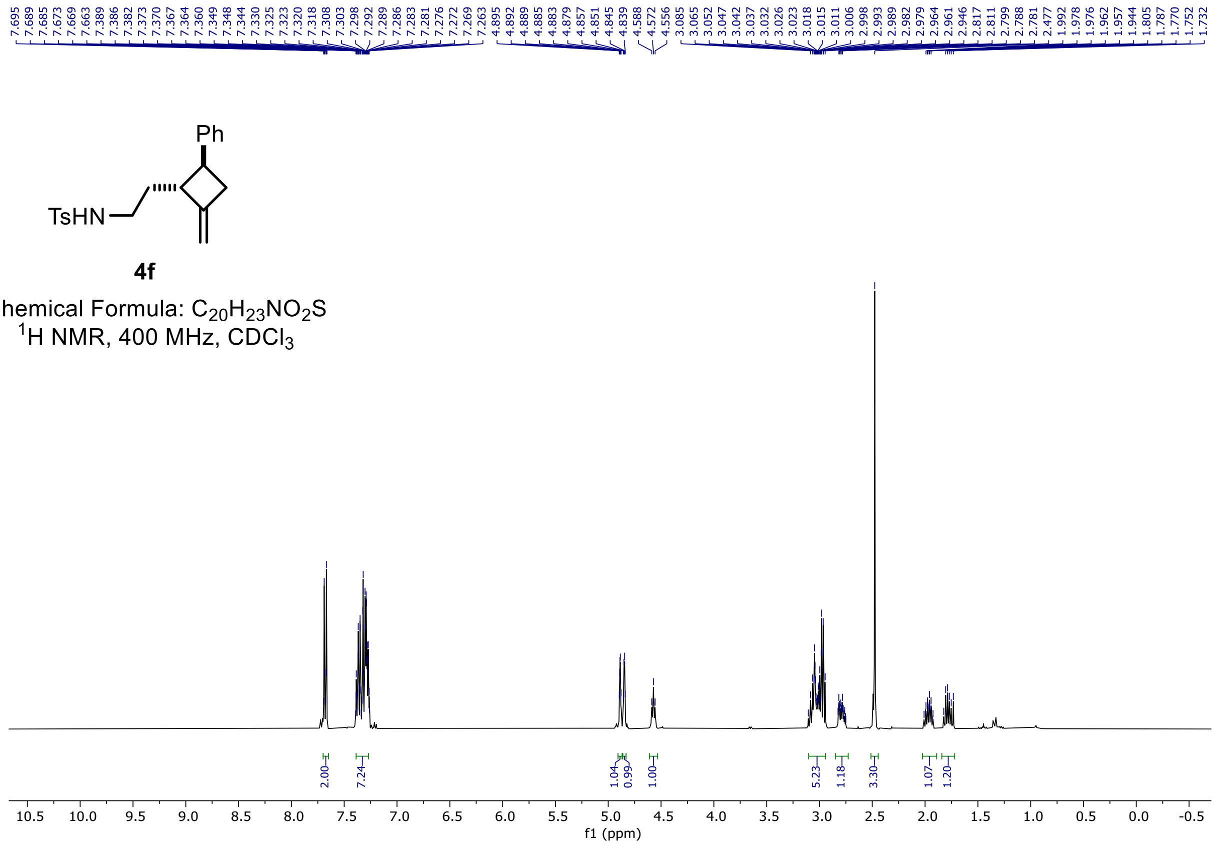

4f

Chemical Formula: $C_{20}H_{23}NO_2S$

^{1}H NMR, 400 MHz, $CDCl_3$

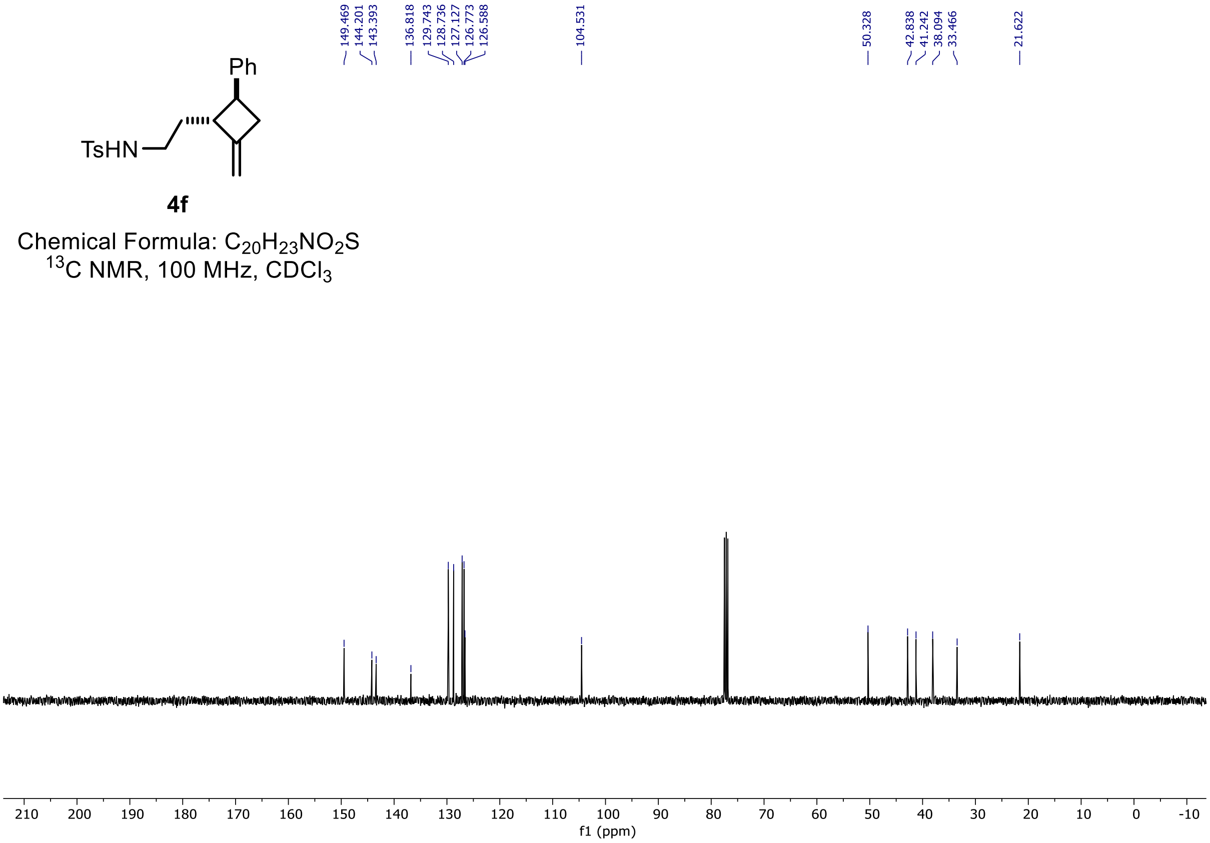

4f

Chemical Formula: $C_{20}H_{23}NO_2S$

^{13}C NMR, 100 MHz, $CDCl_3$

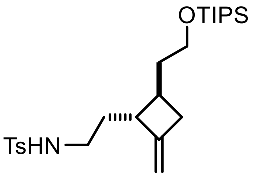

4g

Chemical Formula: $C_{25}H_{43}NO_3SSi$

^{1}H NMR, 600 MHz, $CDCl_3$

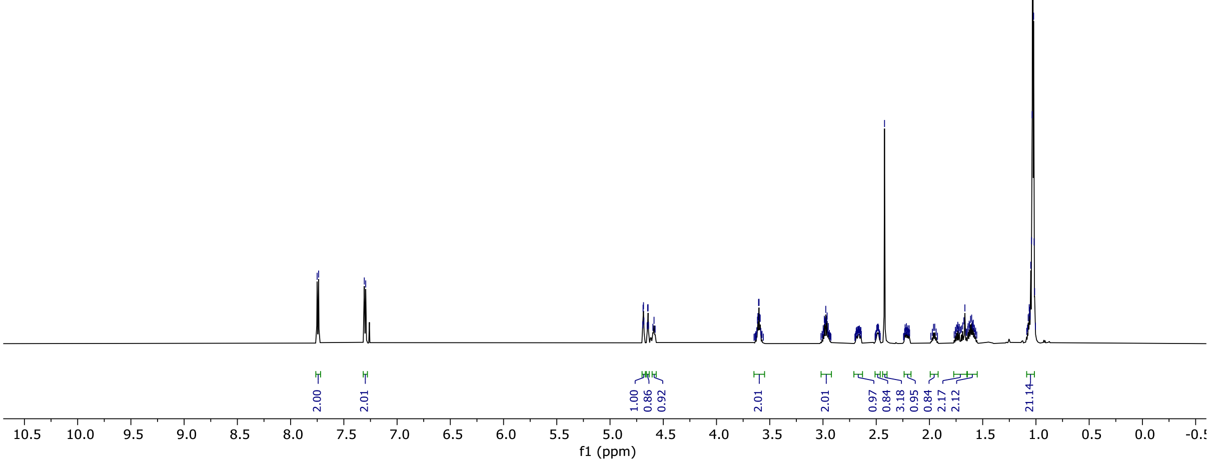

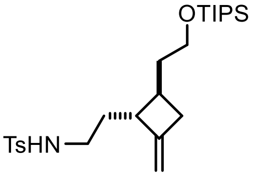

4g

Chemical Formula: $C_{25}H_{43}NO_3SSi$

^{13}C NMR, 100 MHz, $CDCl_3$

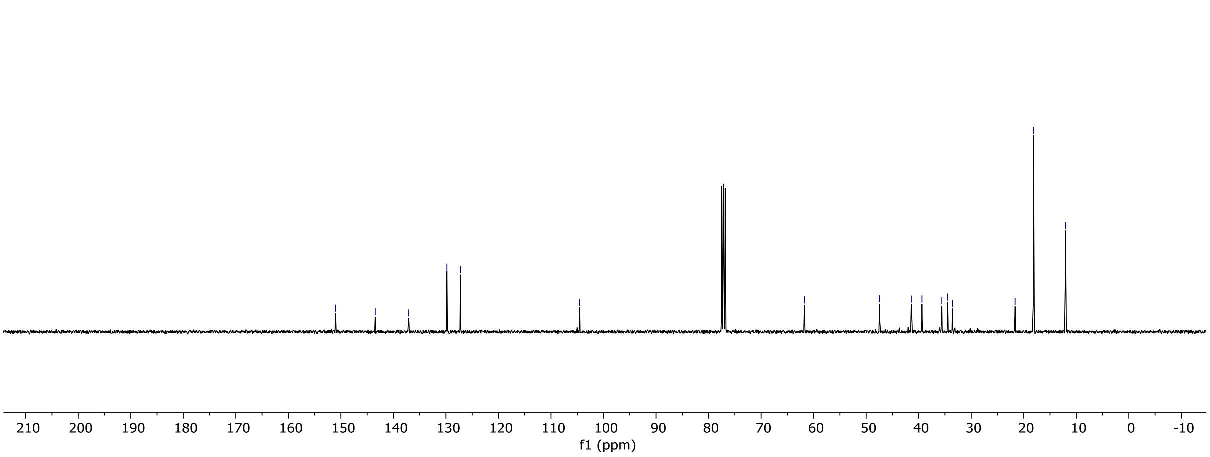

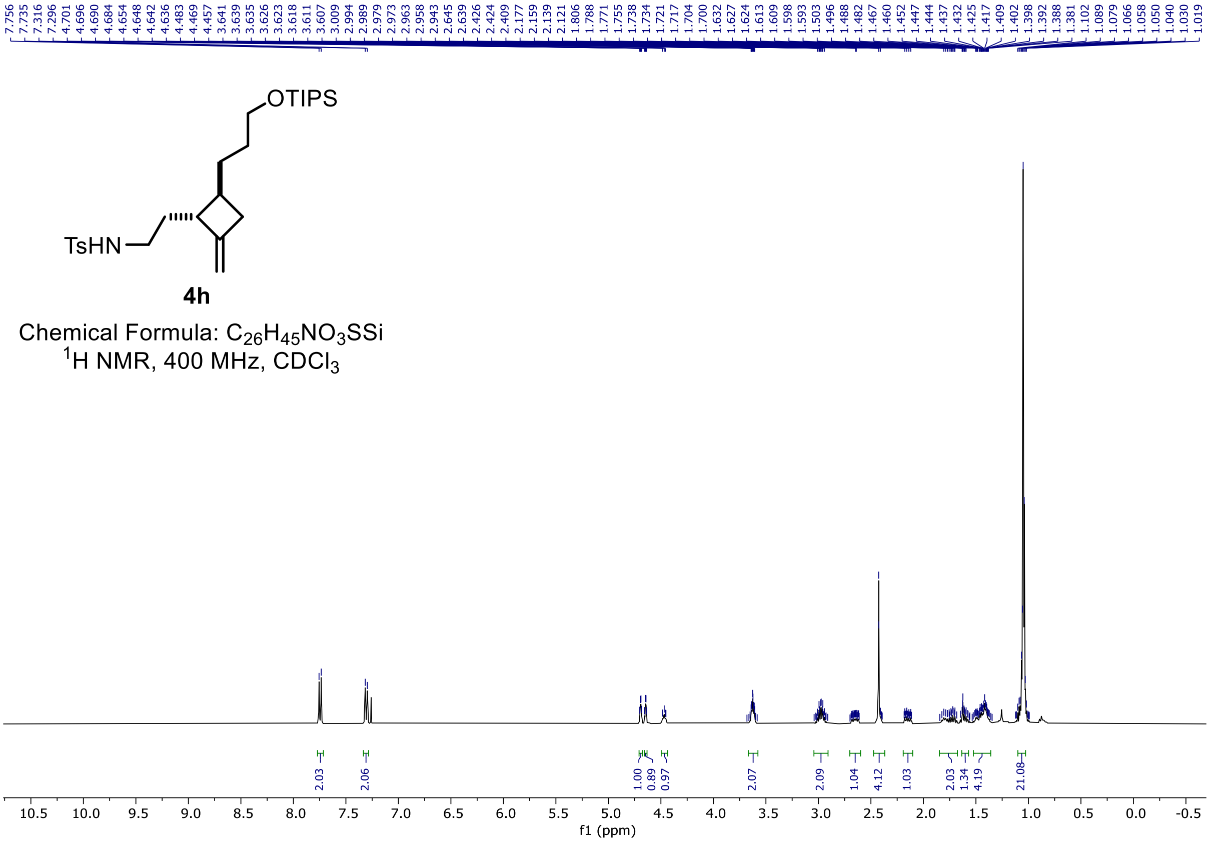
7.756 7.735 7.316 7.296 4.701 4.696 4.690 4.684 4.654 4.648 4.642 4.636 4.483 4.469 4.457 3.641 3.639 3.635 3.626 3.623 3.618 3.611 3.607 3.009 2.994 2.989 2.979 2.973 2.963 2.958 2.943 2.645 2.639 2.426 2.424 2.409 2.177 2.159 2.139 2.121 1.806 1.788 1.771 1.755 1.738 1.734 1.721 1.717 1.704 1.700 1.632 1.627 1.624 1.613 1.609 1.598 1.593 1.503 1.496 1.488 1.482 1.467 1.460 1.452 1.447 1.444 1.437 1.432 1.425 1.417 1.409 1.402 1.398 1.392 1.388 1.381 1.102 1.089 1.079 1.066 1.058 1.050 1.040 1.030 1.019
OTIPS
TsHN
4h
Chemical Formula: C26H45NO3SSi
1H NMR, 400 MHz, CDCl3
2.03 2.06 1.00 0.89 0.97 2.07 2.09 1.04 4.12 1.03 2.03 1.34 4.19 21.08
10.5 10.0 9.5 9.0 8.5 8.0 7.5 7.0 6.5 6.0 5.5 5.0 4.5 4.0 3.5 3.0 2.5 2.0 1.5 1.0 0.5 0.0 -0.5
f1 (ppm)

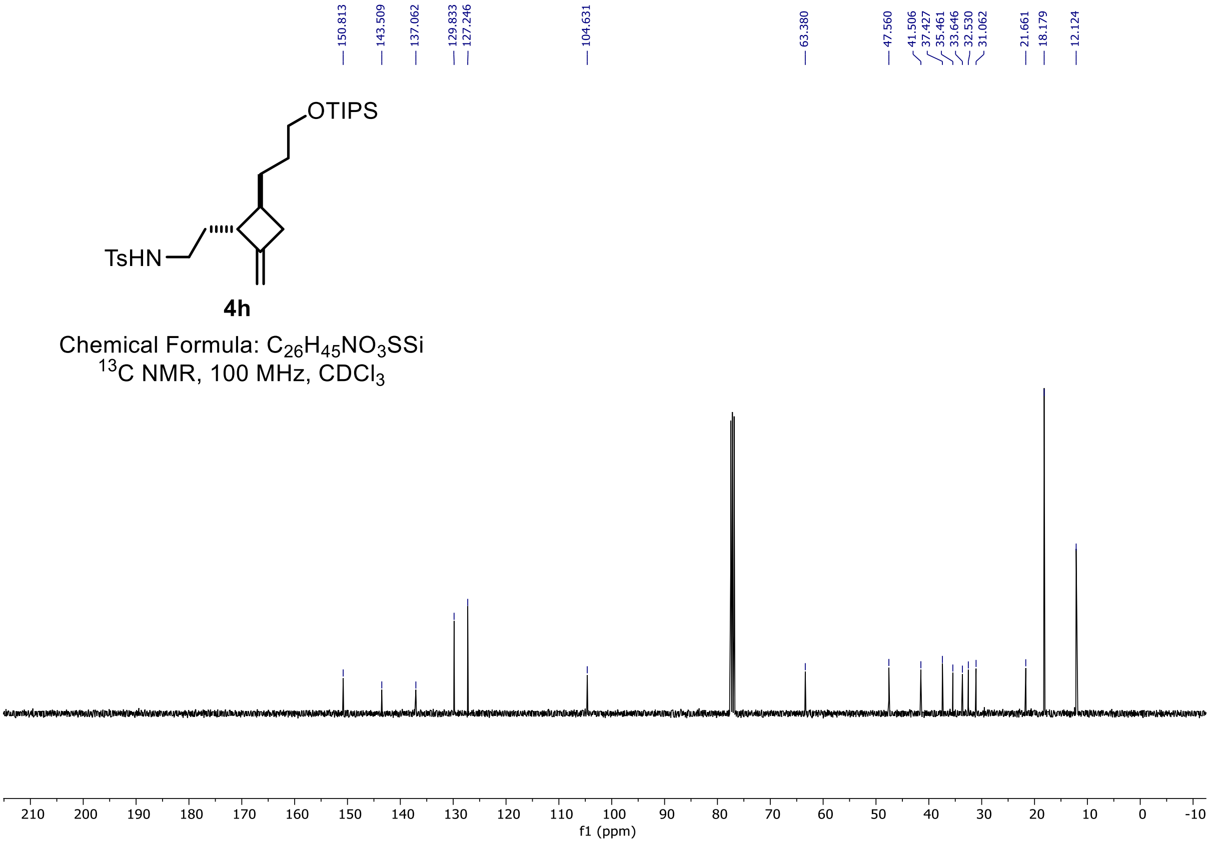
150.813 143.509 137.062 129.833 127.246 104.631 63.380 47.560 41.506 37.427 35.461 33.646 32.530 31.062 21.661 18.179 12.124
OTIPS
TsHN
4h
Chemical Formula: C26H45NO3SSi
13C NMR, 100 MHz, CDCl3
210 200 190 180 170 160 150 140 130 120 110 100 90 80 70 60 50 40 30 20 10 0 -10
f1 (ppm)

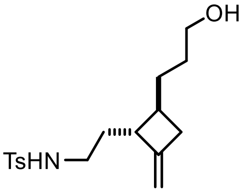

4h-A

Chemical Formula: $C_{17}H_{25}NO_3S$

^1H NMR, 400 MHz, $CDCl_3$

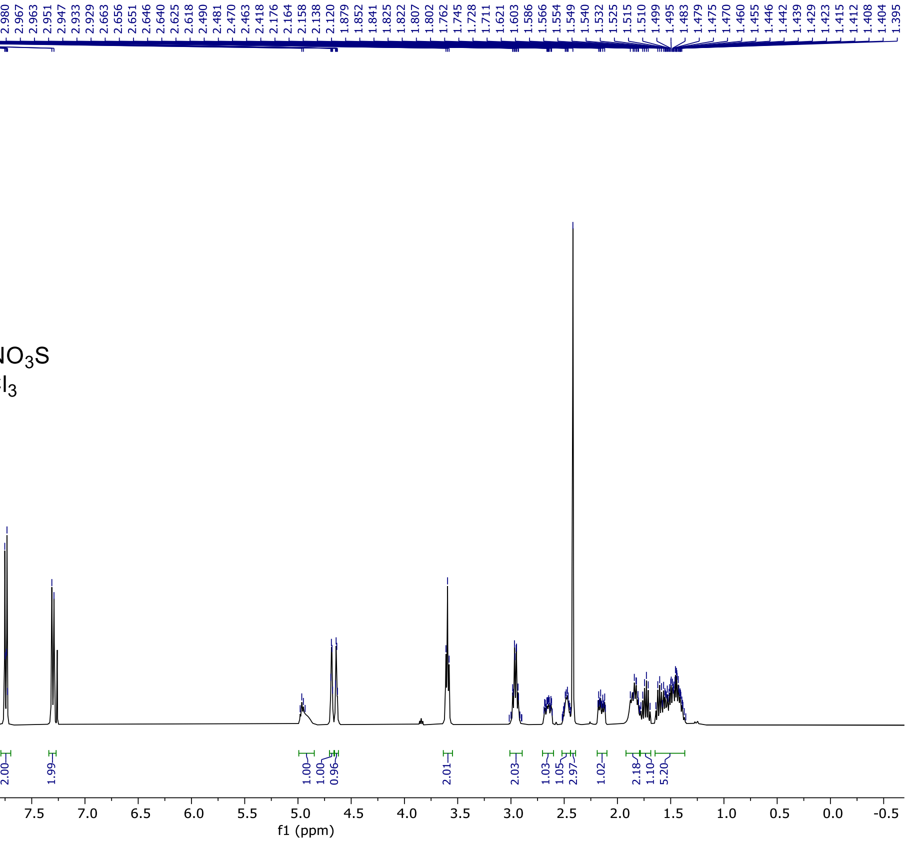

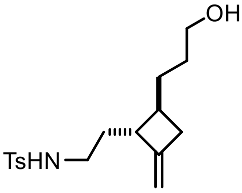

4h-A

Chemical Formula: $C_{17}H_{25}NO_3S$

^{13}C NMR, 100 MHz, $CDCl_3$

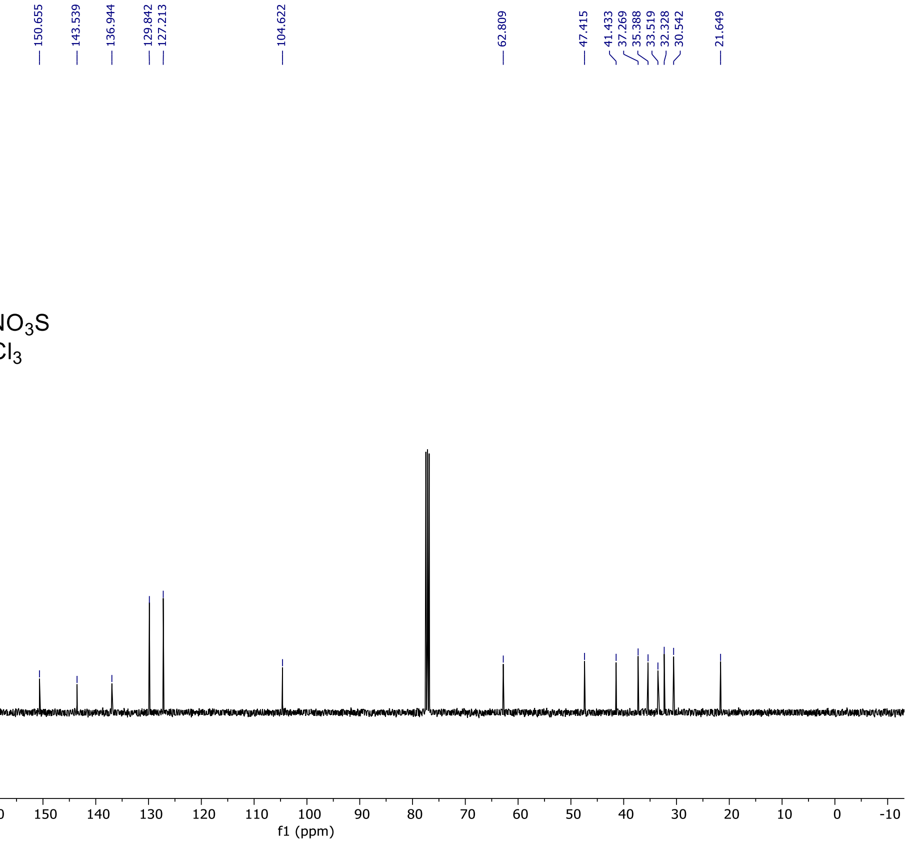

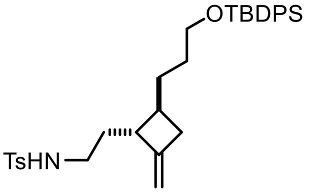

4i

Chemical Formula: $C_{33}H_{43}NO_3SSi$
^{1}H NMR, 400 MHz, $CDCl_3$

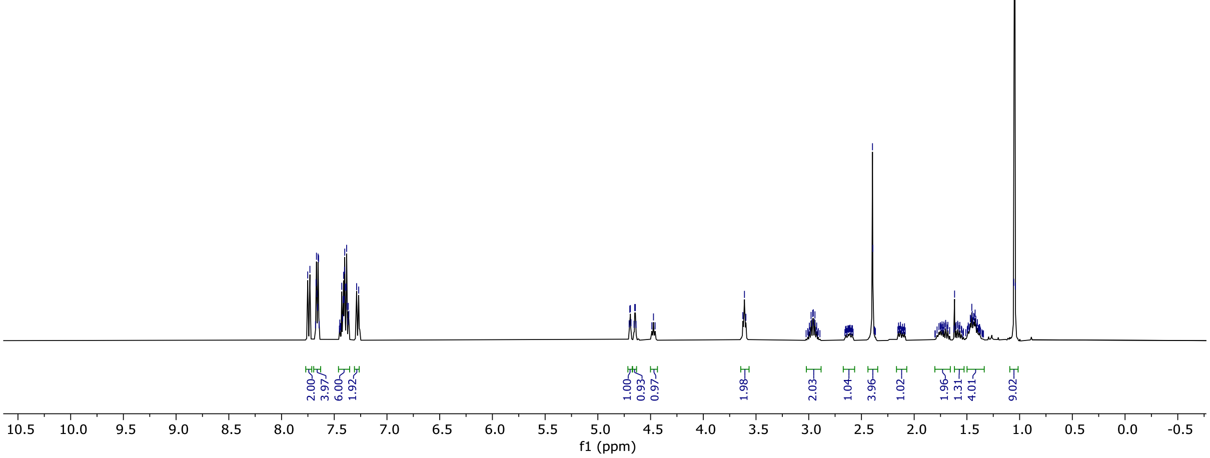

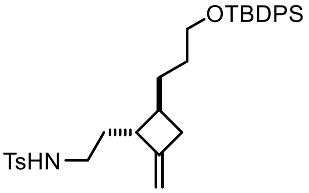

4i

Chemical Formula: $C_{33}H_{43}NO_3SSi$
^{13}C NMR, 100 MHz, $CDCl_3$

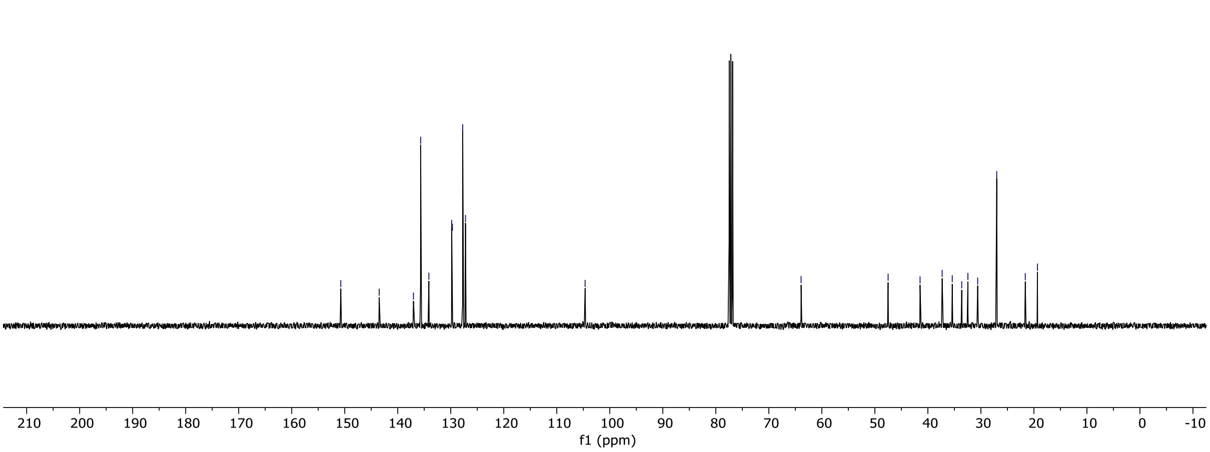

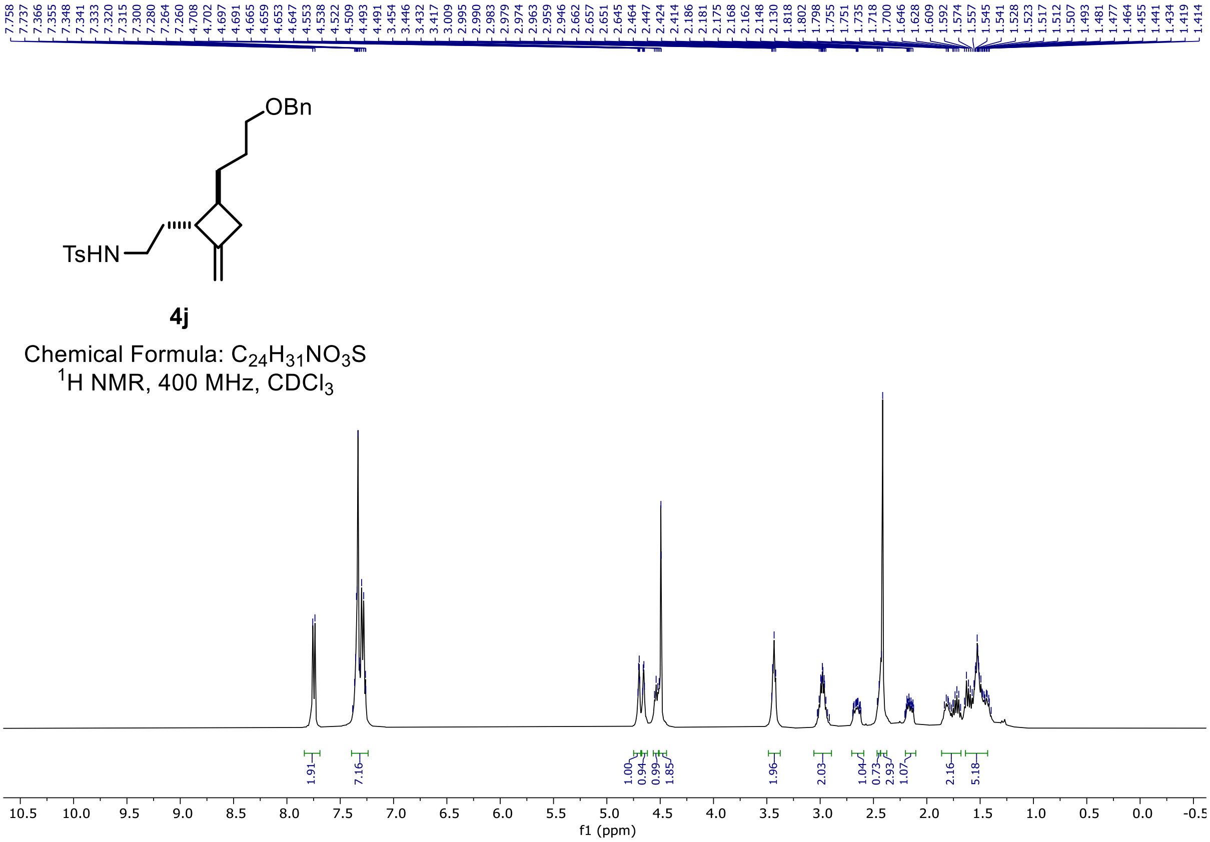

4j

Chemical Formula: $C_{24}H_{31}NO_3S$

^{1}H NMR, 400 MHz, $CDCl_3$

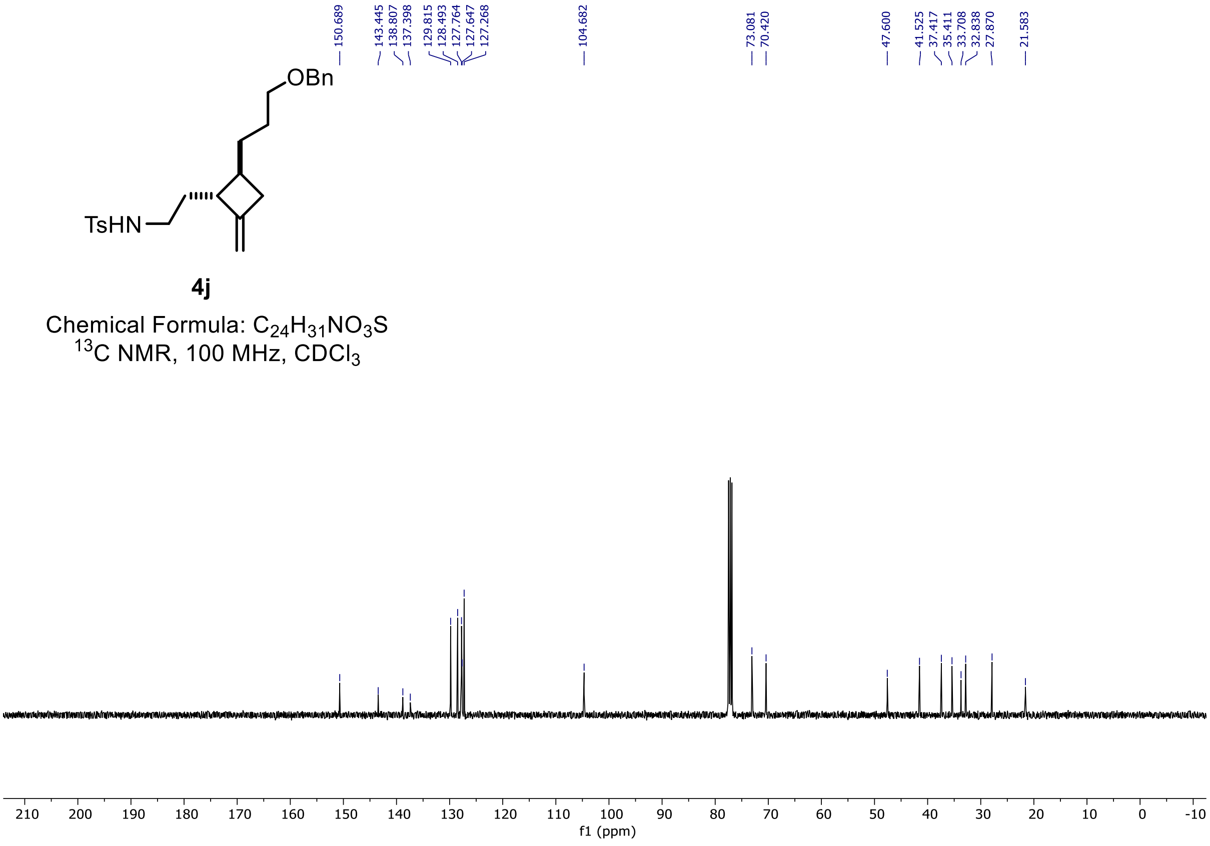

4j

Chemical Formula: $C_{24}H_{31}NO_3S$

^{13}C NMR, 100 MHz, $CDCl_3$

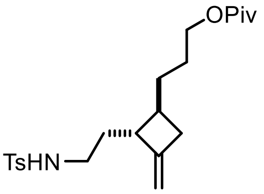

4k

Chemical Formula: $C_{22}H_{33}NO_4S$

^1H NMR, 400 MHz, $CDCl_3$

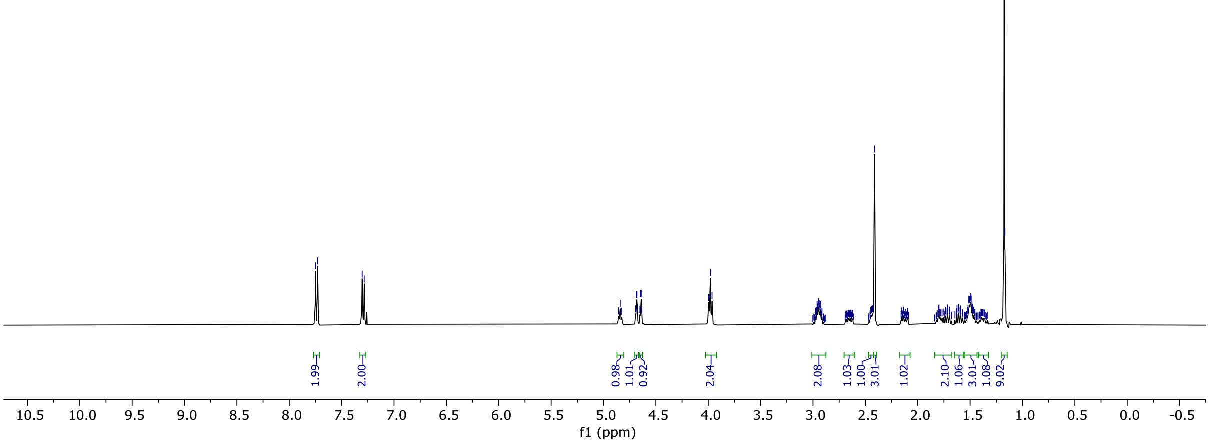

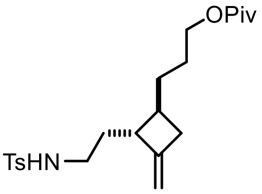

4k

Chemical Formula: $C_{22}H_{33}NO_4S$

^{13}C NMR, 100 MHz, $CDCl_3$

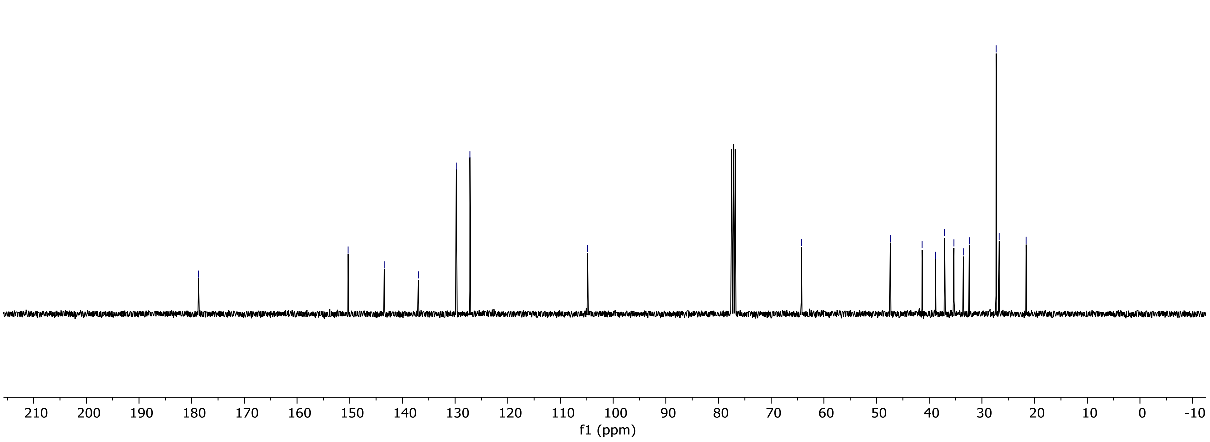

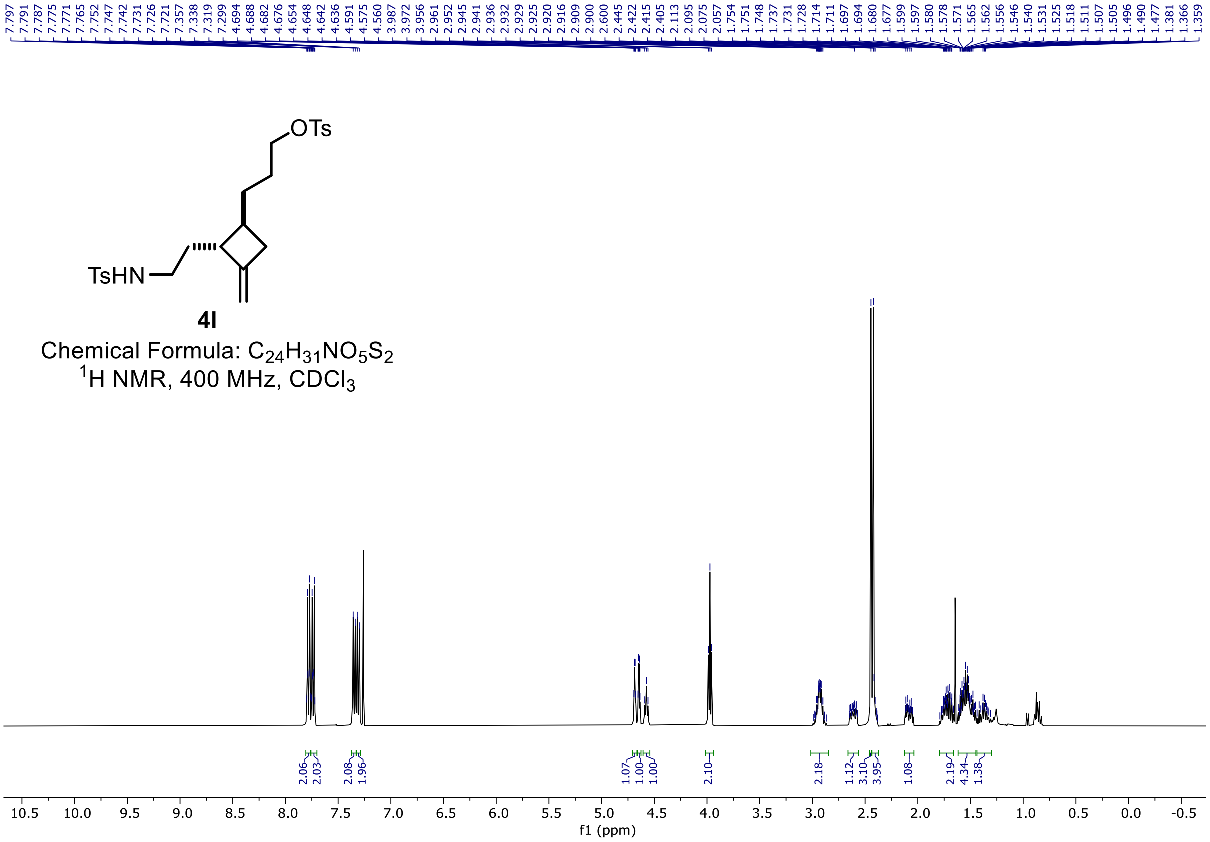

4l

Chemical Formula: $C_{24}H_{31}NO_5S_2$

^{1}H NMR, 400 MHz, $CDCl_3$

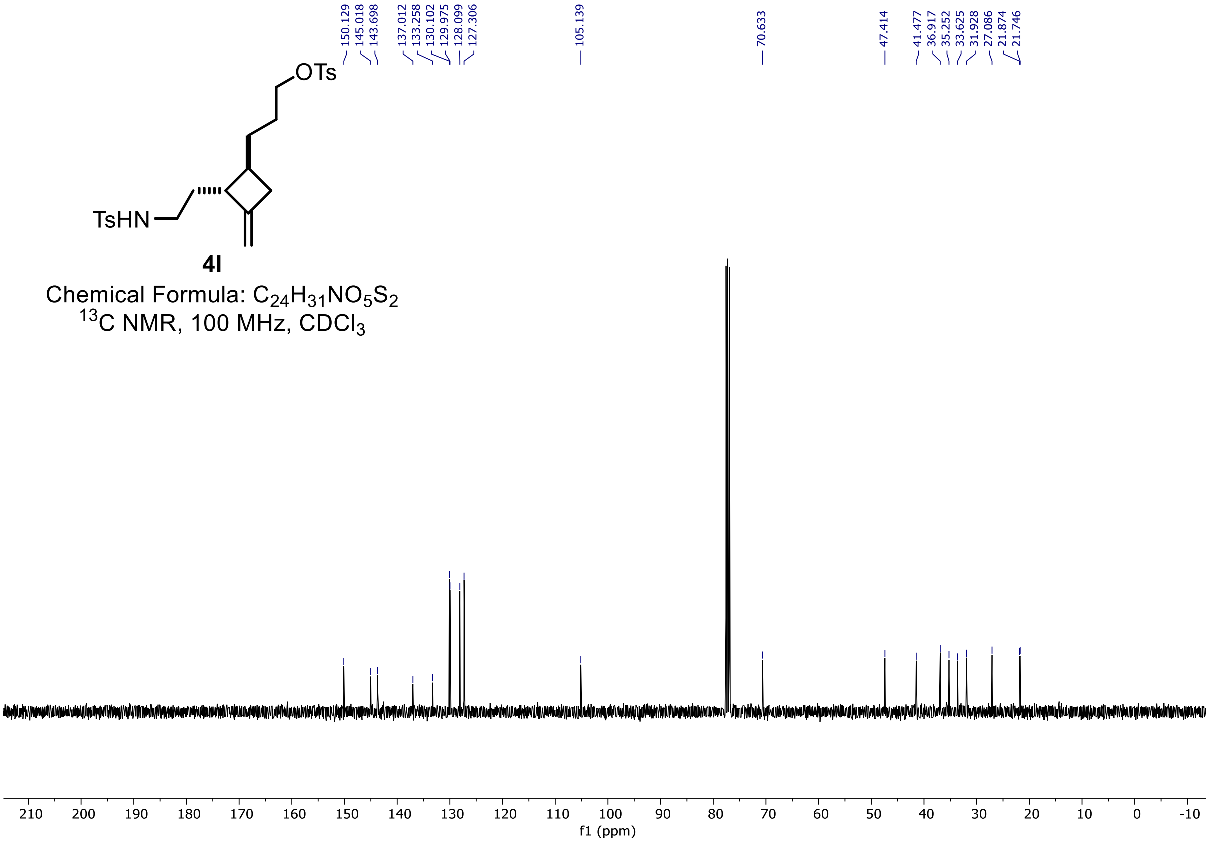

4l

Chemical Formula: $C_{24}H_{31}NO_5S_2$

^{13}C NMR, 100 MHz, $CDCl_3$

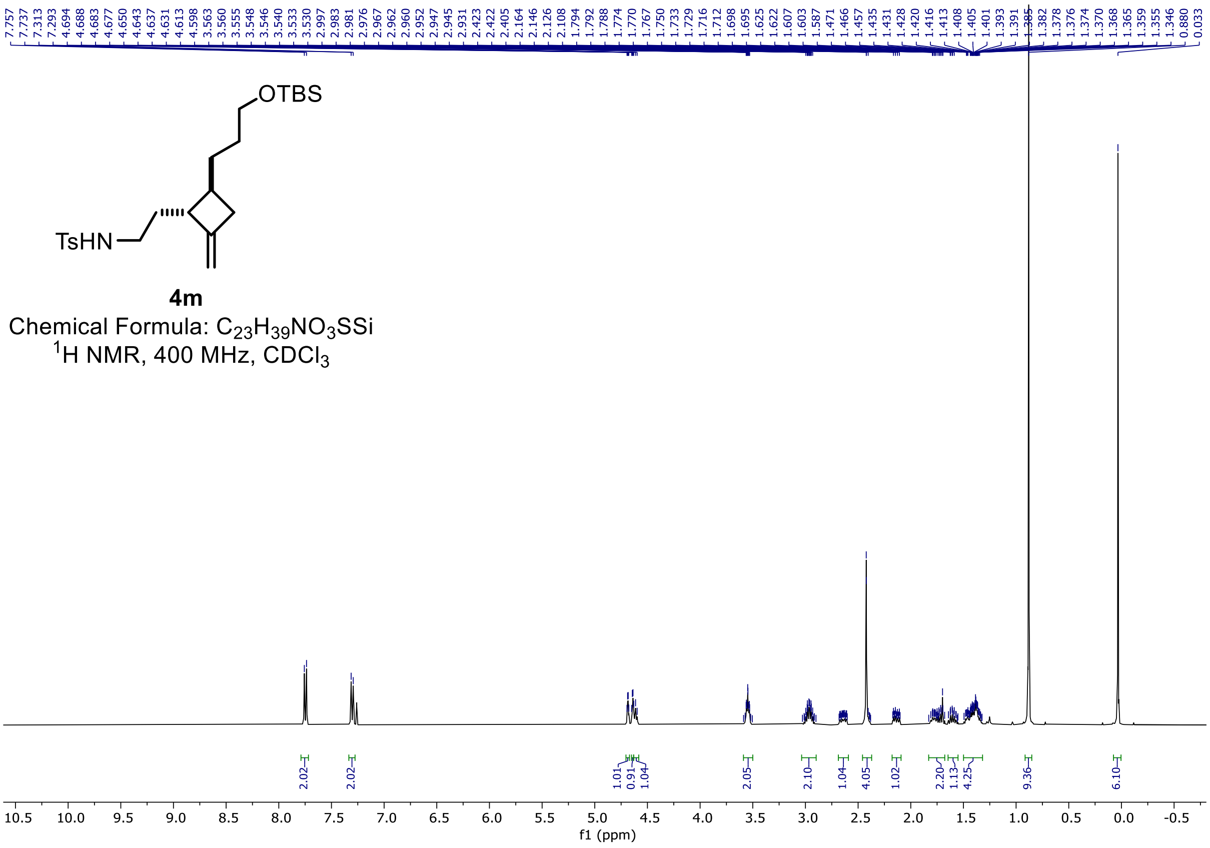

4m
Chemical Formula: $C_{23}H_{39}NO_3SSi$
^1H NMR, 400 MHz, $CDCl_3$

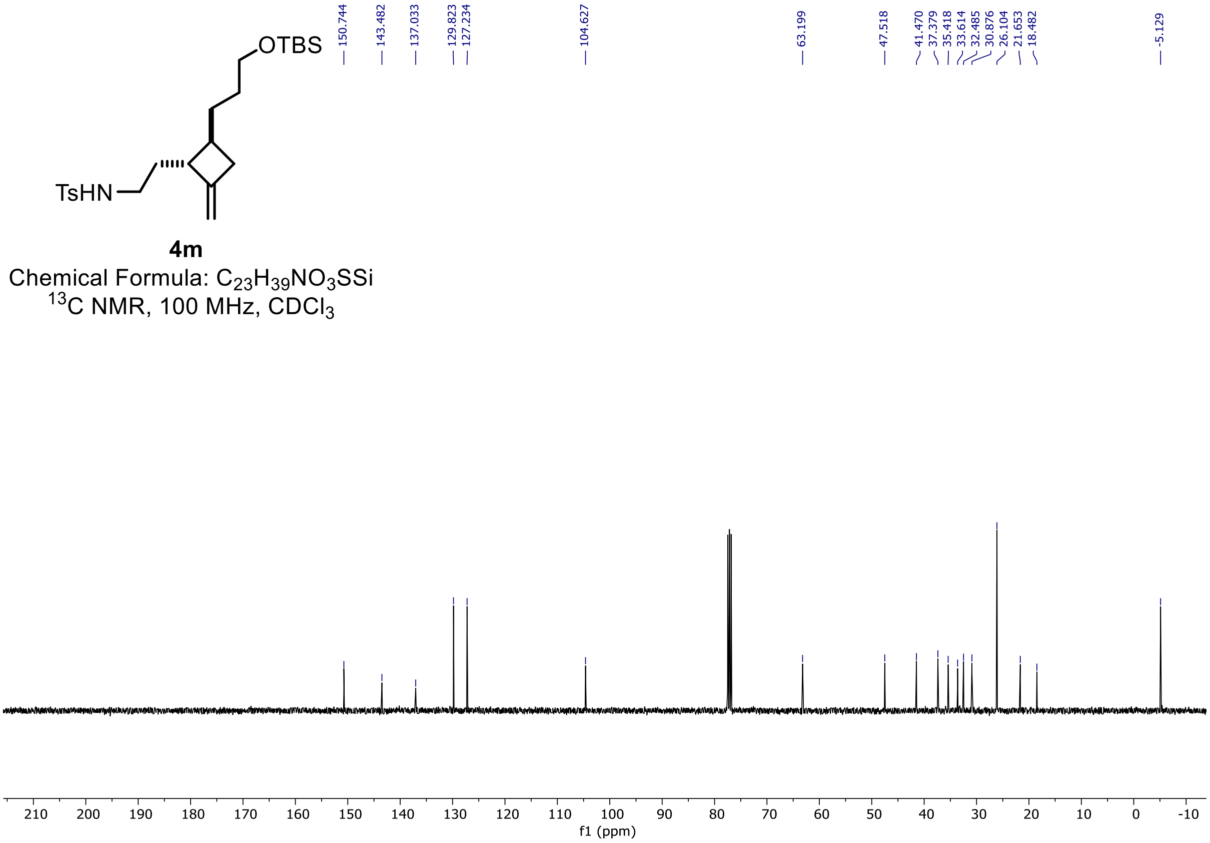

4m
Chemical Formula: $C_{23}H_{39}NO_3SSi$
^{13}C NMR, 100 MHz, $CDCl_3$

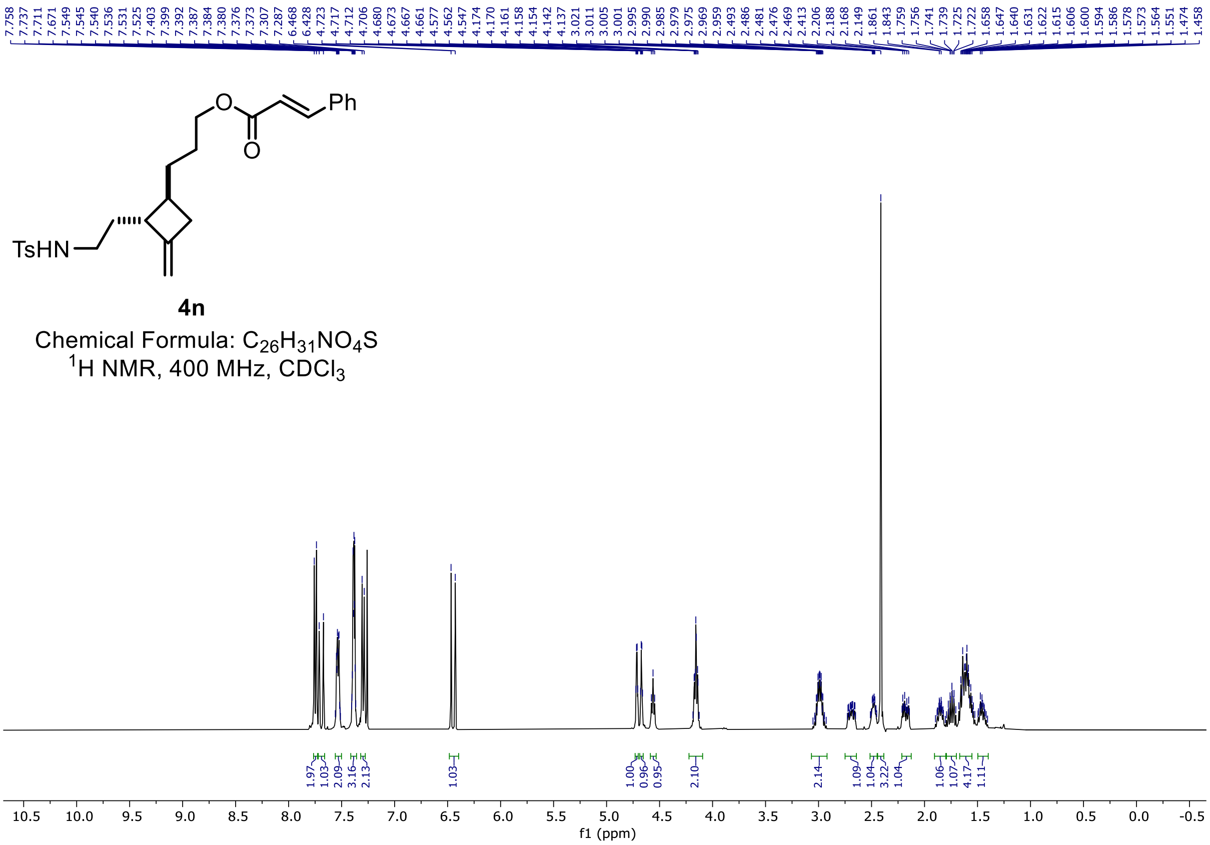

4n

Chemical Formula: $C_{26}H_{31}NO_4S$

^{1}H NMR, 400 MHz, $CDCl_3$

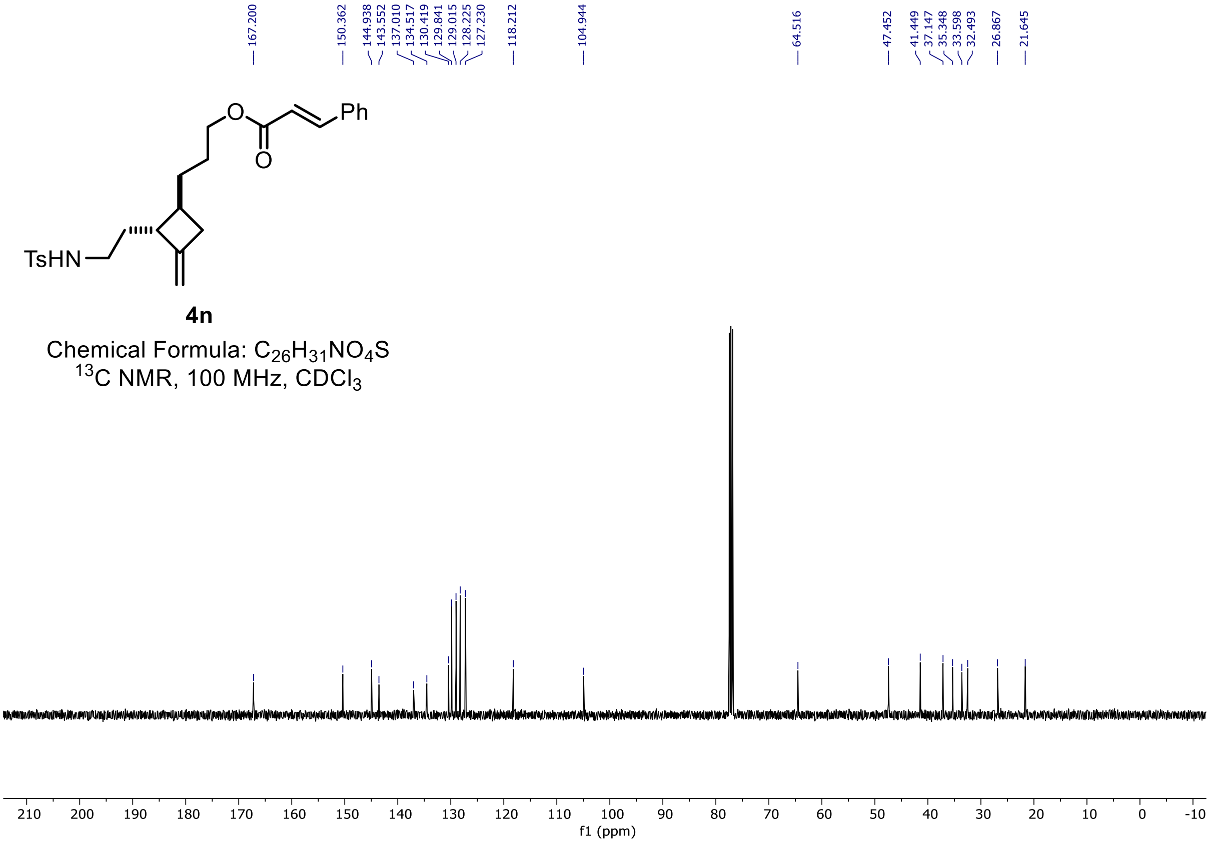

4n

Chemical Formula: $C_{26}H_{31}NO_4S$

^{13}C NMR, 100 MHz, $CDCl_3$

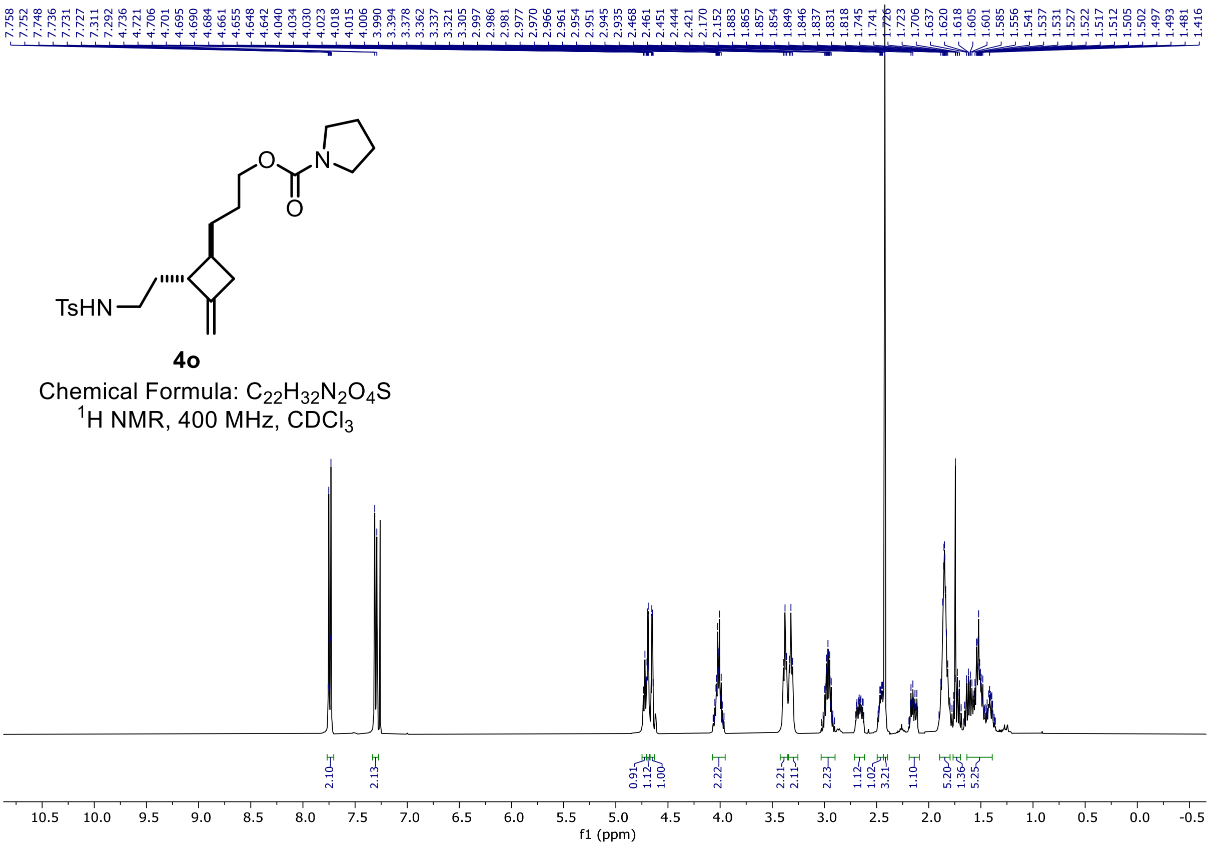
7.758
7.752
7.748
7.736
7.731
7.727
7.311
7.292
4.736
4.721
4.706
4.701
4.695
4.690
4.684
4.661
4.655
4.648
4.642
4.040
4.034
4.030
4.023
4.018
4.015
4.006
3.990
3.394
3.378
3.362
3.337
3.321
3.305
2.997
2.986
2.981
2.977
2.970
2.966
2.961
2.954
2.951
2.945
2.935
2.468
2.461
2.451
2.444
2.421
2.170
2.152
1.883
1.865
1.857
1.854
1.849
1.846
1.837
1.831
1.818
1.745
1.741
1.726
1.723
1.706
1.637
1.620
1.618
1.605
1.601
1.585
1.556
1.541
1.537
1.531
1.527
1.522
1.517
1.512
1.505
1.502
1.497
1.493
1.481
1.416
TsHN
O
N
O
4o
Chemical Formula: C22H32N2O4S
1H NMR, 400 MHz, CDCl3
2.10
2.13
0.91
1.12
1.00
2.22
2.21
2.11
2.23
1.12
1.02
3.21
1.10
5.20
1.36
5.25
10.5 10.0 9.5 9.0 8.5 8.0 7.5 7.0 6.5 6.0 5.5 5.0 4.5 4.0 3.5 3.0 2.5 2.0 1.5 1.0 0.5 0.0 -0.5
f1 (ppm)

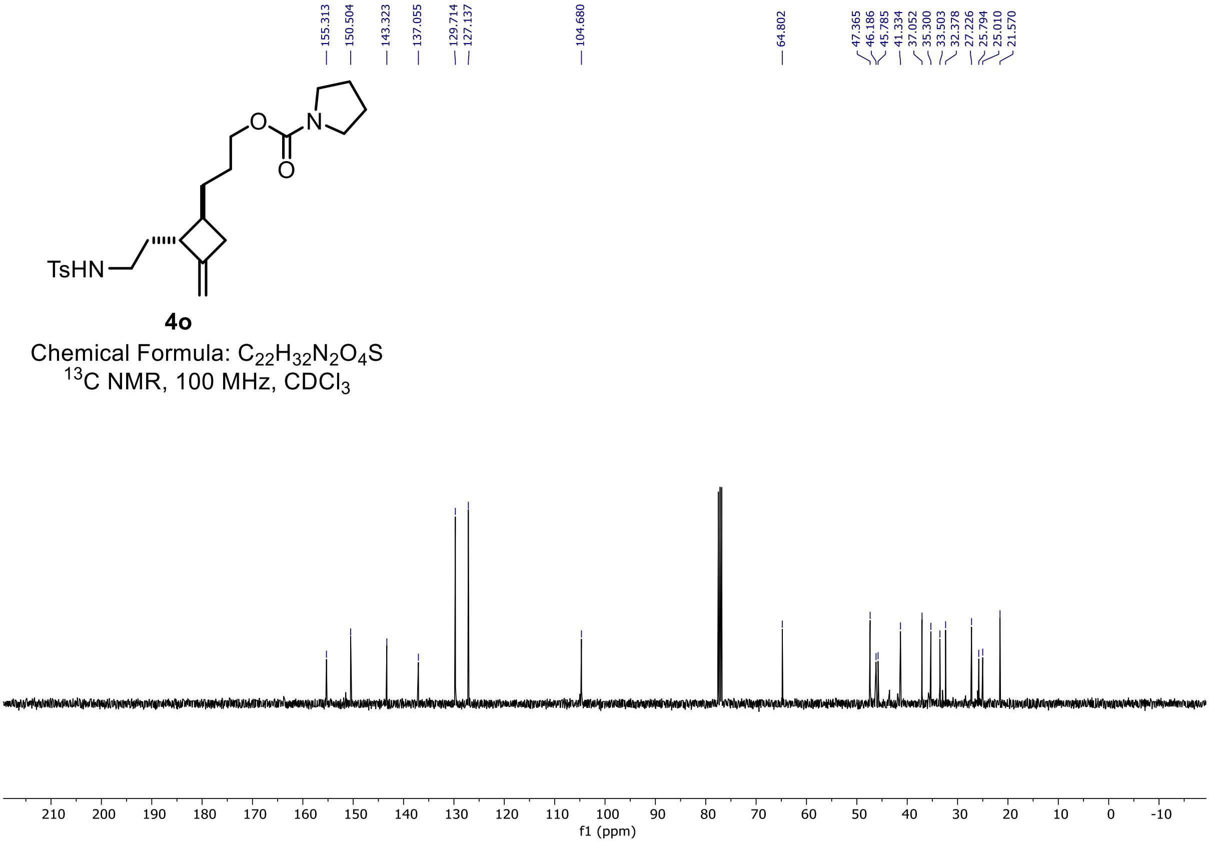
155.313
150.504
143.323
137.055
129.714
127.137
104.680
64.802
47.365
46.186
45.785
41.334
37.052
35.300
33.503
32.378
27.226
25.794
25.010
21.570
TsHN
O
N
O
4o
Chemical Formula: C22H32N2O4S
13C NMR, 100 MHz, CDCl3
210 200 190 180 170 160 150 140 130 120 110 100 90 80 70 60 50 40 30 20 10 0 -10
f1 (ppm)

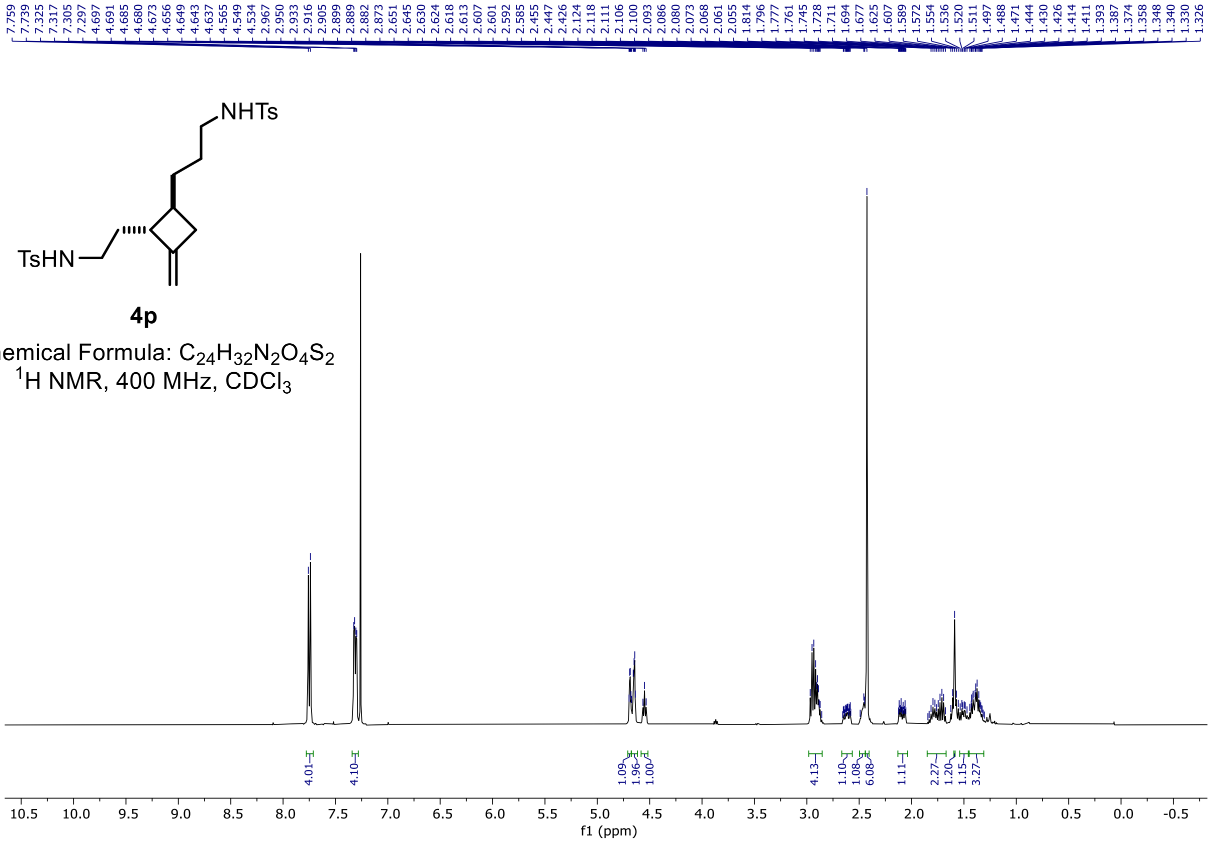

4p

Chemical Formula: $C_{24}H_{32}N_2O_4S_2$

^{1}H NMR, 400 MHz, $CDCl_3$

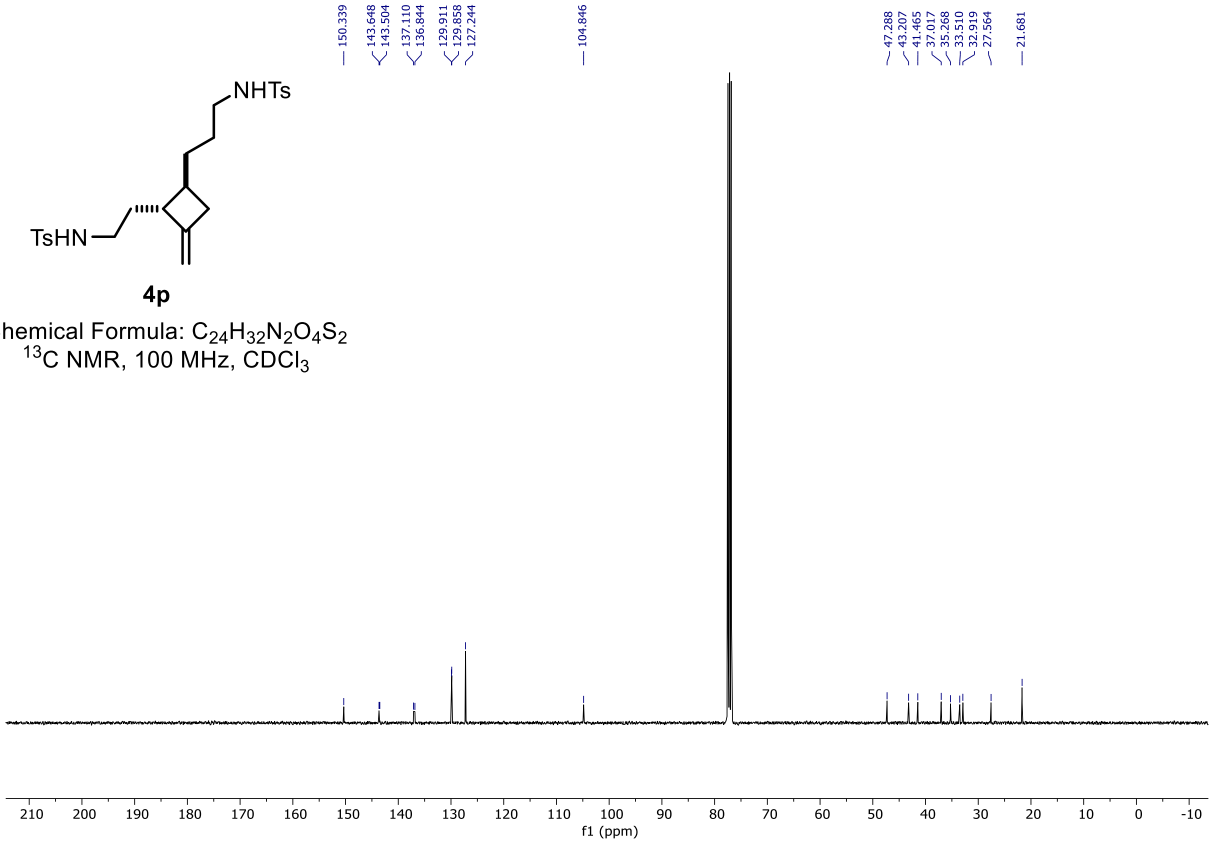

4p

Chemical Formula: $C_{24}H_{32}N_2O_4S_2$

^{13}C NMR, 100 MHz, $CDCl_3$

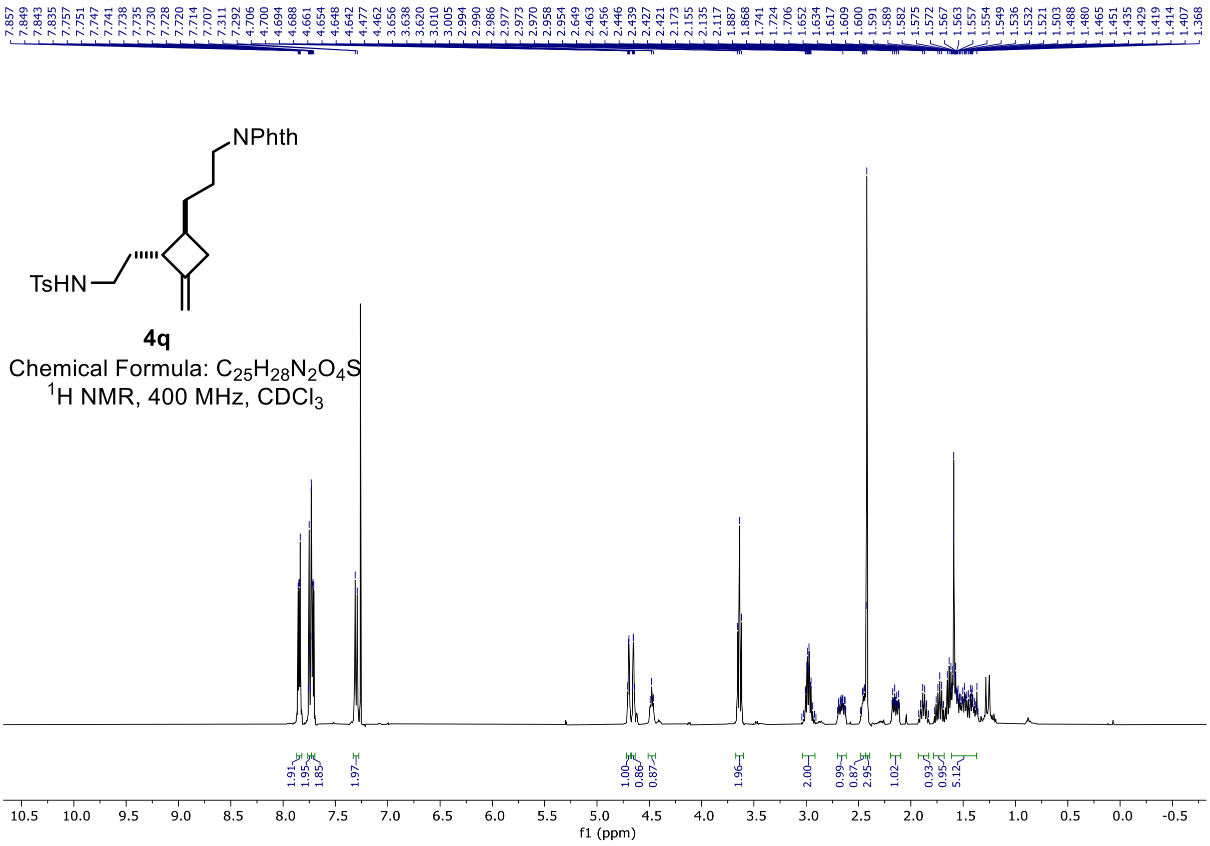
7.857 7.849 7.843 7.835 7.757 7.751 7.741 7.738 7.735 7.730 7.728 7.720 7.714 7.707 7.311 7.292 4.706 4.700 4.694 4.688 4.661 4.654 4.648 4.642 4.477 4.462 3.656 3.638 3.620 3.010 3.005 2.994 2.990 2.986 2.977 2.973 2.970 2.958 2.954 2.649 2.463 2.456 2.446 2.439 2.427 2.421 2.173 2.155 2.135 2.117 1.887 1.868 1.741 1.724 1.706 1.652 1.634 1.617 1.609 1.600 1.591 1.589 1.582 1.575 1.572 1.567 1.563 1.557 1.554 1.549 1.536 1.532 1.521 1.503 1.488 1.480 1.465 1.451 1.435 1.429 1.419 1.414 1.407 1.368
NPhth
TsHN
4q
Chemical Formula: $C_{25}H_{28}N_2O_4S$
^1H NMR, 400 MHz, $CDCl_3$
1.91 1.95 1.85 1.97 1.00 0.86 0.87 1.96 2.00 0.99 0.87 2.95 1.02 0.93 0.95 5.12
10.5 10.0 9.5 9.0 8.5 8.0 7.5 7.0 6.5 6.0 5.5 5.0 4.5 4.0 3.5 3.0 2.5 2.0 1.5 1.0 0.5 0.0 -0.5
f1 (ppm)

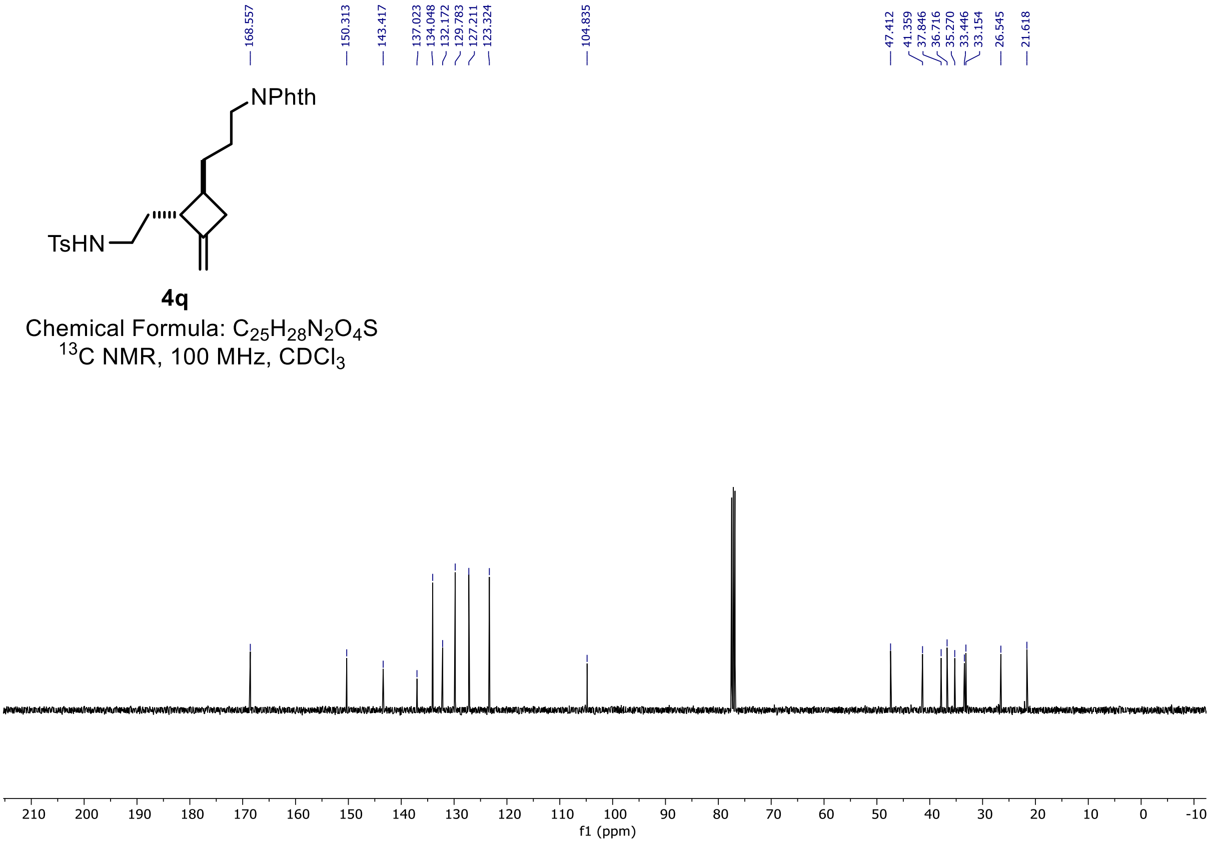
168.557 150.313 143.417 137.023 134.048 132.172 129.783 127.211 123.324 104.835 47.412 41.359 37.846 36.716 35.270 33.446 33.154 26.545 21.618
NPhth
TsHN
4q
Chemical Formula: $C_{25}H_{28}N_2O_4S$
^{13}C NMR, 100 MHz, $CDCl_3$
210 200 190 180 170 160 150 140 130 120 110 100 90 80 70 60 50 40 30 20 10 0 -10
f1 (ppm)

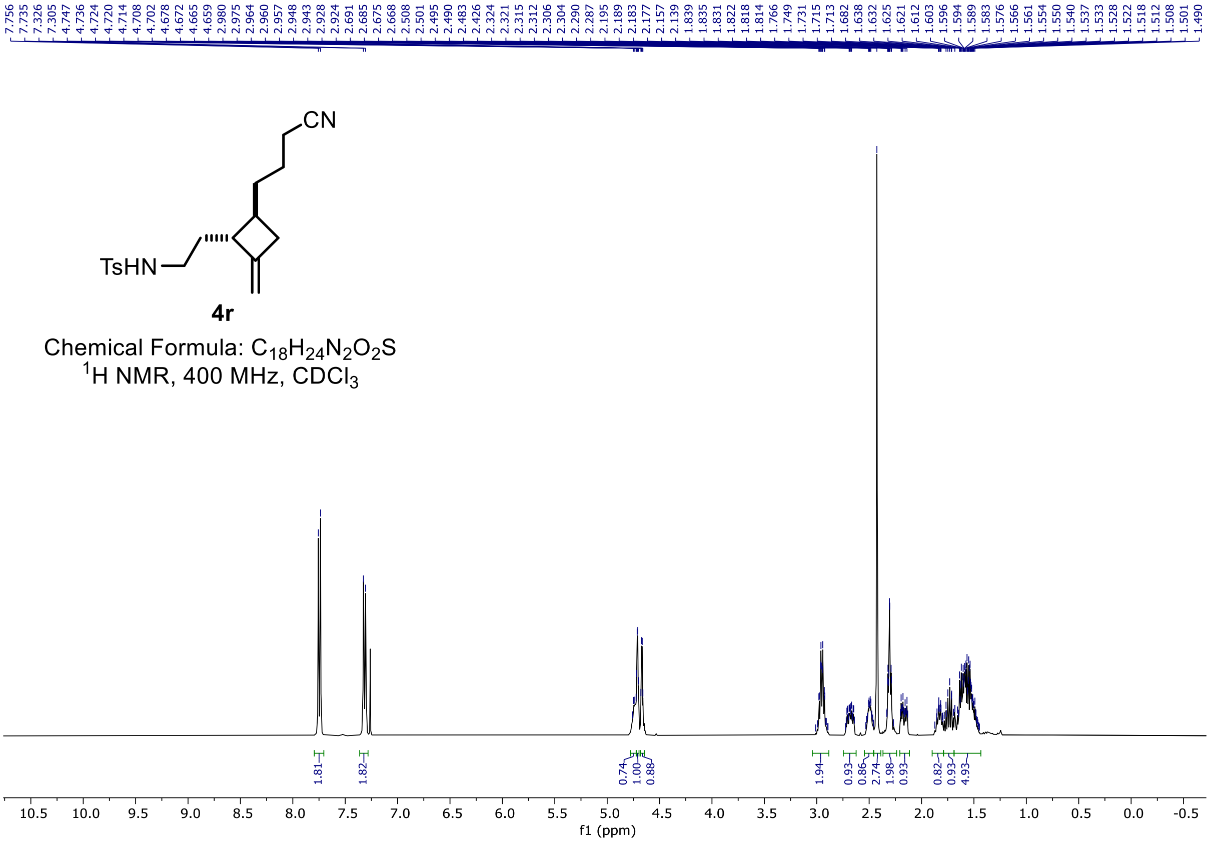
7.756 7.735 7.326 7.305 4.747 4.736 4.724 4.720 4.714 4.708 4.702 4.678 4.672 4.665 4.659 2.980 2.975 2.964 2.960 2.957 2.948 2.943 2.928 2.924 2.691 2.685 2.675 2.668 2.508 2.501 2.495 2.490 2.483 2.426 2.324 2.321 2.315 2.312 2.306 2.304 2.290 2.287 2.195 2.189 2.183 2.177 2.157 2.139 1.839 1.835 1.831 1.822 1.818 1.814 1.766 1.749 1.731 1.715 1.713 1.682 1.638 1.632 1.625 1.621 1.612 1.603 1.596 1.594 1.589 1.583 1.576 1.566 1.561 1.554 1.550 1.540 1.537 1.533 1.528 1.522 1.518 1.512 1.508 1.501 1.490
CN
TsHN
4r
Chemical Formula: C18H24N2O2S
1H NMR, 400 MHz, CDCl3
1.81 1.82 0.74 1.00 0.88 1.94 0.93 0.86 2.74 1.98 0.93 0.82 0.93 4.93
10.5 10.0 9.5 9.0 8.5 8.0 7.5 7.0 6.5 6.0 5.5 5.0 4.5 4.0 3.5 3.0 2.5 2.0 1.5 1.0 0.5 0.0 -0.5
f1 (ppm)

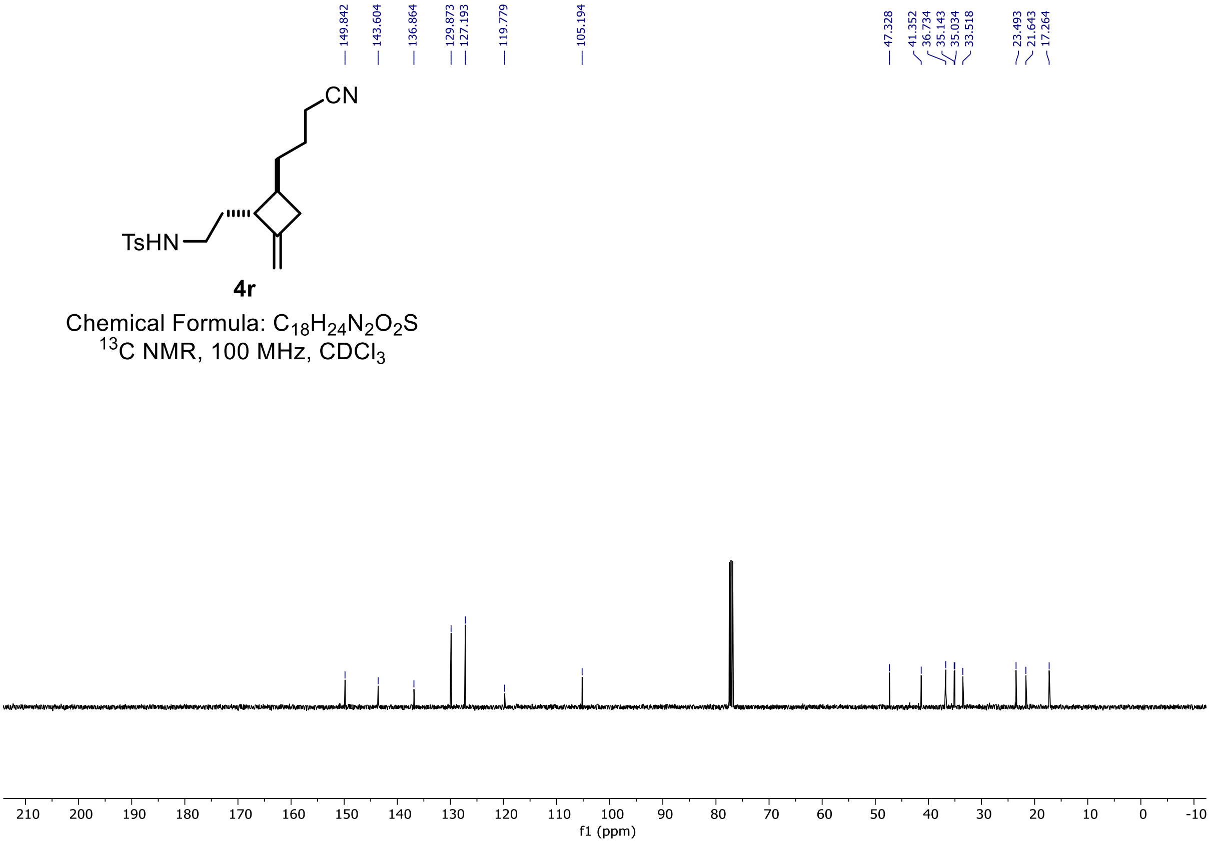
149.842 143.604 136.864 129.873 127.193 119.779 105.194 47.328 41.352 36.734 35.143 35.034 33.518 23.493 21.643 17.264
CN
TsHN
4r
Chemical Formula: C18H24N2O2S
13C NMR, 100 MHz, CDCl3
210 200 190 180 170 160 150 140 130 120 110 100 90 80 70 60 50 40 30 20 10 0 -10
f1 (ppm)

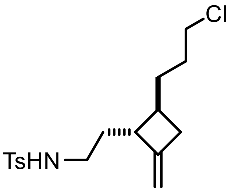

4s

Chemical Formula: $C_{17}H_{24}ClNO_2S$

^1H NMR, 400 MHz, $CDCl_3$

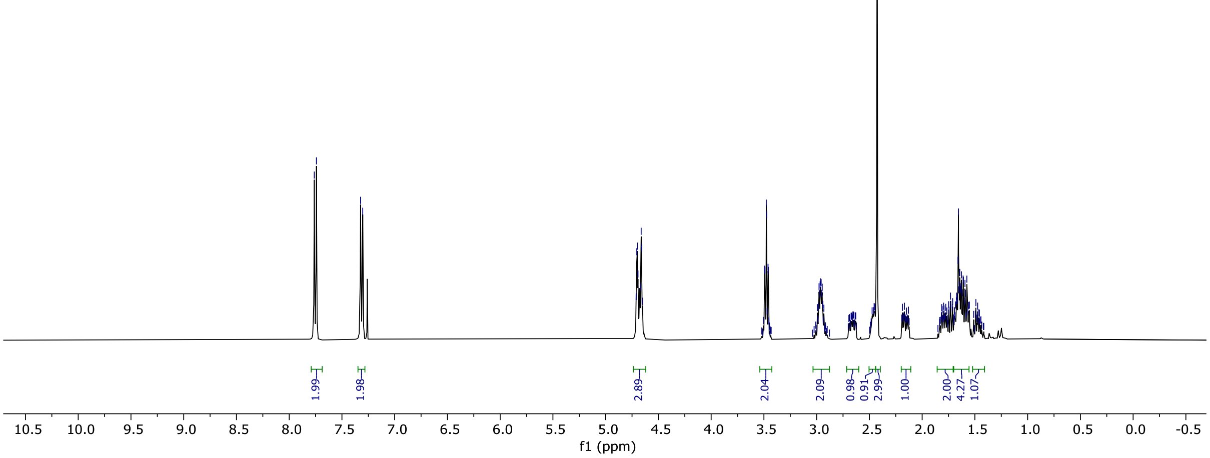

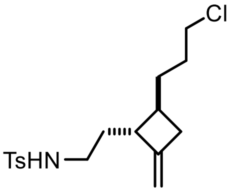

4s

Chemical Formula: $C_{17}H_{24}ClNO_2S$

^{13}C NMR, 100 MHz, $CDCl_3$

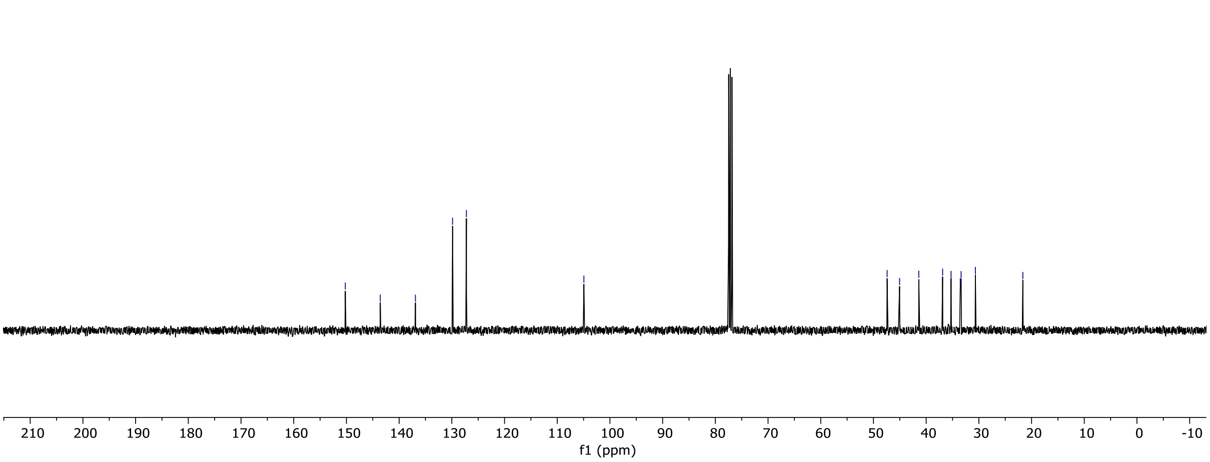

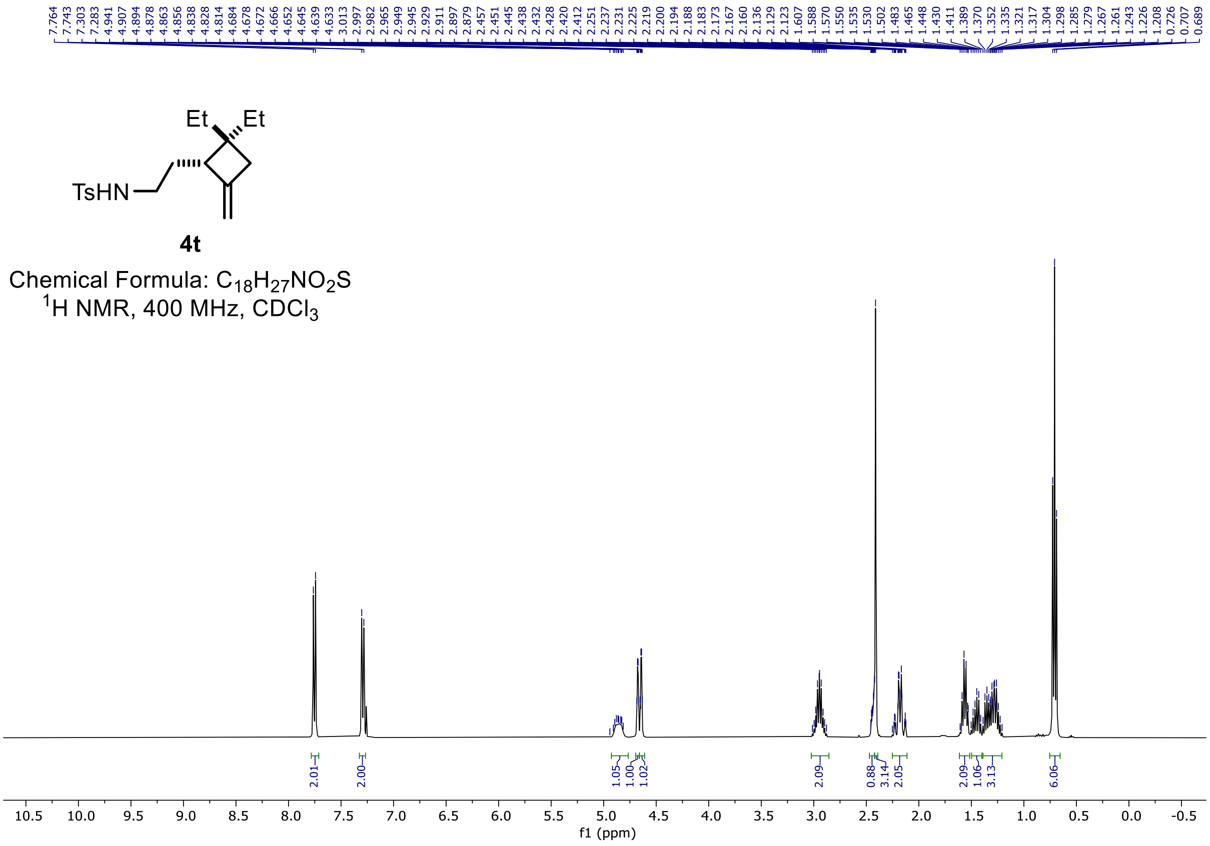

4t

Chemical Formula: $C_{18}H_{27}NO_2S$

^1H NMR, 400 MHz, $CDCl_3$

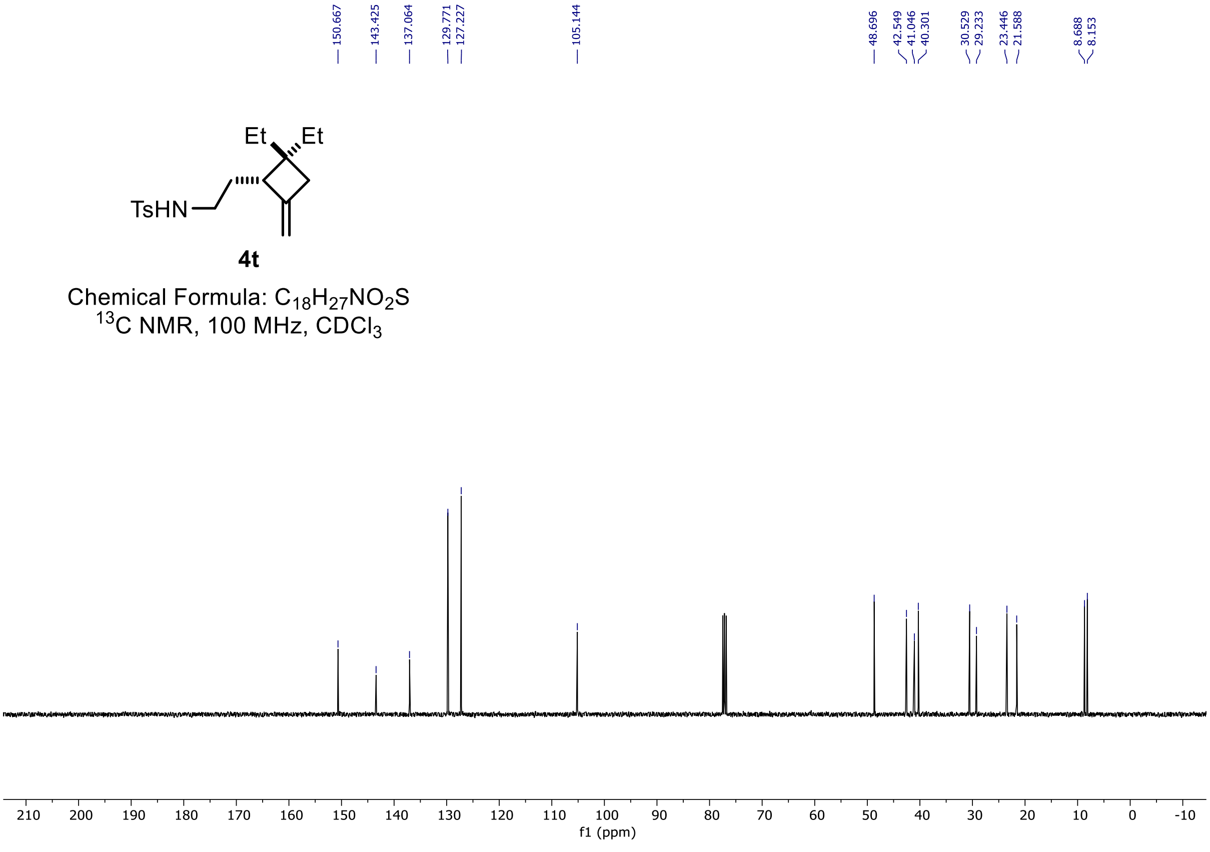

4t

Chemical Formula: $C_{18}H_{27}NO_2S$

^{13}C NMR, 100 MHz, $CDCl_3$

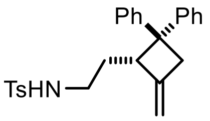

4u

Chemical Formula: $C_{26}H_{27}NO_2S$

^{1}H NMR, 400 MHz, $CDCl_3$

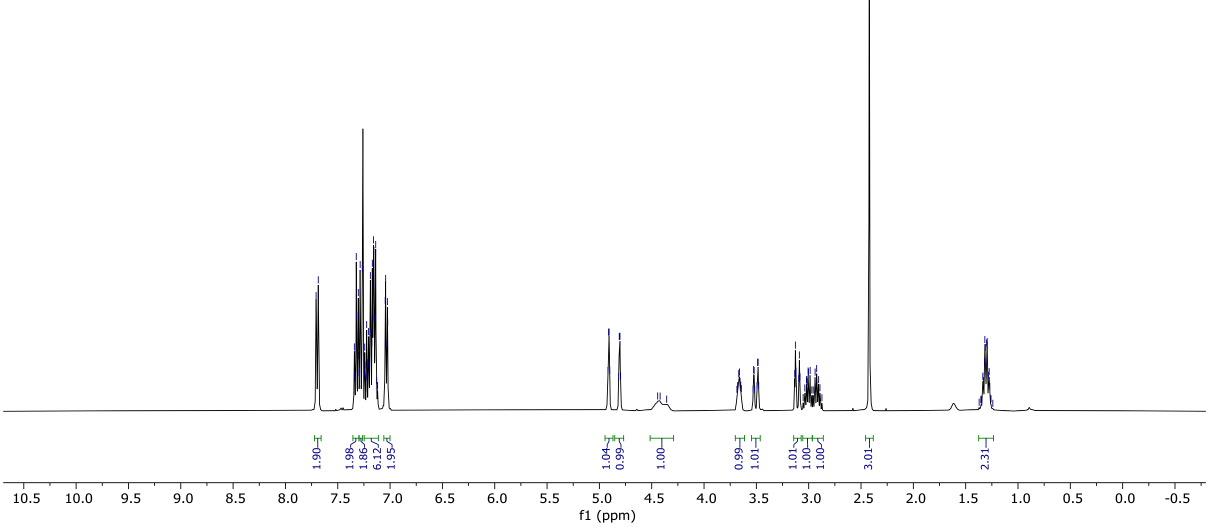

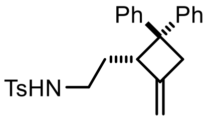

4u

Chemical Formula: $C_{26}H_{27}NO_2S$

^{13}C NMR, 100 MHz, $CDCl_3$

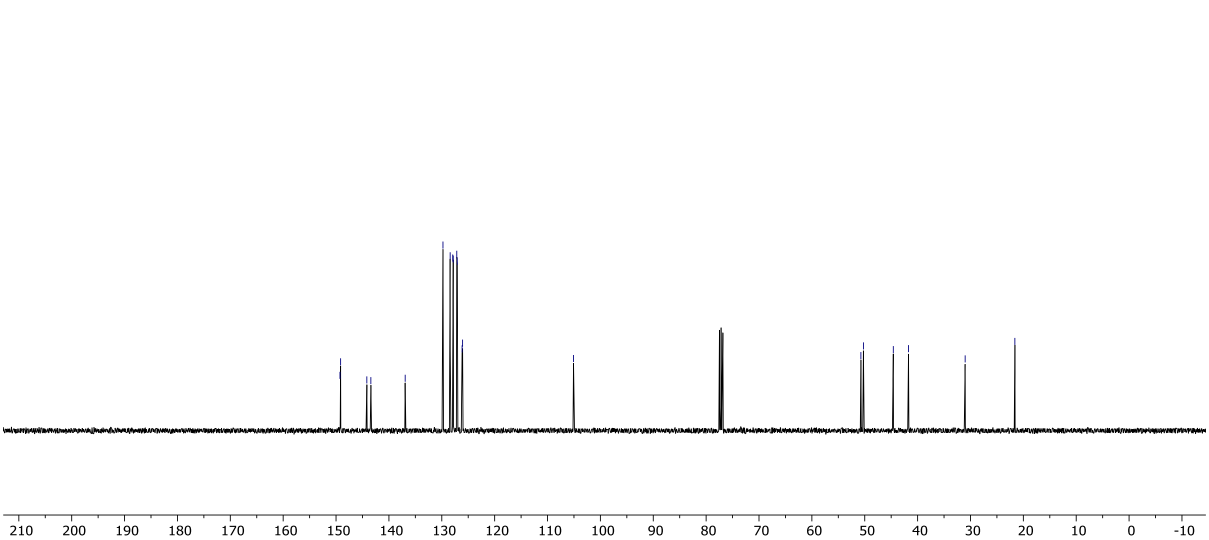

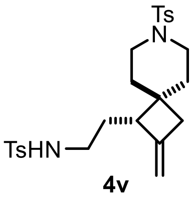

4v

Chemical Formula: $C_{25}H_{32}N_2O_4S_2$

^{1}H NMR, 400 MHz, $CDCl_3$

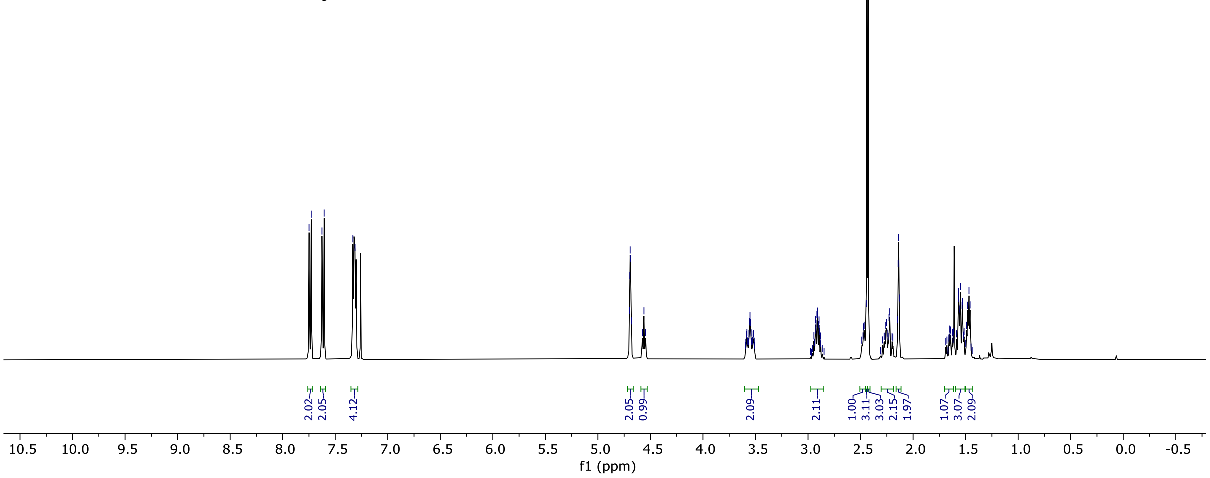

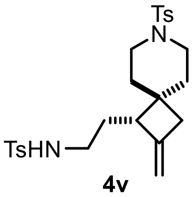

4v

Chemical Formula: $C_{25}H_{32}N_2O_4S_2$

^{13}C NMR, 100 MHz, $CDCl_3$

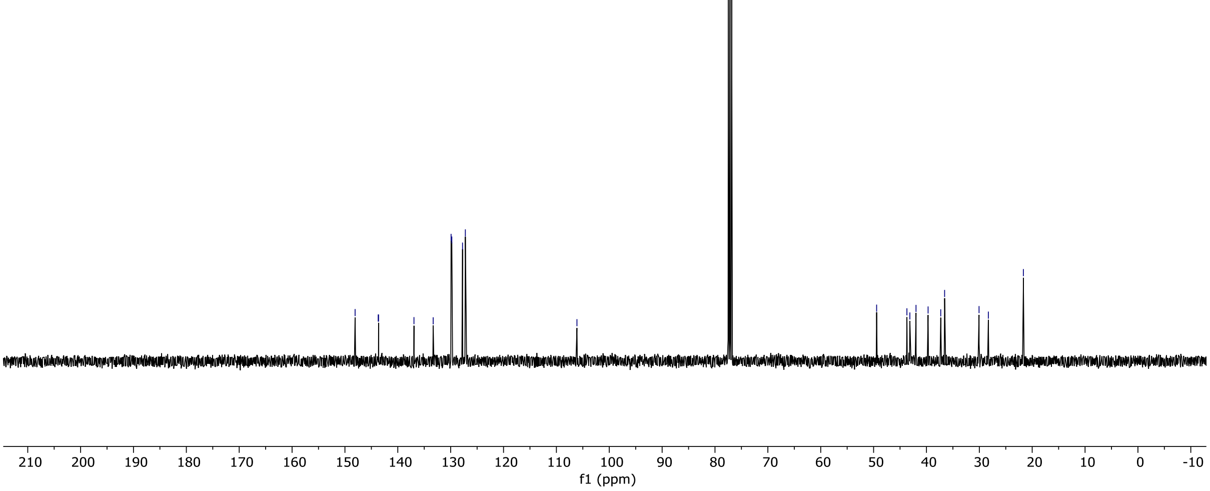

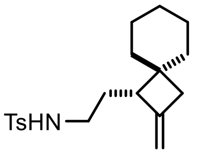

4w

Chemical Formula: $C_{19}H_{27}NO_2S$

^{1}H NMR, 400 MHz, $CDCl_3$

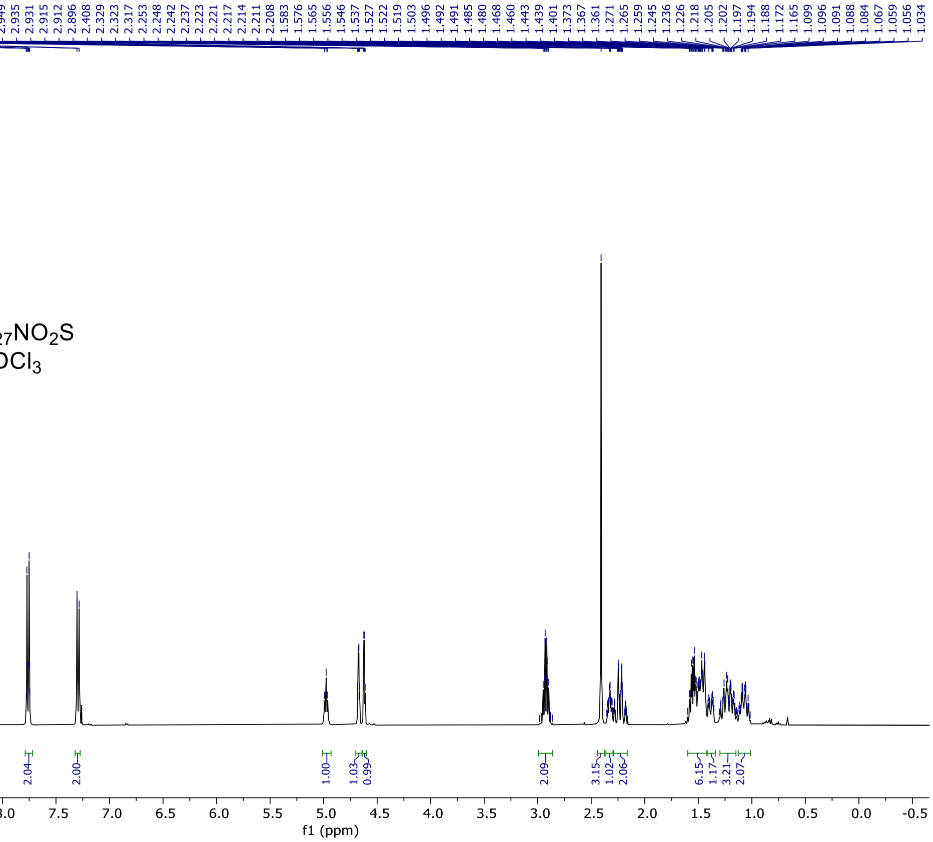

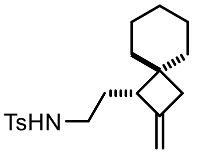

4w

Chemical Formula: $C_{19}H_{27}NO_2S$

^{13}C NMR, 100 MHz, $CDCl_3$

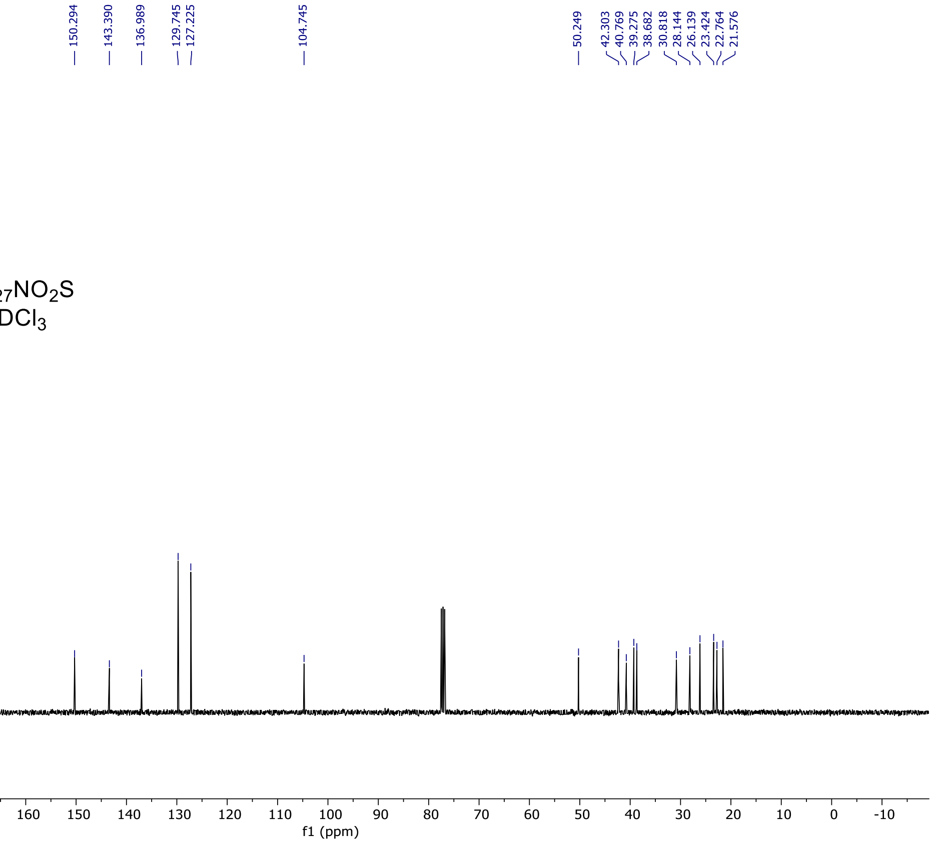

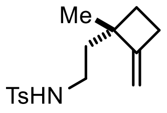

4x

Chemical Formula: $C_{15}H_{21}NO_2S$

^1H NMR, 400 MHz, $CDCl_3$

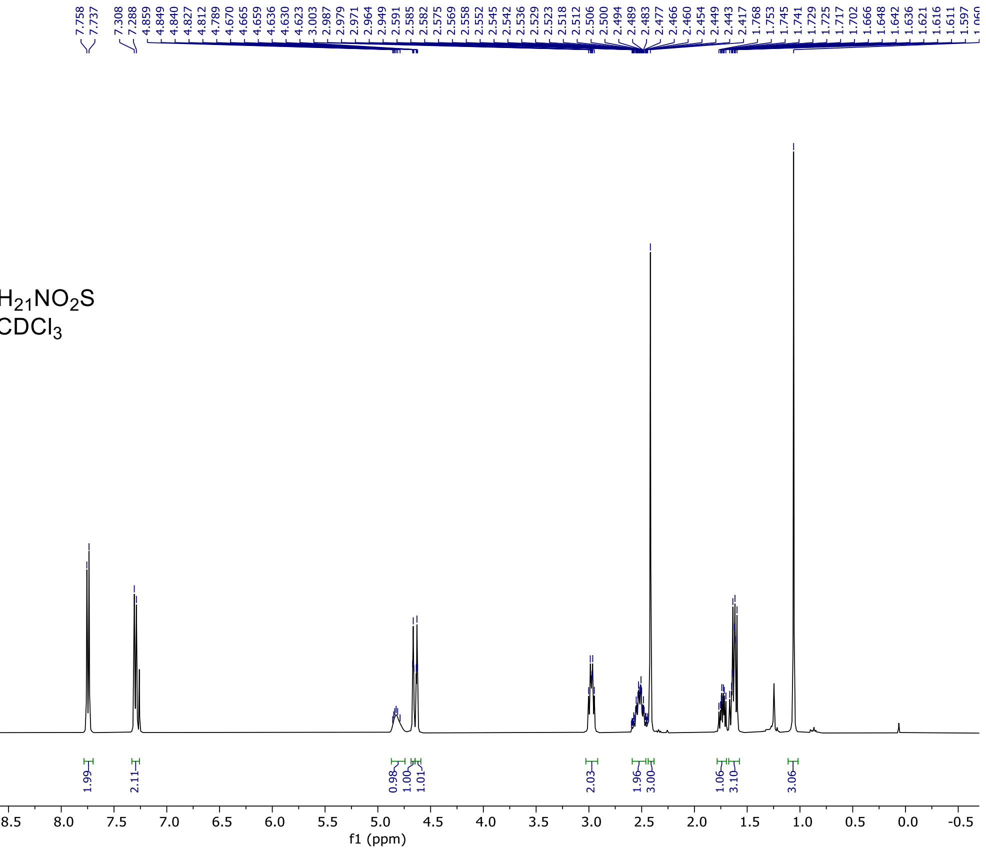

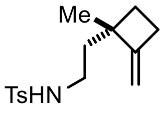

4x

Chemical Formula: $C_{15}H_{21}NO_2S$

^{13}C NMR, 100 MHz, $CDCl_3$

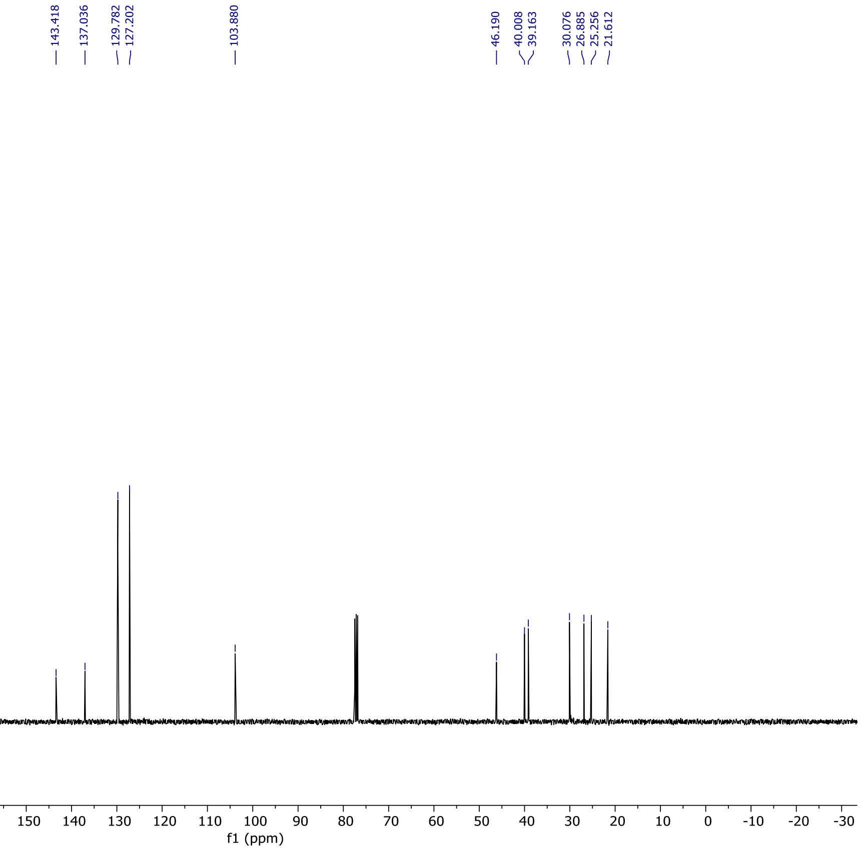

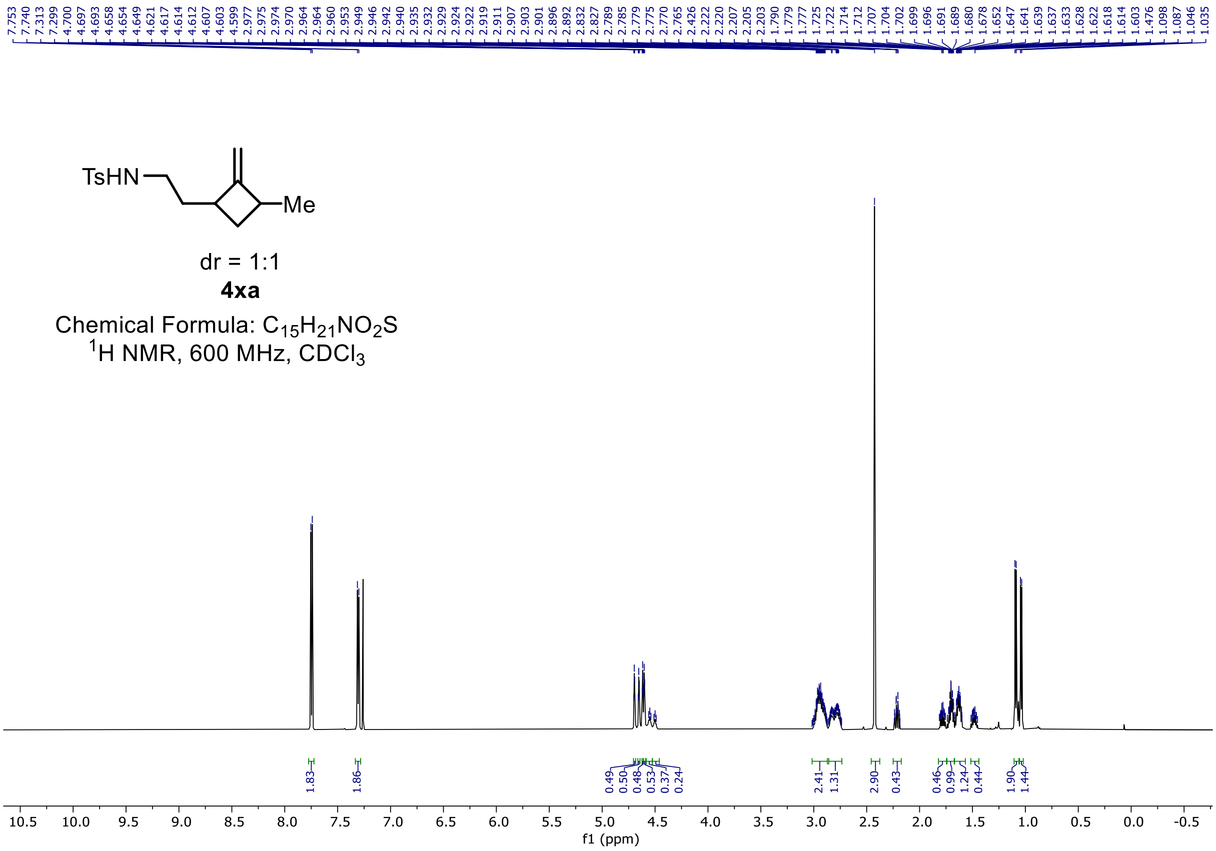
7.753 7.740 7.313 7.299 4.700 4.697 4.693 4.658 4.654 4.649 4.621 4.617 4.614 4.612 4.607 4.603 4.599 2.977 2.975 2.974 2.970 2.964 2.964 2.960 2.953 2.949 2.946 2.942 2.940 2.935 2.932 2.929 2.924 2.922 2.919 2.911 2.907 2.903 2.901 2.896 2.892 2.832 2.827 2.789 2.785 2.779 2.775 2.770 2.765 2.426 2.222 2.220 2.207 2.205 2.203 1.790 1.779 1.777 1.725 1.722 1.714 1.712 1.707 1.704 1.702 1.699 1.696 1.691 1.689 1.680 1.678 1.652 1.647 1.641 1.639 1.637 1.633 1.628 1.622 1.618 1.614 1.603 1.476 1.098 1.087 1.046 1.035
TsHN
Me
dr = 1:1
4xa
Chemical Formula: $C_{15}H_{21}NO_2S$
^{1}H NMR, 600 MHz, $CDCl_3$
1.83
1.86
0.49 0.50 0.48 0.53 0.37 0.24
2.41 1.31
2.90
0.43
0.46 0.99 1.24 0.44
1.90 1.44
10.5 10.0 9.5 9.0 8.5 8.0 7.5 7.0 6.5 6.0 5.5 5.0 4.5 4.0 3.5 3.0 2.5 2.0 1.5 1.0 0.5 0.0 -0.5
f1 (ppm)

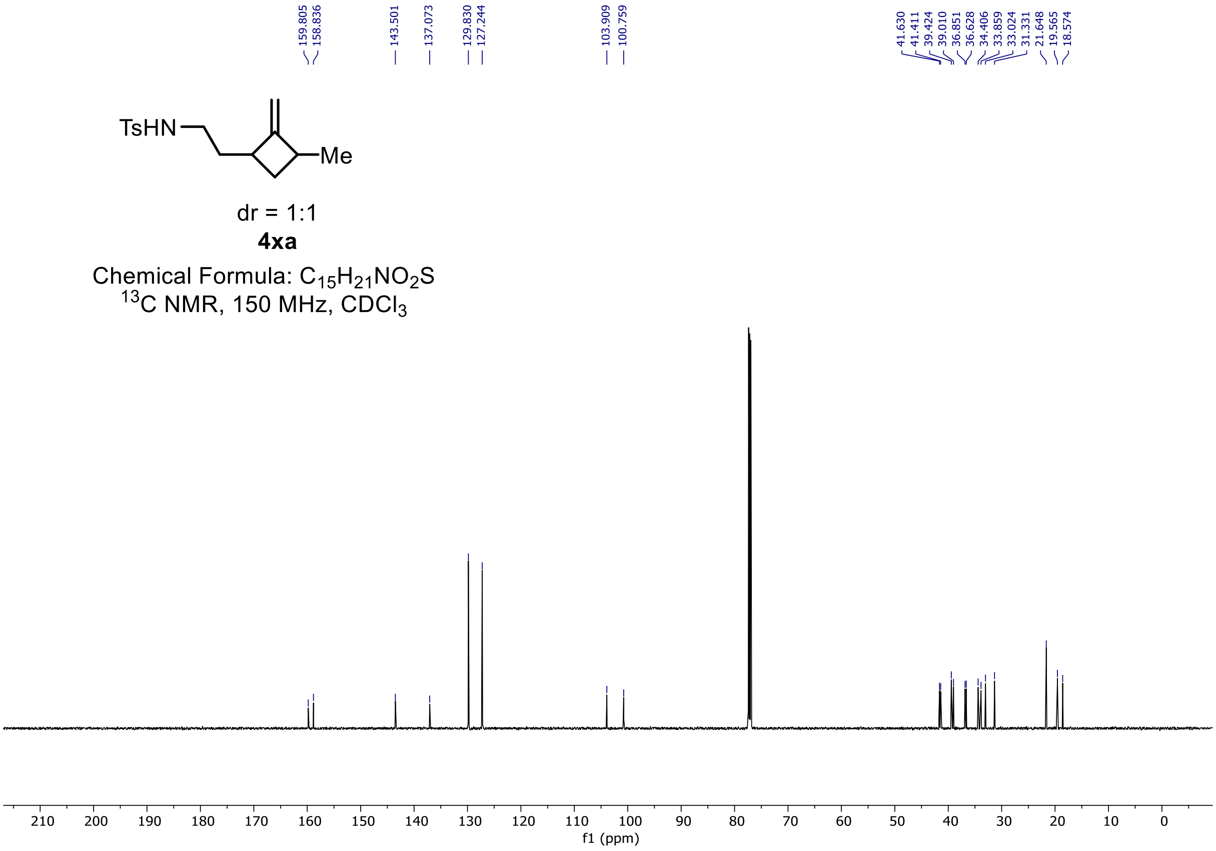
159.805 158.836 143.501 137.073 129.830 127.244 103.909 100.759 41.630 41.411 39.424 39.010 36.851 36.628 34.406 33.859 33.024 31.331 21.648 19.565 18.574
TsHN
Me
dr = 1:1
4xa
Chemical Formula: $C_{15}H_{21}NO_2S$
^{13}C NMR, 150 MHz, $CDCl_3$
210 200 190 180 170 160 150 140 130 120 110 100 90 80 70 60 50 40 30 20 10 0
f1 (ppm)

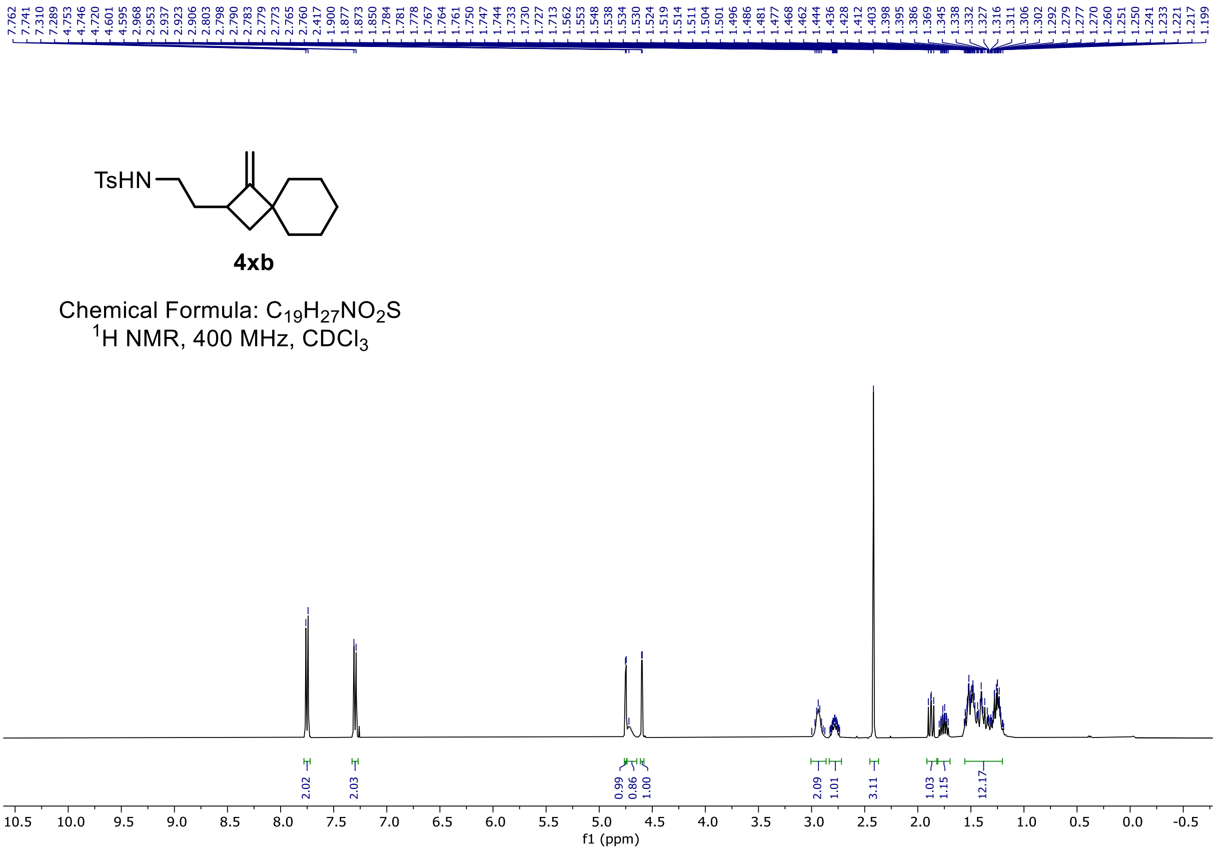

4xb

Chemical Formula: $C_{19}H_{27}NO_2S$

^{1}H NMR, 400 MHz, $CDCl_3$

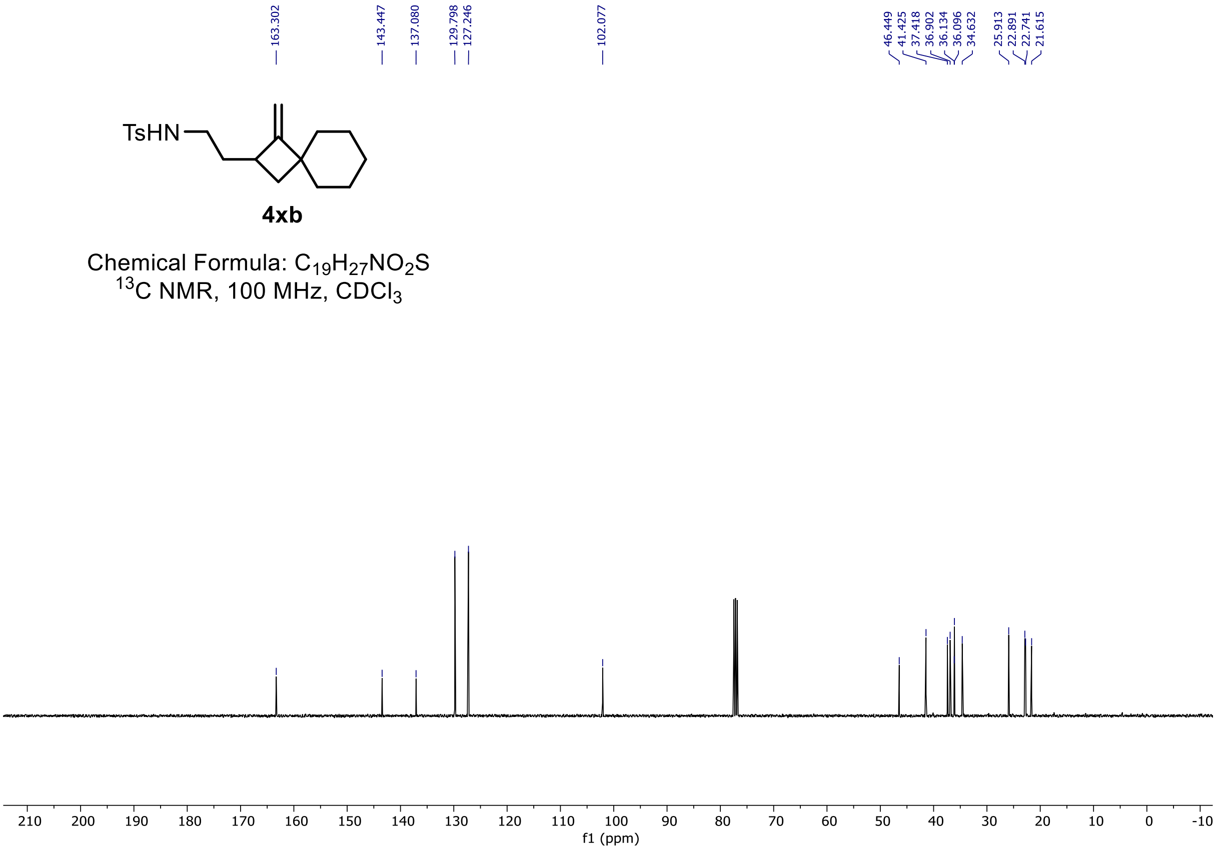

4xb

Chemical Formula: $C_{19}H_{27}NO_2S$

^{13}C NMR, 100 MHz, $CDCl_3$

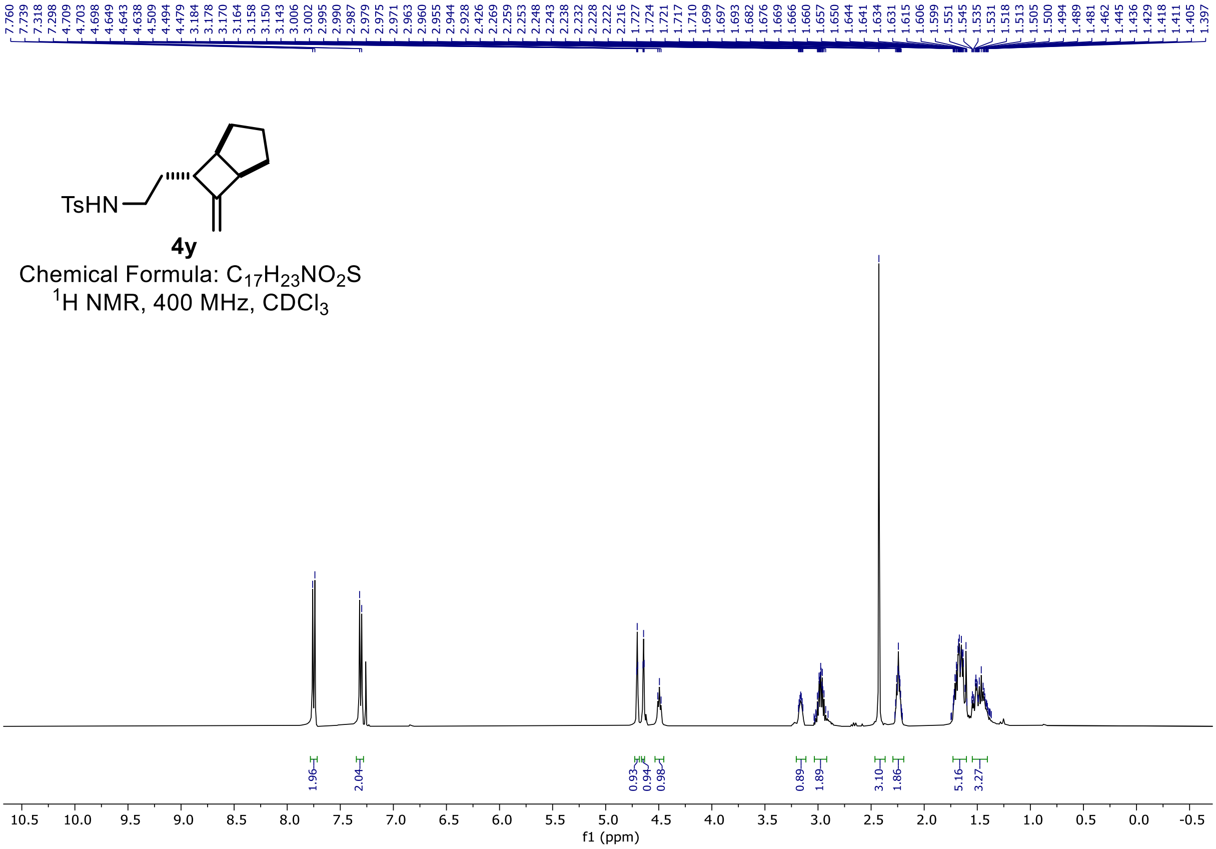
7.760 7.739 7.318 7.298 4.709 4.703 4.698 4.649 4.643 4.638 4.509 4.494 4.479 3.184 3.178 3.170 3.164 3.158 3.150 3.143 3.006 3.002 2.995 2.990 2.987 2.979 2.975 2.971 2.963 2.960 2.955 2.944 2.928 2.426 2.269 2.259 2.253 2.248 2.243 2.238 2.232 2.228 2.222 2.216 1.727 1.724 1.721 1.717 1.710 1.699 1.697 1.693 1.682 1.676 1.669 1.666 1.660 1.657 1.650 1.644 1.641 1.634 1.631 1.615 1.606 1.599 1.551 1.545 1.535 1.531 1.518 1.513 1.505 1.500 1.494 1.489 1.481 1.462 1.445 1.436 1.429 1.418 1.411 1.405 1.397
TsHN
4y
Chemical Formula: $C_{17}H_{23}NO_2S$
^1H NMR, 400 MHz, $CDCl_3$
1.96 2.04 0.93 0.94 0.98 0.89 1.89 3.10 1.86 5.16 3.27
10.5 10.0 9.5 9.0 8.5 8.0 7.5 7.0 6.5 6.0 5.5 5.0 4.5 4.0 3.5 3.0 2.5 2.0 1.5 1.0 0.5 0.0 -0.5
f1 (ppm)

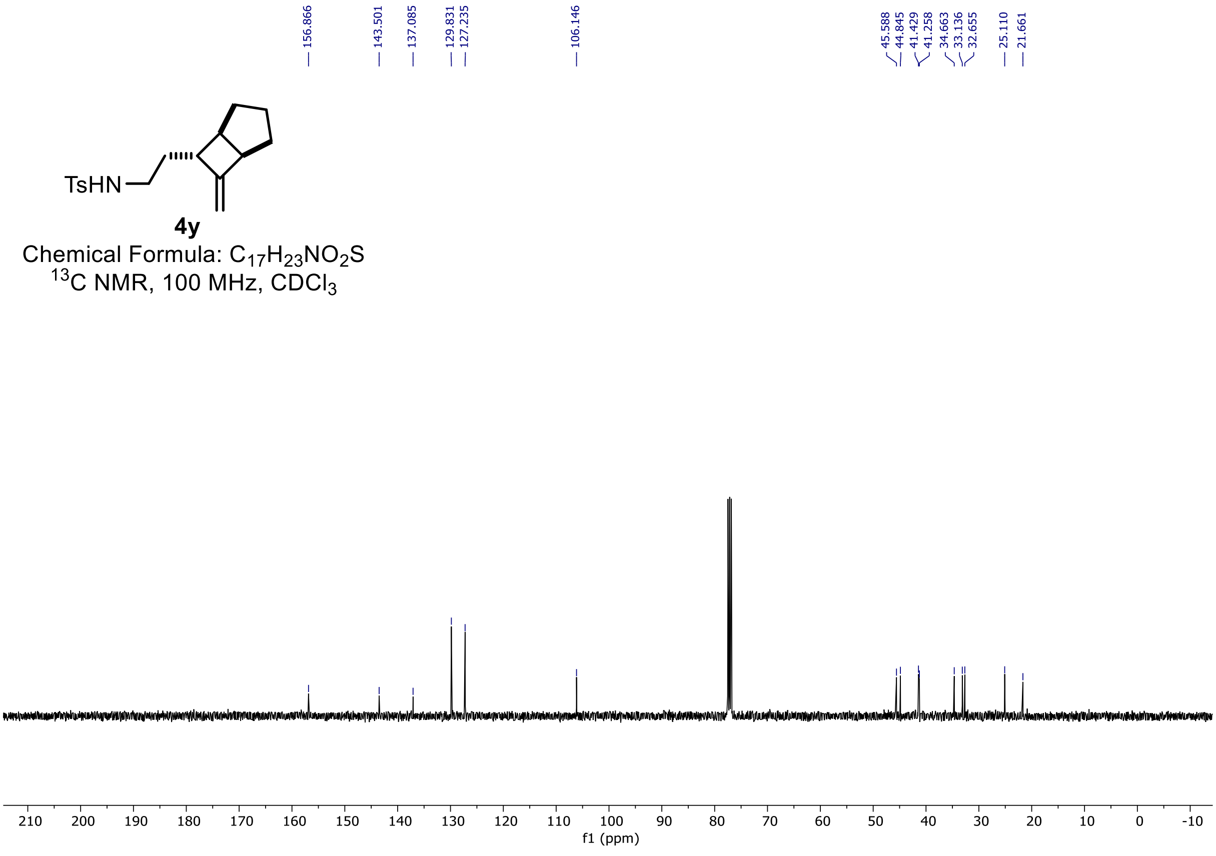
156.866 143.501 137.085 129.831 127.235 106.146 45.588 44.845 41.429 41.258 34.663 33.136 32.655 25.110 21.661
TsHN
4y
Chemical Formula: $C_{17}H_{23}NO_2S$
^{13}C NMR, 100 MHz, $CDCl_3$
210 200 190 180 170 160 150 140 130 120 110 100 90 80 70 60 50 40 30 20 10 0 -10
f1 (ppm)

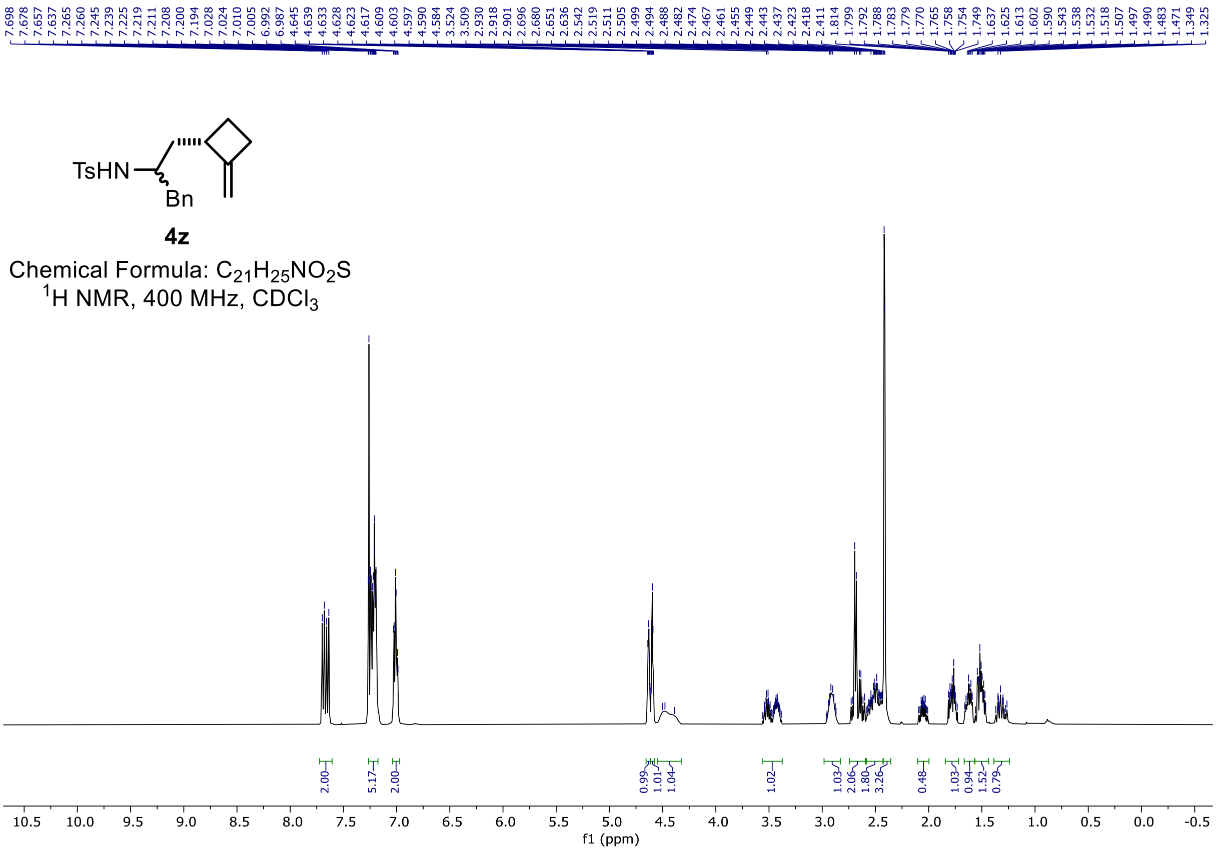
7.698 7.678 7.657 7.637 7.265 7.260 7.245 7.239 7.225 7.219 7.211 7.208 7.200 7.194 7.028 7.024 7.010 7.005 6.992 6.987 4.645 4.639 4.633 4.628 4.623 4.617 4.609 4.603 4.597 4.590 4.584 3.524 3.509 2.930 2.918 2.901 2.696 2.680 2.651 2.636 2.542 2.519 2.511 2.505 2.499 2.494 2.488 2.482 2.474 2.467 2.461 2.455 2.449 2.443 2.437 2.423 2.418 2.411 1.814 1.799 1.792 1.788 1.783 1.779 1.770 1.765 1.758 1.754 1.749 1.637 1.625 1.613 1.602 1.590 1.543 1.538 1.532 1.518 1.507 1.497 1.490 1.483 1.471 1.349 1.325
TsHN
Bn
4z
Chemical Formula: C21H25NO2S
1H NMR, 400 MHz, CDCl3
2.00 5.17 2.00 0.99 1.01 1.04 1.02 1.03 2.06 1.80 3.26 0.48 1.03 0.94 1.52 0.79
10.5 10.0 9.5 9.0 8.5 8.0 7.5 7.0 6.5 6.0 5.5 5.0 4.5 4.0 3.5 3.0 2.5 2.0 1.5 1.0 0.5 0.0 -0.5
f1 (ppm)

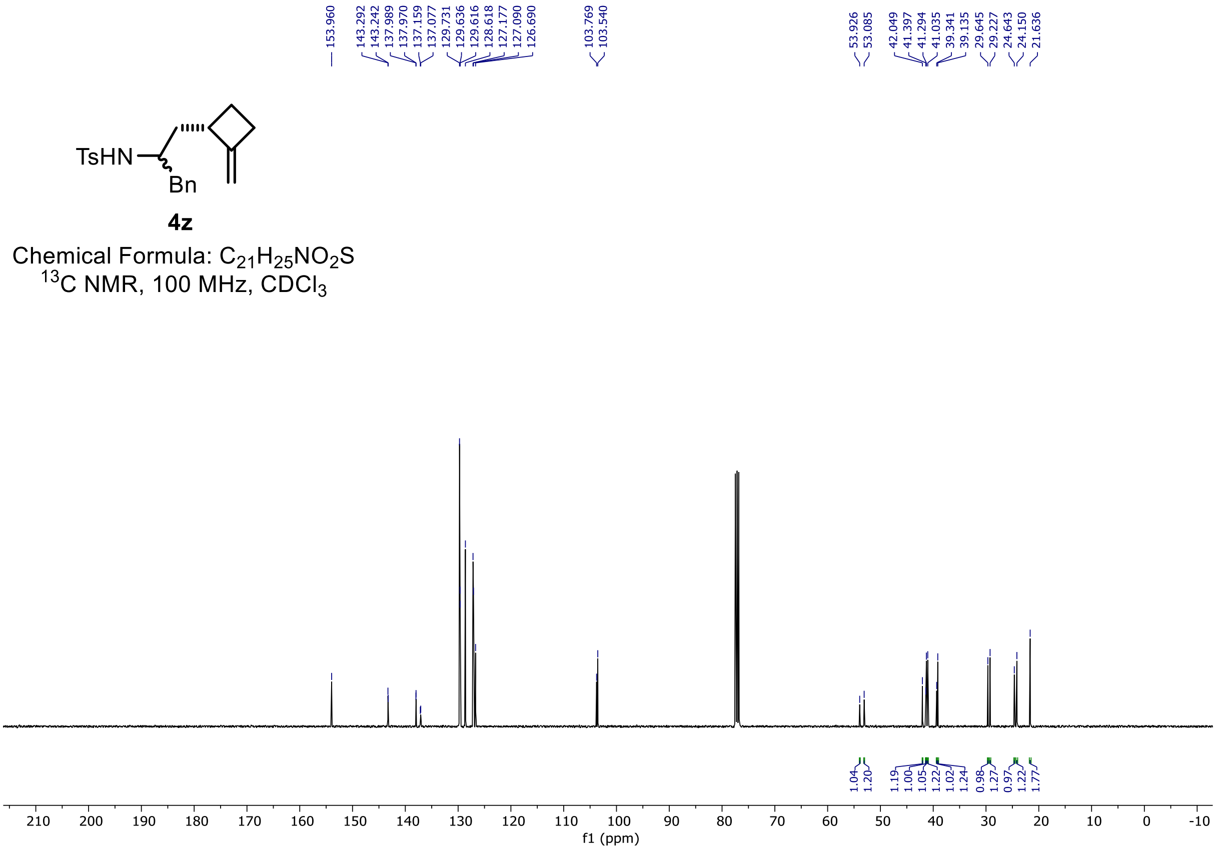
153.960 143.292 143.242 137.989 137.970 137.159 137.077 129.731 129.636 129.616 128.618 127.177 127.090 126.690 103.769 103.540 53.926 53.085 42.049 41.397 41.294 41.035 39.341 39.135 29.645 29.227 24.643 24.150 21.636
TsHN
Bn
4z
Chemical Formula: C21H25NO2S
13C NMR, 100 MHz, CDCl3
1.04 1.20 1.19 1.00 1.05 1.22 1.02 1.24 0.98 1.27 0.97 1.22 1.77
210 200 190 180 170 160 150 140 130 120 110 100 90 80 70 60 50 40 30 20 10 0 -10
f1 (ppm)

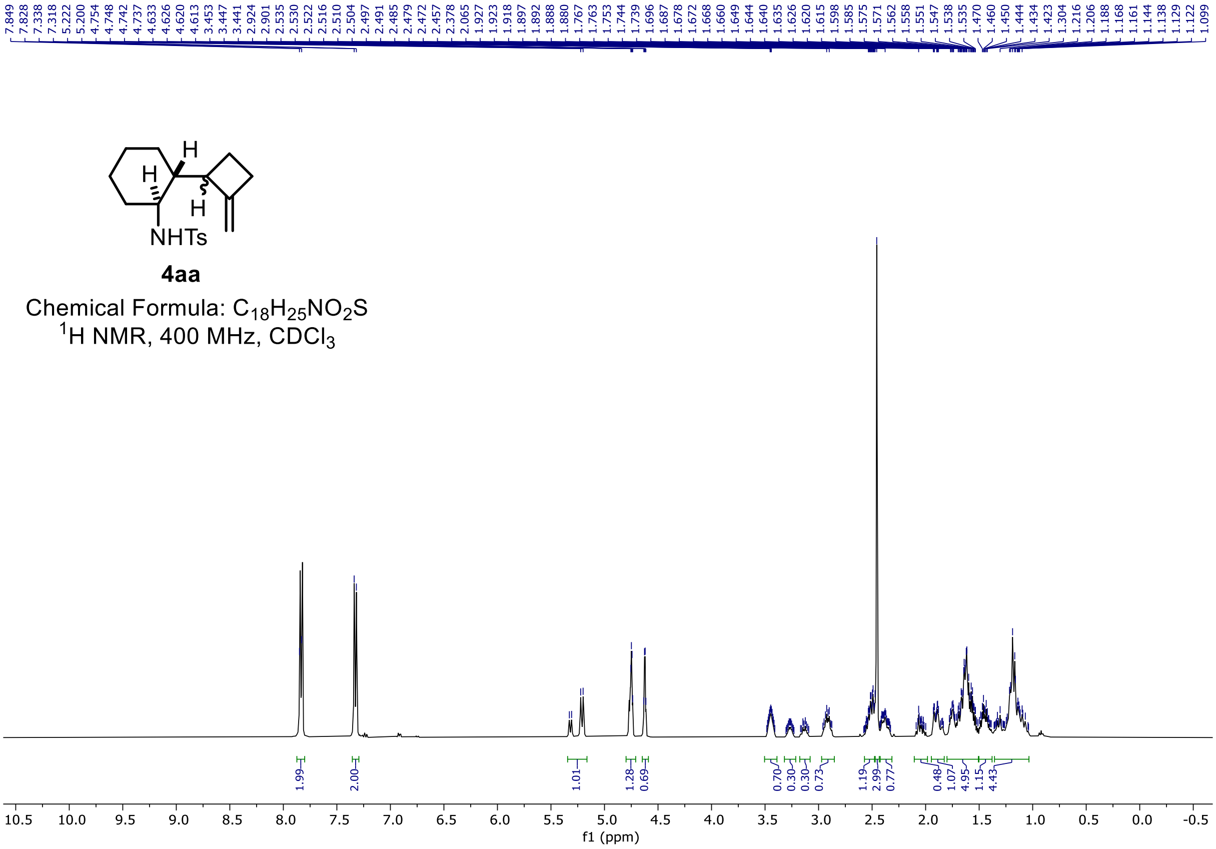
H
H
H
NHTs
4aa
Chemical Formula: $C_{18}H_{25}NO_2S$
^{1}H NMR, 400 MHz, $CDCl_3$
f1 (ppm)

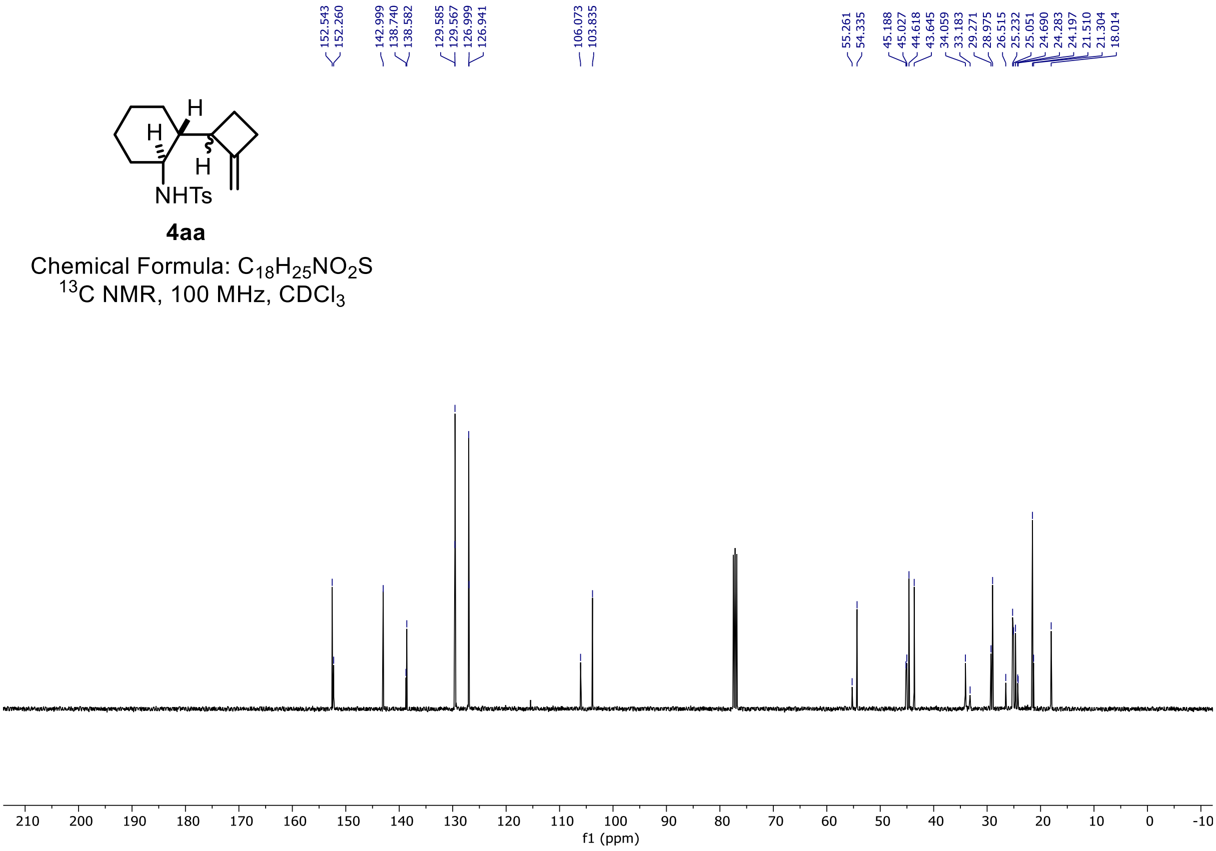
H
H
H
NHTs
4aa
Chemical Formula: $C_{18}H_{25}NO_2S$
^{13}C NMR, 100 MHz, $CDCl_3$
f1 (ppm)

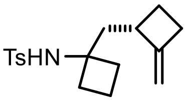

4ab
Chemical Formula: $C_{17}H_{23}NO_2S$
^1H NMR, 400 MHz, $CDCl_3$

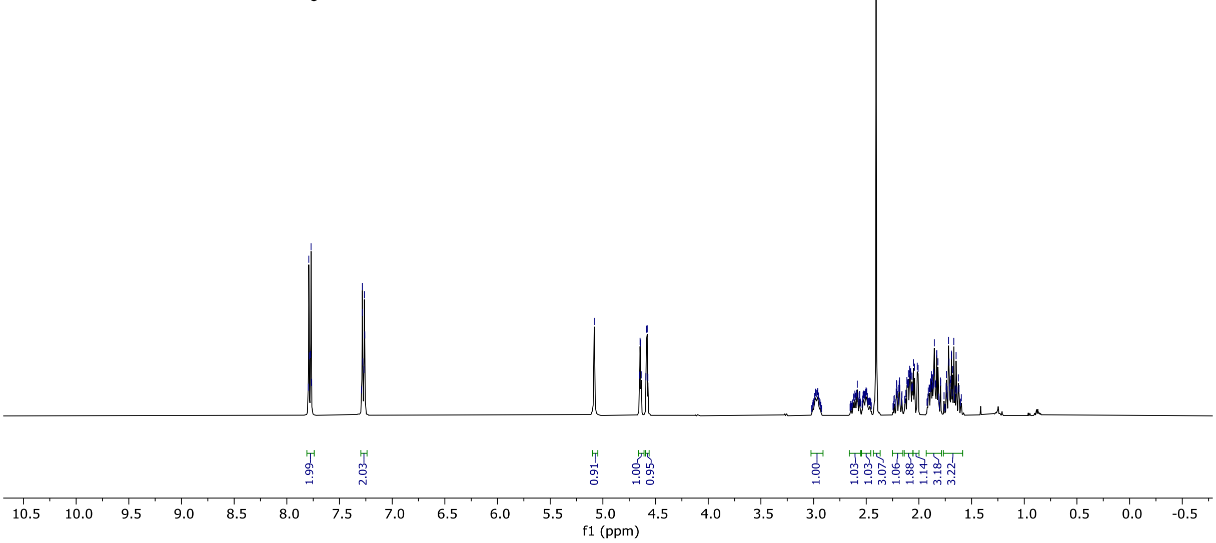

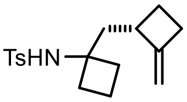

4ab
Chemical Formula: $C_{17}H_{23}NO_2S$
^{13}C NMR, 100 MHz, $CDCl_3$

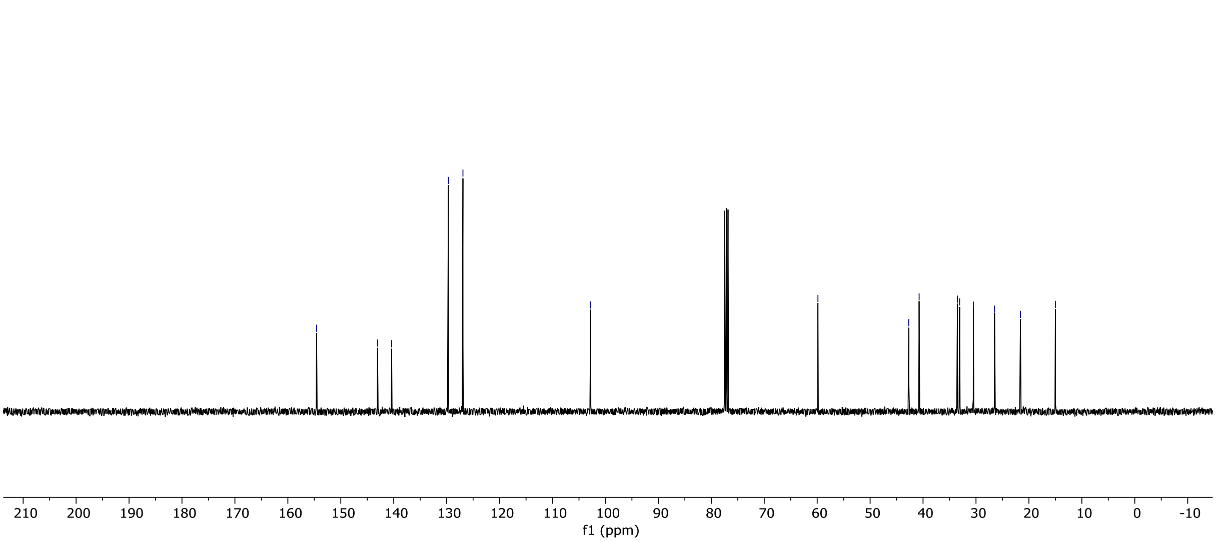

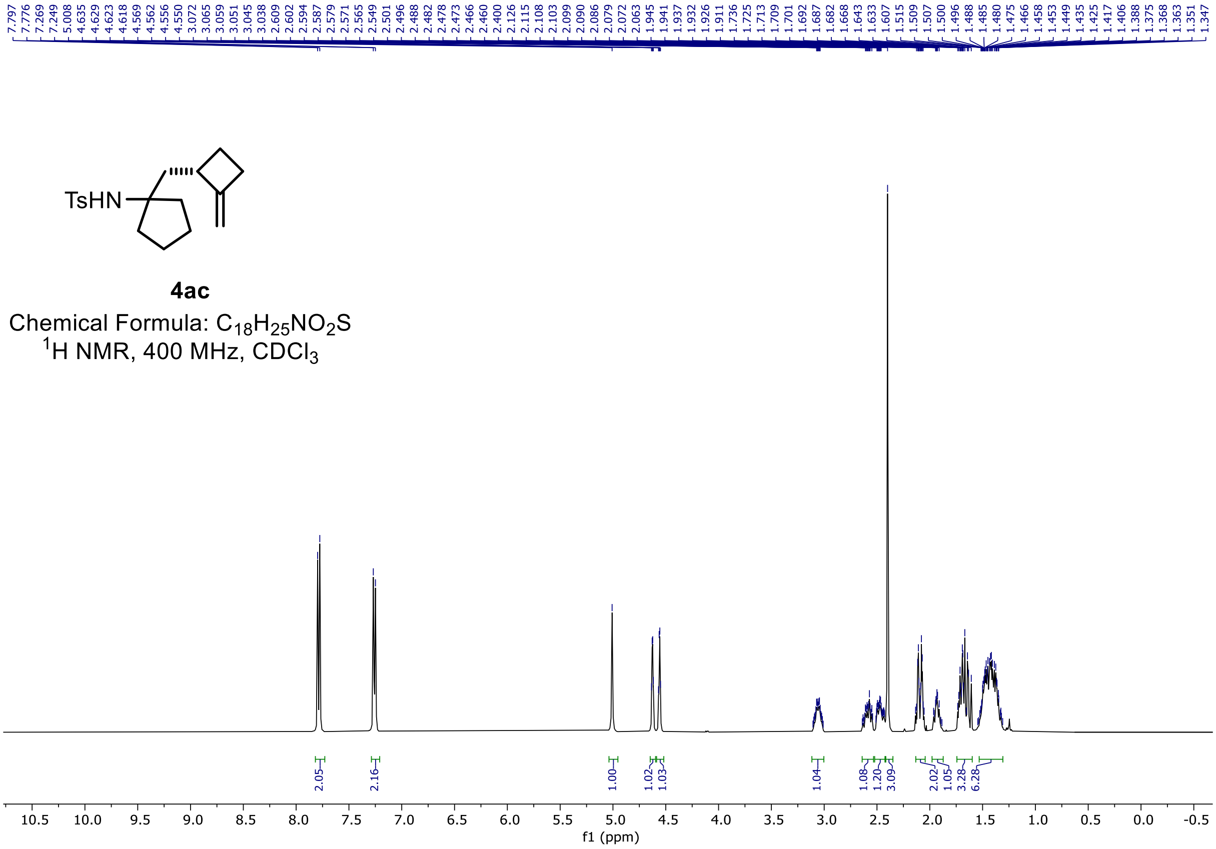
7.797 7.776 7.269 7.249 5.008 4.635 4.629 4.623 4.618 4.569 4.562 4.556 4.550 3.072 3.065 3.059 3.051 3.045 3.038 2.609 2.602 2.594 2.587 2.579 2.571 2.565 2.549 2.501 2.496 2.488 2.482 2.478 2.473 2.466 2.460 2.400 2.126 2.115 2.108 2.103 2.099 2.090 2.086 2.079 2.072 2.063 1.945 1.941 1.937 1.932 1.926 1.911 1.736 1.725 1.713 1.709 1.701 1.692 1.687 1.682 1.668 1.643 1.633 1.607 1.515 1.509 1.507 1.500 1.496 1.488 1.485 1.480 1.475 1.466 1.458 1.453 1.449 1.435 1.425 1.417 1.406 1.388 1.375 1.368 1.363 1.351 1.347
TsHN
4ac
Chemical Formula: $C_{18}H_{25}NO_2S$
^{1}H NMR, 400 MHz, $CDCl_3$
2.05 2.16 1.00 1.02 1.03 1.04 1.08 1.20 3.09 2.02 1.05 3.28 6.28
10.5 10.0 9.5 9.0 8.5 8.0 7.5 7.0 6.5 6.0 5.5 5.0 4.5 4.0 3.5 3.0 2.5 2.0 1.5 1.0 0.5 0.0 -0.5
f1 (ppm)

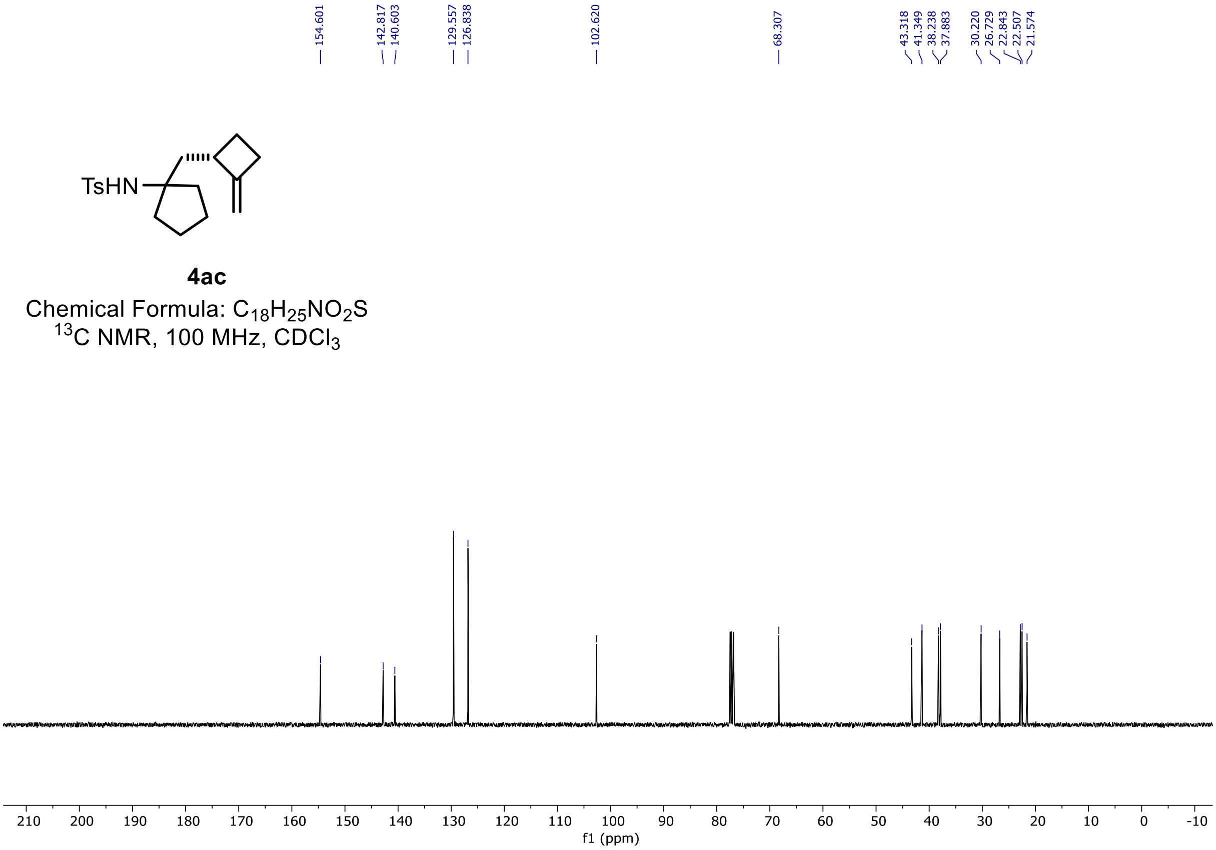
154.601 142.817 140.603 129.557 126.838 102.620 68.307 43.318 41.349 38.238 37.883 30.220 26.729 22.843 22.507 21.574
TsHN
4ac
Chemical Formula: $C_{18}H_{25}NO_2S$
^{13}C NMR, 100 MHz, $CDCl_3$
210 200 190 180 170 160 150 140 130 120 110 100 90 80 70 60 50 40 30 20 10 0 -10
f1 (ppm)

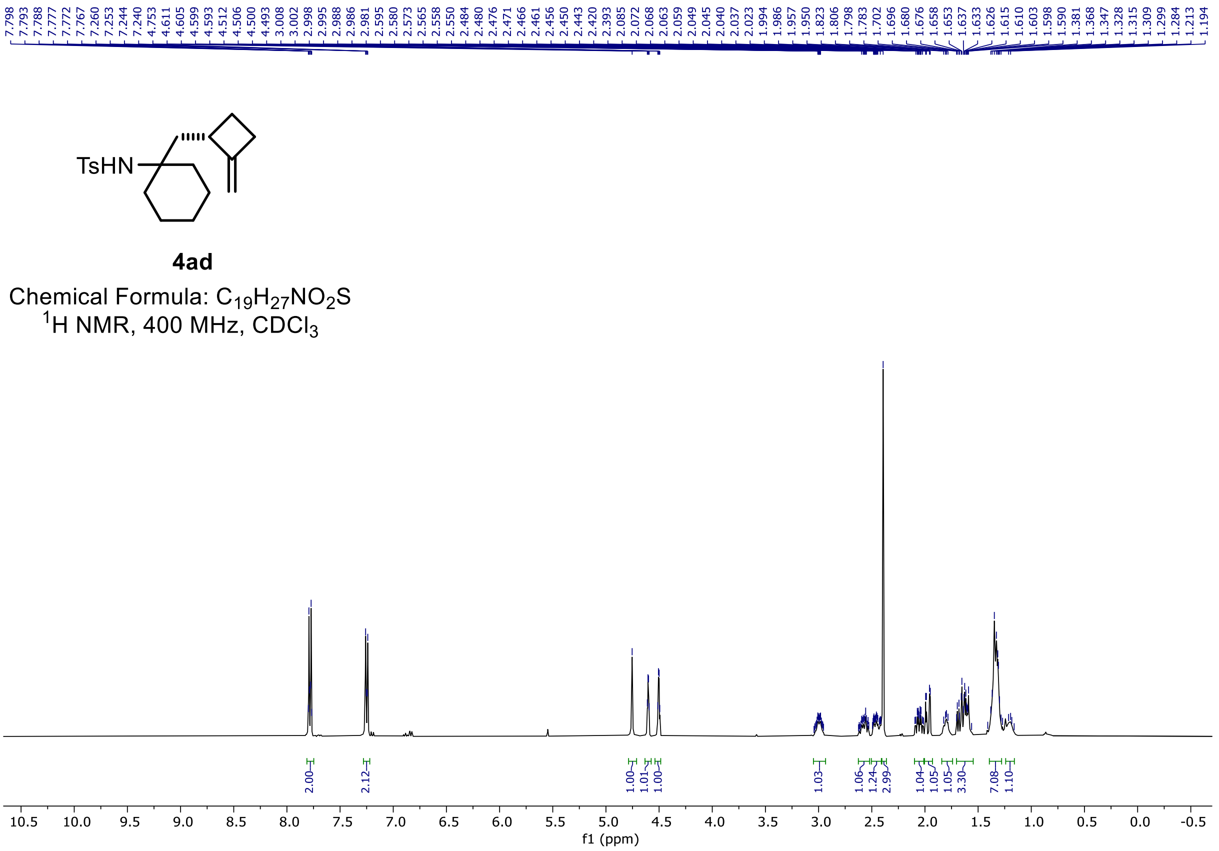
7.798 7.793 7.788 7.777 7.772 7.767 7.260 7.253 7.244 7.240 4.753 4.611 4.605 4.599 4.593 4.512 4.506 4.500 4.493 3.008 3.002 2.998 2.995 2.988 2.986 2.981 2.595 2.580 2.573 2.565 2.558 2.550 2.484 2.480 2.476 2.471 2.466 2.461 2.456 2.450 2.443 2.420 2.393 2.085 2.072 2.068 2.063 2.059 2.049 2.045 2.040 2.037 2.023 1.994 1.986 1.957 1.950 1.823 1.806 1.798 1.783 1.702 1.696 1.680 1.676 1.658 1.653 1.637 1.633 1.626 1.615 1.610 1.603 1.598 1.590 1.381 1.368 1.347 1.328 1.315 1.309 1.299 1.284 1.213 1.194
TsHN
4ad
Chemical Formula: C19H27NO2S
1H NMR, 400 MHz, CDCl3
2.00 2.12 1.00 1.01 1.00 1.03 1.06 1.24 2.99 1.04 1.05 1.05 3.30 7.08 1.10
10.5 10.0 9.5 9.0 8.5 8.0 7.5 7.0 6.5 6.0 5.5 5.0 4.5 4.0 3.5 3.0 2.5 2.0 1.5 1.0 0.5 0.0 -0.5
f1 (ppm)

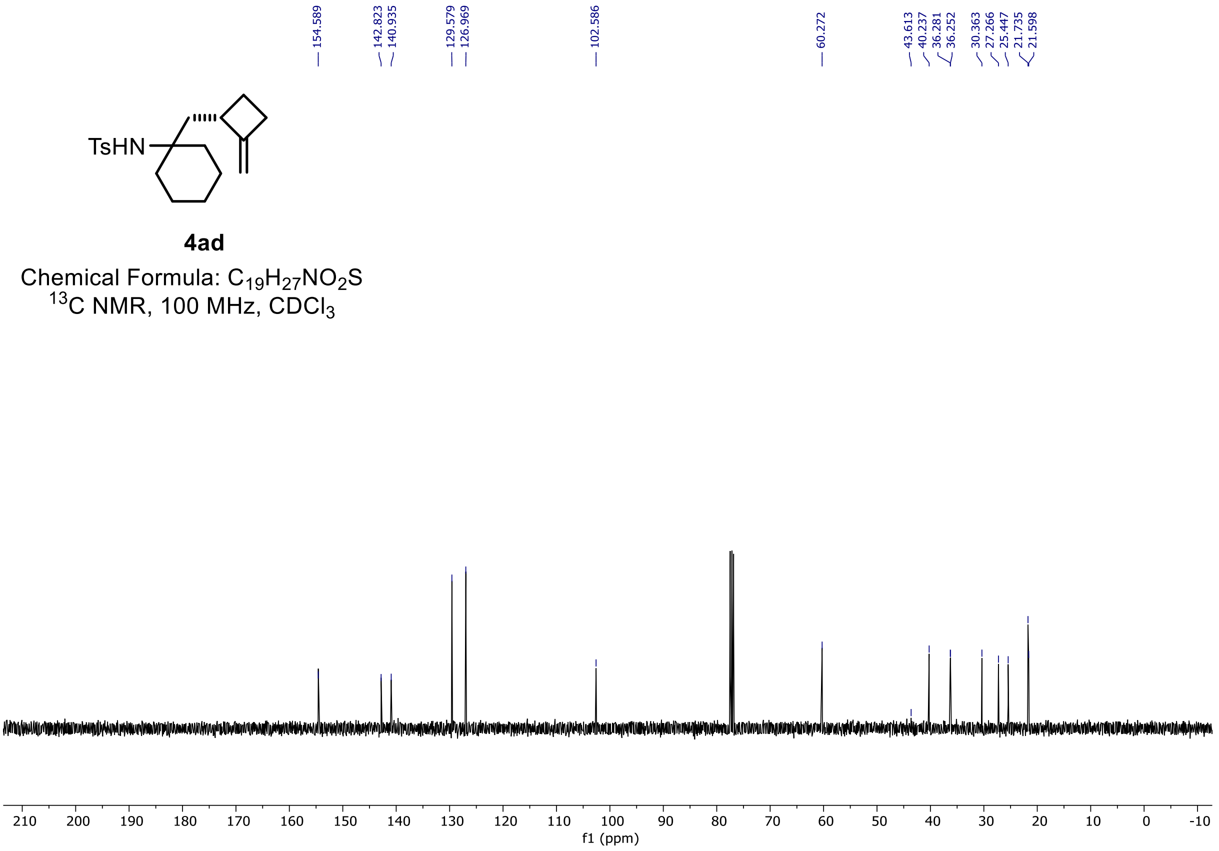
154.589 142.823 140.935 129.579 126.969 102.586 60.272 43.613 40.237 36.281 36.252 30.363 27.266 25.447 21.735 21.598
TsHN
4ad
Chemical Formula: C19H27NO2S
13C NMR, 100 MHz, CDCl3
210 200 190 180 170 160 150 140 130 120 110 100 90 80 70 60 50 40 30 20 10 0 -10
f1 (ppm)

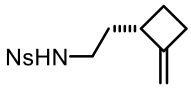

4ae

Chemical Formula: $C_{13}H_{16}N_2O_4S$

^{1}H NMR, 400 MHz, $CDCl_3$

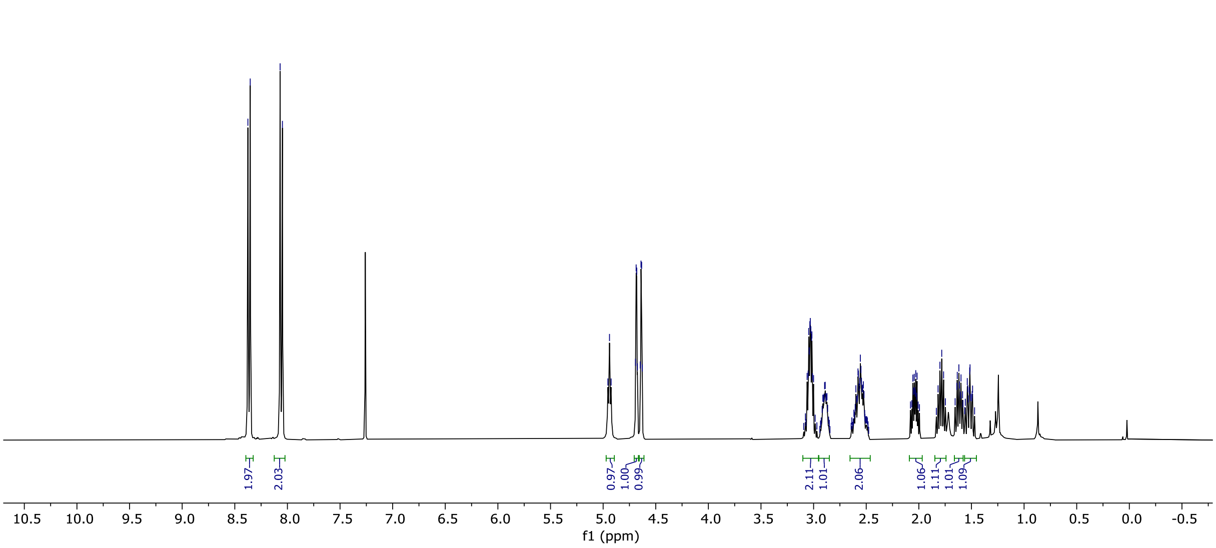

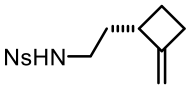

4ae

Chemical Formula: $C_{13}H_{16}N_2O_4S$

^{13}C NMR, 100 MHz, $CDCl_3$

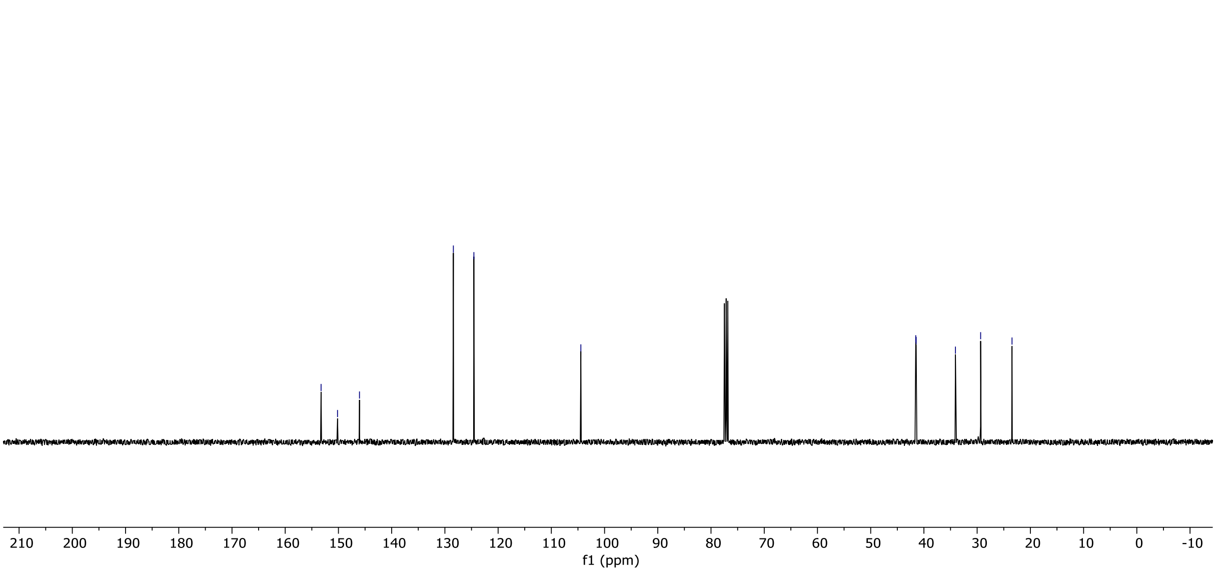

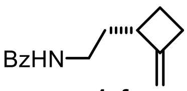

4af

Chemical Formula: $C_{14}H_{17}NO$

^{1}H NMR, 400 MHz, $CDCl_3$

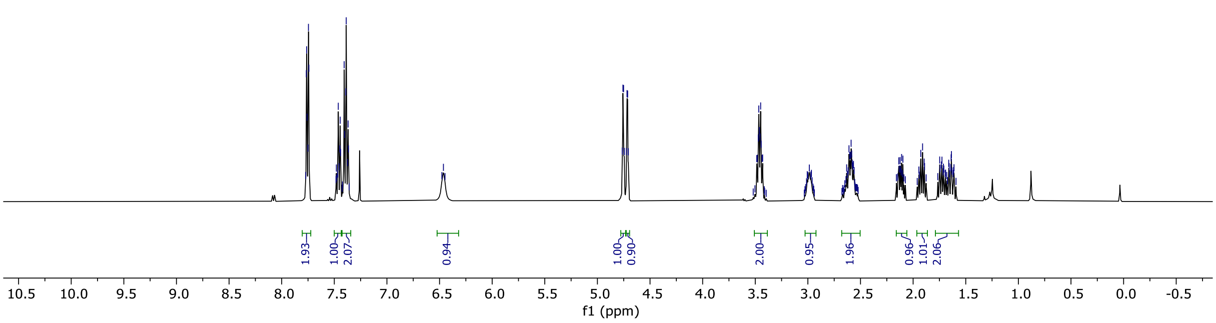

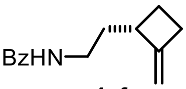

4af

Chemical Formula: $C_{14}H_{17}NO$

^{13}C NMR, 100 MHz, $CDCl_3$

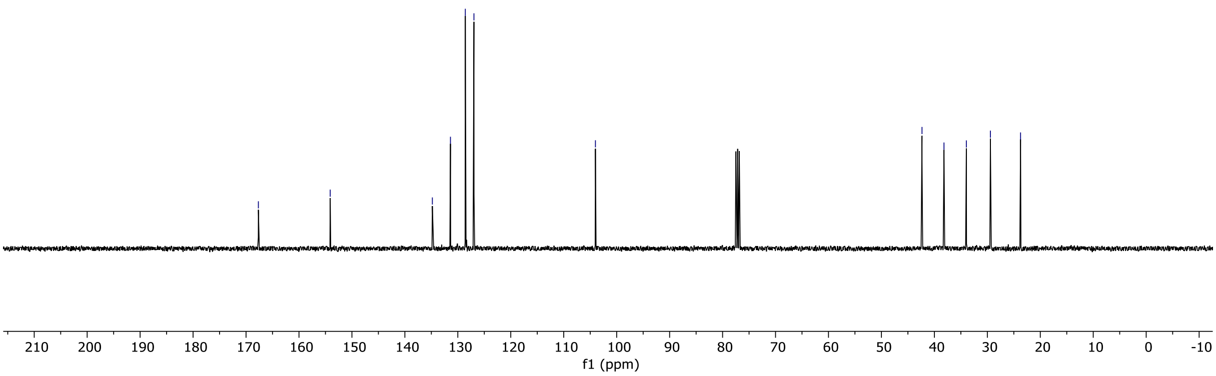

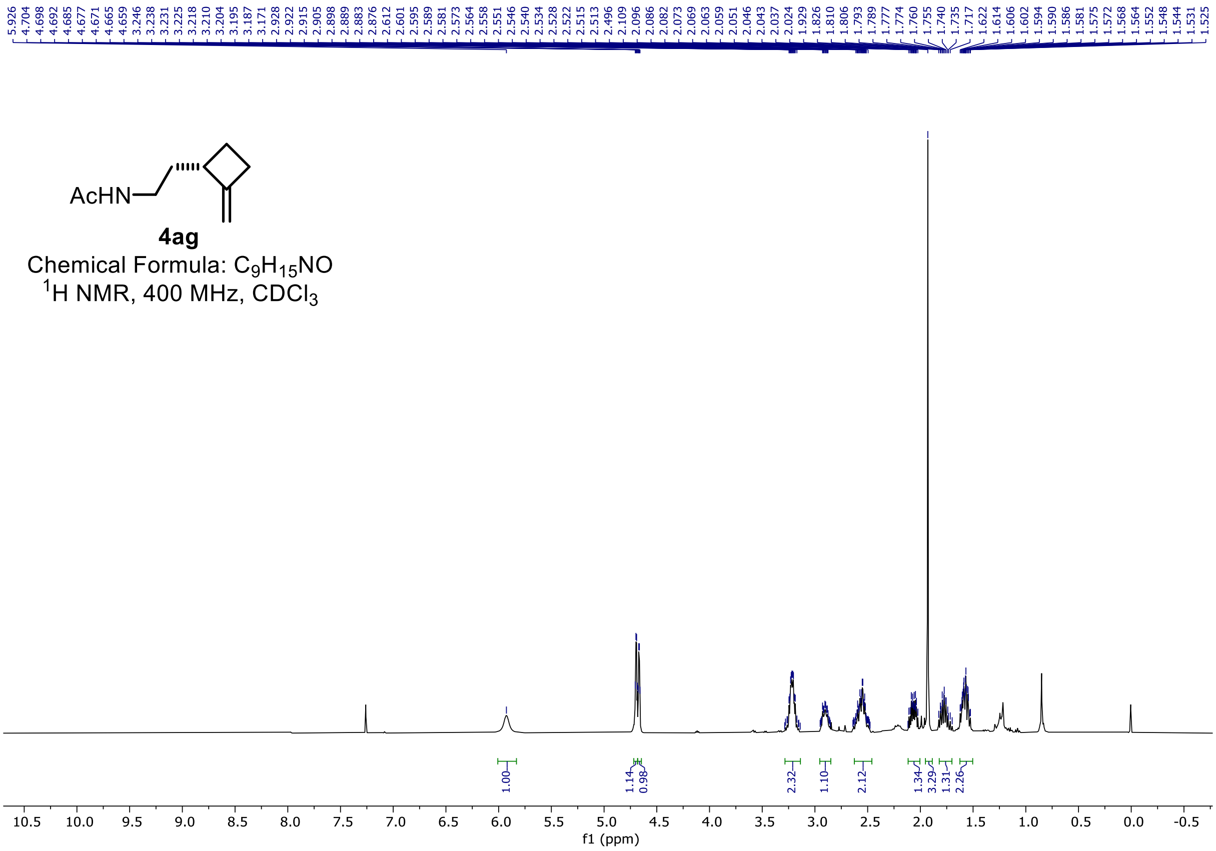
5.926 4.704 4.698 4.692 4.685 4.677 4.671 4.665 4.659 3.246 3.238 3.231 3.225 3.218 3.210 3.204 3.195 3.187 3.171 2.928 2.922 2.915 2.905 2.898 2.889 2.883 2.876 2.612 2.601 2.595 2.589 2.581 2.573 2.564 2.558 2.551 2.546 2.540 2.534 2.528 2.522 2.515 2.513 2.496 2.109 2.096 2.086 2.082 2.073 2.069 2.063 2.059 2.051 2.046 2.043 2.037 2.024 1.929 1.826 1.810 1.806 1.793 1.789 1.777 1.774 1.760 1.755 1.740 1.735 1.717 1.622 1.614 1.606 1.602 1.594 1.590 1.586 1.581 1.575 1.572 1.568 1.564 1.552 1.548 1.544 1.531 1.525
AcHN
4ag
Chemical Formula: C9H15NO
1H NMR, 400 MHz, CDCl3
1.00
1.14
0.98
2.32
1.10
2.12
1.34
3.29
1.31
2.26
10.5 10.0 9.5 9.0 8.5 8.0 7.5 7.0 6.5 6.0 5.5 5.0 4.5 4.0 3.5 3.0 2.5 2.0 1.5 1.0 0.5 0.0 -0.5
f1 (ppm)

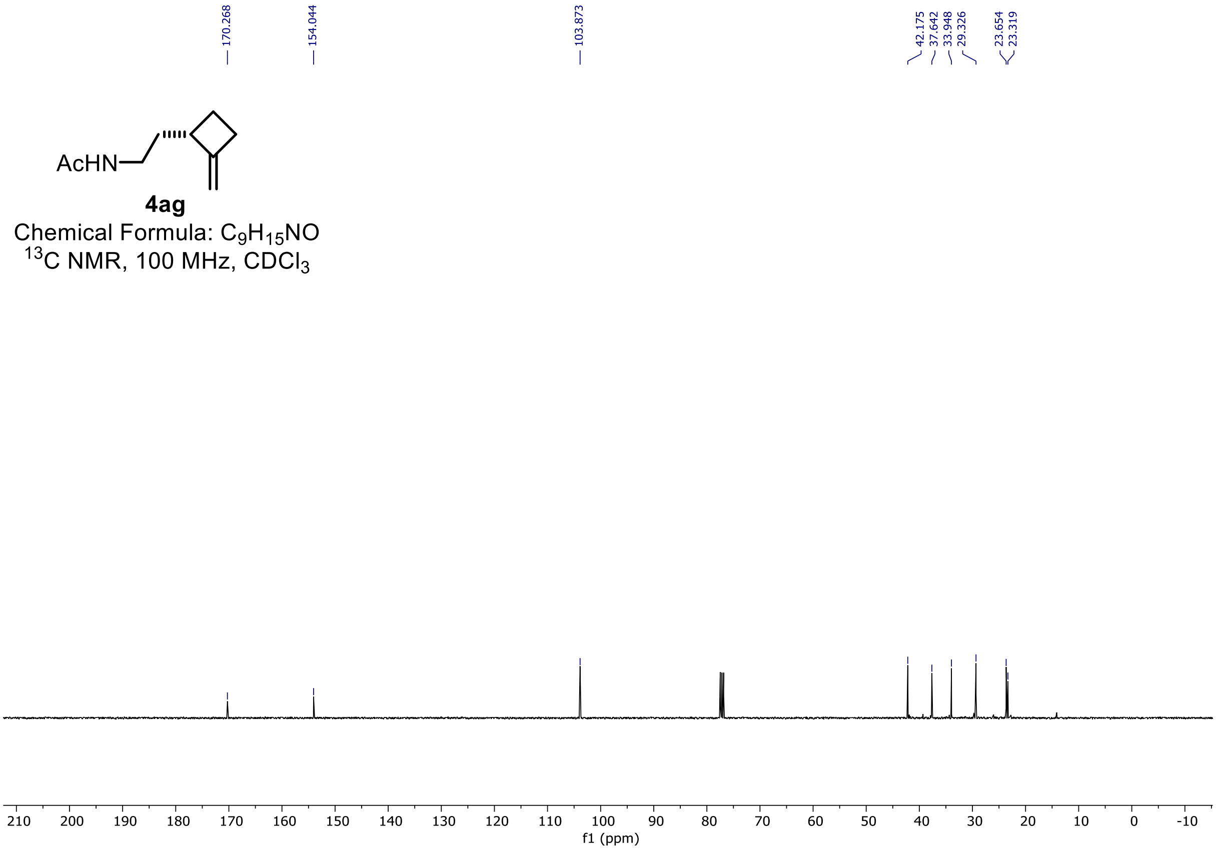
170.268
154.044
103.873
42.175 37.642 33.948 29.326
23.654 23.319
AcHN
4ag
Chemical Formula: C9H15NO
13C NMR, 100 MHz, CDCl3
210 200 190 180 170 160 150 140 130 120 110 100 90 80 70 60 50 40 30 20 10 0 -10
f1 (ppm)

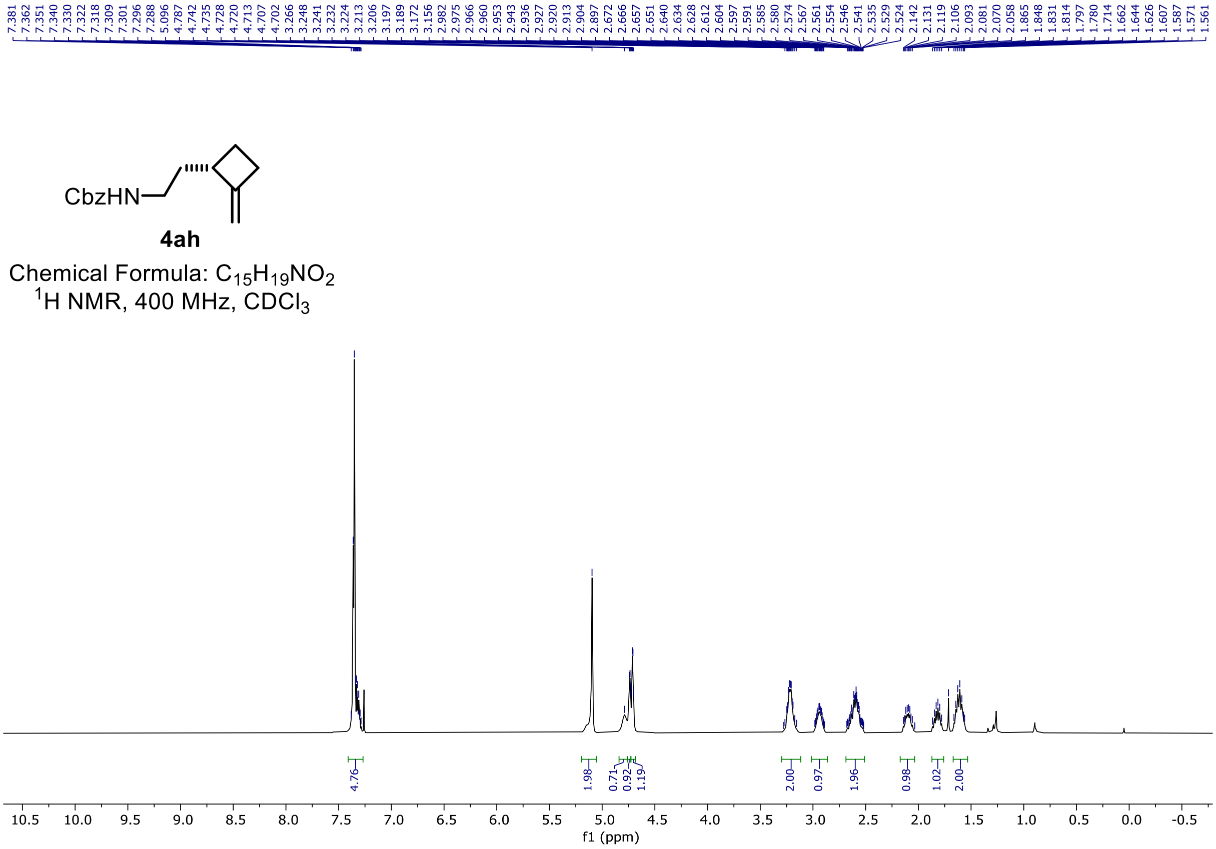
7.381 7.362 7.351 7.340 7.330 7.322 7.318 7.309 7.301 7.296 7.288 5.096 4.787 4.742 4.735 4.728 4.720 4.713 4.707 4.702 3.266 3.248 3.241 3.232 3.224 3.213 3.206 3.197 3.189 3.172 3.156 2.982 2.975 2.966 2.960 2.953 2.943 2.936 2.927 2.920 2.913 2.904 2.897 2.672 2.666 2.657 2.651 2.640 2.634 2.628 2.612 2.604 2.597 2.591 2.585 2.580 2.574 2.567 2.561 2.554 2.546 2.541 2.535 2.529 2.524 2.142 2.131 2.119 2.106 2.093 2.081 2.070 2.058 1.865 1.848 1.831 1.814 1.797 1.780 1.714 1.662 1.644 1.626 1.607 1.587 1.571 1.561
CbzHN
4ah
Chemical Formula: $C_{15}H_{19}NO_2$
^{1}H NMR, 400 MHz, $CDCl_3$
4.76 1.98 0.71 0.92 1.19 2.00 0.97 1.96 0.98 1.02 2.00
10.5 10.0 9.5 9.0 8.5 8.0 7.5 7.0 6.5 6.0 5.5 5.0 4.5 4.0 3.5 3.0 2.5 2.0 1.5 1.0 0.5 0.0 -0.5
f1 (ppm)

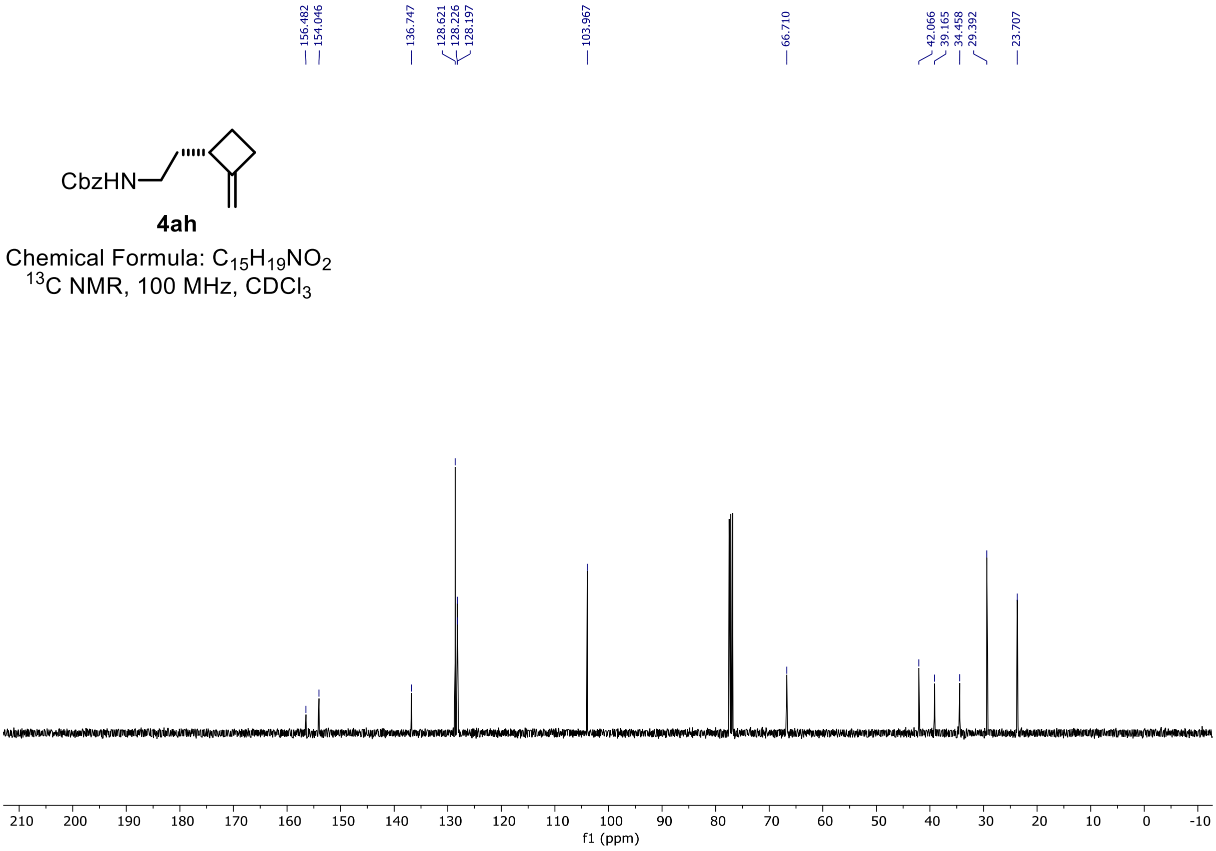
156.482 154.046 136.747 128.621 128.226 128.197 103.967 66.710 42.066 39.165 34.458 29.392 23.707
CbzHN
4ah
Chemical Formula: $C_{15}H_{19}NO_2$
^{13}C NMR, 100 MHz, $CDCl_3$
210 200 190 180 170 160 150 140 130 120 110 100 90 80 70 60 50 40 30 20 10 0 -10
f1 (ppm)

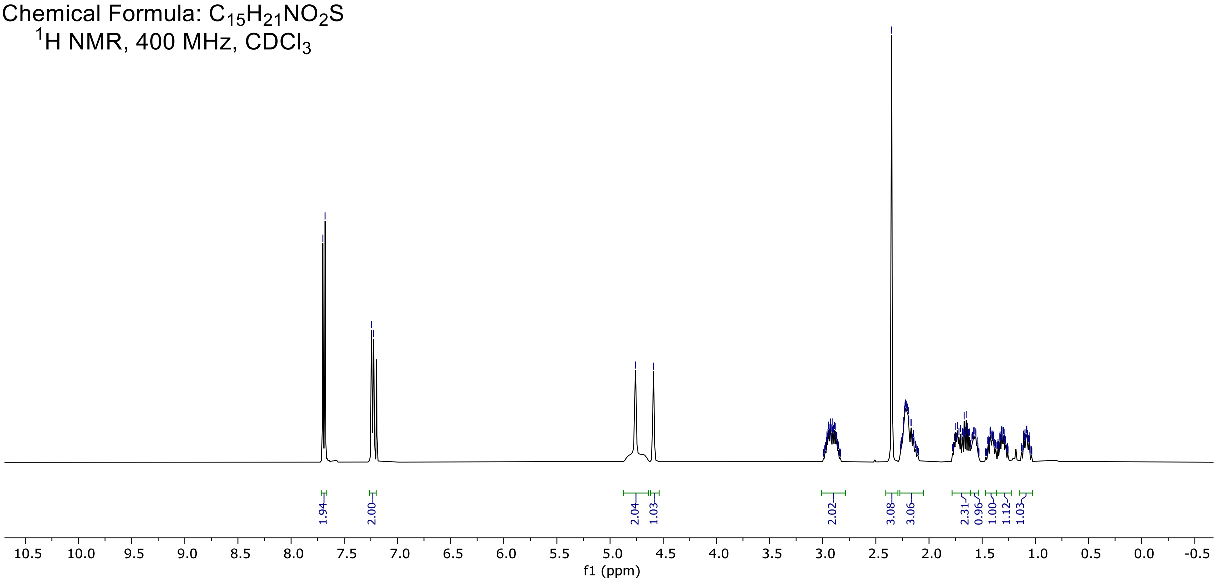

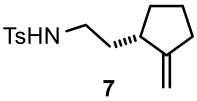

7

Chemical Formula: $C_{15}H_{21}NO_2S$

^{1}H NMR, 400 MHz, $CDCl_3$

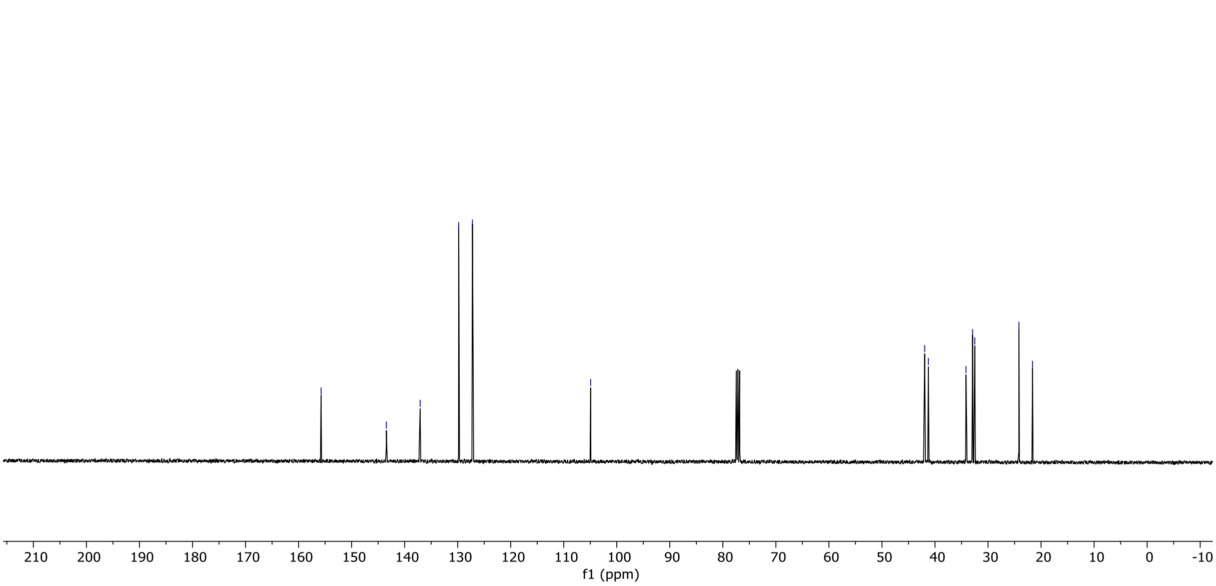

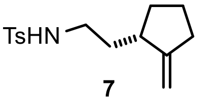

7

Chemical Formula: $C_{15}H_{21}NO_2S$

^{13}C NMR, 100 MHz, $CDCl_3$

5a

Chemical Formula: $C_{14}H_{18}FNO_2S$

^1H NMR, 400 MHz, $CDCl_3$

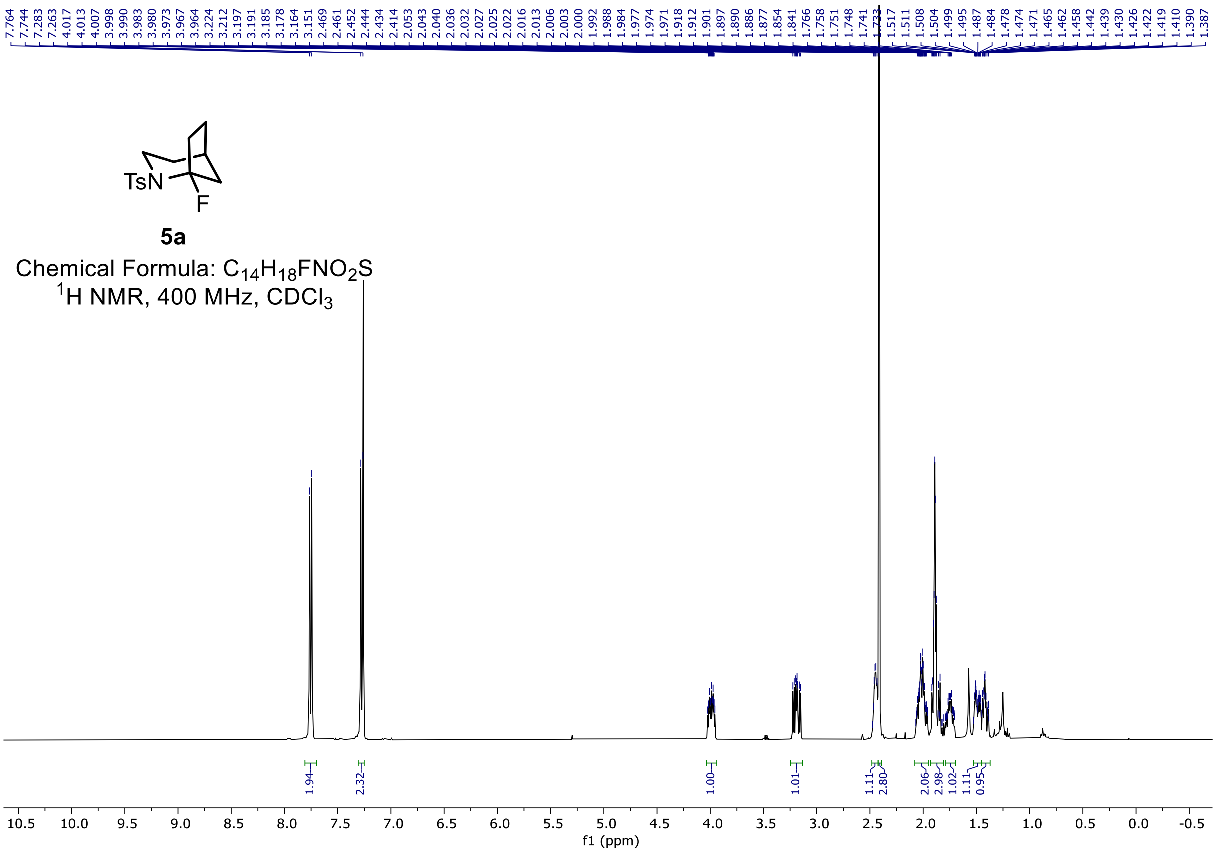

5a

Chemical Formula: $C_{14}H_{18}FNO_2S$

^{13}C NMR, 100 MHz, $CDCl_3$

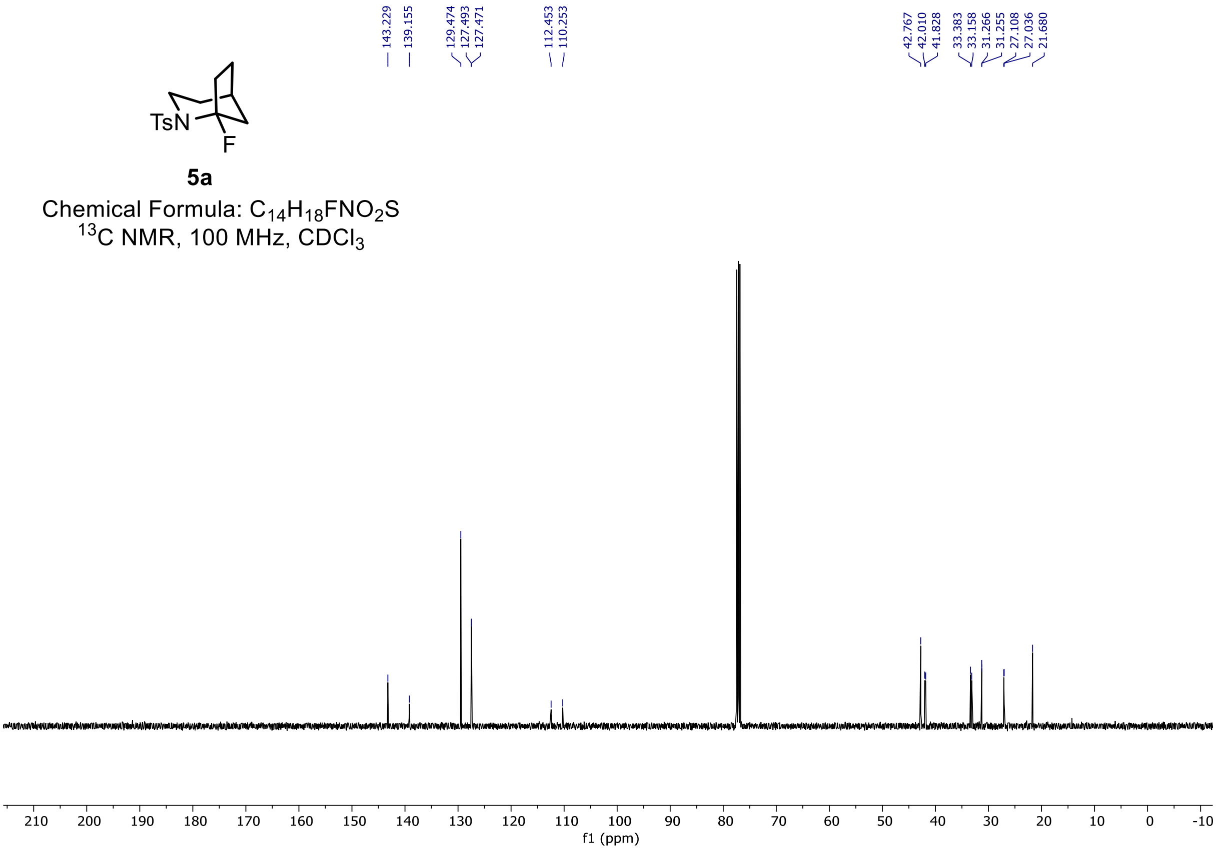

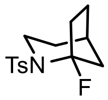

5a

Chemical Formula: $C_{14}H_{18}FNO_2S$

^{19}F NMR, 376 MHz, $CDCl_3$

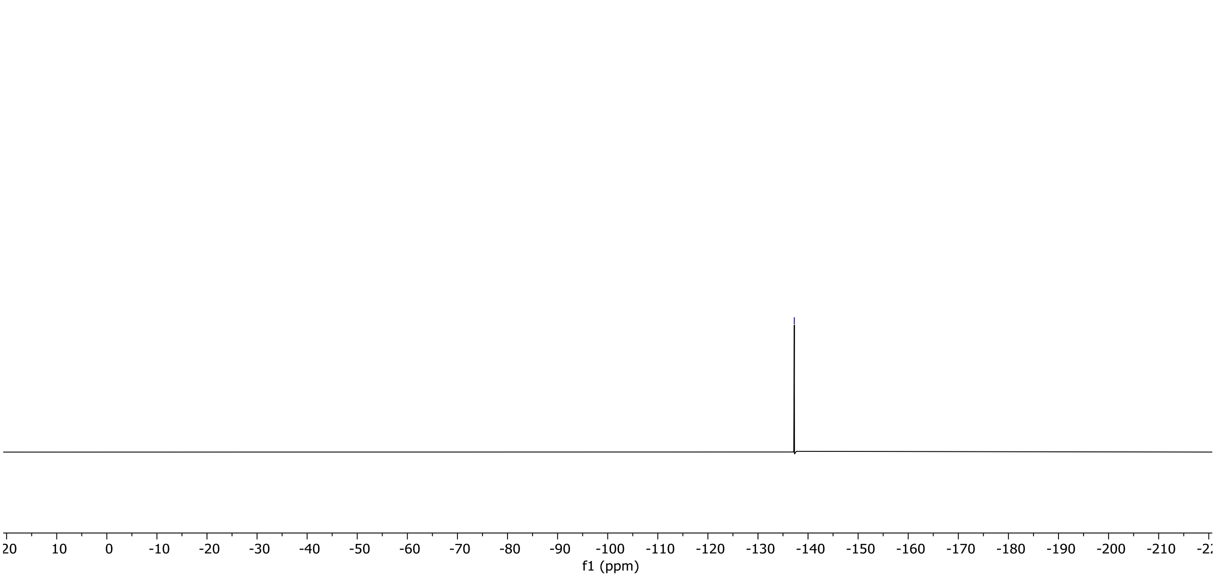

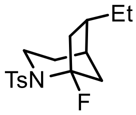

5b

Chemical Formula: $C_{16}H_{22}FNO_2S$

^{1}H NMR, 400 MHz, $CDCl_3$

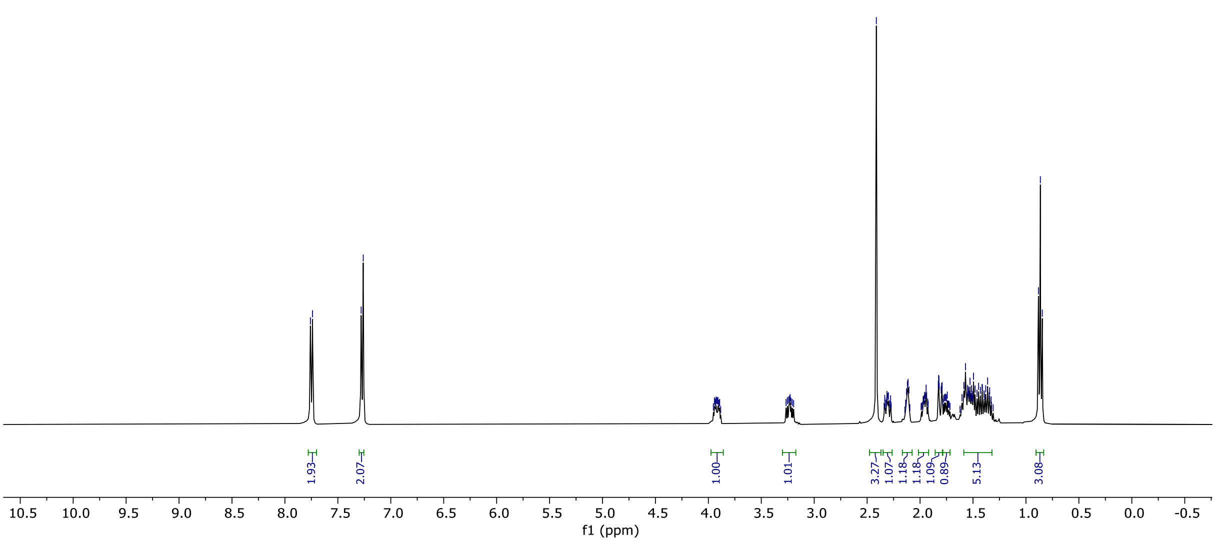

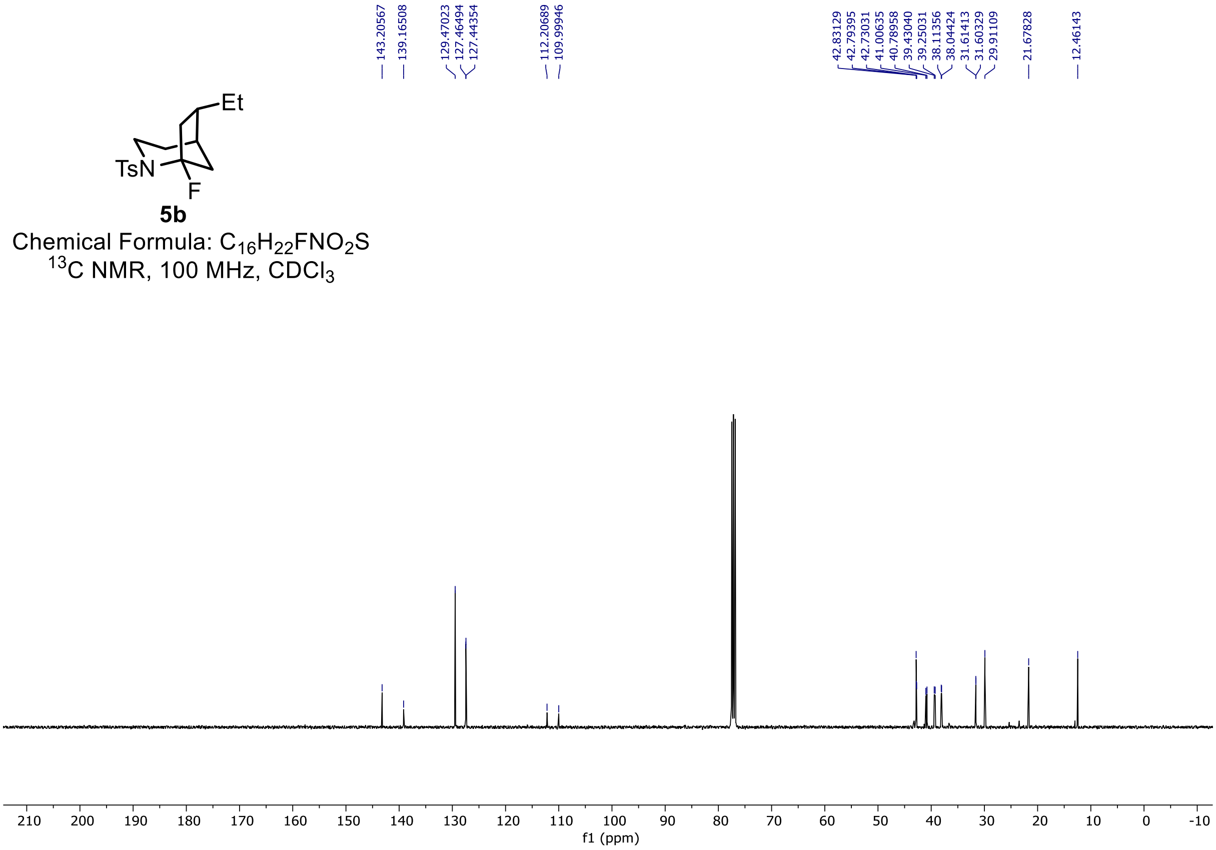

5b

Chemical Formula: $C_{16}H_{22}FNO_2S$

^{13}C NMR, 100 MHz, $CDCl_3$

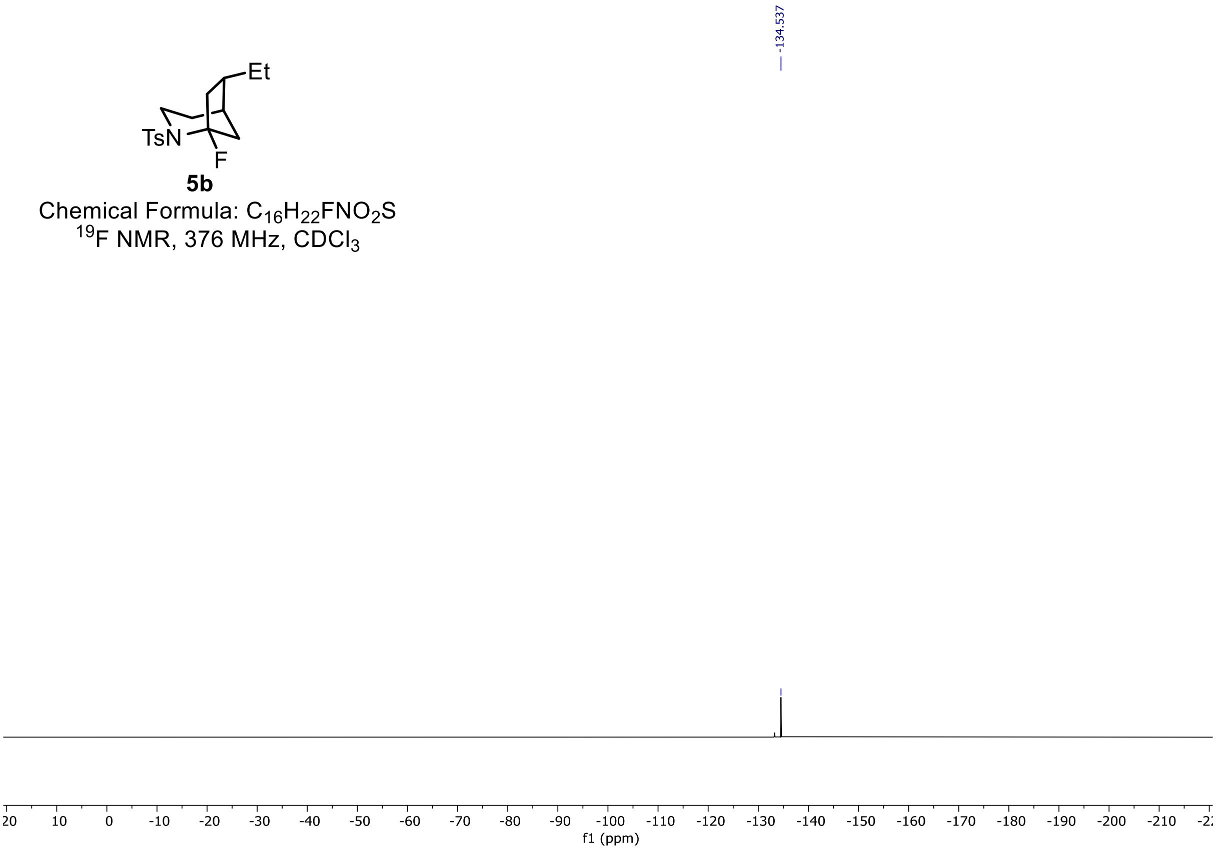

5b

Chemical Formula: $C_{16}H_{22}FNO_2S$

^{19}F NMR, 376 MHz, $CDCl_3$

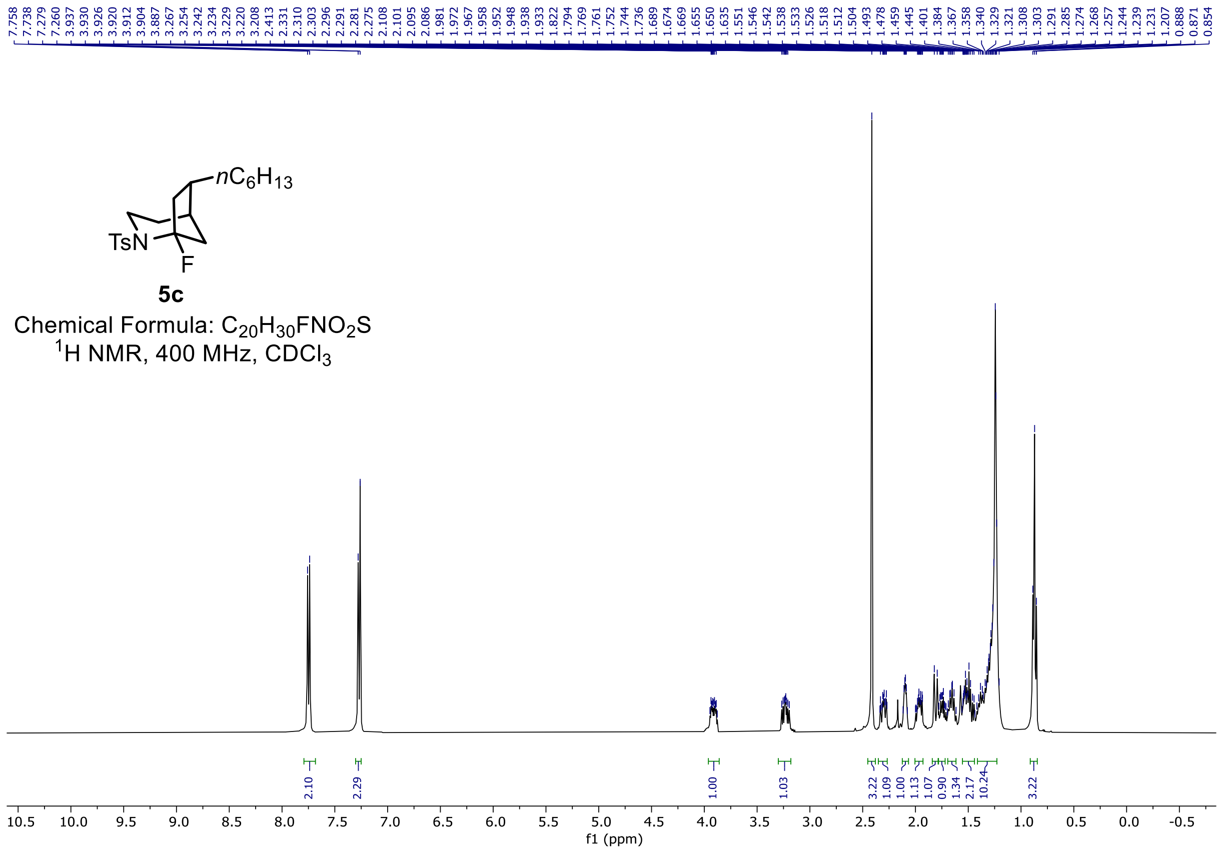

5c

Chemical Formula: $C_{20}H_{30}FNO_2S$

^1H NMR, 400 MHz, $CDCl_3$

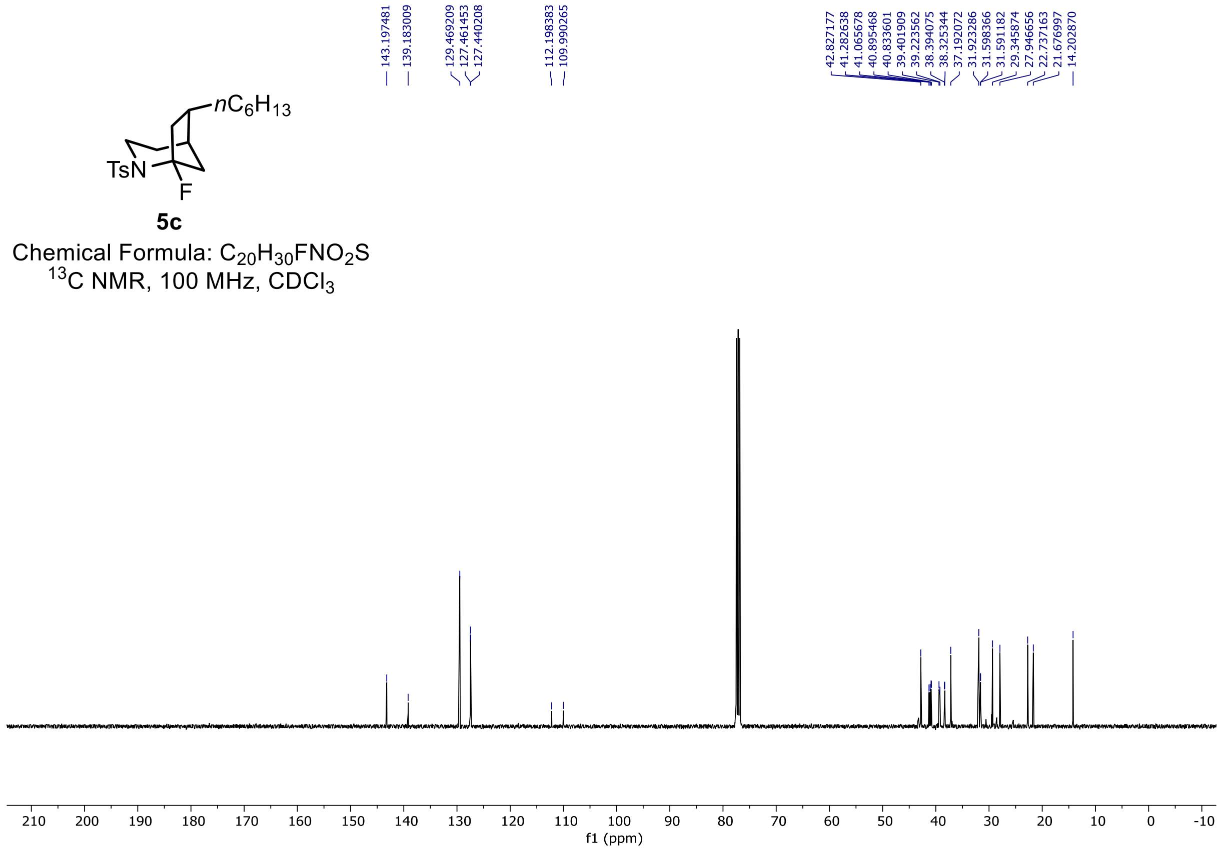

5c

Chemical Formula: $C_{20}H_{30}FNO_2S$

^{13}C NMR, 100 MHz, $CDCl_3$

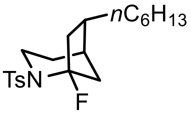

5c

Chemical Formula: $C_{20}H_{30}FNO_2S$

^{19}F NMR, 376 MHz, $CDCl_3$

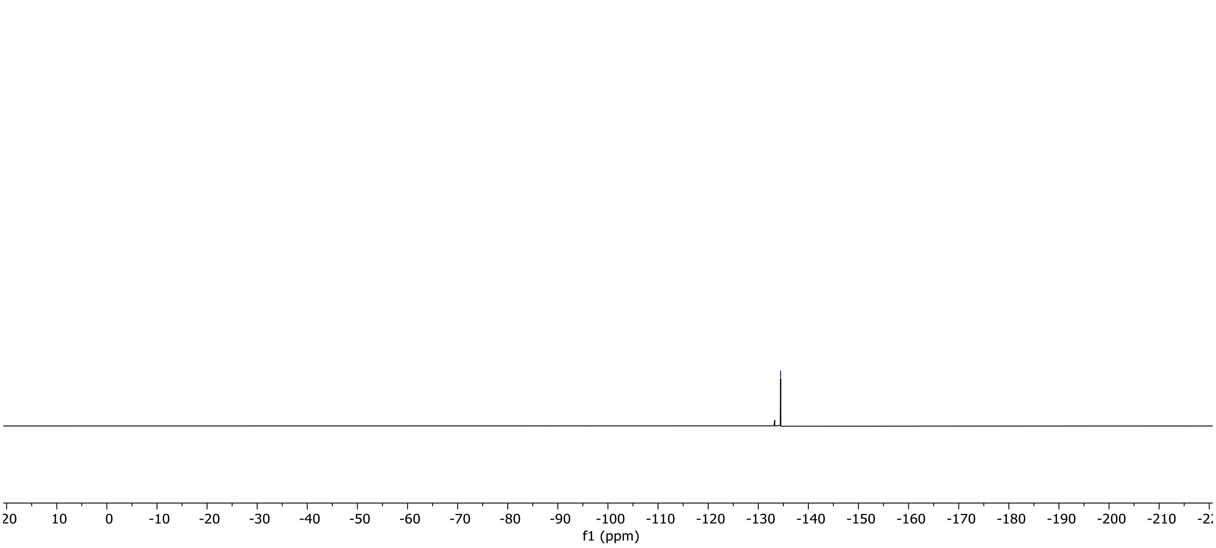

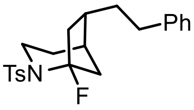

5d

Chemical Formula: $C_{22}H_{26}FNO_2S$

^{1}H NMR, 400 MHz, $CDCl_3$

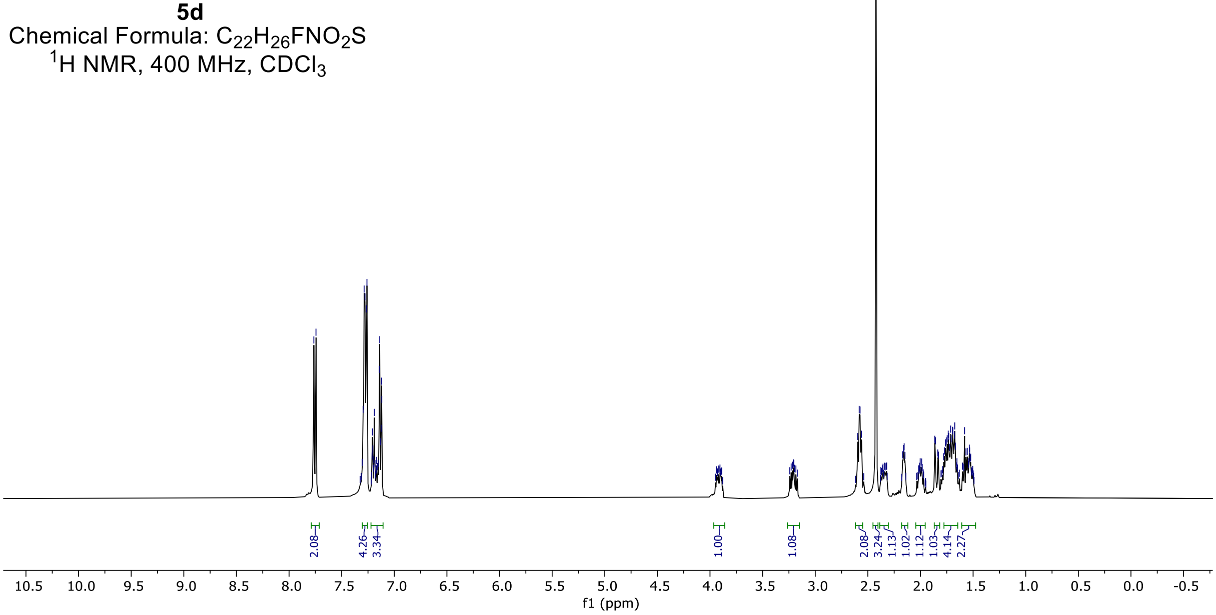

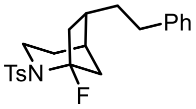

5d
Chemical Formula: $C_{22}H_{26}FNO_2S$
^{13}C NMR, 100 MHz, $CDCl_3$

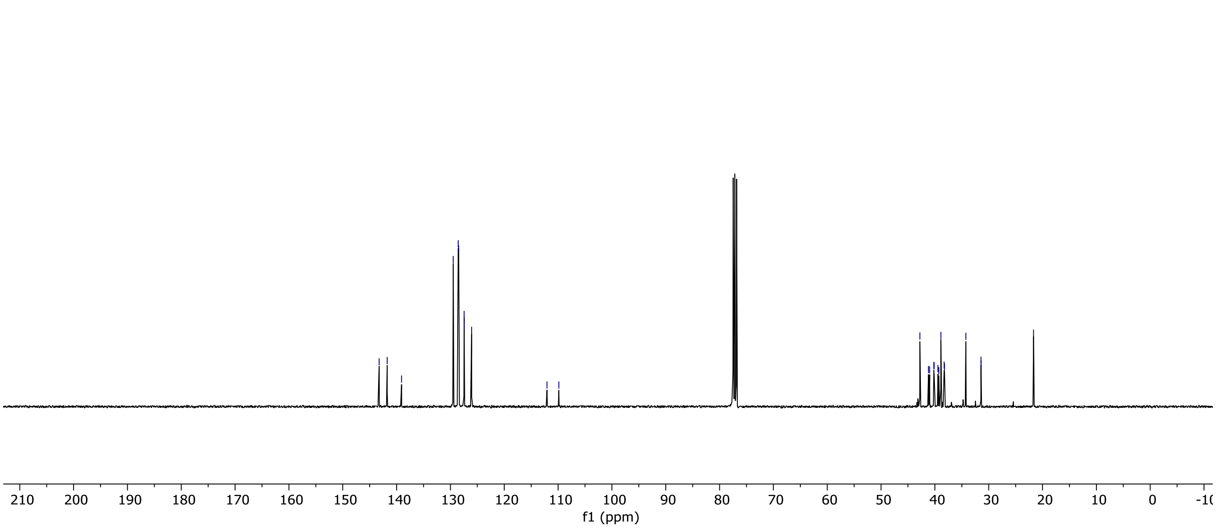

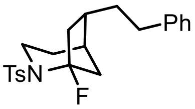

5d
Chemical Formula: $C_{22}H_{26}FNO_2S$
^{19}F NMR, 376 MHz, $CDCl_3$

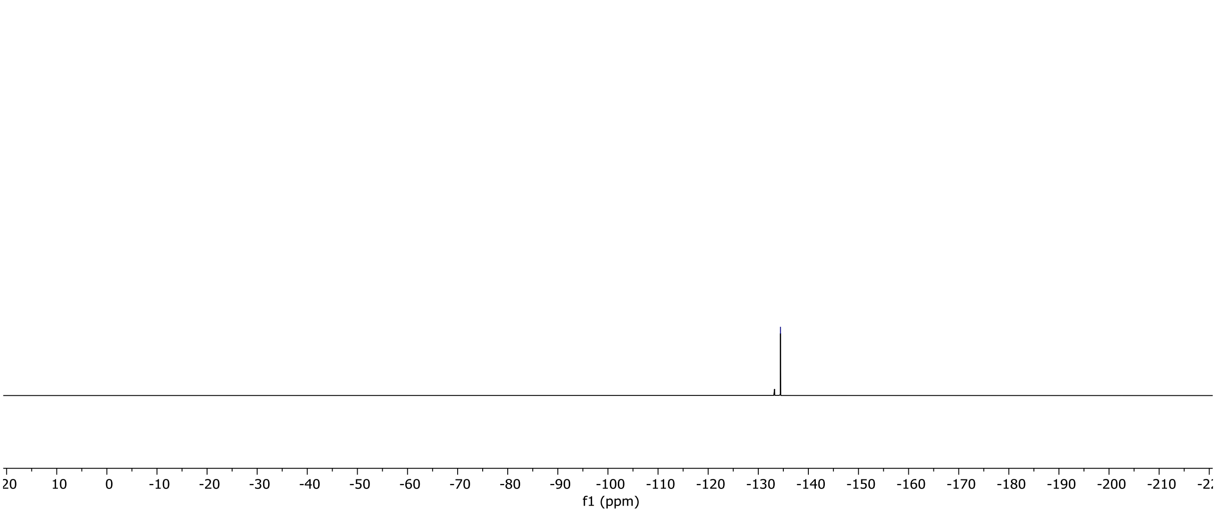

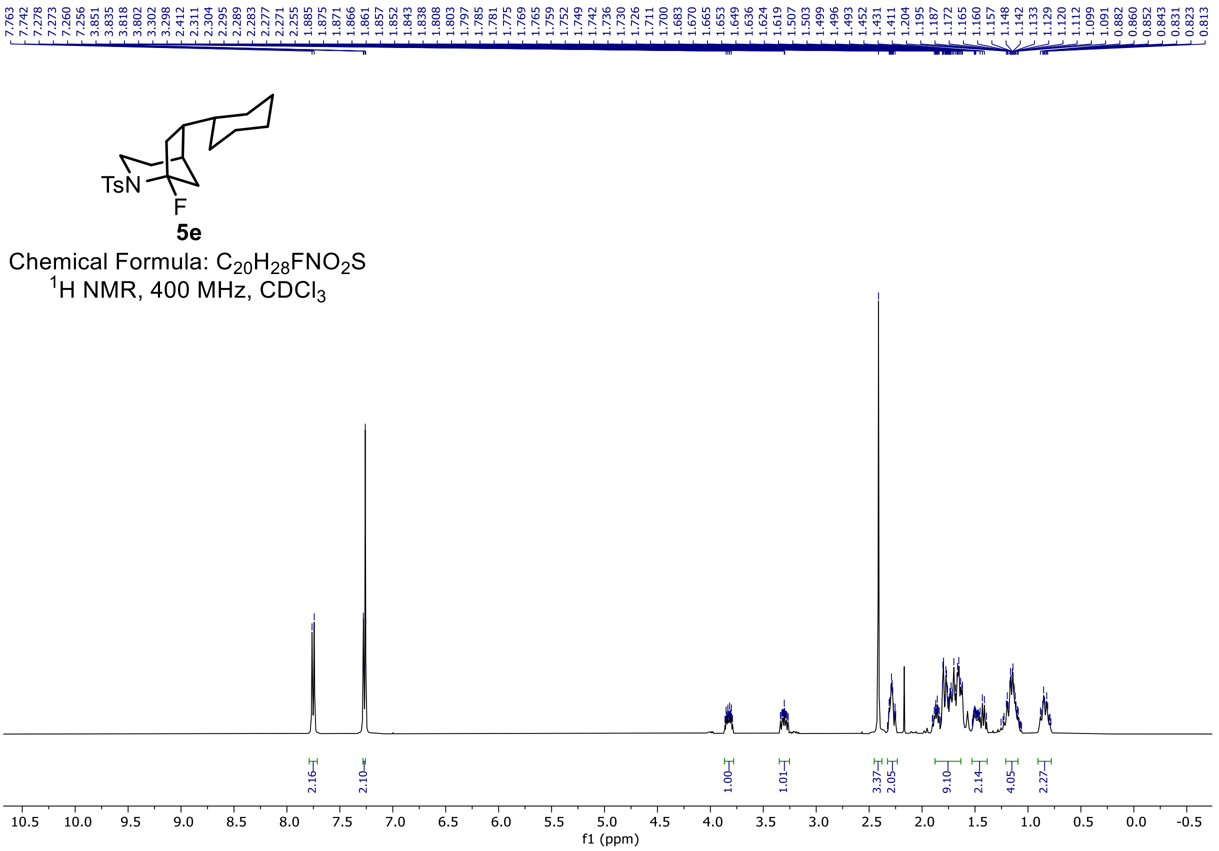

5e

Chemical Formula: $C_{20}H_{28}FNO_2S$

^{1}H NMR, 400 MHz, $CDCl_3$

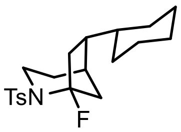

5e

Chemical Formula: $C_{20}H_{28}FNO_2S$

^{13}C NMR, 100 MHz, $CDCl_3$

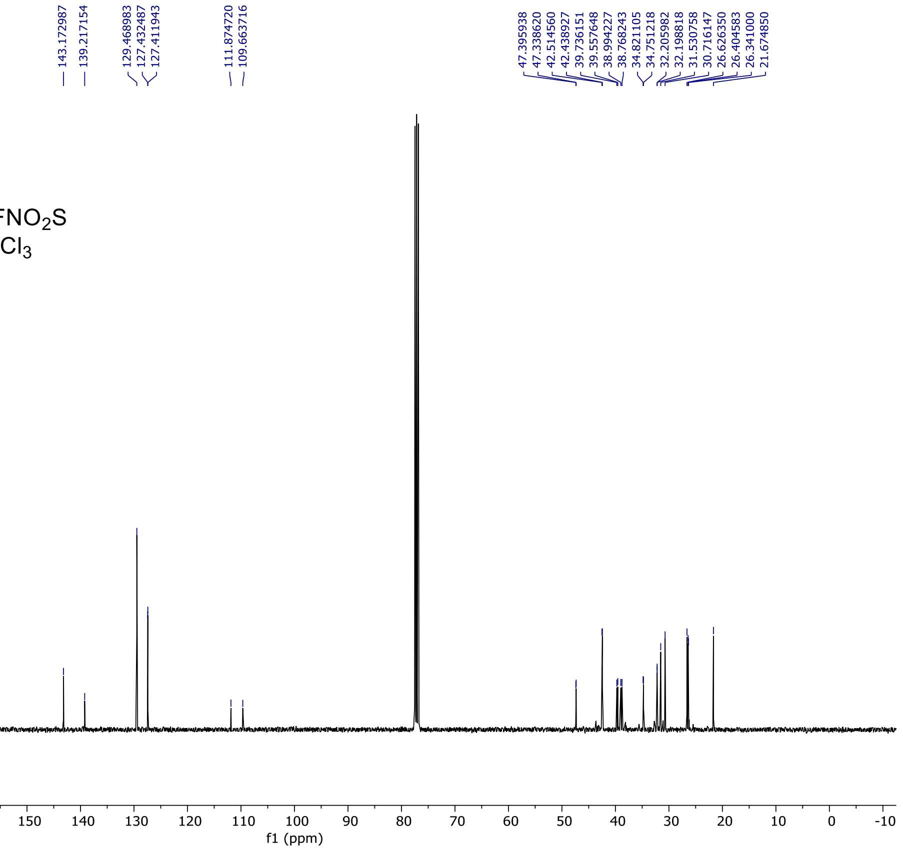

TsN F

5e

Chemical Formula: $C_{20}H_{28}FNO_2S$

^{19}F NMR, 376 MHz, $CDCl_3$

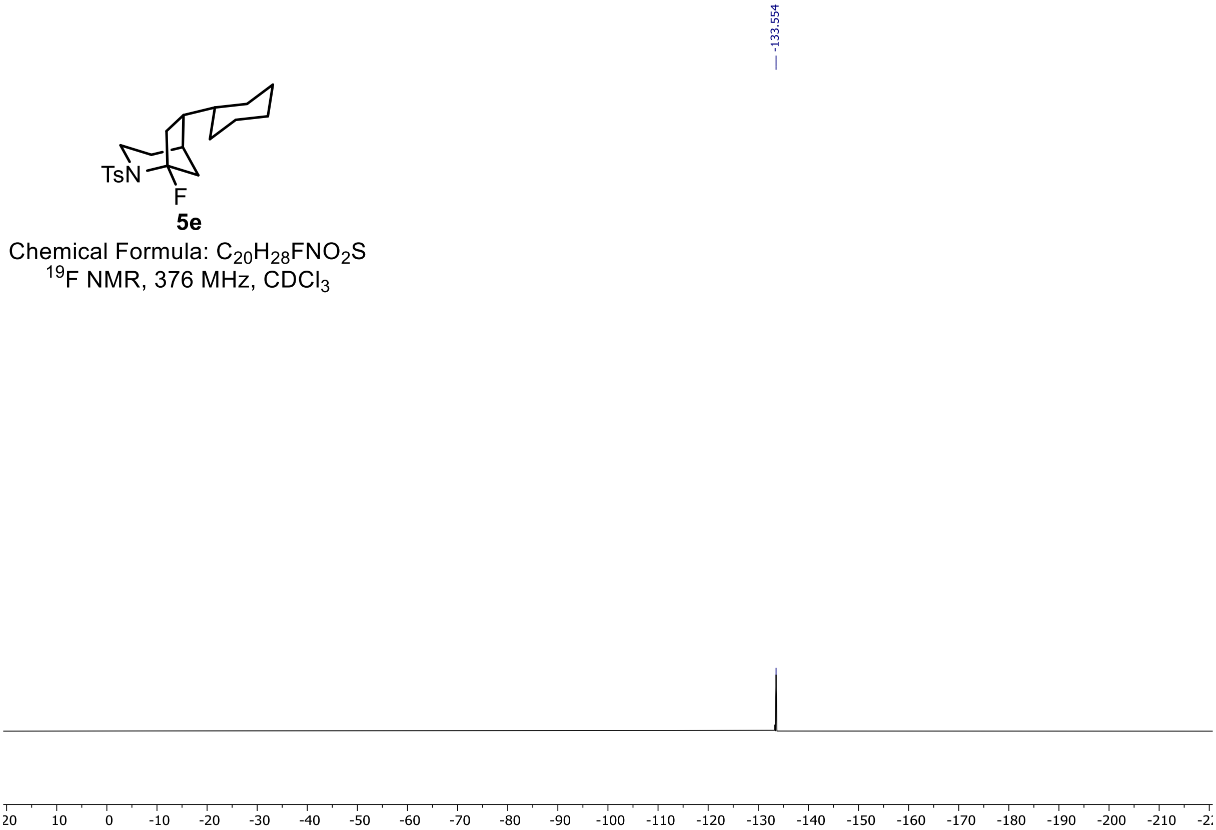

Ph TsN F

5f

Chemical Formula: $C_{20}H_{22}FNO_2S$

^{1}H NMR, 400 MHz, $CDCl_3$

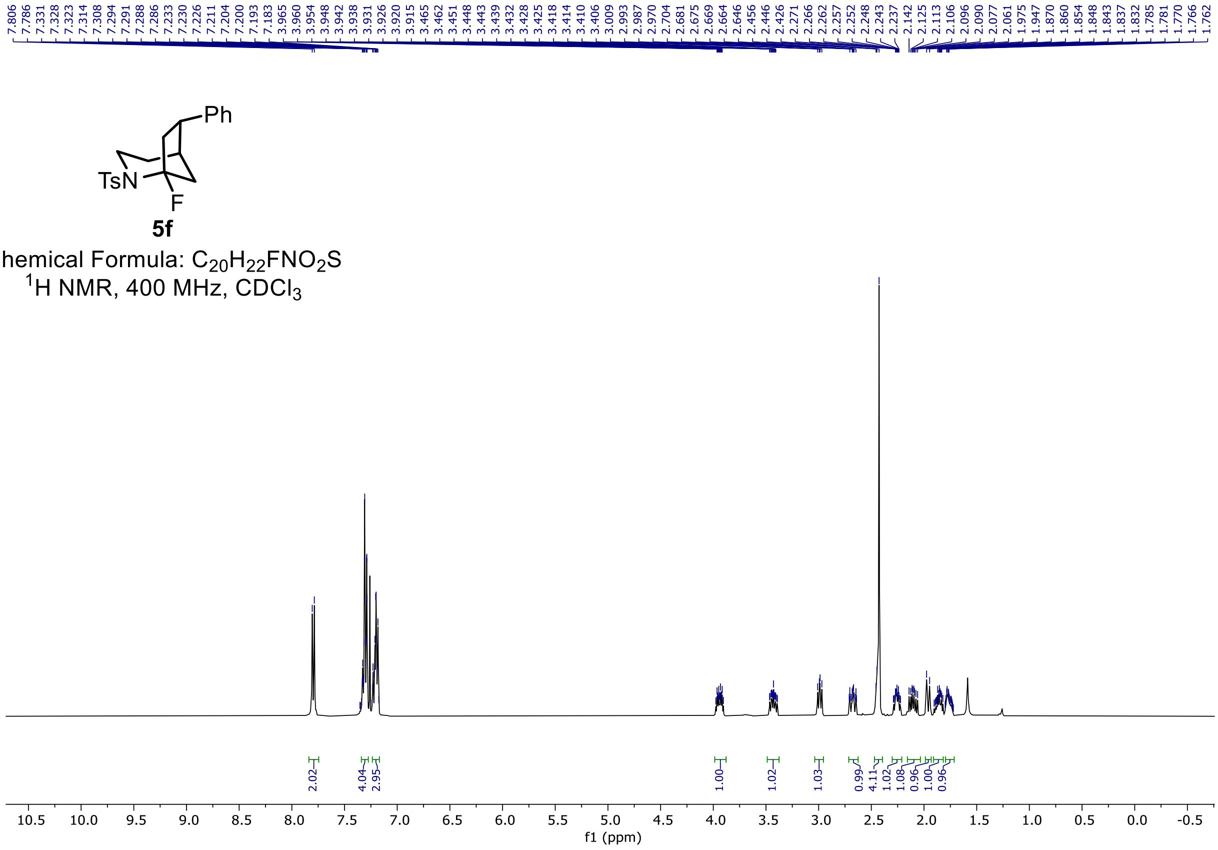

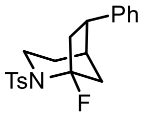

5f

Chemical Formula: $C_{20}H_{22}FNO_2S$
^{13}C NMR, 100 MHz, $CDCl_3$

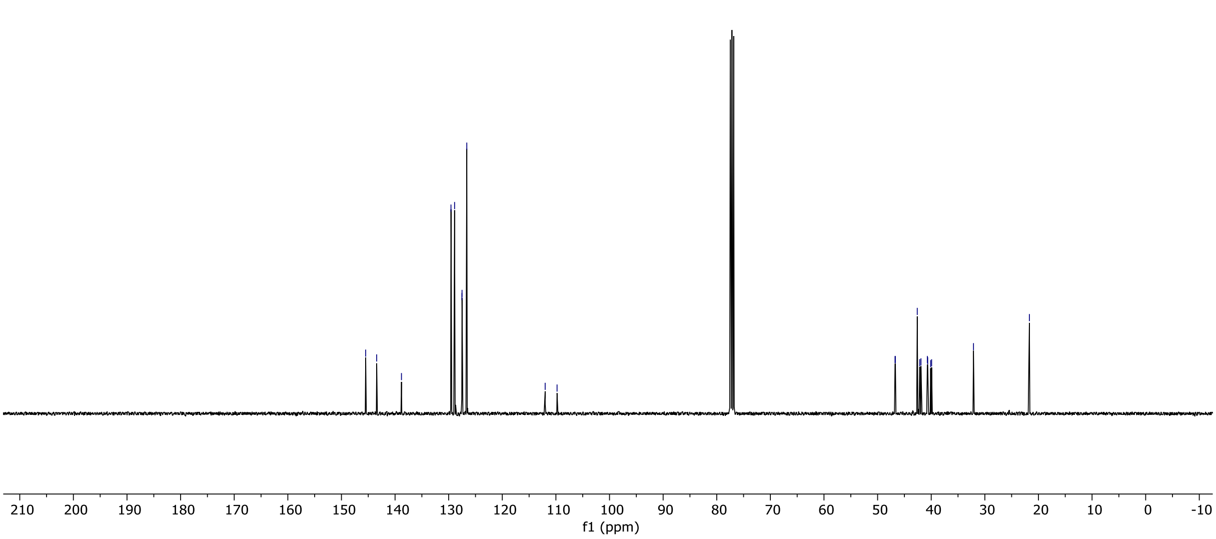

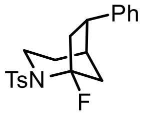

5f

Chemical Formula: $C_{20}H_{22}FNO_2S$
^{19}F NMR, 376 MHz, $CDCl_3$

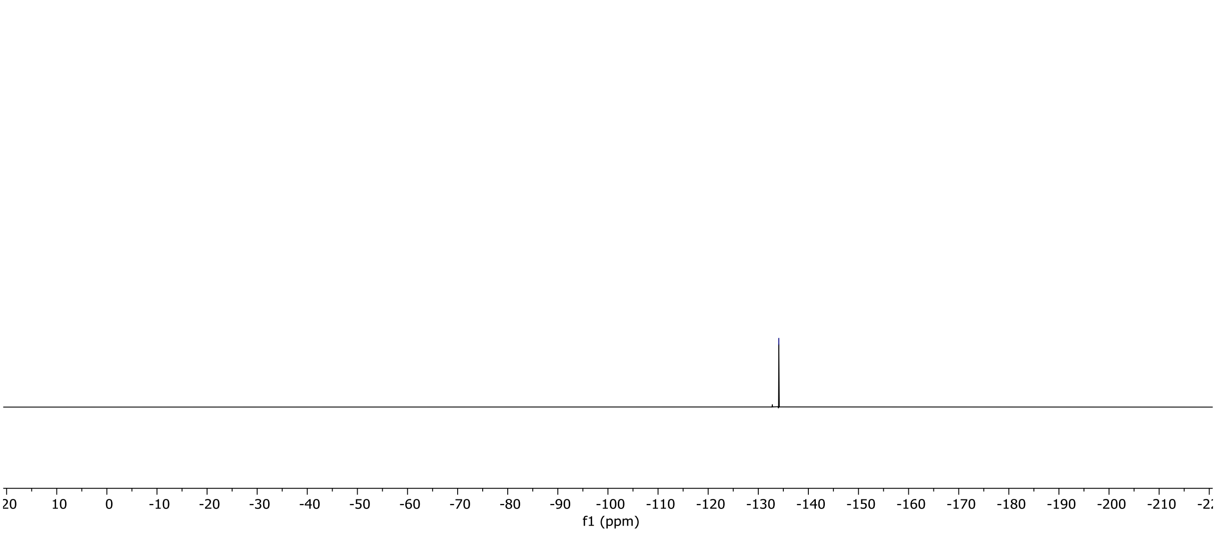

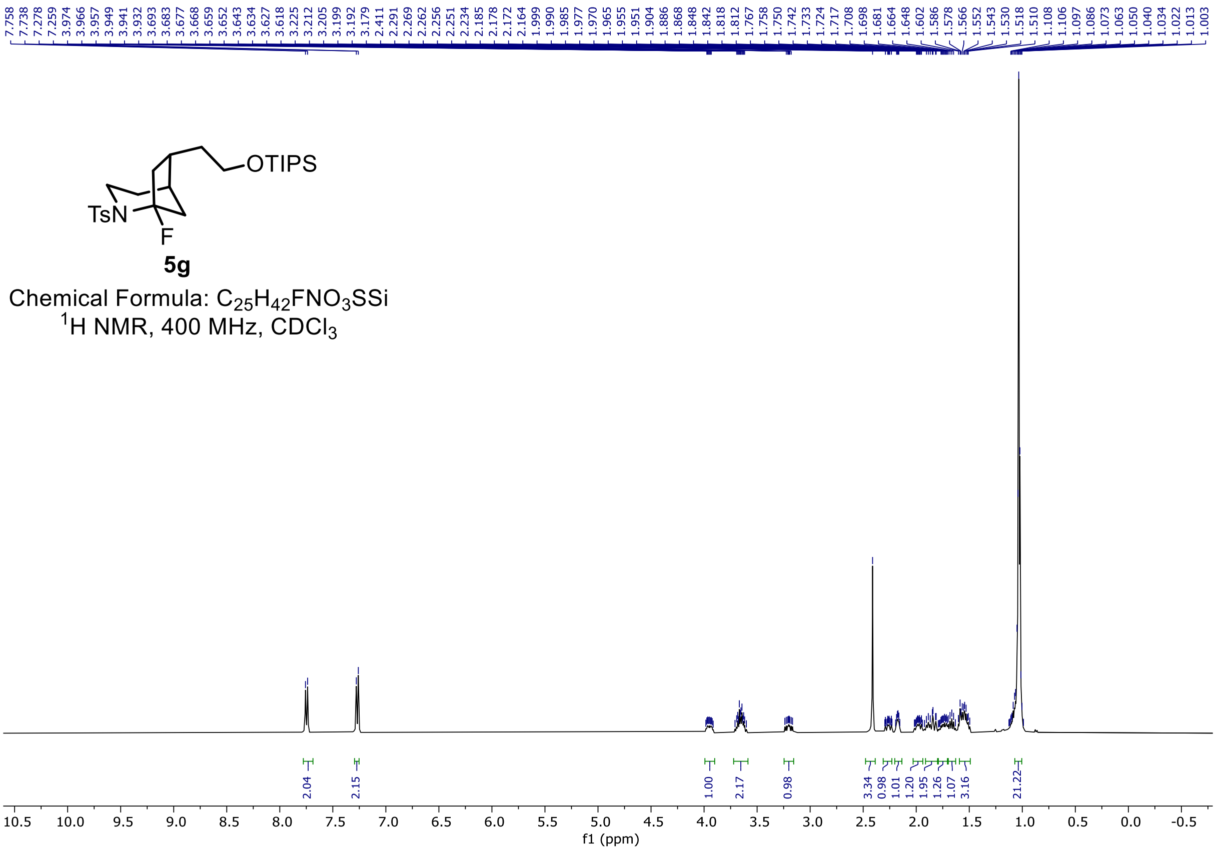
7.758 7.738 7.278 7.259 3.974 3.966 3.957 3.949 3.941 3.932 3.693 3.683 3.677 3.668 3.659 3.652 3.643 3.634 3.627 3.618 3.225 3.212 3.205 3.199 3.192 3.179 2.411 2.291 2.269 2.262 2.256 2.251 2.234 2.185 2.178 2.172 2.164 1.999 1.990 1.985 1.977 1.970 1.965 1.955 1.951 1.904 1.886 1.868 1.848 1.842 1.818 1.812 1.767 1.758 1.750 1.742 1.733 1.724 1.717 1.708 1.698 1.681 1.664 1.648 1.602 1.586 1.578 1.566 1.552 1.543 1.530 1.518 1.510 1.108 1.106 1.097 1.086 1.073 1.063 1.050 1.040 1.034 1.022 1.013 1.003
OTIPS
TsN
F
5g
Chemical Formula: C25H42FNO3SSi
1H NMR, 400 MHz, CDCl3
2.04
2.15
1.00
2.17
0.98
3.34
0.98
1.01
1.20
1.95
1.26
1.07
3.16
21.22
10.5 10.0 9.5 9.0 8.5 8.0 7.5 7.0 6.5 6.0 5.5 5.0 4.5 4.0 3.5 3.0 2.5 2.0 1.5 1.0 0.5 0.0 -0.5
f1 (ppm)

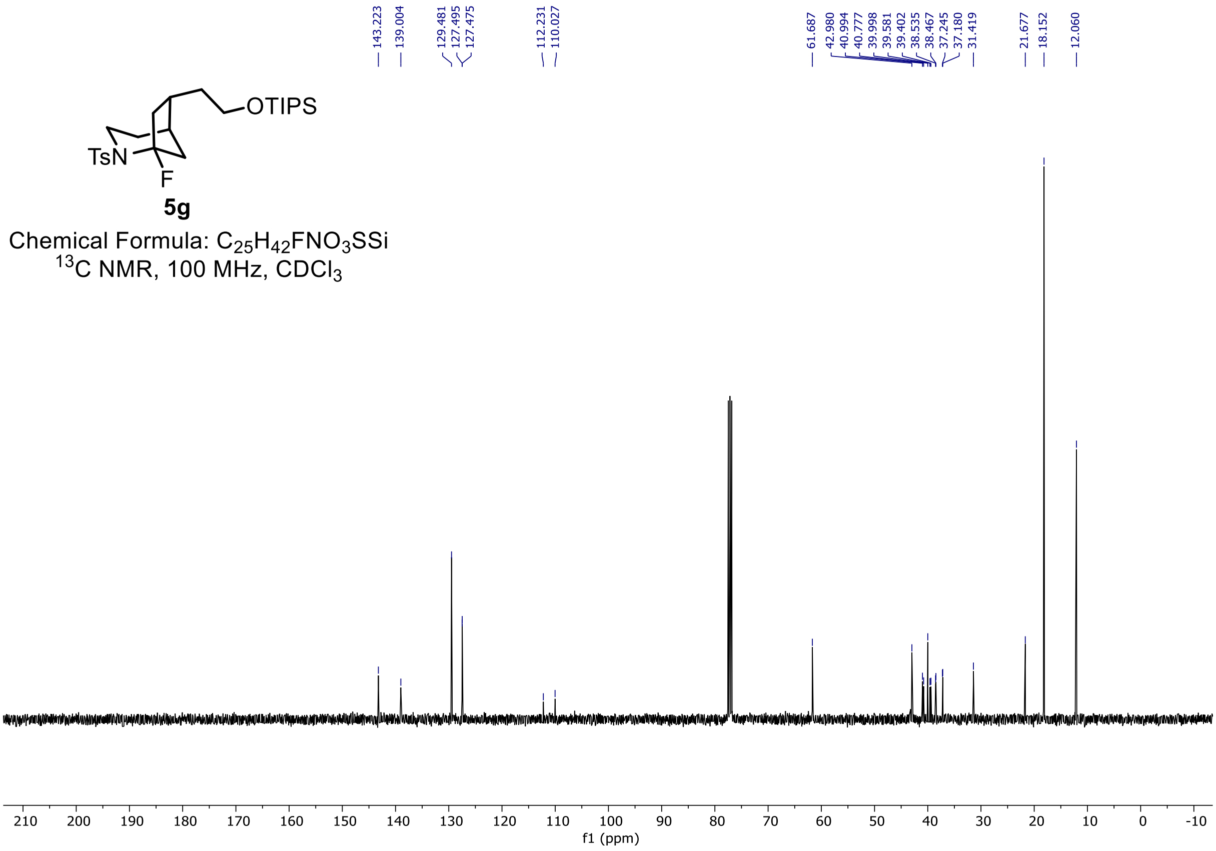
143.223
139.004
129.481
127.495
127.475
112.231
110.027
61.687
42.980
40.994
40.777
39.998
39.581
39.402
38.535
38.467
37.245
37.180
31.419
21.677
18.152
12.060
OTIPS
TsN
F
5g
Chemical Formula: C25H42FNO3SSi
13C NMR, 100 MHz, CDCl3
210 200 190 180 170 160 150 140 130 120 110 100 90 80 70 60 50 40 30 20 10 0 -10
f1 (ppm)

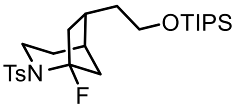

5g

Chemical Formula: $C_{25}H_{42}FNO_3SSi$

^{19}F NMR, 376 MHz, $CDCl_3$

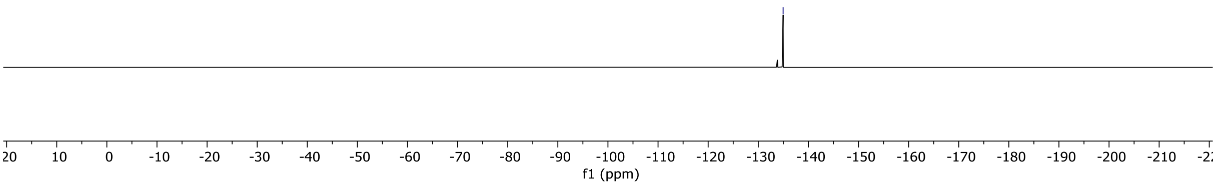

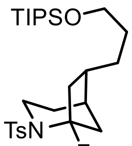

5h

Chemical Formula: $C_{26}H_{44}FNO_3SSi$

^{1}H NMR, 400 MHz, $CDCl_3$

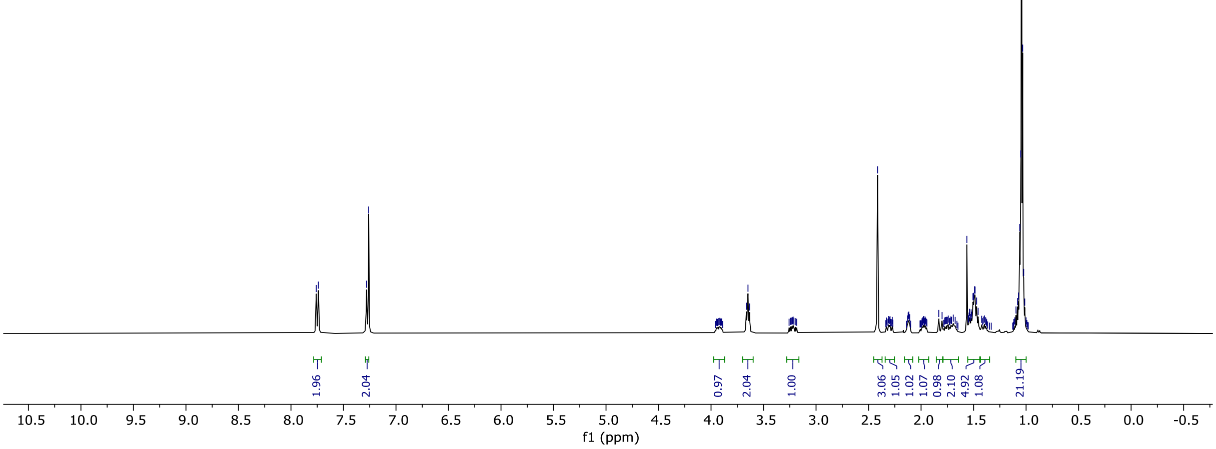

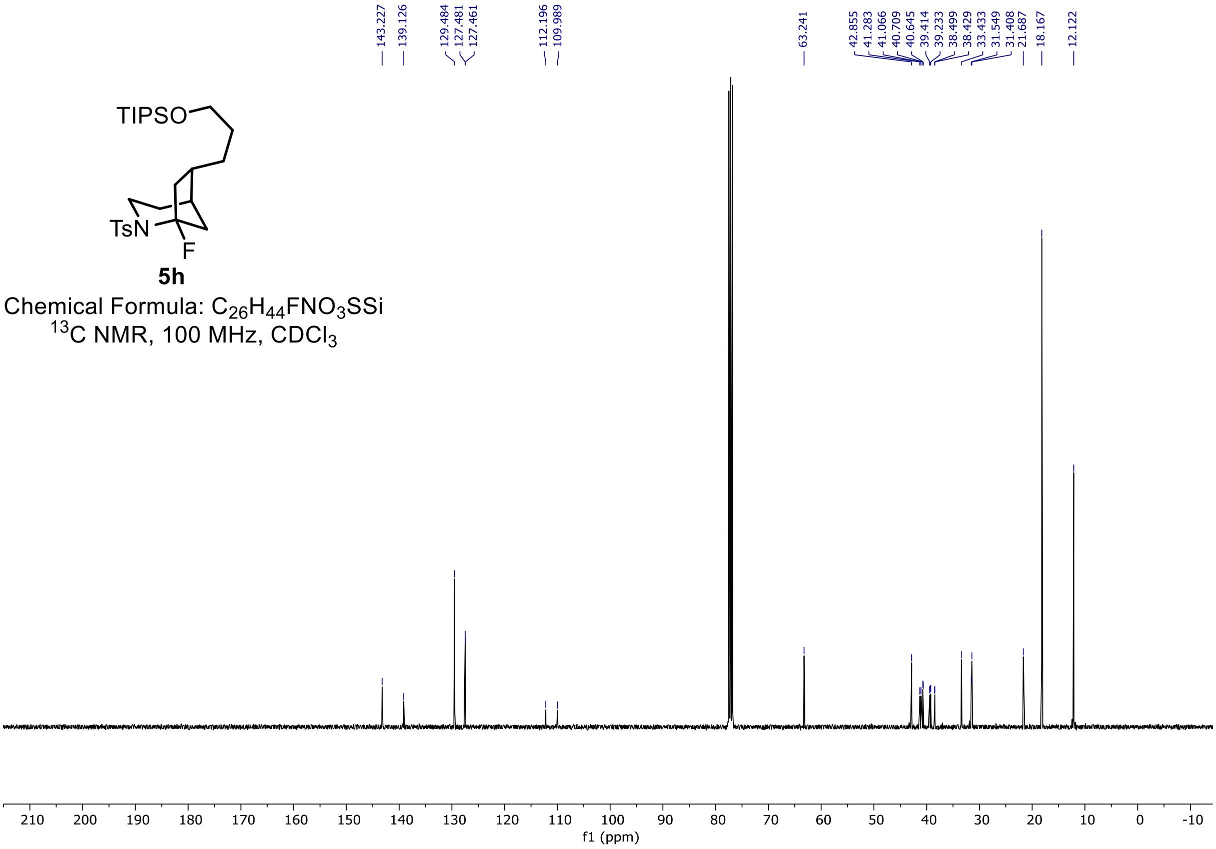

5h

Chemical Formula: $C_{26}H_{44}FNO_3SSi$

^{13}C NMR, 100 MHz, $CDCl_3$

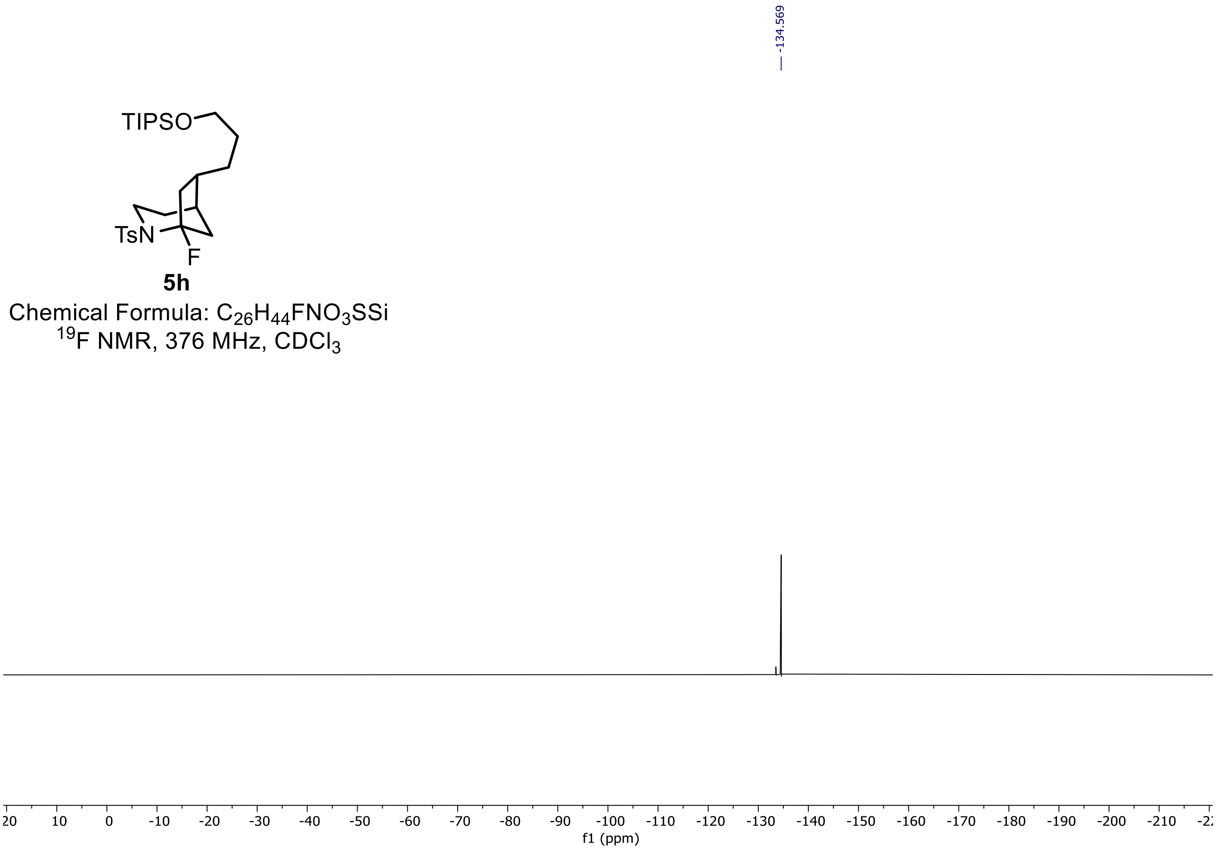

5h

Chemical Formula: $C_{26}H_{44}FNO_3SSi$

^{19}F NMR, 376 MHz, $CDCl_3$

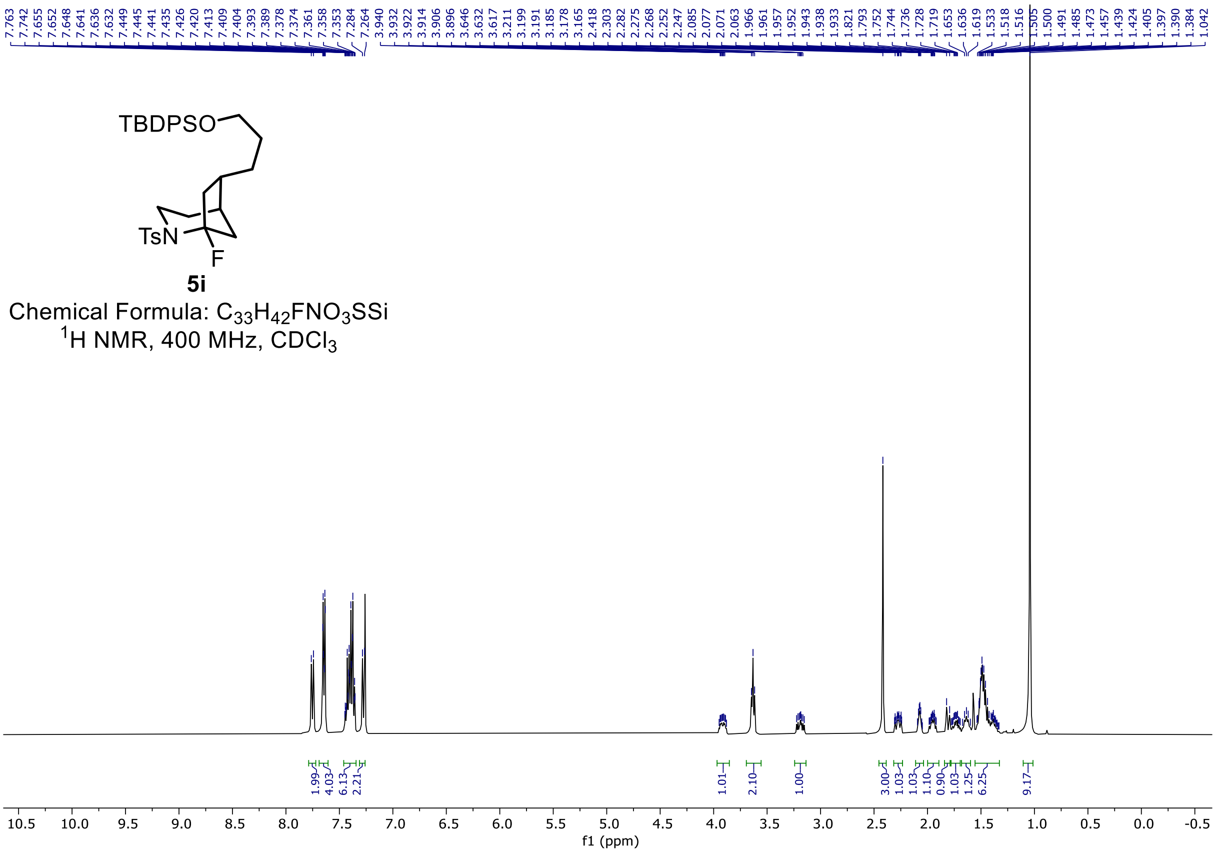
7.763 7.742 7.655 7.652 7.648 7.641 7.636 7.632 7.449 7.445 7.441 7.435 7.426 7.420 7.413 7.409 7.404 7.393 7.389 7.378 7.374 7.361 7.358 7.353 7.284 7.264 3.940 3.932 3.922 3.914 3.906 3.896 3.646 3.632 3.617 3.211 3.199 3.191 3.185 3.178 3.165 2.418 2.303 2.282 2.275 2.268 2.252 2.247 2.085 2.077 2.071 2.063 1.966 1.961 1.957 1.952 1.943 1.938 1.933 1.821 1.793 1.752 1.744 1.736 1.728 1.719 1.653 1.636 1.619 1.533 1.518 1.516 1.505 1.500 1.491 1.485 1.473 1.457 1.439 1.424 1.405 1.397 1.390 1.384 1.042
TBDPSO
TsN
F
5i
Chemical Formula: $C_{33}H_{42}FNO_3SSi$
^1H NMR, 400 MHz, $CDCl_3$
1.99 4.03 6.13 2.21 1.01 2.10 1.00 3.00 1.03 1.03 1.10 0.90 1.03 1.25 6.25 9.17
10.5 10.0 9.5 9.0 8.5 8.0 7.5 7.0 6.5 6.0 5.5 5.0 4.5 4.0 3.5 3.0 2.5 2.0 1.5 1.0 0.5 0.0 -0.5
f1 (ppm)

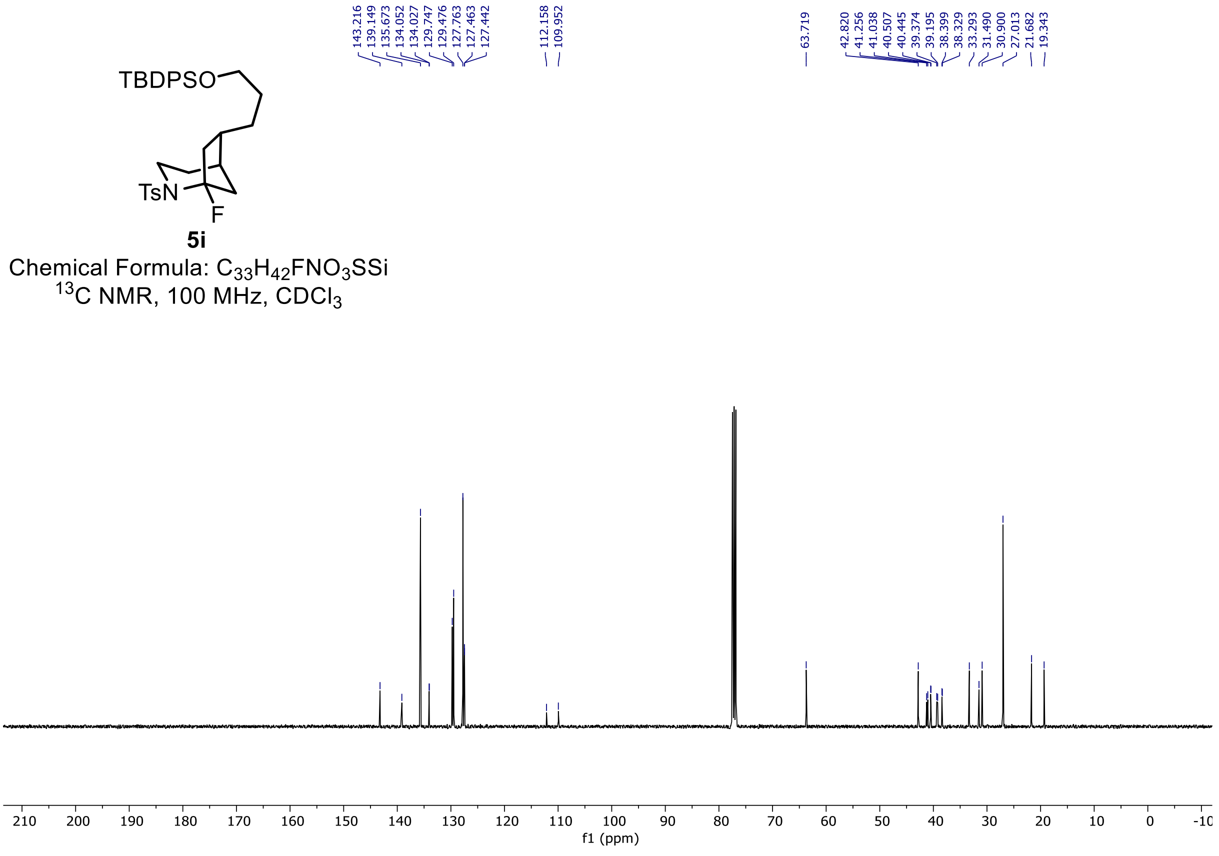
143.216 139.149 135.673 134.052 134.027 129.747 129.476 127.763 127.463 127.442 112.158 109.952 63.719 42.820 41.256 41.038 40.507 40.445 39.374 39.195 38.399 38.329 33.293 31.490 30.900 27.013 21.682 19.343
TBDPSO
TsN
F
5i
Chemical Formula: $C_{33}H_{42}FNO_3SSi$
^{13}C NMR, 100 MHz, $CDCl_3$
210 200 190 180 170 160 150 140 130 120 110 100 90 80 70 60 50 40 30 20 10 0 -10
f1 (ppm)

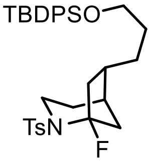

5i

Chemical Formula: $C_{33}H_{42}FNO_3SSi$

^{19}F NMR, 376 MHz, $CDCl_3$

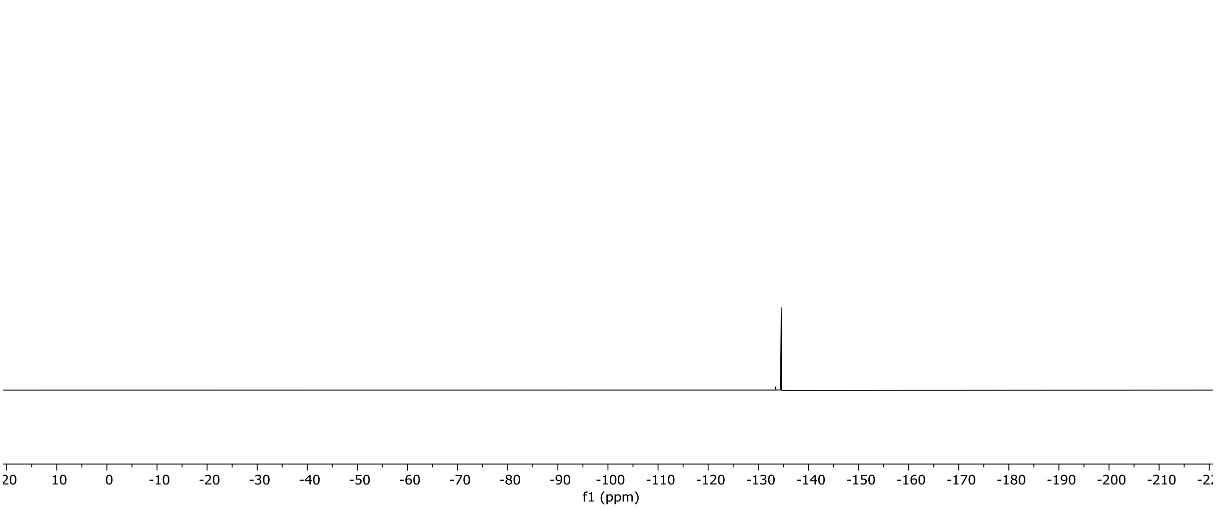

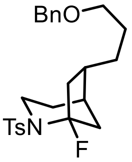

5j

Chemical Formula: $C_{24}H_{30}FNO_3S$

^{1}H NMR, 400 MHz, $CDCl_3$

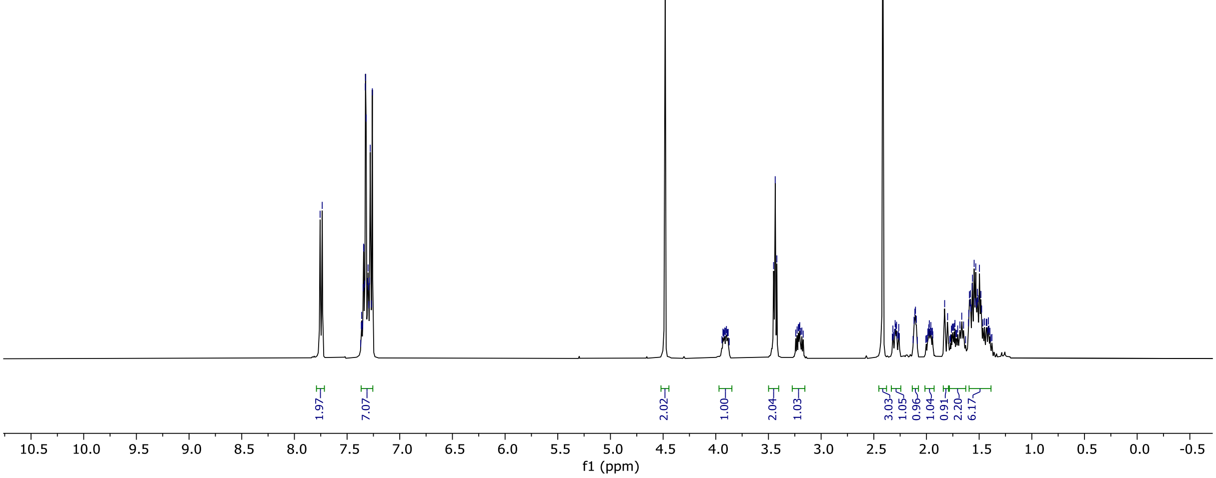

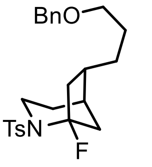

5j

Chemical Formula: $C_{24}H_{30}FNO_3S$

^{13}C NMR, 100 MHz, $CDCl_3$

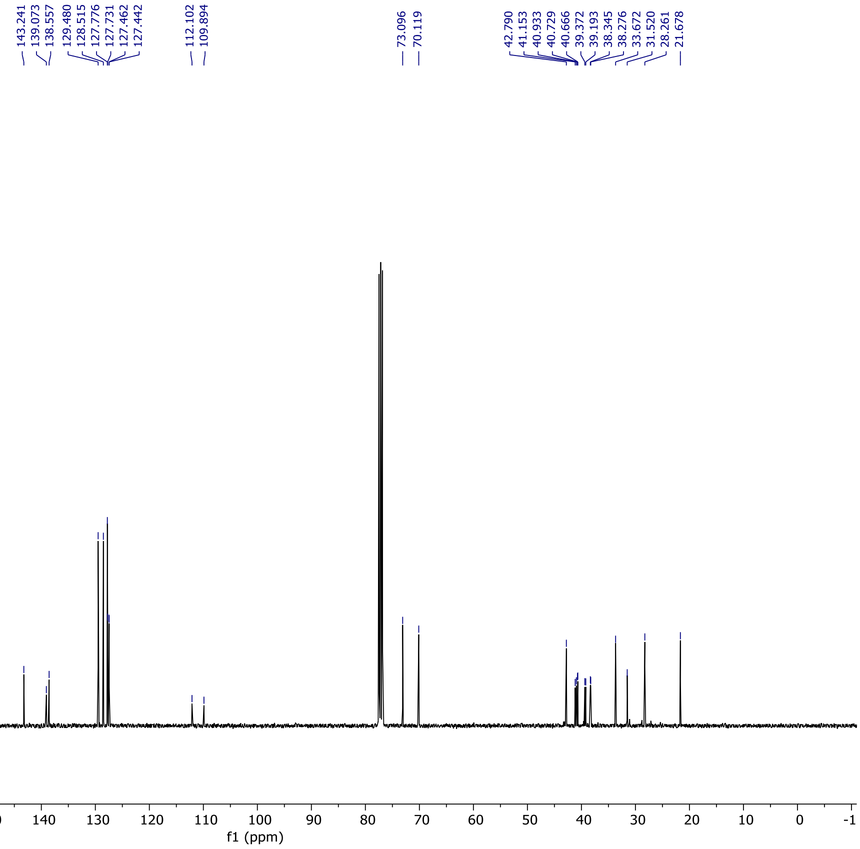

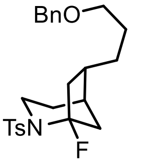

5j

Chemical Formula: $C_{24}H_{30}FNO_3S$

^{19}F NMR, 376 MHz, $CDCl_3$

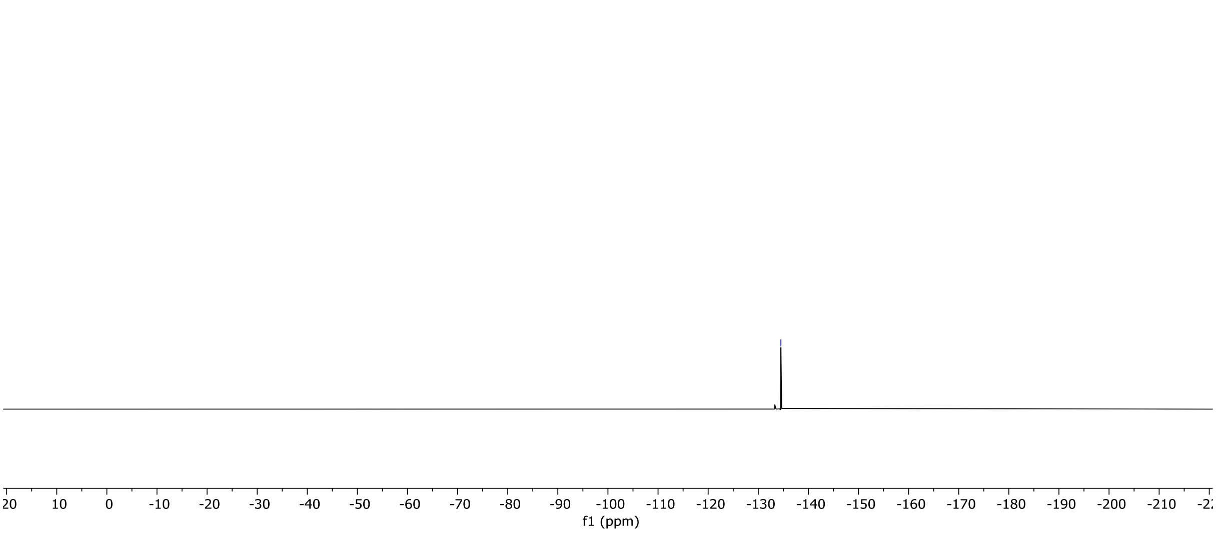

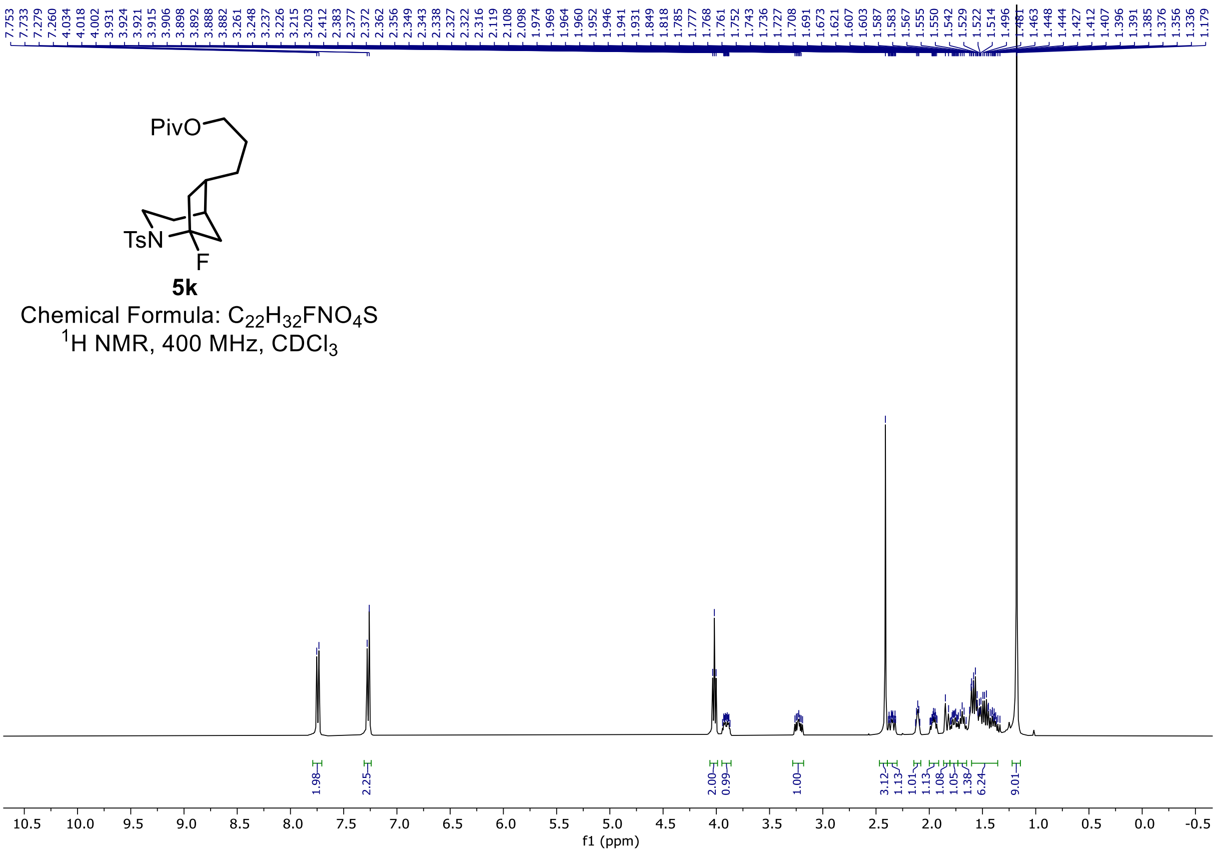
7.753 7.733 7.279 7.260 4.034 4.018 4.002 3.931 3.924 3.921 3.915 3.906 3.898 3.892 3.888 3.882 3.261 3.248 3.237 3.226 3.215 3.203 2.412 2.383 2.377 2.372 2.362 2.356 2.349 2.343 2.338 2.327 2.322 2.316 2.119 2.108 2.098 1.974 1.969 1.964 1.960 1.952 1.946 1.941 1.931 1.849 1.818 1.785 1.777 1.768 1.761 1.752 1.743 1.736 1.727 1.708 1.691 1.673 1.621 1.607 1.603 1.587 1.583 1.567 1.555 1.550 1.542 1.529 1.522 1.514 1.496 1.481 1.463 1.448 1.444 1.427 1.412 1.407 1.396 1.391 1.385 1.376 1.356 1.336 1.179
PivO
TsN
F
5k
Chemical Formula: $C_{22}H_{32}FNO_4S$
^{1}H NMR, 400 MHz, $CDCl_3$
1.98 2.25 2.00 0.99 1.00 3.12 1.13 1.01 1.13 1.08 1.05 1.38 6.24 9.01
f1 (ppm)

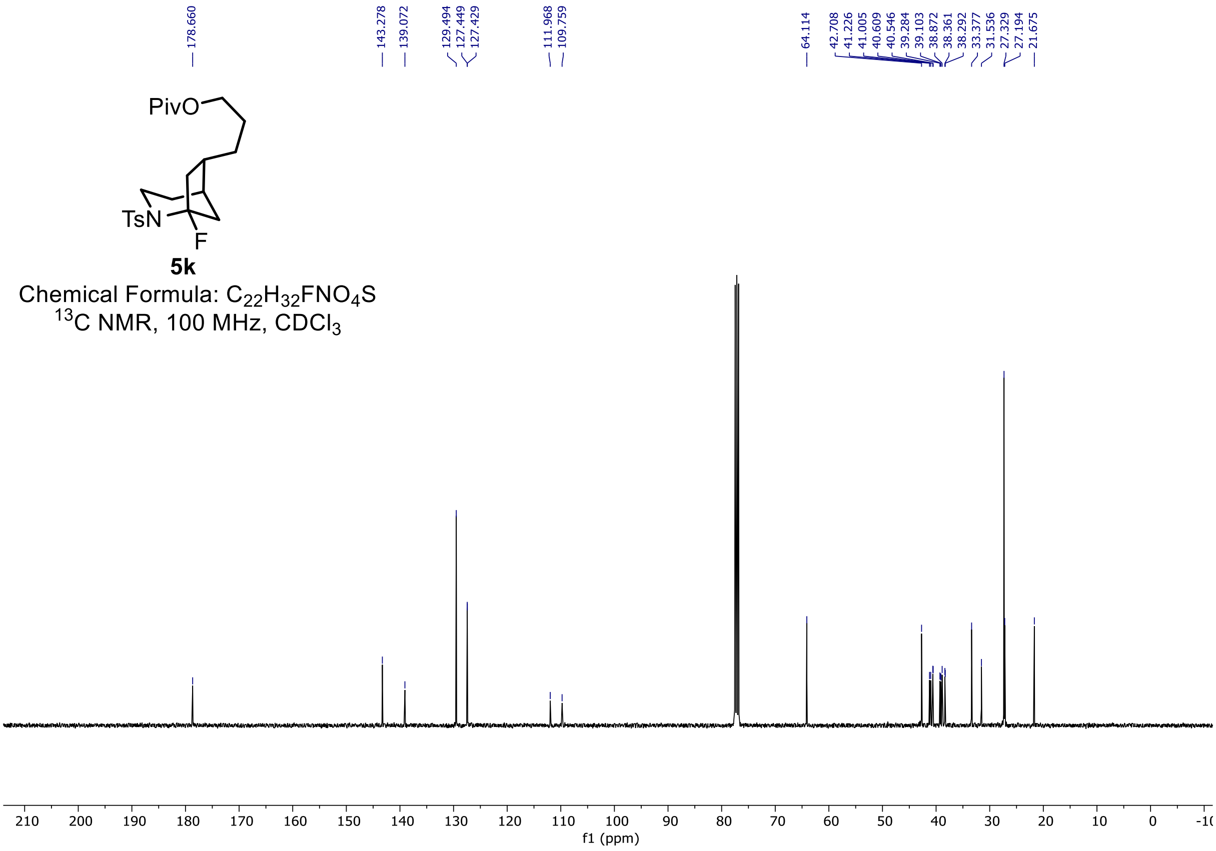
178.660 143.278 139.072 129.494 127.449 127.429 111.968 109.759 64.114 42.708 41.226 41.005 40.609 40.546 39.284 39.103 38.872 38.361 38.292 33.377 31.536 27.329 27.194 21.675
PivO
TsN
F
5k
Chemical Formula: $C_{22}H_{32}FNO_4S$
^{13}C NMR, 100 MHz, $CDCl_3$
f1 (ppm)

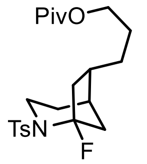

5k

Chemical Formula: $C_{22}H_{32}FNO_4S$

^{19}F NMR, 376 MHz, $CDCl_3$

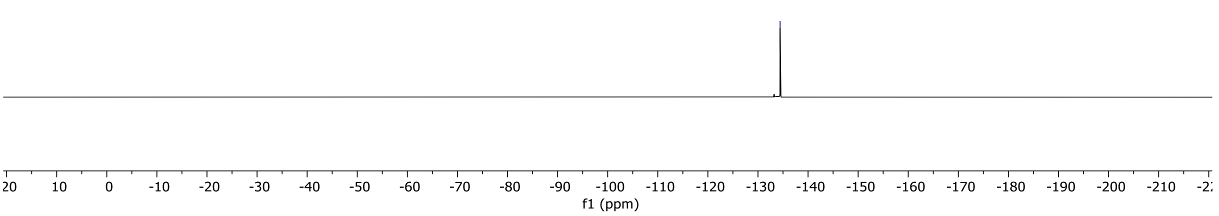

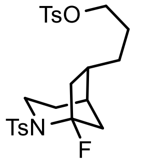

5l

Chemical Formula: $C_{24}H_{30}FNO_5S_2$

^{1}H NMR, 400 MHz, $CDCl_3$

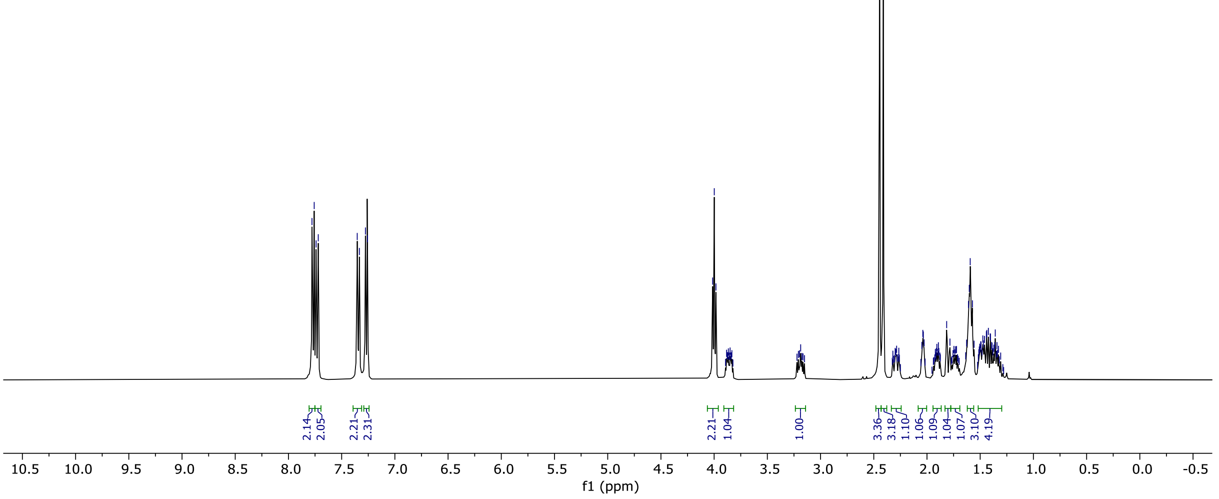

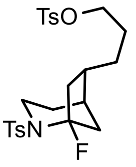

5l

Chemical Formula: $C_{24}H_{30}FNO_5S_2$

^{13}C NMR, 100 MHz, $CDCl_3$

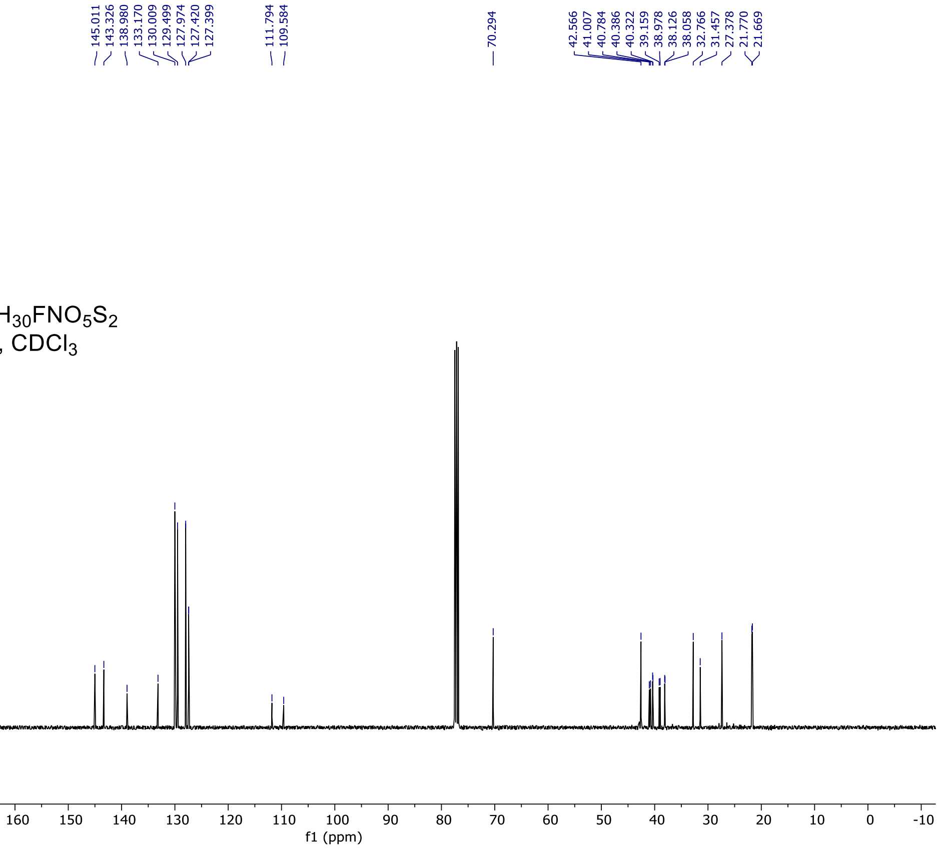

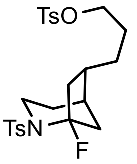

5l

Chemical Formula: $C_{24}H_{30}FNO_5S_2$

^{19}F NMR, 376 MHz, $CDCl_3$

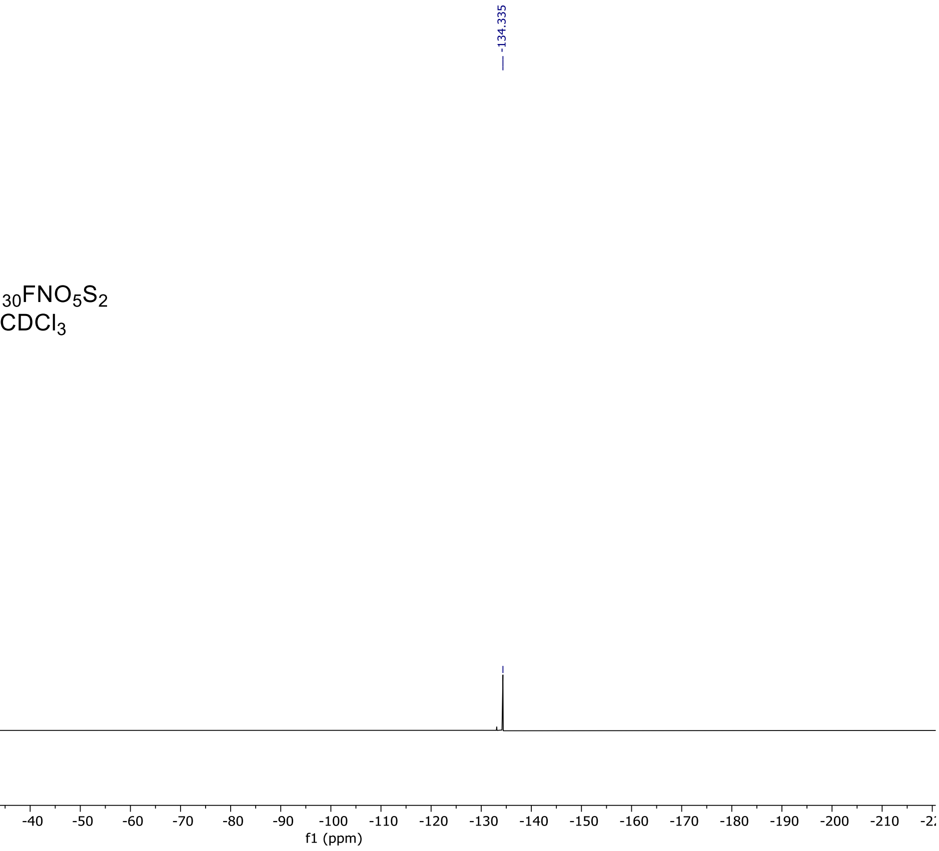

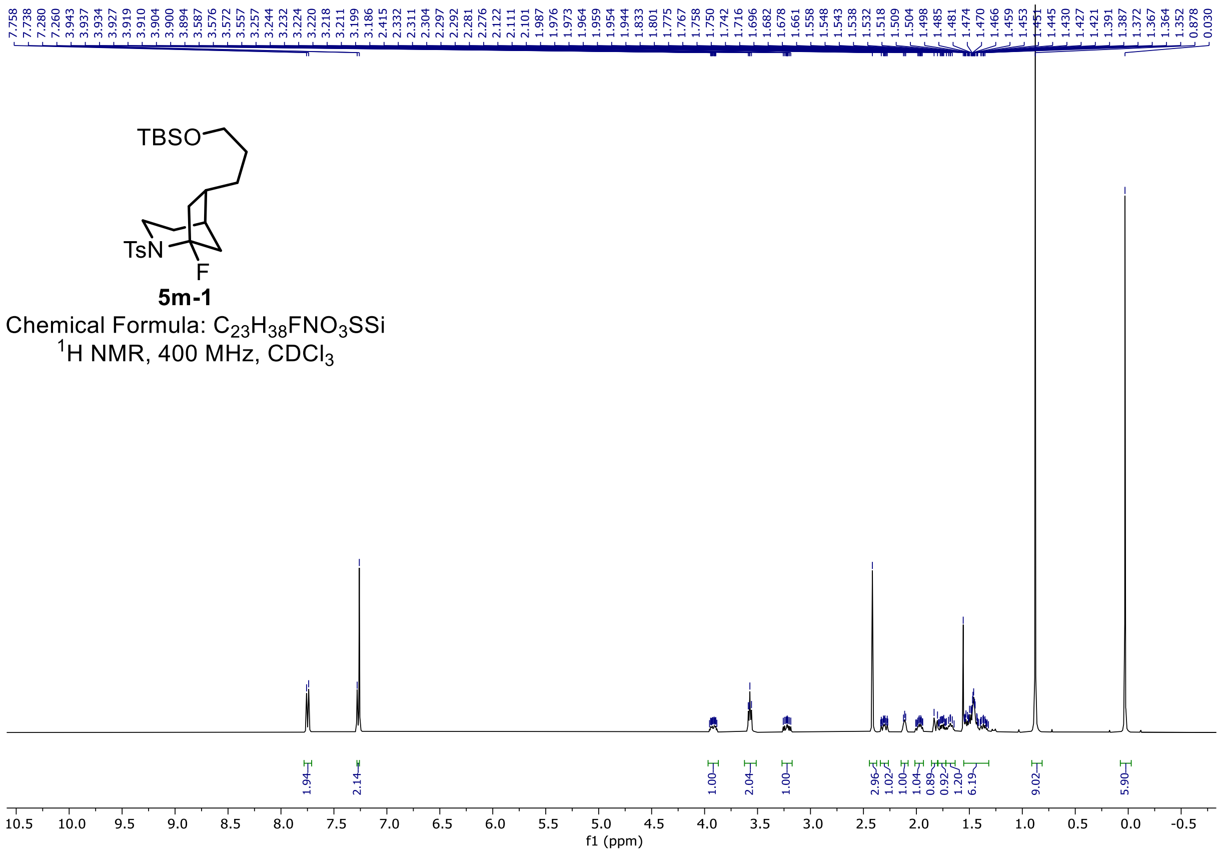

5m-1

Chemical Formula: $C_{23}H_{38}FNO_3SSi$

^{1}H NMR, 400 MHz, $CDCl_3$

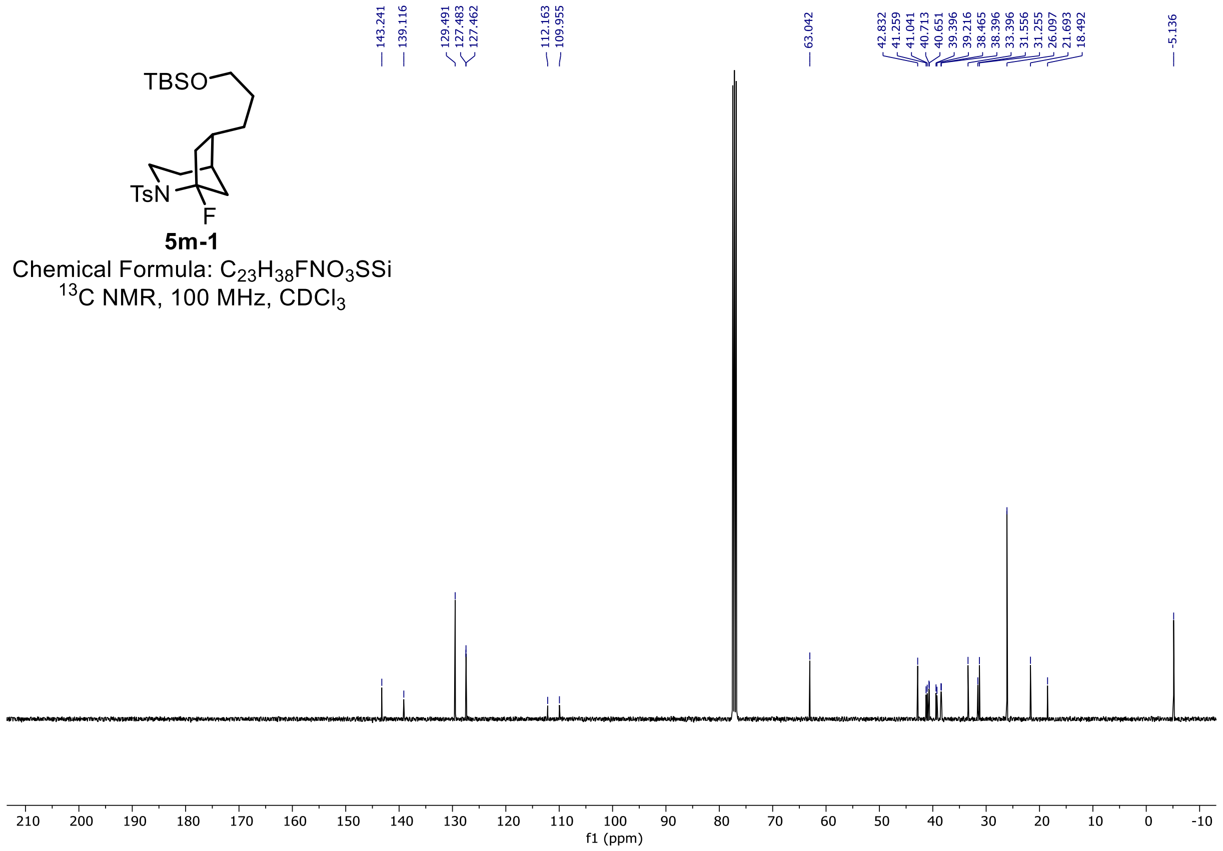

5m-1

Chemical Formula: $C_{23}H_{38}FNO_3SSi$

^{13}C NMR, 100 MHz, $CDCl_3$

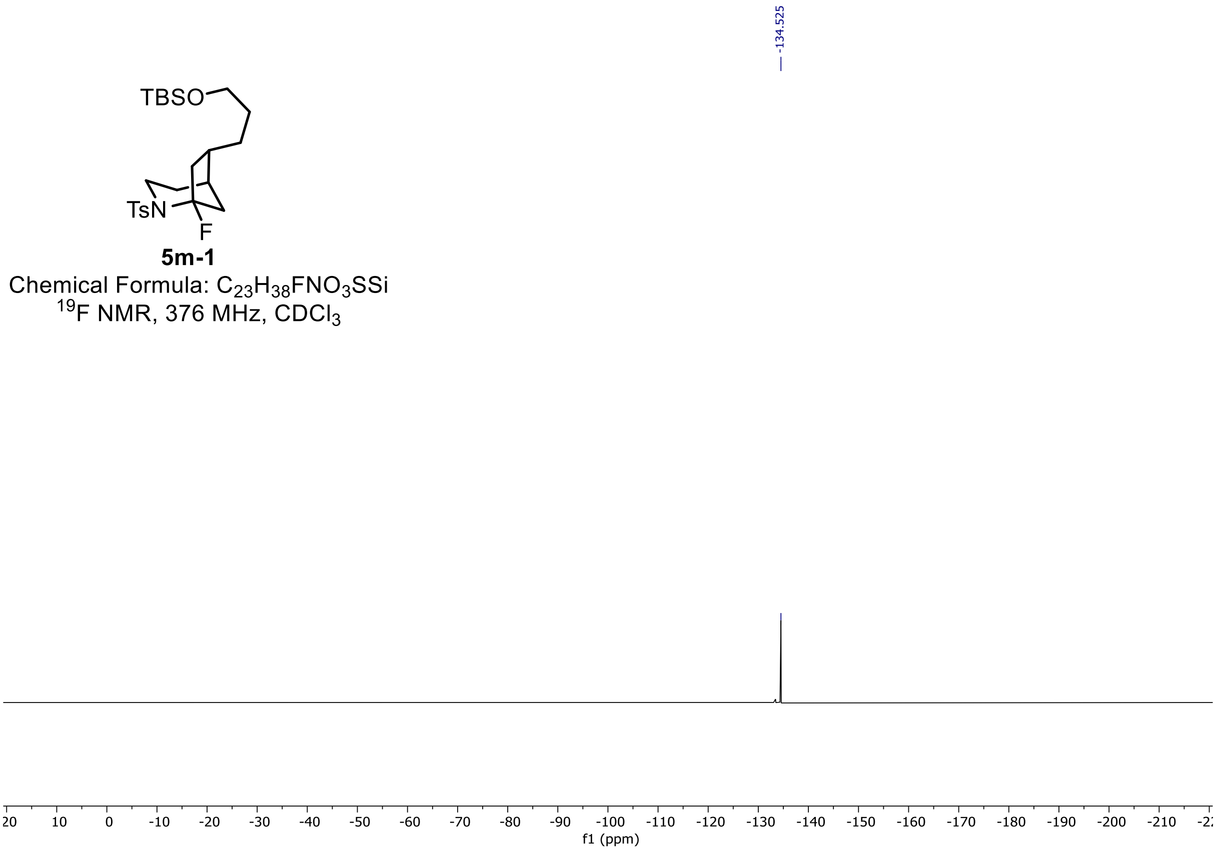

5m-1

Chemical Formula: $C_{23}H_{38}FNO_3SSi$

^{19}F NMR, 376 MHz, $CDCl_3$

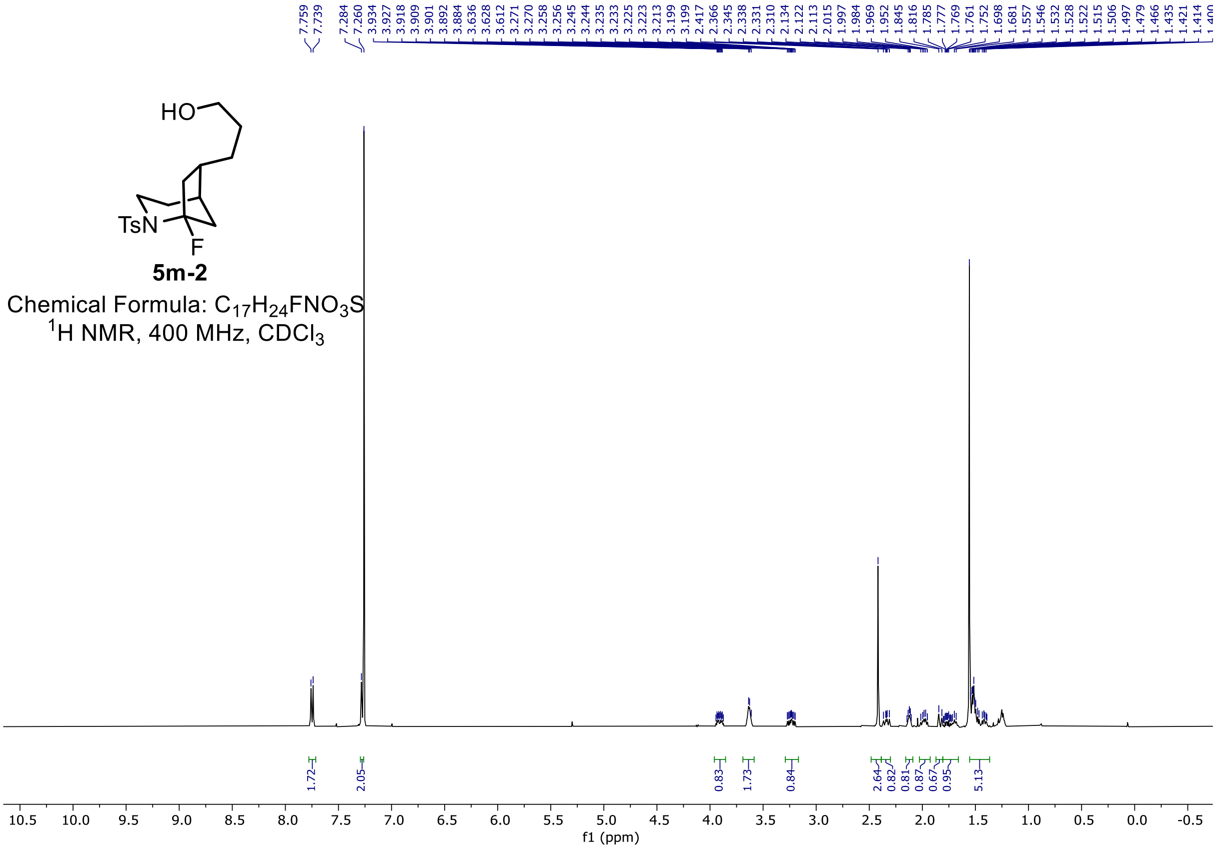

5m-2

Chemical Formula: $C_{17}H_{24}FNO_3S$

^{1}H NMR, 400 MHz, $CDCl_3$

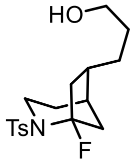

5m-2

Chemical Formula: $C_{17}H_{24}FNO_3S$

^{13}C NMR, 200 MHz, $CDCl_3$

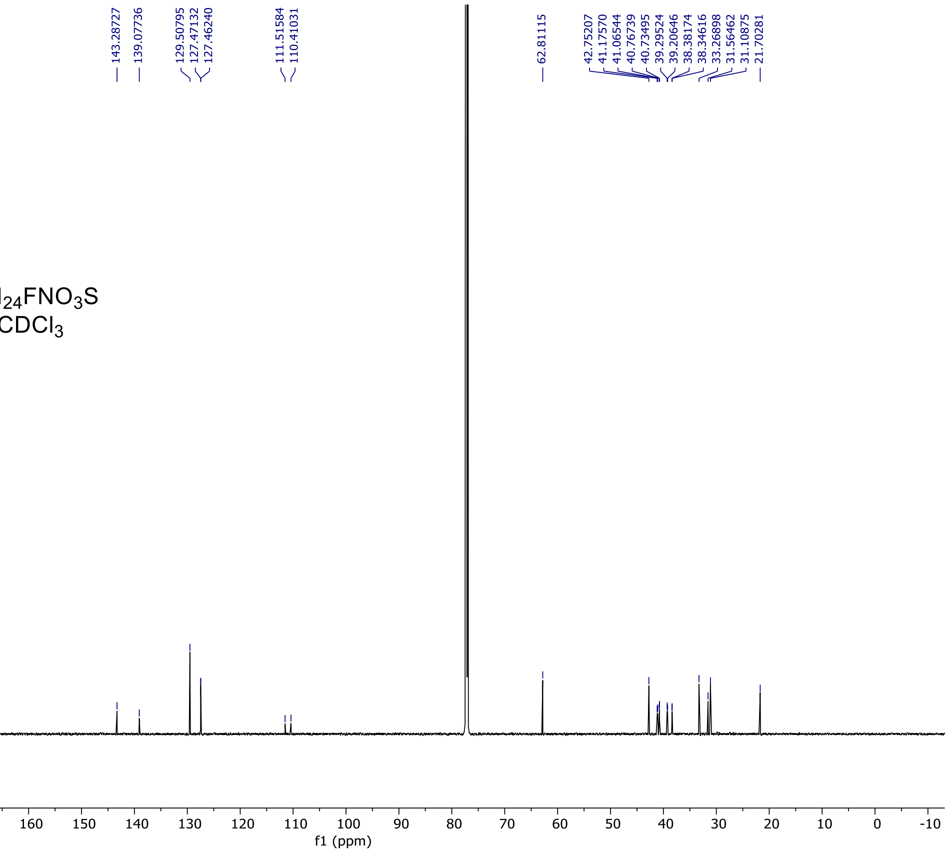

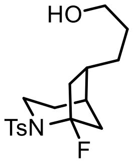

5m-2

Chemical Formula: $C_{17}H_{24}FNO_3S$

^{19}F NMR, 376 MHz, $CDCl_3$

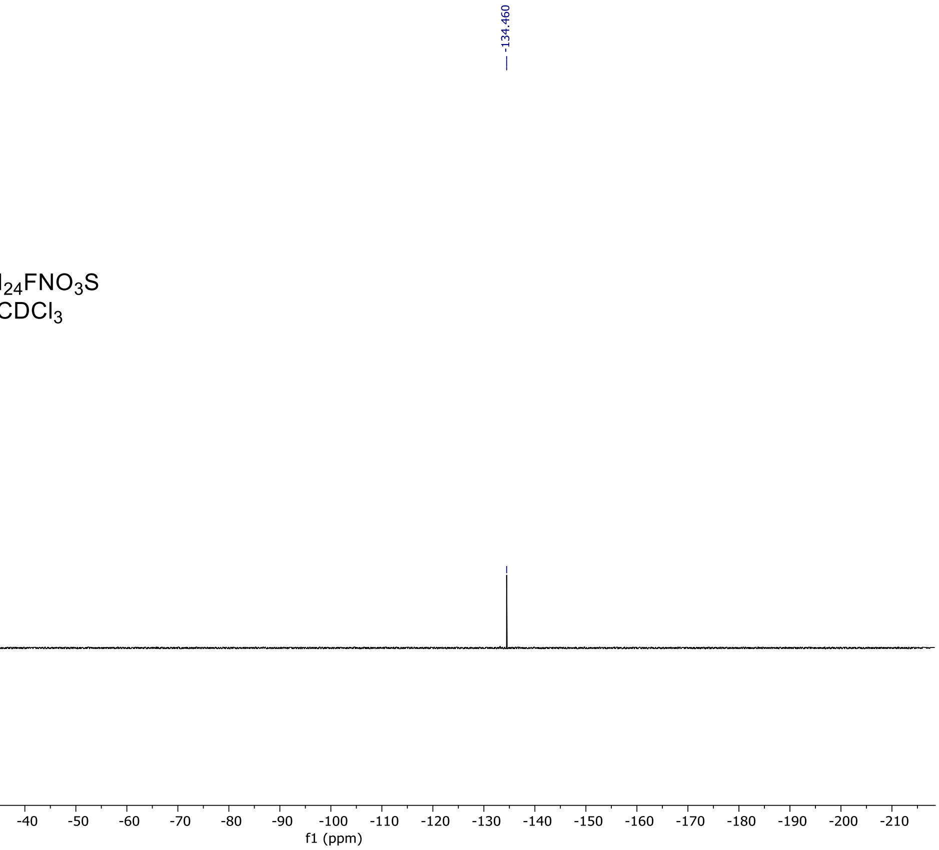

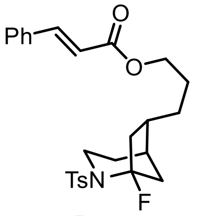

5n

Chemical Formula: $C_{26}H_{30}FNO_4S$

^{1}H NMR, 400 MHz, $CDCl_3$

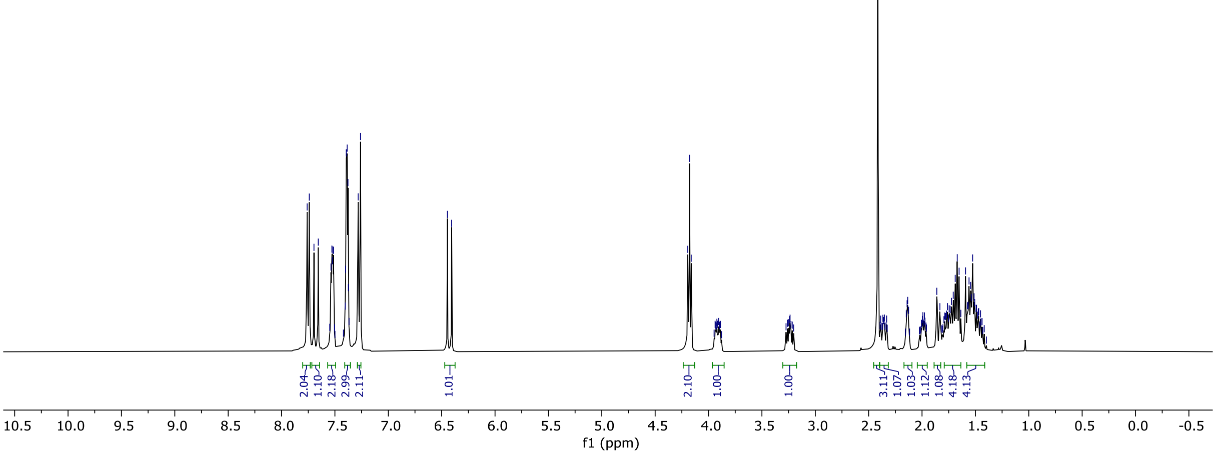

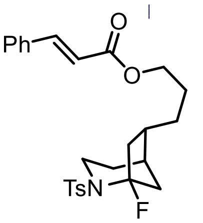

5n

Chemical Formula: $C_{26}H_{30}FNO_4S$

^{13}C NMR, 100 MHz, $CDCl_3$

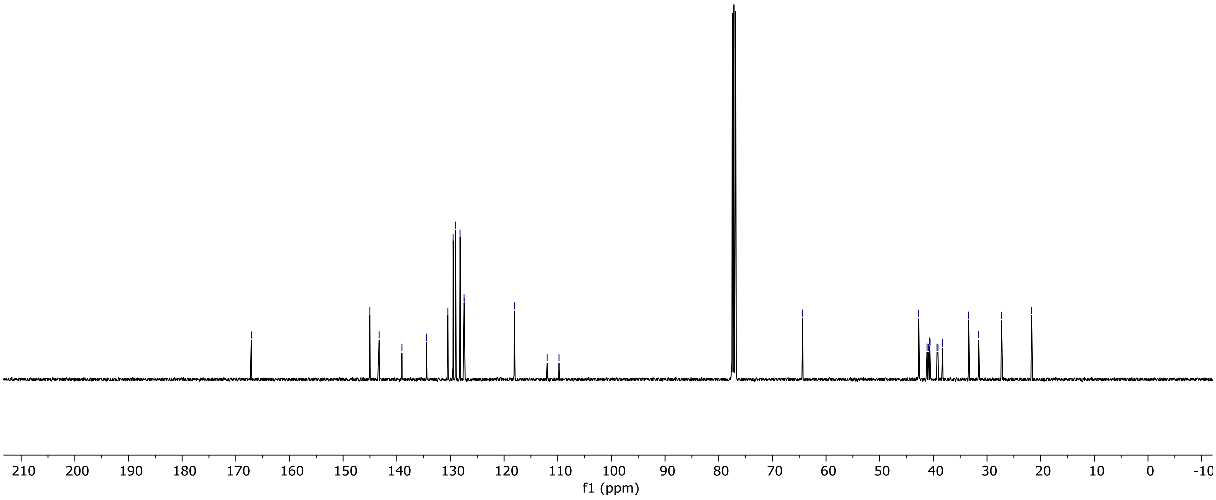

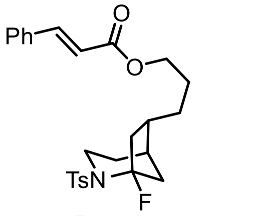

5n

Chemical Formula: $C_{26}H_{30}FNO_4S$

^{19}F NMR, 376 MHz, $CDCl_3$

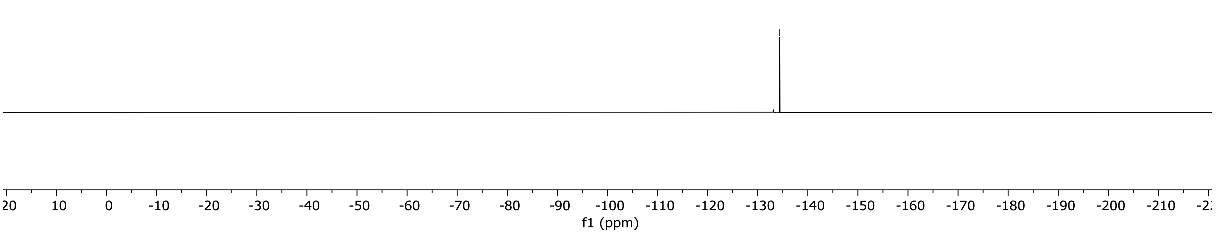

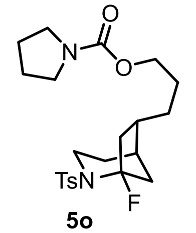

5o

Chemical Formula: $C_{22}H_{31}FN_2O_4S$

^{1}H NMR, 400 MHz, $CDCl_3$

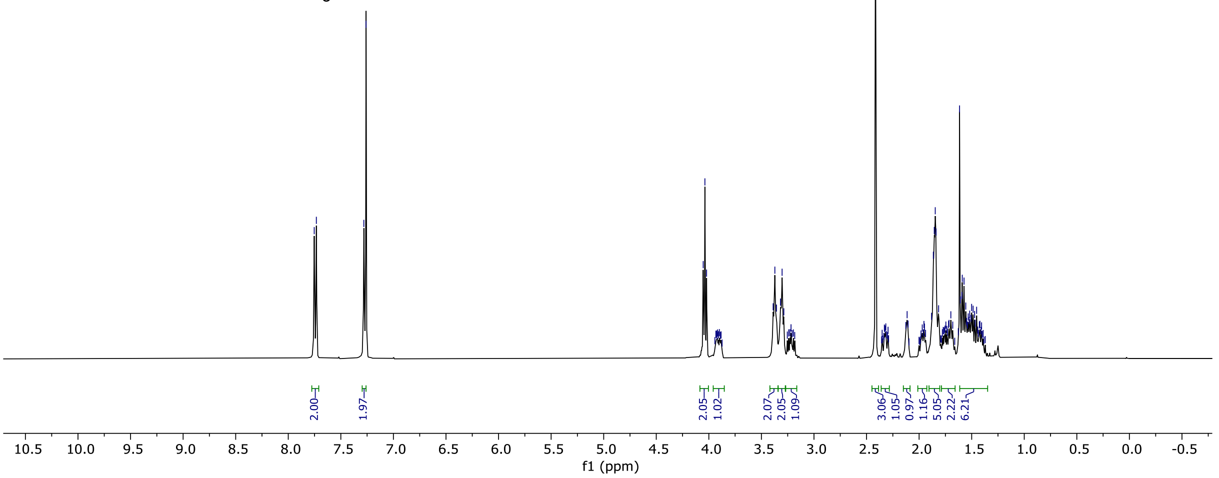

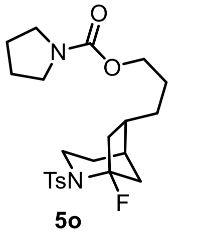

5o

Chemical Formula: $C_{22}H_{31}FN_2O_4S$

^{13}C NMR, 100 MHz, $CDCl_3$

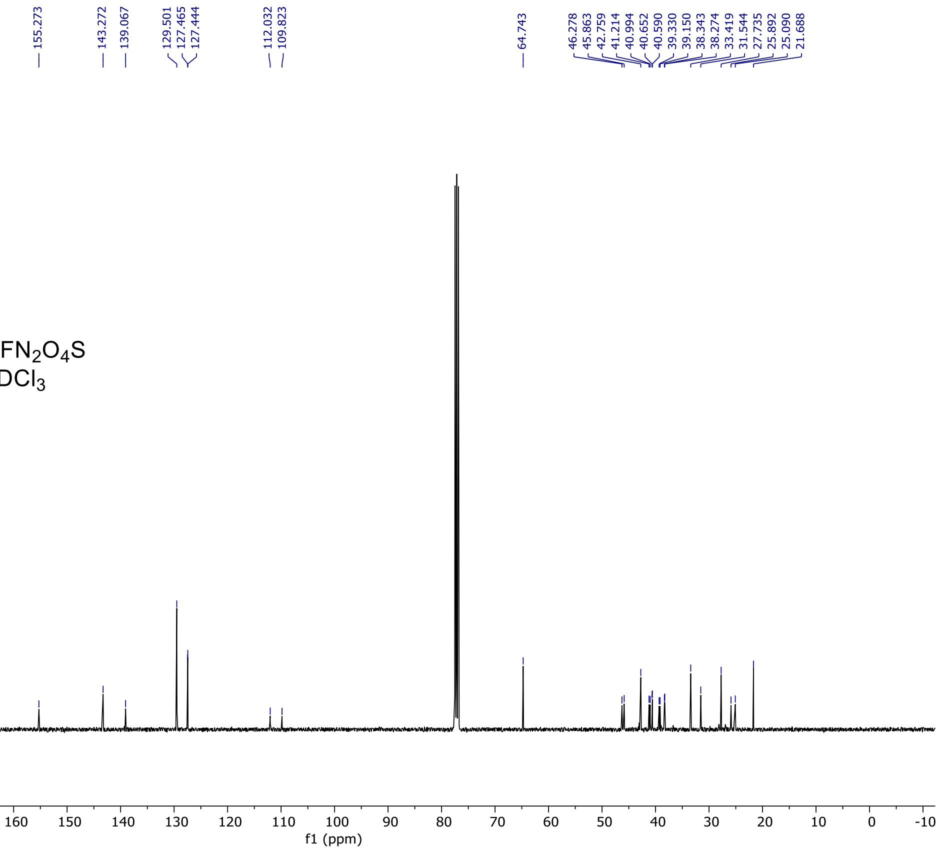

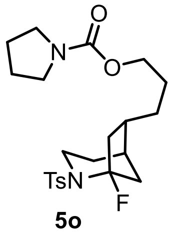

5o

Chemical Formula: $C_{22}H_{31}FN_2O_4S$

^{19}F NMR, 376 MHz, $CDCl_3$

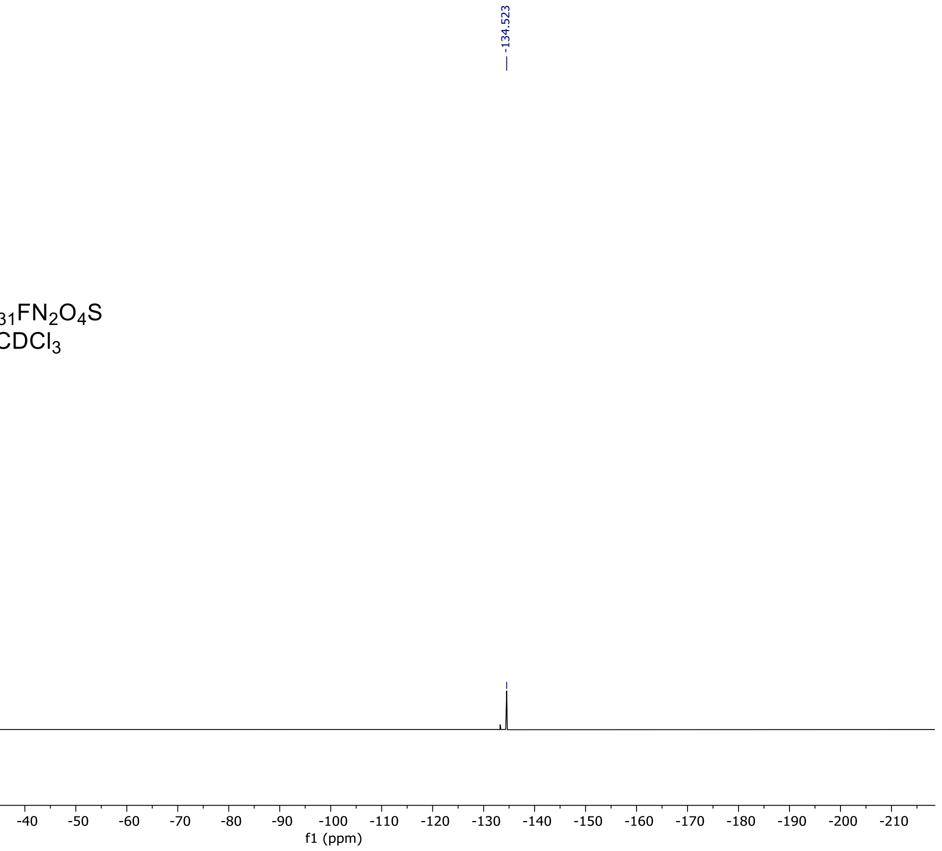

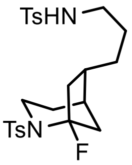

5p

Chemical Formula: $C_{24}H_{31}FN_2O_4S_2$

^1H NMR, 400 MHz, $CDCl_3$

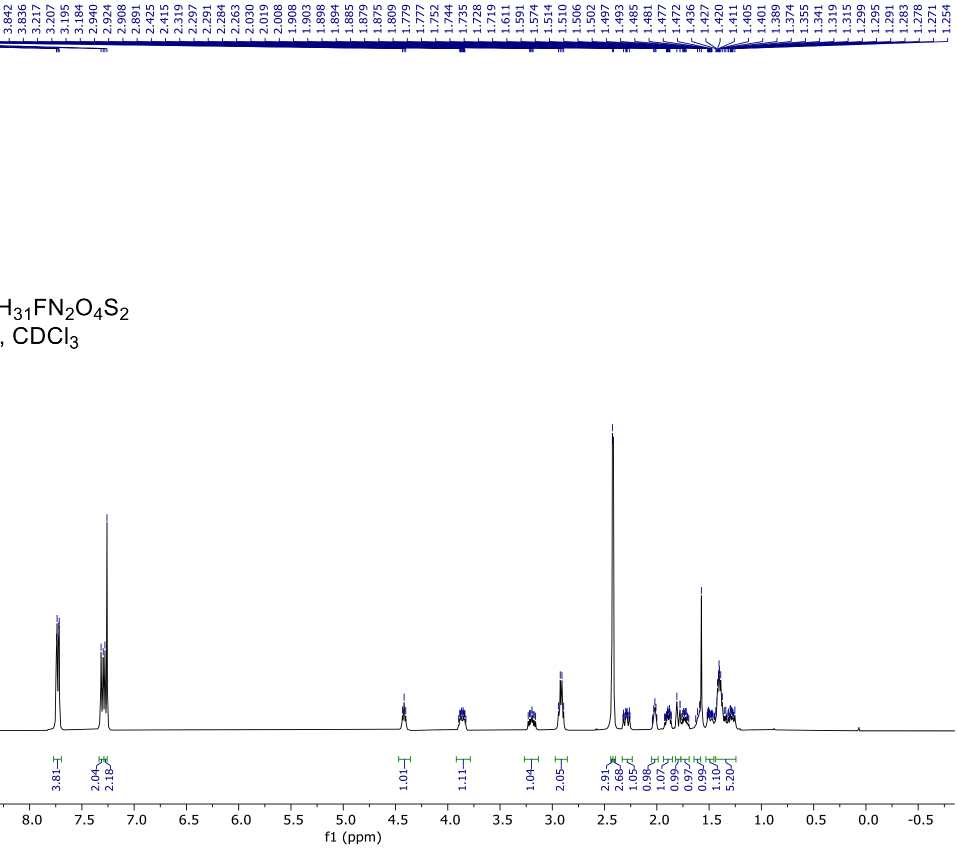

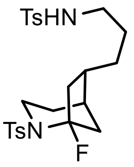

5p

Chemical Formula: $C_{24}H_{31}FN_2O_4S_2$

^{13}C NMR, 100 MHz, $CDCl_3$

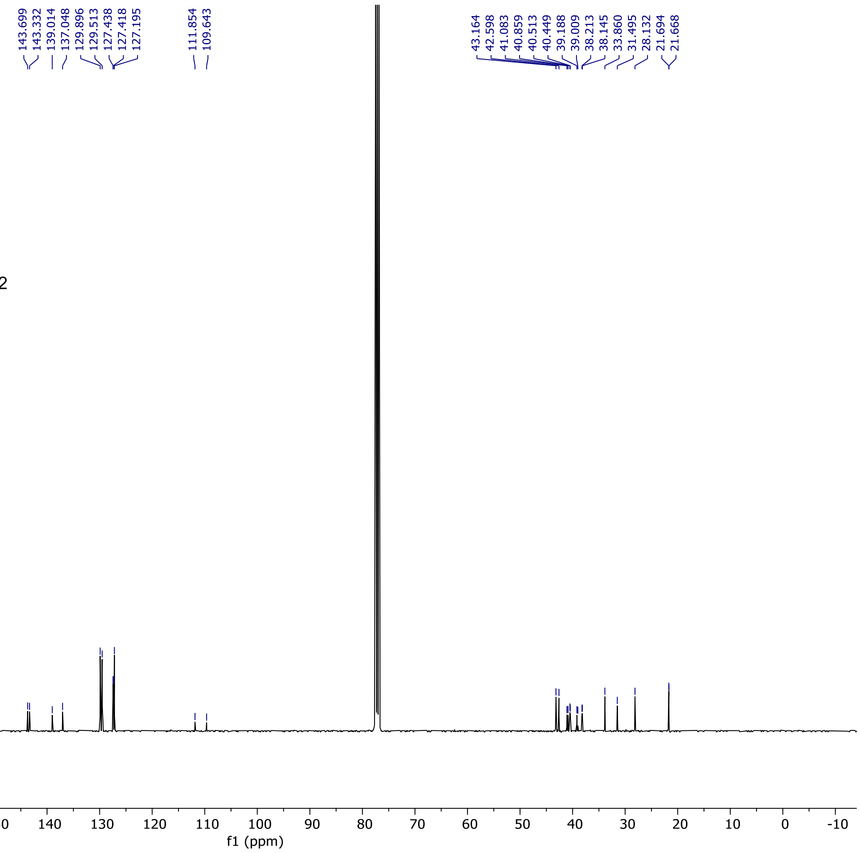

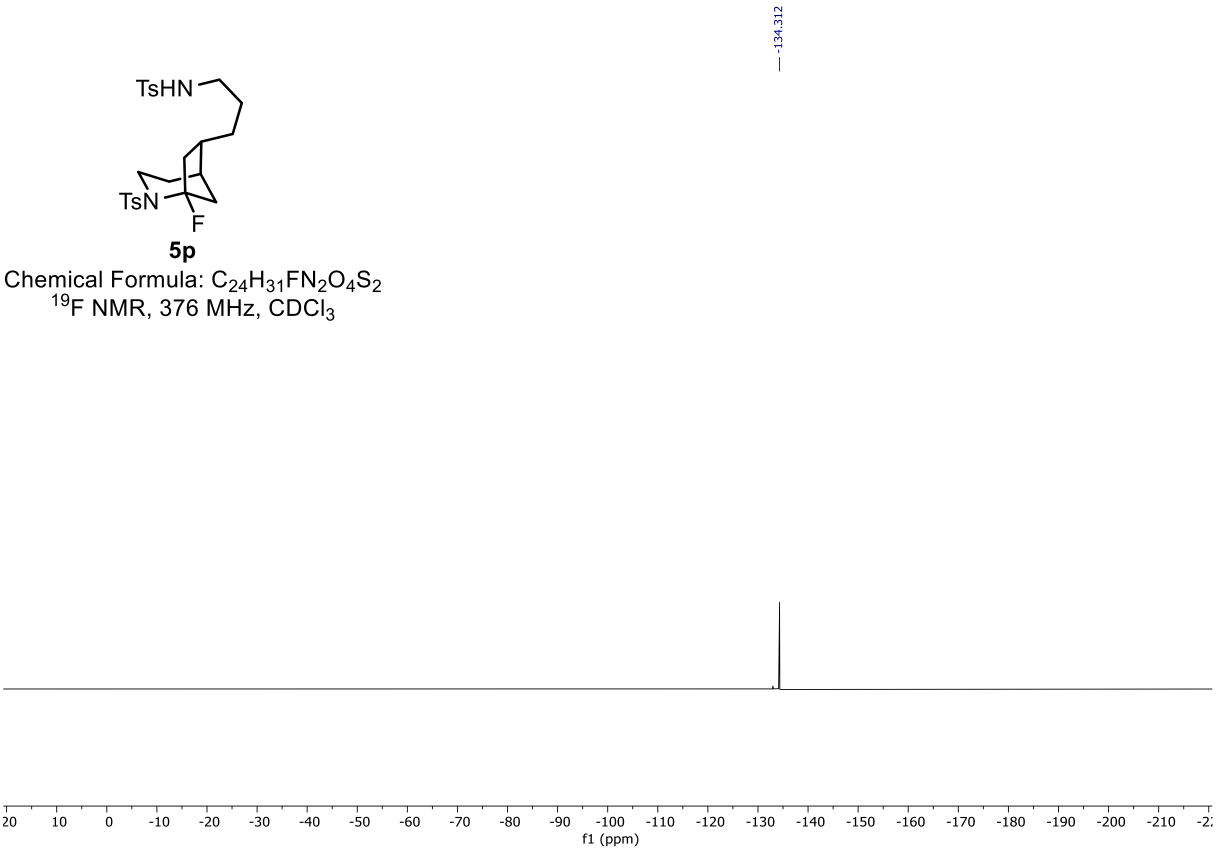

5p
Chemical Formula: $C_{24}H_{31}FN_2O_4S_2$
^{19}F NMR, 376 MHz, $CDCl_3$

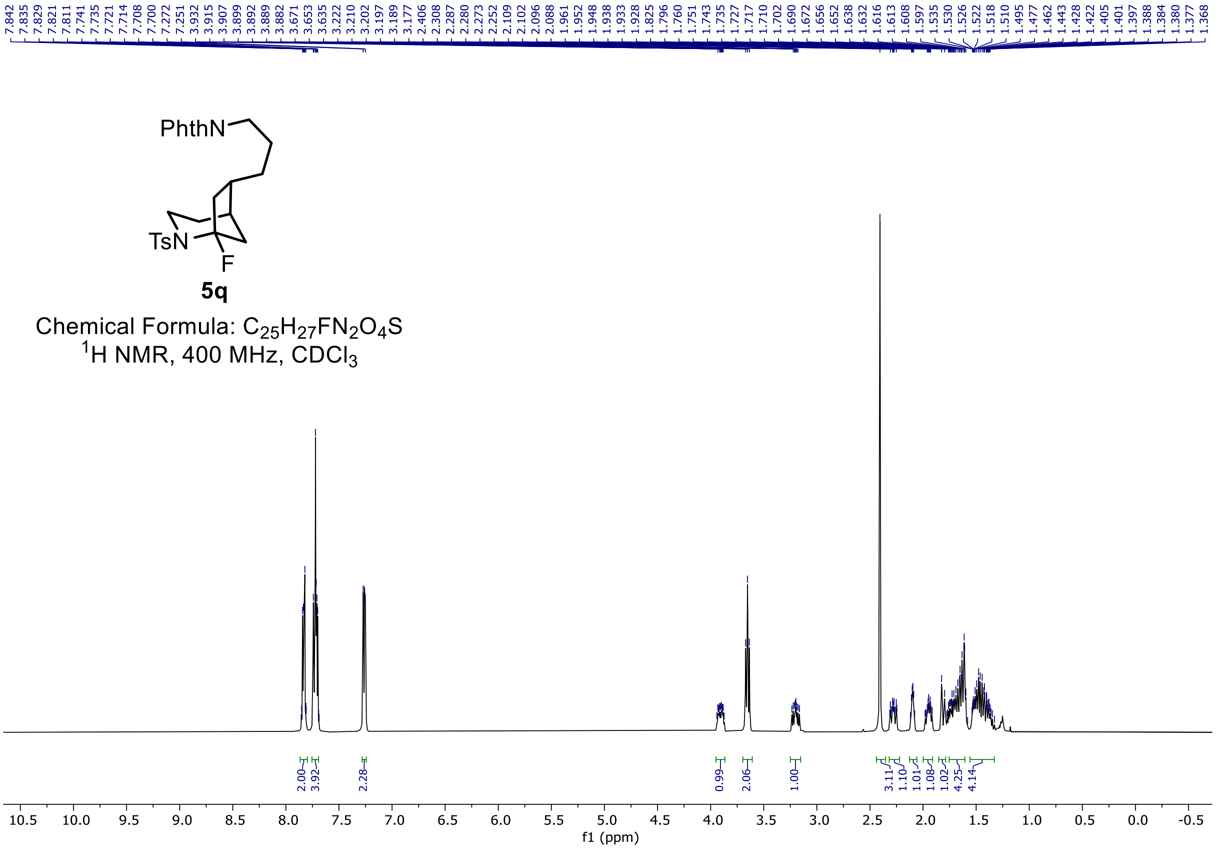

5q
Chemical Formula: $C_{25}H_{27}FN_2O_4S$
^{1}H NMR, 400 MHz, $CDCl_3$

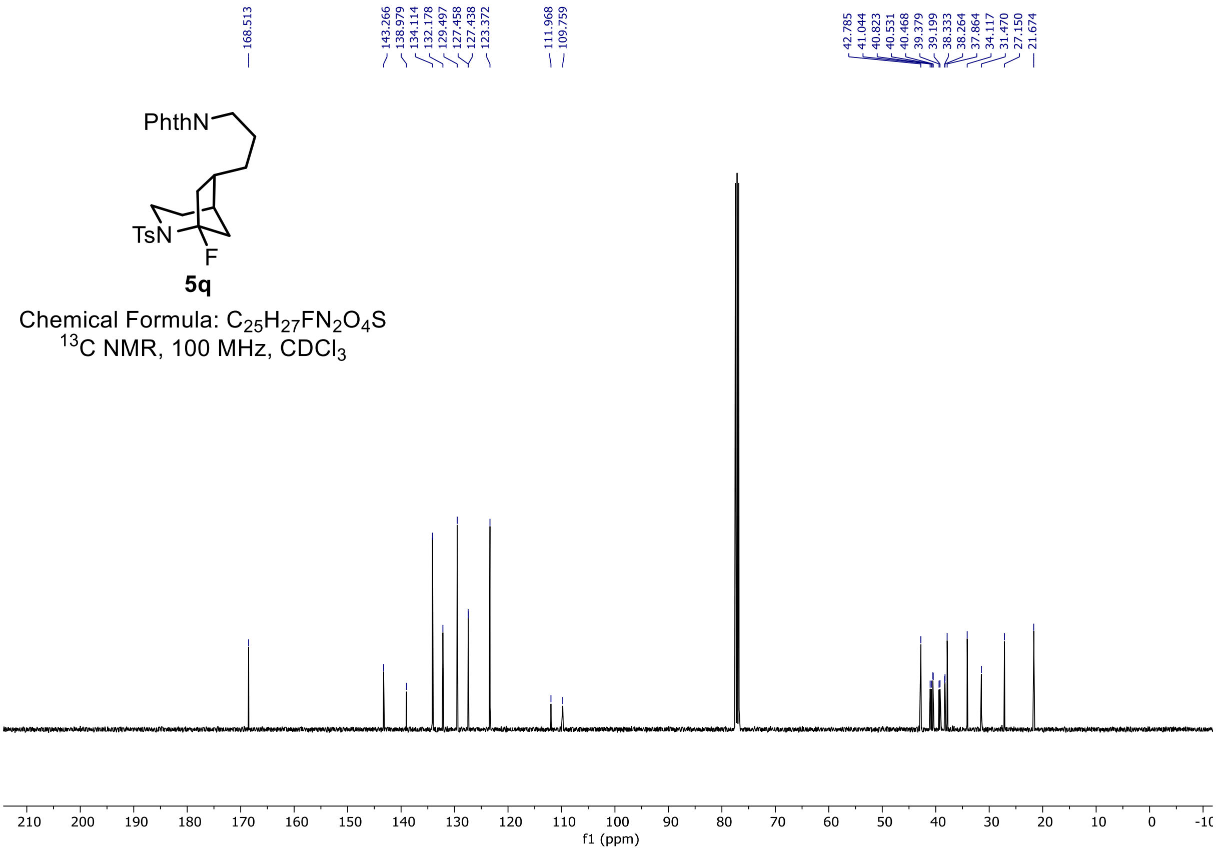

5q

Chemical Formula: $C_{25}H_{27}FN_2O_4S$
^{13}C NMR, 100 MHz, $CDCl_3$

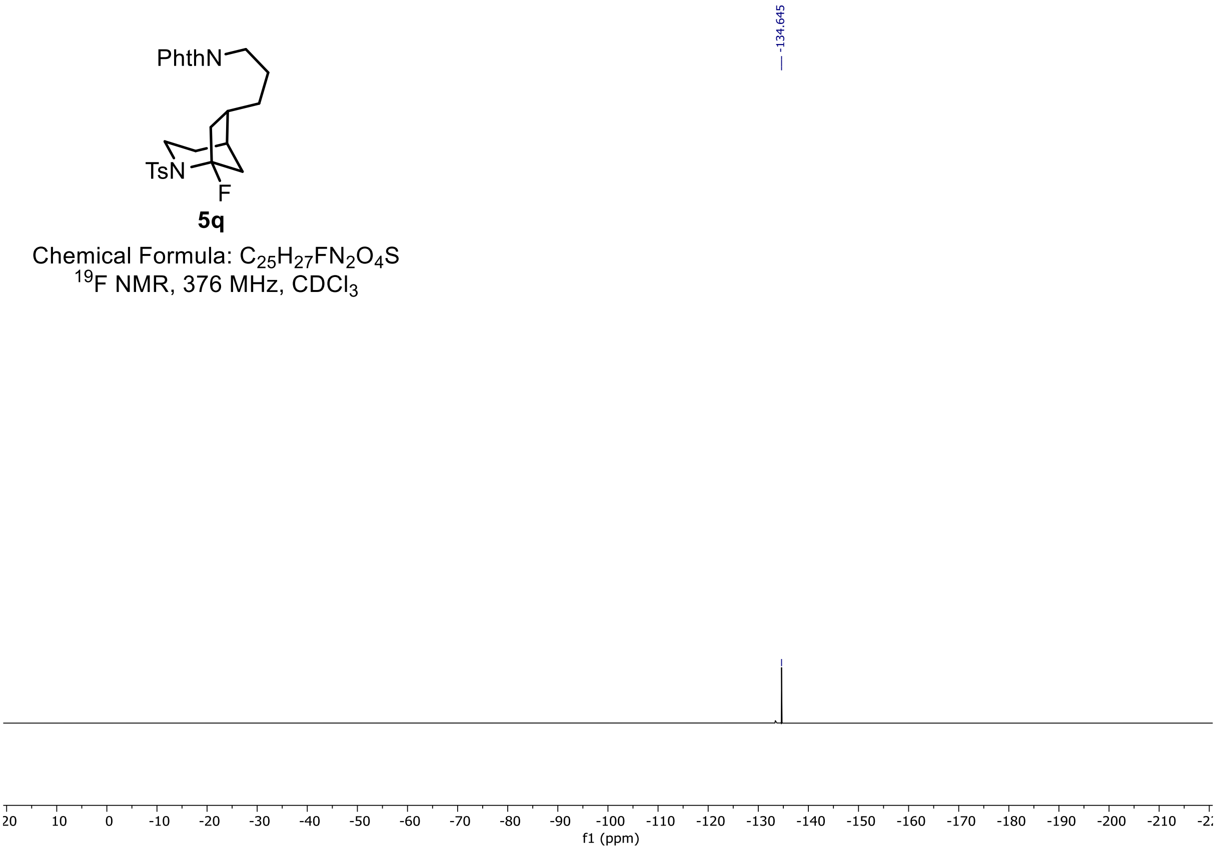

5q

Chemical Formula: $C_{25}H_{27}FN_2O_4S$
^{19}F NMR, 376 MHz, $CDCl_3$

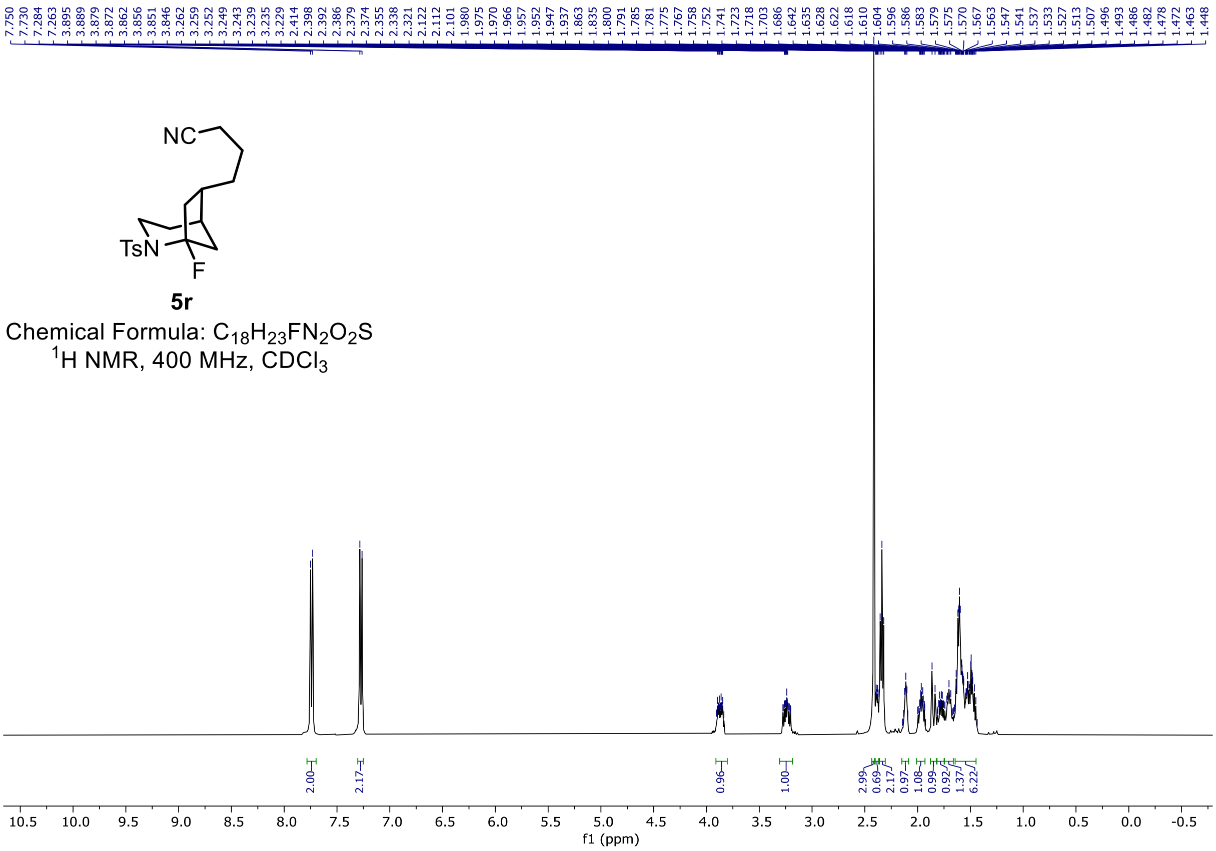
7.750 7.730 7.284 7.263 3.895 3.889 3.879 3.872 3.862 3.856 3.851 3.846 3.262 3.259 3.252 3.249 3.243 3.239 3.235 3.229 2.414 2.398 2.392 2.386 2.379 2.374 2.355 2.338 2.321 2.122 2.112 2.101 1.980 1.975 1.970 1.966 1.957 1.952 1.947 1.937 1.863 1.835 1.800 1.791 1.785 1.781 1.775 1.767 1.758 1.752 1.741 1.723 1.718 1.703 1.686 1.642 1.635 1.628 1.622 1.618 1.610 1.604 1.596 1.586 1.583 1.579 1.575 1.570 1.567 1.563 1.547 1.541 1.537 1.533 1.527 1.513 1.507 1.496 1.493 1.486 1.482 1.478 1.472 1.463 1.448
NC
TsN
F
5r
Chemical Formula: C18H23FN2O2S
1H NMR, 400 MHz, CDCl3
2.00
2.17
0.96
1.00
2.99
0.69
2.17
0.97
1.08
0.99
0.92
1.37
6.22
10.5 10.0 9.5 9.0 8.5 8.0 7.5 7.0 6.5 6.0 5.5 5.0 4.5 4.0 3.5 3.0 2.5 2.0 1.5 1.0 0.5 0.0 -0.5
f1 (ppm)

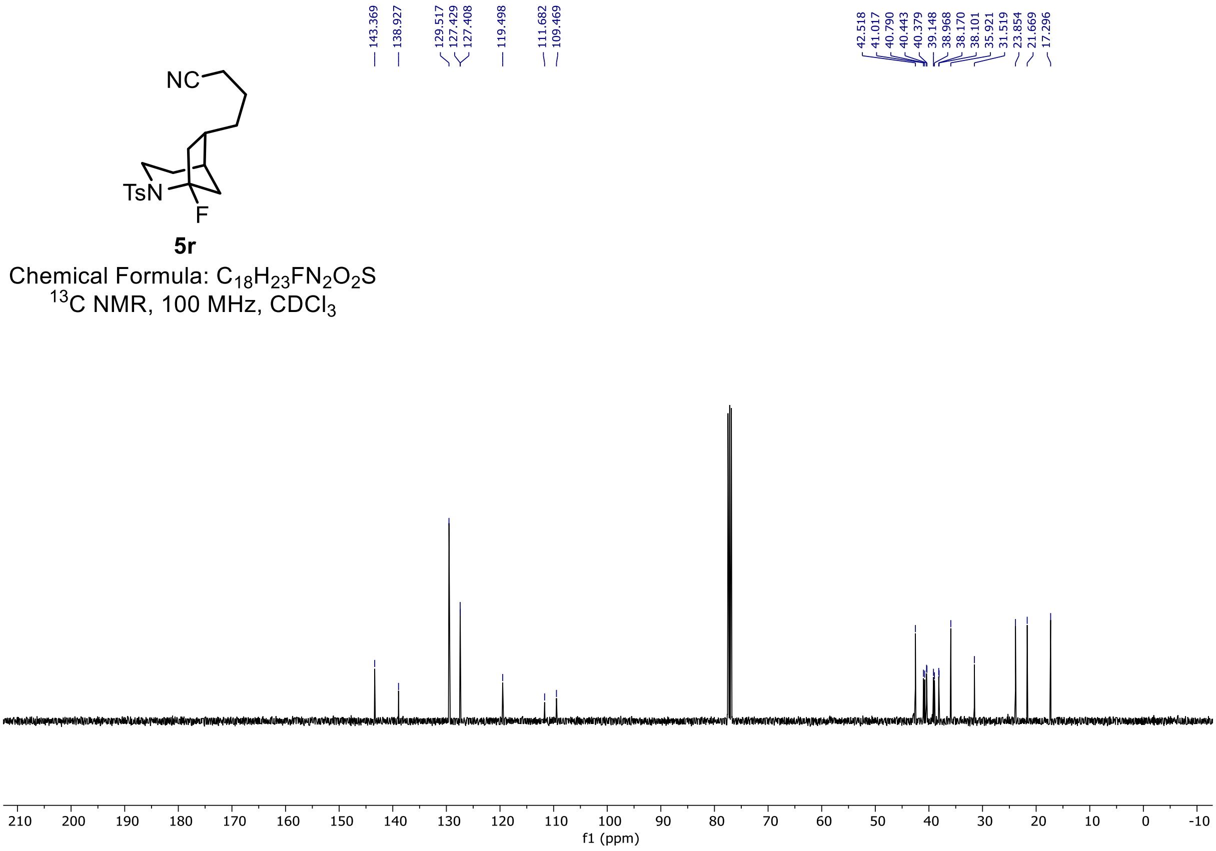
143.369 138.927 129.517 127.429 127.408 119.498 111.682 109.469
42.518 41.017 40.790 40.443 40.379 39.148 38.968 38.170 38.101 35.921 31.519 23.854 21.669 17.296
NC
TsN
F
5r
Chemical Formula: C18H23FN2O2S
13C NMR, 100 MHz, CDCl3
210 200 190 180 170 160 150 140 130 120 110 100 90 80 70 60 50 40 30 20 10 0 -10
f1 (ppm)

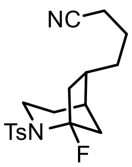

5r

Chemical Formula: $C_{18}H_{23}FN_2O_2S$

^{19}F NMR, 376 MHz, $CDCl_3$

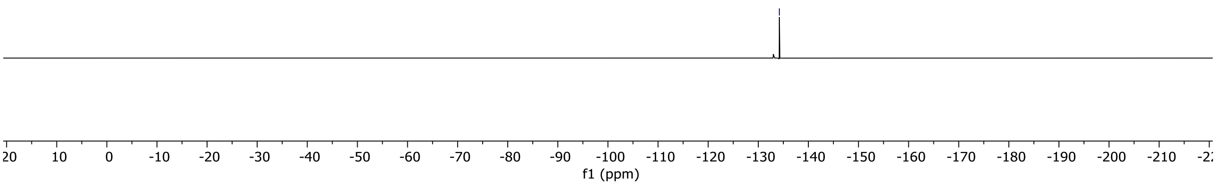

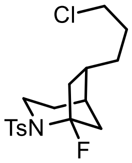

5s

Chemical Formula: $C_{17}H_{23}ClFNO_2S$

^1H NMR, 400 MHz, $CDCl_3$

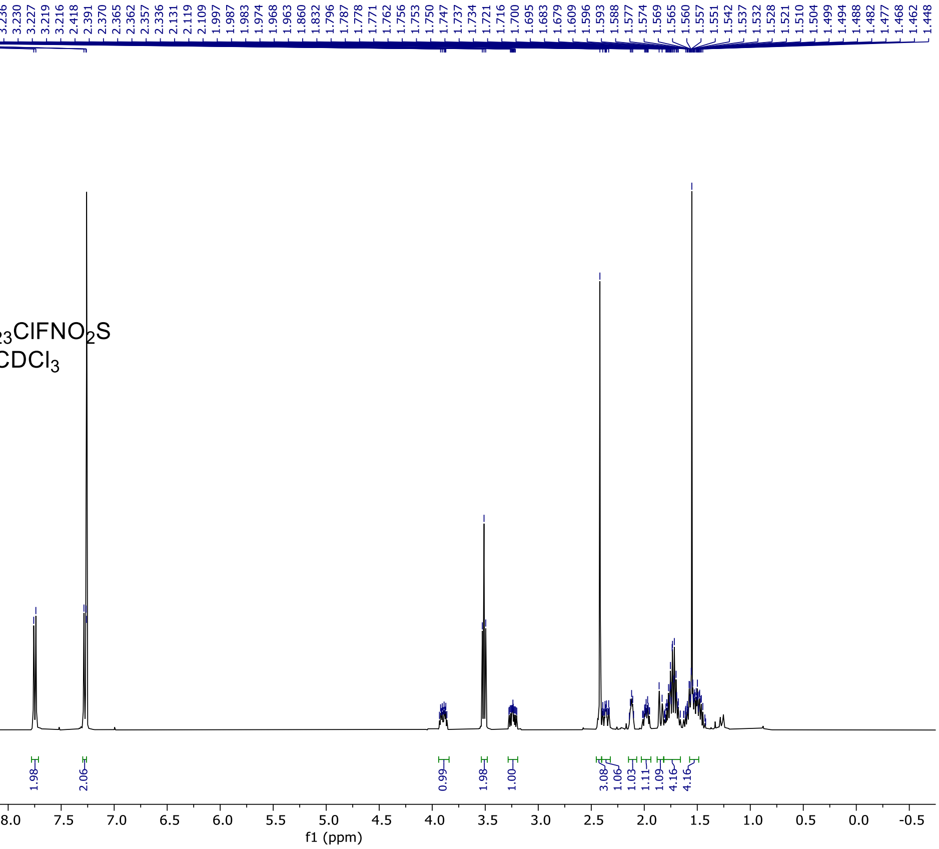

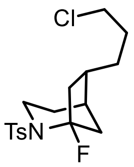

5s

Chemical Formula: $C_{17}H_{23}ClFNO_2S$

^{13}C NMR, 100 MHz, $CDCl_3$

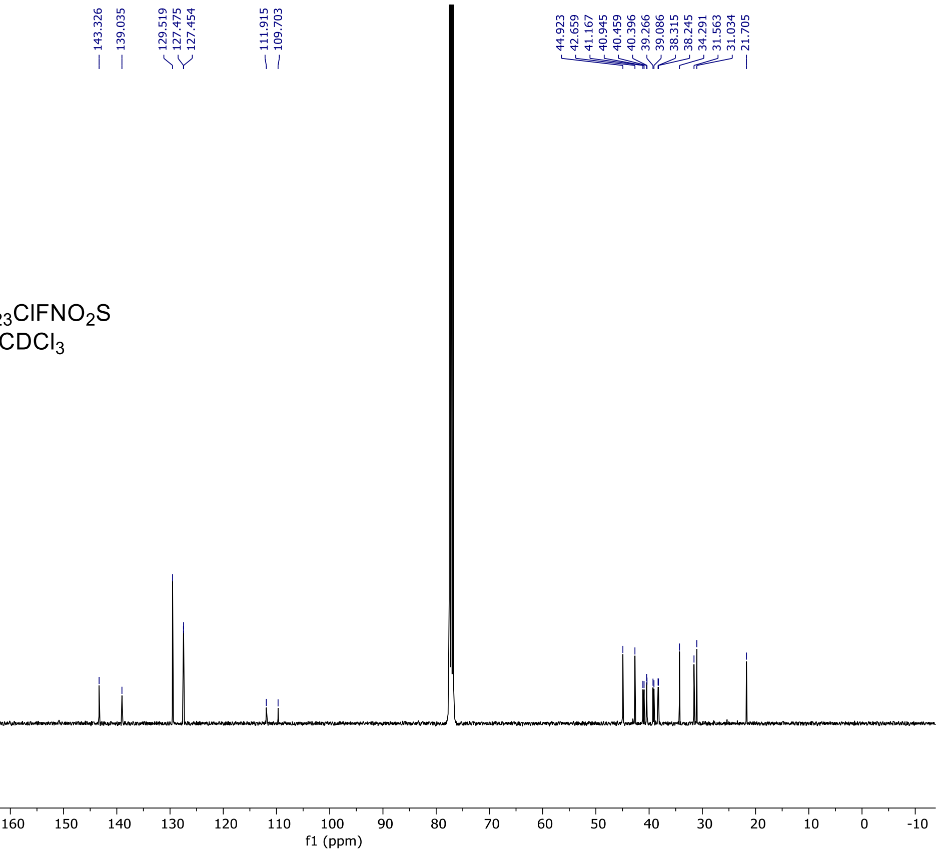

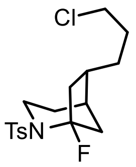

5s

Chemical Formula: $C_{17}H_{23}ClFNO_2S$

^{19}F NMR, 376 MHz, $CDCl_3$

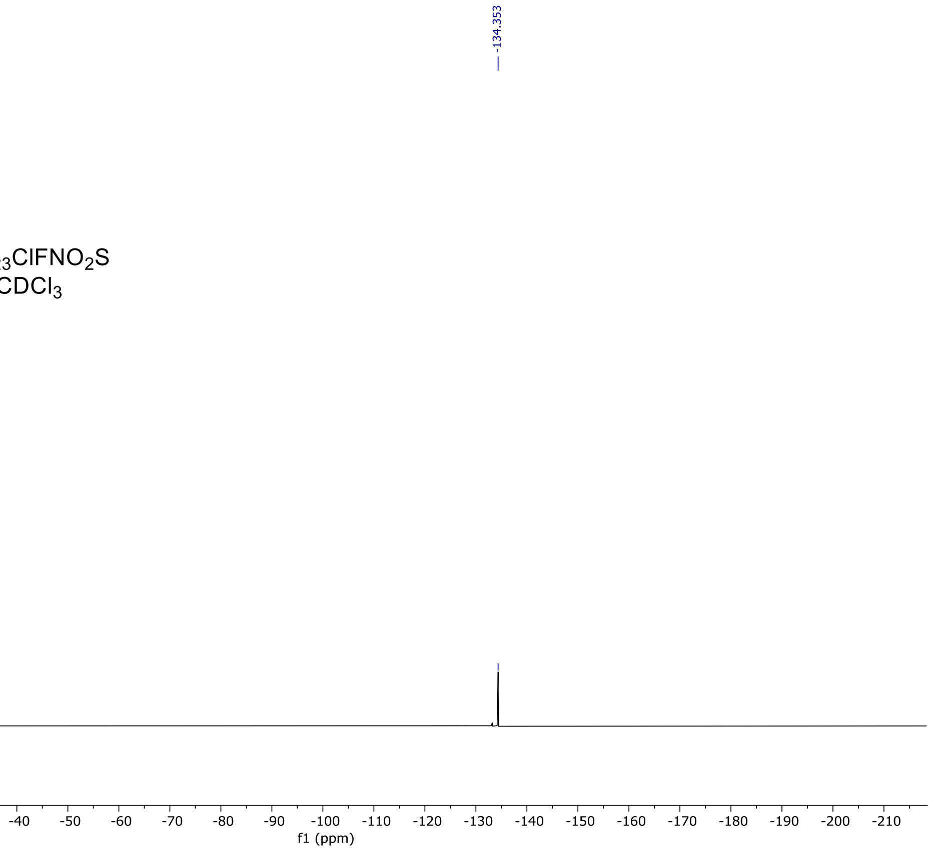

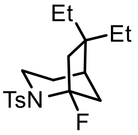

5t

Chemical Formula: $C_{18}H_{26}FNO_2S$

^{1}H NMR, 600 MHz, $CDCl_3$

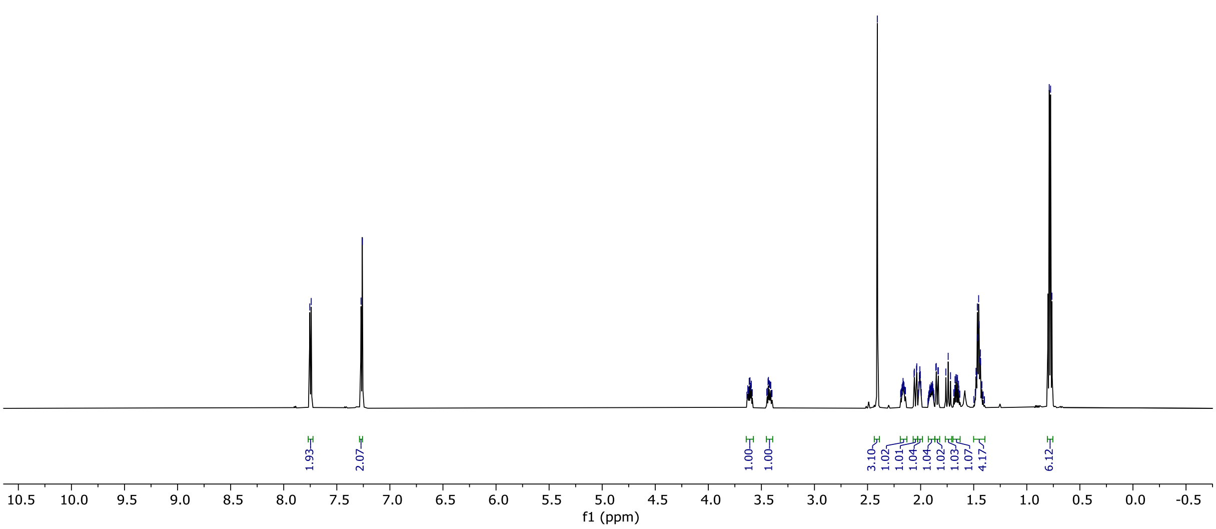

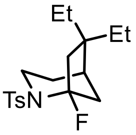

5t

Chemical Formula: $C_{18}H_{26}FNO_2S$

^{13}C NMR, 150 MHz, $CDCl_3$

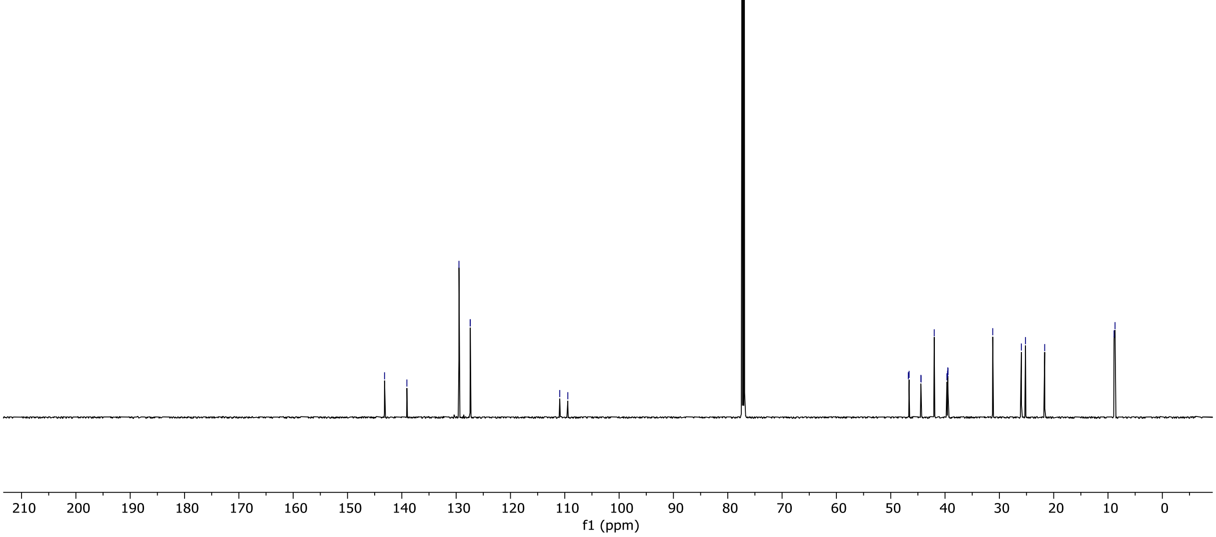

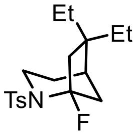

5t

Chemical Formula: $C_{18}H_{26}FNO_2S$

^{19}F NMR, 376 MHz, $CDCl_3$

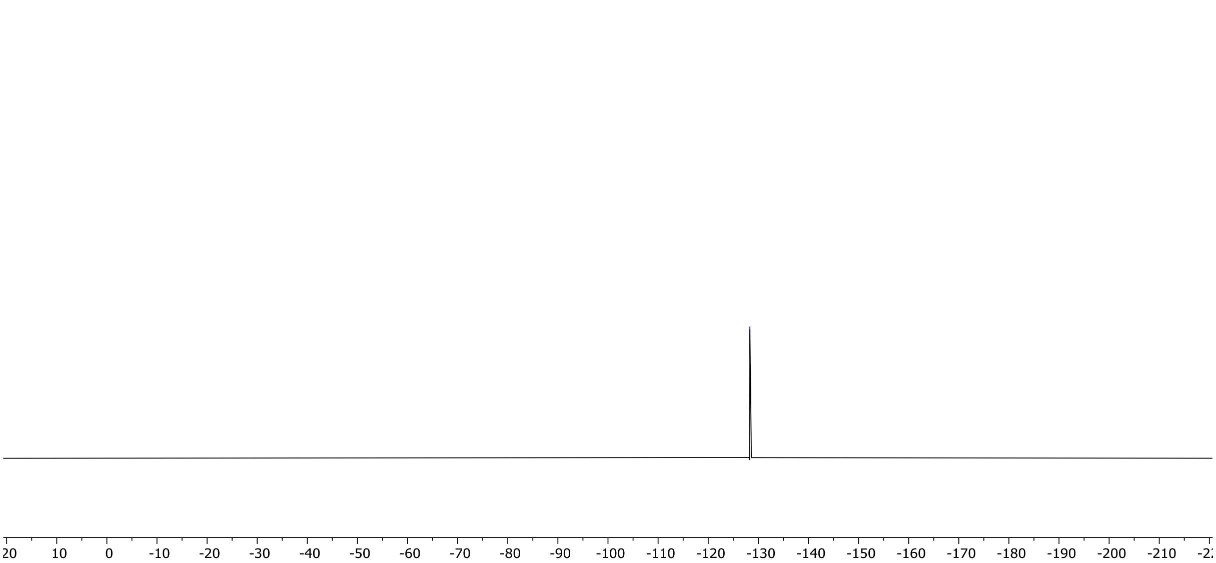

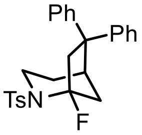

5u

Chemical Formula: $C_{26}H_{26}FNO_2S$

^{1}H NMR, 600 MHz, $CDCl_3$

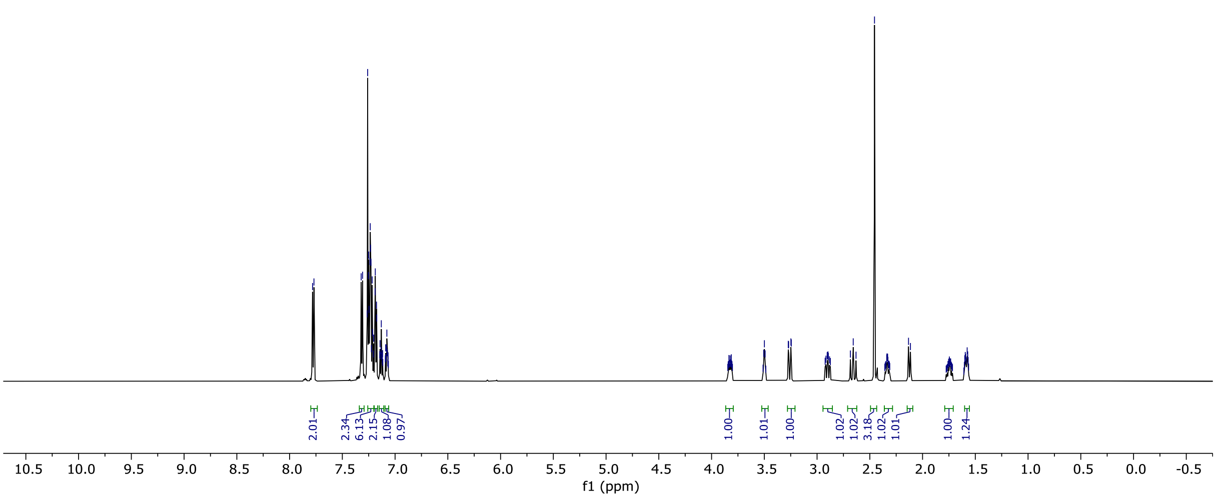

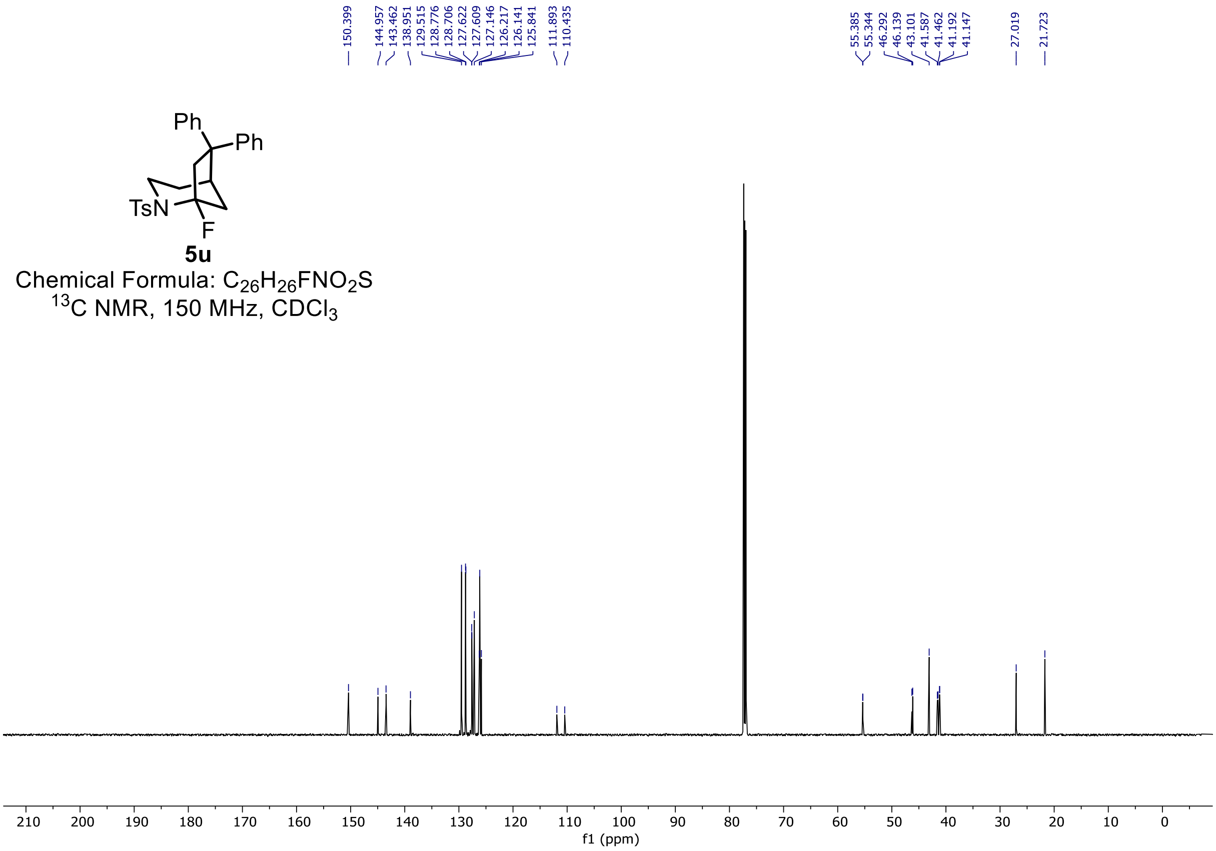
150.399
144.957
143.462
138.951
129.515
128.776
128.706
127.622
127.609
127.146
126.217
126.141
125.841
111.893
110.435
55.385
55.344
46.292
46.139
43.101
41.587
41.462
41.192
41.147
27.019
21.723
Ph
Ph
TsN
F
5u
Chemical Formula: $C_{26}H_{26}FNO_2S$
^{13}C NMR, 150 MHz, $CDCl_3$
f1 (ppm)

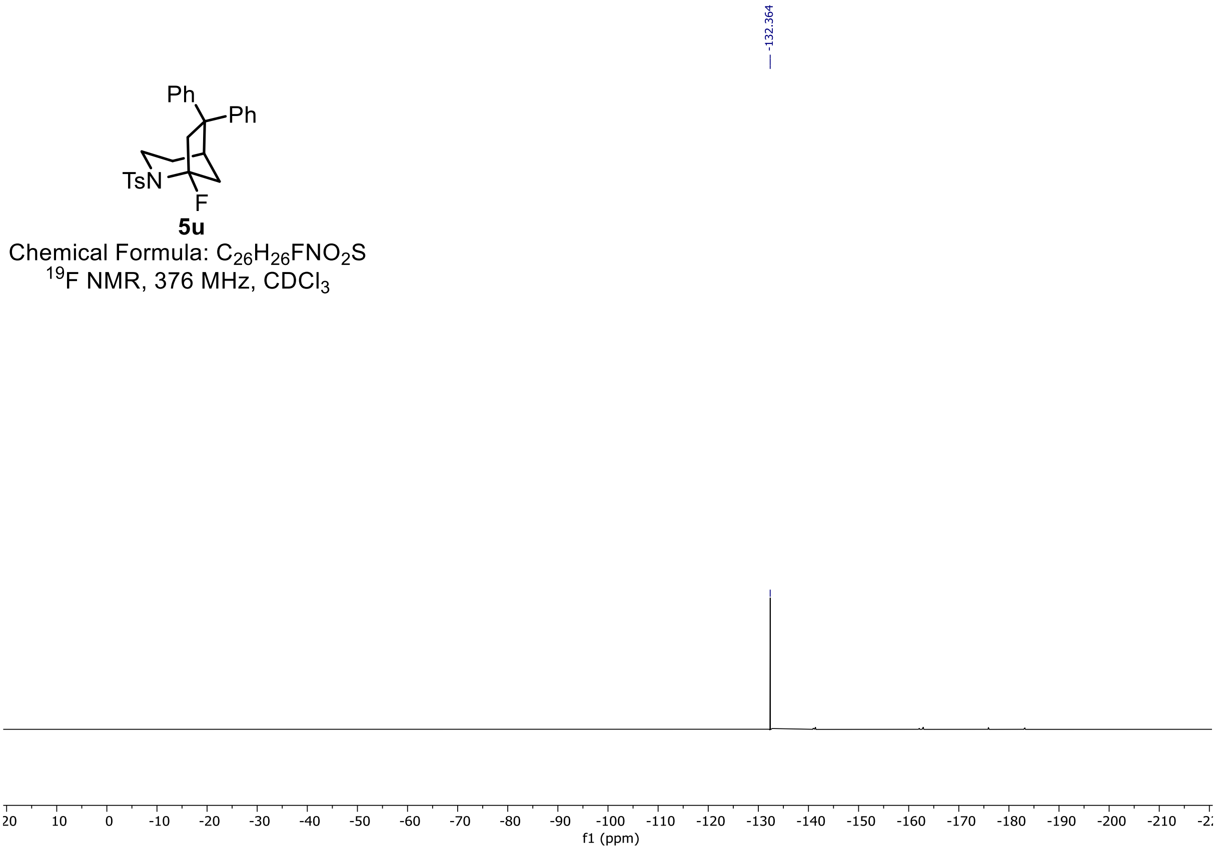
-132.364
Ph
Ph
TsN
F
5u
Chemical Formula: $C_{26}H_{26}FNO_2S$
^{19}F NMR, 376 MHz, $CDCl_3$
f1 (ppm)

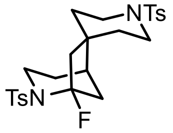

5v

Chemical Formula: $C_{25}H_{31}FN_2O_4S_2$

^1H NMR, 400 MHz, $CDCl_3$

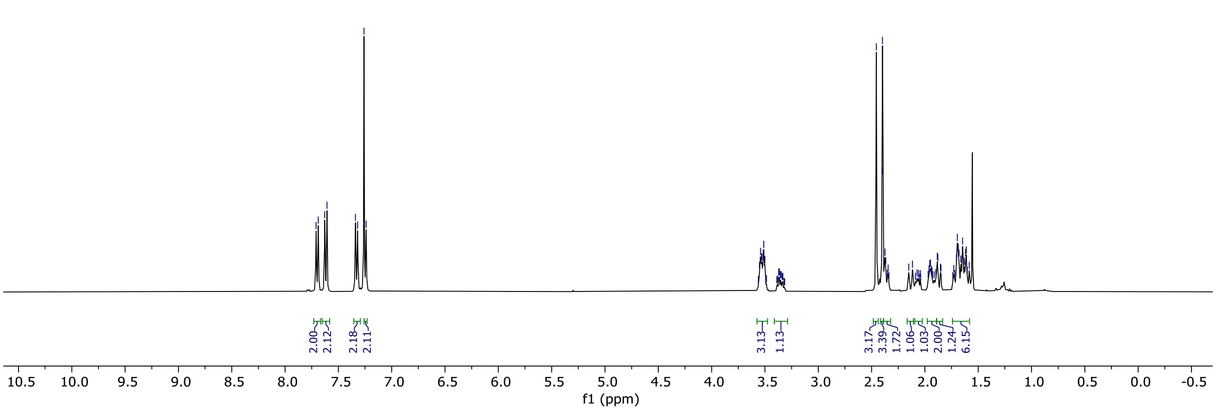

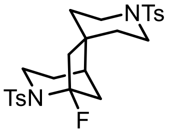

5v

Chemical Formula: $C_{25}H_{31}FN_2O_4S_2$

^{13}C NMR, 100 MHz, $CDCl_3$

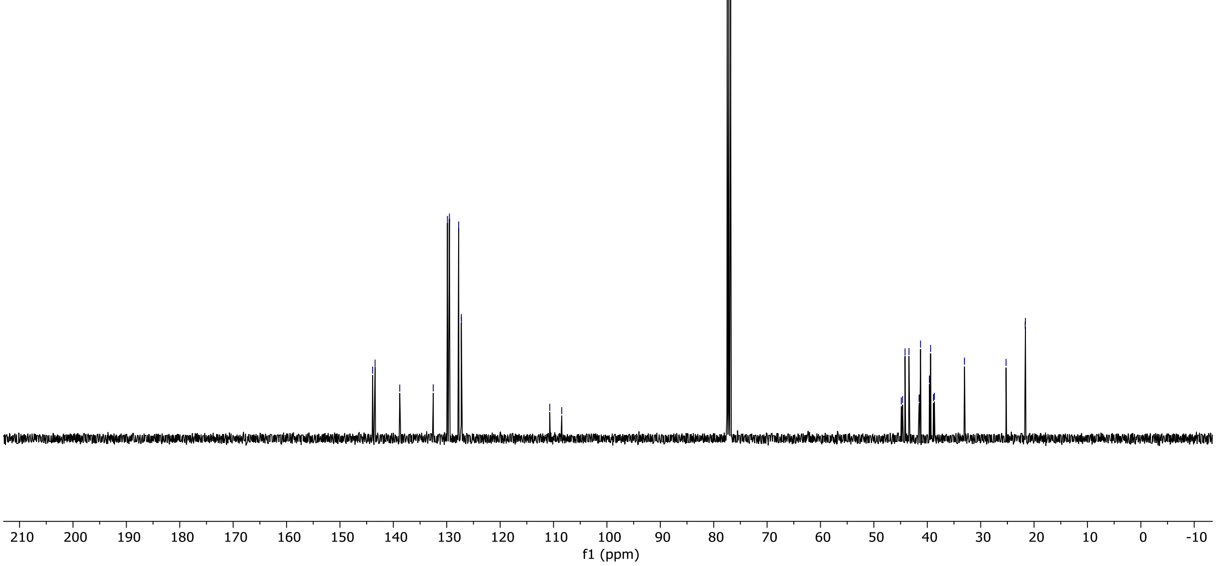

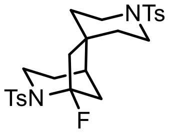

5v

Chemical Formula: $C_{25}H_{31}FN_2O_4S_2$

^{19}F NMR, 376 MHz, $CDCl_3$

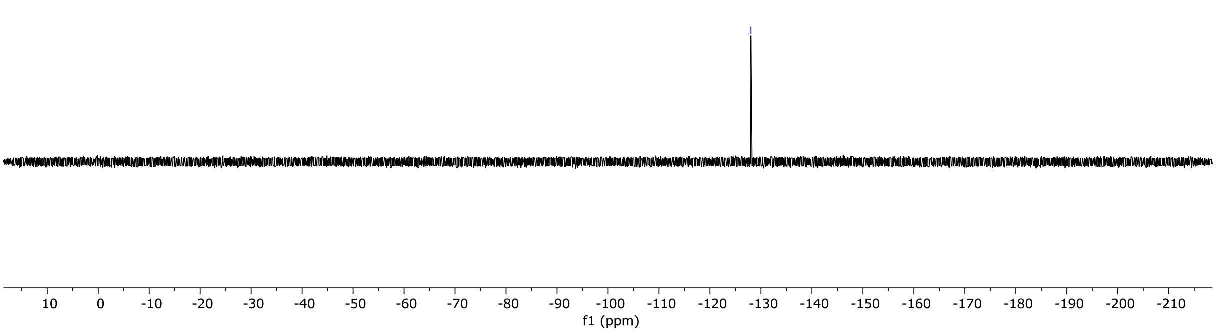

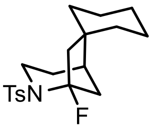

5w

Chemical Formula: $C_{19}H_{26}FNO_2S$

^{1}H NMR, 400 MHz, $CDCl_3$

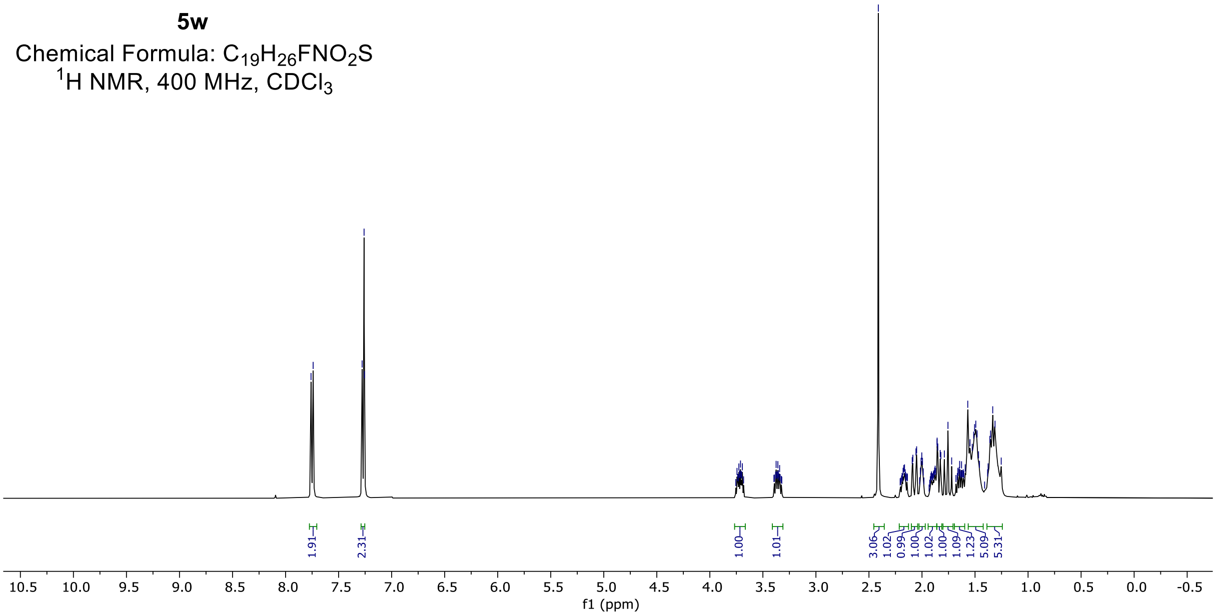

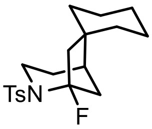

5w

Chemical Formula: $C_{19}H_{26}FNO_2S$

^{13}C NMR, 100 MHz, $CDCl_3$

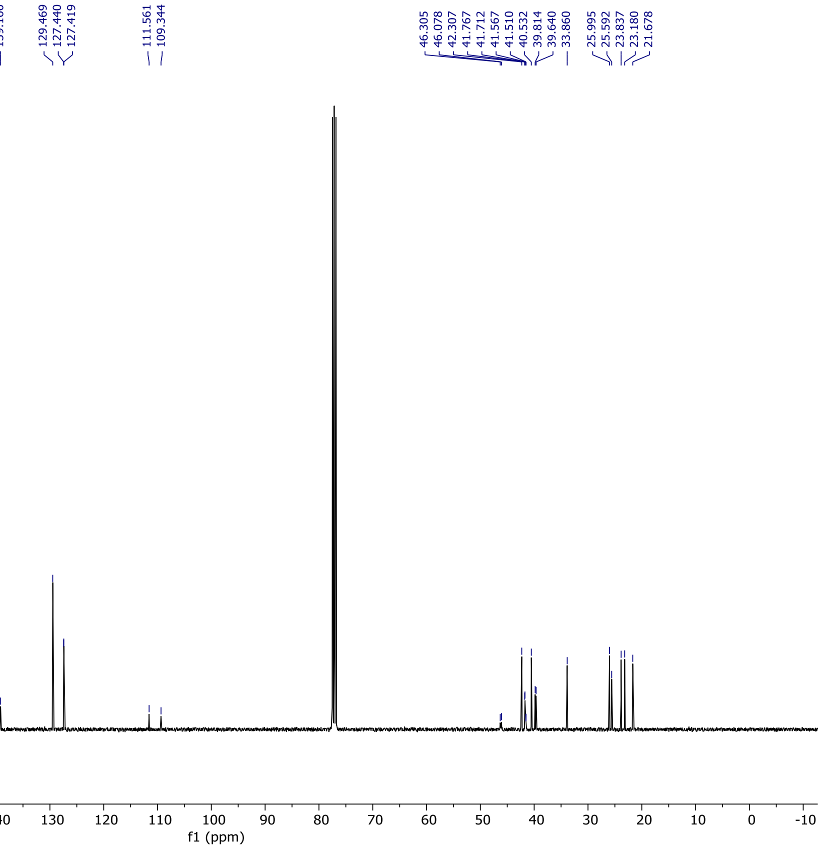

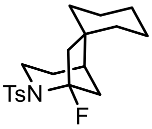

5w

Chemical Formula: $C_{19}H_{26}FNO_2S$

^{19}F NMR, 376 MHz, $CDCl_3$

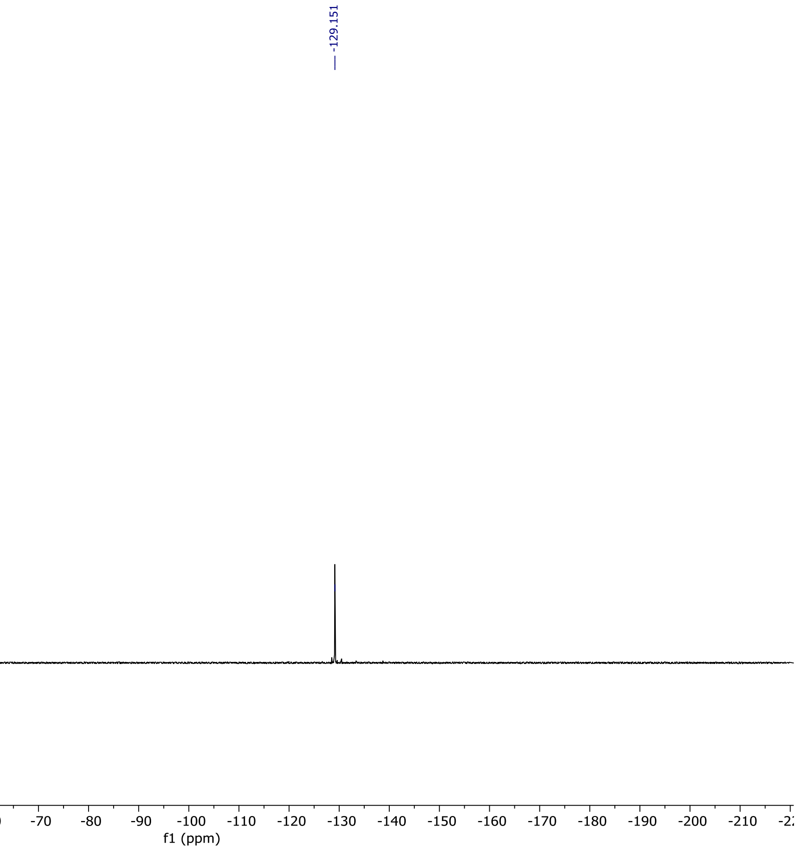

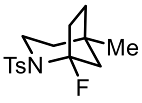

5x

Chemical Formula: $C_{15}H_{20}FNO_2S$

^{1}H NMR, 800 MHz, $CDCl_3$

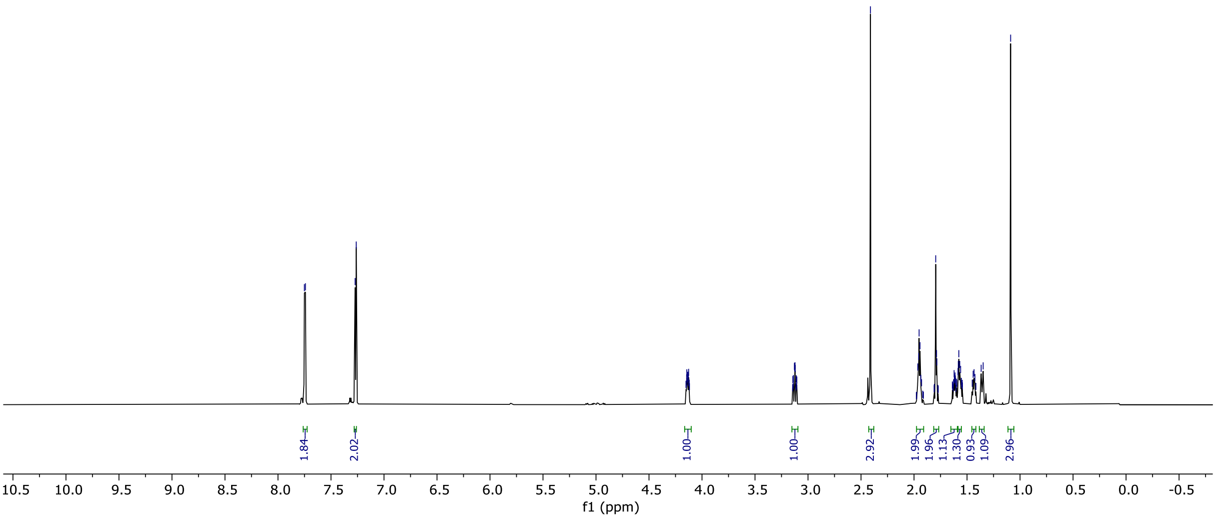

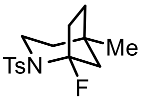

5x

Chemical Formula: $C_{15}H_{20}FNO_2S$

^{13}C NMR, 200 MHz, $CDCl_3$

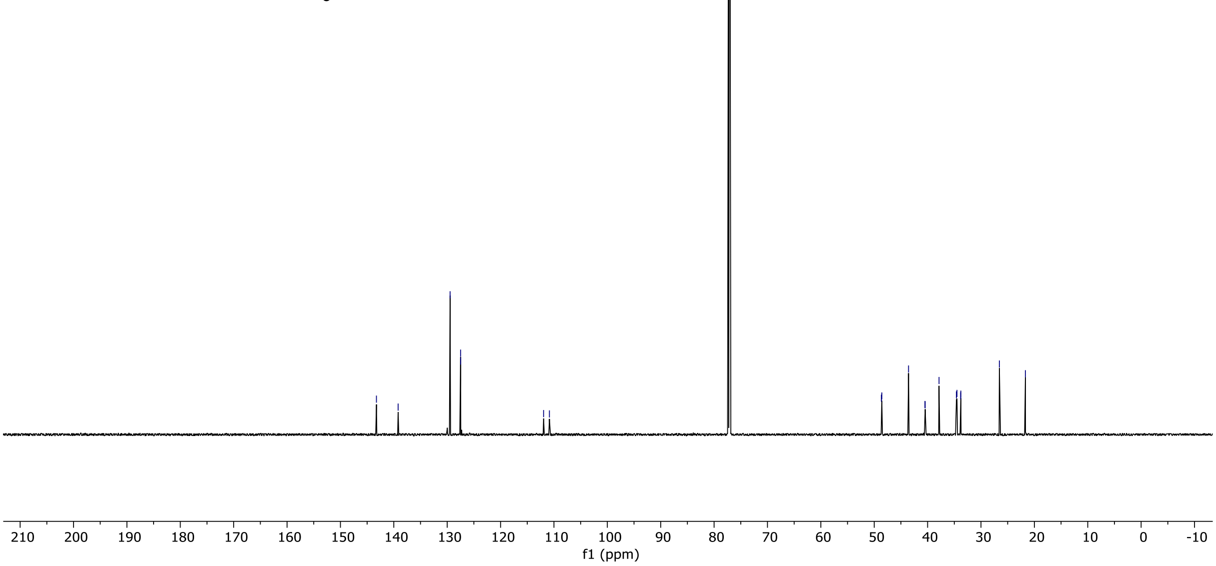

5x

Chemical Formula: $C_{15}H_{20}FNO_2S$

^{19}F NMR, 376 MHz, $CDCl_3$

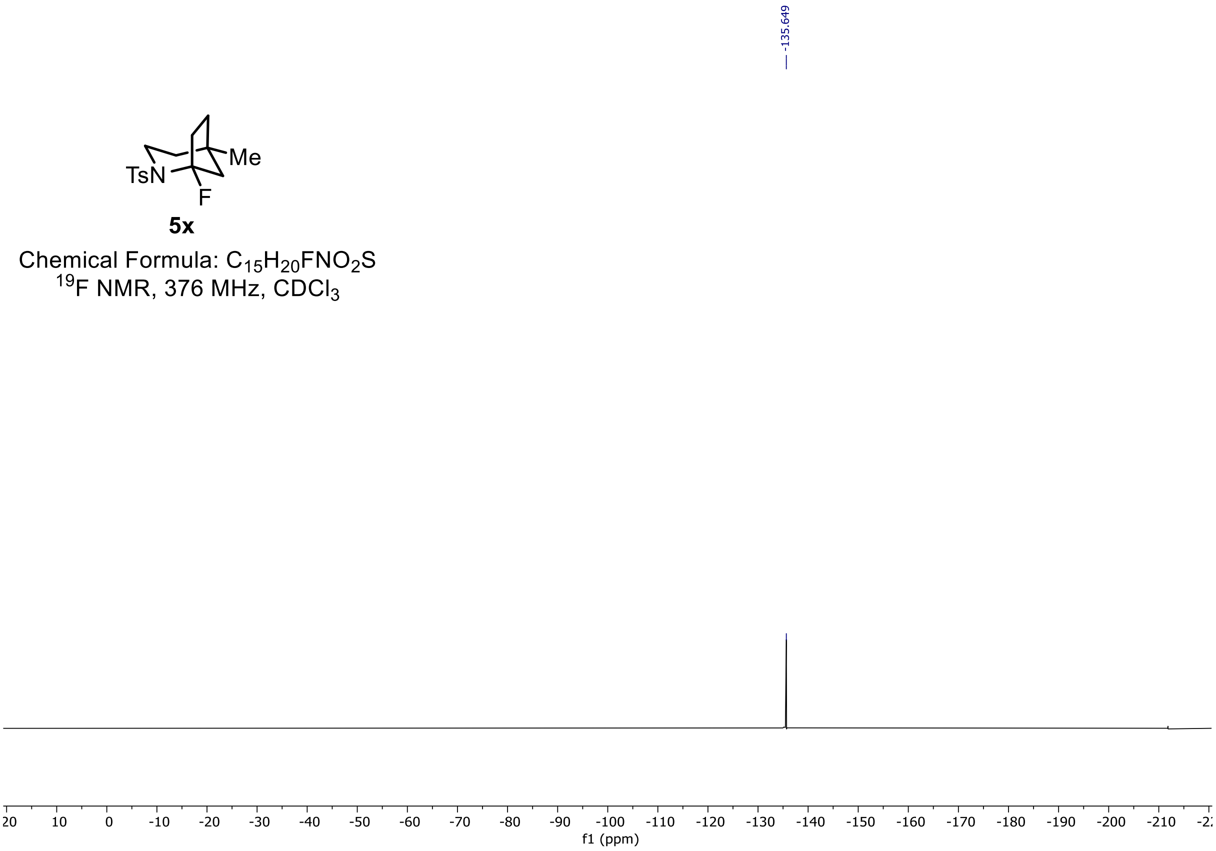

dr = 1.9:1

5xa

Chemical Formula: $C_{15}H_{20}FNO_2S$

^{1}H NMR, 600 MHz, $CDCl_3$

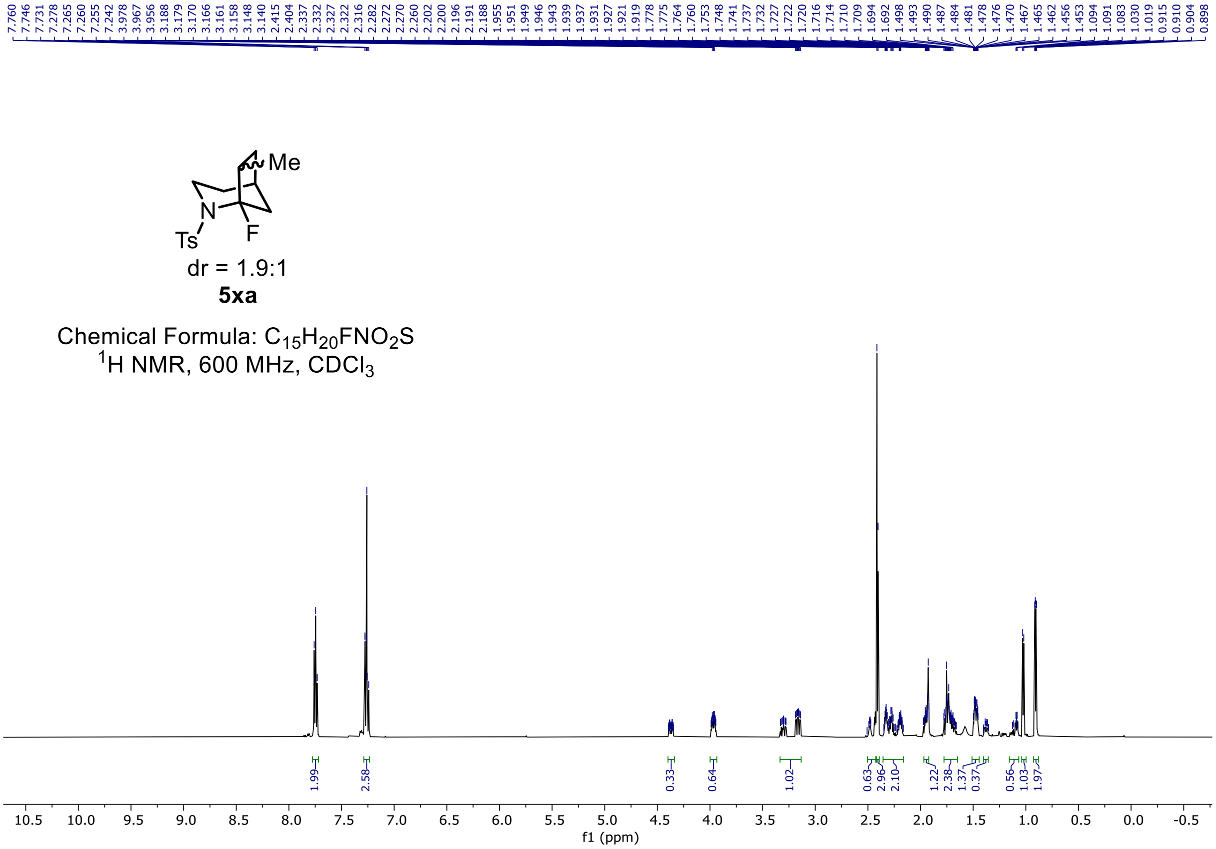

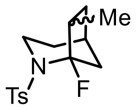

dr = 1.9:1

5xa

Chemical Formula: $C_{15}H_{20}FNO_2S$

^{13}C NMR, 150 MHz, $CDCl_3$

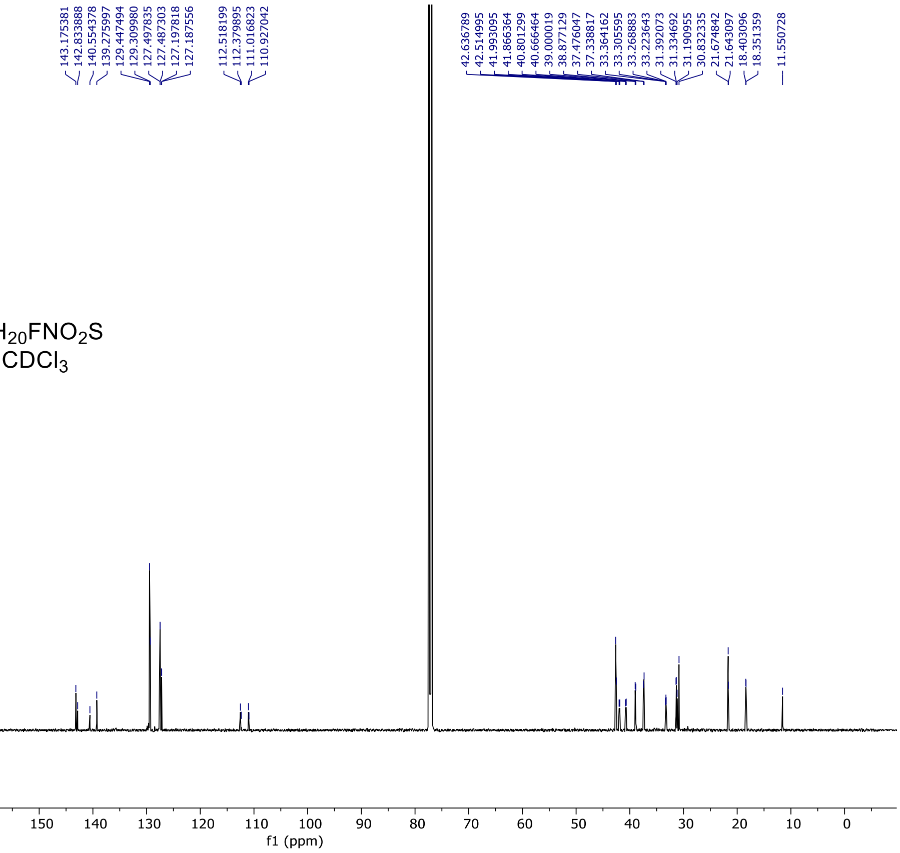

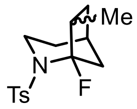

dr = 1.9:1

5xa

Chemical Formula: $C_{15}H_{20}FNO_2S$

^{19}F NMR, 376 MHz, $CDCl_3$

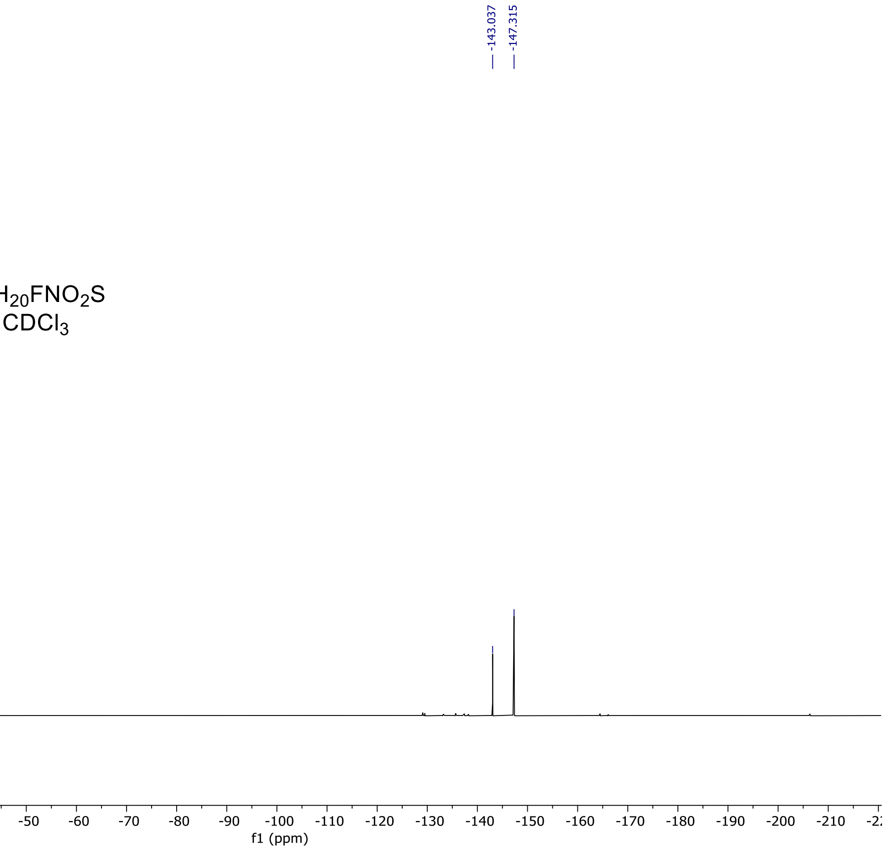

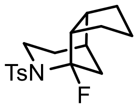

5y

Chemical Formula: $C_{17}H_{22}FNO_2S$

^{1}H NMR, 400 MHz, $CDCl_3$

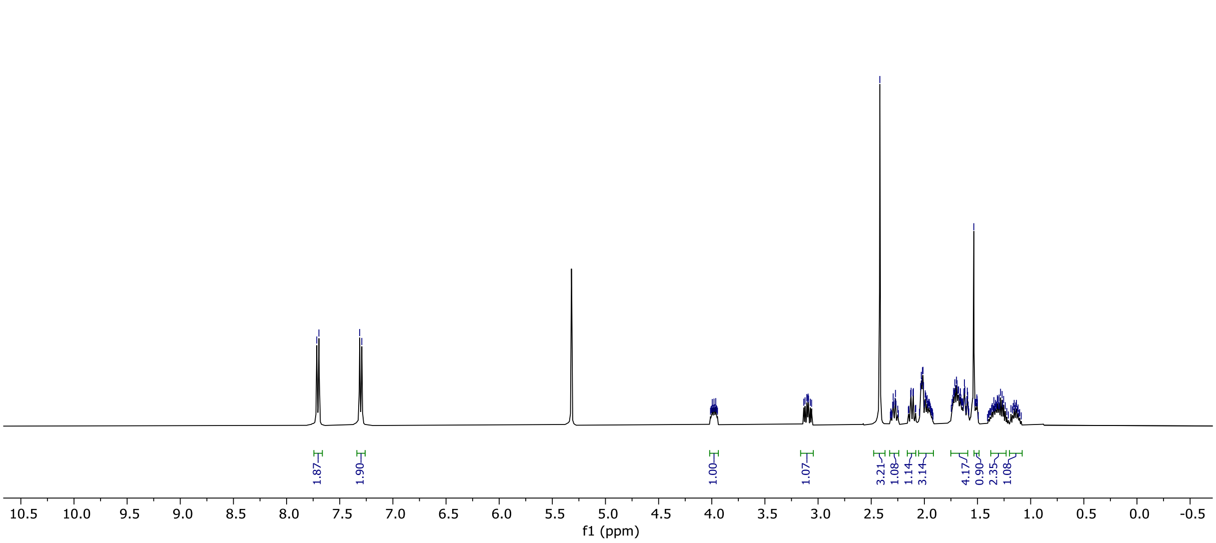

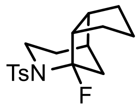

5y

Chemical Formula: $C_{17}H_{22}FNO_2S$

^{13}C NMR, 100 MHz, $CDCl_3$

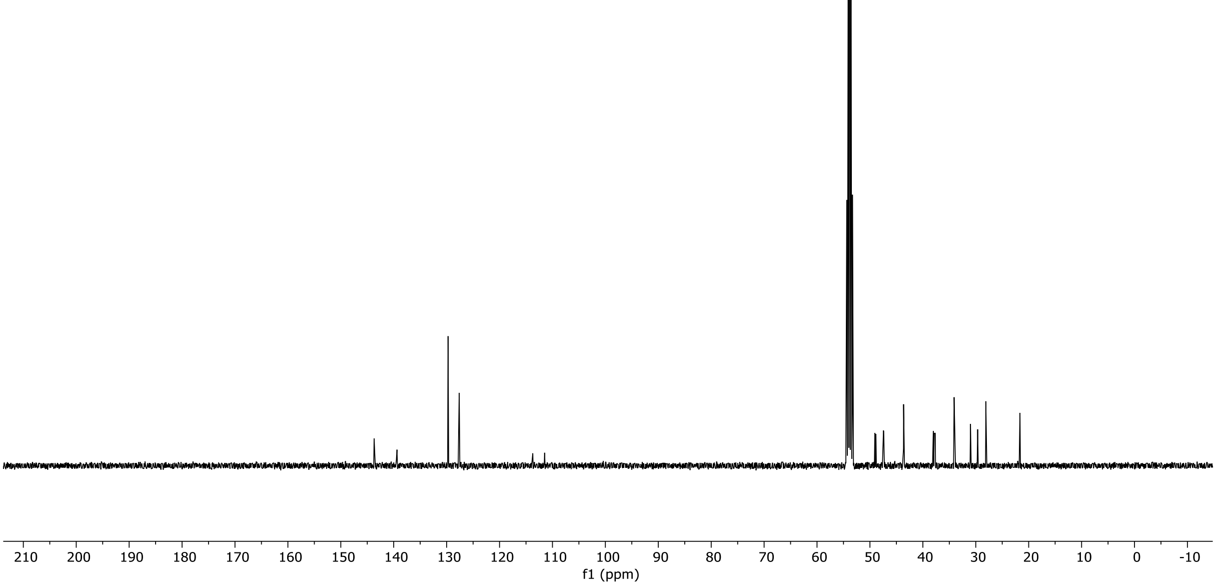

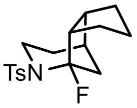

5y

Chemical Formula: $C_{17}H_{22}FNO_2S$

^{19}F NMR, 376 MHz, $CDCl_3$

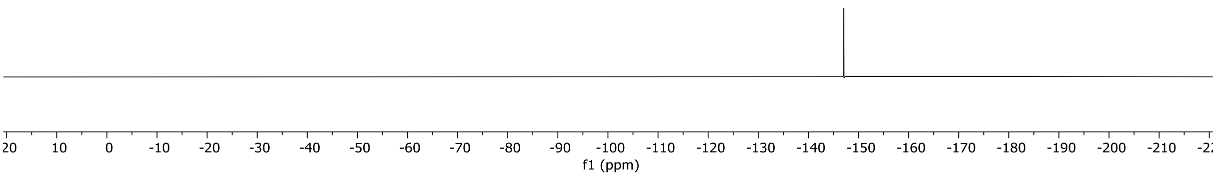

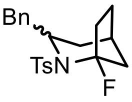

5z

Chemical Formula: $C_{21}H_{24}FNO_2S$

^{1}H NMR, 600 MHz, $CDCl_3$

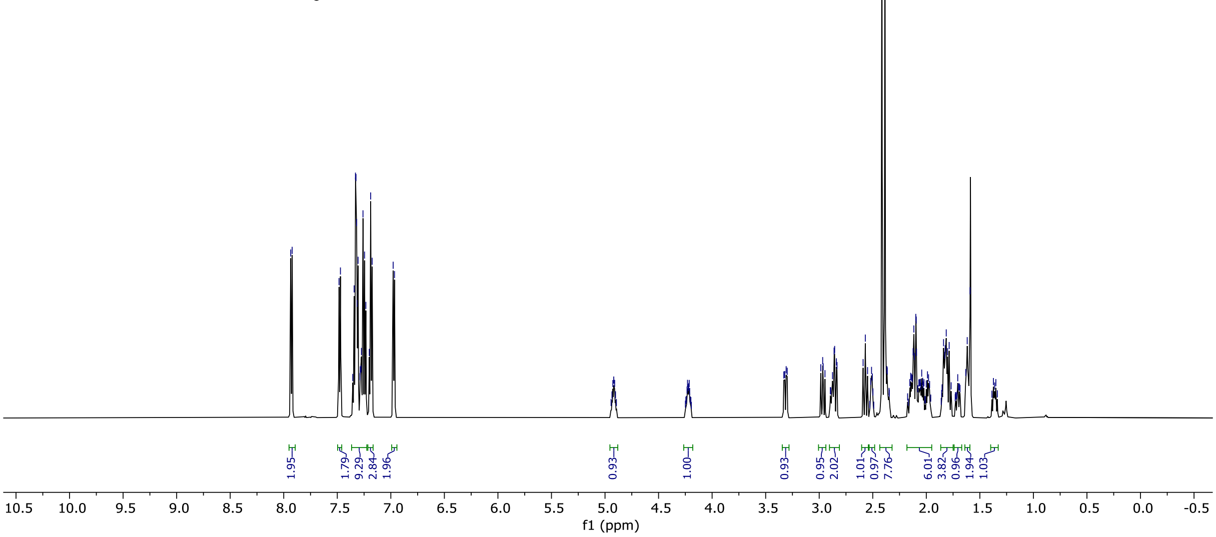

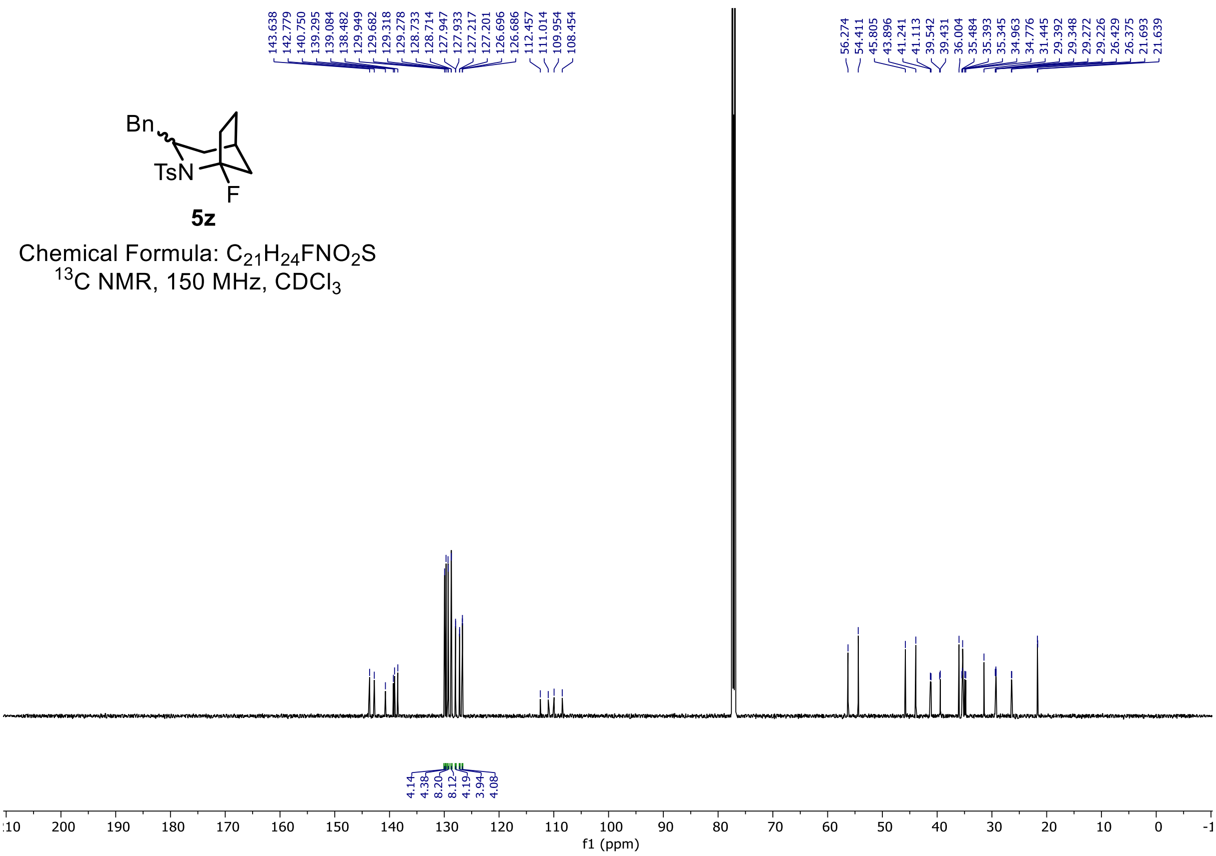

143.638
142.779
140.750
139.295
139.084
138.482
129.949
129.682
129.318
129.278
128.733
128.714
127.947
127.933
127.217
127.201
126.696
126.686
112.457
111.014
109.954
108.454
56.274
54.411
45.805
43.896
41.241
41.113
39.542
39.431
36.004
35.484
35.393
35.345
34.963
34.776
31.445
29.392
29.348
29.272
29.226
26.429
26.375
21.693
21.639
Bn
TsN
F
5z
Chemical Formula: $C_{21}H_{24}FNO_2S$
^{13}C NMR, 150 MHz, $CDCl_3$
4.14
4.38
8.20
8.12
4.19
3.94
4.08
f1 (ppm)

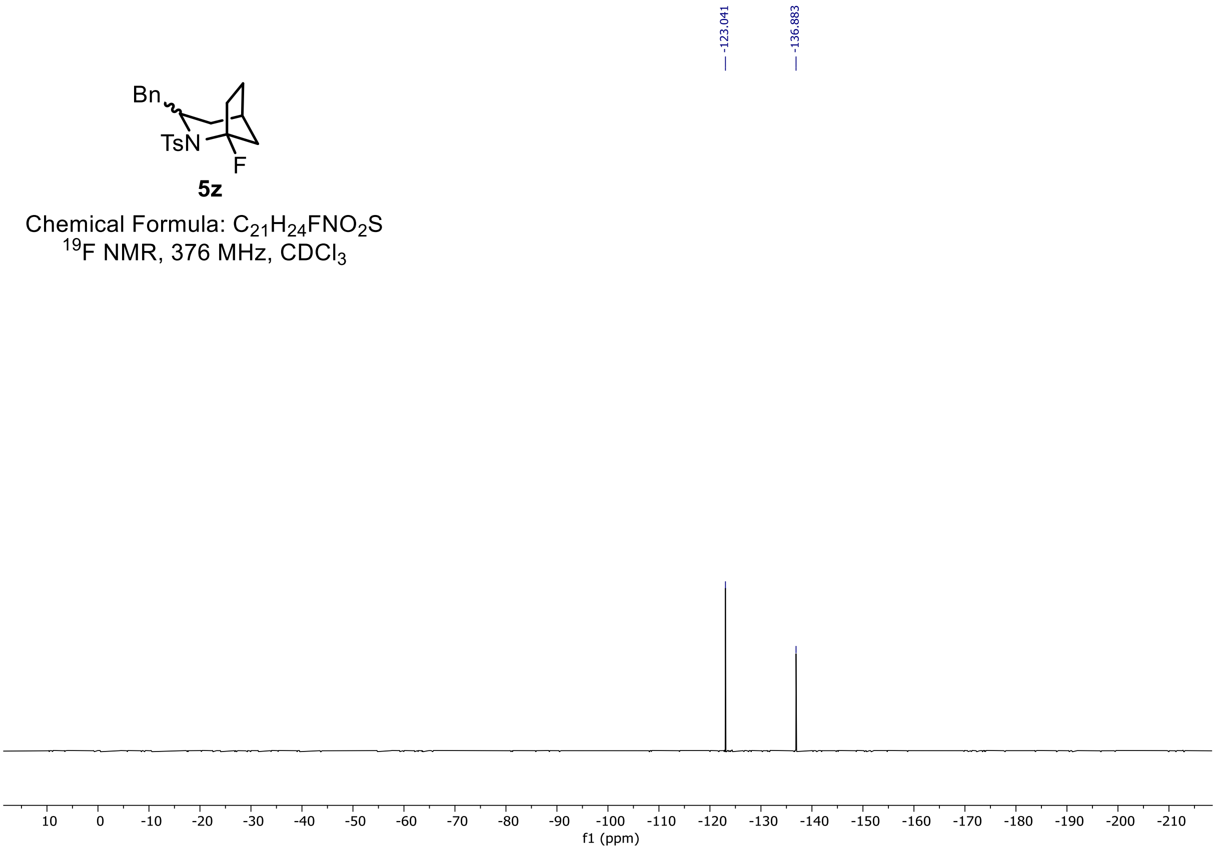

-123.041
-136.883
Bn
TsN
F
5z
Chemical Formula: $C_{21}H_{24}FNO_2S$
^{19}F NMR, 376 MHz, $CDCl_3$
f1 (ppm)

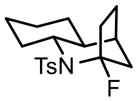

5aa

Chemical Formula: $C_{18}H_{24}FNO_2S$

^{1}H NMR, 600 MHz, $CDCl_3$

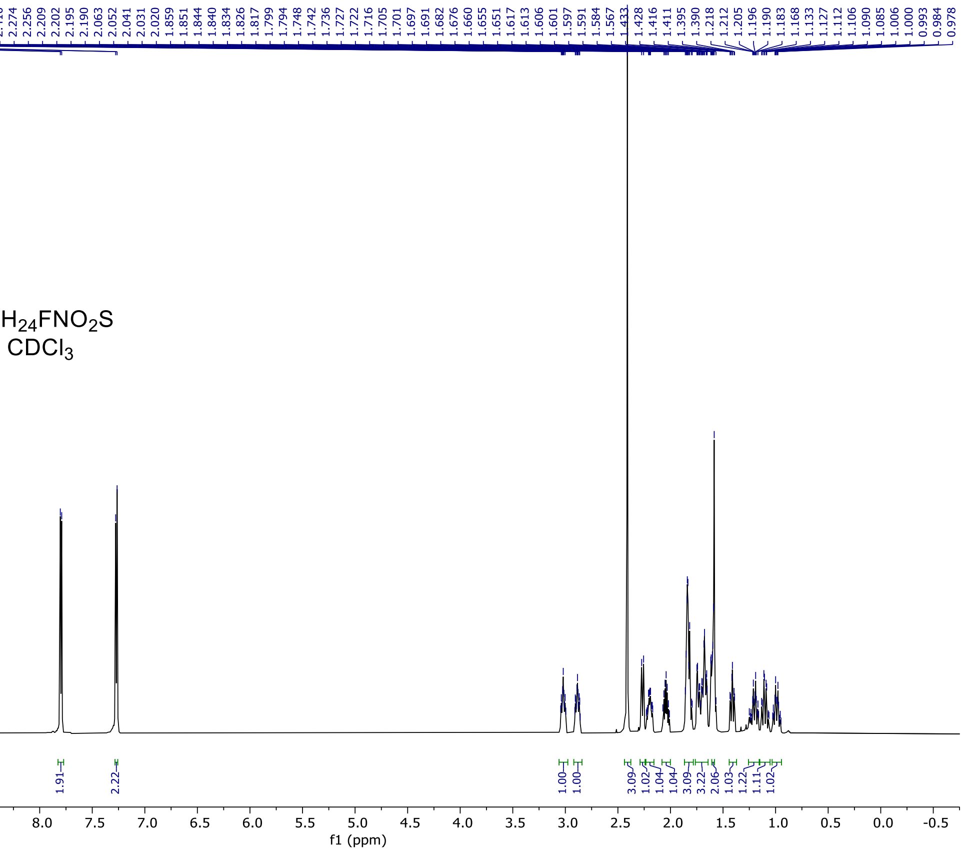

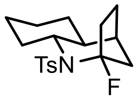

5aa

Chemical Formula: $C_{18}H_{24}FNO_2S$

^{13}C NMR, 150 MHz, $CDCl_3$

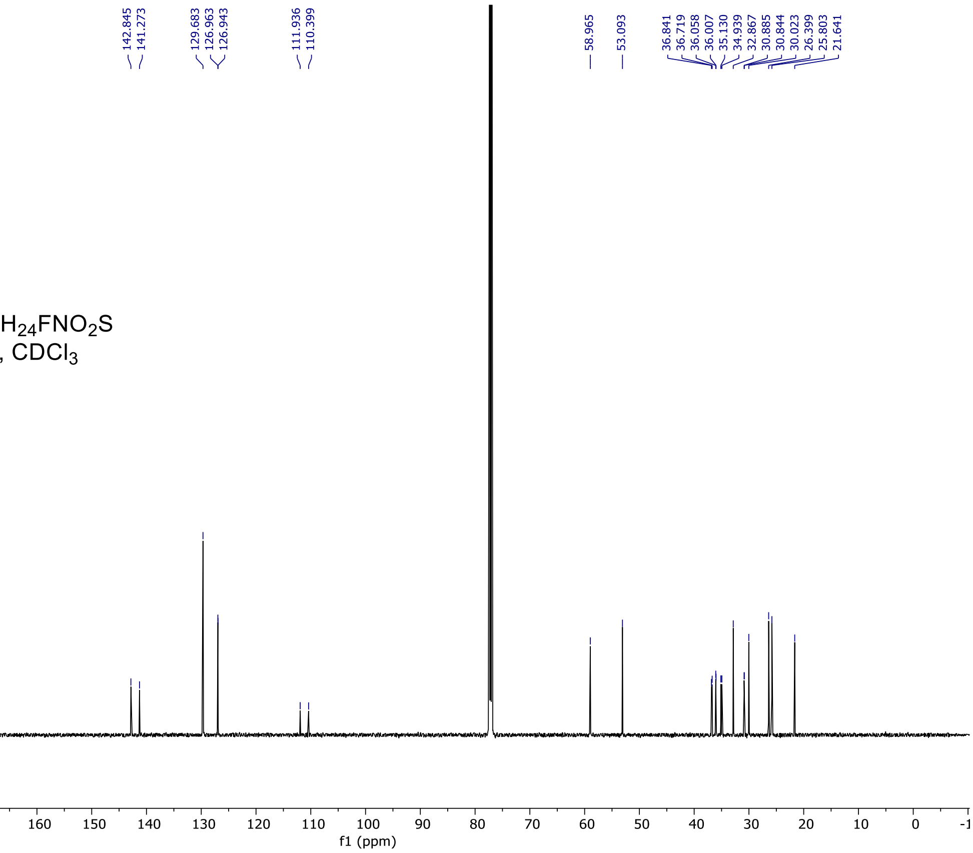

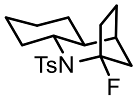

5aa
Chemical Formula: $C_{18}H_{24}FNO_2S$
^{19}F NMR, 376 MHz, $CDCl_3$

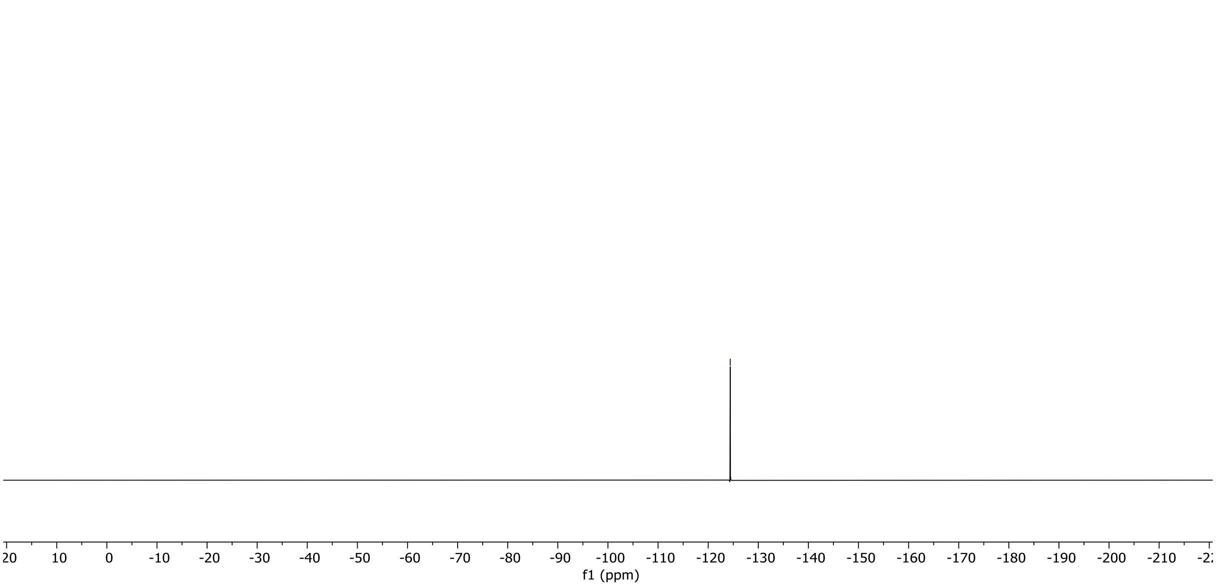

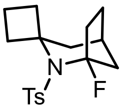

5ab
Chemical Formula: $C_{17}H_{22}FNO_2S$
^{1}H NMR, 400 MHz, $CDCl_3$

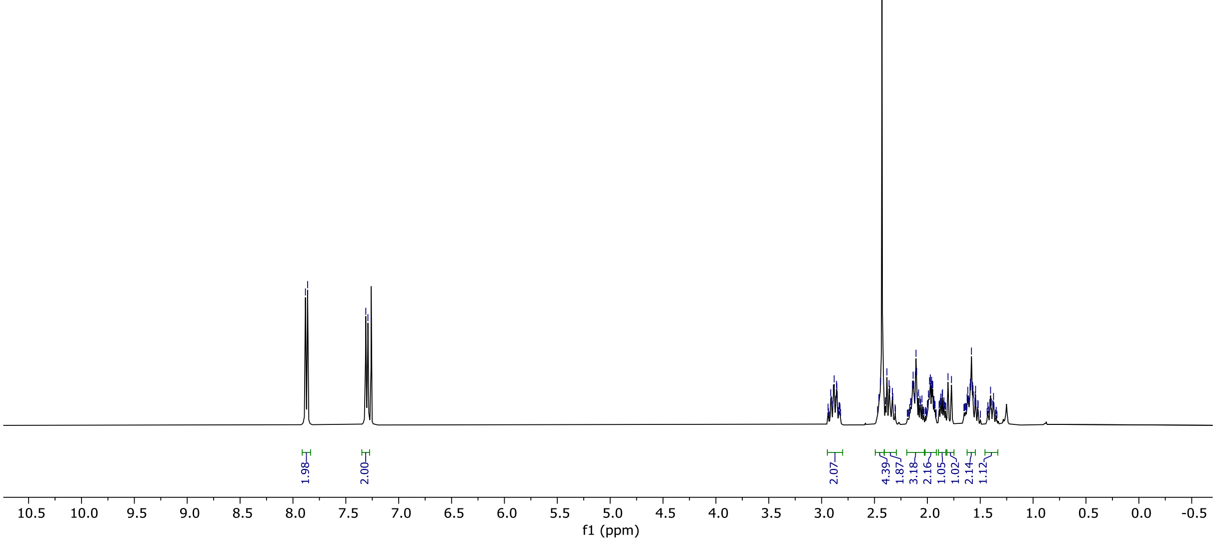

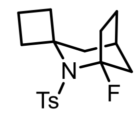

5ab

Chemical Formula: $C_{17}H_{22}FNO_2S$

^{13}C NMR, 100 MHz, $CDCl_3$

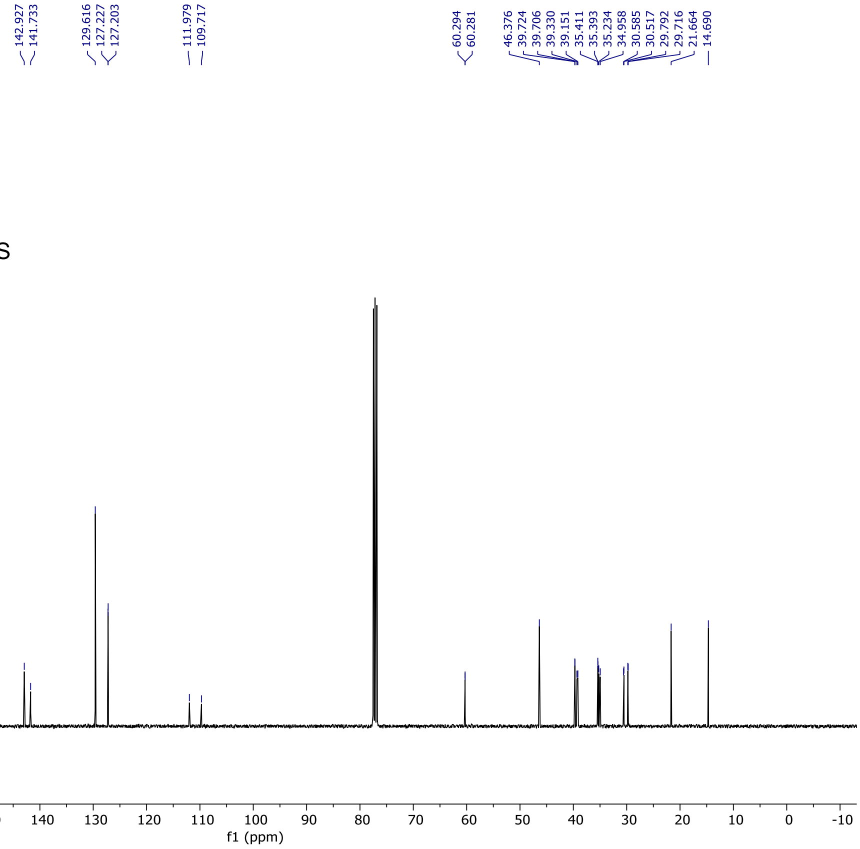

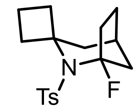

5ab

Chemical Formula: $C_{17}H_{22}FNO_2S$

^{19}F NMR, 376 MHz, $CDCl_3$

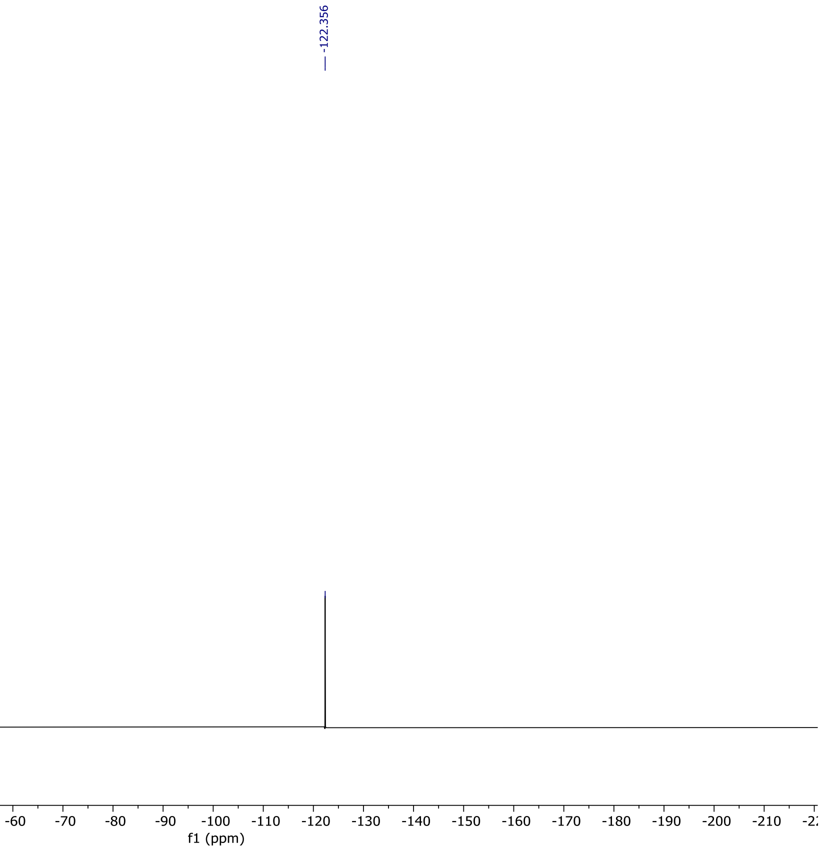

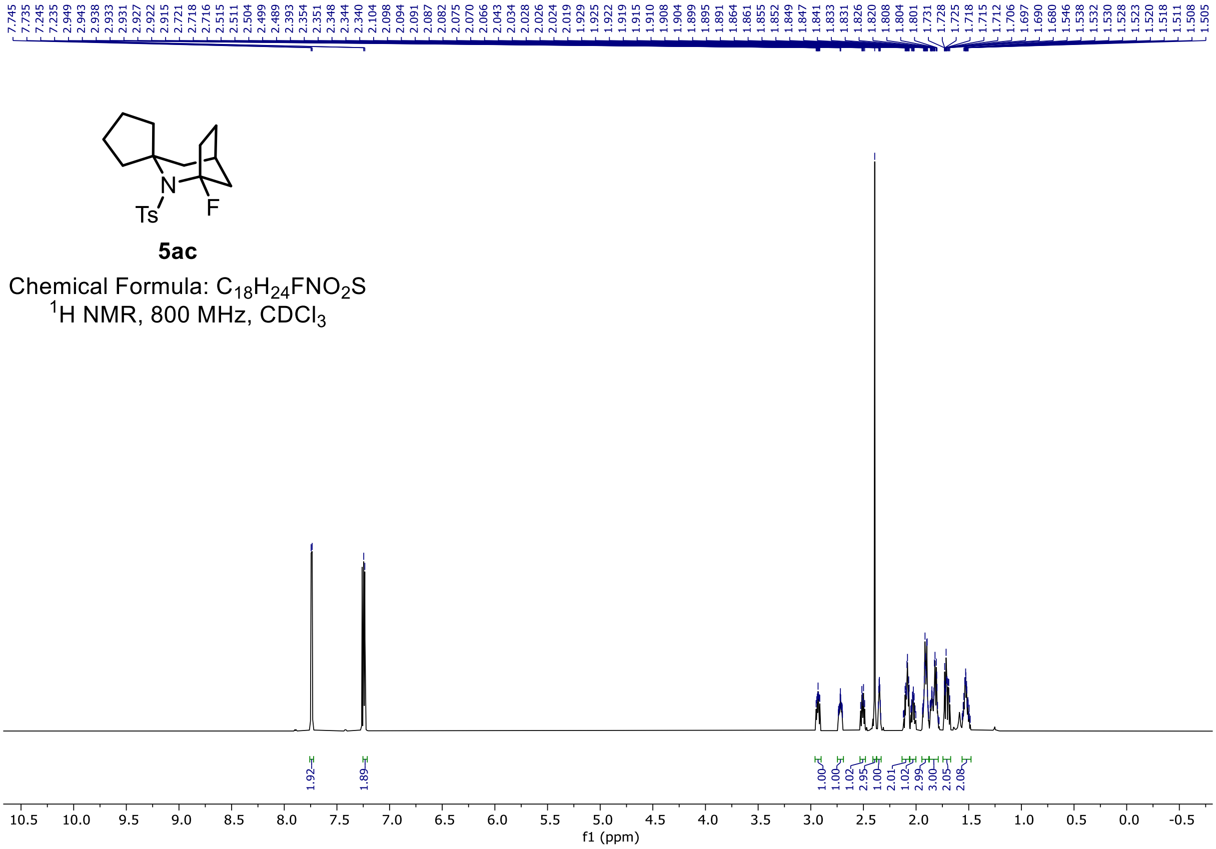

7.745 7.735 7.245 7.235 2.949 2.943 2.938 2.933 2.931 2.927 2.922 2.915 2.721 2.718 2.716 2.515 2.511 2.504 2.499 2.489 2.393 2.354 2.351 2.348 2.344 2.340 2.104 2.098 2.094 2.091 2.087 2.082 2.075 2.070 2.066 2.043 2.034 2.028 2.026 2.024 2.019 1.929 1.925 1.922 1.919 1.915 1.910 1.908 1.904 1.899 1.895 1.891 1.864 1.861 1.855 1.852 1.849 1.847 1.841 1.833 1.831 1.826 1.820 1.808 1.804 1.801 1.731 1.728 1.725 1.718 1.715 1.712 1.706 1.697 1.690 1.680 1.546 1.538 1.532 1.530 1.528 1.523 1.520 1.518 1.511 1.508 1.505
N
Ts
F
5ac
Chemical Formula: $C_{18}H_{24}FNO_2S$
^{1}H NMR, 800 MHz, $CDCl_3$
1.92 1.89 1.00 1.00 1.02 2.95 1.00 2.01 1.02 2.99 3.00 2.05 2.08
10.5 10.0 9.5 9.0 8.5 8.0 7.5 7.0 6.5 6.0 5.5 5.0 4.5 4.0 3.5 3.0 2.5 2.0 1.5 1.0 0.5 0.0 -0.5
f1 (ppm)

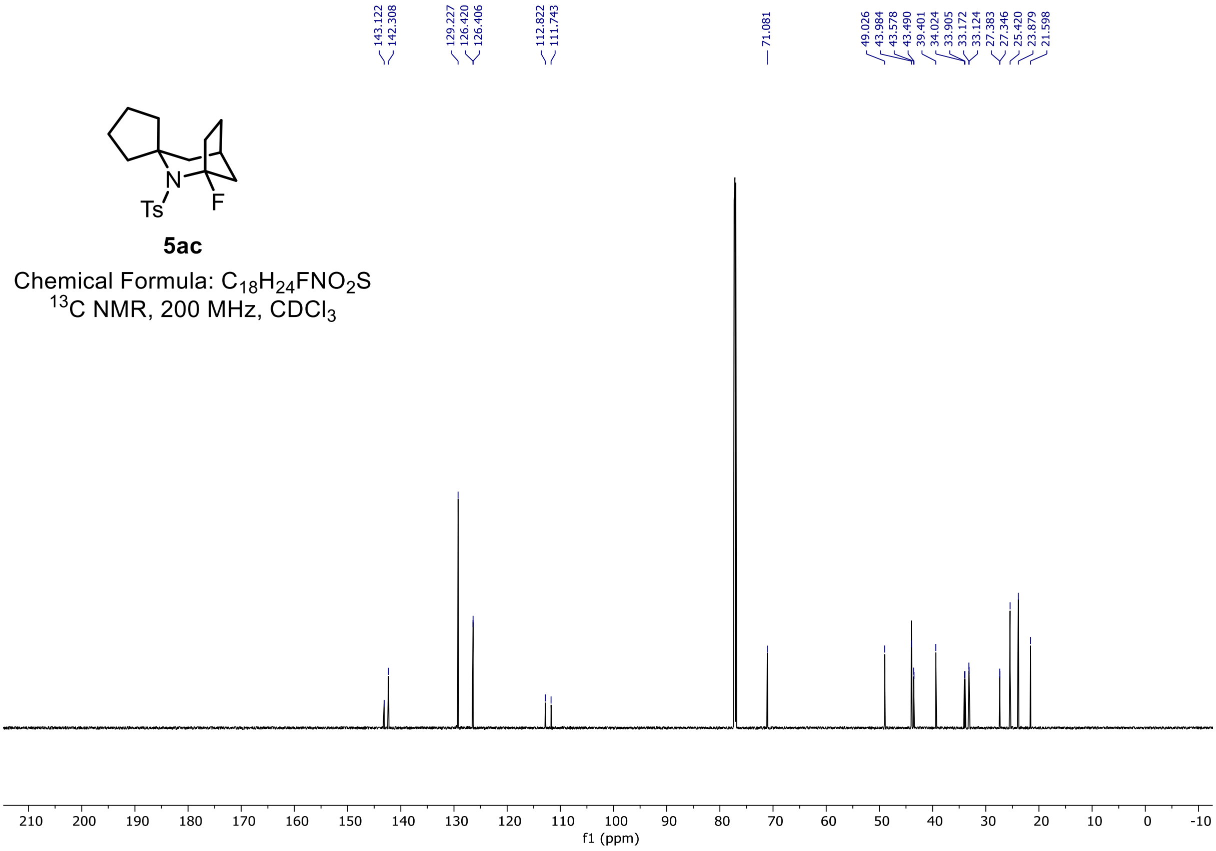

143.122 142.308 129.227 126.420 126.406 112.822 111.743 71.081 49.026 43.984 43.578 43.490 39.401 34.024 33.905 33.172 33.124 27.383 27.346 25.420 23.879 21.598
N
Ts
F
5ac
Chemical Formula: $C_{18}H_{24}FNO_2S$
^{13}C NMR, 200 MHz, $CDCl_3$
210 200 190 180 170 160 150 140 130 120 110 100 90 80 70 60 50 40 30 20 10 0 -10
f1 (ppm)

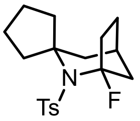

5ac

Chemical Formula: $C_{18}H_{24}FNO_2S$

^{19}F NMR, 376 MHz, $CDCl_3$

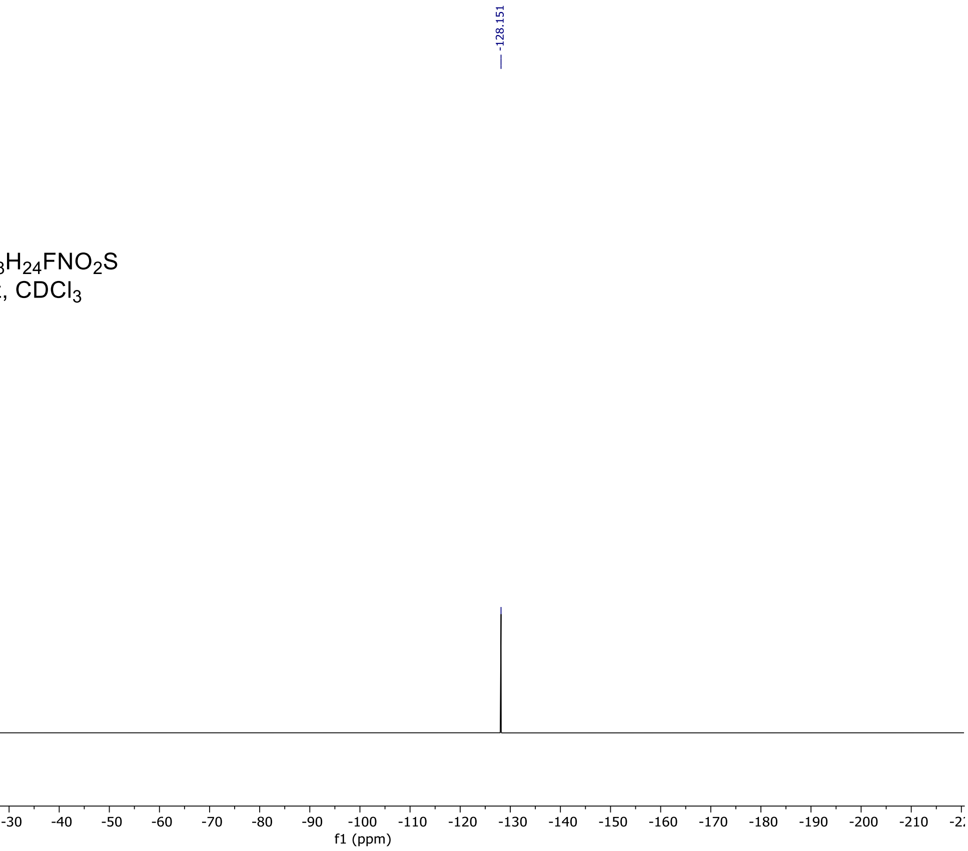

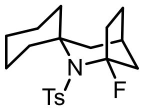

5ad

Chemical Formula: $C_{19}H_{26}FNO_2S$

^{1}H NMR, 400 MHz, $CDCl_3$

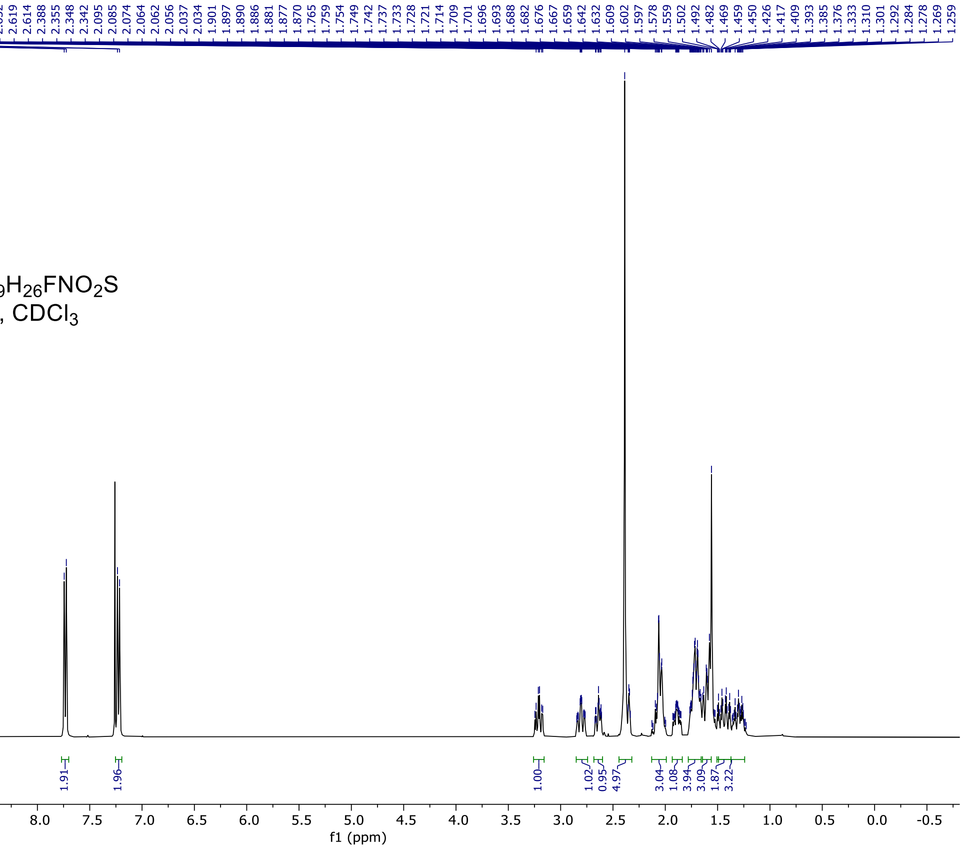

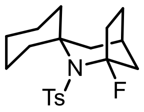

5ad

Chemical Formula: $C_{19}H_{26}FNO_2S$

^{13}C NMR, 100 MHz, $CDCl_3$

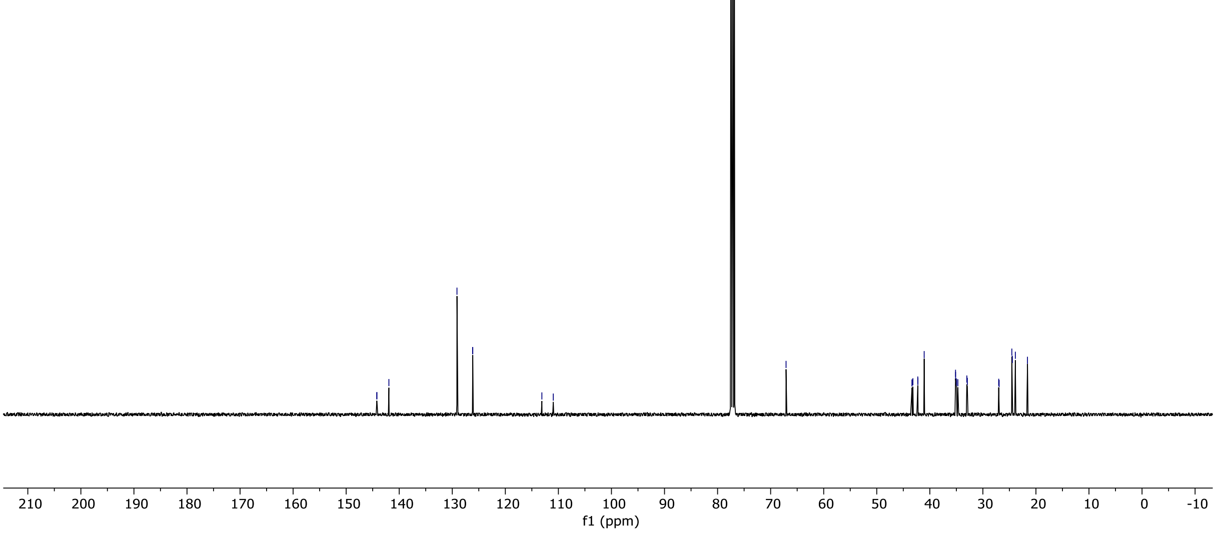

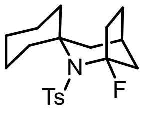

5ad

Chemical Formula: $C_{19}H_{26}FNO_2S$

^{19}F NMR, 376 MHz, $CDCl_3$

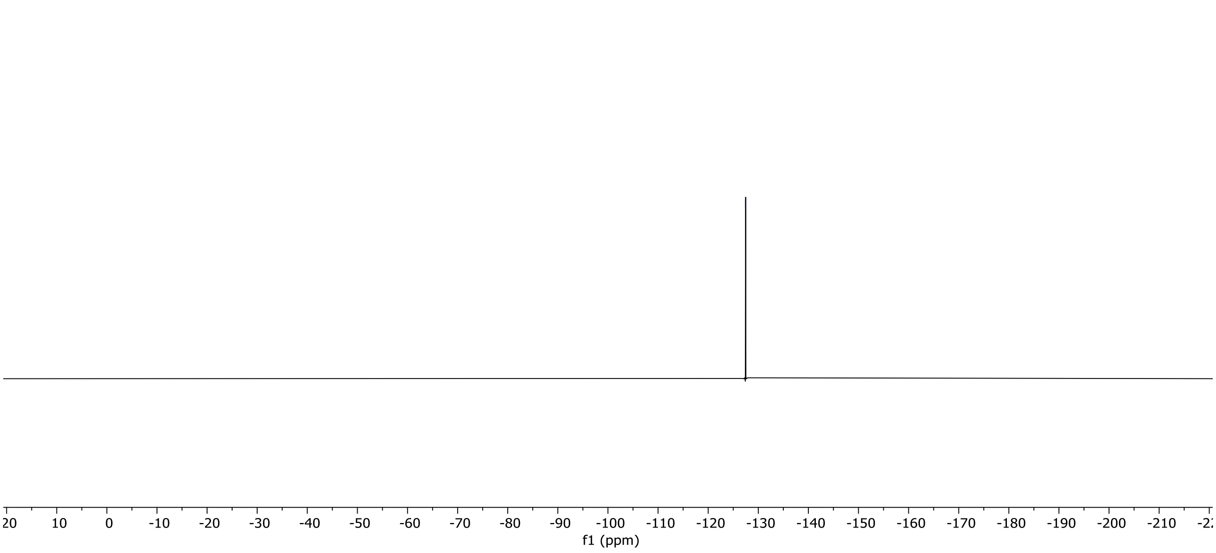

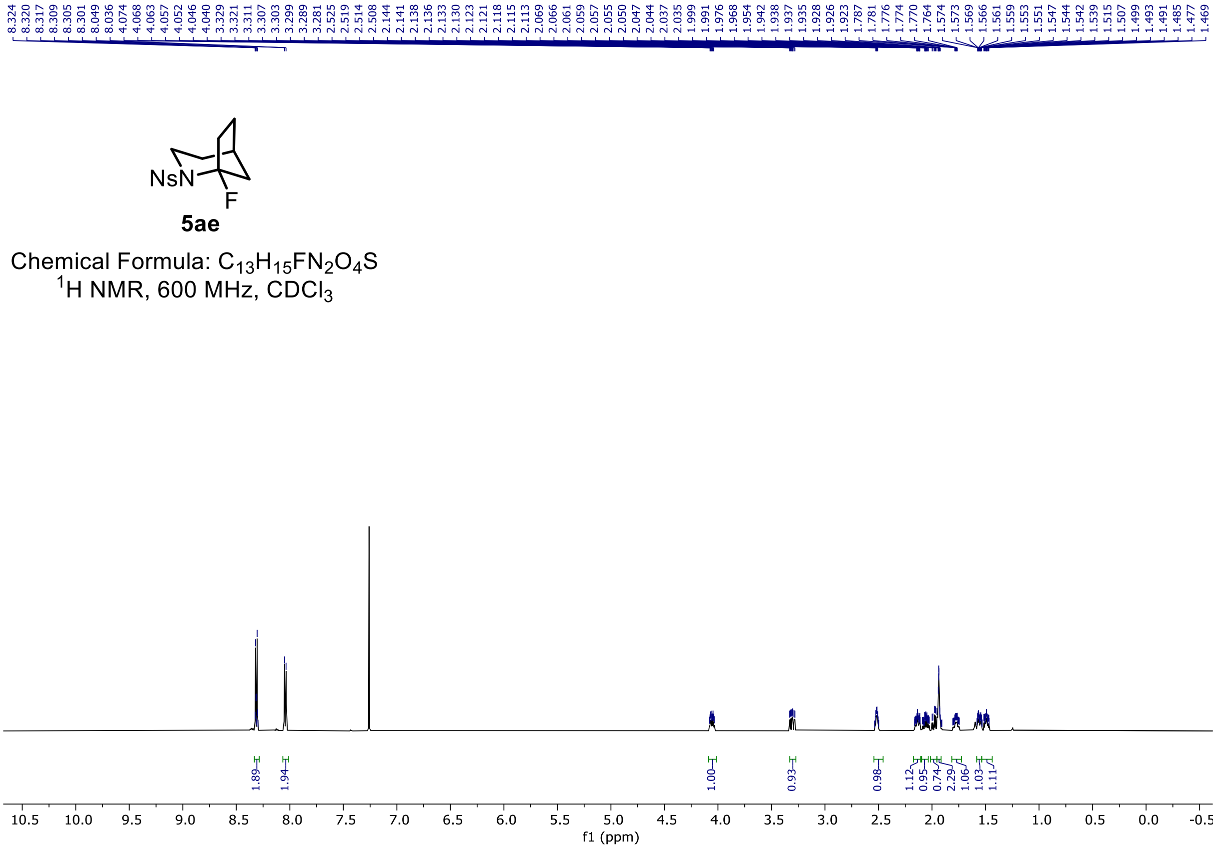
8.324 8.320 8.317 8.309 8.305 8.301 8.049 8.036 4.074 4.068 4.063 4.057 4.052 4.046 4.040 3.329 3.321 3.311 3.307 3.303 3.299 3.289 3.281 2.525 2.519 2.514 2.508 2.144 2.141 2.138 2.136 2.133 2.130 2.123 2.121 2.118 2.115 2.113 2.069 2.066 2.061 2.059 2.057 2.055 2.050 2.047 2.044 2.037 2.035 1.999 1.991 1.976 1.968 1.954 1.942 1.938 1.937 1.935 1.928 1.926 1.923 1.787 1.781 1.776 1.770 1.764 1.574 1.573 1.569 1.566 1.561 1.559 1.553 1.551 1.547 1.544 1.542 1.539 1.515 1.507 1.499 1.493 1.491 1.485 1.477 1.469
NsN
F
5ae
Chemical Formula: C13H15FN2O4S
1H NMR, 600 MHz, CDCl3
1.89 1.94 1.00 0.93 0.98 1.12 0.95 0.74 2.29 1.06 1.03 1.11
10.5 10.0 9.5 9.0 8.5 8.0 7.5 7.0 6.5 6.0 5.5 5.0 4.5 4.0 3.5 3.0 2.5 2.0 1.5 1.0 0.5 0.0 -0.5
f1 (ppm)

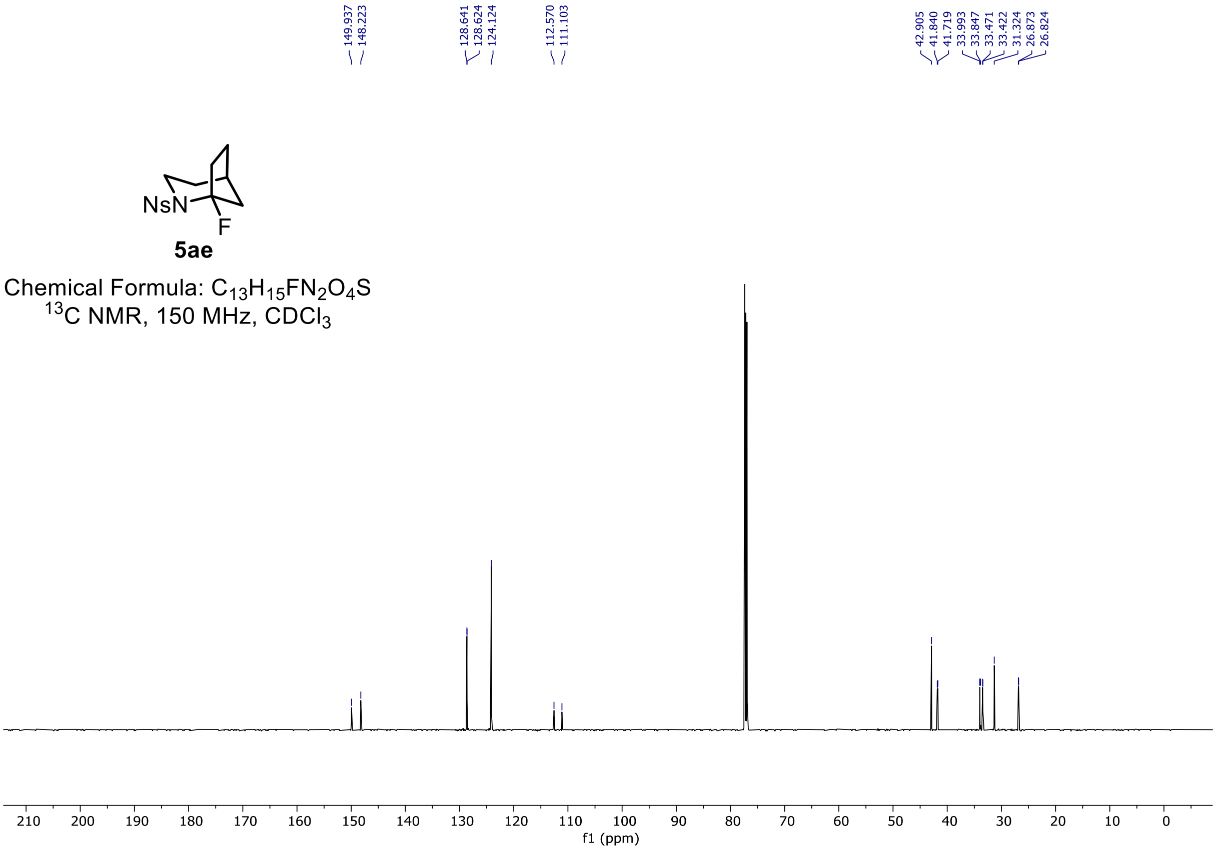
149.937 148.223 128.641 128.624 124.124 112.570 111.103 42.905 41.840 41.719 33.993 33.847 33.471 33.422 31.324 26.873 26.824
NsN
F
5ae
Chemical Formula: C13H15FN2O4S
13C NMR, 150 MHz, CDCl3
210 200 190 180 170 160 150 140 130 120 110 100 90 80 70 60 50 40 30 20 10 0
f1 (ppm)

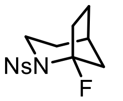

5ae

Chemical Formula: $C_{13}H_{15}FN_2O_4S$

^{19}F NMR, 376 MHz, $CDCl_3$

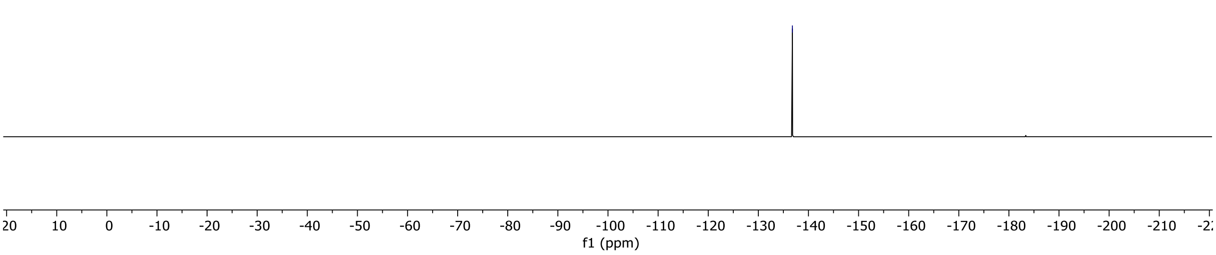

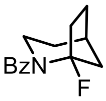

5af

Chemical Formula: $C_{14}H_{16}FNO$

^{1}H NMR, 600 MHz, $CDCl_3$

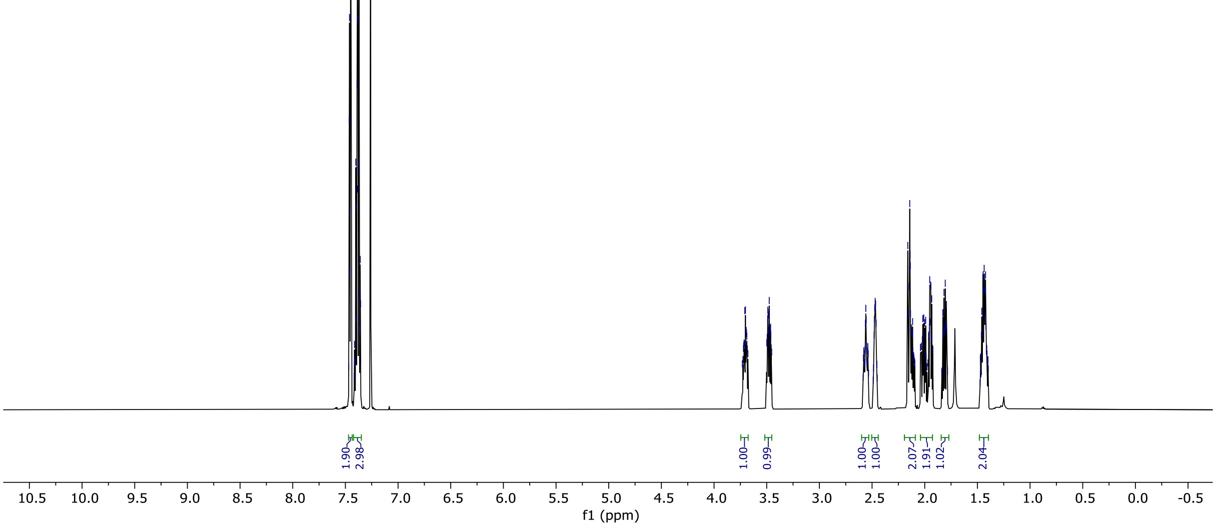

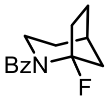

5af

Chemical Formula: $C_{14}H_{16}FNO$

^{13}C NMR, 150 MHz, $CDCl_3$

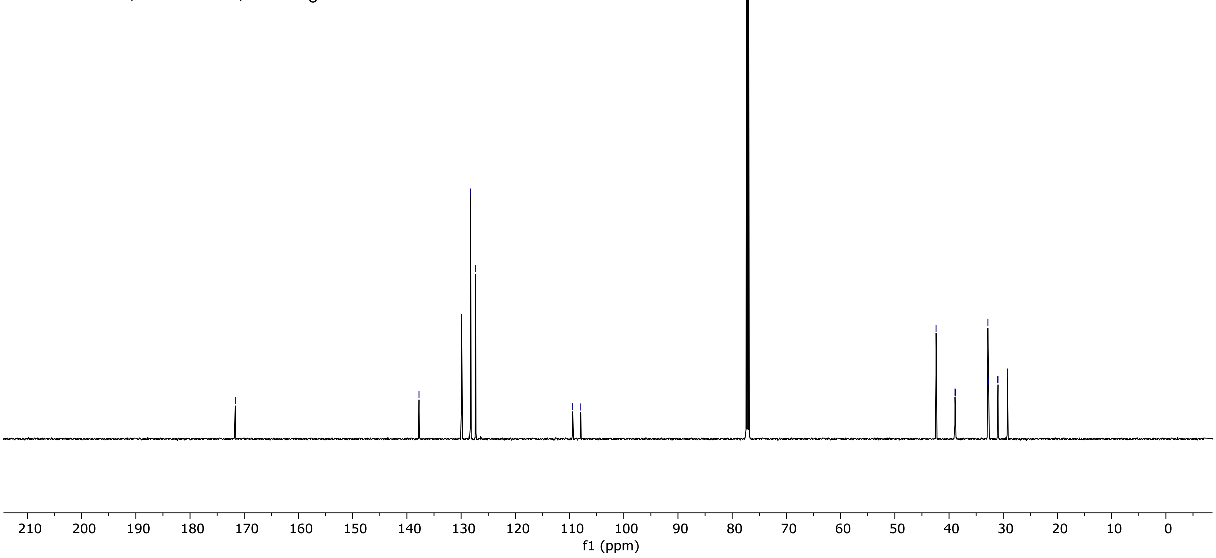

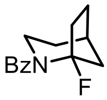

5af

Chemical Formula: $C_{14}H_{16}FNO$

^{19}F NMR, 376 MHz, $CDCl_3$

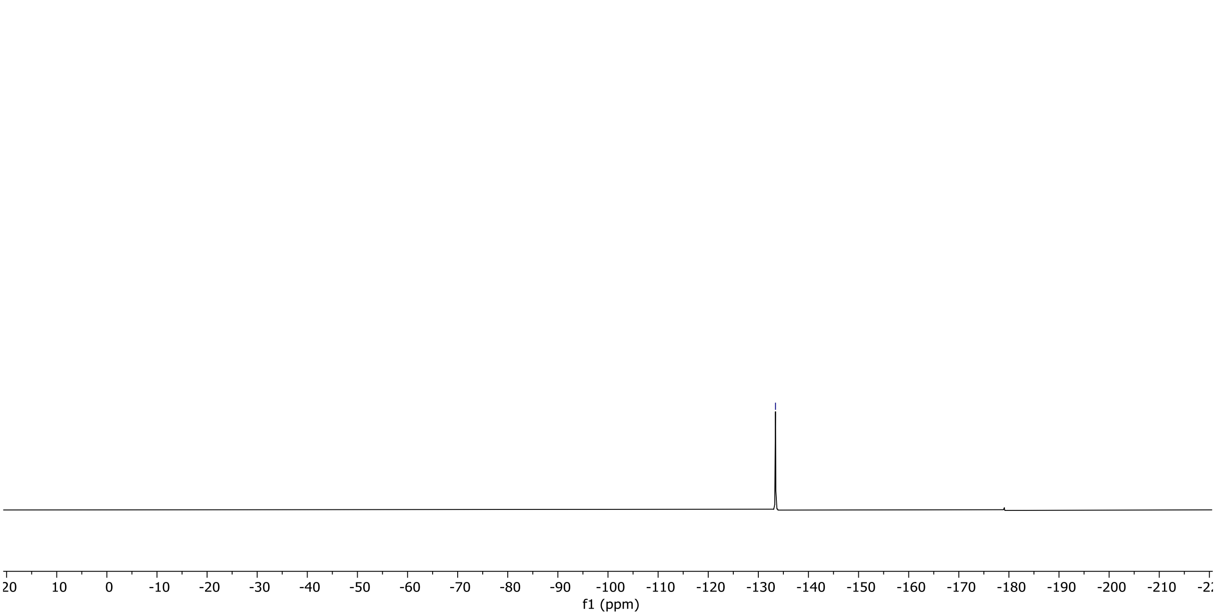

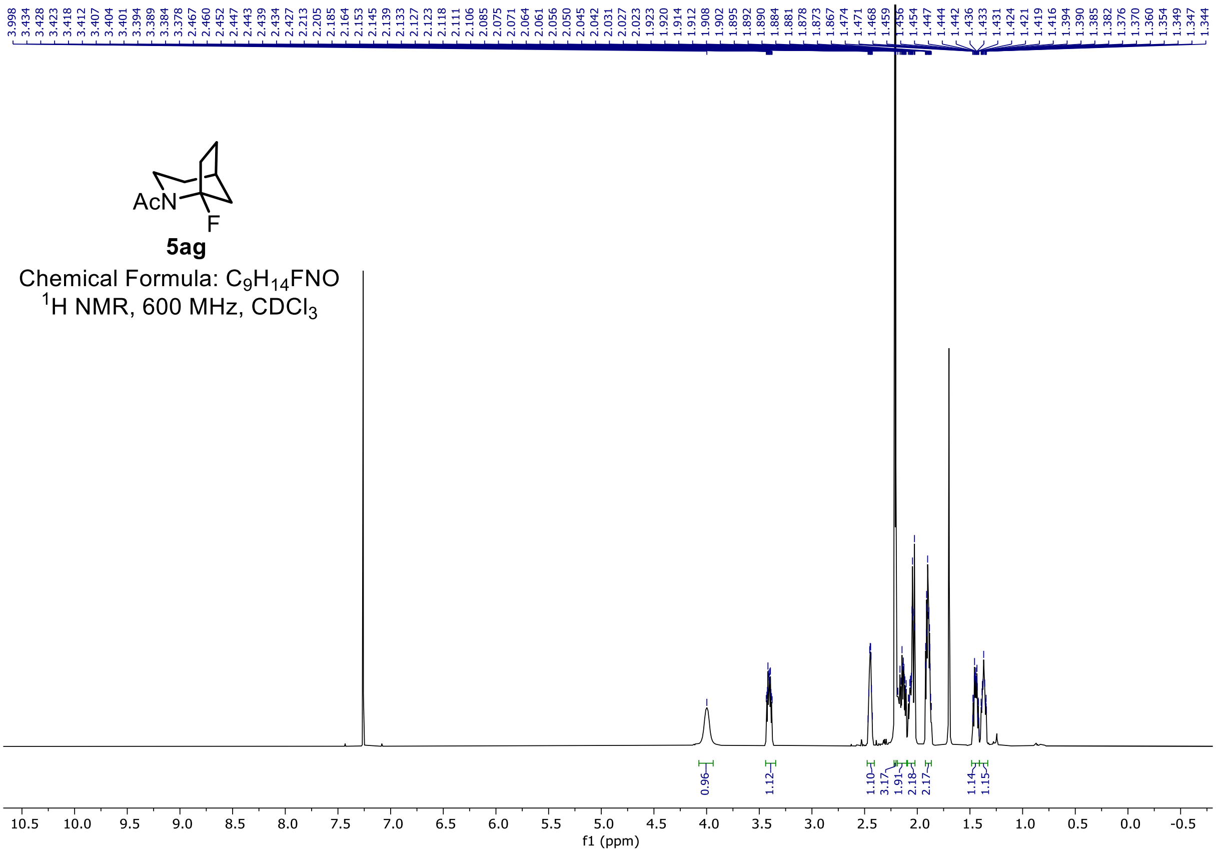
3.998 3.434 3.428 3.423 3.418 3.412 3.407 3.404 3.401 3.394 3.389 3.384 3.378 2.467 2.460 2.452 2.447 2.443 2.439 2.434 2.427 2.213 2.205 2.185 2.164 2.153 2.145 2.139 2.133 2.127 2.123 2.118 2.111 2.106 2.085 2.075 2.071 2.064 2.061 2.056 2.050 2.045 2.042 2.031 2.027 2.023 1.923 1.920 1.914 1.912 1.908 1.902 1.895 1.892 1.890 1.884 1.881 1.878 1.873 1.867 1.474 1.471 1.468 1.459 1.456 1.454 1.447 1.444 1.442 1.436 1.433 1.431 1.424 1.421 1.419 1.416 1.394 1.390 1.385 1.382 1.376 1.370 1.360 1.354 1.349 1.347 1.344
AcN
F
5ag
Chemical Formula: C9H14FNO
1H NMR, 600 MHz, CDCl3
0.96
1.12
1.10
3.17
1.91
2.18
2.17
1.14
1.15
10.5 10.0 9.5 9.0 8.5 8.0 7.5 7.0 6.5 6.0 5.5 5.0 4.5 4.0 3.5 3.0 2.5 2.0 1.5 1.0 0.5 0.0 -0.5
f1 (ppm)

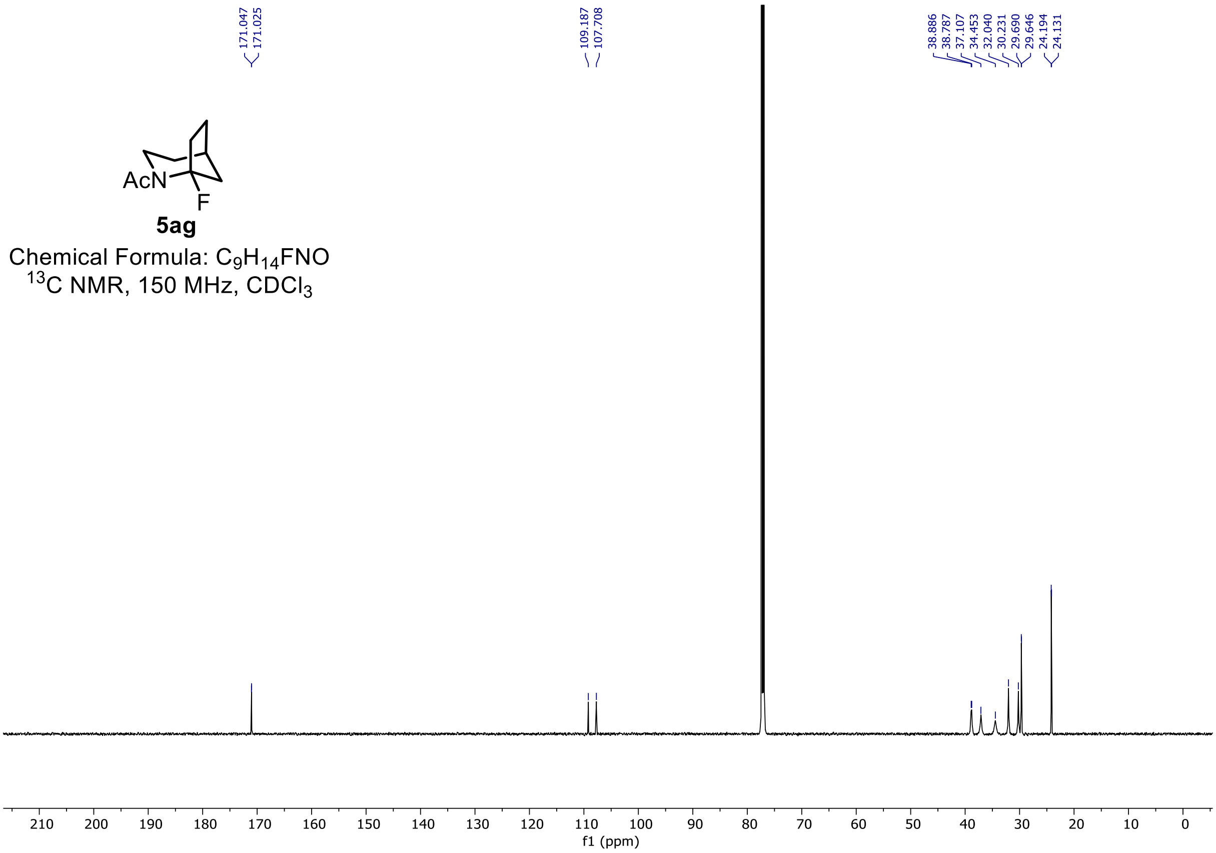
171.047 171.025
109.187 107.708
38.886 38.787 37.107 34.453 32.040 30.231 29.690 29.646 24.194 24.131
AcN
F
5ag
Chemical Formula: C9H14FNO
13C NMR, 150 MHz, CDCl3
210 200 190 180 170 160 150 140 130 120 110 100 90 80 70 60 50 40 30 20 10 0
f1 (ppm)

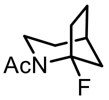

5ag

Chemical Formula: $C_9H_{14}FNO$

^{19}F NMR, 376 MHz, $CDCl_3$

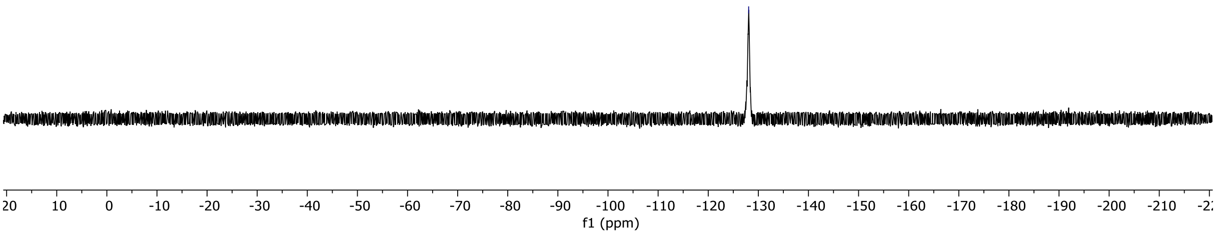

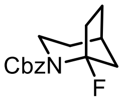

5ah

Chemical Formula: $C_{15}H_{18}FNO_2$

^{1}H NMR, 600 MHz, $CDCl_3$

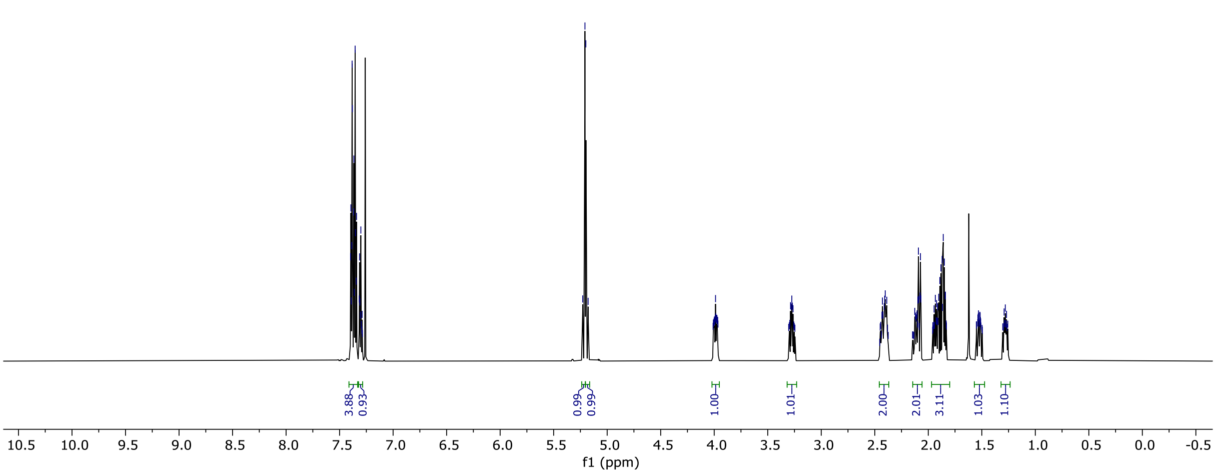

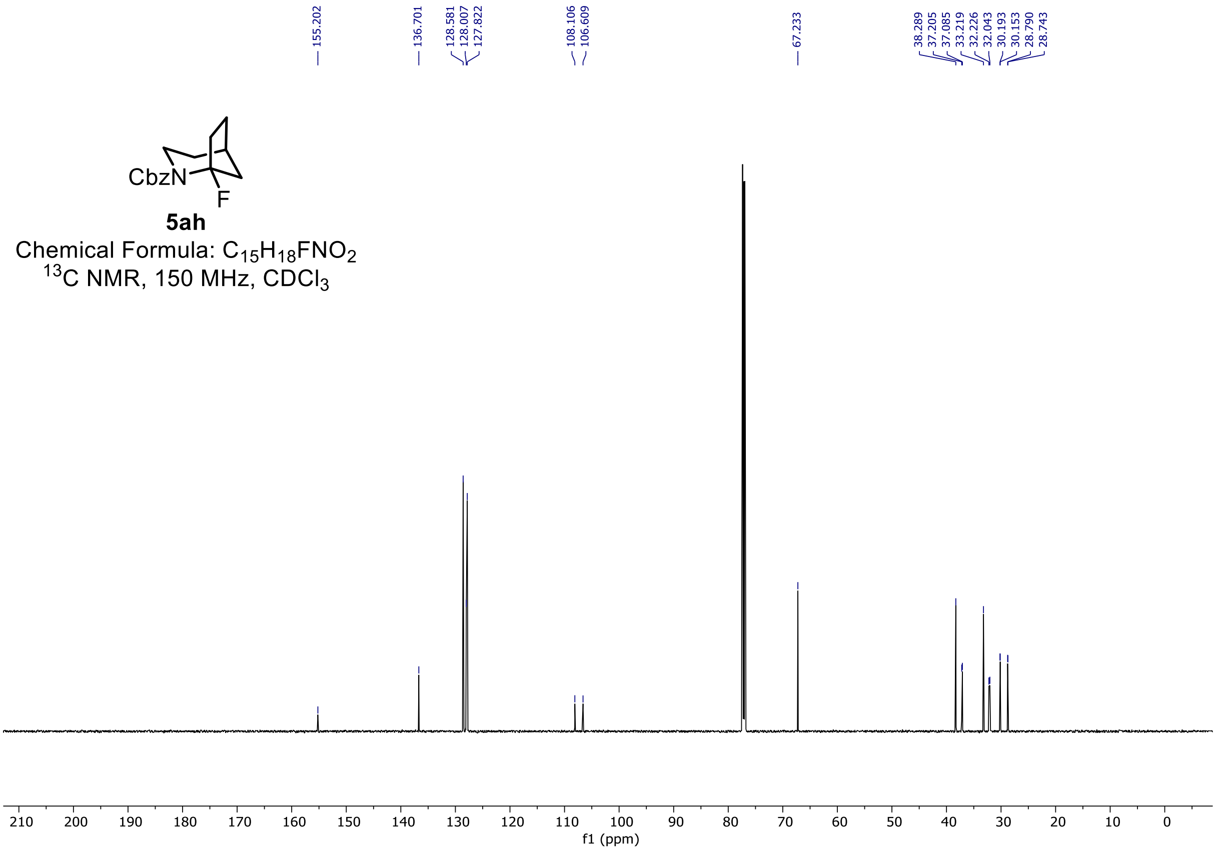

5ah
Chemical Formula: $C_{15}H_{18}FNO_2$
^{13}C NMR, 150 MHz, $CDCl_3$

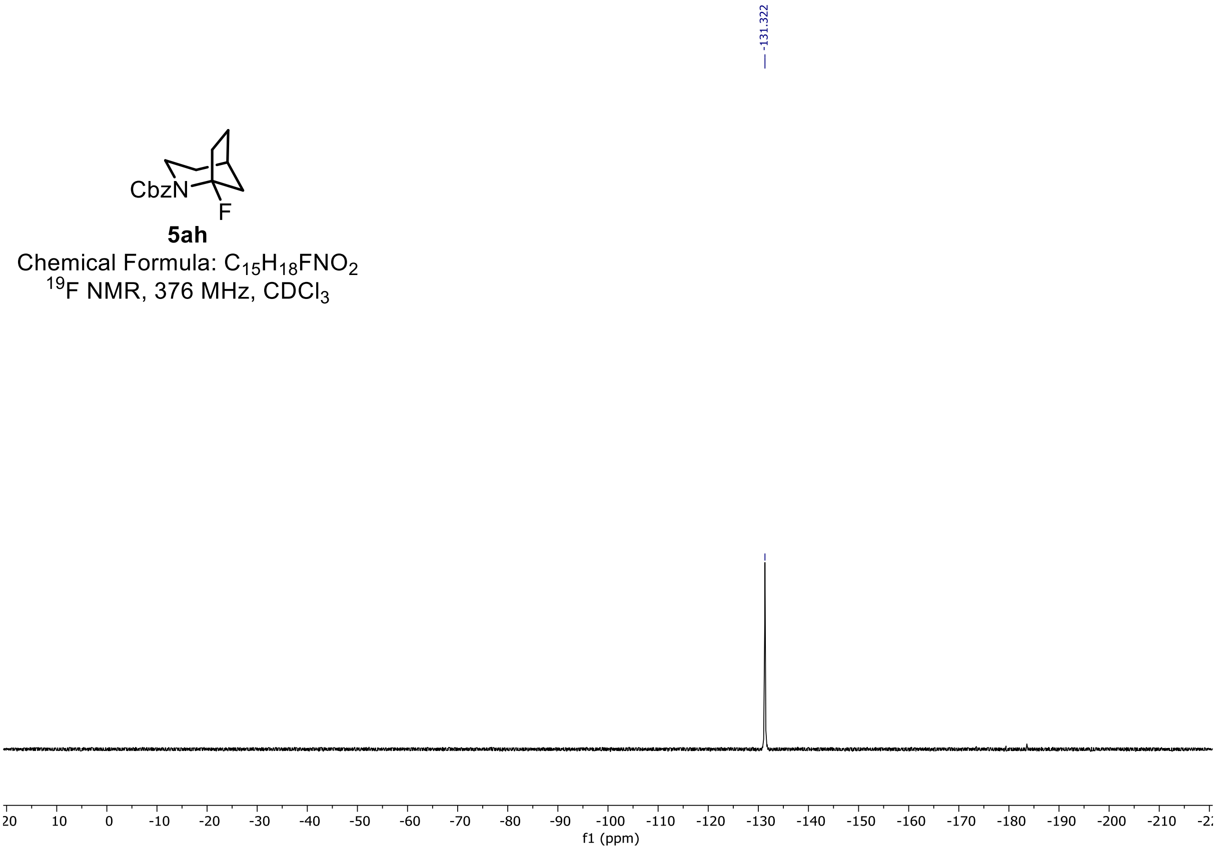

5ah
Chemical Formula: $C_{15}H_{18}FNO_2$
^{19}F NMR, 376 MHz, $CDCl_3$

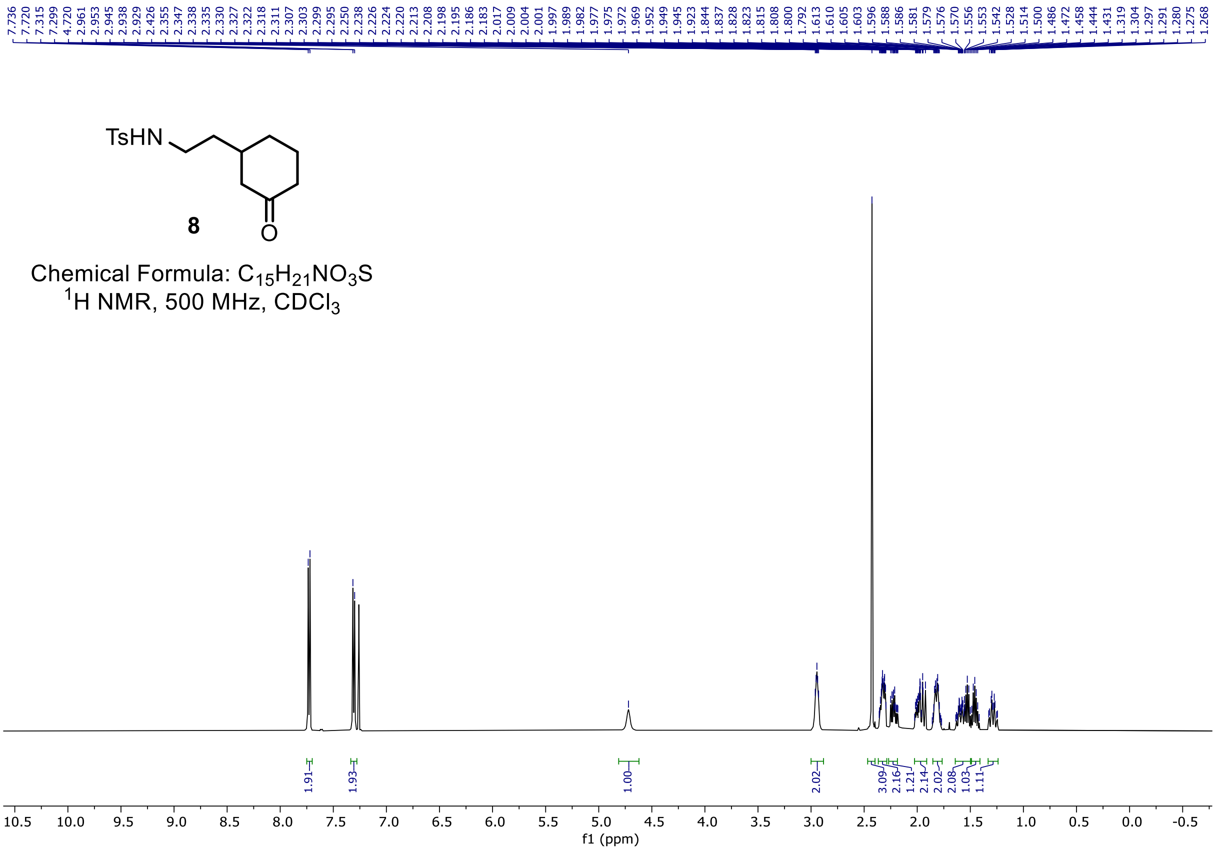
7.736 7.720 7.315 7.299 4.720 2.961 2.953 2.945 2.938 2.929 2.426 2.355 2.347 2.338 2.335 2.330 2.327 2.322 2.318 2.311 2.307 2.303 2.299 2.295 2.250 2.238 2.226 2.224 2.220 2.213 2.208 2.198 2.195 2.186 2.183 2.017 2.009 2.004 2.001 1.997 1.989 1.982 1.977 1.975 1.972 1.969 1.952 1.949 1.945 1.923 1.844 1.837 1.828 1.823 1.815 1.808 1.800 1.792 1.613 1.610 1.605 1.603 1.596 1.588 1.586 1.581 1.579 1.576 1.570 1.556 1.553 1.542 1.528 1.514 1.500 1.486 1.472 1.458 1.444 1.431 1.319 1.304 1.297 1.291 1.280 1.275 1.268
TsHN
8
O
Chemical Formula: $C_{15}H_{21}NO_3S$
^{1}H NMR, 500 MHz, $CDCl_3$
1.91 1.93 1.00 2.02 3.09 2.16 1.21 2.14 2.02 2.08 1.03 1.11
10.5 10.0 9.5 9.0 8.5 8.0 7.5 7.0 6.5 6.0 5.5 5.0 4.5 4.0 3.5 3.0 2.5 2.0 1.5 1.0 0.5 0.0 -0.5
f1 (ppm)

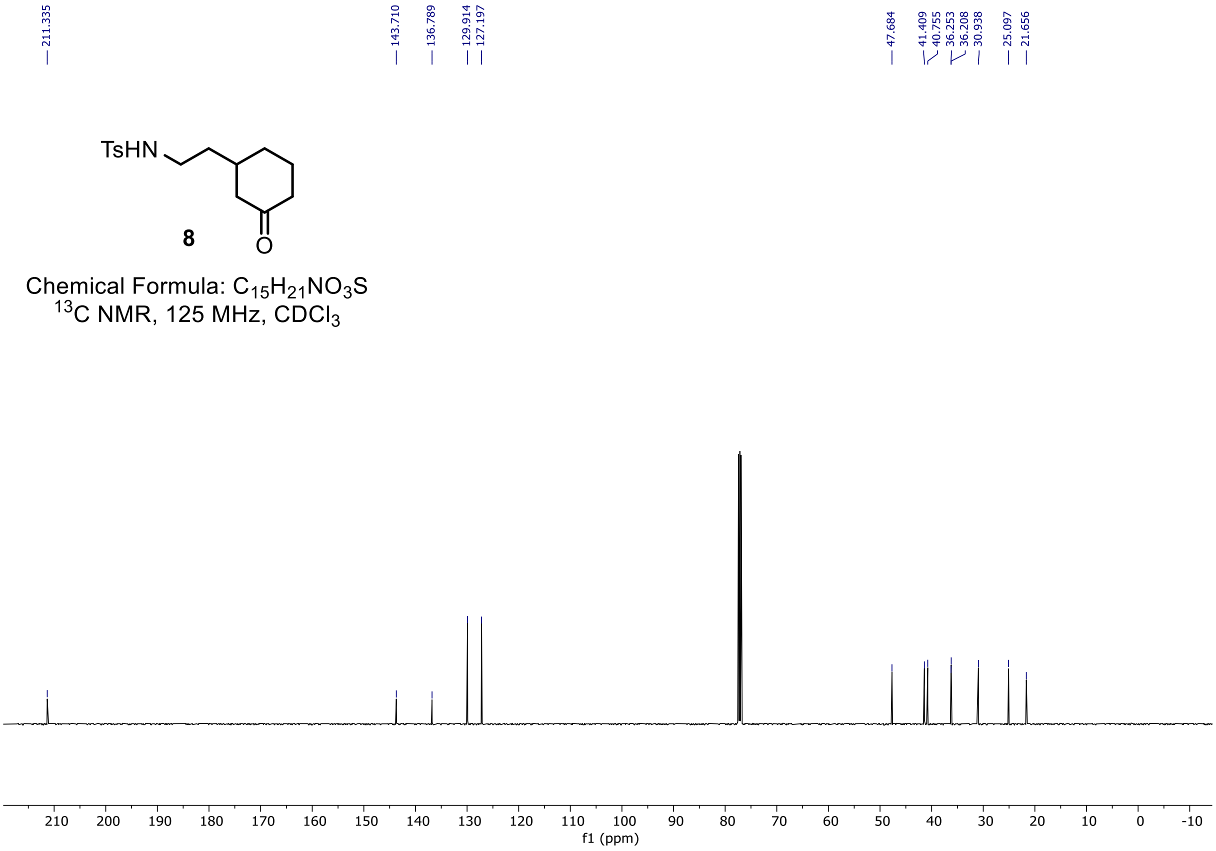
211.335
143.710 136.789 129.914 127.197
47.684 41.409 40.755 36.253 36.208 30.938 25.097 21.656
TsHN
8
O
Chemical Formula: $C_{15}H_{21}NO_3S$
^{13}C NMR, 125 MHz, $CDCl_3$
210 200 190 180 170 160 150 140 130 120 110 100 90 80 70 60 50 40 30 20 10 0 -10
f1 (ppm)

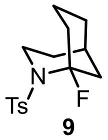

9

Chemical Formula: $C_{15}H_{20}FNO_2S$
^{1}H NMR, 600 MHz, $CDCl_3$

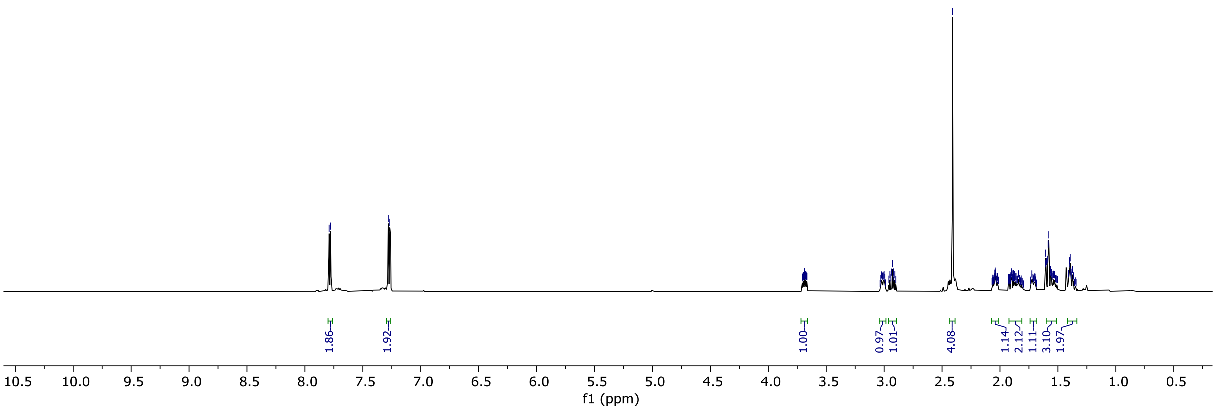

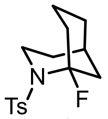

9

Chemical Formula: $C_{15}H_{20}FNO_2S$
^{13}C NMR, 150 MHz, $CDCl_3$

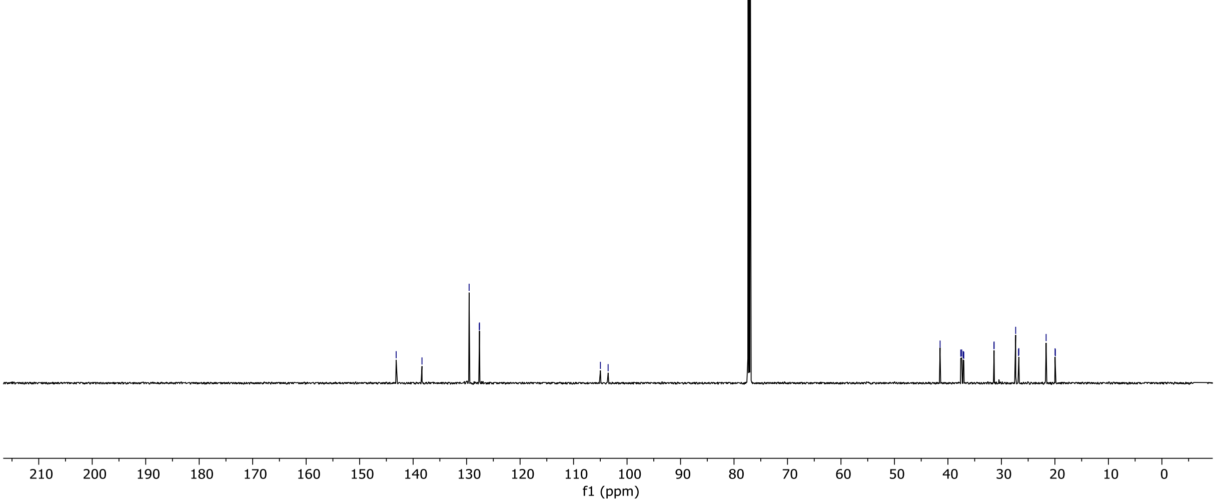

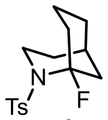

9

Chemical Formula: $C_{15}H_{20}FNO_2S$

^{19}F NMR, 376 MHz, $CDCl_3$

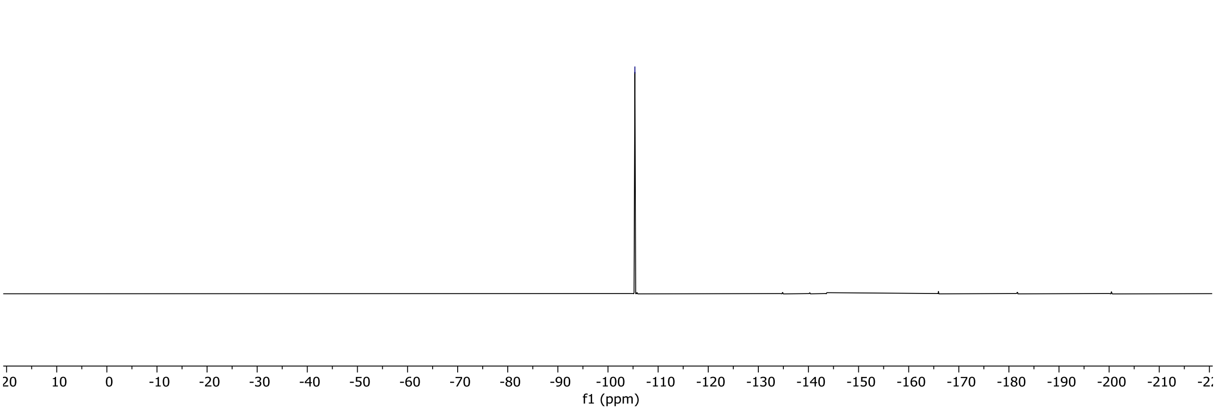

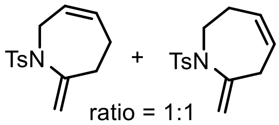

10a **10b**

Chemical Formula: $C_{14}H_{17}NO_2S$

^{1}H NMR, 500 MHz, $CDCl_3$

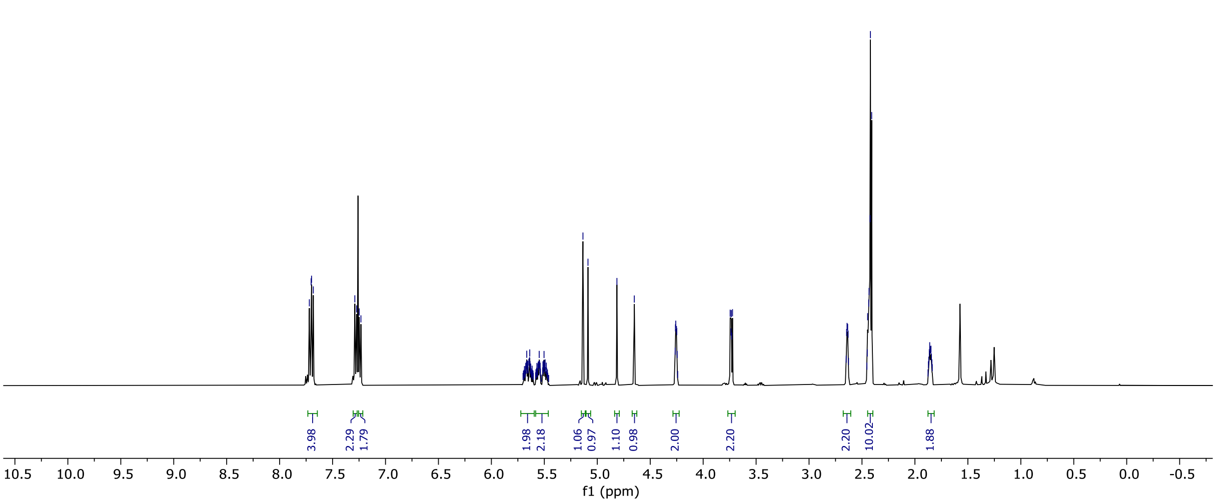

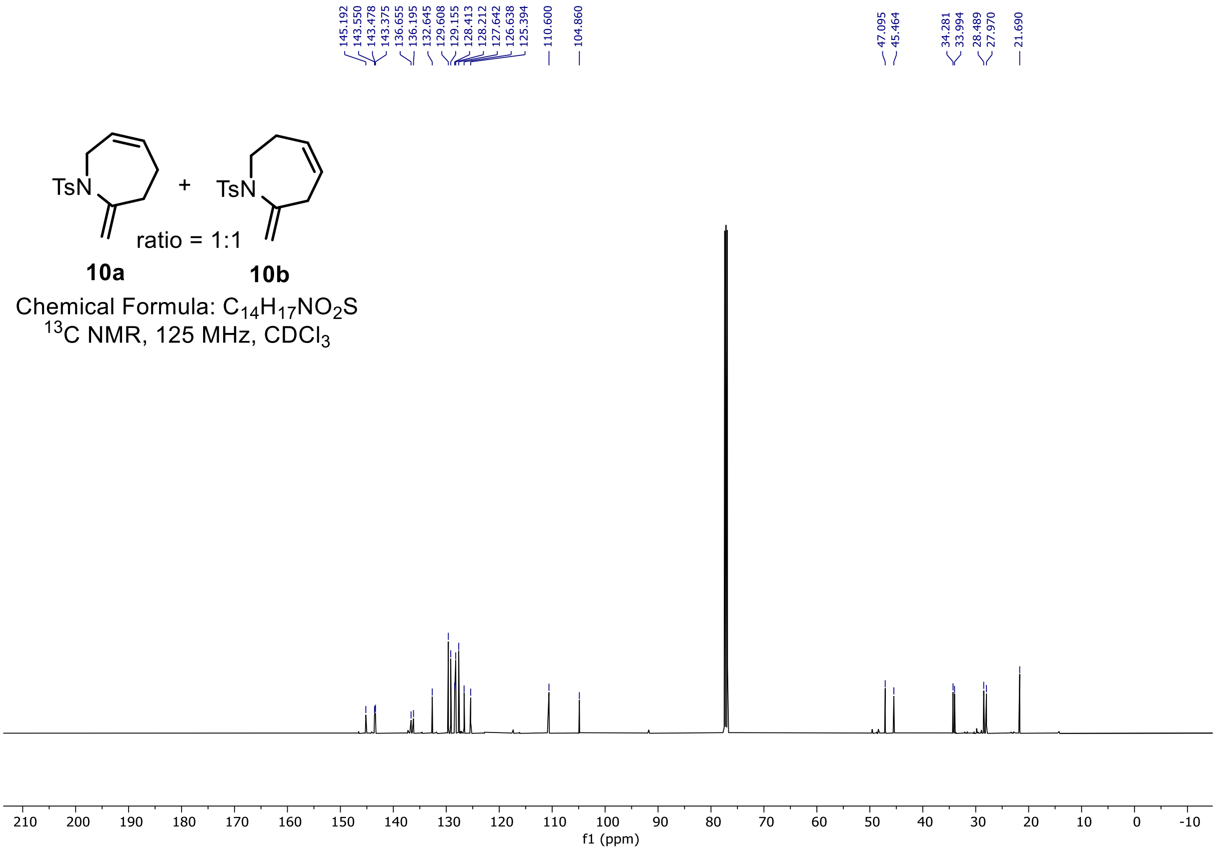
TsN
+
TsN
ratio = 1:1
10a
10b
Chemical Formula: $C_{14}H_{17}NO_2S$
^{13}C NMR, 125 MHz, $CDCl_3$
f1 (ppm)

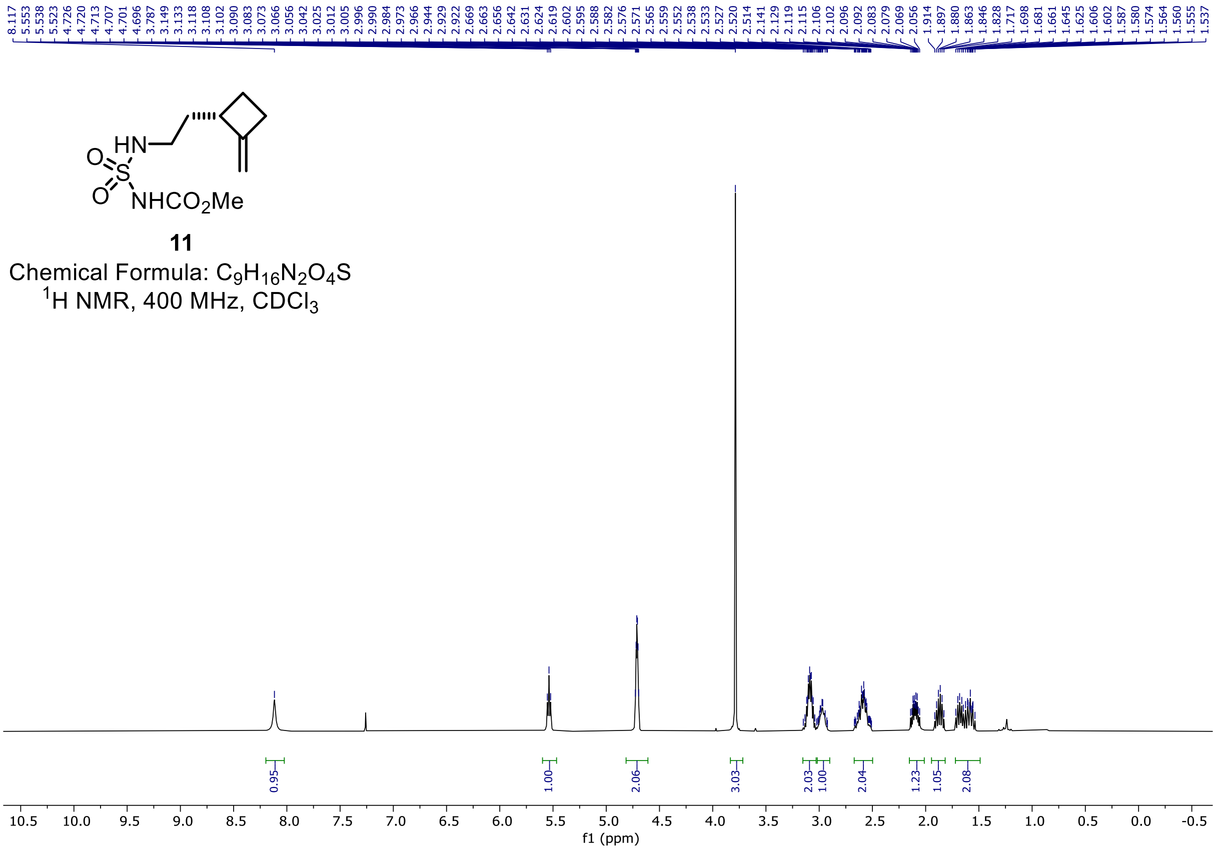
HN
NHCO2Me
11
Chemical Formula: $C_9H_{16}N_2O_4S$
^{1}H NMR, 400 MHz, $CDCl_3$
f1 (ppm)

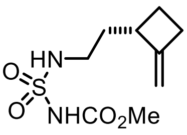

11

Chemical Formula: $C_9H_{16}N_2O_4S$

^{13}C NMR, 100 MHz, $CDCl_3$

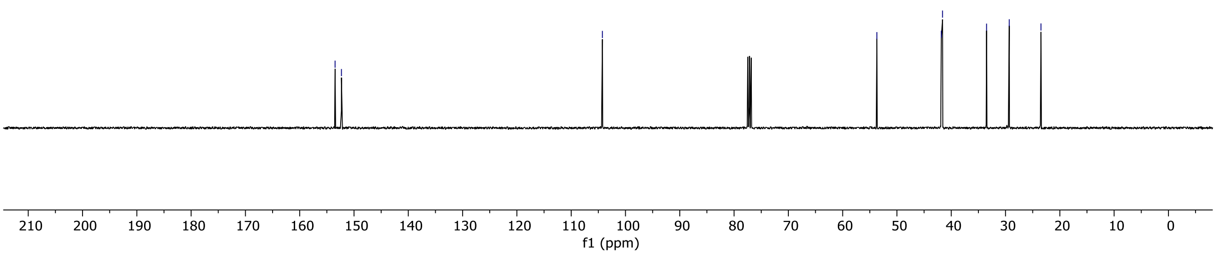

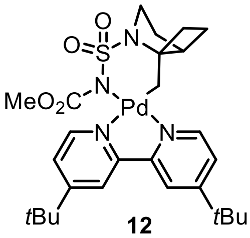

12

Chemical Formula: $C_{27}H_{38}N_4O_4PdS$

^{1}H NMR, 600 MHz, $CDCl_3$

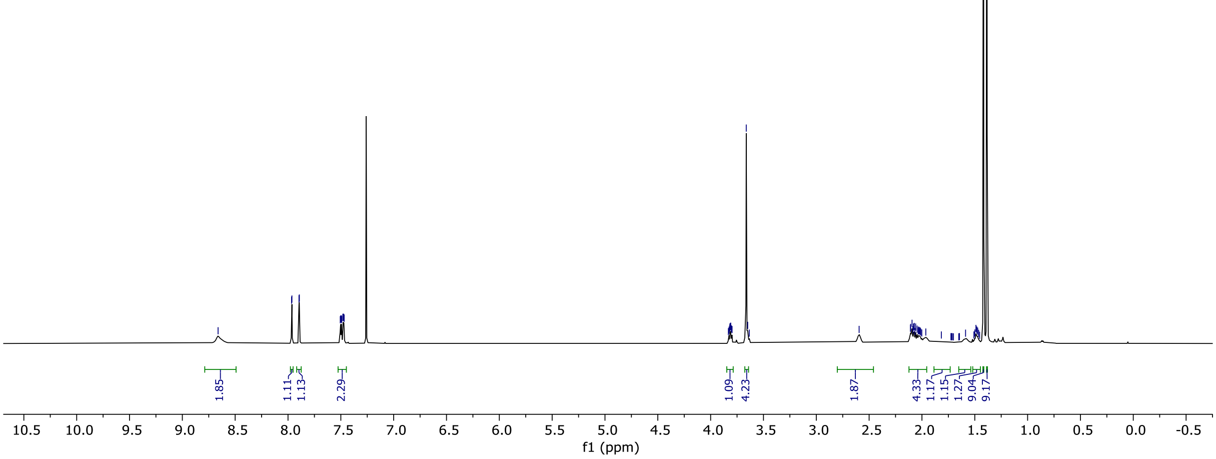

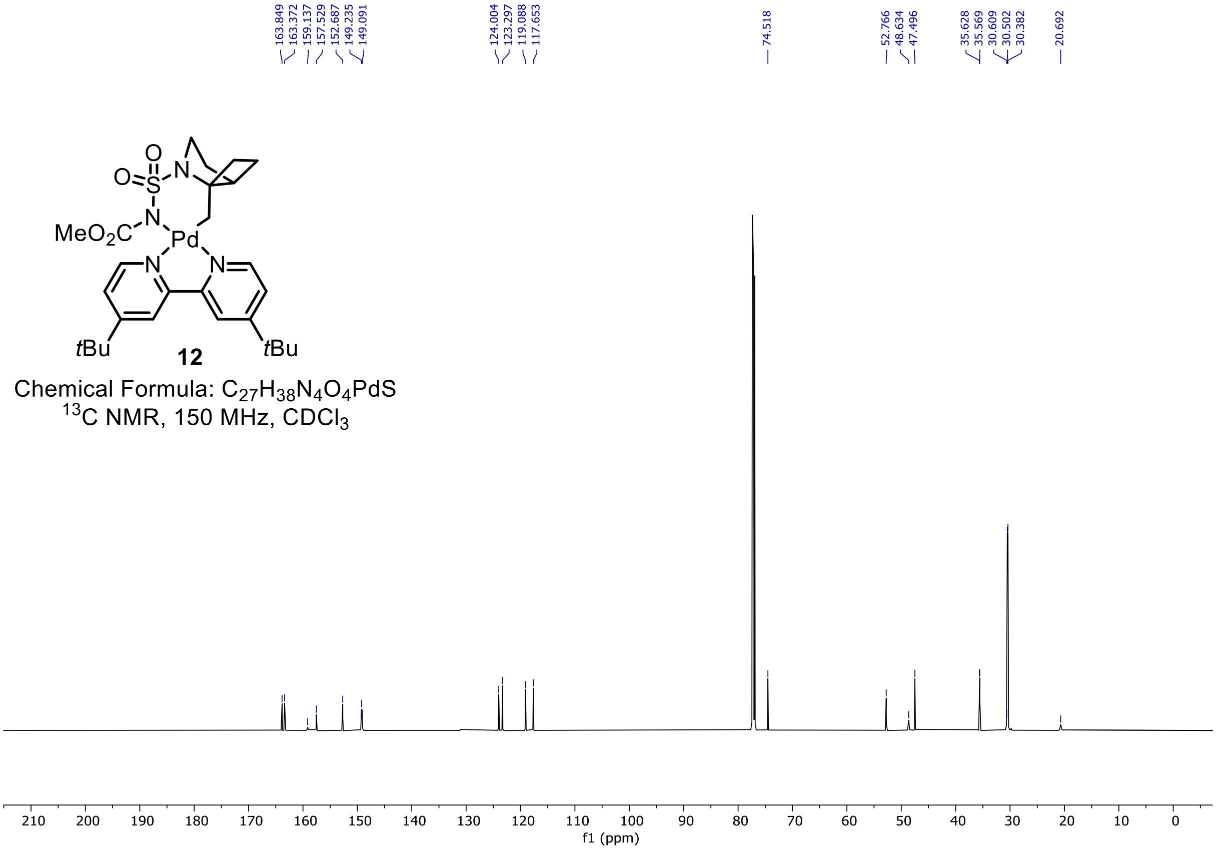
163.849
163.372
159.137
157.529
152.687
149.235
149.091
124.004
123.297
119.088
117.653
74.518
52.766
48.634
47.496
35.628
35.569
30.609
30.502
30.382
20.692
MeO2C
Pd
tBu
tBu
12
Chemical Formula: C27H38N4O4PdS
13C NMR, 150 MHz, CDCl3
210 200 190 180 170 160 150 140 130 120 110 100 90 80 70 60 50 40 30 20 10 0
f1 (ppm)

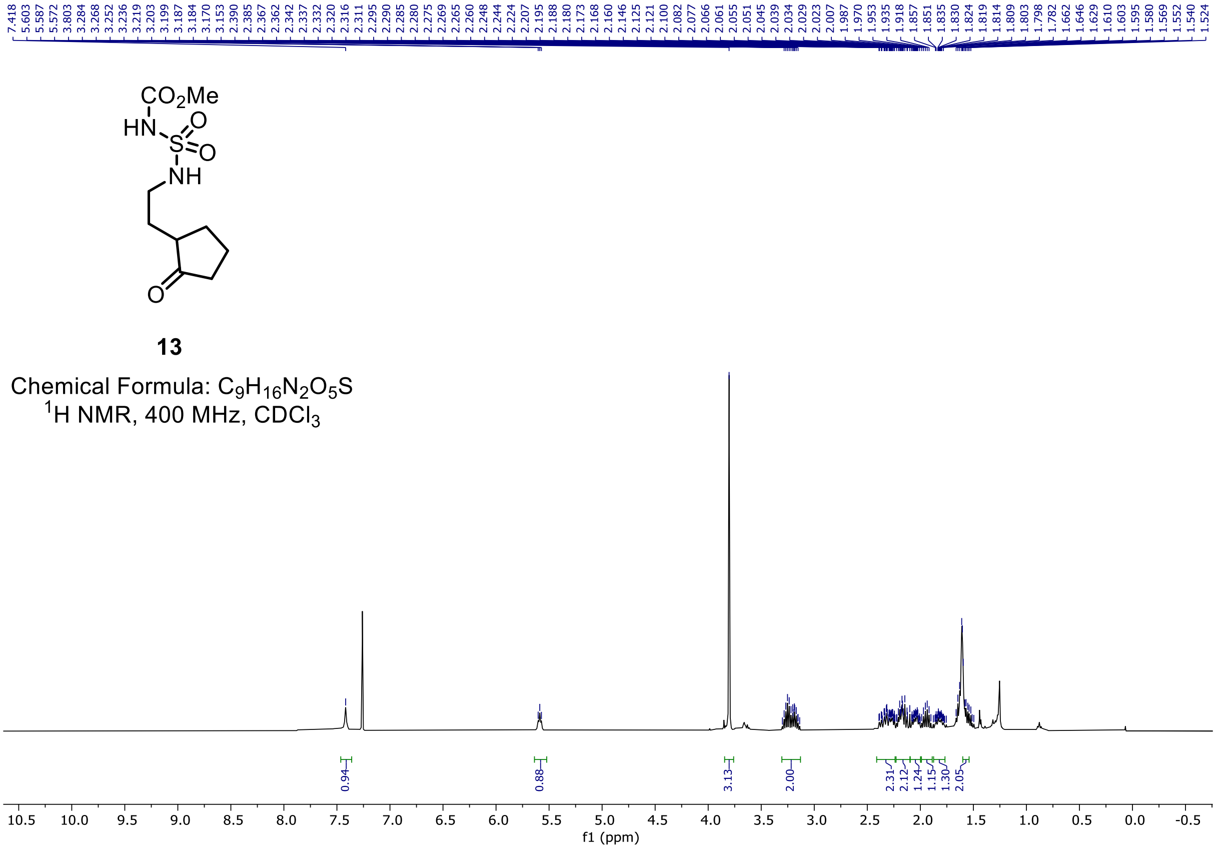
7.418
5.603
5.587
5.572
3.803
3.284
3.268
3.252
3.236
3.219
3.203
3.199
3.187
3.184
3.170
3.153
2.390
2.385
2.367
2.362
2.342
2.337
2.332
2.320
2.316
2.311
2.295
2.290
2.285
2.280
2.275
2.269
2.265
2.260
2.248
2.244
2.224
2.207
2.195
2.188
2.180
2.173
2.168
2.160
2.146
2.125
2.121
2.100
2.082
2.077
2.066
2.061
2.055
2.051
2.045
2.039
2.034
2.029
2.023
2.007
1.987
1.970
1.953
1.935
1.918
1.857
1.851
1.835
1.830
1.824
1.819
1.814
1.809
1.803
1.798
1.782
1.662
1.646
1.629
1.610
1.603
1.595
1.580
1.569
1.552
1.540
1.524
CO2Me
HN
NH
13
Chemical Formula: C9H16N2O5S
1H NMR, 400 MHz, CDCl3
0.94
0.88
3.13
2.00
2.31
2.12
1.24
1.15
1.30
2.05
10.5 10.0 9.5 9.0 8.5 8.0 7.5 7.0 6.5 6.0 5.5 5.0 4.5 4.0 3.5 3.0 2.5 2.0 1.5 1.0 0.5 0.0 -0.5
f1 (ppm)

13

Chemical Formula: $C_9H_{16}N_2O_5S$

^{13}C NMR, 150 MHz, $CDCl_3$

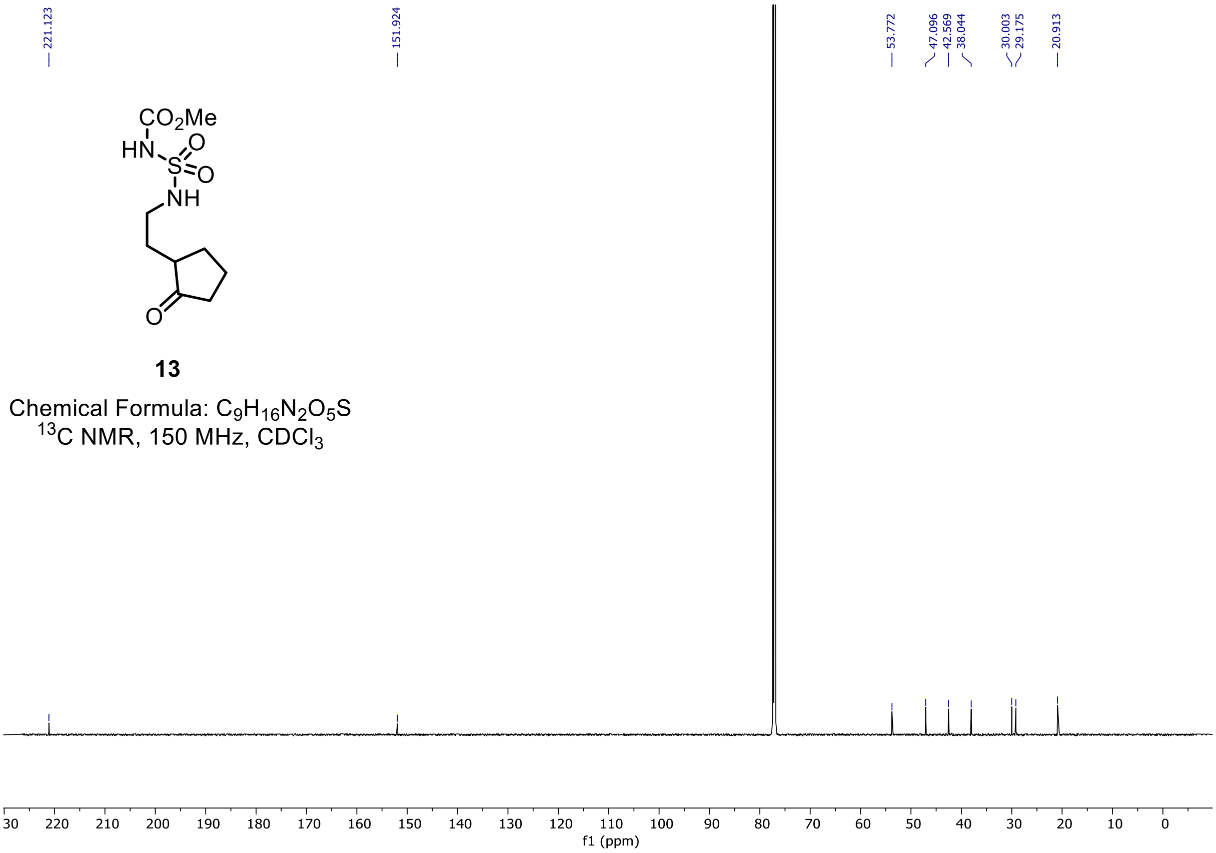

14

Chemical Formula: $C_9H_{16}N_2O_5S$

^{1}H NMR, 500 MHz, $CDCl_3$

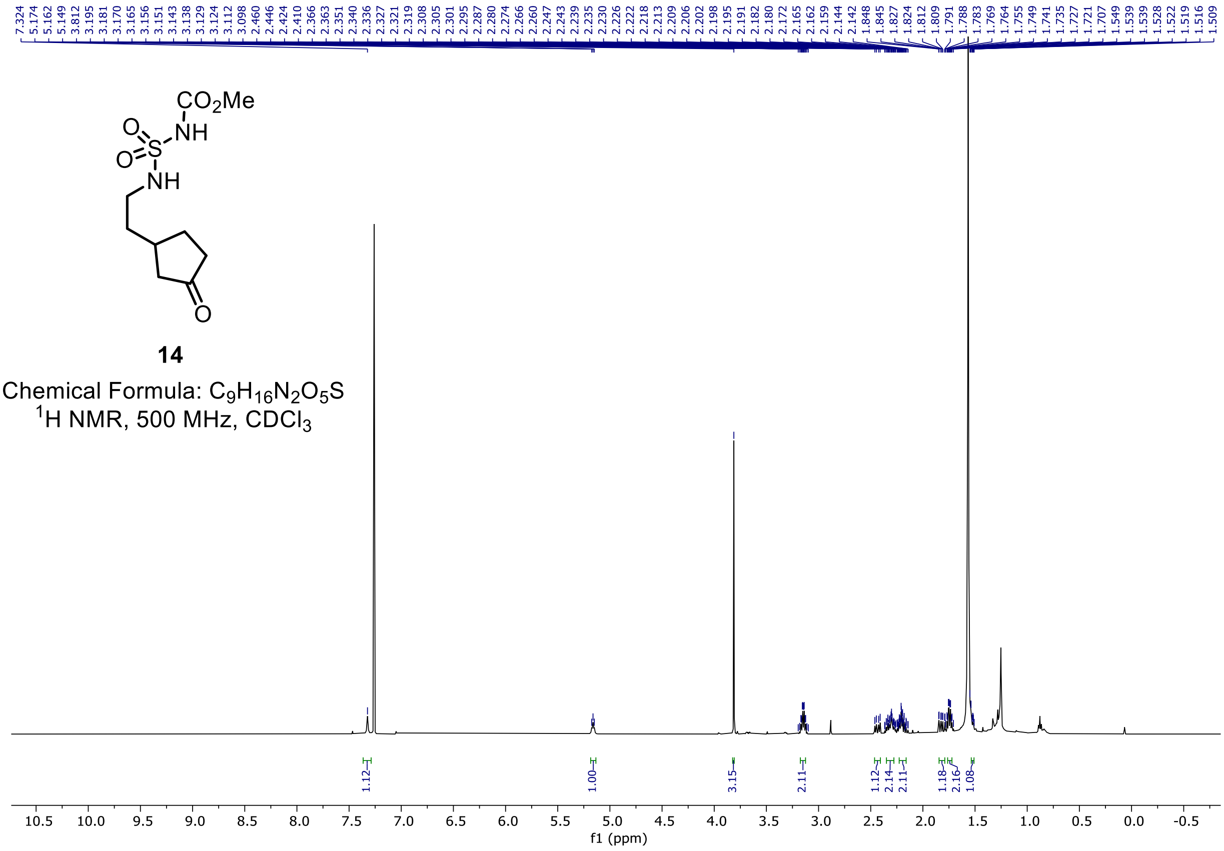

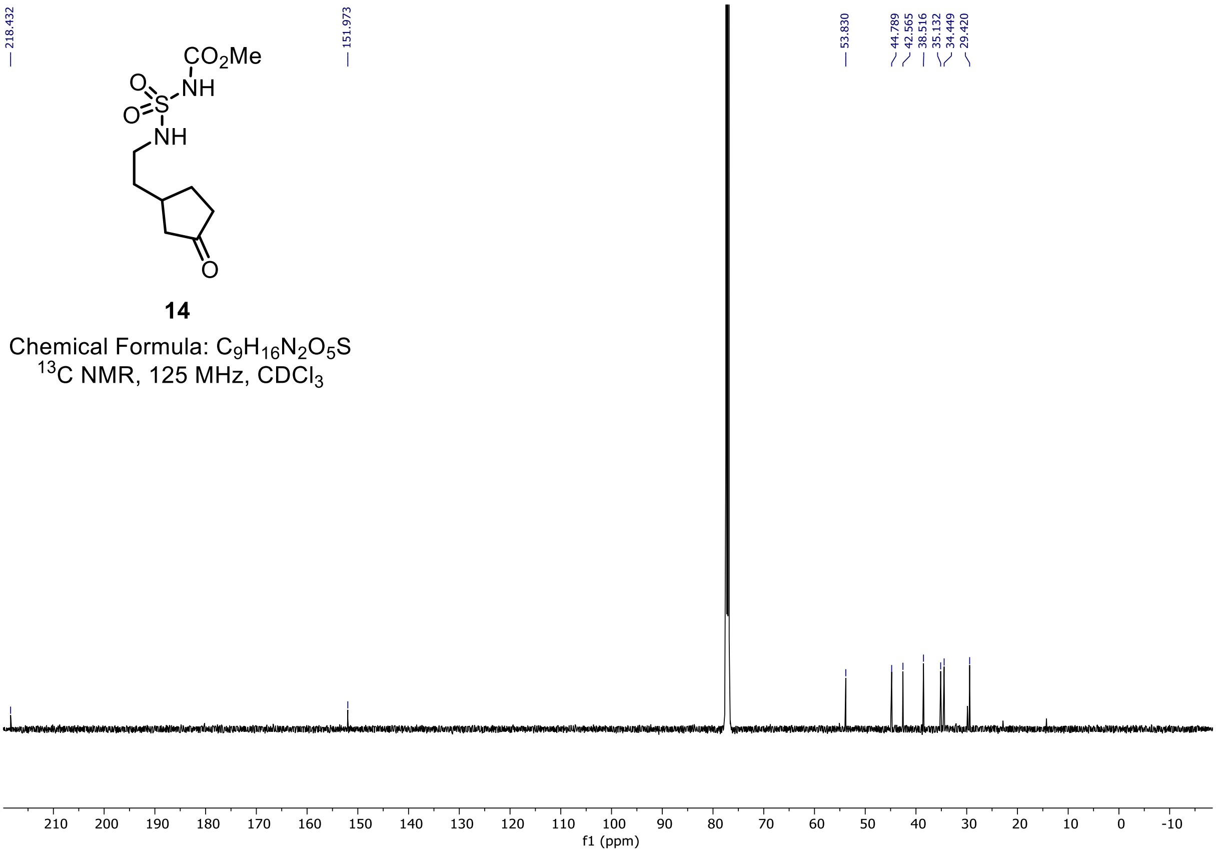

218.432
151.973
53.830
44.789
42.565
38.516
35.132
34.449
29.420
CO2Me
NH
O
S
O
NH
O
14
Chemical Formula: C9H16N2O5S
13C NMR, 125 MHz, CDCl3
210 200 190 180 170 160 150 140 130 120 110 100 90 80 70 60 50 40 30 20 10 0 -10
f1 (ppm)

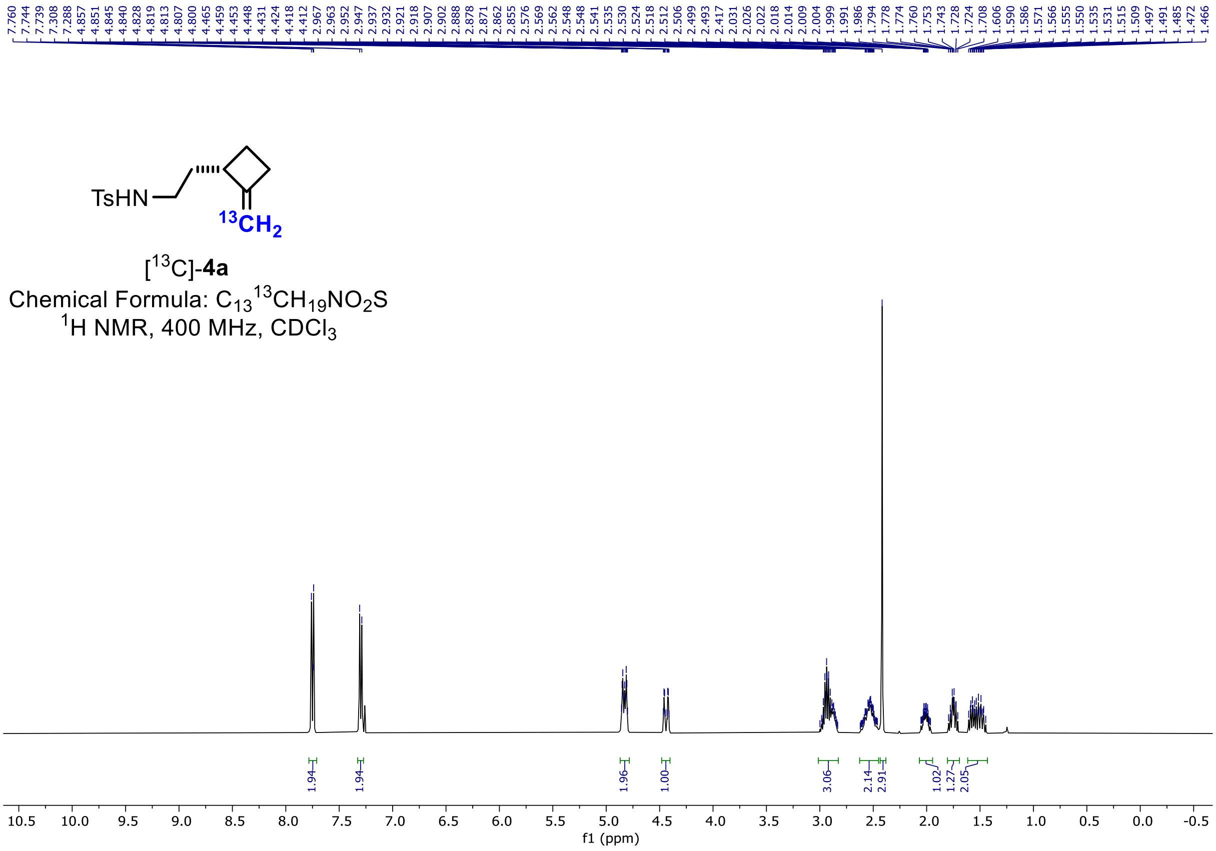

7.760 7.744 7.739 7.308 7.288 4.857 4.851 4.845 4.840 4.828 4.819 4.813 4.807 4.800 4.465 4.459 4.453 4.448 4.431 4.424 4.418 4.412 2.967 2.963 2.952 2.947 2.937 2.932 2.921 2.918 2.907 2.902 2.888 2.878 2.871 2.862 2.855 2.576 2.569 2.562 2.548 2.548 2.541 2.535 2.530 2.524 2.518 2.512 2.506 2.499 2.493 2.417 2.031 2.026 2.022 2.018 2.014 2.009 2.004 1.999 1.991 1.986 1.794 1.778 1.774 1.760 1.753 1.743 1.728 1.724 1.708 1.606 1.590 1.586 1.571 1.566 1.555 1.550 1.535 1.531 1.515 1.509 1.497 1.491 1.485 1.472 1.466
TsHN
13CH2
[13C]-4a
Chemical Formula: C13 13CH19NO2S
1H NMR, 400 MHz, CDCl3
1.94 1.94 1.96 1.00 3.06 2.14 2.91 1.02 1.27 2.05
10.5 10.0 9.5 9.0 8.5 8.0 7.5 7.0 6.5 6.0 5.5 5.0 4.5 4.0 3.5 3.0 2.5 2.0 1.5 1.0 0.5 0.0 -0.5
f1 (ppm)

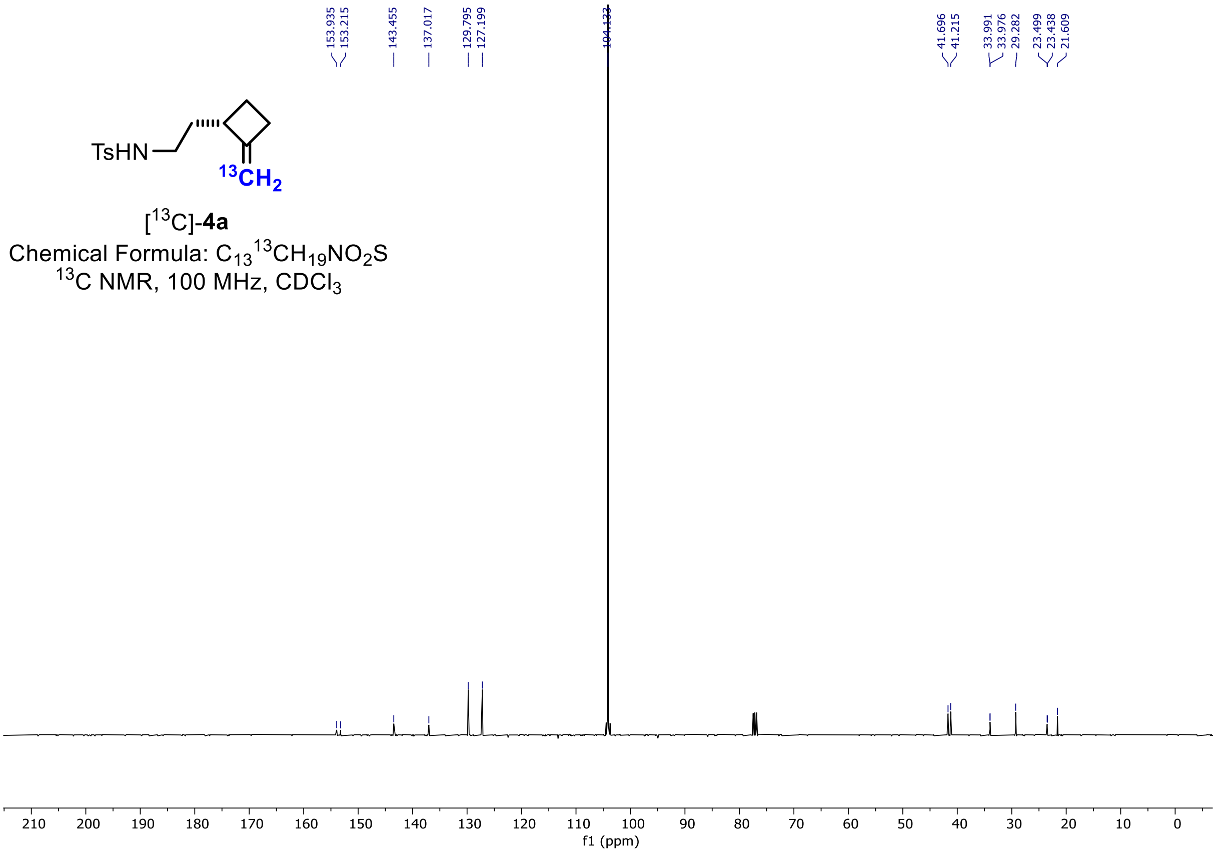
TsHN
13CH2
[13C]-4a
Chemical Formula: C13 13CH19NO2S
13C NMR, 100 MHz, CDCl3
153.935
153.215
143.455
137.017
129.795
127.199
104.133
41.696
41.215
33.991
33.976
29.282
23.499
23.438
21.609
210 200 190 180 170 160 150 140 130 120 110 100 90 80 70 60 50 40 30 20 10 0
f1 (ppm)

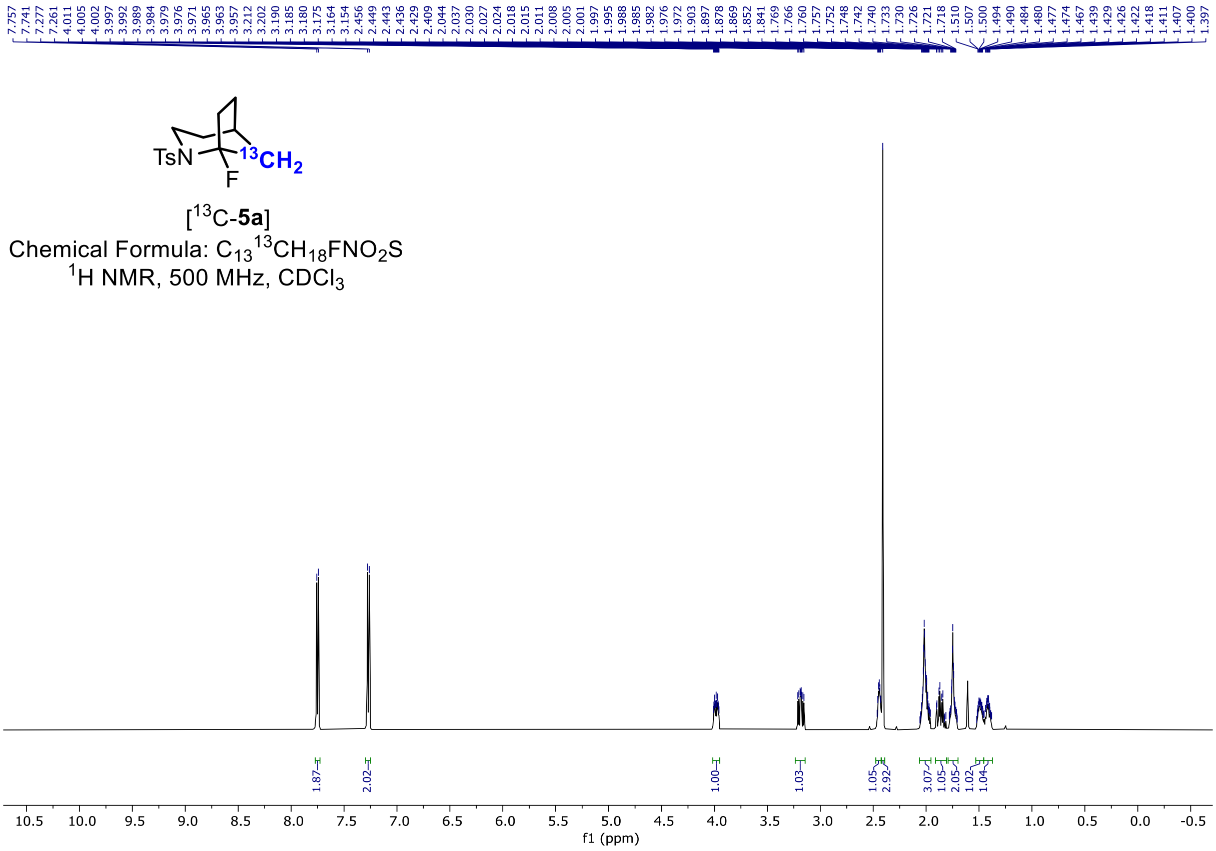
7.757
7.741
7.277
7.261
4.011
4.005
4.002
3.997
3.992
3.989
3.984
3.979
3.976
3.971
3.965
3.963
3.957
3.212
3.202
3.190
3.185
3.180
3.175
3.164
3.154
2.456
2.449
2.443
2.436
2.429
2.409
2.044
2.037
2.030
2.027
2.024
2.018
2.015
2.011
2.008
2.005
2.001
1.997
1.995
1.988
1.985
1.982
1.976
1.972
1.903
1.897
1.878
1.869
1.852
1.841
1.769
1.766
1.760
1.757
1.752
1.748
1.742
1.740
1.733
1.730
1.726
1.721
1.718
1.510
1.507
1.500
1.494
1.490
1.484
1.480
1.477
1.474
1.467
1.439
1.429
1.426
1.422
1.418
1.411
1.407
1.400
1.397
TsN
F
13CH2
[13C-5a]
Chemical Formula: C13 13CH18FNO2S
1H NMR, 500 MHz, CDCl3
1.87
2.02
1.00
1.03
1.05
2.92
3.07
1.05
2.05
1.02
1.04
10.5 10.0 9.5 9.0 8.5 8.0 7.5 7.0 6.5 6.0 5.5 5.0 4.5 4.0 3.5 3.0 2.5 2.0 1.5 1.0 0.5 0.0 -0.5
f1 (ppm)

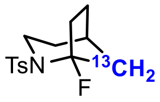

[^{13}C-**5a**]

Chemical Formula: $C_{13}{}^{13}CH_{18}FNO_2S$

^{13}C NMR, 150 MHz, $CDCl_3$

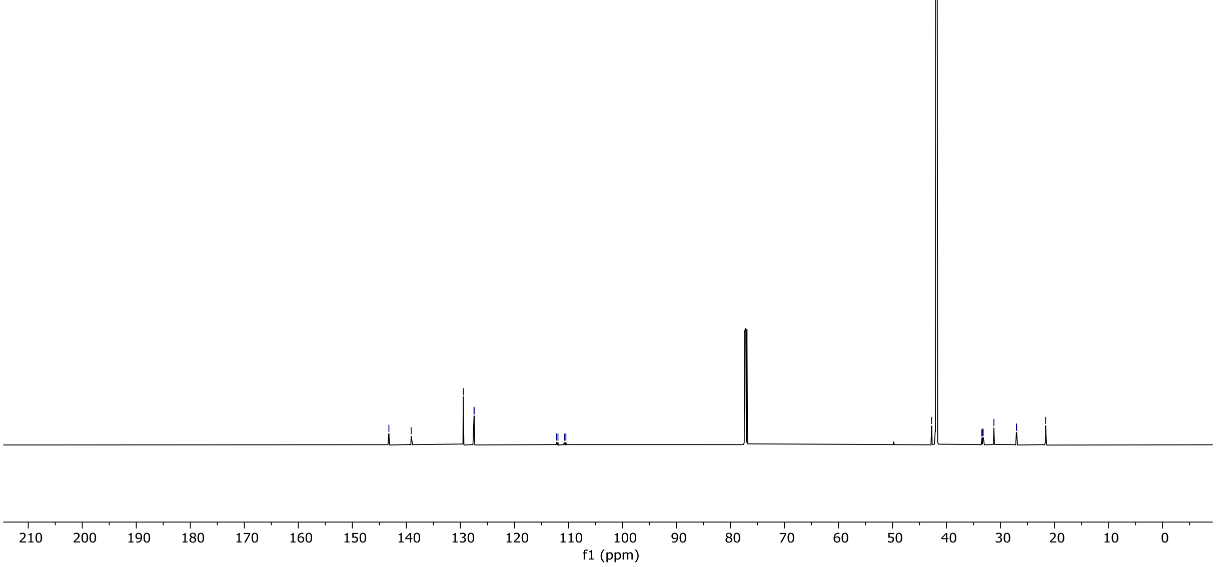

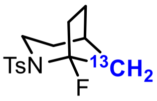

[^{13}C-**5a**]

Chemical Formula: $C_{13}{}^{13}CH_{18}FNO_2S$

^{19}F NMR, 376 MHz, $CDCl_3$

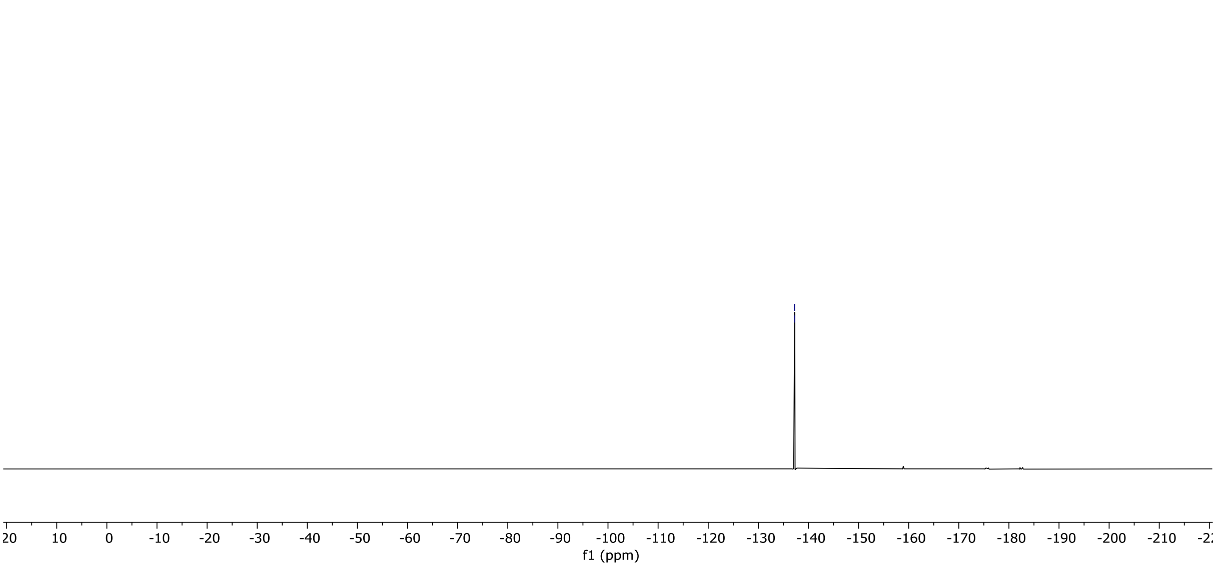

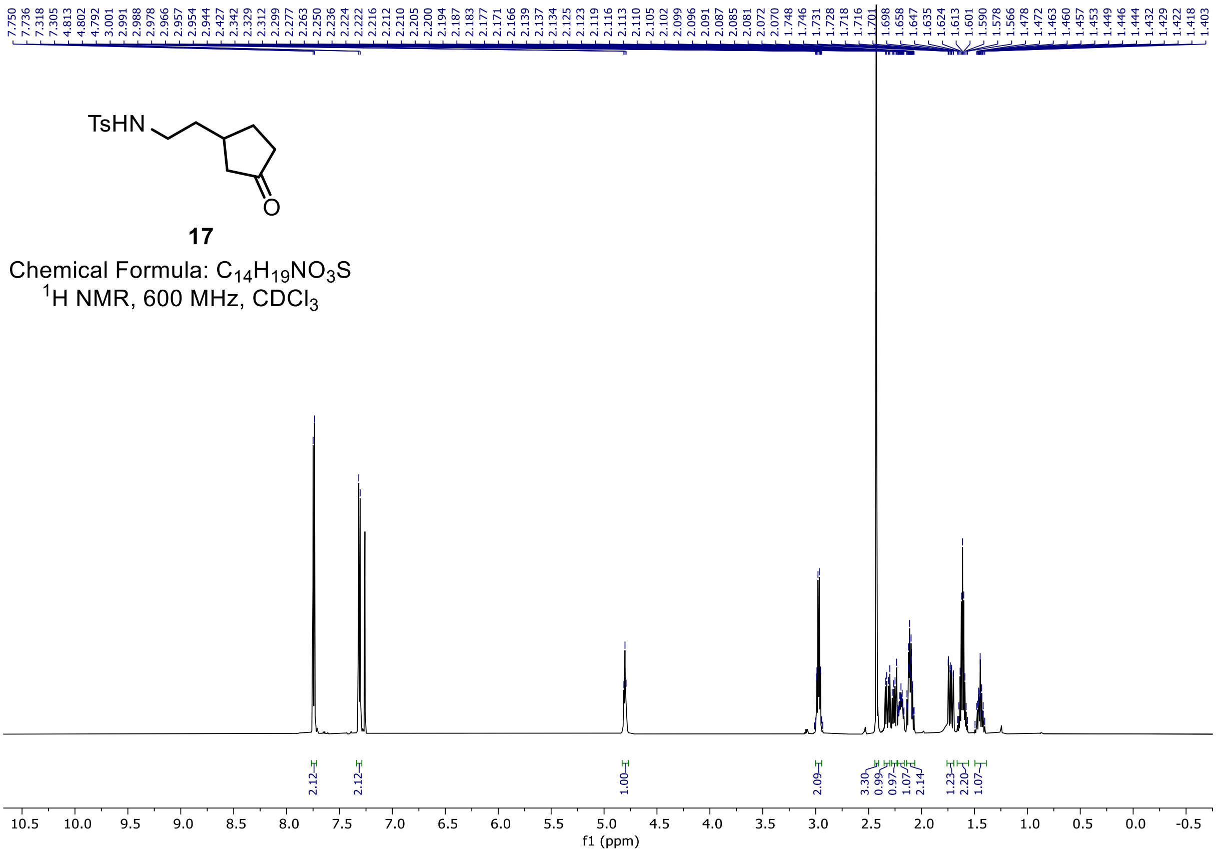
7.750
7.736
7.318
7.305
4.813
4.792
3.001
2.991
2.988
2.978
2.966
2.957
2.954
2.944
2.427
2.342
2.329
2.312
2.299
2.277
2.263
2.250
2.236
2.224
2.222
2.216
2.212
2.210
2.205
2.200
2.194
2.187
2.183
2.177
2.171
2.166
2.139
2.137
2.134
2.125
2.123
2.119
2.116
2.113
2.110
2.105
2.102
2.099
2.096
2.091
2.087
2.085
2.081
2.072
2.070
1.748
1.746
1.731
1.728
1.718
1.716
1.701
1.698
1.658
1.647
1.635
1.624
1.613
1.601
1.590
1.578
1.566
1.478
1.472
1.463
1.460
1.457
1.453
1.449
1.446
1.444
1.432
1.429
1.422
1.418
1.403
TsHN
O
17
Chemical Formula: $C_{14}H_{19}NO_3S$
^{1}H NMR, 600 MHz, $CDCl_3$
2.12
2.12
1.00
2.09
3.30
0.99
0.97
1.07
2.14
1.23
2.20
1.07
10.5 10.0 9.5 9.0 8.5 8.0 7.5 7.0 6.5 6.0 5.5 5.0 4.5 4.0 3.5 3.0 2.5 2.0 1.5 1.0 0.5 0.0 -0.5
f1 (ppm)

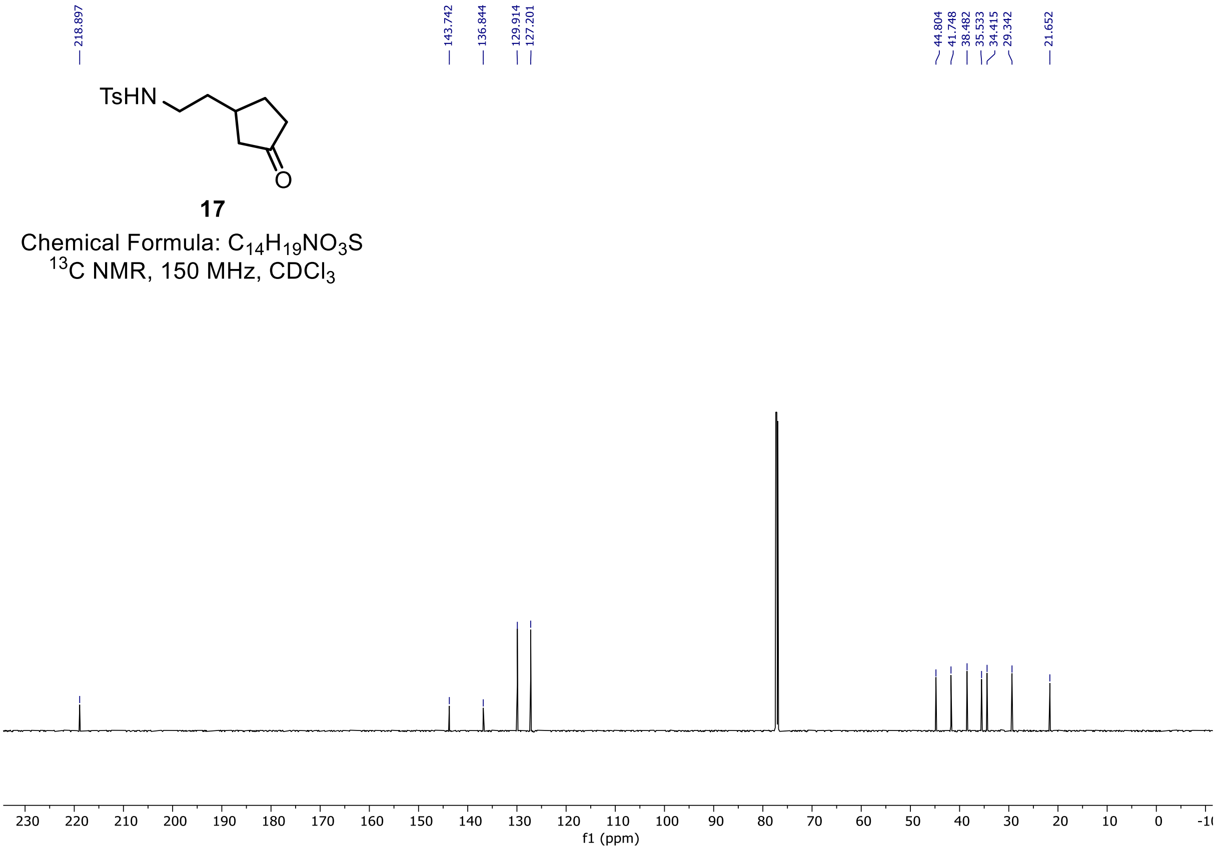
218.897
143.742
136.844
129.914
127.201
44.804
41.748
38.482
35.533
34.415
29.342
21.652
TsHN
O
17
Chemical Formula: $C_{14}H_{19}NO_3S$
^{13}C NMR, 150 MHz, $CDCl_3$
230 220 210 200 190 180 170 160 150 140 130 120 110 100 90 80 70 60 50 40 30 20 10 0
f1 (ppm)

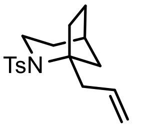

18

Chemical Formula: $C_{17}H_{23}NO_2S$

^{1}H NMR, 600 MHz, $CDCl_3$

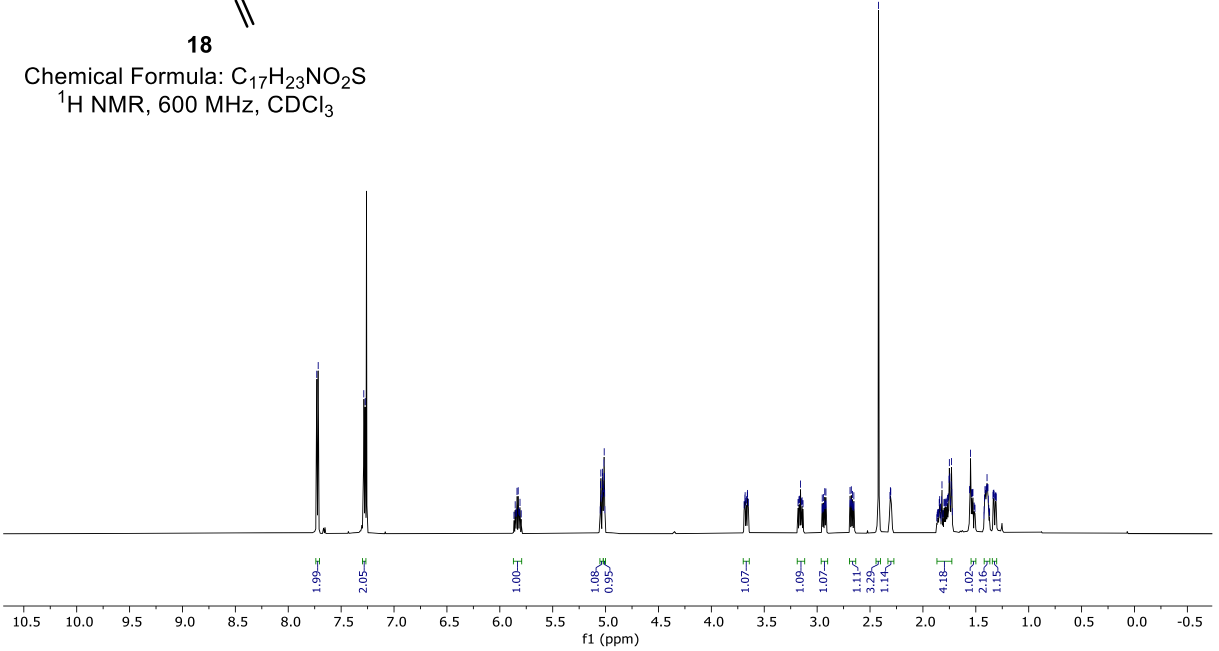

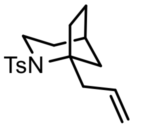

18

Chemical Formula: $C_{17}H_{23}NO_2S$

^{13}C NMR, 150 MHz, $CDCl_3$

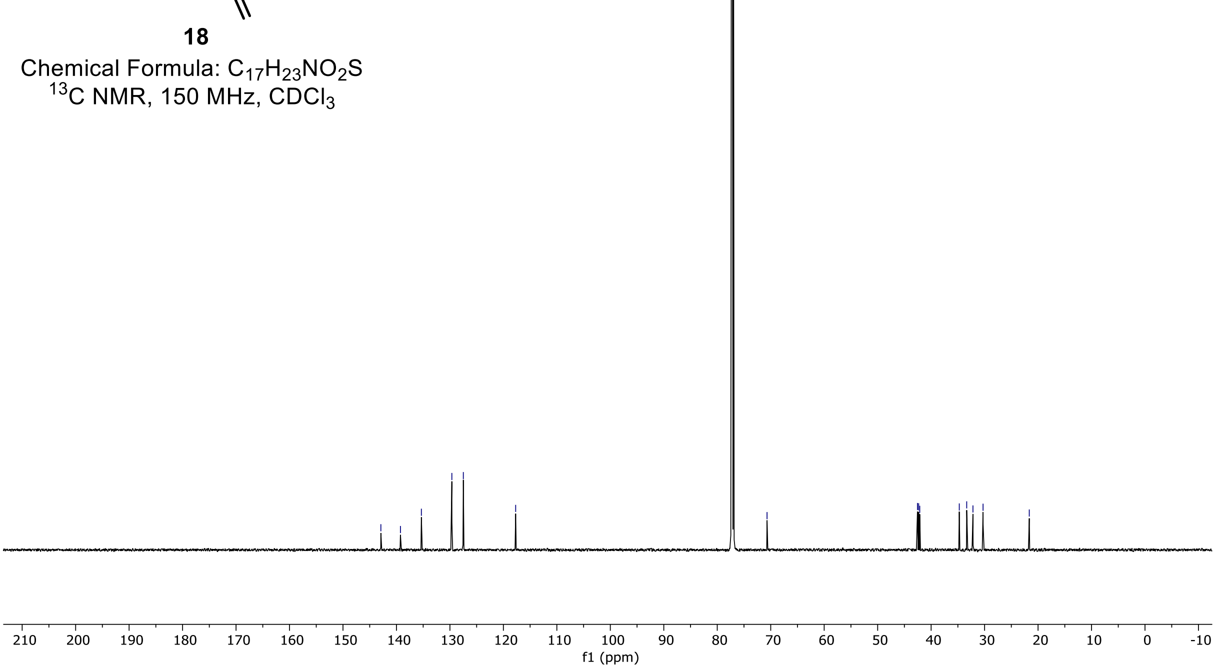

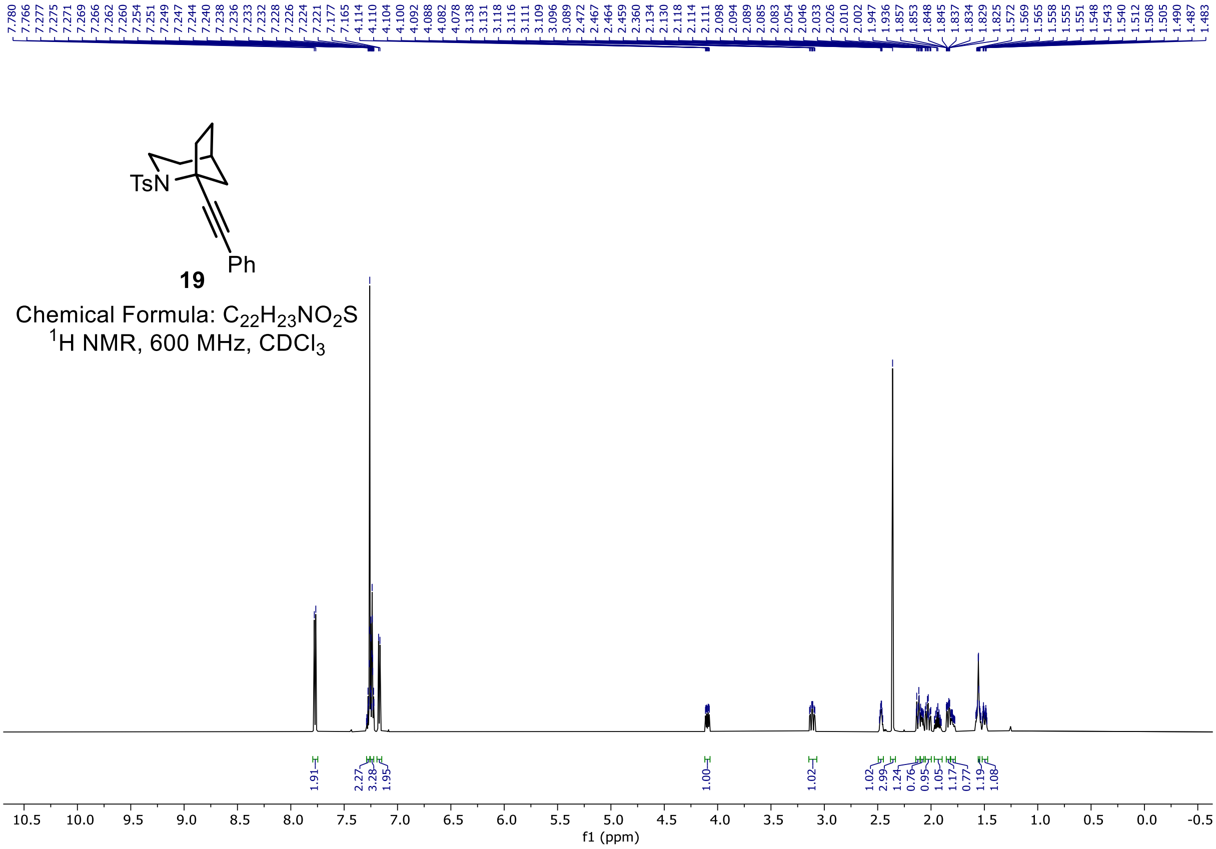
7.780 7.766 7.277 7.275 7.271 7.269 7.266 7.262 7.260 7.254 7.251 7.249 7.247 7.244 7.240 7.238 7.236 7.233 7.232 7.228 7.226 7.224 7.221 7.177 7.165 4.114 4.110 4.104 4.100 4.092 4.088 4.082 4.078 3.138 3.131 3.118 3.116 3.111 3.109 3.096 3.089 2.472 2.467 2.464 2.459 2.360 2.134 2.130 2.118 2.114 2.111 2.098 2.094 2.089 2.085 2.083 2.054 2.046 2.033 2.026 2.010 2.002 1.947 1.936 1.857 1.853 1.848 1.845 1.837 1.834 1.829 1.825 1.572 1.569 1.565 1.558 1.555 1.551 1.548 1.543 1.540 1.512 1.508 1.505 1.490 1.487 1.483
TsN
Ph
19
Chemical Formula: $C_{22}H_{23}NO_2S$
^{1}H NMR, 600 MHz, $CDCl_3$
1.91 2.27 3.28 1.95 1.00 1.02 1.02 2.99 1.24 0.76 0.95 1.05 1.17 0.77 1.19 1.08
10.5 10.0 9.5 9.0 8.5 8.0 7.5 7.0 6.5 6.0 5.5 5.0 4.5 4.0 3.5 3.0 2.5 2.0 1.5 1.0 0.5 0.0 -0.5
f1 (ppm)

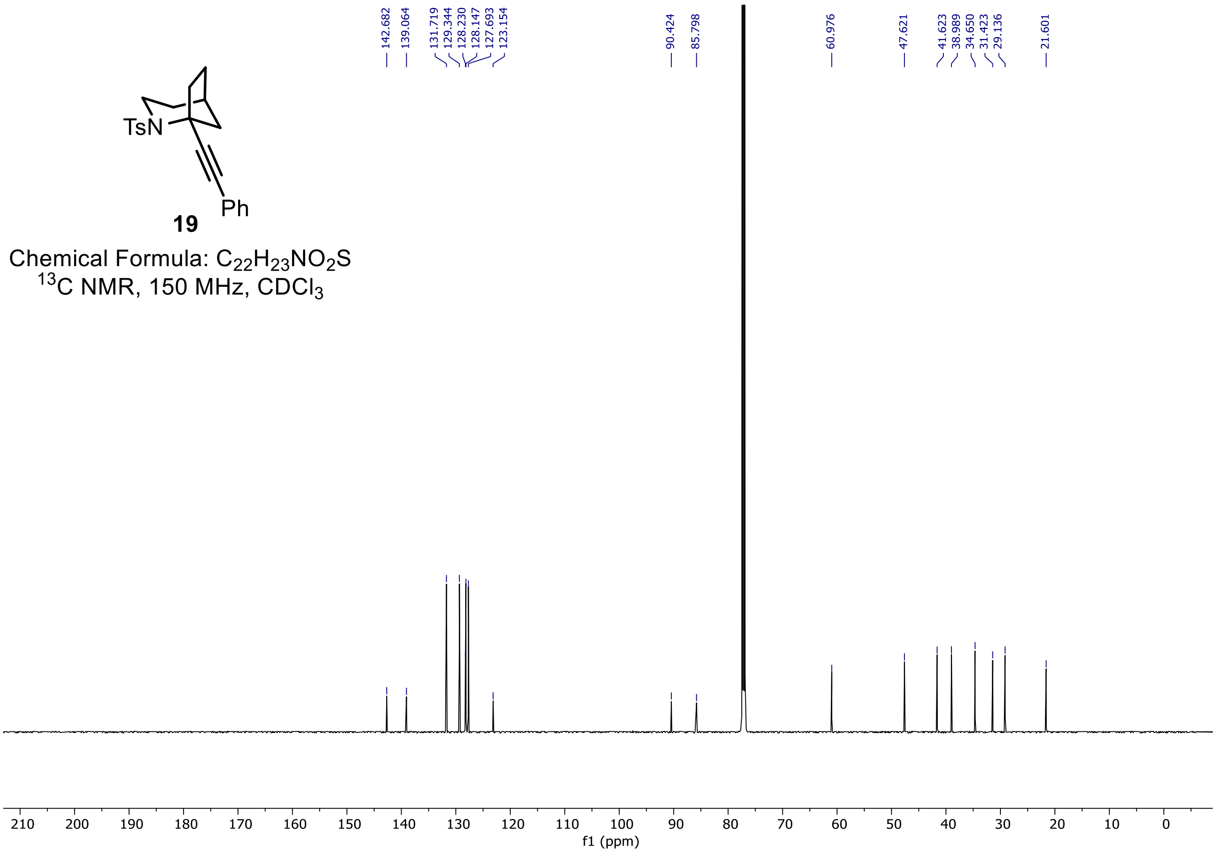
142.682 139.064 131.719 129.344 128.230 128.147 127.693 123.154 90.424 85.798 60.976 47.621 41.623 38.989 34.650 31.423 29.136 21.601
TsN
Ph
19
Chemical Formula: $C_{22}H_{23}NO_2S$
^{13}C NMR, 150 MHz, $CDCl_3$
210 200 190 180 170 160 150 140 130 120 110 100 90 80 70 60 50 40 30 20 10 0
f1 (ppm)

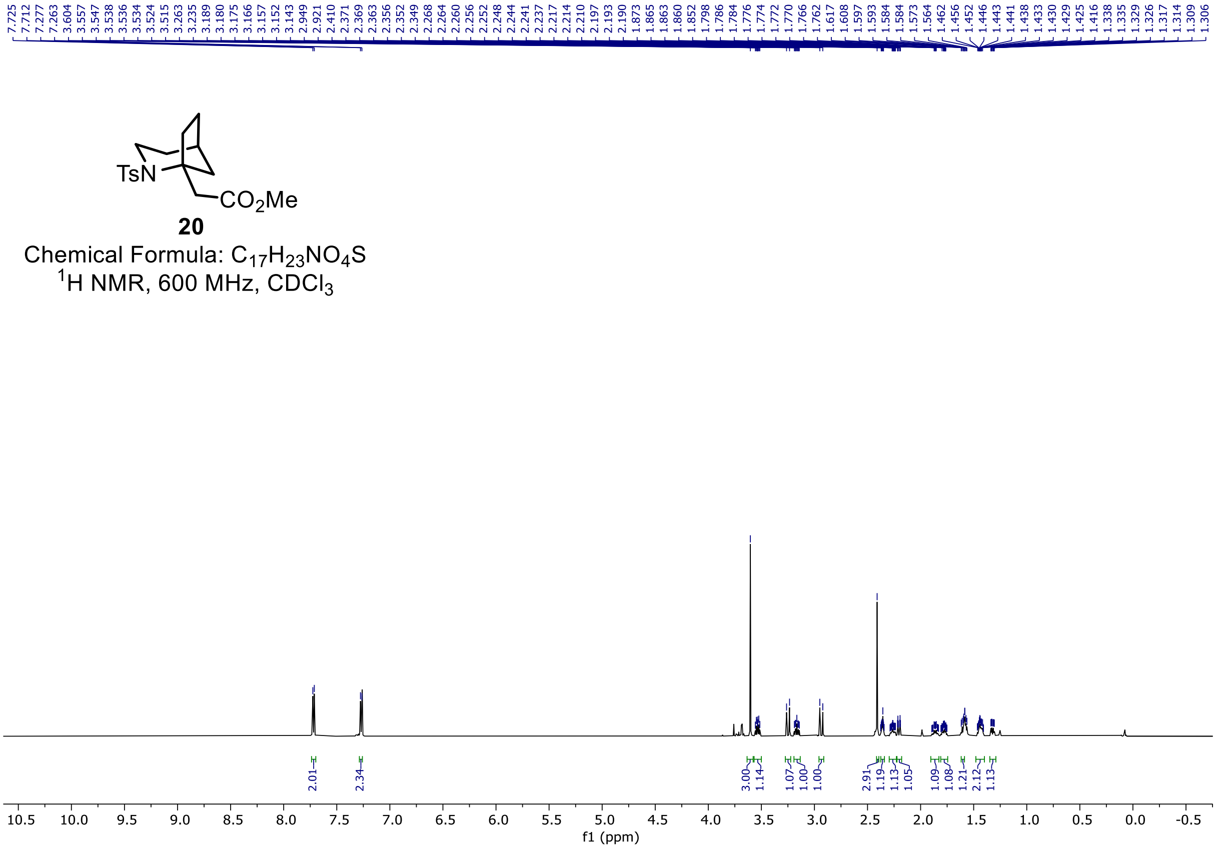
7.725 7.712 7.277 7.263 3.604 3.557 3.547 3.538 3.536 3.534 3.524 3.515 3.263 3.235 3.189 3.180 3.175 3.166 3.157 3.152 3.143 2.949 2.921 2.410 2.371 2.369 2.363 2.356 2.352 2.349 2.268 2.264 2.260 2.256 2.252 2.248 2.244 2.241 2.237 2.217 2.214 2.210 2.197 2.193 2.190 1.873 1.865 1.863 1.860 1.852 1.798 1.786 1.784 1.776 1.774 1.772 1.770 1.766 1.762 1.617 1.608 1.597 1.593 1.584 1.584 1.573 1.564 1.462 1.456 1.452 1.446 1.443 1.441 1.438 1.433 1.430 1.429 1.425 1.416 1.338 1.335 1.329 1.326 1.317 1.314 1.309 1.306
TsN
CO2Me
20
Chemical Formula: C17H23NO4S
1H NMR, 600 MHz, CDCl3
2.01 2.34 3.00 1.14 1.07 1.00 1.00 2.91 1.19 1.13 1.05 1.09 1.08 1.21 2.12 1.13
10.5 10.0 9.5 9.0 8.5 8.0 7.5 7.0 6.5 6.0 5.5 5.0 4.5 4.0 3.5 3.0 2.5 2.0 1.5 1.0 0.5 0.0 -0.5
f1 (ppm)

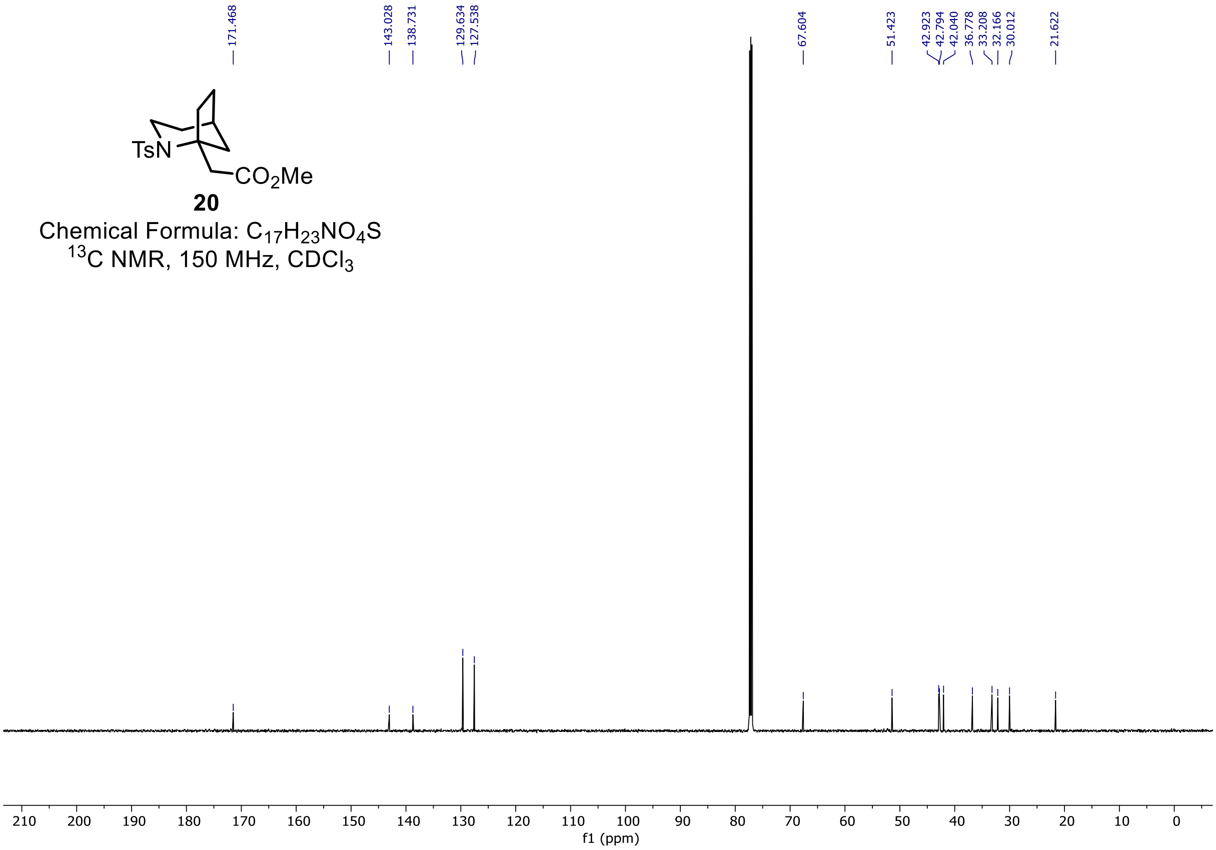
171.468
143.028 138.731
129.634 127.538
67.604
51.423
42.923 42.794 42.040 36.778 33.208 32.166 30.012
21.622
TsN
CO2Me
20
Chemical Formula: C17H23NO4S
13C NMR, 150 MHz, CDCl3
210 200 190 180 170 160 150 140 130 120 110 100 90 80 70 60 50 40 30 20 10 0
f1 (ppm)

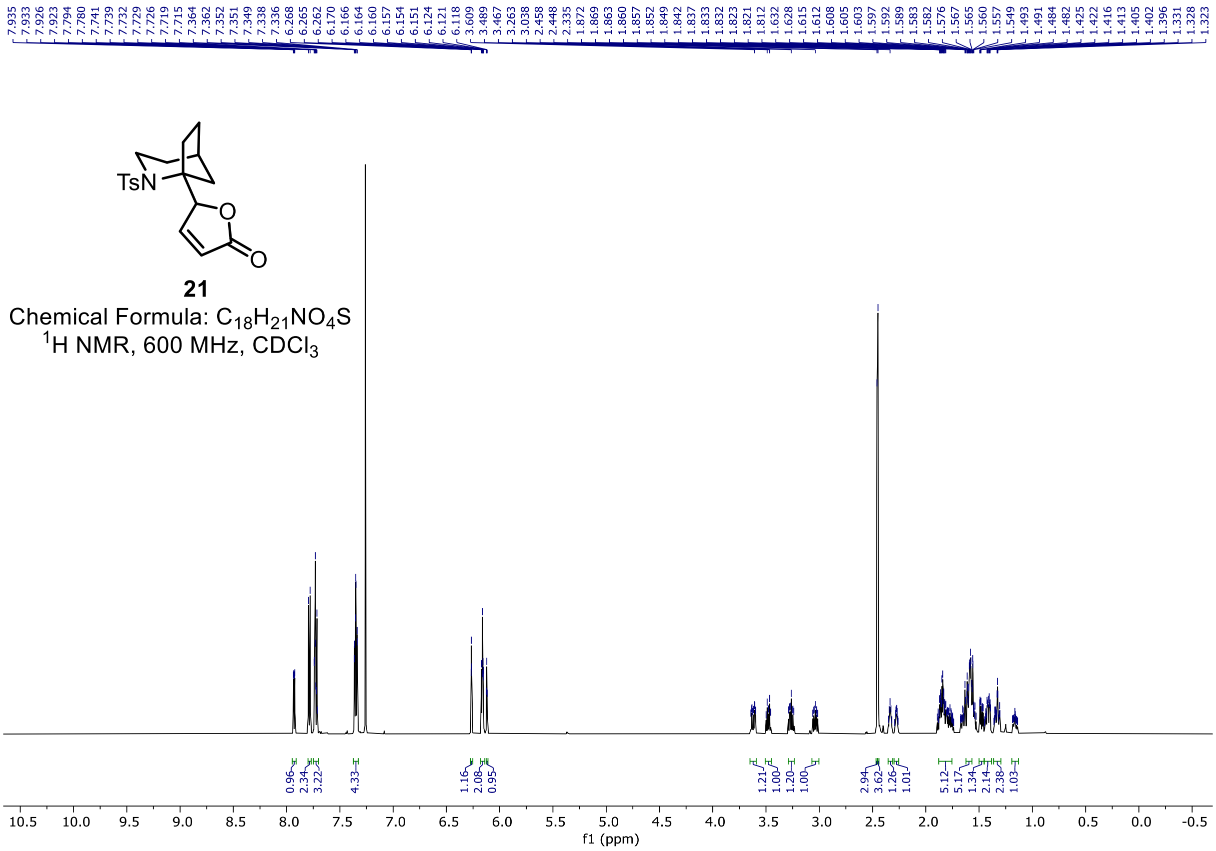

21

Chemical Formula: $C_{18}H_{21}NO_4S$

^1H NMR, 600 MHz, $CDCl_3$

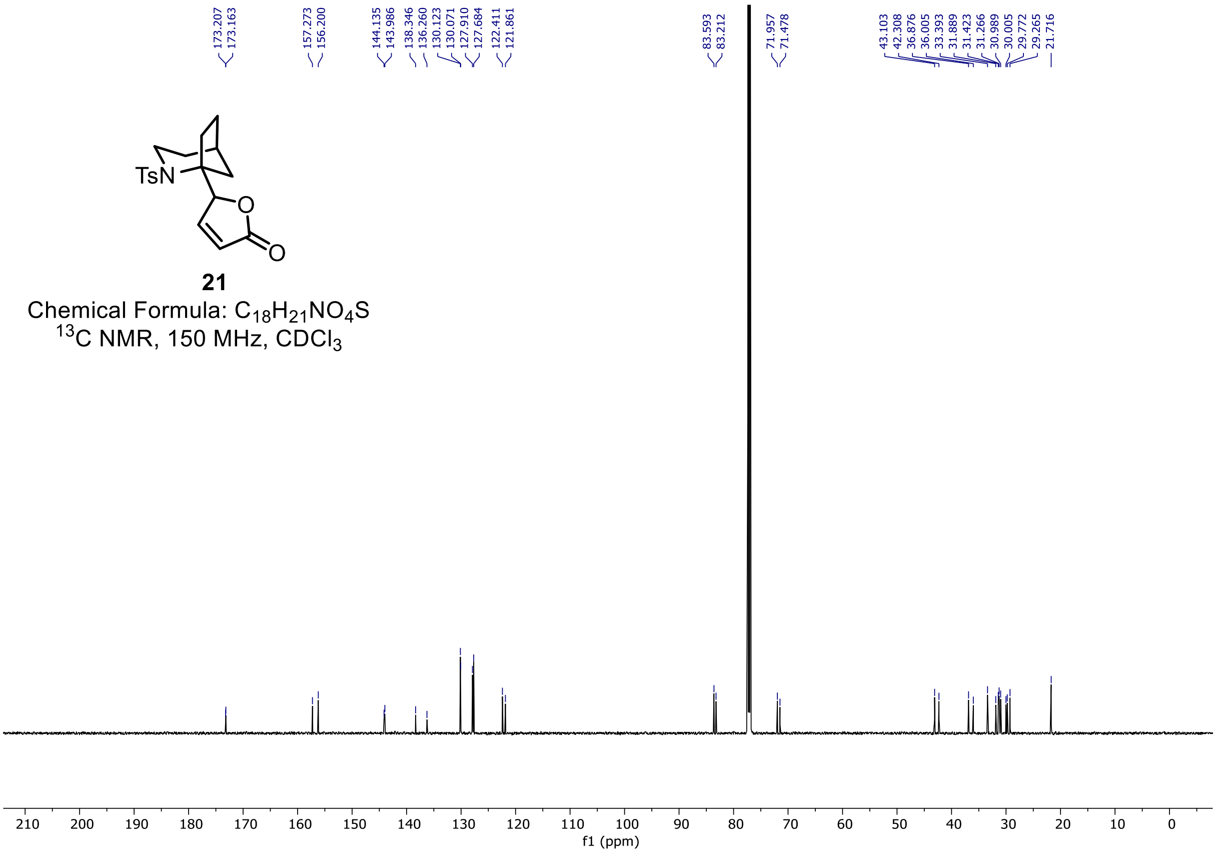

21

Chemical Formula: $C_{18}H_{21}NO_4S$

^{13}C NMR, 150 MHz, $CDCl_3$

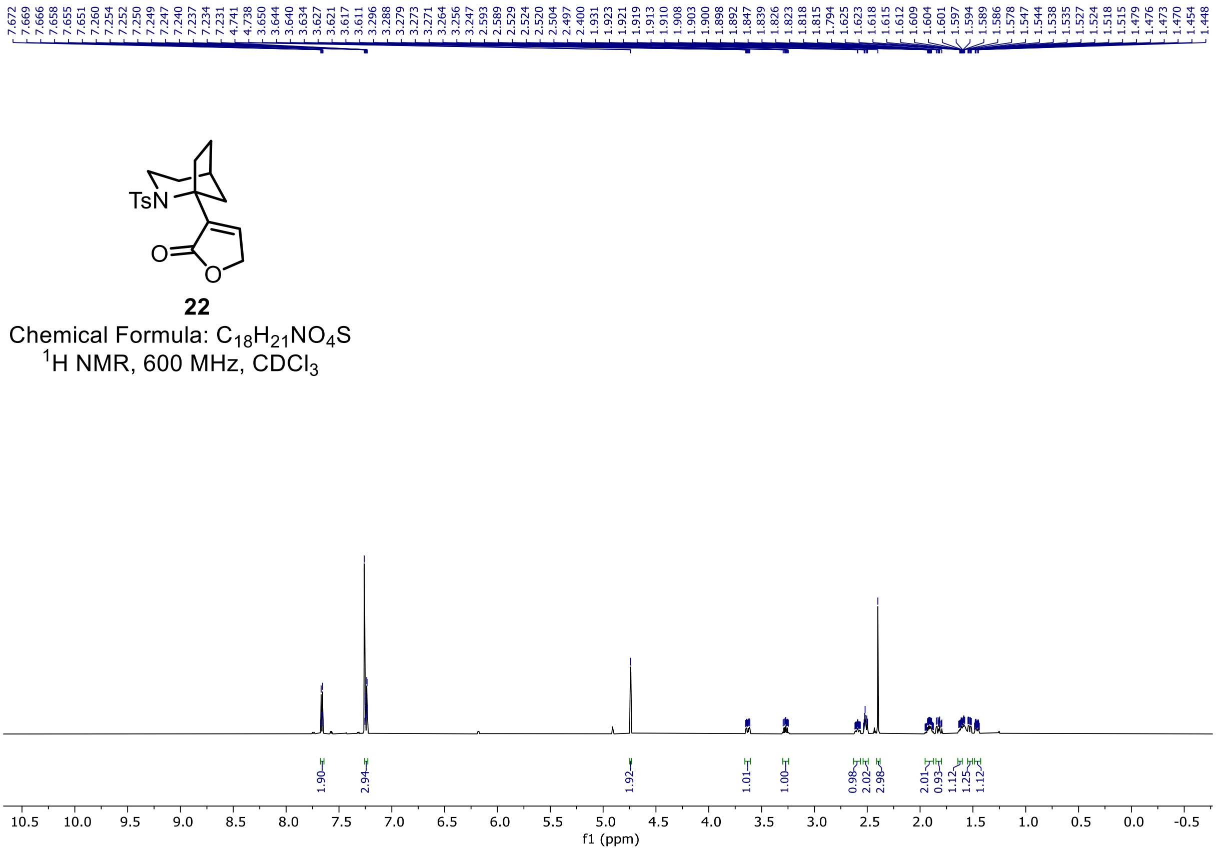

22
Chemical Formula: $C_{18}H_{21}NO_4S$
^{1}H NMR, 600 MHz, $CDCl_3$

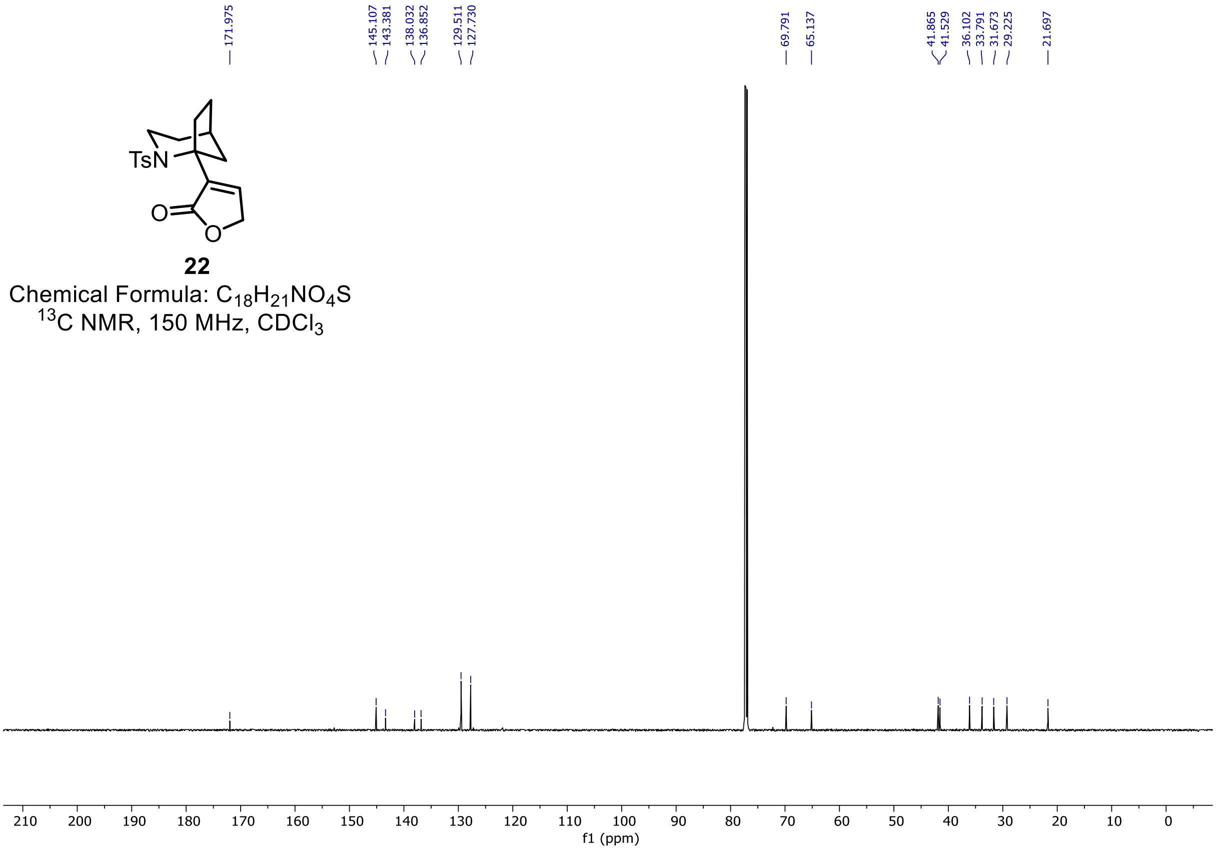

22
Chemical Formula: $C_{18}H_{21}NO_4S$
^{13}C NMR, 150 MHz, $CDCl_3$

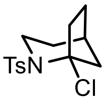

23

Chemical Formula: $C_{14}H_{18}ClNO_2S$

^{1}H NMR, 600 MHz, $CDCl_3$

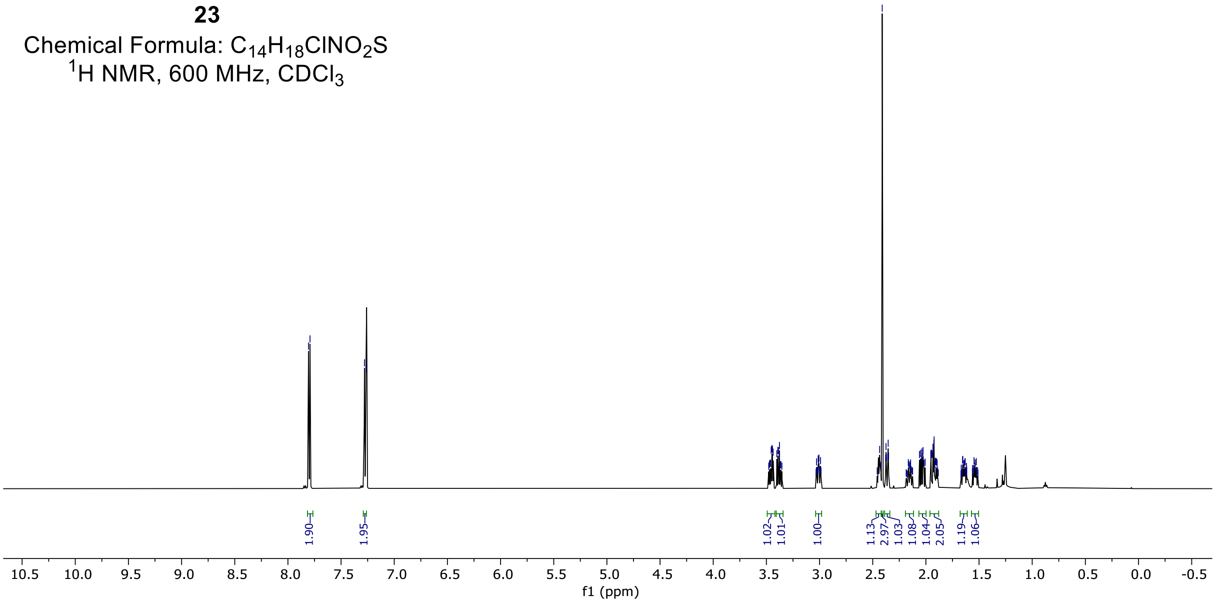

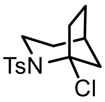

23

Chemical Formula: $C_{14}H_{18}ClNO_2S$

^{13}C NMR, 150 MHz, $CDCl_3$

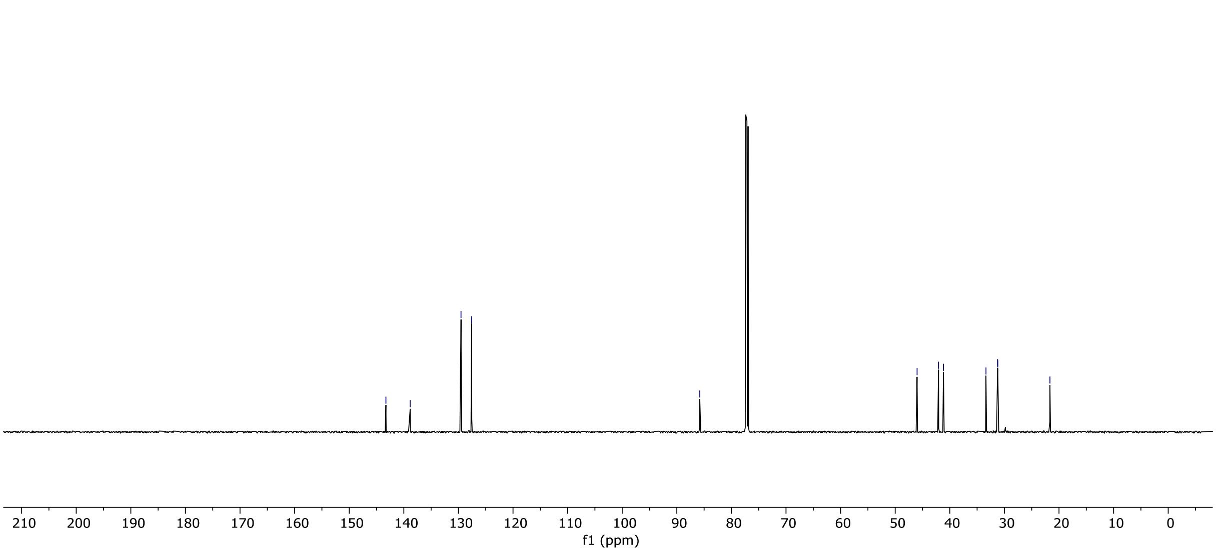

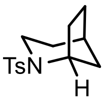

24

Chemical Formula: $C_{14}H_{19}NO_2S$

^1H NMR, 800 MHz, $CDCl_3$

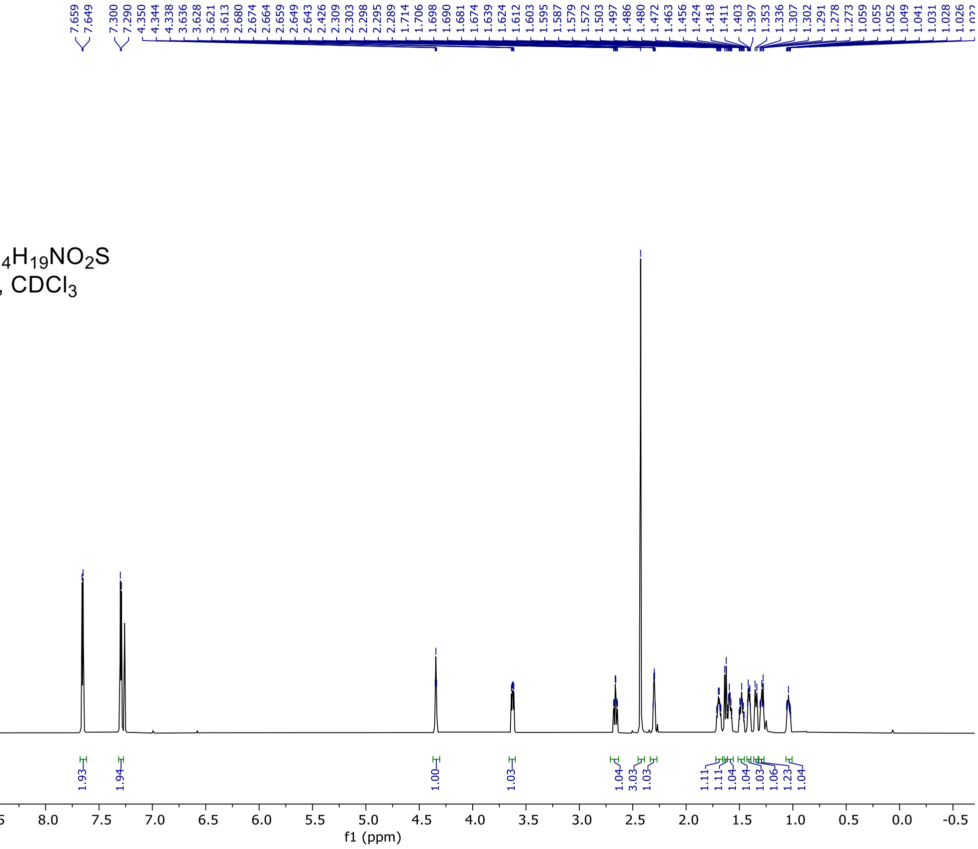

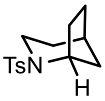

24

Chemical Formula: $C_{14}H_{19}NO_2S$

^{13}C NMR, 200 MHz, $CDCl_3$

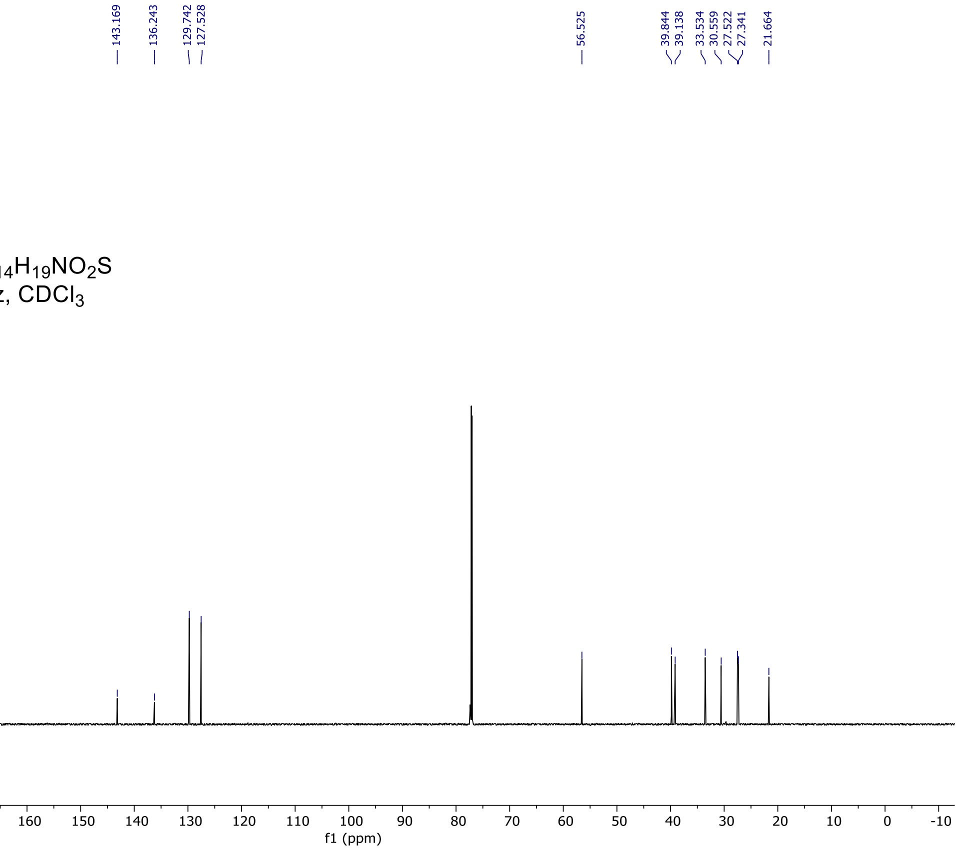

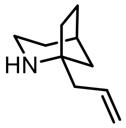

25

Chemical Formula: $C_{10}H_{17}N$

^{1}H NMR, 600 MHz, $CDCl_3$

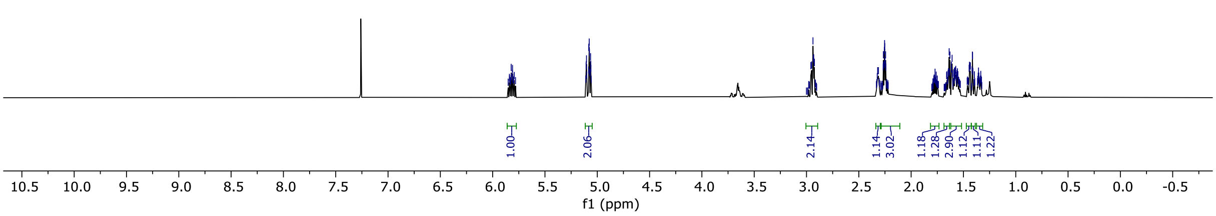

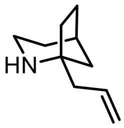

25

Chemical Formula: $C_{10}H_{17}N$

^{13}C NMR, 150 MHz, $CDCl_3$

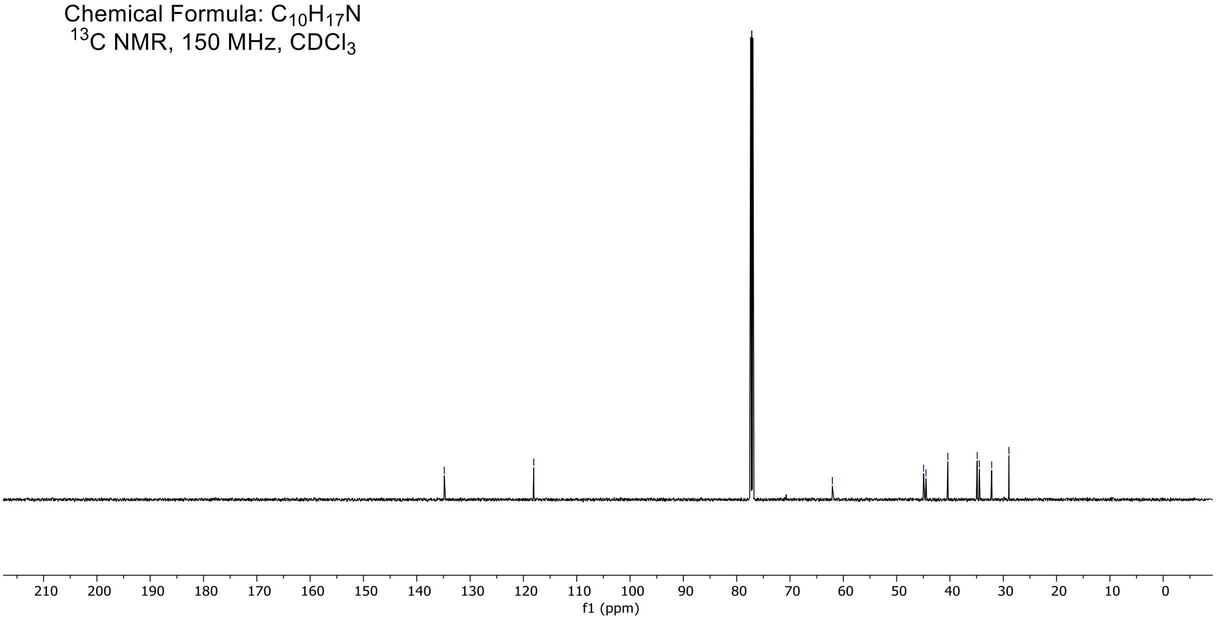

IX. References

1. Hedley, S. J.; Moran, W. J.; Price, D. A.; Harrity, J. P. A. *J. Org. Chem.* **2003**, *68*, 4286−4292.

2. Speck, K.; Magauer, T. *Chem. Eur. J.* **2017**, *23*, 1157−1165.

3. Chen, C.; Dugan, T. R.; Brennessel, W. W.; Weix, D. J.; Holland, P. L. *J. Am. Chem. Soc.* **2014**, *136*, 945−955.

4. (a) Enders, D.; Janeck, C. F.; Raabe, G. *Eur. J. Org. Chem.* **2000**, 3337-3345; (b) Zarbin, P. H. G.; de Oliveira, A. R. M.; Delay, C. E. *Tetrahedron Lett.* **2003**, *44*, 6849–6851.

5. Feng, H.-X.; Wang, Y.-Y.; Chen, J.; Zhou, L. *Adv. Synth. Catal.* **2015**, *357*, 940−944.

6. Zhou, X.; Dong, G. *J. Am. Chem. Soc.* **2015**, *137*, 13715−13721.

7. Ali, S. I.; Nikalje, M. D.; Sudalai, A. *Org. Lett.* **1999**, *1*, 705−707.

8. Pak, C. S.; Kim, T. H.; Ha, S. J. *J. Org. Chem.* **1998**, *63*, 10006−10010.

9. López, M. M.; Jamey, N.; Pinet, A.; Figadère, B.; Ferrié, L. *Org. Lett.* **2021**, *23*, 1626−1631.

10. Wang, S.-G.; Liu, X.-J.; Zhao, Q.-C.; Zheng, C.; Wang, S.-B.; You, S.-L. *Angew. Chem. Int. Ed.* **2015**, *54*, 14929−14932.

11. Yuan, C.-P.; Xie, Z.-Z.; Zheng, Y.; He, J.-T.; Guan, J.-P.; Chen, H.-B.; Xiang, H.-Y.; Chen, K.; Yang. H. *Chem. Commun*. **2023**, *59*, 10125−10128.
